# Design and synthesis of 3,4-*seco*-Lupane triterpene derivatives: targeting tumor angiogenesis and inducing apoptosis in triple-negative breast cancer

**DOI:** 10.3389/fchem.2025.1630939

**Published:** 2025-07-31

**Authors:** Chunyu Gao, Hongbo Teng, Wenxin Zhang, Yaru Zhao, Chunguo Cui, Zerbo Patrice, Liyan Wang, Yan Zhao

**Affiliations:** ^1^College of Chinese Medicinal Materials, Jilin Agricultural University, Changchun, Jilin, China; ^2^ International Joint Laboratory for Development of Animal and Plant Resources for Food and Medicine, Changchun, Jilin, China; ^3^Breast Surgery Department of Bethune Third Hospital, Jilin University, Changchun, Jilin, China; ^4^ Département: Biochimie / Microbiologie UFR Sciences de la Vie et de la Terre, Ouagadougou, Burkina Faso

**Keywords:** seco-lupane triterpene derivatives, triple-negative breast cancer, transcriptomics, apoptosis, angiogenesis

## Abstract

**Background:**

Due to the lack of effective treatment methods and targeted drugs, triple-negative breast cancer (TNBC) is not only difficult to treat clinically, but also has a poor prognosis for patients. This study aims to develop novel anti-TNBC drug candidates by designing 90 derivatives of 3,4-*seco*-lupane triterpene derivatives, a natural product of the genus Eleutherogenus.

**Methods:**

Firstly, 90 derivatives were synthesized and screened, and the compound I-27 showed excellent cytotoxicity (IC_50_=1.02 μM) for MDA-MB-231 cells for further activity verification. Then in vitro tests were carried out to detect the effects of the compound on the proliferation, migration, invasion and apoptosis of TNBC cells. With the help of transcriptomics, the mechanism of action was explored and verified. At the same time, its inhibitory effect on tumor volume and lung metastasis was verified through a mouse model of in vivo test, and its mechanism of action was further verified.

**Results:**

In vitro tests showed that compound I.-27 could effectively inhibit the proliferation, migration and invasion of TNBC cells, and induce apoptosis. Transcriptomic analysis revealed that it has a dual mechanism of action. On the one hand, it inhibits tumor angiogenesis through the ID1/TSP-1 pathway. On the other hand, it promotes apoptosis through the PI3K/AKT/FoxO1 signaling pathway. In vivo tests, the compound significantly reduced tumor volume and inhibited lung metastasis through mouse models. It further confirmed that ID1 is a key target for anti-tumor.

**Conclusions:**

In this study, an anti-TNBC drug with multiple mechanisms was developed from the triterpenoids of 3,4-3,4-*seco*-lupane triterpene derivatives for the first time, and the mechanism of action was clarified by combining transcriptomics, molecular docking and gene knockout technologies. Compound I-27 provides a potential breakthrough for the treatment of triple-negative breast cancer as a potential therapeutic candidate with a novel action mechanism and high potency.

## 1 Introduction

The incidence rate of breast cancer (BC) is increasing annually, making it one of the leading causes of cancer-related deaths among women globally and a significant threat to women’s health (Sedeta et al., 2023). Among BC subtypes, triple-negative breast cancer (TNBC) accounts for approximately 15%–20% of cases (Derakhshan and Reis-Filho, 2022). TNBC is characterized as a highly invasive and metastatic subtype that lacks specific targets for effective therapeutic drugs (Borri and Granaglia, 2021). Due to the absence of estrogen receptor (ER), progesterone receptor (PR), and human epidermal growth factor receptor (HER2) expression (Harborg et al., 2021), TNBC patients face poor prognoses and limited treatment options compared to other breast cancer subtypes. The lack of effective targeted therapies further exacerbates this issue (Criscitiello et al., 2021). Clinical treatments such as chemotherapy and radiotherapy may cause severe side effects, and the recurrence rate after surgical resection remains high, hindering optimal therapeutic outcomes (Vagia et al., 2020). Therefore, there is an urgent need for innovative treatment strategies to address TNBC.

The recruitment of new blood vessels provides essential nutrients for tumor growth and serves as a primary pathway for tumor cells to invade the vascular basement membrane and enter the bloodstream (intravasation) (Folkman, 2002). Although many anti-angiogenic drugs have been widely used in cancer patients (Folkman, 2007). their efficacy is often limited due to severe side effects and intrinsic resistance developed during long-term treatment (Piperdi et al., 2014). Thus, there is a critical need to develop novel safe and effective angiogenesis inhibitors. Inhibitor of differentiation 1 (ID1) acts as a distinctive negative regulator of basic helix-loop-helix (bHLH) activity. The dimerization region of ID1 protein is its helix-loop-helix domain, which exhibits high homology but significant differences outside this region. Amino acid residues in the loop region and adjacent areas play key roles in binding specificity. Notably, ID1 lacks intrinsic transcriptional activity due to the absence of an alkaline DNA-binding domain. High expression levels of ID1 in various tumor tissues indicate its oncogenic properties. Research has demonstrated that ID1 is a necessary factor for neovascularization, including tumor angiogenesis, making the study of the relationship between the ID1 gene and tumors a new research hotspot (Fei et al., 2023). Thrombospondin 1 (TSP1), an endogenous angiogenesis inhibitor and important extracellular matrix protein, is one of the key molecules regulating angiogenesis (Bornstein and Sage, 2002). Activation of Rap1 can suppress ID1 expression while inducing TSP-1 expression (Doebele et al., 2009). Furthermore, phosphorylation of PI3K/AKT activates FoxO1 (Zhang et al., 2011), a protein crucial for tumor suppression (Yang and Hung, 2011). Studies have shown that modulating the PI3K/AKT/FoxO1 signaling pathway exerts anti-breast cancer effects (Smit et al., 2016), anti-pancreatic cancer effects (Boreddy et al., 2011), anti-prostate cancer effects (Shukla et al., 2014), and anti-aging effects (Tzivion and Hay, 2011).

Natural products isolated from plants are valuable sources of anticancer drugs due to their diverse compounds with extensive biological activities (George et al., 2021). Some natural products induce apoptosis in cancer cells, inhibit angiogenesis, and suppress cancer progression and metastasis (Rahman et al., 2022). Natural products or their derivatives serve as important sources of anticancer drugs, such as paclitaxel derived from Taxus brevifolia and its derivative vincristine (Wolfender et al., 2019). In recent years, plant triterpenoids have garnered increasing attention for their anticancer potential. For example, Lantadenes extracted from Lantana camara L. exhibit broad-spectrum activity against skin cancer (Monika et al., 2023). Triterpenoid saponins (tea saponins), which are traditional Chinese medicinal active substances in *Camellia japonica* L., display cytotoxicity against MCF-7 cells (Hui et al., 2024). Betulinic acid, a pentacyclic triterpenoid found in the bark of sycamore, birch, and eucalyptus trees, shows significant potential for cancer prevention and treatment, whether used alone or in combination (Subhasis et al., 2024).

Natural products from plants of the genus *Acanthopanax Miq* in the family Araliaceae possess a variety of biological activities, including anti-inflammatory (Lee et al., 2012), antidepressant (Bian et al., 2018), anticancer (Zhang et al., 2021) effects. Among them, chiisanoside was first discovered in A. chiisanensis Nakai (Hahn et al., 1984).Chiisanoside has a series of significant pharmacological effects, such as anti-osteoporosis, anti-platelet aggregation (Choi et al., 2008), inhibition of angiogenesis (Bian et al., 2017), and antitumor (Yook et al., 1996). Previous research has shown that the preparation process of triterpenoid saponins from the leaves of *Acanthopanax sessiliflorus* and its main active ingredient chiisanoside was established and improved, and it was found to have good anticancer effects. At the same time, further studies have shown that the 3,4-*seco*-lupane triterpene chiisanogenin is significantly superior to the triterpenoid saponin (chiisanoside) with a glycosyl group in terms of anticancer activity (Wang et al., 2022b).

To develop clinically more active drugs and enhance the utilization of plant resources from the genus Acanthopanax, chiisanogenin and compound MH were used as lead compounds for design and synthesis in this study. The most active compound, compound Ⅰ-27 (IC_50_ = 1.02 μM), was identified. Based on the cytotoxic activity of compound Ⅰ-27 on the MDA-MB-231 cell line, its effects on proliferation, migration, and invasion were verified through clone formation experiments, Hoechst33258/PI apoptosis experiments, EDU cell proliferation experiments, Transwell invasion experiments, and cell scratch migration experiments. Transcriptomic analysis and *in vivo* mouse experiments confirmed that compound Ⅰ-27 inhibits tumor angiogenesis and induces TNBC cell apoptosis via the ID1/TSP-1 and PI3K/AKT/FoxO1 signaling pathways. Our findings suggest that compound Ⅰ-27 holds promise as a targeted drug for treating TNBC generation and metastasis.

## 2 Materials and reagents

Dulbecco’s Modified Eagle’s Medium (DMEM), RPMI-1640 medium, and fetal bovine serum (FBS) were purchased from Gibco (Grand Island, NY, USA). Rabbit monoclonal antibodies against ID1, TSP-1, PI3K, p-PI3K, AKT, p-AKT, FoxO1, and β-actin were obtained from Wanlei Life Sciences Co., Ltd. (Shenyang, China). 3-(4,5-dimethyl)-2,5-diphenyl tetrazolium bromide (MTT), 5-ethynyl-2′-deoxyuridine (EDU), Matrigel, Crystal Violet Staining Solution, Hoechst 33342/PI Apoptosis Assay Kit, PrimeScript™ RT kit, TRIzol reagent, streptomycin, and penicillin G sodium were acquired from Thermo Fisher Scientific Inc. (Waltham, MA, USA). The BCA protein assay kit was purchased from Solabio (Beijing, China).

Human breast cancer cell lines (MCF-7, MDA-MB-231, SKBR-3), human hepatocellular carcinoma cell line (HepG2), human prostate cancer cell line (PC-3M), human pancreatic cancer cell line (PANC-1), mouse breast cancer cell line (4T1), and human non-small cell lung cancer cell line (A549) were purchased from Procell Life Science & Technology Co., Ltd. (Wuhan, China). All cell lines were authenticated by short tandem repeat (STR) genotyping to confirm their origin and purity. High-quality genomic DNA was extracted from the tested cell lines, and PCR amplification was performed on multiple preselected STR loci using specific primers. This approach increased the number of tested STR loci and enhanced detection sensitivity. The PCR products were subjected to capillary electrophoresis, where DNA fragments were separated into distinct bands based on their sizes, forming an electropherogram. Finally, the electropherogram was analyzed to determine the corresponding allele numbers at each STR locus. The STR genotyping results demonstrated that all cell lines exhibited a match rate of >90% with the standard cell lines in the ATCC and DSMZ databases (Vittoria et al., 2017).

### 2.1 Synthesis methods and routes of lead compounds

Preparation and identification of chiisanoside: First, choose the leaves of *A. sessiliflorus* (PE 02044945). The leaves of *A. sessiliflorus* (Rupr. and Maxim.) Seed, provided by Lisheng Biologics Co., Ltd. (Jilin China), were identified by Professor Zhang Lianxue. After ultrasonicating the leaves collected at the end of July with a 75% ethanol solution for 24 h (leaves: ethanol = 1:8). Filter the leaves with nylon cloth and concentrate under reduced pressure for later use. D101 macroporous resin was used as the stationary phase, and a 10%–50% ethanol solution was used as the mobile phase for gradient elution. The 50% ethanol solution was collected and concentrated under reduced pressure to obtain crude saponins with a yield of 7%–8%. Finally, using 200–300 mesh silica gel as the stationary phase and Dichloromethane/methanol (6:1, 3:1) solution as the mobile phase for gradient elution, collect the 3:1 component solution, concentrate under reduced pressure to dryness, and obtain Chiisanoside with a yield of 75%. Chiisanoside identified by HPLC and HRMS, HRMS Calcd for C_48_H_74_O_19_ [M + H]^+^: 954.48241; found: 954.48354. (Hyun-Jae et al., 2022).

Preparation of chiisanogenin: Dissolve Chiisanoside in 0.25 mol/L NaOH solution and heat the reaction at 95° C for 2 h. Evaporate to dryness, add 20% citric acid solution, heat and react for 4 h, filter the reaction solvent under reduced pressure until neutral, obtain white powder, and obtain the precursor compound chiisanogenin. Chiisanogenin identified by HPLC and HRMS, HRMS Calcd for C_30_H_44_O_5_ [M + H]^+^: 484.31891; found: 484.31623.

Preparation of compound MH: Dissolve Chiisanoside in a 3 mol/L hydrochloric acid methanol solution (hydrochloric acid: methanol = 1:4). Heat and reflux at a temperature of 80°C for 4 h, let it dry in a cool place, and finally purify the product by column chromatography using 300–400 mesh silica gel as the stationary phase and dichloromethane/methanol (60 : 1) as the mobile phase to obtain the lead compound MH. Compound MH identified by HPLC HRMS Calcd for C_31_H_48_O_6_ [M + H]^+^: 516.3451 1; found: 516.345683.

### 2.2 Synthesis methods and routes of derivative

Synthesis of intermediates: Dissolve chiisanogenin/compound MH in acetone solution (solid-liquid ratio = 1:20), add a small amount of potassium carbonate, and then add 1,3-dibromopropane/1,4-dibromobutane/1,5-dibromopentane/1,6-dibromohexane/1,10-dibromodecane (molar ratio to raw material is 5:1), heat the reaction at 95° C reflux 24 h. Separate and purify the intermediate in 300–400 mesh silica gel chromatography (mobile phase system is dichloromethane methanol), and vacuum concentration.

Amino protection: Catalysts were used in the experimental operation, and the specific steps are as follows. Dissolving benzyloxycarbonyl chloride (molar ratio of 1:3 to raw material), potassium carbonate, 5-methoxytryptamine/5-hydroxytryptamine/2-thiophenamine in dichloromethane, stirring them for 12 h, washing them with diluted hydrochloric acid, and drying the organic layer with anhydrous sodium sulfate. After concentrating the mixture under reduced pressure to dryness, the purified product was obtained using 300–400 mesh silica gel as the stationary phase and dichloromethane/methanol (40:1) as the mobile phase. The obtained product was dissolved in an appropriate amount of dichloromethane, and 4-Dimethylaminopyridine (DMAP) and then ET_3_N (2 eq) were slowly added at 0°C. Finally, (Boc) _2_O (1 eq) was added and heated to room temperature for reaction. After 12 h of reaction, the mixture was concentrated under reduced pressure to dryness, dichloromethane and sodium chloride solution were added for extraction, and the phases were separated. The organic phase was dried with anhydrous sodium sulfate and concentrated, and then purified by column chromatography to obtain the product. The product was dissolved in methanol and then 10% PD/C was also added in. The above solution was reacted with H_2_ for 6 h at room temperature. Then the catalyst in the reaction product was filtered out. Finally, the filtered reaction product was concentrated under reduced pressure to dryness to obtain the amino-protected raw material. Please review the manuscript.

### 2.3 Animals

BALB/C mice (Female, 6 weeks old), were purchased from Yisi Laboratory Animal Technology Co., Ltd. (China Changchun, SCXK-2022–0001). All animal experiments were conducted in accordance with the principles approved by the Animal Ethics Committee of Jilin Agricultural University (China Changchun, 2023-KJT-021).

### 2.4 Cell culture

MCF-7, MDA-MB-231, SKBR-3, HepG2, PC -3M, PANC-1 were cultured in DMEM high glucose medium, 4T1 and A549 were cultured in RPMI-1640 medium containing 10% FBS, 100 U/mL penicillin, and 100 μg/mL streptomycin. containing 10% fetal bovine serum (FBS), and cultured in a humidified incubator containing 5% CO_2_ at 37°C (Ronald et al., 2015).

### 2.5 Cytotoxicity assays

The MTT assay detects the growth rate of test cell lines by evaluating the linear relationship between cell activity (through mitochondrial activity) and absorbance to quantitatively determine cytotoxic activity. Changes in the number of MDA-MB-231, SKBR-3, MCF-7, PC-3M, HepG2, and PANC-1 cells were determined by a colorimetric MTT assay. After inoculating all cell lines used at a density of 1 × 10^5^/mL for 24 h, they were treated with derivatives, DOX, DOC, gemcitabine, lapatinib (100, 50, 25, 12.5, 6.25, 3.125 μM) with or without for 48 h. Subsequently, 100 μL of MTT (5 mg/ml) was introduced into each well. Then place the well plate in the cell culture box and let it stand for 3 h. After that, Remove MTT solution and add 150 μL of DMSO to dissolve the blue purple formazan crystals. Due to the photosensitivity of MTT, after the culture plate is placed in the dark at 37°C for 15–30 min, measure its light absorption value at 570 nm wavelength. The IC_50_ extract concentration value that kills 50% of the cells is calculated using Excel (Seabra et al., 2023).

### 2.6 Colony formation

MDA-MB-231 cells (1 × 10^3^/well) were inoculated in a 6-well plate. After 24 h, they were treated with medium with or without compound Ⅰ-27 (0.5, 1.0, 2.0 μM) for 7 days, and the formation of colonies was observed. During the 7 days, replace the complete culture medium every 3 days. Then add 4% paraformaldehyde fixative to each well, pour out after 10 min, add Himedia Laboratories staining solution and let it stand for 20 min. Take photos through a microscope,counted using ImageJ software (Linyu et al., 2024).

### 2.7 EDU cell proliferation assay

EDU is used to detect cell proliferation. After MDA-MB-231 cells (1 × 10^6^/mL) were inoculated in a six-well plate, cultured for 24 h, they were treated with medium with or without compound Ⅰ-27 (0.125, 0.25, 0.50, 1.00, 2.00 μM) for 24 or 48 h. Then fresh medium containing 50 μM EDU was used to replace the medium. After 6 h of incubation, add 0.5 mL of PBS containing 4% paraformaldehyde to each well, incubate for 30 min, and discard the fixative. Wash the cells twice with PBS, each time for 5 min; And after adding a PBS solution containing 0.3% Triton X-100 at room temperature for 30 min, discard the permeabilization solution and wash it with washing solution. Add 100 μ L of Click reaction solution to each test tube and incubate at room temperature in the dark for 30 min. Discard the Click reaction solution, add washing solution for 5 min and discard, repeat 3 times. Then, add 200 μ L of Hoechst 33,342 solution to each well and incubate at room temperature in the dark for 20 min. After adding the washing solution for 5 min, discard it and repeat the process 3 times. Take photos under a fluorescence microscope (Eve Therese et al., 2023).

### 2.8 Apoptosis assay

MDA-MB-231 cells (1 × 10^6^/mL) were inoculated in a six well plate, cultured for 24 h, they were treated with medium with or without compound Ⅰ-27 (0.5, 1.0, 2.0 μM) for 24 h. Wash cells of different drug concentrations with −4°C PBS, centrifuge at 800 rpm for 3 min, discard the supernatant, and repeat 3 times. Under dark conditions, add PI staining solution and let it stand for 30 min. The Hoechst33258 solution was diluted. 500uL of the diluted Hoechst33258 solution was taken to resuspend the cells and stained for 10 min. The cells were gently pipetted and mixed. 20uL of cells were taken with a pipette and added to a glass slide and observed under a fluorescence inverted microscope (Yunshan et al., 2022).

### 2.9 Transwell migration and scratch wound assays

MDA-MB-231 cells (1 × 10^6^/mL) were cultured in a 6-well plate for the wound healing assay. When the cell density reaches 70%–80%, use a 100 μ L pipette tip to draw a straight line in each well. Then, 1 mL of medium with or without compound Ⅰ-27 (0.125, 0.25, 0.50 μM) was added and treated for 48h. Observe the wound healing at 0h and 48h under a microscope and take photos (Rui et al., 2021).

The Transwell invasion assay was performed using a transwell chamber coated with matrigel. Pre coated with matrix gel in the upper chamber of the 24 well transwell, MDA-MB-231 cells (1 × 10^5^/mL) were introduced into the upper chamber (DMEM without FBS). Add 600 μL of DMEM containing 20% FBS to the lower chamber. After 48 h of cultivation, remove the surface cells on the membrane with a cotton swab. The cells on the surface of the membrane were fixed with 4% paraformaldehyde for 20 min, and stained overnight with Crystal Violet Staining Solution at room temperature. Count under an inverted microscope (Jing et al., 2023).

### 2.10 Transcriptome sequencing

Inoculate MDA-MB-231 cells (1 × 10^6^/mL) into a 90 mm culture dish and culture for 24 h. After treatment with medium containing or not containing compound Ⅰ-27 (0.25, 0.50, 1.00 μM) for 12, 48h, extract total RNA according to the manufacturer’s instructions for TRIzol @ reagent. RNA quantification, RNA purification, reverse transcription, library construction, and sequencing were all performed by Sangon Biotech Co., Ltd (China Shanghai) (Mary, 2012).

### 2.11 Real-time fluorescence reverse transcription polymerase chain reaction (RT-PCR) assay

Inoculate MDA-MB-231 cells into a 6-well plate and treat with or without compound Ⅰ-27 (0.25, 0.50, 1.00 μM) for 12h, 24, 48h. Extract total RNA using TRIzol reagent according to the manufacturer’s instructions and use PrimeScript™ Reverse transcription of cDNA using RT kit. Then amplify cDNA using SYBR GREEN premix and relevant primers. RT-PCR uses the following cycle curves: 95 s°C for 30s, 40 cycles including melting curves at 95°C for 5s and 60 s°C for 30 s, 95° C for 15s, 55°C for 15s, and 95 s°C for 1 s. The primer sequences are listed in Supplementary Appendix S2. Calculate phase expression levels using the 2- △△ CT method (Michael and Joseph, 2018).

### 2.12 Chick embryo chorioallantoic membrane assay

Incubate the eggs in an incubator with a temperature of 37°C and a humidity of 60% for 7 days then, inoculate MDA-MB-231 cells (1 × 10^6^/mL) into a 6-well plate and treat with or without compound Ⅰ-27 (0.5, 1.0, 2.0 μ M) for 12 h. Collect the cell supernatant and concentrate it. Seed eggs by using strong light to determine the top position of the eggshell, and create a window to add the supernatant. to add the supernatant. After sealing and incubating the egg window for 48 h, observe the angiogenesis and take photos (Shiran et al., 2019).

### 2.13 Western blot analysis

Use RIPA lysis buffer to lyse and extract total protein from cells. Measure protein concentration using BCA protein detection kit and balance. Separate proteins by SDS-PAGE electrophoresis and transfer them onto a PVDF membrane. After blocking with 5% skim milk for 2 h, a primary antibody solution was added, including ID1, TSP1, PI3K, p-PI3K, FoxO1, β - actin (all diluted at 1:1000) and AKT, p-AKT (diluted at 1:5000), and incubated overnight at 4° C. Then, the corresponding secondary antibody (diluted at 1:2000) was shaken at room temperature for 2 h. Wash with TBST three times in each step. Soak the PVDF film with developer for 30s and place it in the Analytic Jena chemiluminescence gel imaging to collect images. Using ImageJ software for analysis (Duojiao et al., 2016).

### 2.14 Molecular docking study

The three-dimensional structure of the target (forkhead box protein O1) FoxO1 (PDBID: 3CO7) was obtained from the Protein Data Bank database (https://www.rcsb.org/) To make the results more accurate, conduct more than 10 rounds of docking. Select the conformation with the highest score (lowest affinity) as the docking conformation. Use AutoDockTools-1.5.6 software for molecular docking. The docking images were visualized and analyzed using PyMOL (DeLano Scientific LLC, USA) software.2.16 Tumor growth and lung metastasis xenograft models were established.

A subcutaneous xenograft mouse model of TNBC was established. 4T1 cells (3 × 10^6^/mL) were subcutaneously inoculated into the armpit of BALB/C mice. Three days later, 0.2 mL of 4T1 cells (1 × 10^7^/mL) were subcutaneously injected into the axilla of the right forelimb of mice. When the tumor mass reached a size of 100 mm3, the mice were randomly divided into four groups, and the model group was intraperitoneally injected with the same volume of physiological saline; The doxorubicin group received intraperitoneal injection of 2 mg/kg of doxorubicin hydrochloride (DOX) (Liang et al., 2015), Compound Ⅰ-27 low-dose group was intraperitoneally injected (10 mg/kg); Compound Ⅰ-27 high-dose group intraperitoneal injection (20 mg/kg) once a day for up to 14 days. During the administration process, the tumor volume of the mice was monitored. Half of the mice were euthanized, tumors and organs were dissected, weighed, fixed, and photographed. The remaining mice were used for survival analysis. Make paraffin sections of tumors and organs, prepare for hematoxylin and eosin (H&E) staining, immunohistochemistry, and Tunel. staining analysis Continuously monitor the survival of the remaining mice. Establish a mouse model of breast tumor lung metastasis. 4T1 cells were injected intravenously at a concentration of 1 × 10^7^/mL. Grouping, administration method and time, same as subcutaneous xenograft mouse model. The lungs were removed, fixed with paraformaldehyde, and the lung tumor nodules were manually counted (Alessandro et al., 2014).

### 2.15 Histopathological examination

After ethanol gradient dehydration of the fixed tumor, the tumor tissue was placed in paraffin and left to stand for 60 min. The embedded paraffin tissue was sliced into sections with a thickness of about 4–5 mm using a paraffin slicer, and then baked in a drying oven at 60°C for 30–60 min until the paraffin melted. Finally, H&E staining was performed. Scanning was performed using a Pannoramic MDI digital slice scanner (3D Histoch Co., Ltd, Budapest, Hungary) to capture morphological images (Alotaibi et al., 2021).

### 2.16 Immunohistochemical analysis

Perform immunohistochemical staining on the melted slices, deparaffinize them, and place them in a pH 6.0 citric acid repair solution. Add H_2_O_2_ dropwise around the slices, incubate at room temperature for 20 min, and add primary antibodies (Ki67, CD31, and Tunel). Add PBS to the tissues of the control group, and store them overnight in a refrigerator at 4° C. Wash them three times with PBS, add secondary antibodies, and then place them in a 37° C incubator for half an hour. Wash the tissues three times with PBS, dry the PBS around the tissues, and add diaminobenzidine (DAB) color reagent. Finally, immerse them in hematoxylin and stain them using a Pannoramic MDI digital section scanner. Scan and capture morphological images. (Hartmut et al., 2014).

### 2.17 Transient transfection

4T1 cells were transiently transfected using Lipofectamine 2000 (11668030, Invitrogen, Wuhan, China). The sequences of KD-ID1 and negative control (WT) are: KD-ID1: AAA​AAG​GTG​AAC​GTT​CTG​CTC​TAC​GCT​CGA​GCG​TAG​AGC​AGA​ACG​TTC​ACC​T; NC: AAA​ATT​CTC​ACG​AAC​GTA​GTC​ACG​TCT​CGA​GAC​GTG​ACT​ACG​TTC​GTG​AGA​A.

### 2.18 Statistical analysis

Statistical analysis was conducted using GraphPad Prism 6.0 software (California, USA). All data are presented as mean ± standard deviation and analyzed using one-way analysis of variance (ANOVA). Statistical significance was determined through Student’s t-test. *p* < 0.05 is considered a statistically significant difference.

## 3 Results

### 3.1 Preparation of derivatives

According to the synthesis method and route, a total of 90 compounds were successfully synthesized and isolated. The structures and purity (>90%) of the target products were confirmed based on their physicochemical properties and spectral analysis (^1^HNMR, ^13^CNMR, HPLC and HRMS analysis). Detailed data are provided in the Supplementary Material.

**SCHEME 1:**
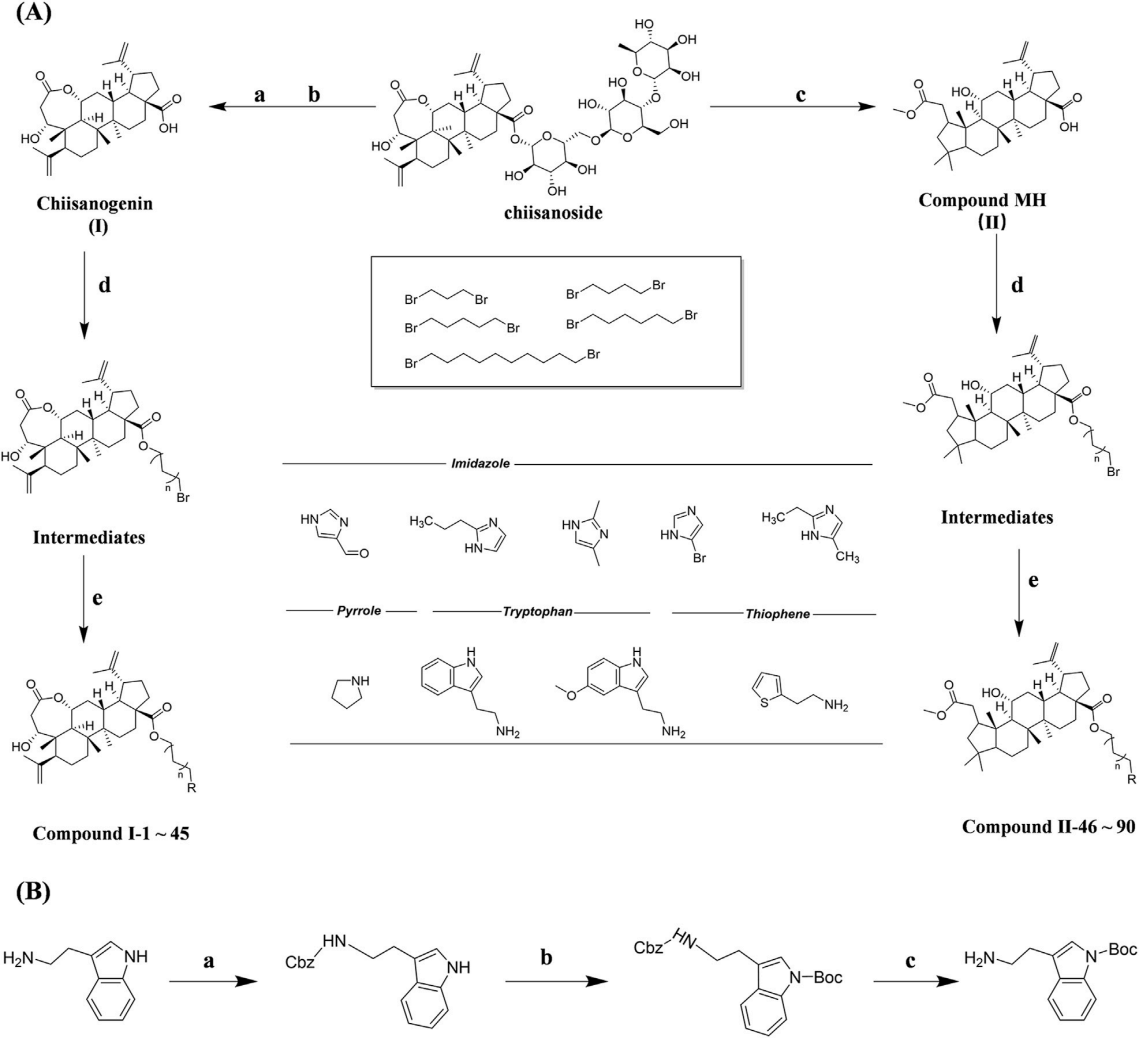
**(A)** Synthesis routes for all derivatives. a. NaOH(0.25 mol/L), MeOH, 95°C, 2 h; b. 20%Citric acid l, 95°C, 4 h; c. HCl (3 mol/L), MeOH, 80°C, reflux, 4h; d. AC, K_2_CO_3_, reflux, 24 h; e. ACN, K_2_CO_3_, reflux, 24 h.Synthesis of intermediate derivatives. **(B)** synthesis of intermediate derivatives. a. Cbz-Cl、K_2_CO_3_, stir, 12h; b. DCM, 0°C, DMAP, ET_3_N, Boc_2_O, 12h; c.MeOH, PD/C, H_2_, 6h.

Chiisanogenin exhibits more significant anti-cancer activity due to the presence of active functional groups such as hydroxyl, carboxyl, and terminal double bonds in its structure. These functional groups can be further utilized for structural modification to enhance its pharmacological properties. The preparation process is illustrated in Scheme 1A. Chiisanoside undergoes alkaline hydrolysis and acid hydrolysis to yield a substantial amount of chiisanogenin and compound MH. In previous laboratory studies, the structures of chiisanoside, chiisanogenin, and compound MH have been elucidated by nuclear magnetic resonance spectroscopy. Similar synthesis and characterization procedures were performed in this study (Wang et al., 2022a). Therefore, after extraction and preparation, we determine the purity by HPLC as shown in Supplementary Figure S1. Ten intermediates were synthesized by substitution reaction between C-28 carboxyl group of two lead compounds and dibromoalkyl group (3–6, 10 carbons). Substitution reaction of amino groups in imidazole, indole, and thiophene reactions using intermediate terminal bromine groups. Dibromoalkanes are used as intermediates between azo, amino and imino groups and lead compounds to form raw material intermediates, which are identified by HPLC as shown in Supplementary Figure S1. Since indole compounds have two azo groups and will undergo substitution reaction prior to bromoalkanes, imino protection is performed first. The preparation process is shown in Scheme 1B.

### 3.2 Toxicity of compounds on different human tumor cell lines

90 derivatives exhibited varying degrees of cytotoxicity across seven human tumor cell lines, with specific IC50 values presented in Table 1. As shown in Table 2, all serotonin-related derivatives (compounds Ⅰ-9, Ⅰ-18, Ⅰ-27, Ⅰ-36, Ⅱ-46, Ⅱ-54, Ⅱ-64, Ⅱ-73, Ⅱ-81, and Ⅱ-90) demonstrated excellent cytotoxic activity against seven human tumor cell lines (IC50 = 1.02–11.46 μM). According to Table 2, most compounds showed significantly higher toxicity toward MDA-MB-231 cells compared to the other six types of tumor cells. The cytotoxicity ranking of derivatives was indole > thiophene > imidazole. Substituted amino groups exhibited stronger cytotoxic activity than substituted imino groups. Furthermore, as indicated in Table 3, nitrogen-containing five-membered rings demonstrated greater cytotoxic activity than sulfur-containing five-membered rings.

**TABLE 1:** IC_50_ value of cytotoxicity of derivatives to seven human tumor cell lines (μM).

Compound	PANC-1	HepG2	A549	PC-3M	SKBR-3	MDA-MB-231	MCF-7
Chiisanogenin(Ⅰ)	>200	>200	145.3	134.2	>200	176.9	>200
Ⅰ-1	39.42	30.53	35.19	21.16	24.76	19.78	45.61
Ⅰ-2	32.61	17.61	23.61	16.8	10.25	18.37	5.98
Ⅰ-3	10.47	6.25	8.48	9.2	10.67	8.17	8.46
Ⅰ-4	171.1	>200	75.77	48.01	46.79	52.31	>200
Ⅰ-5	16.11	7.77	11.35	9	8.71	11.83	12.52
Ⅰ-6	25.17	8.51	10.14	11.87	10.49	11.93	14.97
Ⅰ-7	9.96	3.58	5.54	3.86	5.56	1.3	2.68
Ⅰ-8	15.14	6.08	11.43	11.47	8.32	5.64	6.28
Ⅰ-9	2.83	4.06	3.64	2.48	2.68	3.27	2.14
Ⅰ-10	34.75	15.47	12.46	21.34	13.19	17.89	30.41
Ⅰ-11	41.37	27.74	28.83	16.1	15.24	14.15	26.52
Ⅰ-12	15.72	8.25	6.2	7.66	6.59	8.47	8.64
Ⅰ-13	173.3	49.56	46.52	38.46	36.12	40.35	>200
Ⅰ-14	26.46	12.63	15.92	12.24	11.63	17.63	13.47
Ⅰ-15	21.85	7.49	9.95	10.97	9.64	11.47	3.3
Ⅰ-16	7.65	4.08	5.36	3.78	6.39	3.07	3.34
Ⅰ-17	13.58	6.55	12.48	13.6	7.85	6.74	7.46
Ⅰ-18	1.82	5.36	4.71	3.58	2.71	2.67	4.16
Ⅰ-19	21.82	12.36	39.02	11.61	16.79	15.46	26.52
Ⅰ-20	15.04	9.9	12.94	8.38	7.24	15.28	9.31
Ⅰ-21	11.12	7.46	6.18	8.31	9.47	8.47	8.35
Ⅰ-22	94.45	136.1	34.25	24.28	20.13	37.35	135.7
Ⅰ-23	13.6	6.42	8.85	7.22	14.29	9.46	8.17
Ⅰ-24	16.74	6.4	8.83	9.63	8.47	9.36	3.29
Ⅰ-25	5.8	4.36	6.95	6.36	6.85	3.85	3.47
Ⅰ-26	9.47	7.42	13.54	14.62	12.83	10.28	5.34
Ⅰ-27	2.35	6.42	7.92	2.36	3.05	1.02	3.25
Ⅰ-28	17.27	16.7	53.34	15.41	14.76	13.49	24.64
Ⅰ-29	11.44	6.62	11.02	7.72	5.97	10.45	7.83
Ⅰ-30	12.94	7.65	5.64	6.75	7.49	6.28	7.04
Ⅰ-31	>200	>200	81.56	60.85	50.67	65.82	>200
Ⅰ-32	88.78	3.75	6	7.27	5.49	8.51	6.86
Ⅰ-33	10.25	5.48	6.74	9.52	5.56	4.34	4.63
Ⅰ-34	6.55	2.44	3.37	3.8	3.76	1.78	1.98
Ⅰ-35	8.9	9.81	8.47	7.45	6.47	5.71	6.74
Ⅰ-36	5.46	5.17	6.38	4.67	2.3	3.04	4.34
Ⅰ-37	16.44	14.59	35.29	19.52	20.74	13.67	12.11
Ⅰ-38	8.77	6.89	8.12	7.07	10.71	8.24	7.9
Ⅰ-39	29.82	12.23	14.77	11.26	10.54	9.42	25.1
Ⅰ-40	72.22	92.84	38.57	40.76	38.56	29.18	>200
Ⅰ-41	80.48	12.47	15.31	17.89	6.07	8.18	13.56
Ⅰ-42	9.78	10.59	7.52	6.18	7.33	5.47	3.56
43	3.25	3.89	4.28	5.3	4.12	4.32	3.56
Ⅰ-44	6.98	6.09	6.17	5.82	6.92	5.9	3.49
Ⅰ-45	4.13	6.67	7.85	5.47	6.31	3.48	5.42
compound MH(Ⅱ)	>200	>200	>200	103.4	92.33	75.6	>200
Ⅱ-46	37.45	19.78	25.49	28.49	18.79	15.79	23.46
Ⅱ-47	21.96	24.54	15.72	17.3	14.58	13.45	23.48
Ⅱ-48	33.41	21.34	11.71	12.7	11.31	10.38	14.41
Ⅱ-49	63.73	61.69	46.23	32.1	37.25	40.26	111.6
Ⅱ-50	96.67	13.01	18.08	15.99	19.34	14.59	11.74
Ⅱ-51	26.47	15.73	24.82	11.78	10.48	9.7	9.98
Ⅱ-52	7.16	2.76	4.17	3.24	3.35	4.53	2.16
Ⅱ-53	5.42	8.74	8.14	6.34	5.7	4.67	10.47
Ⅱ-54	5.71	5.34	4.38	5.46	4.88	3.65	6.15
Ⅱ-55	46.53	12.08	26.47	34.45	27.63	23.07	18.79
Ⅱ-56	34.04	31.8	10.81	15.77	9.54	10.34	62.41
Ⅱ-57	28.57	19.24	14.56	10.63	9.64	8.47	12.6
Ⅱ-58	64.53	56.72	47.76	87.45	45.53	43.08	123.57
Ⅱ-59	88.49	10.81	17.75	14.49	15.27	16.78	12.9
Ⅱ-60	28.92	23.36	27.44	15.28	14.96	13.49	11.66
Ⅱ-61	5.33	1.46	4.16	2.72	3.47	3.58	2.48
Ⅱ-62	9.67	9.61	9.34	8.14	7.92	6.14	11.36
Ⅱ-63	6.35	8.4	9.82	7.61	3.67	2.34	2.34
Ⅱ-64	29.68	18.48	15.35	16.07	11.75	13.26	14.61
Ⅱ-65	25.91	14.97	14.29	12.31	11.78	10.85	14.59
Ⅱ-66	51.17	32.26	16.46	14.9	12.67	11.64	19.55
Ⅱ-67	57.59	78.49	56.49	90.78	57.46	68.98	145.23
Ⅱ-68	23.94	14.94	9.78	13.63	10.96	19.08	15.18
Ⅱ-69	22.54	14.72	14.49	9.22	8.28	7.89	9.13
Ⅱ-70	4.74	2.41	3.86	2.77	2.73	3.71	2.07
Ⅱ-71	10.47	11.47	12.57	11.96	10.94	8.29	12.74
Ⅱ-72	8.49	5.38	6.28	6.02	2.56	1.58	5.46
Ⅱ-73	7.68	13.14	14.36	10.13	9.46	8.46	4.42
Ⅱ-74	22.66	13.94	10.71	11.42	14.72	11.04	15.55
Ⅱ-75	13.6	8.21	6.07	5.84	7.94	9.63	6.34
Ⅱ-76	79.46	80.45	67.05	76.12	109.34	89.46	106.59
Ⅱ-77	14.19	13.24	7.52	9.73	9.86	8.64	8.13
Ⅱ-78	24.83	15.34	19.5	9.76	8.71	9.64	8.08
Ⅱ-79	6.34	1.27	2.36	3.84	3.45	4.75	1.74
Ⅱ-80	6.34	8.46	9.34	8.42	7.28	6.18	7.28
Ⅱ-81	7.35	7.65	6.18	8.39	3.17	2.57	2.46
Ⅱ-82	14.86	11.14	20.6	9.08	15.26	9.46	10.25
Ⅱ-83	11.76	7.6	6.32	7.41	7.2	10.72	12.14
Ⅱ-84	10.9	7.06	6.04	6.43	7.16	8.46	5.21
Ⅱ-85	67.19	61.35	74.34	64.13	75.63	59.14	95.31
Ⅱ-86	11.85	7.41	6.21	8.31	7.46	6.49	7.33
Ⅱ-87	9.47	4.95	7.36	6.01	6.75	8.41	5.53
Ⅱ-88	7.99	3.55	4.33	3.66	3.78	5.38	1.94
Ⅱ-89	8.79	8.17	7.24	8.86	9.17	8.19	9.06
Ⅱ-90	11.46	9.71	9.34	10.35	8.47	7.28	8.17

**TABLE 2:** Effects of different molecular fragments on MDA-MB-231 cells.

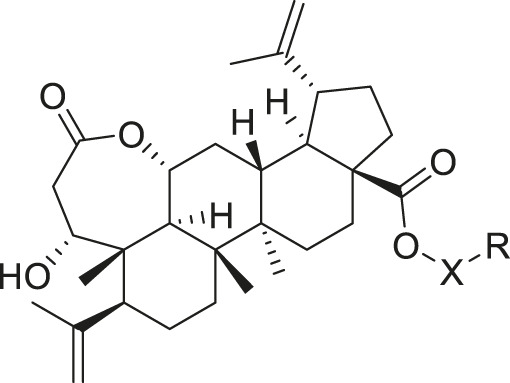 Ⅰ				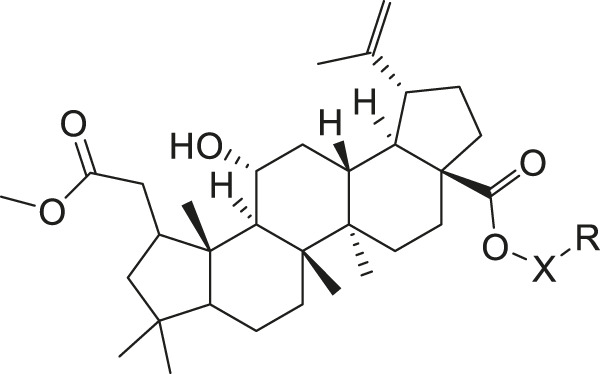 Ⅱ			
Compound	X	R	IC_50_	Compound	X	R	IC_50_
Ⅰ-19	(CH_2_)_5_	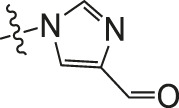	15.46	Ⅱ-64	(CH_2_)_5_	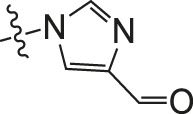	13.26
Ⅰ-20	(CH_2_)_5_	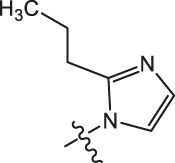	15.28	Ⅱ-65	(CH_2_)_5_	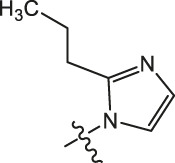	10.85
Ⅰ-21	(CH_2_)_5_	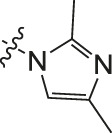	8.47	Ⅱ-66	(CH_2_)_5_	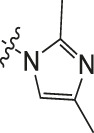	11.64
Ⅰ-22	(CH_2_)_5_	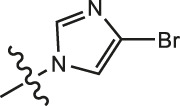	37.35	Ⅱ-67	(CH_2_)_5_	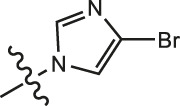	68.98
Ⅰ-23	(CH_2_)_5_	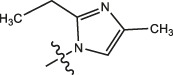	9.46	Ⅱ-68	(CH_2_)_5_	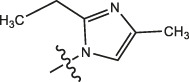	19.08
Ⅰ-24	(CH_2_)_5_	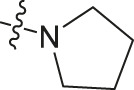	9.36	Ⅱ-69	(CH_2_)_5_	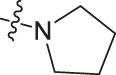	7.89
Ⅰ-25	(CH_2_)_5_	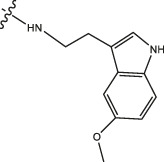	3.85	Ⅱ-70	(CH_2_)_5_	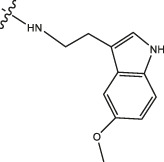	3.71
Ⅰ-26	(CH_2_)_5_	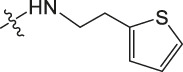	10.28	Ⅱ-71	(CH_2_)_5_	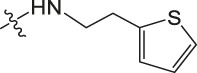	8.29
Ⅰ-27	(CH_2_)_5_	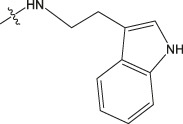	1.02	Ⅱ-72	(CH_2_)_5_	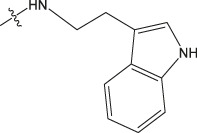	1.58

**TABLE 3:** Cytotoxicity (μM) of 2-propylimidazole compounds on MDA-MB-231 cells.

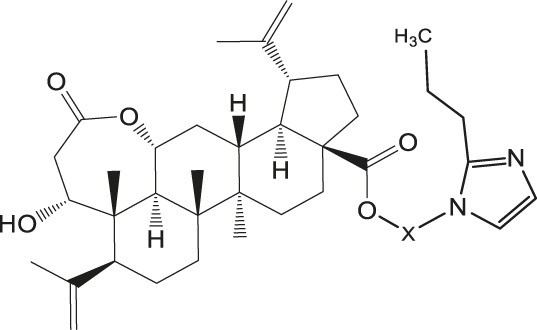 Ⅰ			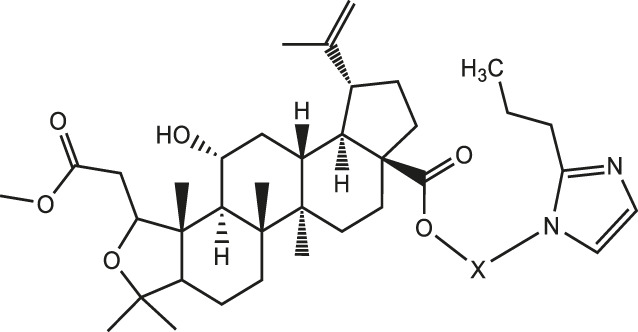 Ⅱ		
Compound	X	IC_50_	Compound	X	IC_50_
Ⅰ-2	(CH_2_)_3_	18.37	Ⅱ-47	(CH_2_)_3_	13.45
Ⅰ-11	(CH_2_)_4_	14.15	Ⅱ-56	(CH_2_)_4_	10.34
Ⅰ-20	(CH_2_)_5_	15.28	Ⅱ-65	(CH_2_)_5_	10.85
Ⅰ-29	(CH_2_)_6_	10.45	Ⅱ-74	(CH_2_)_6_	11.04
Ⅰ-38	(CH_2_)_10_	8.24	Ⅱ-83	(CH_2_)_10_	10.72

As illustrated in Table 3, taking 2-propylimidazole compounds as an example, we observed that when chiisanogenin served as the lead compound, the cytotoxic activity increased with the carbon number of the intermediate dibromoalkane. In contrast, when compound MH acted as the lead compound, no significant change in activity was noted.

Interestingly, as shown in Table 4, during the cytotoxicity analysis of indoleamine compounds on MDA-MB-231 cells, the highest activity was observed when the dibromoalkane contained five carbons, potentially due to the connection of the imine group. Notably, compounds linked to tryptophan exhibited the strongest activity. Therefore, we conclude that the incorporation of indole compounds significantly enhances the cytotoxicity of the lead compound. Among these derivatives, compound Ⅰ-27 demonstrated the highest cytotoxicity against seven human tumor cell lines, particularly against MDA-MB-231 cells, with an IC_50_ value of 1.02 μM. Consequently, we selected the MDA-MB-231 cell line as a model to investigate the anticancer activity and mechanism of action of compound Ⅰ-27.

**TABLE 4:** Cytotoxicity (μM) of tryptamine compounds on MDA-MB-231 cells.

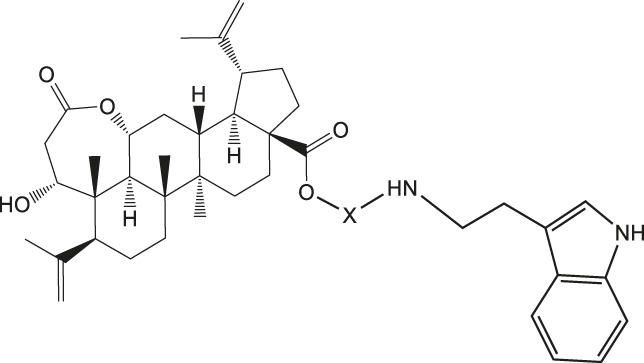 Ⅰ			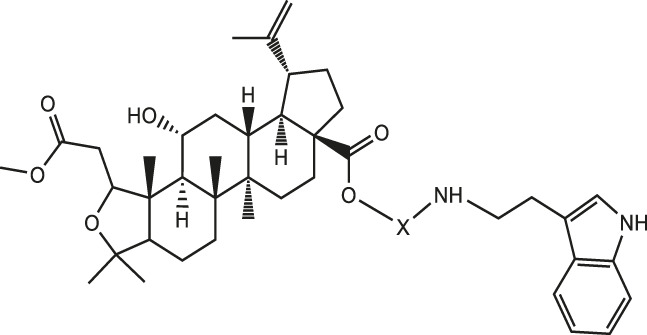 Ⅱ		
Compound	X	IC_50_	Compound	X	IC_50_
Ⅰ-9	(CH_2_)_3_	3.27	Ⅱ-54	(CH_2_)_3_	3.65
Ⅰ-18	(CH_2_)_4_	2.67	Ⅱ-63	(CH_2_)_4_	2.34
Ⅰ-27	(CH_2_)_5_	1.02	Ⅱ-72	(CH_2_)_5_	1.58
Ⅰ-36	(CH_2_)_6_	3.04	Ⅱ-81	(CH_2_)_6_	2.57
Ⅰ-45	(CH_2_)_10_	3.48	Ⅱ-90	(CH_2_)_10_	7.28

### 3.3 Compound Ⅰ-27 inhibits the proliferation of TNBC cells

**FIGURE 1:**
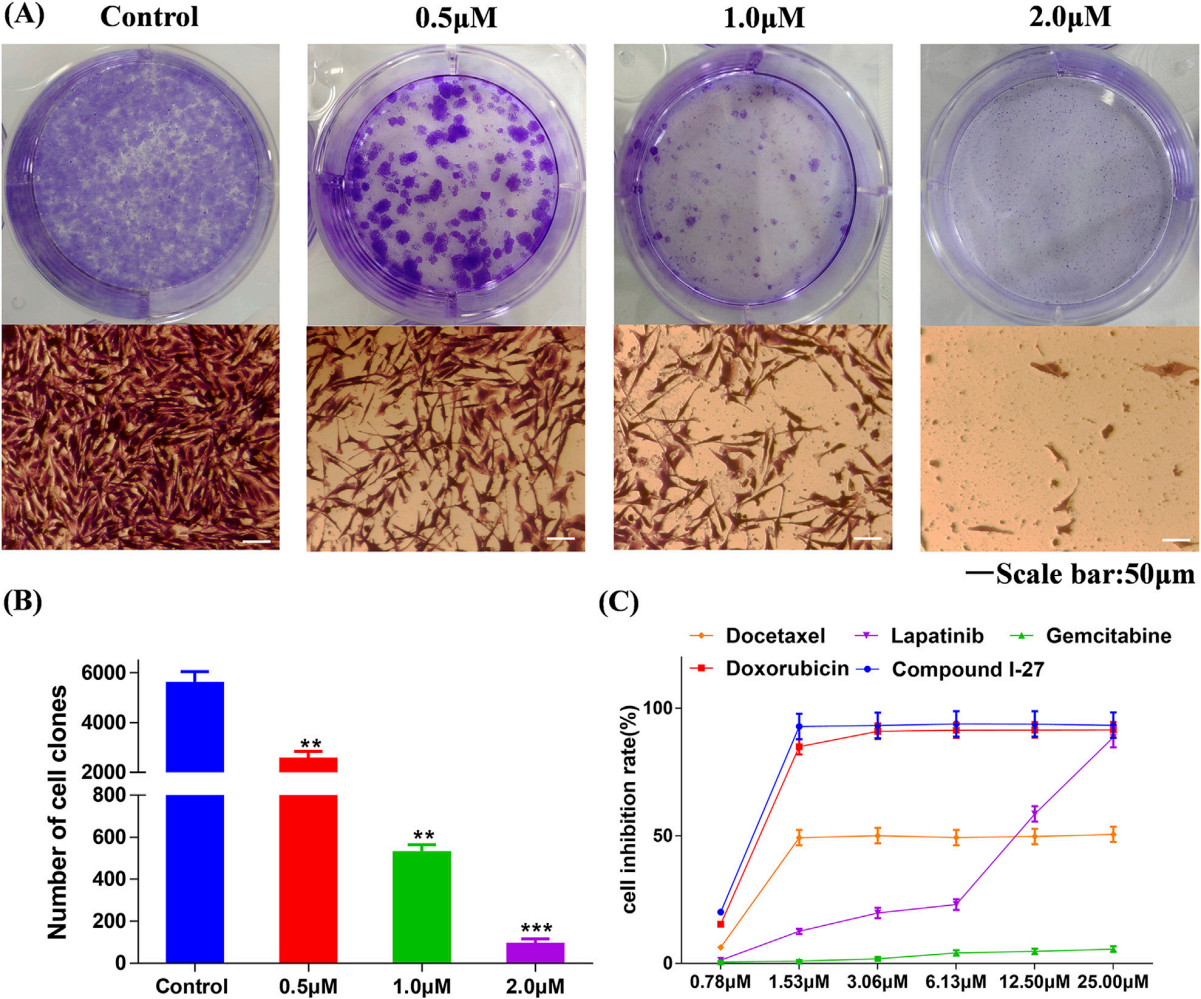
Compound Ⅰ-27 inhibits the proliferation of cells. **(A,B)** Effect of compound Ⅰ-27 on clone formation in cells. Data are presented as the mean ± SD (n = 3). Compared to the control group, **p < 0.01 ***p < 0.05. **(C)** MDA-MB-231 growth curves for 48 h of different drug treatments. Data are presented as the mean ± SD (n = 6).

One of the main characteristics of anticancer drugs is cytotoxicity (Pfeffer and Singh, 2018). Through the colony formation assay, we further investigated the comprehensive effects of compound Ⅰ-27 on MDA-MB-231 cells. The results are presented in Figure 1A. Compared with the blank control group, in the treatment group, as the concentration of compound Ⅰ-27 increased, the number of colonies formed by MDA-MB-231 cells was significantly reduced, indicating that compound Ⅰ-27 effectively inhibits cell colony formation ability (Figure 1B, *p* < 0.005). Additionally, we compared compound Ⅰ-27 with four commonly used clinical drugs for treating TNBC (doxorubicin, docetaxel, lapatinib, and gemcitabine). The results demonstrated that at a concentration of 1.53 μM, compound Ⅰ-27 exhibited significantly higher cytotoxicity than other clinical drugs, achieving a cell inhibition rate approaching 95%. Moreover, the cytotoxic effect of compound Ⅰ-27 on MDA-MB-231 cells increased in a concentration-dependent manner (Figure 1, *p* < 0.01).

### 3.4 Compound Ⅰ-27 inhibits the migration and invasion of TNBC cells

**FIGURE 2:**
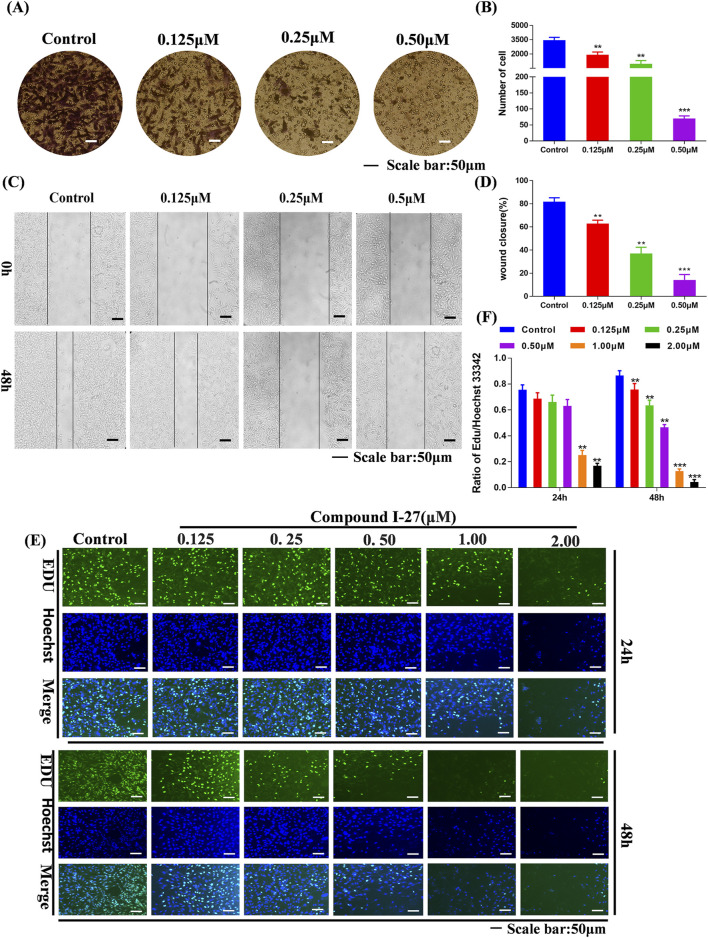
Effect of compound Ⅰ-27 on the proliferation of cells. **(A)** Transwell experiments **(B)** Cell scratch assay **(C)** Quantify the number of cell invasions. **(D)** Quantify wound healing rate. **(E)** EDU detection of cell proliferation (Scale bar = 50). **(F)** Quantitative results of cell proliferation rate.

Transfer is the main reason for the low quality of life and survival rate of TNBC patients (Chen et al., 2024). The wound healing assay and transwell assay were employed to investigate the effects of compound Ⅰ-27 on cell migration and invasion. Prior to these experiments, it was necessary to optimize the concentration and duration of compound Ⅰ-27’s action. A concentration that exhibited minimal cytotoxicity to MDA-MB-231 cells was selected to minimize the influence of cell death on the experimental results. The results demonstrated that, compared with the blank control group, the fluorescence intensity of cells in the treatment group decreased as the concentration and exposure time increased, indicating a significant increase in the inhibitory effect of compound Ⅰ-27 on cell proliferation (Figure 2E). At 24 h, no significant change in cell proliferation was observed when the concentration of compound Ⅰ-27 was increased to 0.50 μM. However, at 48 h, all tested concentrations significantly inhibited cell proliferation (Figure 2F, *p* < 0.005). Therefore, concentrations of 0.125, 0.25, and 0.50 μM were chosen for a 24-h incubation period to further evaluate the effects of compound Ⅰ-27 on cell migration and invasion. In the blank condition, MDA-MB-231 cells penetrated the transwell chamber coated with matrix gel, demonstrating their invasive capability. To investigate the effect of compound Ⅰ-27 on the invasion ability of MDA-MB-231 cells, Transwell experiments were conducted (Figure 2A). The results indicated that, compared with the blank control group, the invasion ability of cells was significantly reduced as the concentration of compound Ⅰ-27 increased. At a concentration of 0.50 μM, the cell invasion rate was the lowest (Figure 2B, *p* < 0.005). Additionally, the impact of compound Ⅰ-27 on the migration ability of MDA-MB-231 cells was evaluated using a wound healing assay (Figure 2C). In the blank group, MDA-MB-231 cells migrated and nearly completely covered the scratched area. Compared with the blank group, the wound healing rate decreased significantly with increasing concentrations of compound Ⅰ-27, with the lowest wound healing rate observed at 0.5 μM (Figure 2D, *p* < 0.005). In summary, compound Ⅰ-27 effectively inhibits both cell invasion and wound healing.

### 3.5 Compound Ⅰ-27 inhibits angiogenesis by promoting the generation of TSP1 through inhibiting ID1

**FIGURE 3:**
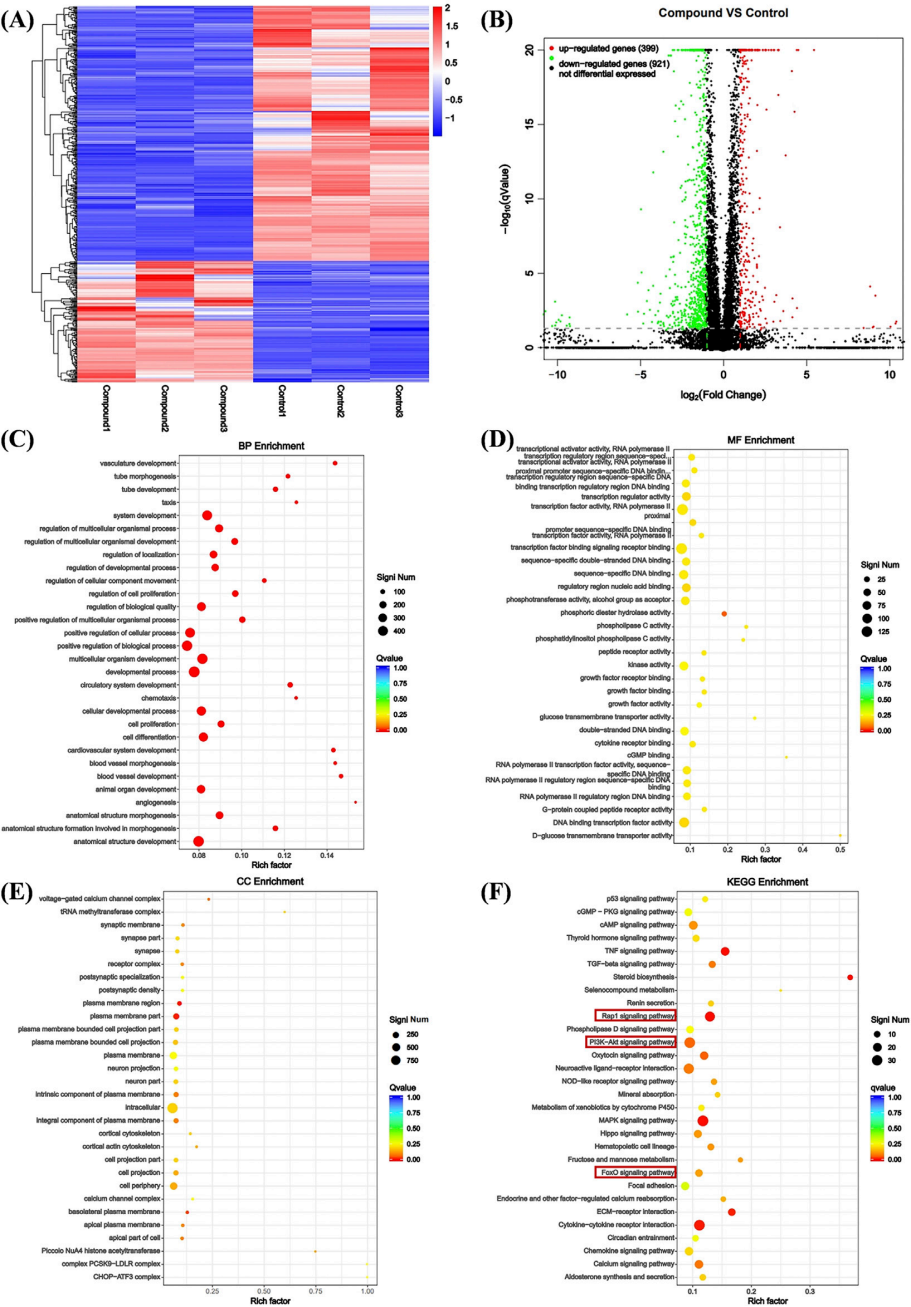
Analysis of DEGs. **(A)** Heat map analysis of DEGs. X-axis coordinate: sample name, Y-axis coordinate: differential gene name, color: gene expression level. **(B)** Volcano analysis of DEGs. X-axis: Value of gene differential changes, Y-axis: Significance of gene expression changes, color: Number of upregulated and downregulated genes. **(C)** Enrichment was performed for each GO term under BP. **(D)** Enrichment was performed for each GO term under CC. **(E)** Enrichment was performed for each GO term under MF. **(F)** Bubble plot of KEGG pathway enrichment for differentially expressed genes.

Using transcriptomics analysis to analyze gene regulatory changes before and after drug administration. Differentially expressed genes (DEGs) include upregulated and downregulated genes, which are determined by the difference in relative expression levels between the control group and the treatment group. DEGs detection is used to show DEG aggregation, indicating good data reproducibility before and after sample treatment (Figure 3A) The volcano plot can visually compare the differences in gene expression levels between the blank group and the treated group. The results showed that after comparing the two groups, we found a total of 1320 DEGs, including 399 genes upregulated and 921 genes downregulated (Figure 3A).

We performed Gene Ontology (GO) enrichment analysis on the differentially expressed genes (DEGs) of the control group and compound Ⅰ-27 group, selecting the top 20 categories with significant differences and strong functional relevance for biological process (BP), cellular component (CC), and molecular function (MF) analysis. In BP, the DEGs were primarily associated with angiogenesis, cell proliferation, cell differentiation, and vascular morphological growth processes (Figure 3C). In CC, the DEGs were mainly distributed in intracellular regions, plasma membrane regions, and cortical cytoskeleton (Figure 3D). In MF, the DEGs predominantly participated in sequence-specific DNA binding in transcription regulatory regions and phosphotransferase activity (Figure 3E). Additionally, KEGG pathway enrichment analysis revealed the top 30 most significantly enriched signaling pathways, including the Rap1 signaling pathway and the PI3K/Akt/FoxO1 signaling pathway (Figure 3F). Combining GO enrichment analysis with KEGG results, we propose that the Rap1 signaling pathway, which is related to angiogenesis, and the PI3K/Akt/FoxO1 signaling pathway, which is associated with cell apoptosis, may play critical roles in the anti-TNBC effects of compound Ⅰ-27.

### 3.6 Compound Ⅰ-27 inhibits tumor angiogenesis through the ID1/TSP-1 pathway

**FIGURE 4:**
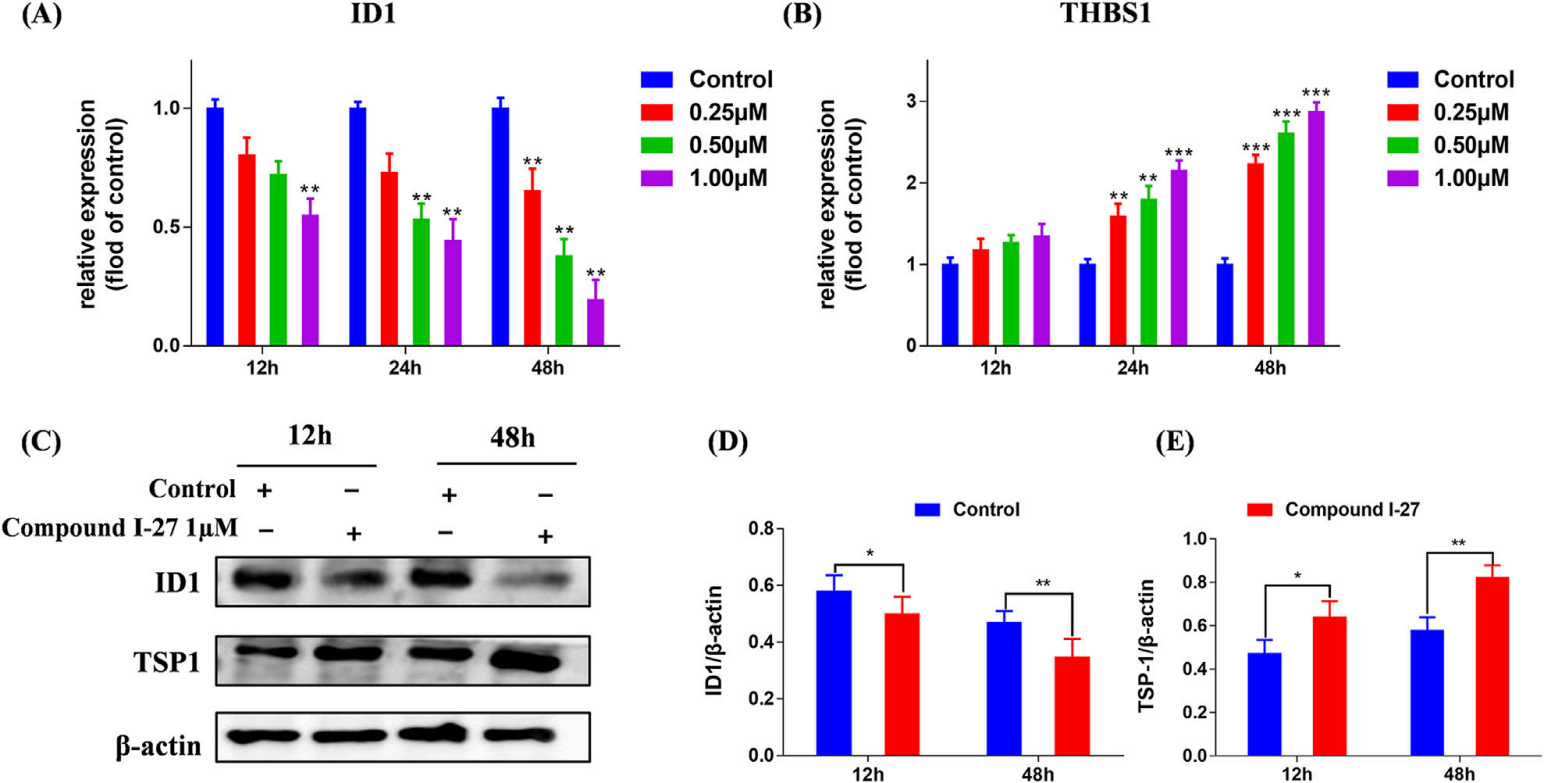
Validation of the gene expression levels of ID1 and TSP-1 through RT-qPCR and Western blotting experiments. **(A,B)** Gene expression changes of ID1 and THBS1 at different times and concentrations were detected by RT-qPCR. **(C)** Different time points - ID1 and THBS1 protein expression levels. **(D)** ID1/β-actin ratio **(E)** TSP-1/β-actin ratio. Data are presented as the mean ± SD (n = 3). Compared to the control group, *p < 0.05, **p < 0.01, ***p < 0.005.

We selected proteins ID1 and TSP-1, which are associated with the Rap1 signaling pathway, for validation. Using RT-qPCR detection, we examined the mRNA expression levels of ID1 and THBS1 in cells following treatment with compound Ⅰ-27. The results demonstrated that after treating cells with compound Ⅰ-27 (1 μM), the mRNA expression of ID1 significantly decreased as the treatment time increased. After 48 h of treatment with compound Ⅰ-27, the mRNA expression of ID1 significantly decreased with increasing concentration (Figure 4A, *p* < 0.005). In contrast, the mRNA expression level of THBS1 exhibited an opposite trend to that of ID1 (Figure 4B, *p* < 0.005). Protein expression levels of ID1 and TSP-1 were further analyzed by Western blotting (Figure 4C). The results indicated that as the treatment time with compound Ⅰ-27 (1 μM) increased, the protein expression level of ID1/β-actin significantly decreased (Figure 4D, *p* < 0.01), while the protein expression level of TSP-1/β-actin significantly increased (Figure 4E, *p* < 0.01). These findings were consistent with the mRNA and protein expression trends observed in the DEGs analysis. Therefore, we conclude that compound Ⅰ-27 may inhibit TNBC tumor angiogenesis through the ID1/TSP-1 signaling pathway.

### 3.7 Compound Ⅰ-27 promotes apoptosis through the PI3K/AKT/FoxO1 signaling pathway

**FIGURE 5:**
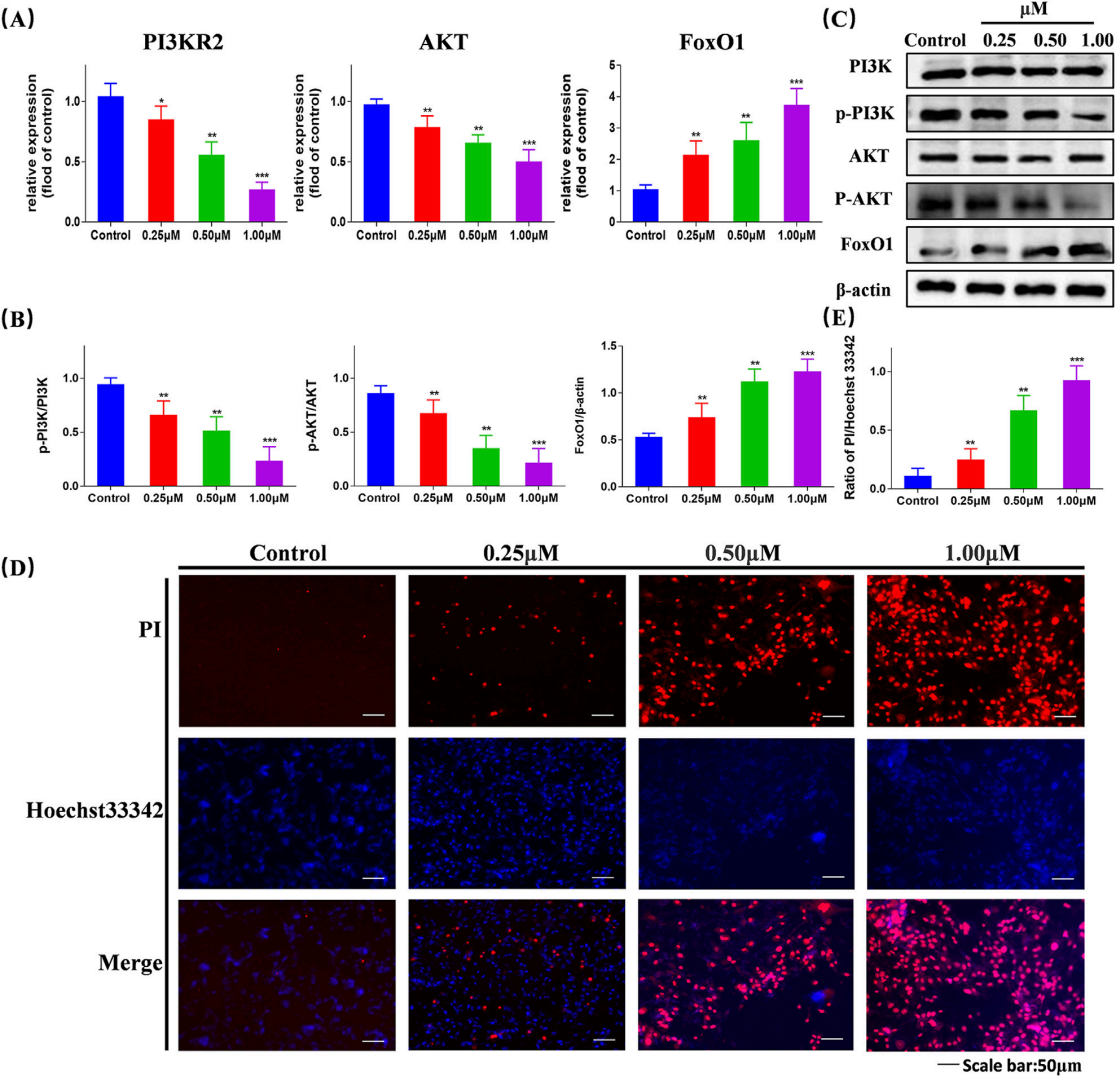
Validation of the gene expression levels of PI3K, AKT, and FoxO1 through RT-qPCR and Western blotting experiments, validate that compound Ⅰ-27 induces cell apoptosis **(A)** Changes in gene expression of different concentrations of P13KR2, AKT, FoxO1 detected by RT-qPCR. **(B,C)** Expression levels of PI3K/p-PI3K, AKT/p-AKT and FoxO1/β-actin proteins. **(D,E)** Hoechst 33342/PI staining and quantitative graph Data are presented as the mean ± SD (n = 3). Compared to the control group, *p < 0.05, **p < 0.01, ***p < 0.005.

We selected proteins PI3K, AKT, and FoxO1, which are associated with the PI3K/AKT/FoxO1 signaling pathway, for validation. Using RT-qPCR detection, we examined the mRNA expression levels of PI3KR2, AKT, and FoxO1 in cells following treatment (Figure 5A). Compared with the blank control group, the mRNA expression levels of PI3K and AKT in the compound Ⅰ-27 group decreased with increasing concentration (p < 0.005), while the mRNA expression level of FoxO1 increased with increasing concentration (p < 0.005). By Western blotting analysis, we further evaluated the protein expression levels of PI3K, AKT, and FoxO1 (Figure 5C). As the concentration of compound Ⅰ-27 increased, the protein expression levels of p-PI3K/PI3K and p-AKT/AKT significantly decreased, whereas the protein expression level of FoxO1/β-actin increased (Figure 5B, p < 0.005). These findings were consistent with the mRNA and protein expression trends observed in the DEGs analysis.

We assessed cell apoptosis using Hoechst 33342/PI staining (Figure 5D). Compared with the control group, in the compound Ⅰ-27 treatment group, as the concentration increased, the fluorescence intensity was significantly enhanced, indicating a higher cell mortality rate (Figure 5E, p < 0.005). Therefore, we propose that compound Ⅰ-27 may induce apoptosis in TNBC tumor cells via the PI3K/AKT/FoxO1 signaling pathway.

### 3.8 Simulation effect of compound Ⅰ-27 binding to PI3K proteins

**FIGURE 6:**
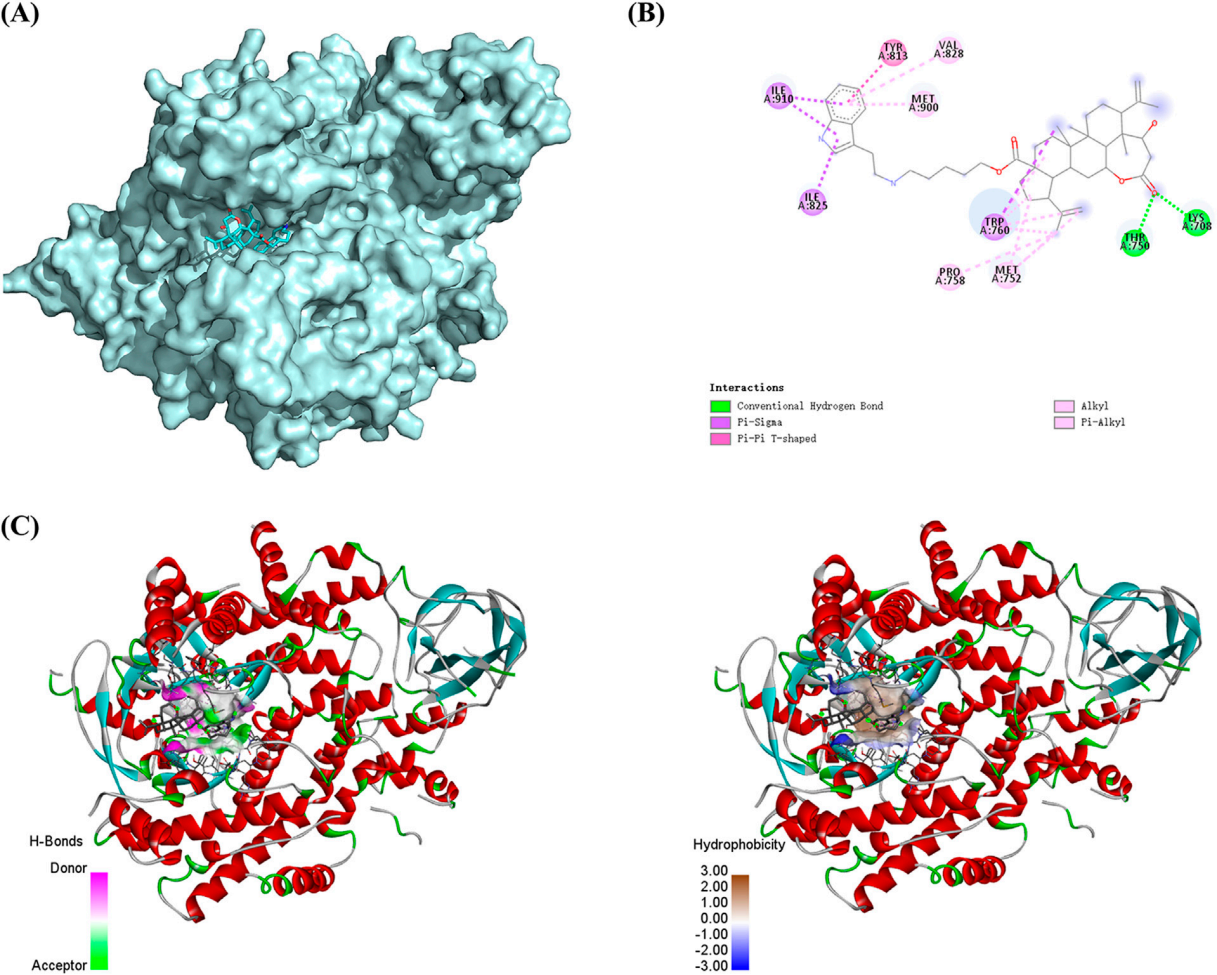
Molecular docking. **(A)** Spatial mosaic conformation of the domain of compound Ⅰ-27 with PI3K protein. **(B)** Planar binding conformation (interaction with protein amino acid residues) of compound Ⅰ-27 with PI3K protein. **(C)** Hydrogen bonding and hydrophobic interactions between compound Ⅰ-27 and the structural domain of PI3K protein.

We selected the PI3K protein domain with the highest affinity (−9.8 kcal/mol). The conformation of compound Ⅰ-27 can be visualized, and it is tightly embedded within the active pocket of PI3K (Figure 6A). Compound Ⅰ-27 appears to form hydrogen bonds with amino acid residues LYSA: 708 and THRA: 750 on the A chain of the PI3K protein (Figure 6B). From a spatial conformation perspective, under the influence of p-π, π-π, and other forces, the hydrogen bonding interactions between compound Ⅰ-27 and PI3K are primarily concentrated at C-1 of chiisanogenin, while hydrophobic interactions are clearly distributed in the aromatic ring of serotonin (Figure 6C). Therefore, compound Ⅰ-27 can effectively bind to the active pocket of PI3K.

### 3.9 Compound Ⅰ-27 inhibits the growth of TNBC *in vivo*

**FIGURE 7:**
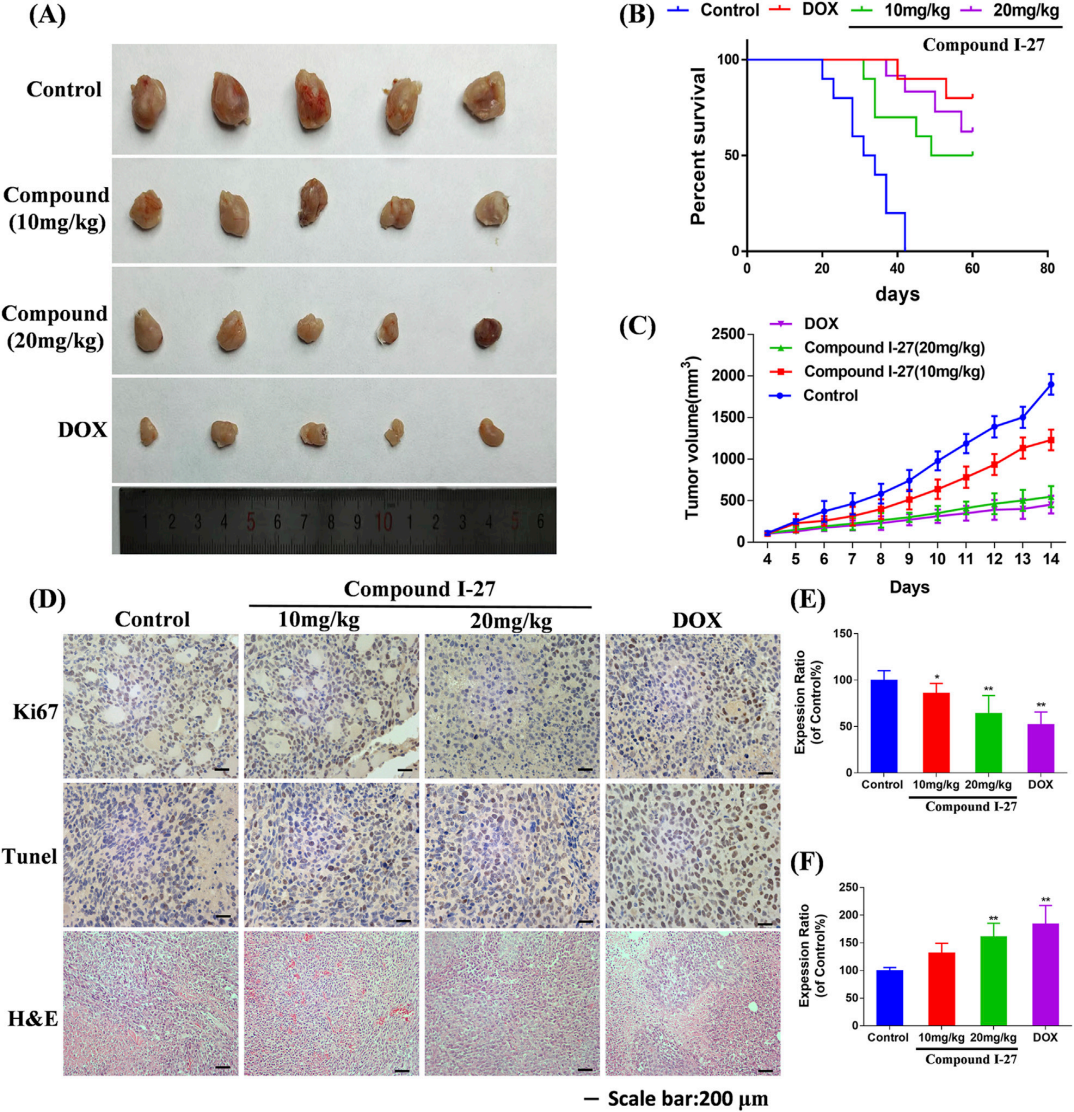
Inhibitory effect of compound Ⅰ-27 on tumor growth. **(A)** Tumour size **(B)** Survival curves. **(C)** Tumour volume. **(D)** Immunohistochemical staining of Ki67 protein, TUNEL staining, H&E staining of tumor tissue. **(E)** The positive signal intensity of Ki67. **(F)** Detection of apoptosis by TUNEL staining. The numerical values are expressed as mean ± standard deviation (n = 5), and compared with the control group, *p < 0.05, **p < 0.01.

To investigate the effect of compound Ⅰ-27 on TNBC in mice, we established a 4T1 xenograft tumor model (Figure 7A). Compared with the control group, the tumor volume in the compound Ⅰ-27 group significantly decreased and exhibited a dose-dependent trend. Compared with DOX, there was no significant difference in tumor volume between the compound Ⅰ-27 (20 mg/kg) group and the DOX group (Figure 7C, *p* < 0.01). In the survival analysis experiment (Figure 7B), compared with the model group, the median survival time of the treatment group significantly increased (*p* < 0.01), while there was no significant difference in median survival time between the compound Ⅰ-27 (20 mg/kg) group and the DOX group.

The expression level of the proliferation marker Ki67 protein in tumor tissue was detected by immunohistochemistry (Figure 7D). Compared with the control group, the number of Ki67-positive cells in the compound Ⅰ-27 group significantly decreased in a dose-dependent manner. Compared with the DOX group, there was no significant change in the number of Ki67-positive cells in the compound Ⅰ-27 (20 mg/kg) group (Figure 7E, *p* < 0.01). Cell apoptosis in tumor tissue was assessed through TUNEL staining. Compared with the control group, the number of TUNEL-positive cells in the compound Ⅰ-27 group increased in a dose-dependent manner. Compared with the DOX group, there was no significant difference in the number of TUNEL-positive cells in the compound Ⅰ-27 (20 mg/kg) group (Figure 7F, *p* < 0.01). H&E staining results showed that, compared with the blank group, the gaps in tumor tissue sections of each dose group of compound Ⅰ-27 decreased with increasing concentration. Therefore, we conclude that compound Ⅰ-27 can inhibit the growth of TNBC tumors in mice and improve their survival time.

### 3.10 Compound Ⅰ-27 inhibits angiogenesis and suppresses TNBC metastasis

**FIGURE 8:**
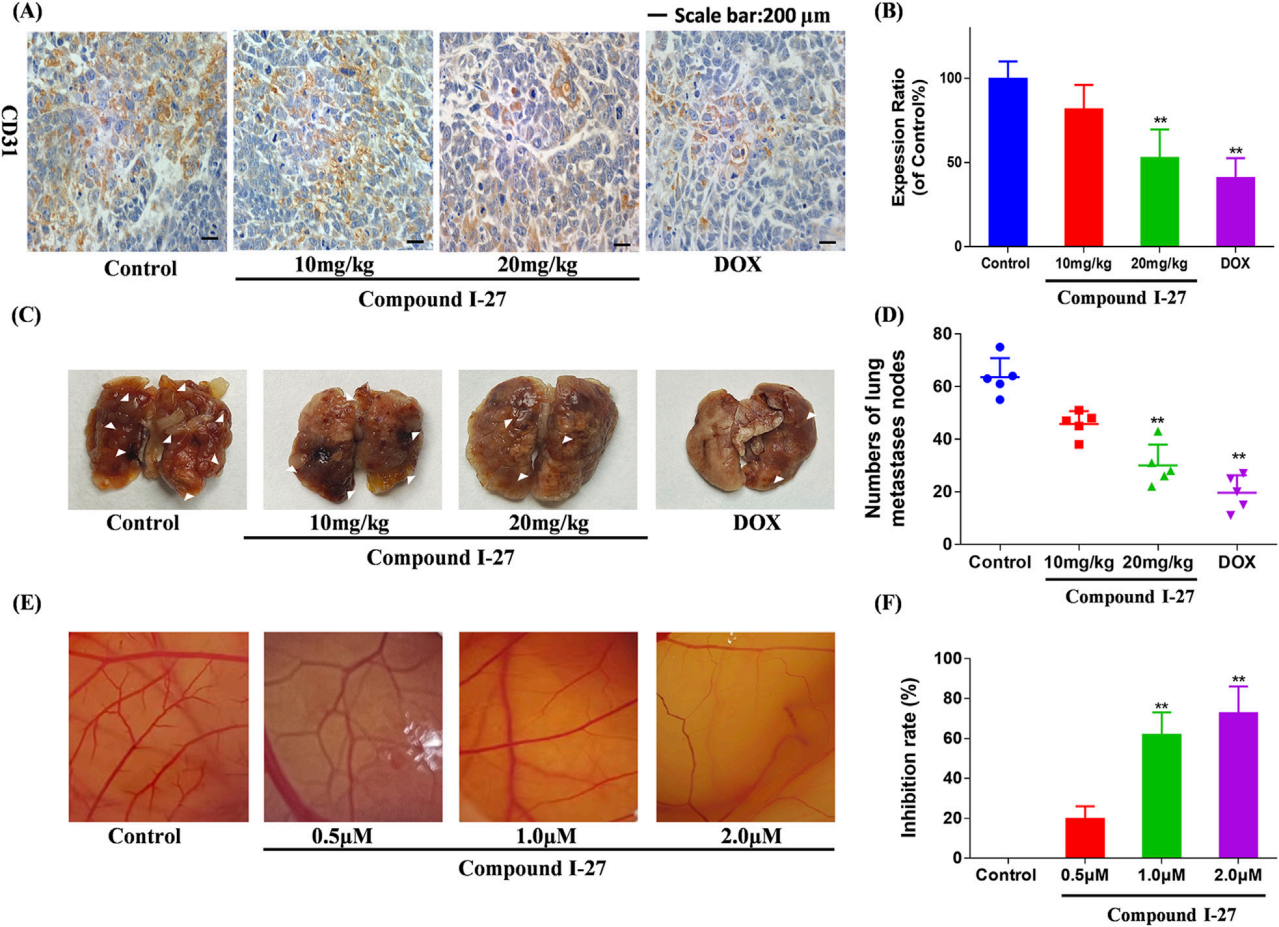
Compound Ⅰ-27 can inhibit angiogenesis. **(A)** Number of pulmonary nodules. **(B)** Immunohistochemical expression of CD31, a biomarker of angiogenesis. **(C)** Perform the CAM experiment to verify the effect of compound Ⅰ-27 on angiogenesis. **(D)** Number of pulmonary nodules. **(E)** inhibition rate of angiogenesis. **(F)** The positive signal intensity of CD31.The numerical values are expressed as mean ± standard deviation (n = 5), and compared with the control group, *p < 0.05, **p < 0.01.

To investigate the anti-tumor metastasis ability of compound Ⅰ-27 in mice, we established a mouse TNBC lung metastasis model (Figure 8C). Compared with the blank group, the number of pulmonary nodules in the compound Ⅰ-27 group significantly decreased in a dose-dependent manner. Compared with the DOX group, there was no significant difference in the number of pulmonary nodules in the compound Ⅰ-27 (20 mg/kg) group (Figure 8D, p < 0.01). These experimental results indicate that compound Ⅰ-27 can inhibit TNBC lung metastasis. Angiogenesis in tumor tissue is a key factor for tumor cell metastasis. Therefore, we performed immunohistochemical detection of the angiogenesis marker CD31 in tumor tissues from each group (Figure 8A). Compared with the control group, the expression of CD31 in the compound Ⅰ-27 group was significantly reduced in a dose-dependent manner. Compared with the DOX group, the expression of CD31 in the compound Ⅰ-27 (20 mg/kg) group showed no significant difference (Figure 8B, *p* < 0.01). The CAM (chick chorioallantoic membrane) can rapidly develop from an avascular membrane morphology into a membrane with a dense vascular network (Kundeková et al., 2021) CAM is an important model for studying the biological behavior of tumors. Due to its easy observation, simple operation, high repeatability, short experimental period, and ease of large sample replication, it is currently a classic method for studying angiogenesis and widely used in the preliminary screening of anti-tumor drugs (Harper et al., 2021). Therefore, the CAM experiment was performed using compound Ⅰ-27 to verify the effect of compound Ⅰ-27 on neovascularization (Figure 8E), compared with the control group, in the compound Ⅰ-27 administration group, as the administration concentration increased, the ability of compound Ⅰ-27 to inhibit angiogenesis was significantly increased (Figure 8F, *p* < 0.01)). In summary, compound Ⅰ-27 can inhibit tumor cell metastasis by inhibiting tumor tissue angiogenesis.

### 3.11 ID1 is a key target for inhibiting tumor angiogenesis

**FIGURE 9:**
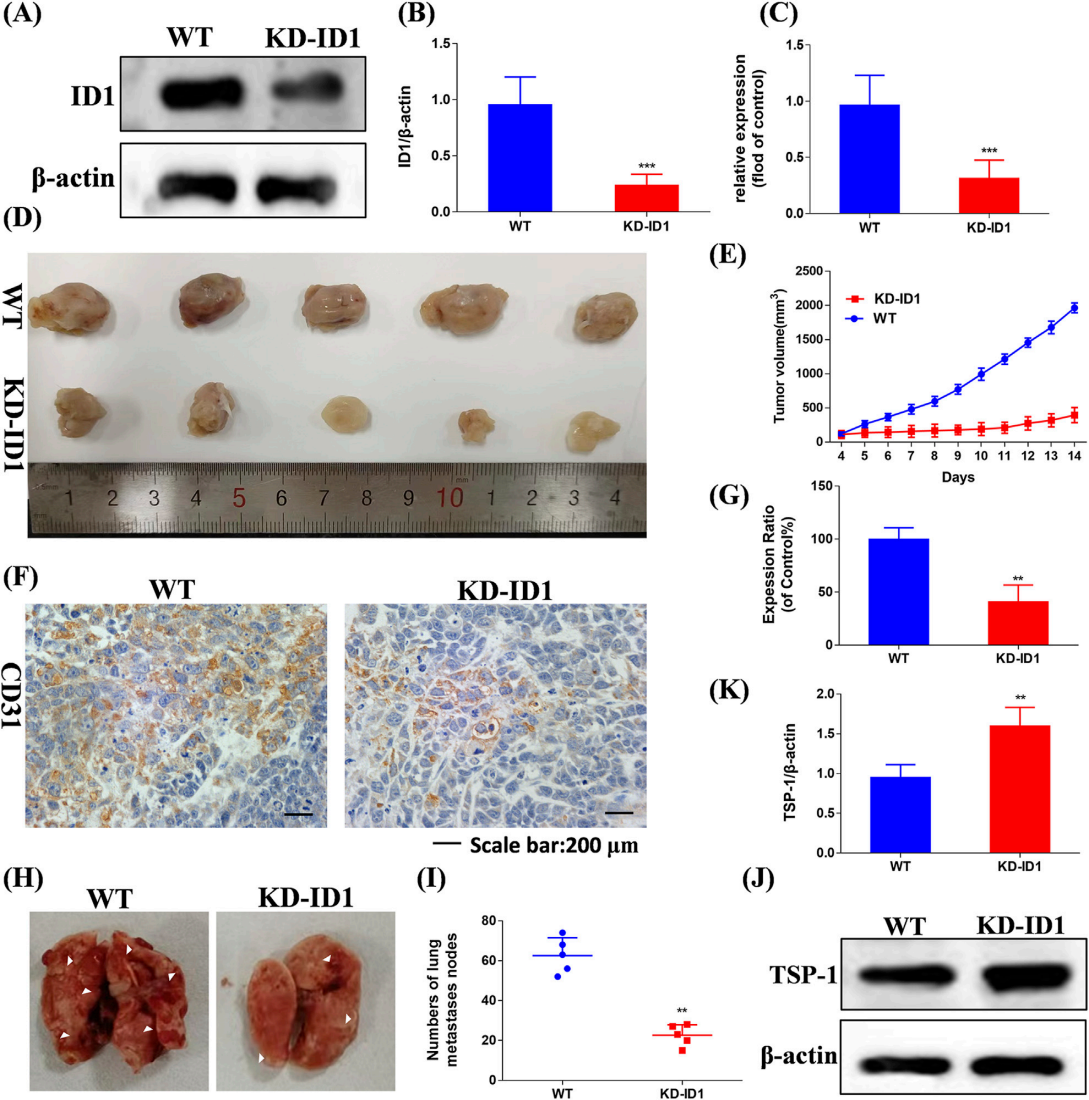
Compound Ⅰ-27 inhibits the key target of TNBC angiogenesis and cell apoptosis - ID1 **(A,B)** Detection of protein expression level after ID1 gene knockout. **(C)** Detection of gene expression level after ID1 gene knockout. **(D)** Tumour size. **(E)** Tumour volume. **(F)** Immunohistochemical expression of CD31, a biomarker of angiogenesis. **(G)** The positive signal intensity of CD31. **(H,I)** Number of pulmonary nodules. **(J,K)** Detection of TSP-1 protein expression level after ID1 gene knockout. The numerical values are expressed as mean ± standard deviation (n = 5), and compared with the control group, *p < 0.05, **p < 0.01, ***p < 0.005.

We knocked down the expression of ID1 in 4T1 cells using KD-ID1 plasmid, and the knockdown effect was verified by Western blotting and RT-qPCR (Figures 9A–C, p < 0.005). In the 4T1 xenograft tumor model experiment, compared with the WT group, the KD-ID1 group exhibited a significant reduction in tumor volume, and simultaneously, the number of positive cells for the angiogenesis marker CD31 was significantly decreased (Figures 9D–G, *p* < 0.01). In the mouse TNBC lung metastasis experiment, compared with the WT group, the KD-ID1 group showed a marked decrease in the number of lung nodules (Figures 9H,I, *p* < 0.01). Therefore, KD-ID1 can inhibit angiogenesis in TNBC tumor tissue, thereby suppressing the growth and metastasis of TNBC.

To further verify that ID1 inhibits tumor angiogenesis and metastasis by upregulating TSP1, we used Western blotting to evaluate the expression of TSP-1 (Figure 9J). The results showed that after knocking down ID1, the expression of TSP-1 was significantly increased (Figure 9K, *p* < 0.01). In conclusion, ID1 is a critical target for inhibiting tumor angiogenesis in TNBC. By upregulating TSP-1 to suppress angiogenesis, it effectively inhibits the growth and metastasis of TNBC.

## 4 Discussion and conclusion

TNBC is the most destructive malignant tumor because it has a high degree of invasiveness and a lack of targetable molecules (Li et al., 2020). This limits both the number of available targets for TNBC and the development of effective therapies. Therefore, among all breast cancer subtypes, TNBC has the highest recurrence rate and mortality rate (Singh and Yadav, 2021). In conclusion, identifying new targets and developing new therapeutic candidates for TNBC are urgent priorities. Natural products, as important sources of small molecule drugs, lead compounds, and small molecule probes, play an irreplaceable role in elucidating biological mechanisms and treating related diseases According to relevant statistics, natural products and their derivatives account for about half of the small molecule drugs approved for market by the US Food and Drug Administration (FDA). (Carlson, 2010). Natural products are an important source of anti-tumor drugs, and research on the anti-tumor mechanism of 3,4-*seco*-lupane type triterpenoids is becoming increasingly in-depth. There are also more and more studies on derivatization of their structures to enhance activity and improve drug properties. (Grishko and Galaiko, 2016).

The results of our previous research demonstrated that chiisanogenin exhibits significantly greater anticancer activity compared to chiisanoside. Following a substitution reaction with dibromoalkane, the resulting compound displays higher biological activity than chiisanogenin. Therefore, in this study, chiisanogenin was selected as the lead compound, and dibromoalkane was used as an intermediate bridge to connect chiisanogenin with azo, imino, or indole groups to further enhance its biological activity. Through MTT assays conducted on seven human tumor cell lines, we identified that indole derivatives exhibit high cytotoxicity against MDA-MB-231 cells. In the cytotoxicity analysis of indole tryptamine compounds targeting MDA-MB-231, compound Ⅰ-27 was found to possess the strongest cytotoxicity. Consequently, compound Ⅰ-27 was selected for mechanistic studies on TNBC. Using the MTT assay, compound Ⅰ-27 exhibited significantly superior cytotoxic activity compared to four clinically effective TNBC drugs. We quantified the cell mortality rate using Hoechst 33342/PI staining. As the concentration of compound Ⅰ-27 increased, the cell mortality rate significantly increased. The incidence of distal organ metastasis in TNBC patients is notably higher than in other breast cancer subtypes. Approximately 20%–30% of breast cancer patients develop lung, brain, bone, or liver metastases after diagnosis and treatment, with lung metastasis being particularly critical due to its association with 60%–70% of patient deaths (Foulkes et al., 2010). Tumor metastasis is a series of multi-step and complex processes, including changes in tumor cells, related regulatory factors, and changes in the tumor microenvironment (Leung et al., 2015). Determination of the activity of compound Ⅰ-27 on colony formation, migration, and invasion of MDA-MB-231 cells. Through cell scratch experiments, Transwell experiments and cloning experiments, a significant reduction in migration and colony formation was observed.

In transcriptomics, GO and KEGG enrichment analyses are employed to study drug-disease cross-target proteins, thereby predicting genes and their corresponding proteins in related pathways. By utilizing the KEGG database to organize relevant pathways, a comprehensive investigation of the biological processes (BP) associated with the Rap1 signaling pathway and the PI3K/AKT/FoxO1 signaling pathway is conducted. Simultaneously, GO analysis is performed to more accurately describe the characteristics of gene products. Transcriptomics analysis revealed significant differences between the two sample groups. A total of 399 upregulated genes and 921 downregulated genes were identified as differentially expressed genes (DEGs). This transcriptomics analysis provides valuable insights into the mechanisms underlying the anti-TNBC tumorigenesis and metastasis effects of compound Ⅰ-27. Furthermore, RT-qPCR validation confirmed the results of the transcriptomics analysis. TSP1 has been shown to inhibit angiogenesis and endothelial cell proliferation (Liu et al., 2015). Through transcriptomics analysis, we found that compound Ⅰ-27 inhibits tumor angiogenesis by inhibiting ID1 and activating TSP-1. We used the CAM experiment to verify whether compound Ⅰ-27 has the effect of inhibiting angiogenesis. The experimental results show that as the administration concentration increases, the number of blood vessels gradually decreases. Abnormal activation of the PI3K/AKT signaling pathway is very common in breast cancer and is related to cell transformation, tumorigenesis, cancer development and progression (Khatpe et al., 2021). The PI3K/AKT/FoxO1 signaling pathway is one of the important pathways causing malignant processes and drug resistance in TNBC patients (Qin et al., 2022). We studied the downstream protein FoxO1 of the PI3K/AKT pathway and further explored the mechanism of action of compound Ⅰ-27 on apoptosis of TNBC. Transcriptomics analysis results showed that compound Ⅰ-27 phosphorylates PI3K and AKT and activates FoxO1.

This article also investigates the molecular mechanism of compound Ⅰ-27 in mouse xenograft 4T1 cell tumor tissue. We established xenograft tumors in mice, and the results of IHC analysis on tumor tissues showed that the number of positive cells for the angiogenesis marker CD31 and the proliferation marker Ki67 decreased with increasing concentration of compound Ⅰ-27. This indicates that compound Ⅰ-27 exhibits anti-tumor angiogenesis activity and effectively inhibits tumorigenesis and metastasis. The experimental results from the *in vivo* lung metastasis model demonstrate that the number of lung nodules in mice significantly decreased after treatment with different concentrations of compound Ⅰ-27. As a natural angiogenesis inhibitor, high expression of TSP1 can suppress the formation of tumor blood vessels. FoxO belongs to the forkhead box (FOX) transcription factor family and comprises four members in mammals: FoxO1, FoxO3, FoxO4, and FoxO6. These members share a conserved DNA-binding domain that binds to specific double-stranded DNA sites, including the DAF-16 binding element and the insulin-responsive element. FoxO is involved in various cellular functions, such as cell proliferation, apoptosis, differentiation, and DNA repair. FoxO1, recognized as a classic tumor suppressor gene within the FoxO family, also serves as an important regulator of blood vessel growth (Wilhelm et al., 2016). Its high expression can restrict blood vessel dilation and inhibit tumor growth and metastasis. To further verify that compound Ⅰ-27 inhibits tumor angiogenesis by regulating TSP-1 through ID1, we conducted experiments using tumor-bearing mice after knocking down ID1 in 4T1 cells. The results showed that after ID1 knockdown, the tumor volume was significantly reduced, the expression of angiogenesis markers was markedly decreased, the rate of tumor lung metastasis was significantly lowered, and the number of lung nodules was reduced. Further detection of TSP-1 expression revealed that after ID1 knockdown, TSP-1 expression was significantly increased. This further confirms that compound Ⅰ-27, as an ID1 inhibitor, regulates TSP-1 to effectively inhibit tumor tissue angiogenesis and suppress tumorigenesis and metastasis.

**FIGURE 10:**
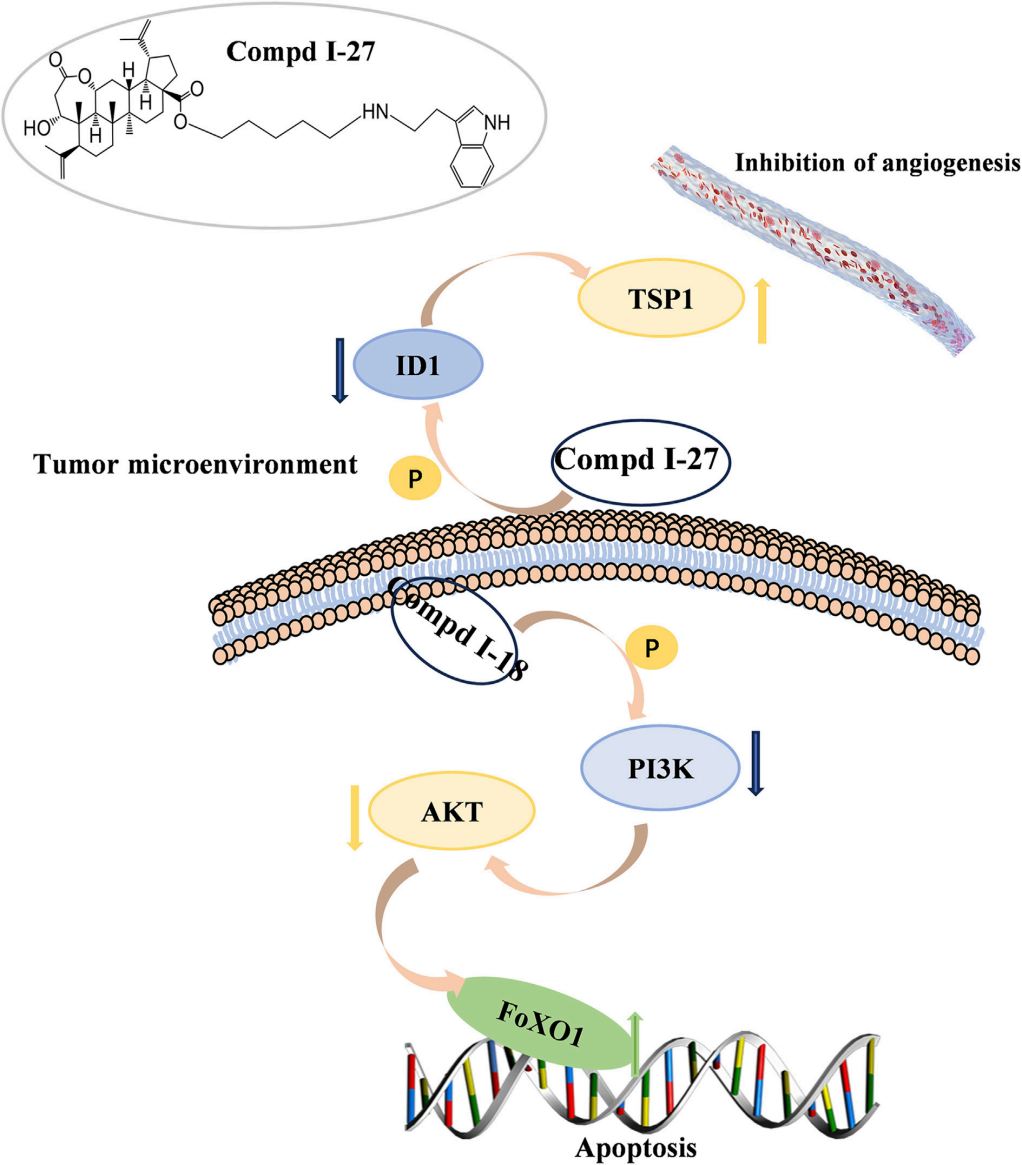
90 kinds of new chiisanoside derivatives were designed and synthesized. The connected tryptamine compound improves the anti-tumorigenesis of TNBC. Compound Ⅰ-27 can restore the anti-metastatic effect of TNBC. As a new tumor angiogenesis and invasion and metastasis inhibitor, compound Ⅰ-27 significantly resists damage from TNBC.

In summary, this study demonstrates that compound Ⅰ-27 is a newly designed and synthesized derivative with chiisanogenin as the lead compound. Compound Ⅰ-27 exhibits significant anti-tumor effects by targeting the tumor vascular system and inducing apoptosis, thereby inhibiting the growth and metastasis of MDA-MB-231 cells. Furthermore, we elucidated the mechanisms by which compound Ⅰ-27 inhibits TNBC tumor angiogenesis via the ID1/TSP-1 signaling pathway and induces TNBC cell apoptosis through the PI3K/AKT/FoxO1 signaling pathway (Figure 10). By inhibiting the key target of TNBC angiogenesis and apoptosis - ID1, compound Ⅰ-27 holds potential as a novel candidate drug for anti-TNBC therapy.

## Data Availability

The data presented in the study are deposited in the NCBI repository, accession number PRJNA1291846.
