# Supplementary material for: Design and synthesis of 3,4-seco-Lupane triterpene derivatives: targeting tumor angiogenesis and inducing apoptosis in triple-negative breast cancer
Source: Front Chem. 2025 Jul 31;13:1630939. doi: 10.3389/fchem.2025.1630939 (PMC12351286; doi:10.3389/fchem.2025.1630939)
Supplement: Supplementary file 1 [file DataSheet2.pdf]

Figure S1. *HPLC tracing of chiisanoside.*
Figure S2. *HPLC tracing of chiisanogenin(**I**).*
Figure S3. *HPLC tracing of compound **MH(II)**.*
Figure S4. *HPLC tracing of compound **I-1**.*
Figures S5. *^{13}C and ^{1}H NMR of compound **I-1**.*
Figure S6. *HPLC tracing of compound **I-2**.*
Figures S7. *^{13}C and ^{1}H NMR of compound **I-2**.*
Figure S8. *HPLC tracing of compound **I-3**.*
Figures S9. *^{13}C and ^{1}H NMR of compound **I-3**.*
Figure S10. *HPLC tracing of compound **I-4**.*
Figures S11. *^{13}C and ^{1}H NMR of compound **I-4**.*
Figure S12. *HPLC tracing of compound **I-5**.*
Figures S13. *^{13}C and ^{1}H NMR of compound **I-5**.*
Figure S14. *HPLC tracing of compound **I-6**.*
Figures S15. *^{13}C and ^{1}H NMR of compound **I-6**.*
Figure S16. *HPLC tracing of compound **I-**7.*
Figures S17. *^{13}C and ^{1}H NMR of compound **I-**7.*
Figure S18. *HPLC tracing of compound **I-8**.*
Figures S19. *^{13}C and ^{1}H NMR of compound **I-8**.*
Figure S20. *HPLC tracing of compound **I-9**.*
Figures S21. *^{13}C and ^{1}H NMR of compound **I-9**.*
Figure S22. *HPLC tracing of compound **I-10**.*
Figures S23. *^{13}C and ^{1}H NMR of compound **I-10**.*
Figure S24. *HPLC tracing of compound **I-11.***
Figures S25. *^{13}C and ^{1}H NMR of compound **I-11**.*
Figure S26. *HPLC tracing of compound **I-12.***
Figures S27. *^{13}C and ^{1}H NMR of compound **I-12**.*
Figure S28. *HPLC tracing of compound **I-13.***
Figures S29. *^{13}C and ^{1}H NMR of compound **I-13**.*
Figure S30. *HPLC tracing of compound **I-14.***
Figures S31. *^{13}C and ^{1}H NMR of compound **I-14**.*
Figure S32. *HPLC tracing of compound **I-15.***
Figures S33. *^{13}C and ^{1}H NMR of compound **I-15**.*
Figure S34. *HPLC tracing of compound **I-16.***
Figures S35. *^{13}C and ^{1}H NMR of compound **I-16**.*
Figure S36. *HPLC tracing of compound **I-1**7**.***
Figures S37. *^{13}C and ^{1}H NMR of compound **I-1**7.*
Figure S38. *HPLC tracing of compound **I-18.***
Figures S39. *^{13}C and ^{1}H NMR of compound **I-18**.*
Figure S40. *HPLC tracing of compound **I-19.***
Figures S41. *^{13}C and ^{1}H NMR of compound **I-19**.*
Figure S42. *HPLC tracing of compound **I-20.***
Figures S43. *^{13}C and ^{1}H NMR of compound **I-20**.*
Figure S44. *HPLC tracing of compound **I-21.***

Figures S45. *^{13}C and ^{1}H NMR of compound* ***I-21***.
Figure S46. *HPLC tracing of compound* ***I-22***
Figures S47. *^{13}C and ^{1}H NMR of compound* ***I-22***.
Figure S48. *HPLC tracing of compound* ***I-23.***
Figures S49. *^{13}C and ^{1}H NMR of compound* ***I-23***.
Figure S50. *HPLC tracing of compound* ***I-24.***
Figures S51. *^{13}C and ^{1}H NMR of compound* ***I-24***.
Figure S52. *HPLC tracing of compound* ***I-25.***
Figures S53. *^{13}C and ^{1}H NMR of compound* ***I-25***.
Figure S54. *HPLC tracing of compound* ***I-26.***
Figures S55. *^{13}C and ^{1}H NMR of compound* ***I-26***.
Figure S56. *HPLC tracing of compound* ***I-2****7*.
Figures S57. *^{13}C and ^{1}H NMR of compound* ***I-2****7*.
Figure S58. *HPLC tracing of compound* ***I-28***.
Figures S59. *^{13}C and ^{1}H NMR of compound* ***I-28***.
Figure S60. *HPLC tracing of compound* ***I-29***.
Figures S61. *^{13}C and ^{1}H NMR of compound* ***I-29***.
Figure S62. *HPLC tracing of compound* ***I-30***.
Figures S63. *^{13}C and ^{1}H NMR of compound* ***I-30***.
Figure S64. *HPLC tracing of compound* ***I-31***.
Figures S65. *^{13}C and ^{1}H NMR of compound* ***I-31***.
Figure S66. *HPLC tracing of compound* ***I-32***.
Figures S67. *^{13}C and ^{1}H NMR of compound* ***I-32***.
Figure S68. *HPLC tracing of compound* ***I-33***.
Figures S69. *^{13}C and ^{1}H NMR of compound* ***I-33***.
Figure S70. *HPLC tracing of compound* ***I-34***.
Figures S71. *^{13}C and ^{1}H NMR of compound* ***I-34***.
Figure S72. *HPLC tracing of compound* ***I-35***.
Figures S73. *^{13}C and ^{1}H NMR of compound* ***I-35***.
Figure S74. *HPLC tracing of compound* ***I-36***.
Figures S75. *^{13}C and ^{1}H NMR of compound* ***I-36***.
Figure S76. *HPLC tracing of compound* ***I-3****7*.
Figures S77. *^{13}C and ^{1}H NMR of compound* ***I-3****7*.
Figure S78. *HPLC tracing of compound* ***I-38***.
Figures S79. *^{13}C and ^{1}H NMR of compound* ***I-38***.
Figure S80. *HPLC tracing of compound* ***I-39***.
Figures S81. *^{13}C and ^{1}H NMR of compound* ***I-39***.
Figure S82. *HPLC tracing of compound* ***I-40***.
Figures S83. *^{13}C and ^{1}H NMR of compound* ***I-40***.
Figure S84. *HPLC tracing of compound* ***I-41***.
Figures S85. *^{13}C and ^{1}H NMR of compound* ***I-41***.
Figure S86. *HPLC tracing of compound* ***I-42***.
Figures S87. *^{13}C and ^{1}H NMR of compound* ***I-42***.
Figure S88. *HPLC tracing of compound* ***I-43.***

Figures S89. *^{13}C and ^{1}H NMR of compound* ***I-43***.
Figure S90. *HPLC tracing of compound* ***I-44***.
Figures S91. *^{13}C and ^{1}H NMR of compound* ***I-44***.
Figure S92. *HPLC tracing of compound* ***I-45***.
Figures S93. *^{13}C and ^{1}H NMR of compound* ***I-45***.
Figure S94. *HPLC tracing of compound* ***II-46***.
Figures S95. *^{13}C and ^{1}H NMR of compound* ***II-46***.
Figure S96. *HPLC tracing of compound* ***II-47***
Figures S97. *^{13}C and ^{1}H NMR of compound* ***II-47***.
Figure S98. *HPLC tracing of compound* ***II-48***.
Figures S99. *^{13}C and ^{1}H NMR of compound* ***II-48***.
Figure S100. *HPLC tracing of compound* ***II-49***.
Figures S101. *^{13}C and ^{1}H NMR of compound* ***II-49***.
Figure S102. *HPLC tracing of compound* ***II-50***.
Figures S103. *^{13}C and ^{1}H NMR of compound* ***II-50***.
Figure S104. *HPLC tracing of compound* ***II-51***.
Figures S105. *^{13}C and ^{1}H NMR of compound* ***II-51***.
Figure S106. *HPLC tracing of compound* ***II-52***.
Figures S107. *^{13}C and ^{1}H NMR of compound* ***II-52***.
Figure S108. *HPLC tracing of compound* ***II-53***.
Figures S109. *^{13}C and ^{1}H NMR of compound* ***II-53***.
Figure S110. *HPLC tracing of compound* ***II-54***.
Figures S111. *^{13}C and ^{1}H NMR of compound* ***II-54***.
Figure S112. *HPLC tracing of compound* ***II-55***.
Figures S113. *^{13}C and ^{1}H NMR of compound* ***II-55***.
Figure S114. *HPLC tracing of compound* ***II-56***.
Figures S115. *^{13}C and ^{1}H NMR of compound* ***II-56***.
Figure S116. *HPLC tracing of compound* ***II-57***.
Figures S117. *^{13}C and ^{1}H NMR of compound* ***II-57***.
Figure S118. *HPLC tracing of compound* ***II-58***.
Figures S119. *^{13}C and ^{1}H NMR of compound* ***II-58***.
Figure S120. *HPLC tracing of compound* ***II-59***.
Figures S121. *^{13}C and ^{1}H NMR of compound* ***II-59***.
Figure S122. *HPLC tracing of compound* ***II-60***.
Figures S123. *^{13}C and ^{1}H NMR of compound* ***II-60***.
Figure S124. *HPLC tracing of compound* ***II-61***.
Figures S125. *^{13}C and ^{1}H NMR of compound* ***II-61***.
Figure S126. *HPLC tracing of compound* ***II-62***.
Figures S127. *^{13}C and ^{1}H NMR of compound* ***II-62***.
Figure S128. *HPLC tracing of compound* ***II-63***.
Figures S129. *^{13}C and ^{1}H NMR of compound* ***II-63***.
Figure S130. *HPLC tracing of compound* ***II-64***.
Figures S131. *^{13}C and ^{1}H NMR of compound* ***II-64***.
Figure S132. *HPLC tracing of compound* ***II-65***.

Figures S133. *^{13}C and ^{1}H NMR of compound **II-65***.
Figure S134. *HPLC tracing of compound **II-66***.
Figures S135. *^{13}C and ^{1}H NMR of compound **II-66***.
Figure S136. *HPLC tracing of compound **II-67***.
Figures S137. *^{13}C and ^{1}H NMR of compound **II-67***.
Figure S138. *HPLC tracing of compound **II-68***.
Figures S139. *^{13}C and ^{1}H NMR of compound **II-68***.
Figure S140. *HPLC tracing of compound **II-69***.
Figures S141. *^{13}C and ^{1}H NMR of compound **II-69***.
Figure S142. *HPLC tracing of compound **II-70***.
Figures S143. *^{13}C and ^{1}H NMR of compound **II-70***.
Figure S144. *HPLC tracing of compound **II-71***.
Figures S145. *^{13}C and ^{1}H NMR of compound **II-71***.
Figure S146. *HPLC tracing of compound **II-72***.
Figures S147. *^{13}C and ^{1}H NMR of compound **II-72***.
Figure S148. *HPLC tracing of compound **II-73***.
Figures S149. *^{13}C and ^{1}H NMR of compound **II-73***.
Figure S150. *HPLC tracing of compound **II-74***.
Figures S151. *^{13}C and ^{1}H NMR of compound **II-74***.
Figure S152. *HPLC tracing of compound **II-75***.
Figures S153. *^{13}C and ^{1}H NMR of compound **II-75***.
Figure S154. *HPLC tracing of compound **II-76***.
Figures S155. *^{13}C and ^{1}H NMR of compound **II-76***.
Figure S156. *HPLC tracing of compound **II-77***.
Figures S157. *^{13}C and ^{1}H NMR of compound **II-77***.
Figure S158. *HPLC tracing of compound **II-78***.
Figures S159. *^{13}C and ^{1}H NMR of compound **II-78***.
Figure S160. *HPLC tracing of compound **II-79***.
Figures S161. *^{13}C and ^{1}H NMR of compound **II-79***.
Figure S162. *HPLC tracing of compound **II-80***.
Figures S163. *^{13}C and ^{1}H NMR of compound **II-80***.
Figure S164. *HPLC tracing of compound **II-81***.
Figures S165. *^{13}C and ^{1}H NMR of compound **II-81***.
Figure S166. *HPLC tracing of compound **II-82***.
Figures S167. *^{13}C and ^{1}H NMR of compound **II-82***.
Figure S168. *HPLC tracing of compound **II-83***.
Figures S169. *^{13}C and ^{1}H NMR of compound **II-83***.
Figure S170. *HPLC tracing of compound **II-84***.
Figures S171. *^{13}C and ^{1}H NMR of compound **II-84***.
Figure S172. *HPLC tracing of compound **II-85***.
Figures S173. *^{13}C and ^{1}H NMR of compound **II-85***.
Figure S174. *HPLC tracing of compound **II-86***.
Figures S175. *^{13}C and ^{1}H NMR of compound **II-86***.
Figure S176. *HPLC tracing of compound **II-87***.

Figures S177. *^{13}C and ^{1}H NMR of compound **II-87**.*
Figure S178. *HPLC tracing of compound **II-88**.*
Figures S179. *^{13}C and ^{1}H NMR of compound **II-88**.*
Figure S180. *HPLC tracing of compound **II-89**.*
Figures S181. *^{13}C and ^{1}H NMR of compound **II-89**.*
Figure S182. *HPLC tracing of compound **II-90**.*
Figures S183. *^{13}C and ^{1}H NMR of compound **II-90**.*

Table S2. Primer pair sequences of target genes.

Gene Symbol	Sequence	
	FORWARD	REVERSE
Pik3r2	ATGGCGGGCCCTGAGGGCTT	GCGGGCGGCAGGCGGCGGGC
Akt1	GTGATCCTGGTGAAGGAGA	TTAATGTGCCCGTCCTTGT
Id1	GGTAAACGTGCTGCTCTACG	GATCTGGATCTCACCTCGGC
THBS-1	AGACTCCGCATCGCAAAGG	TCACCACGTTGTTGTCAAGGG
FOXO1	TCGTCATAATCTGTCCCTACACA	CACCATTGGCAATGAGCGGTTC
β-actin	CACCATTGGCAATGAGCGGTTC	AGGTCTTTGCGGATGTCCACGT

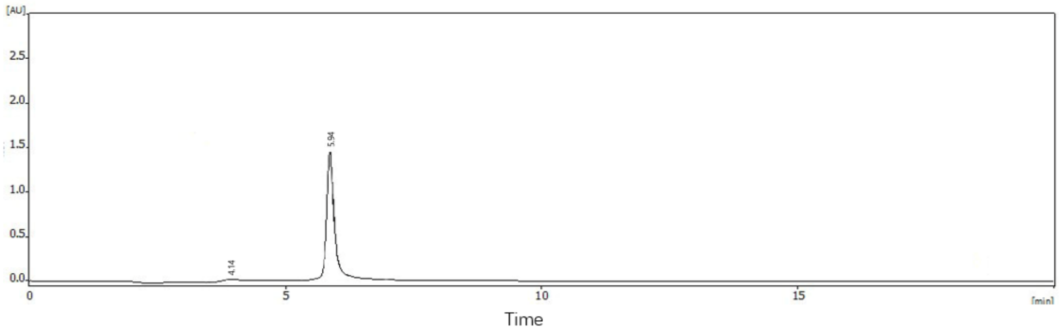

	RT	Area	Area %	Height
1	5.935	32486.349	98.1	2367.922
2	4.136	629.196	1.9	36.791

Time	A (water,0.5% Phosphoric acid)	B (Acetonitrile)
0	65	35
10	10	90
30	10	90
40	65	35

Figure S1. *HPLC tracing of chiisanoside.*

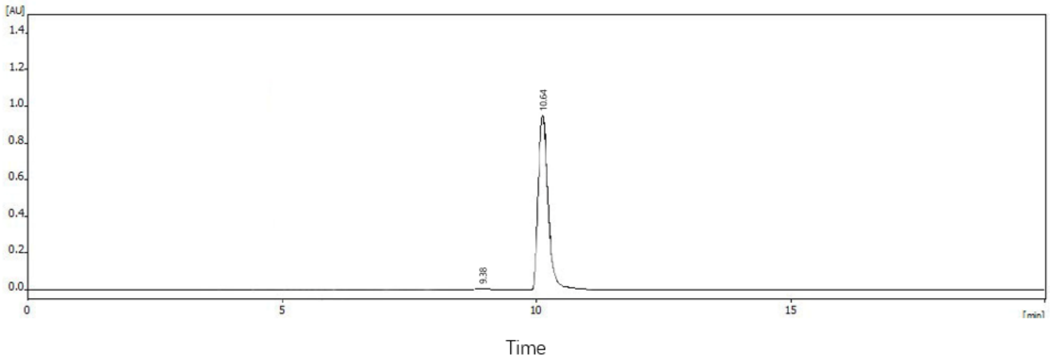

	RT	Area	Area %	Height
1	10.642	42655.348	97.1	3417.694
2	9.375	1273.950	2.9	259.694

Time	A (water,0.5% Phosphoric acid)	B (Acetonitrile)
0	65	35
10	10	90
30	10	90
40	65	35

Figure S2. *HPLC tracing of chiisanogenin(I).*

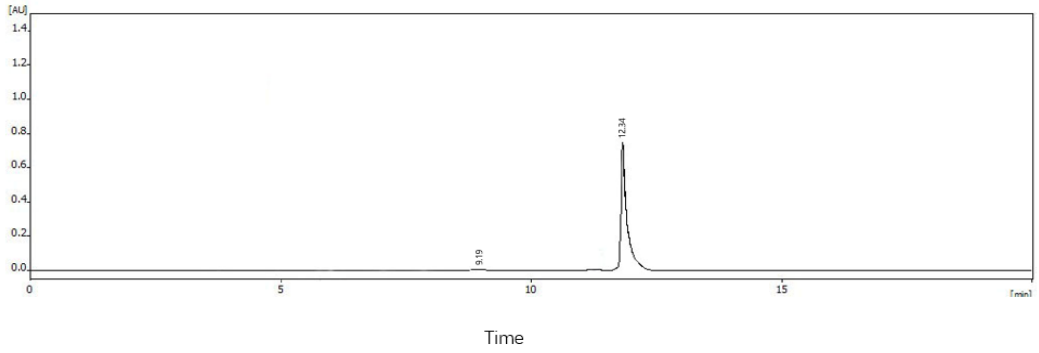

	RT	Area	Area %	Height
1	12.341	28679.649	98.5	2567.911
2	9.192	844.375	1.5	113.952

Time	A (water,0.5% Phosphoric acid)	B (Acetonitrile)
0	65	35
10	10	90
30	10	90
40	65	35

Figure S3. *HPLC tracing of compound* ***MH(II)***.

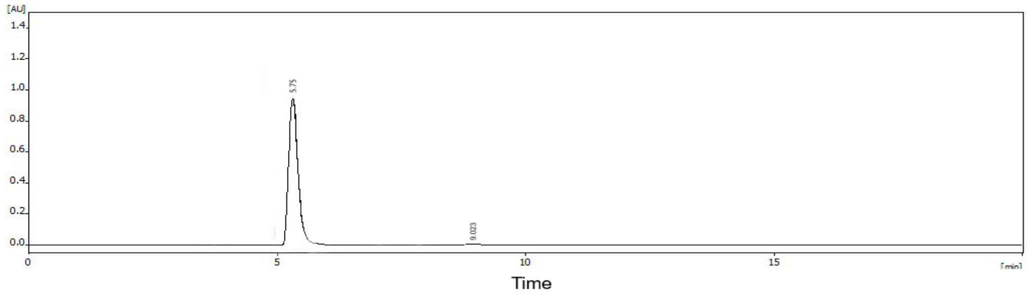

	RT	Area	Area %	Height
1	5.750	21564.247	97.1	1498.572
2	9.023	644.040	2.9	13.671

Time	A (water,0.5% Phosphoric acid)	B (Acetonitrile)
0	65	35
10	10	90
30	10	90
40	65	35

Figure S4. *HPLC tracing of compound **I-1**.*

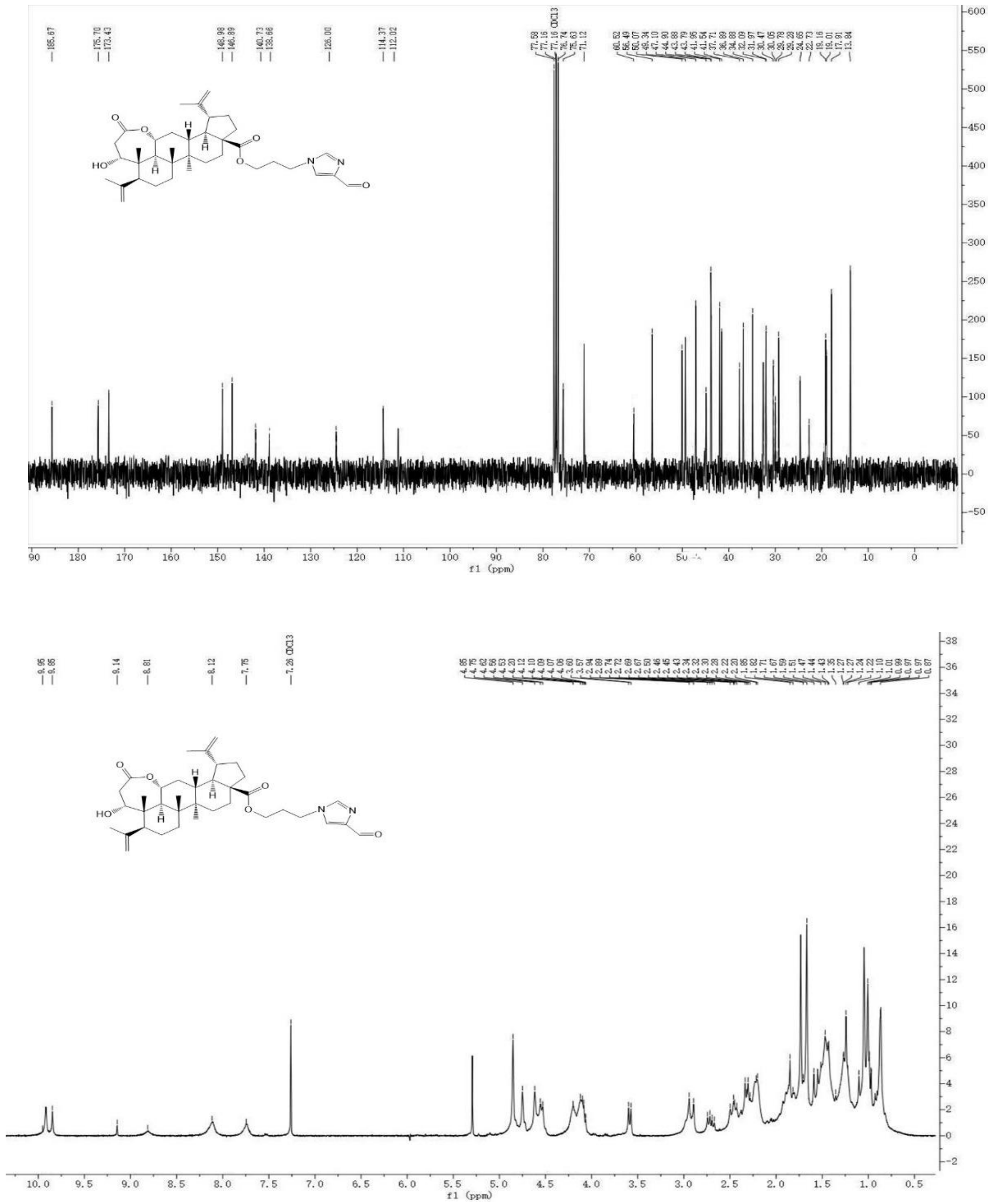

Figures S5. *^{13}C and ^{1}H NMR of compound **I-1**.*

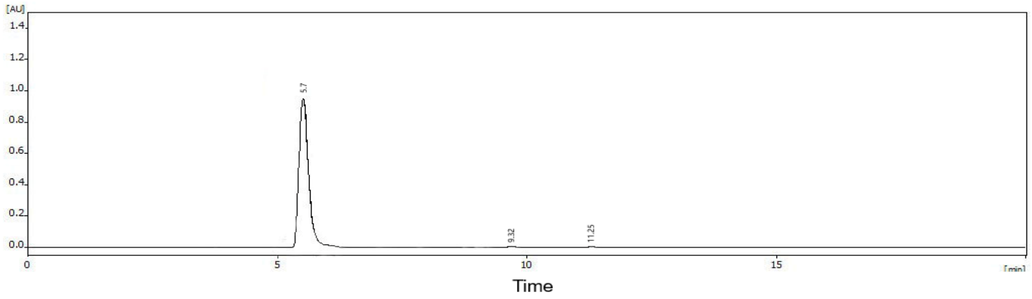

	RT	Area	Area %	Height
1	5.698	21619.247	97.0	1646.572
2	9.320	289.742	1.3	10.256
3	11.247	378.894	1.7	5.364

Time	A (water,0.5% Phosphoric acid)	B (Acetonitrile)
0	65	35
10	10	90
30	10	90
40	65	35

Figure S6. *HPLC tracing of compound **I-2**.*

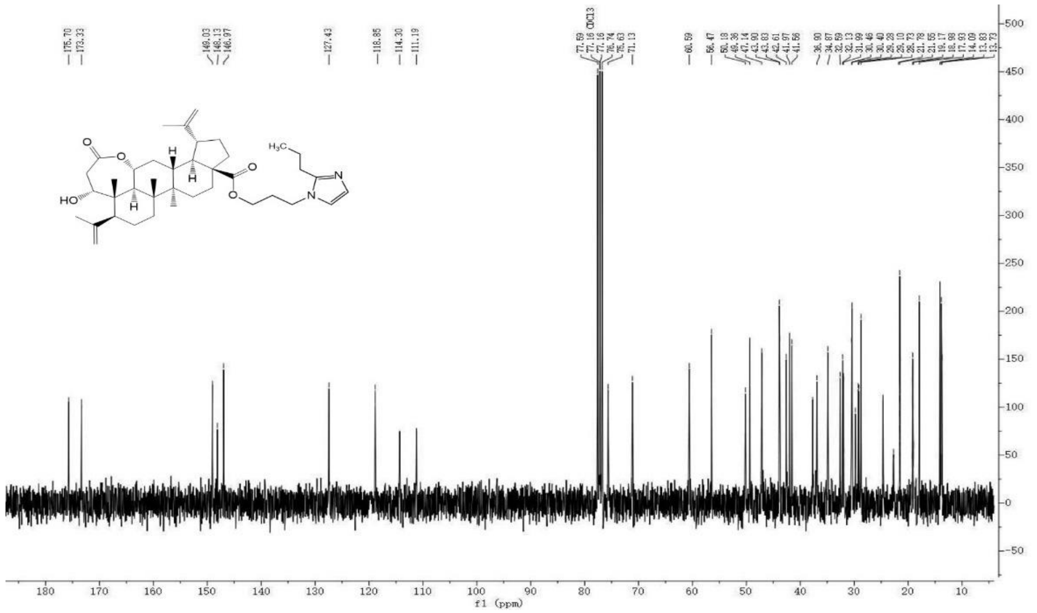

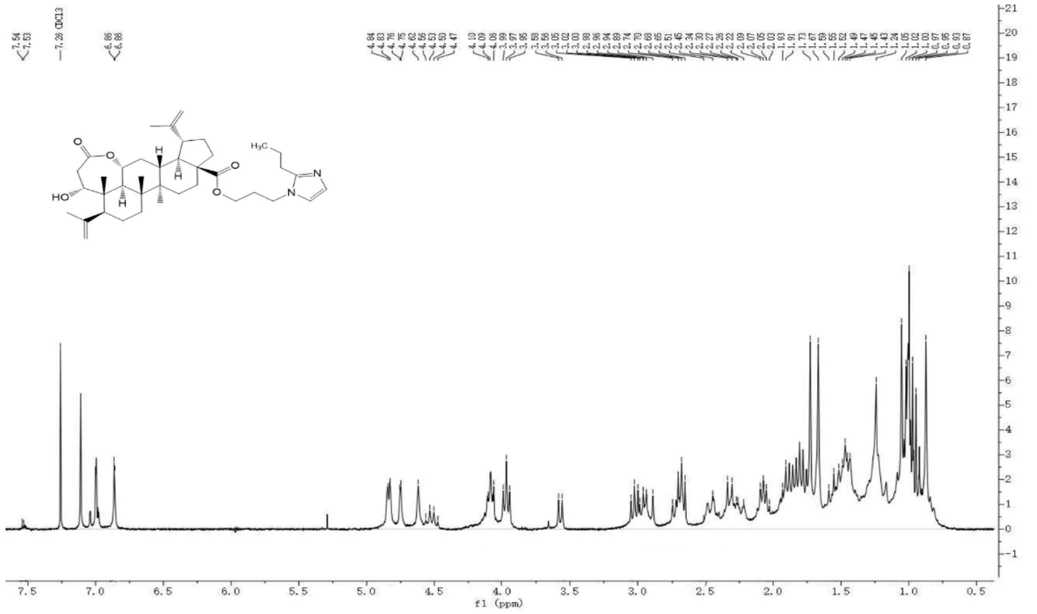

Figures S7. *^{13}C and ^{1}H NMR of compound **I-2**.*

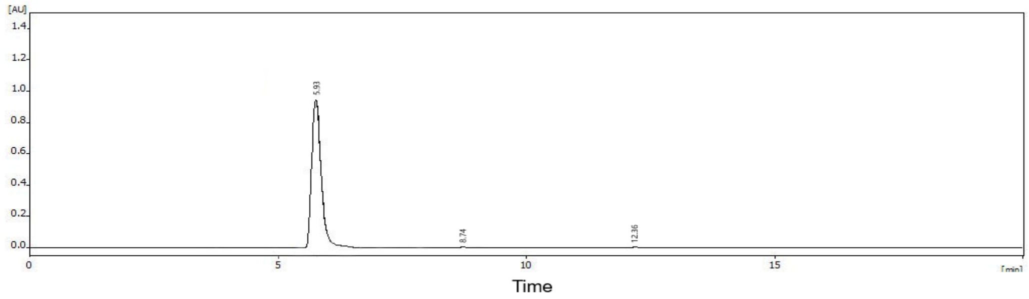

	RT	Area	Area %	Height
1	5.934	46823.658	97.8	1359.195
2	8.740	622.400	1.3	25.648
3	12.360	430.892	0.9	20.547

Time	A (water,0.5% Phosphoric acid)	B (Acetonitrile)
0	65	35
10	10	90
30	10	90
40	65	35

Figure S8. *HPLC tracing of compound* ***I-3***.

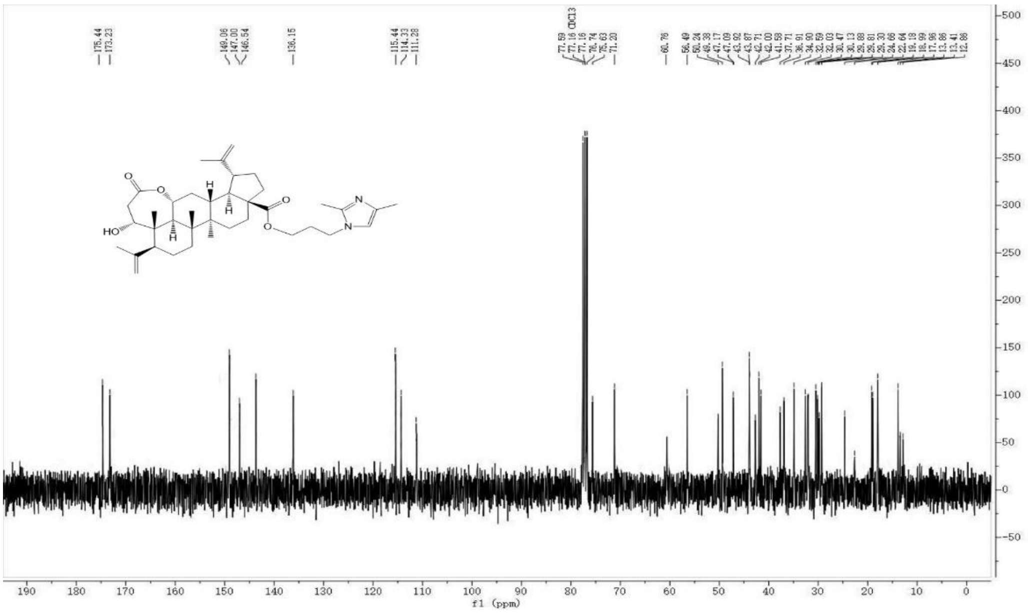

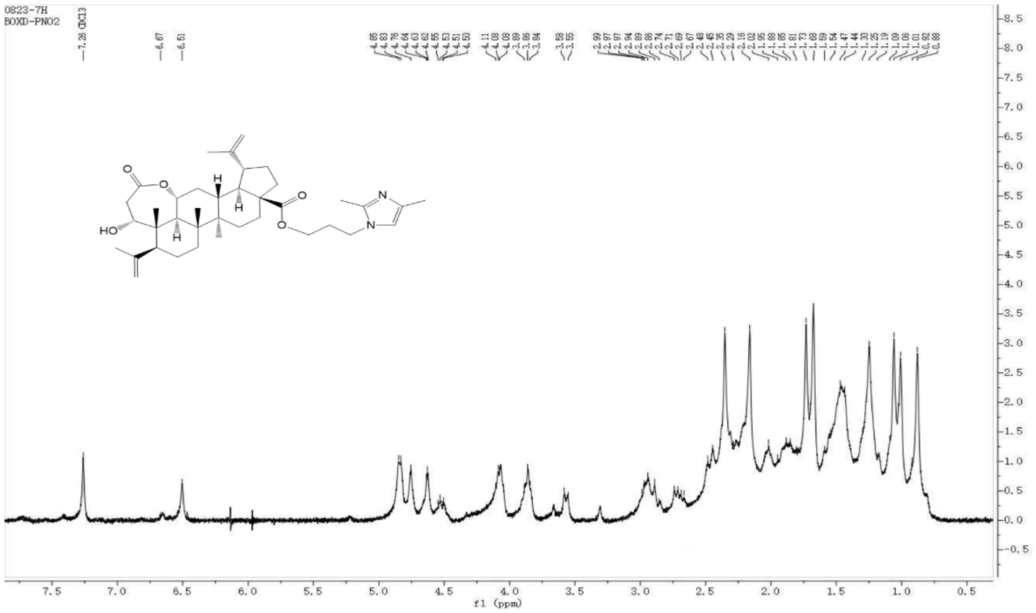

Figures S9. *^{13}C and ^{1}H NMR of compound* ***I-3***.

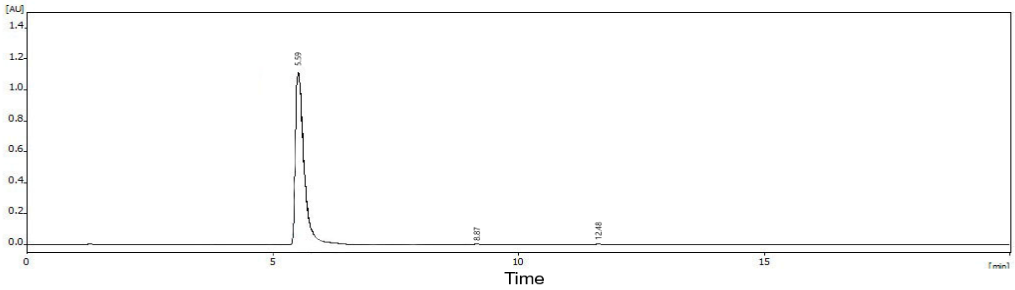

	RT	Area	Area %	Height
1	5.591	247.549	0.7	26.491
2	8.872	34692.289	98.1	1568.563
3	12.479	424.371	1.2	14.594

Time	A (water,0.5% Phosphoric acid)	B (Acetonitrile)
0	65	35
10	10	90
30	10	90
40	65	35

Figure S10. *HPLC tracing of compound **I-4**.*

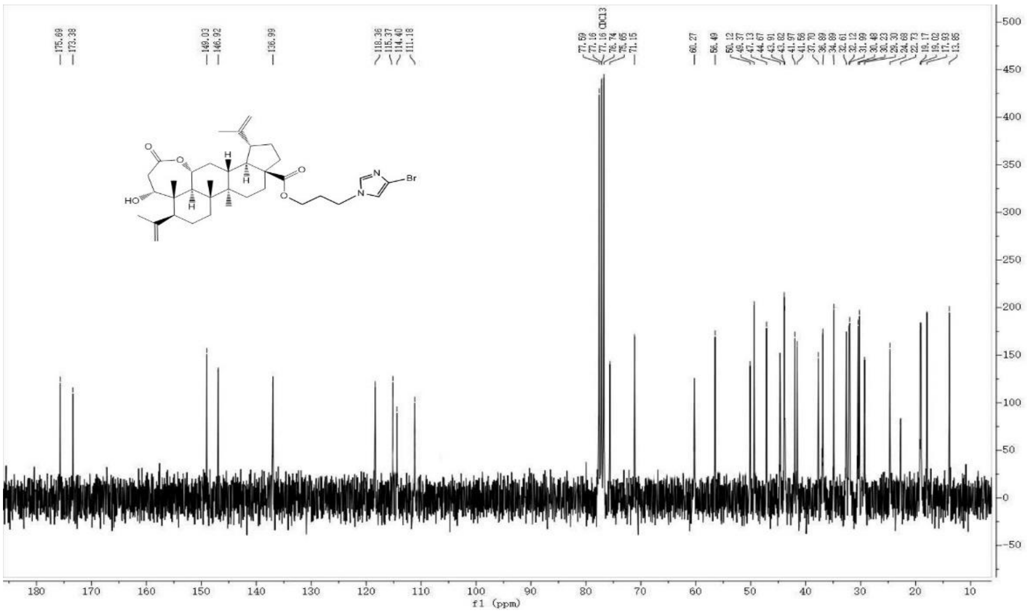

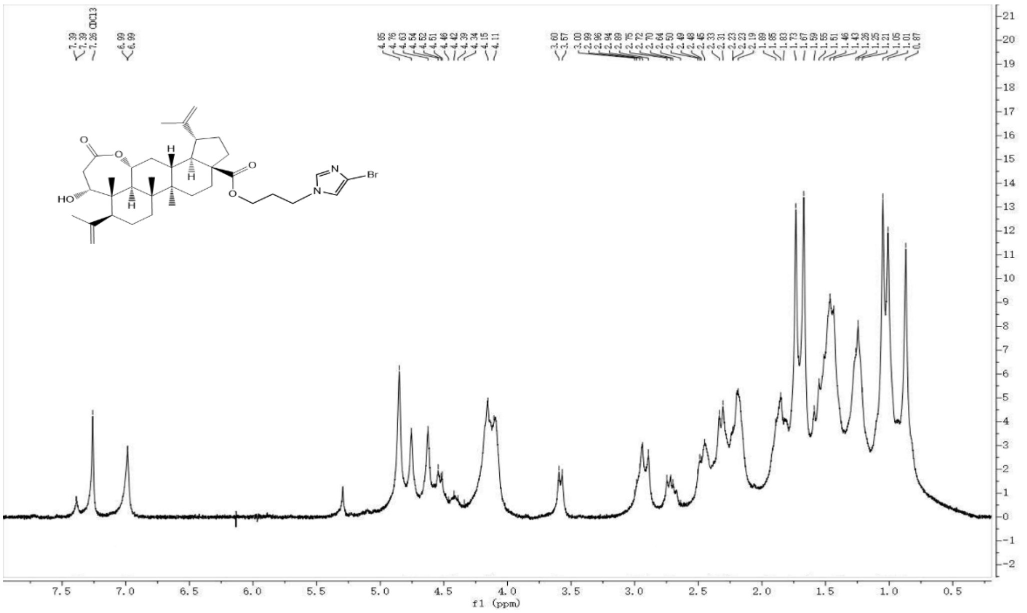

Figures S11. *^{13}C and ^{1}H NMR of compound **I-4**.*

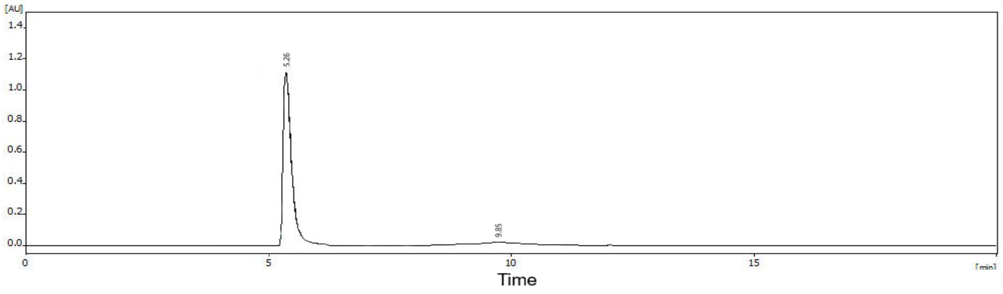

	RT	Area	Area %	Height
1	5.262	45969.189	98.4	2648.251
2	9.850	747.466	1.6	32.156

Time	A (water,0.5% Phosphoric acid)	B (Acetonitrile)
0	65	35
10	10	90
30	10	90
40	65	35

Figure S12. *HPLC tracing of compound* ***I-5****.*

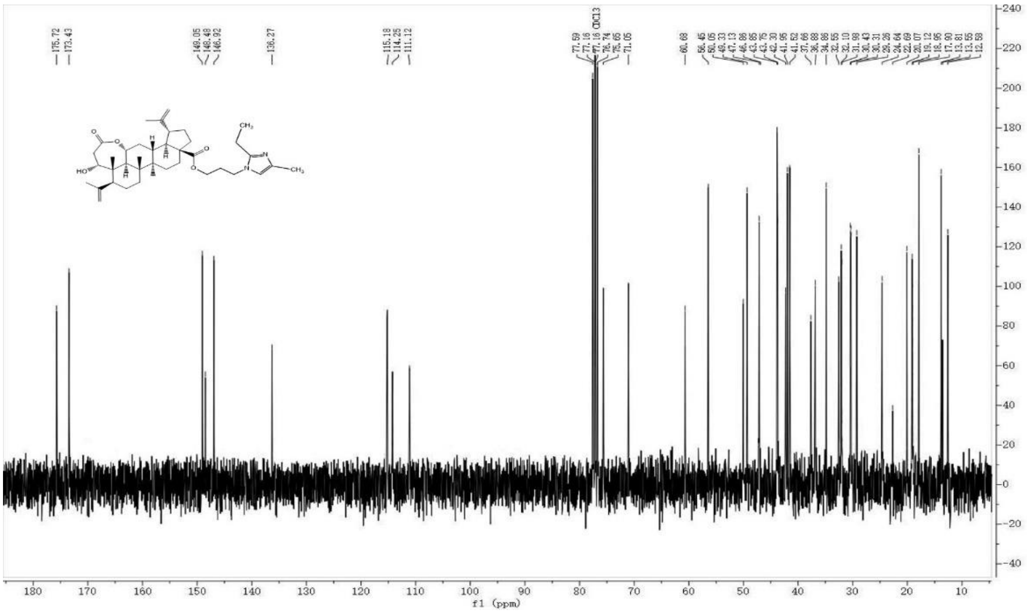

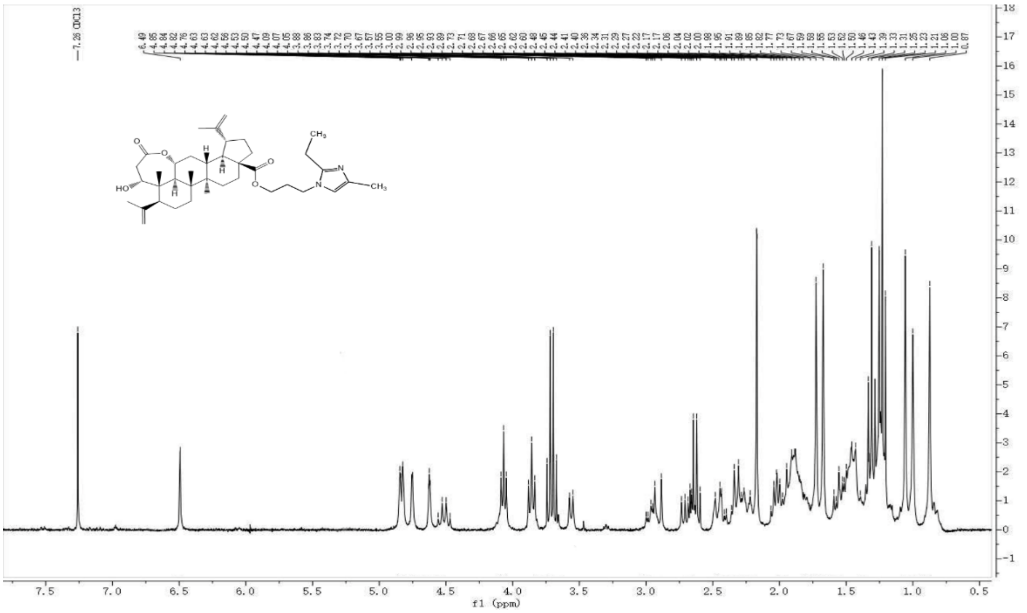

Figures S13. ^{13}*C and* ^{1}*H NMR of compound* ***I-5****.*

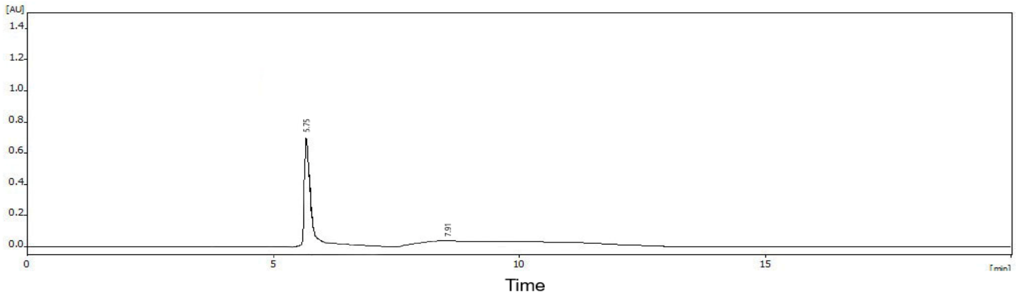

	RT	Area	Area %	Height
1	5.749	26947.269	97.5	2516.891
2	7.913	468.159	2.5	165.899

Time	A (water,0.5% Phosphoric acid)	B (Acetonitrile)
0	65	35
10	10	90
30	10	90
40	65	35

Figure S14. *HPLC tracing of compound **I-6**.*

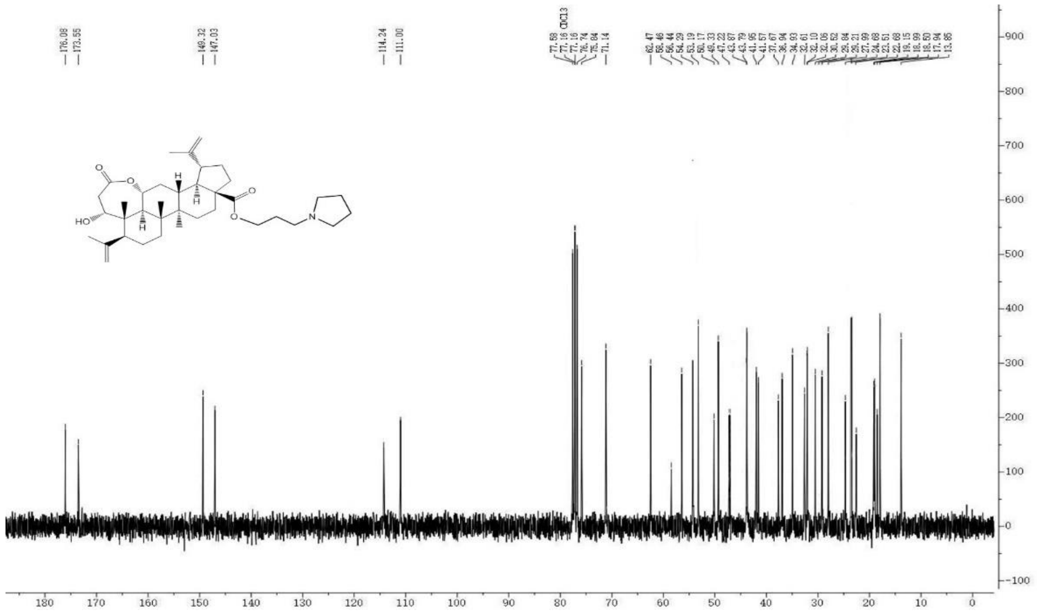

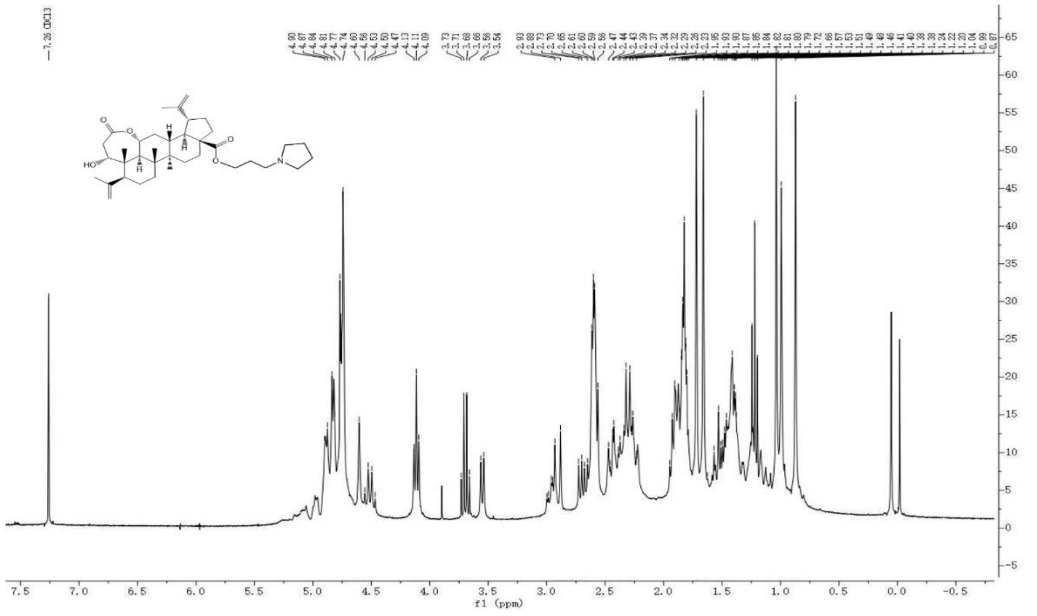

Figures S15. *^{13}C and ^{1}H NMR of compound **I-6**.*

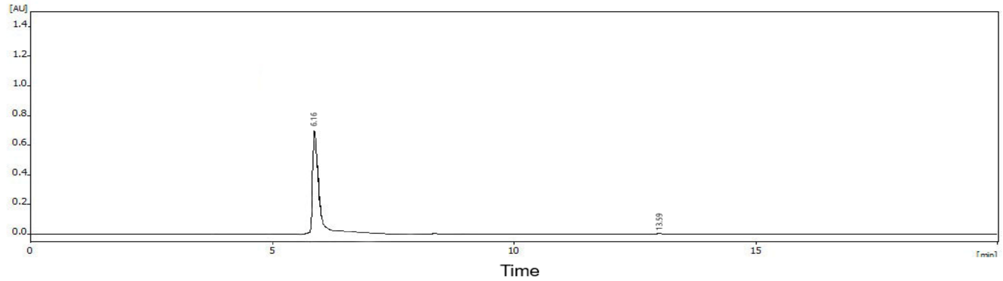

	RT	Area	Area %	Height
1	6.157	34896.296	98.3	2364.921
2	13.593	603.496	1.7	63.289

Time	A (water,0.5% Phosphoric acid)	B (Acetonitrile)
0	65	35
10	10	90
30	10	90
40	65	35

Figure S16. *HPLC tracing of compound **I-7**.*

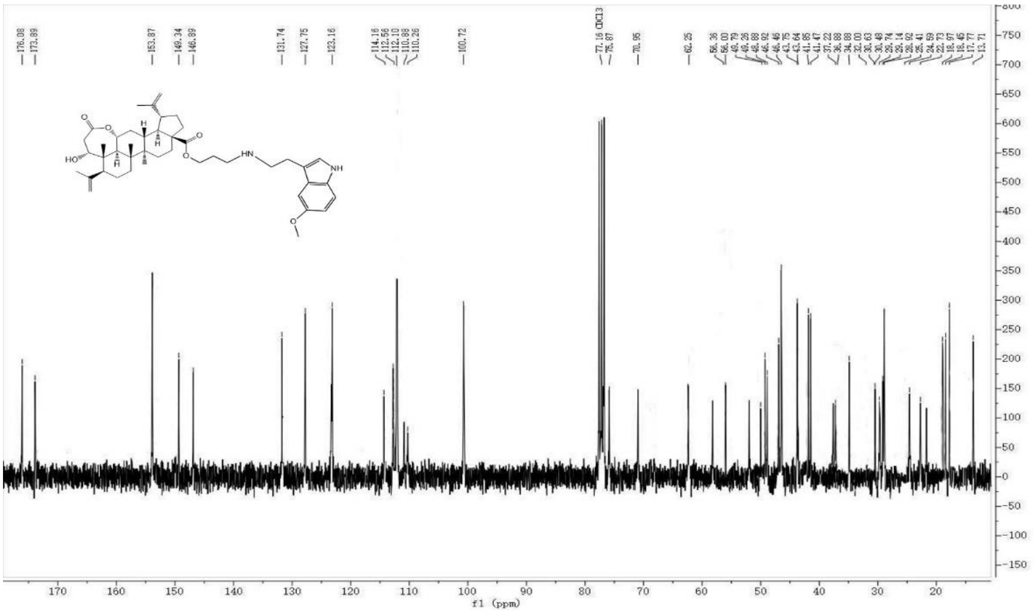

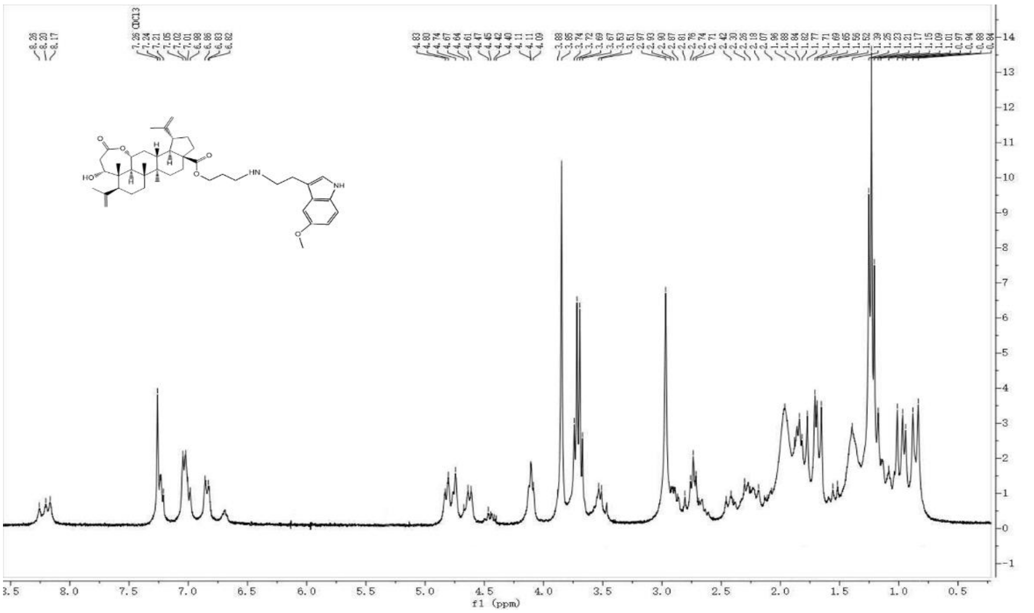

Figures S17. *^{13}C and ^{1}H NMR of compound **I-7**.*

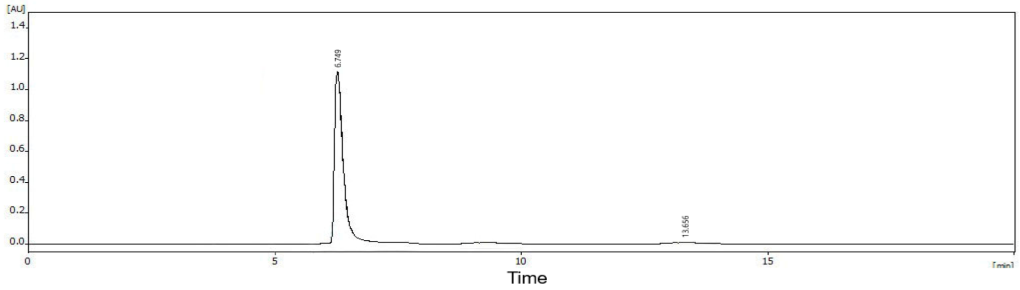

	RT	Area	Area %	Height
1	6.749	25684.952	98.6	1648.694
2	13.656	364.695	1.4	34.591

Time	A (water,0.5% Phosphoric acid)	B (Acetonitrile)
0	65	35
10	10	90
30	10	90
40	65	35

Figure S18. *HPLC tracing of compound* ***I-8****.*

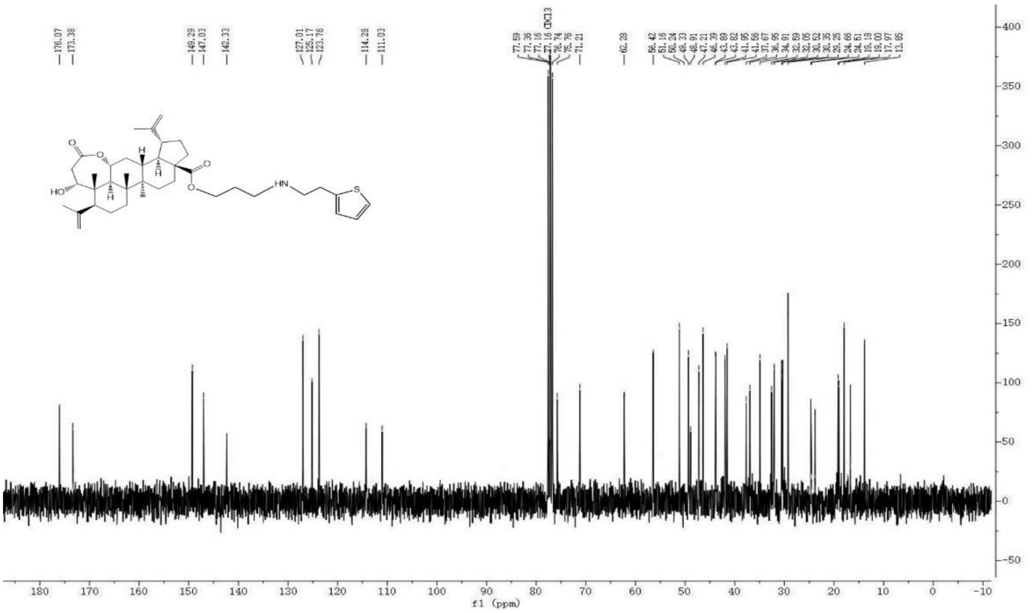

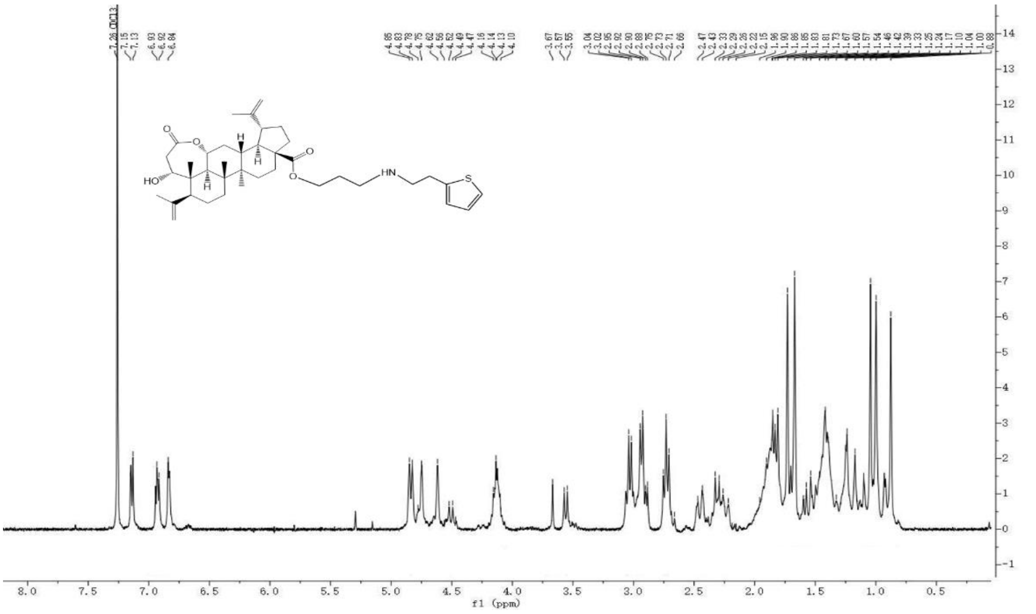

Figures S19. *^{13}C and ^{1}H NMR of compound* ***I-8****.*

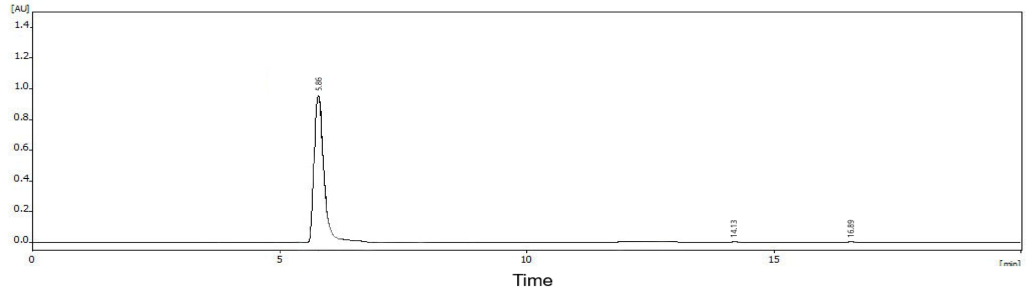

	RT	Area	Area %	Height
1	5.864	538.539	1.5	159.155
2	14.128	34897.359	97.2	1646.572
3	16.89	466.734	1.3	69.891

Time	A (water,0.5% Phosphoric acid)	B (Acetonitrile)
0	65	35
10	10	90
30	10	90
40	65	35

Figure S20. *HPLC tracing of compound **I-9**.*

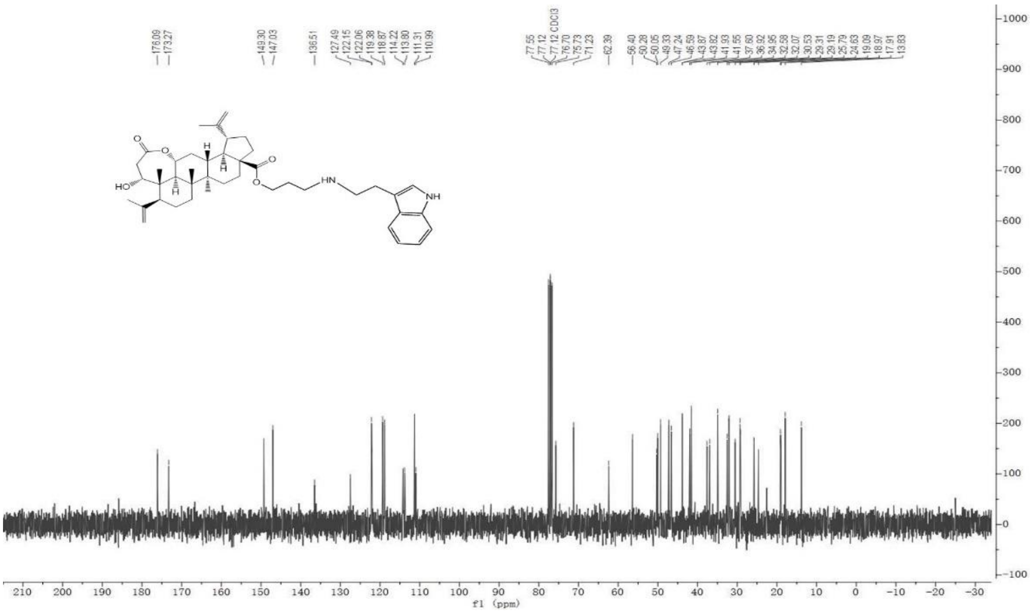

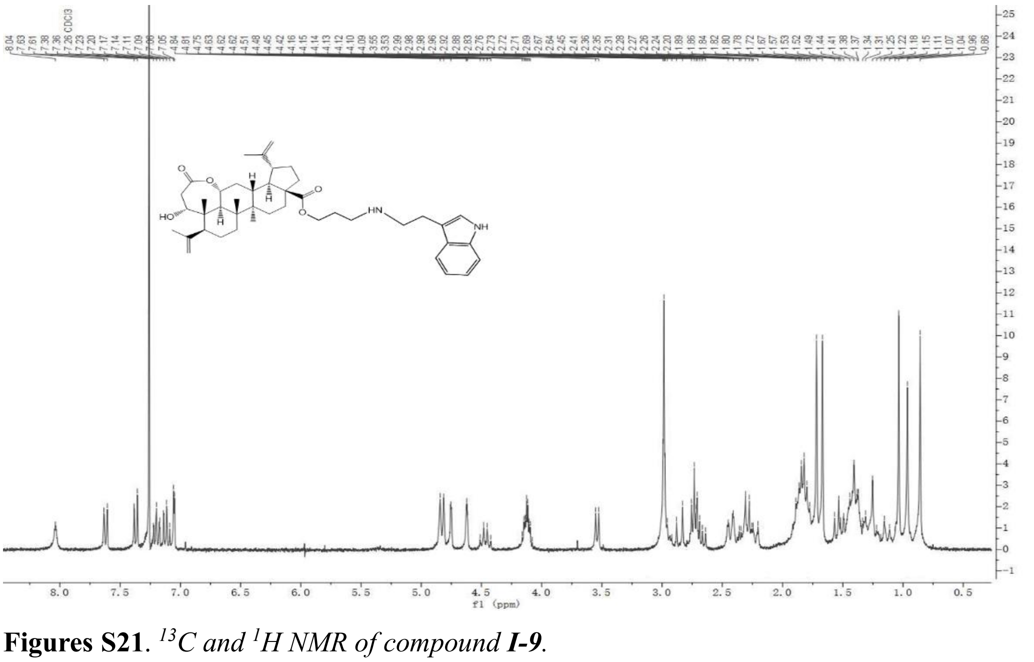

Figures S21. *^{13}C and ^{1}H NMR of compound **I-9**.*

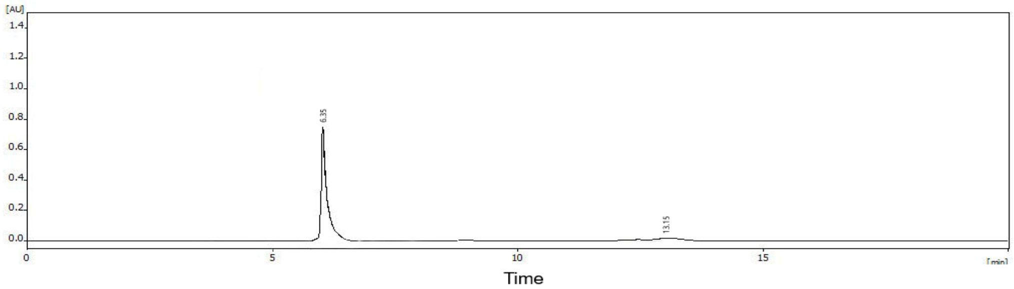

	RT	Area	Area %	Height
1	6.357	25946.194	98.6	3452.188
2	13.154	3783.820	1.4	23.548

Time	A (water,0.5% Phosphoric acid)	B (Acetonitrile)
0	65	35
10	10	90
30	10	90
40	65	35

Figure S22. *HPLC tracing of compound **I-10**.*

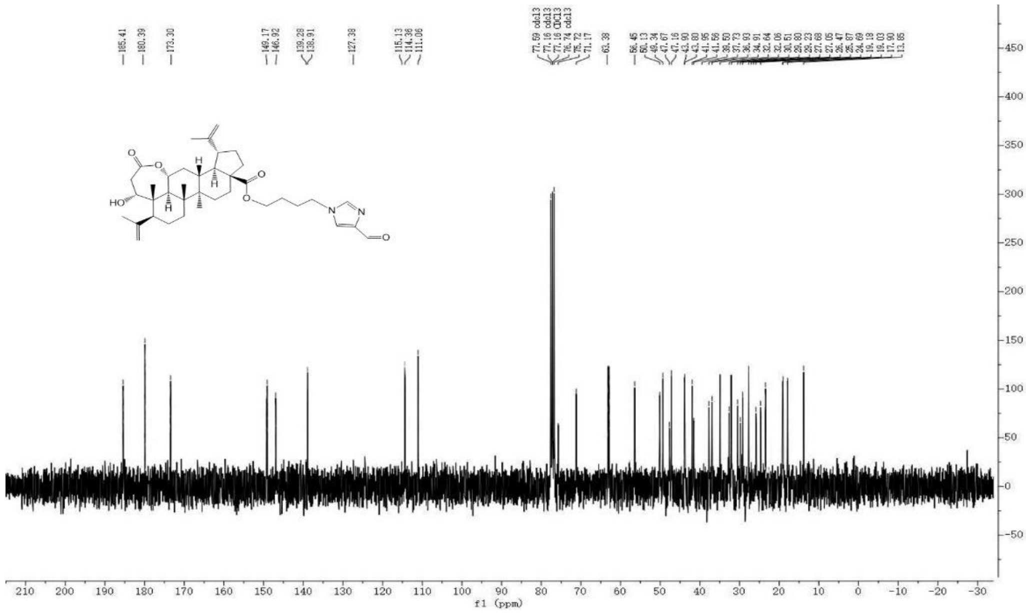

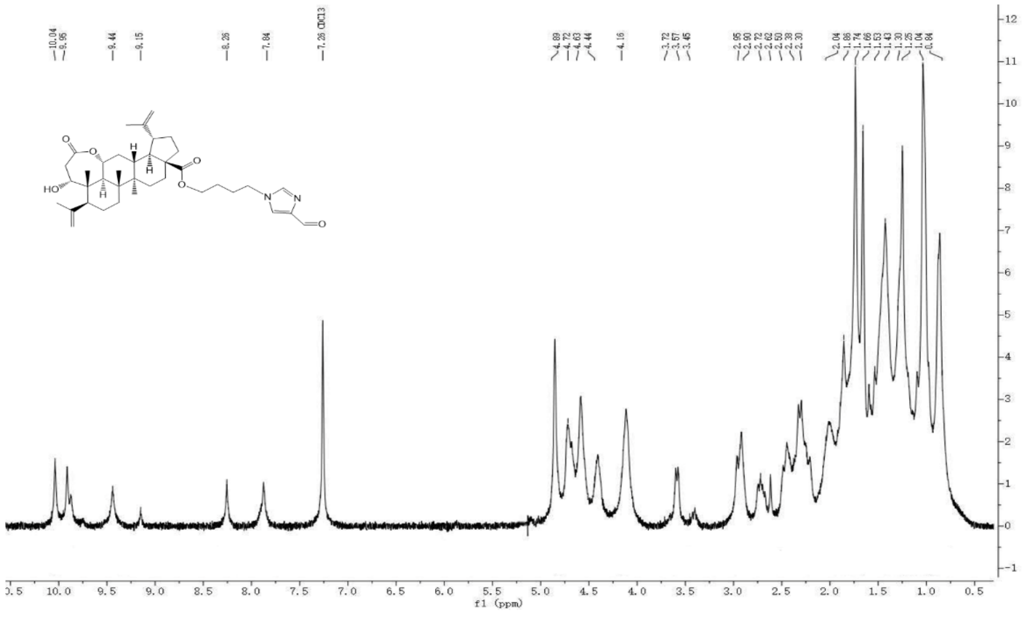

Figures S23. *^{13}C and ^{1}H NMR of compound **I-10**.*

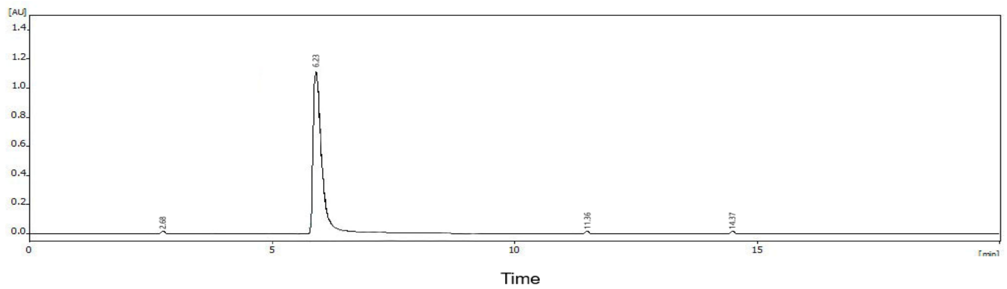

	RT	Area	Area %	Height
1	2.678	360.703	1.1	25.891
2	6.2334	31545.156	96.2	2614.159
3	11.359	426.286	1.3	26.970
4	14.369	459.077	1.4	54.150

Time	A (water,0.5% Phosphoric acid)	B (Acetonitrile)
0	65	35
10	10	90
30	10	90
40	65	35

Figure S24. *HPLC tracing of compound* ***I-11.***

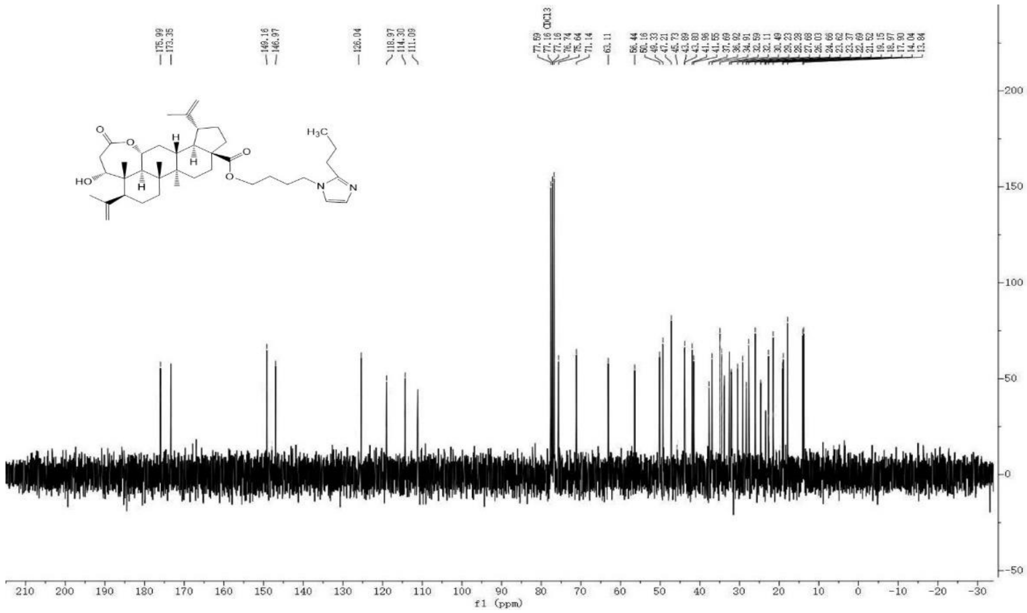

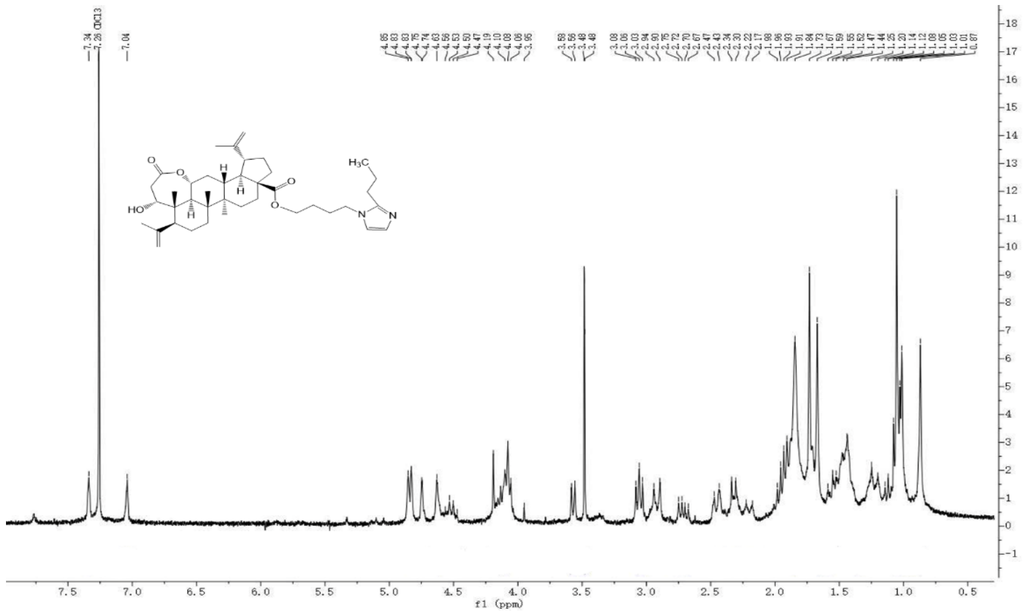

Figures S25. *^{13}C and ^{1}H NMR of compound* ***I-11***.

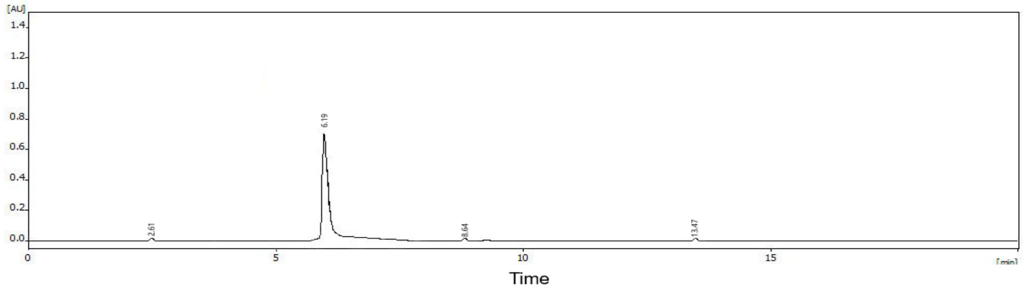

	RT	Area	Area %	Height
1	2.613	321.728	1.0	13.568
2	6.193	31561.561	98.1	3315.156
3	8.642	193.037	0.6	91.157
4	13.469	96.519	0.3	3.596

Time	A (water,0.5% Phosphoric acid)	B (Acetonitrile)
0	65	35
10	10	90
30	10	90
40	65	35

Figure S26. *HPLC tracing of compound **I-12.***

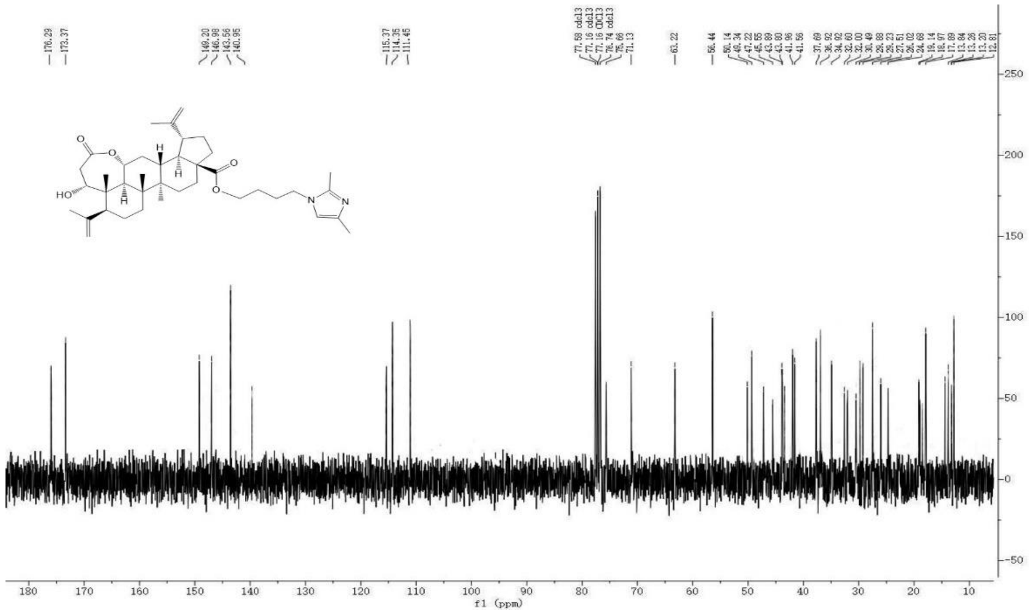

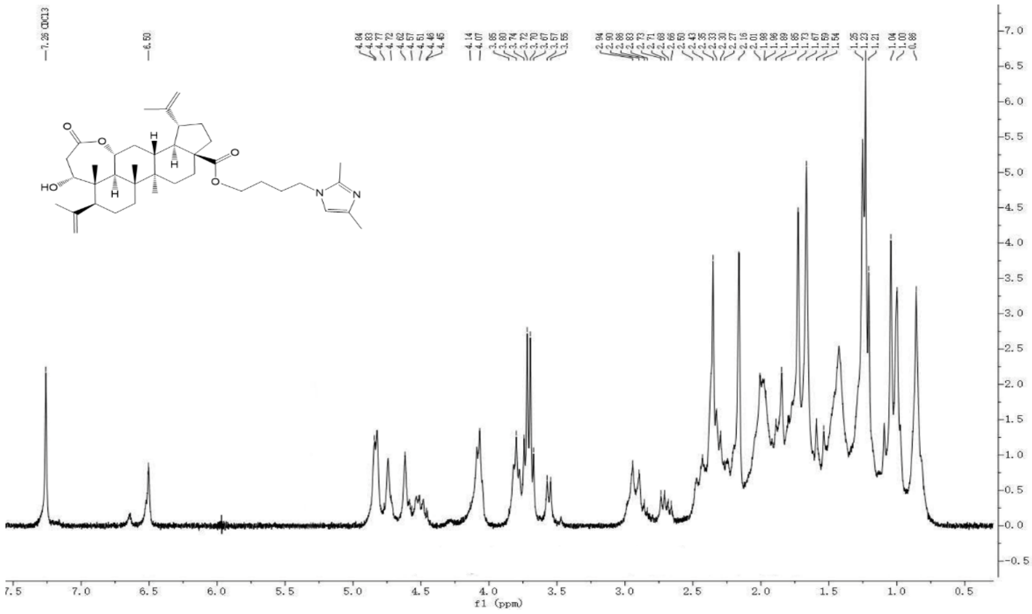

Figures S27. *^{13}C and ^{1}H NMR of compound **I-12**.*

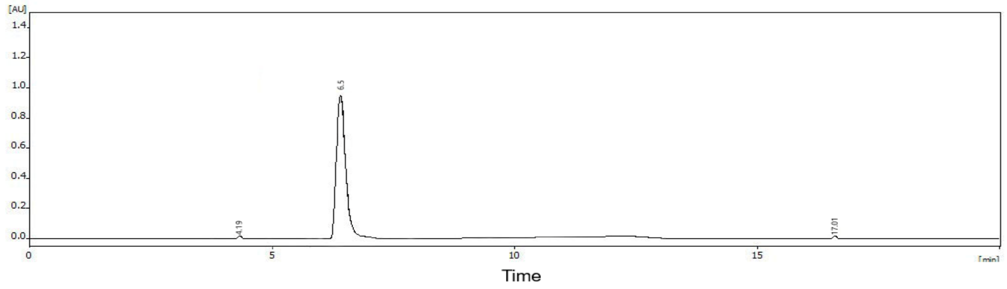

	RT	Area	Area %	Height
1	4.187	215.330	0.6	32.152
2	6.496	35314.158	98.4	2641.521
3	17.010	358.883	1.0	321.891

Time	A (water,0.5% Phosphoric acid)	B (Acetonitrile)
0	65	35
10	10	90
30	10	90
40	65	35

Figure S28. *HPLC tracing of compound **I-13.***

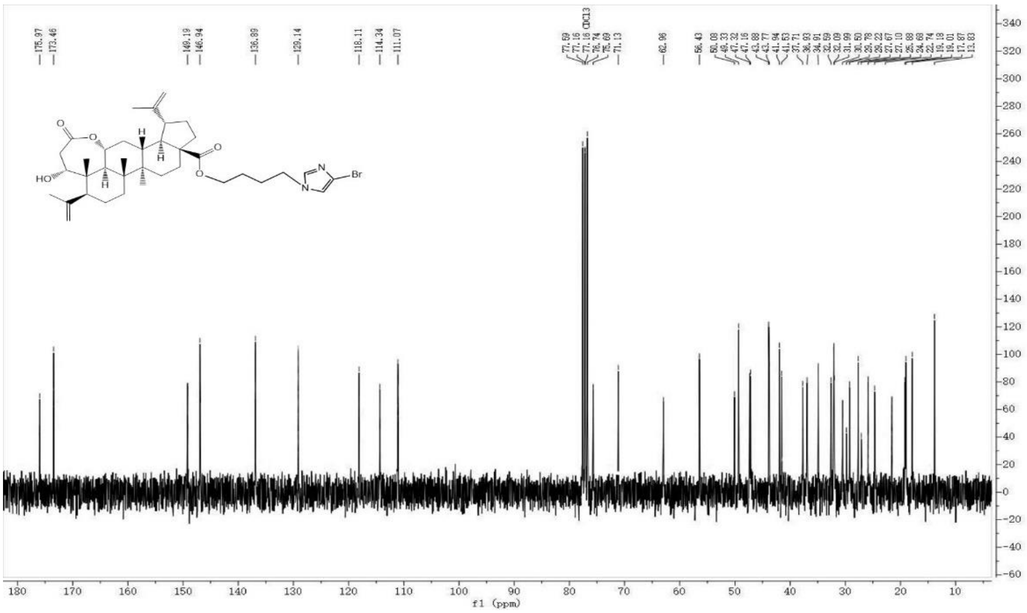

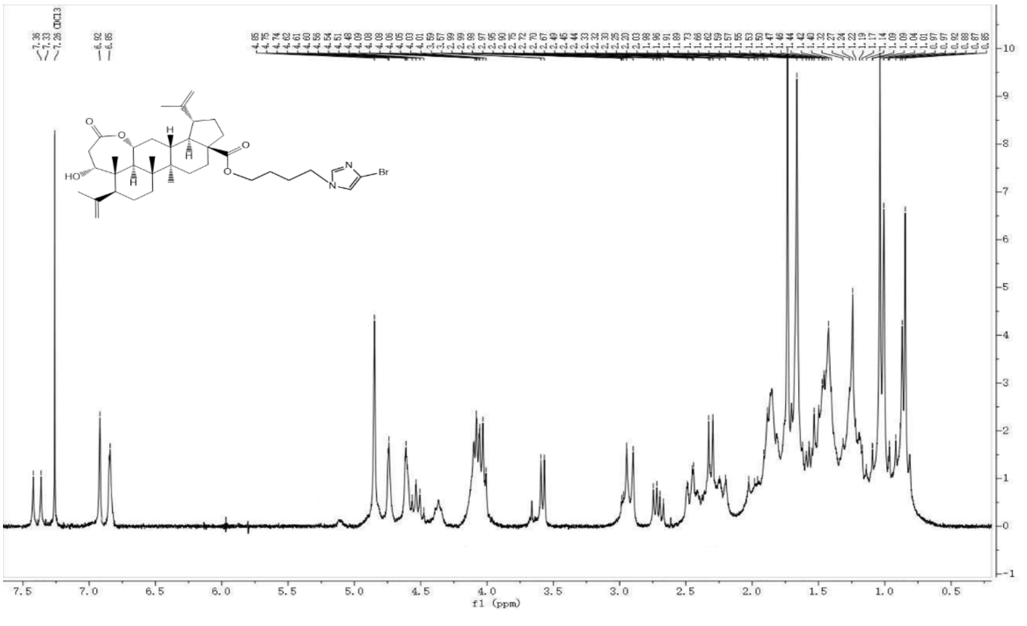

Figures S29. *^{13}C and ^{1}H NMR of compound **I-13**.*

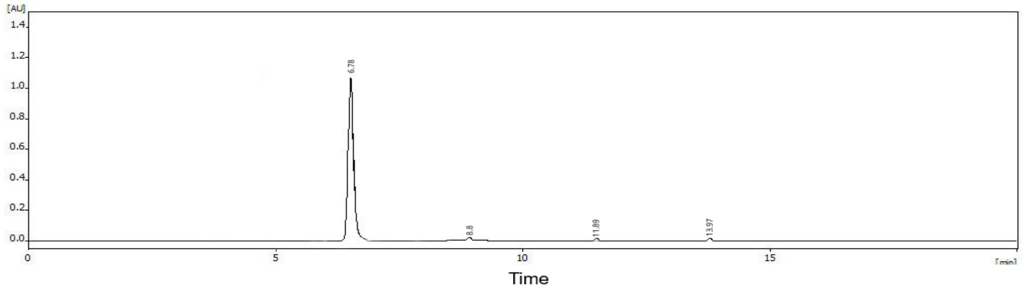

	RT	Area	Area %	Height
1	6.784	244.005	0.7	16.892
2	8.796	34021.318	97.6	2644.189
3	11.893	2209.147	0.6	49.181
4	13.968	383.436	1.1	62.358

Time	A (water,0.5% Phosphoric acid)	B (Acetonitrile)
0	65	35
10	10	90
30	10	90
40	65	35

Figure S30. *HPLC tracing of compound **I-14.***

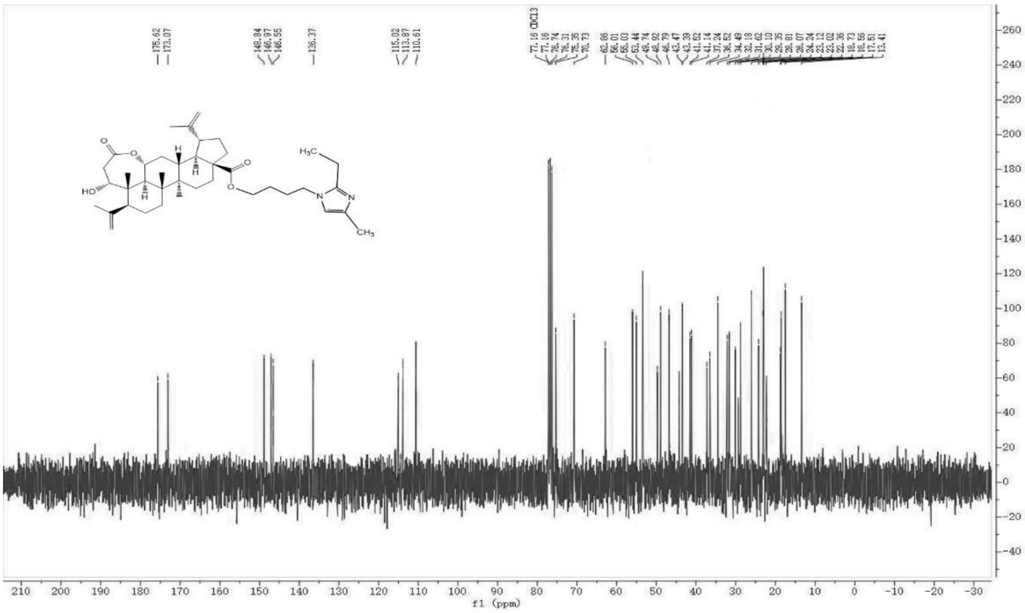

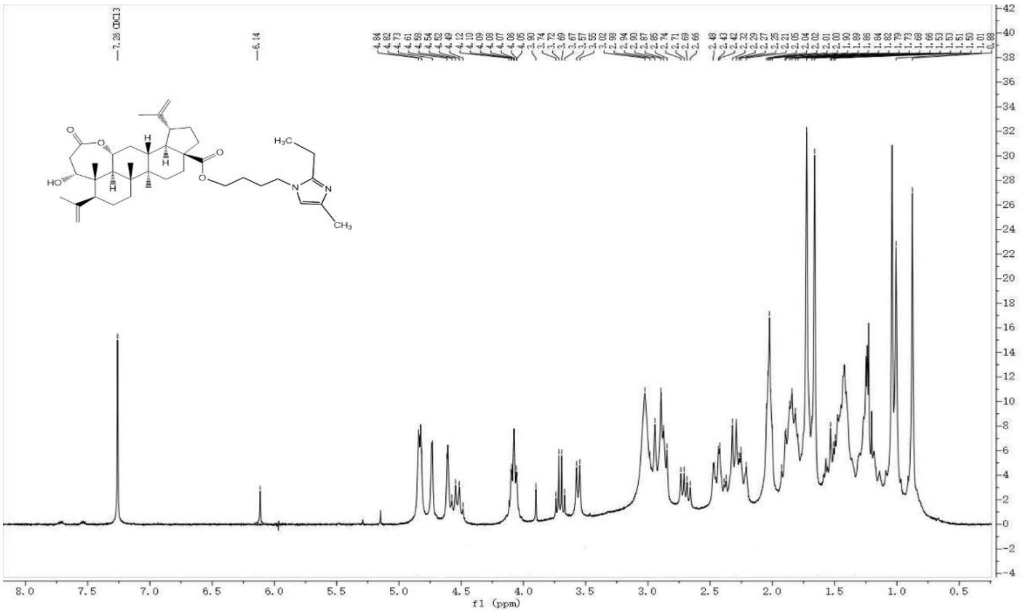

Figures S31. *^{13}C and ^{1}H NMR of compound **I-14**.*

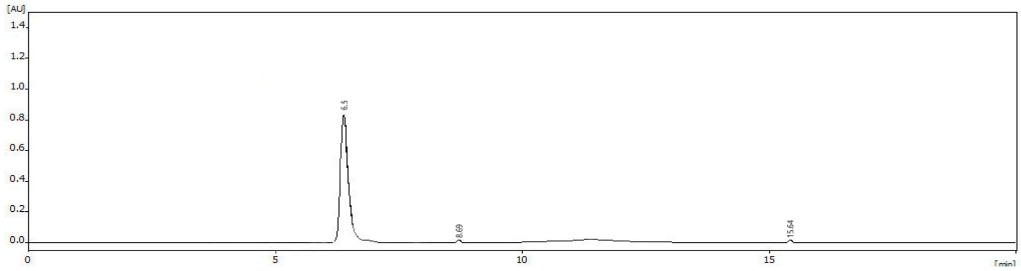

	RT	Area	Area %	Height
1	6.498	390.573	0.7	22.514
2	8.694	54212.259	97.0	1646.572
3	15.639	725.350	1.3	13.854

Time	A (water,0.5% Phosphoric acid)	B (Acetonitrile)
0	65	35
10	10	90
30	10	90
40	65	35

Figure S32. *HPLC tracing of compound **I-15.***

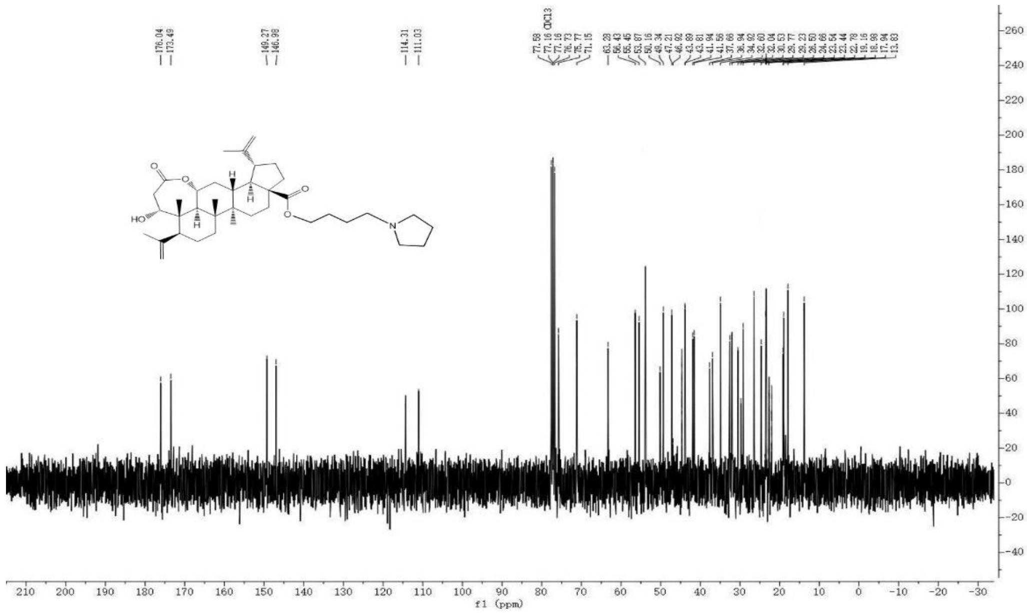

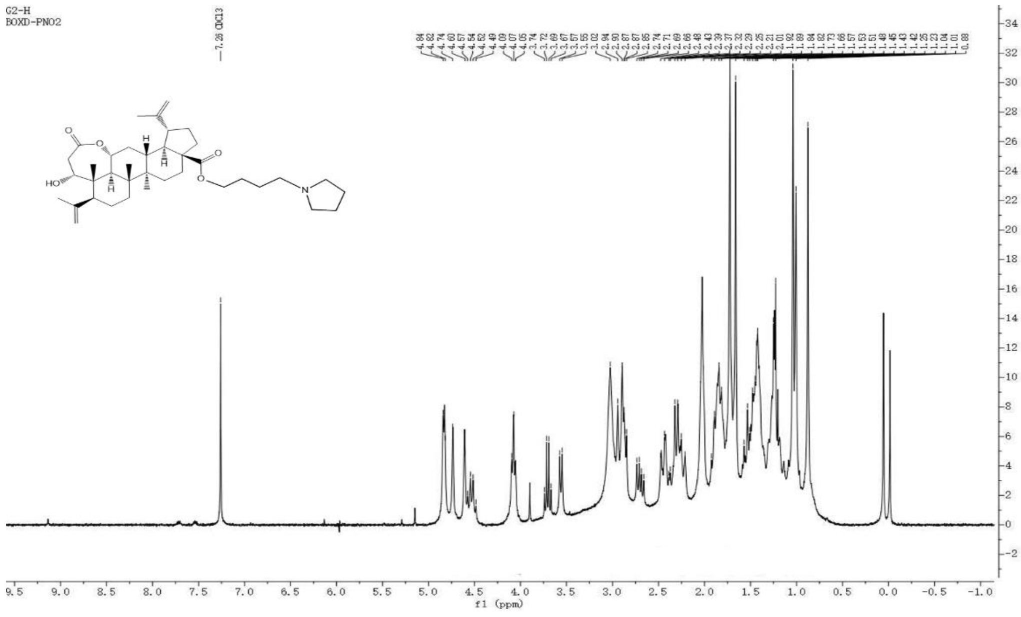

Figures S33. *^{13}C and ^{1}H NMR of compound **I-15**.*

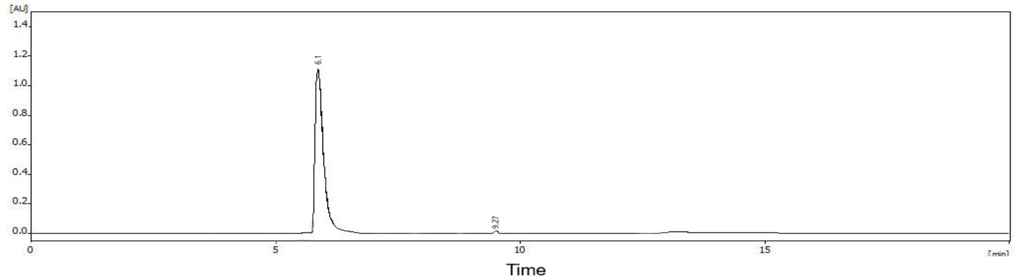

	RT	Area	Area %	Height
1	6.097	36489.192	98.5	1563.150
2	9.267	555.673	1.5	265.15

Time	A (water,0.5% Phosphoric acid)	B (Acetonitrile)
0	65	35
10	10	90
30	10	90
40	65	35

Figure S34. *HPLC tracing of compound* ***I-16.***

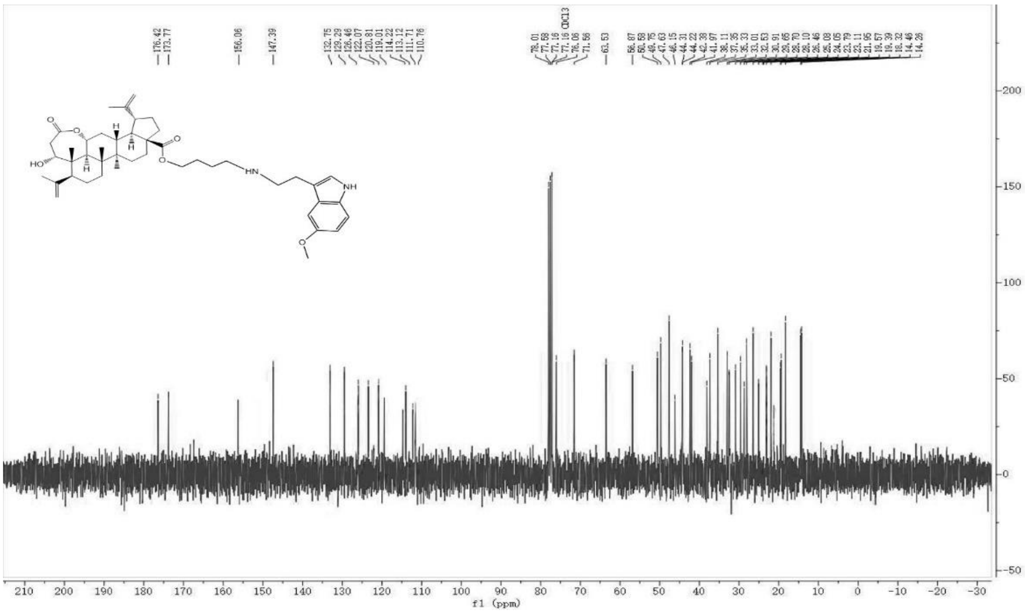

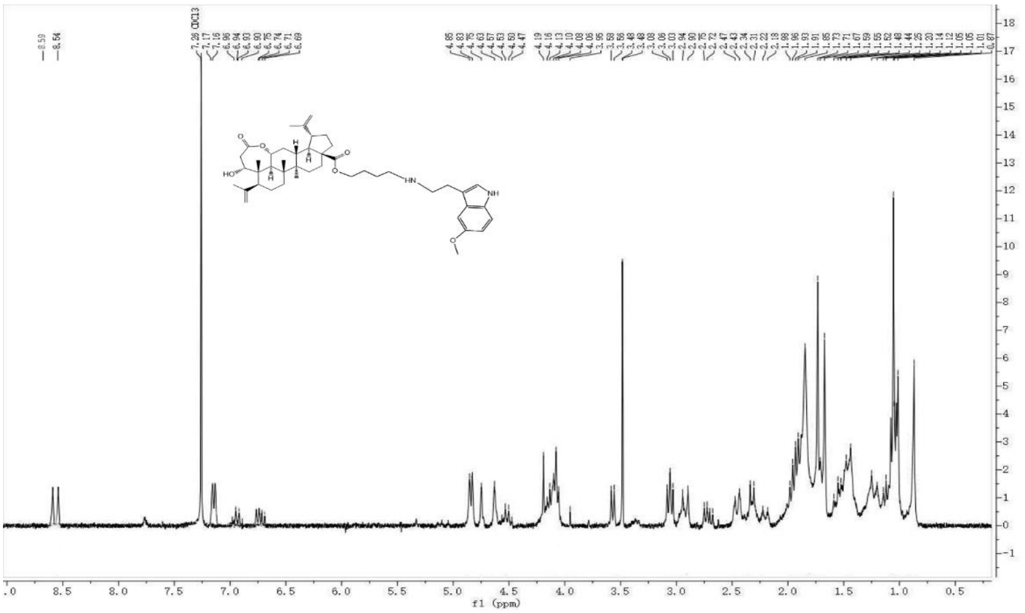

Figures S35. *^{13}C and ^{1}H NMR of compound* ***I-16***.

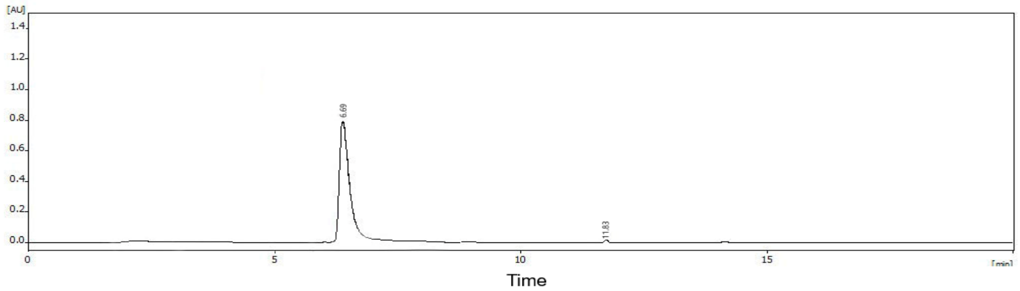

	RT	Area	Area %	Height
1	6.691	34695.153	96.0	2645.138
2	11.831	1445.631	4.0	110.208

Time	A (water,0.5% Phosphoric acid)	B (Acetonitrile)
0	65	35
10	10	90
30	10	90
40	65	35

Figure S36. *HPLC tracing of compound **I-17.***

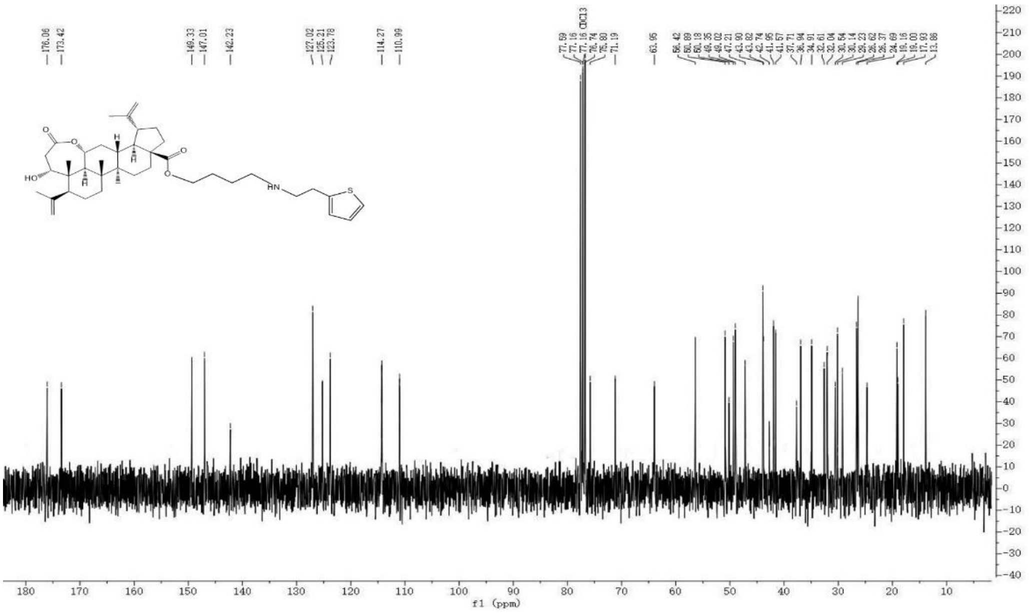

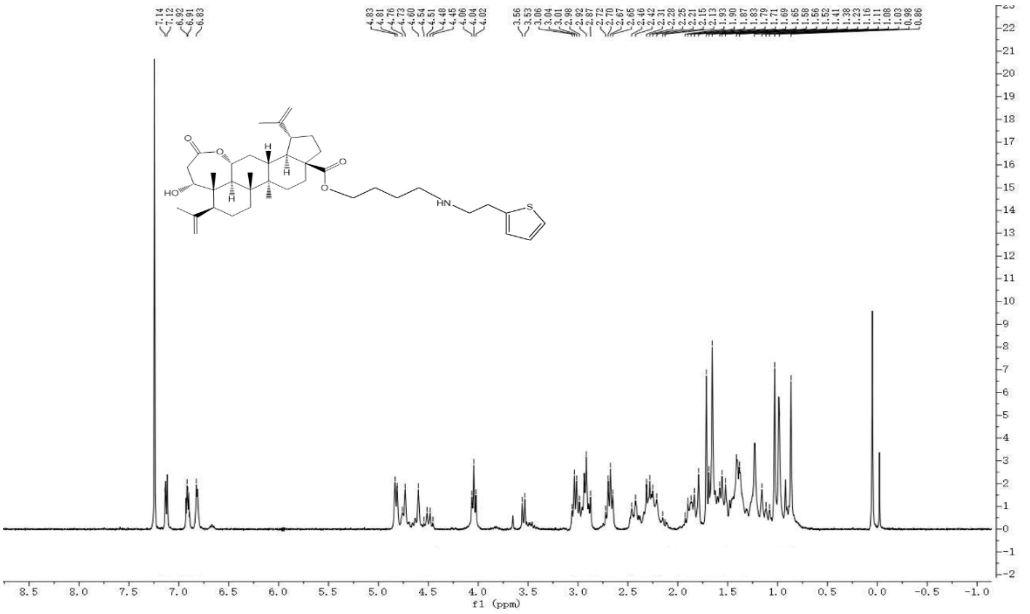

Figures S37. *^{13}C and ^{1}H NMR of compound **I-17**.*

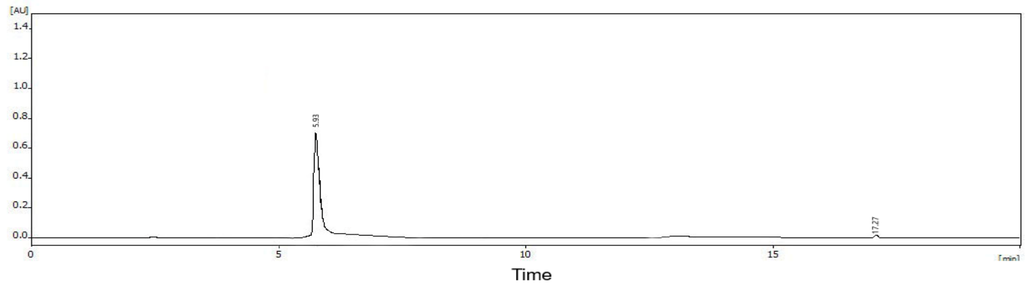

	RT	Area	Area %	Height
1	5.930	39544.268	96.4	2544.285
2	17.269	1476.757	3.6	95.412

Time	A (water,0.5% Phosphoric acid)	B (Acetonitrile)
0	65	35
10	10	90
30	10	90
40	65	35

Figure S38. *HPLC tracing of compound **I-18.***

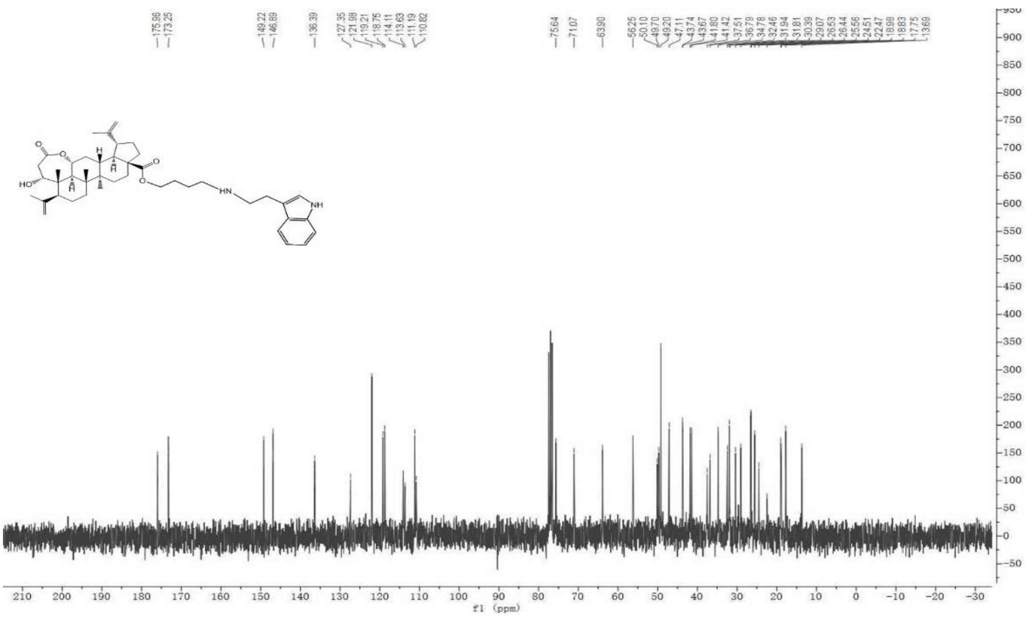

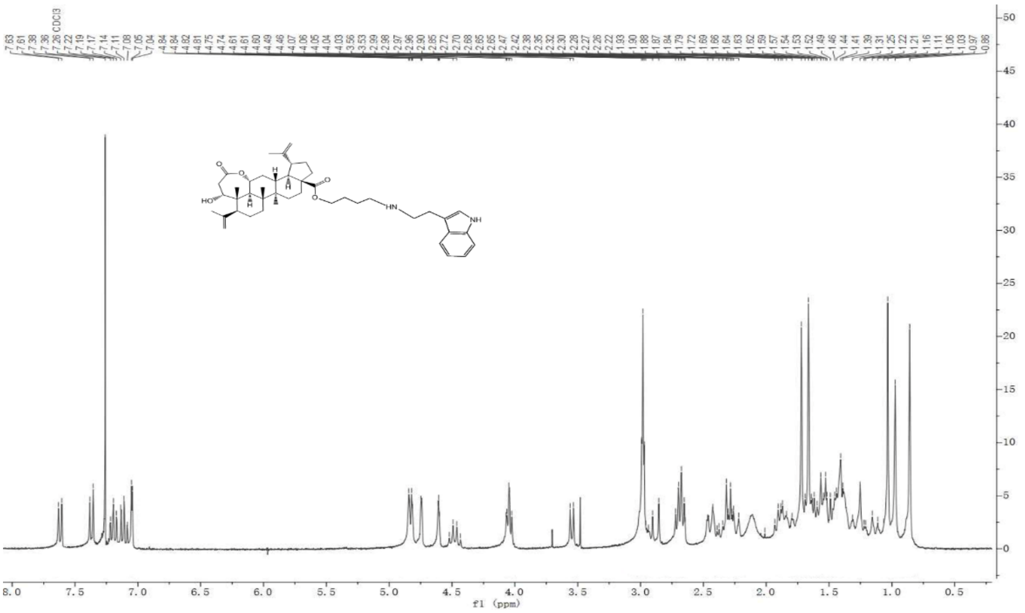

Figures S39. *^{13}C and ^{1}H NMR of compound **I-18**.*

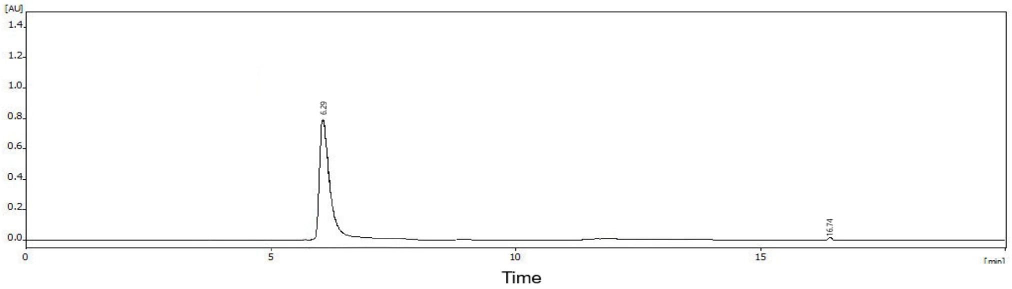

	RT	Area	Area %	Height
1	6.294	36591.150	97.8	1630.154
2	16.743	823.114	2.2	41.32

Time	A (water,0.5% Phosphoric acid)	B (Acetonitrile)
0	65	35
10	10	90
30	10	90
40	65	35

Figure S40. *HPLC tracing of compound **I-19.***

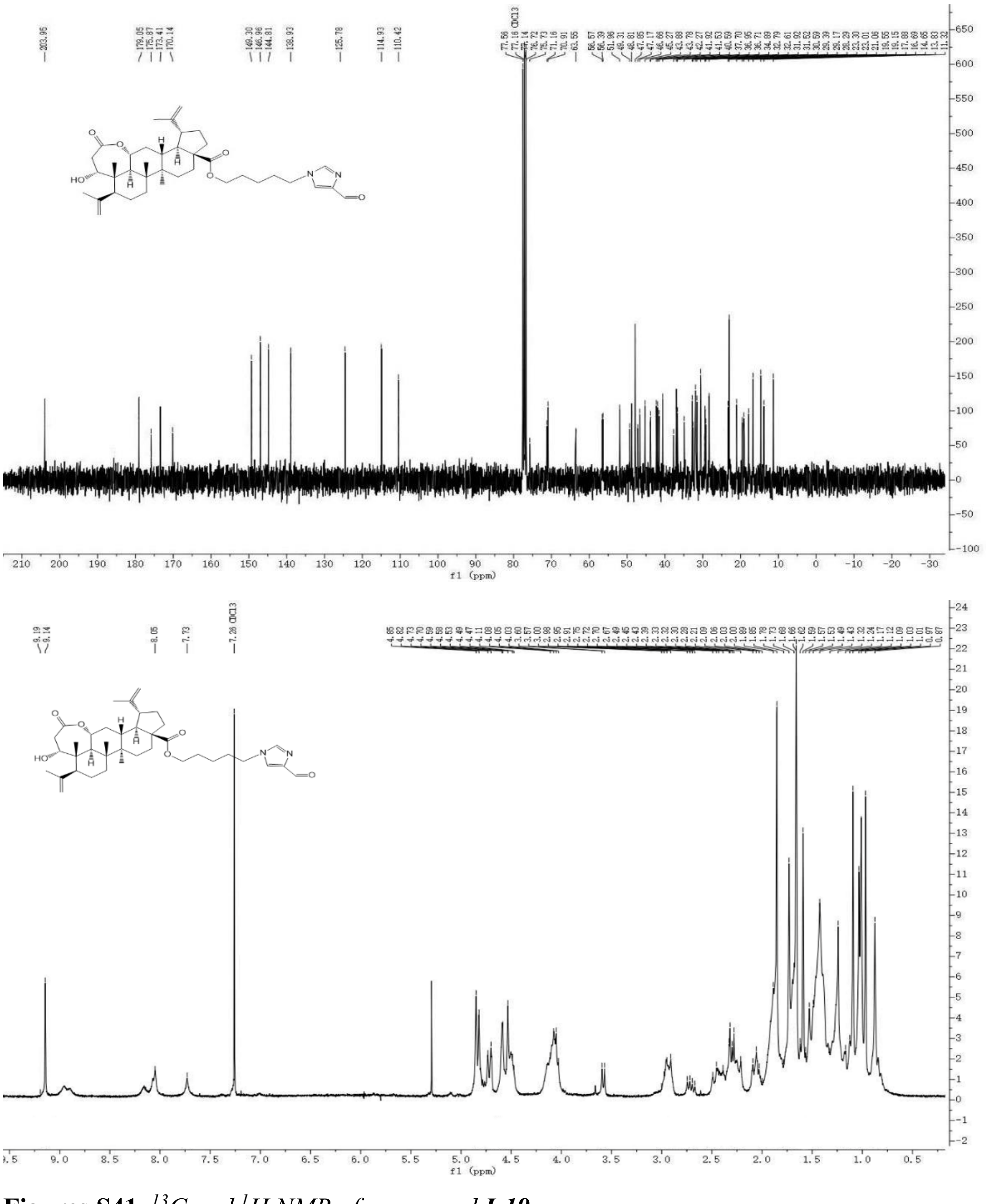

Figures S41. *^{13}C and ^{1}H NMR of compound **I-19**.*

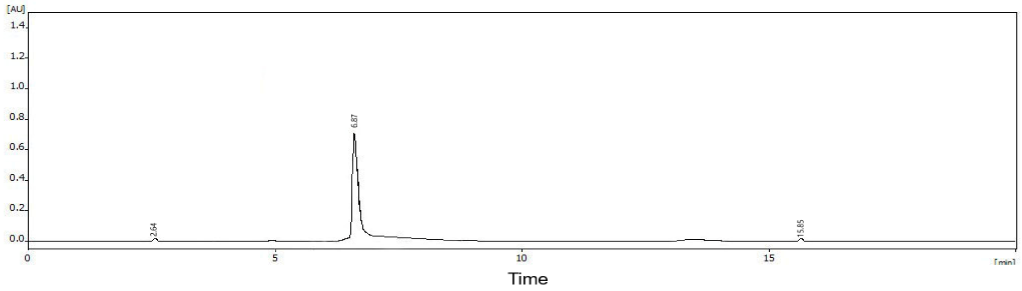

	RT	Area	Area %	Height
1	2.643	580.611	1.3	34.592
2	6.874	43188.492	96.7	1952.264
3	15.846	893.247	2.0	45.268

Time	A (water,0.5% Phosphoric acid)	B (Acetonitrile)
0	65	35
10	10	90
30	10	90
40	65	35

Figure S42. *HPLC tracing of compound **I-20.***

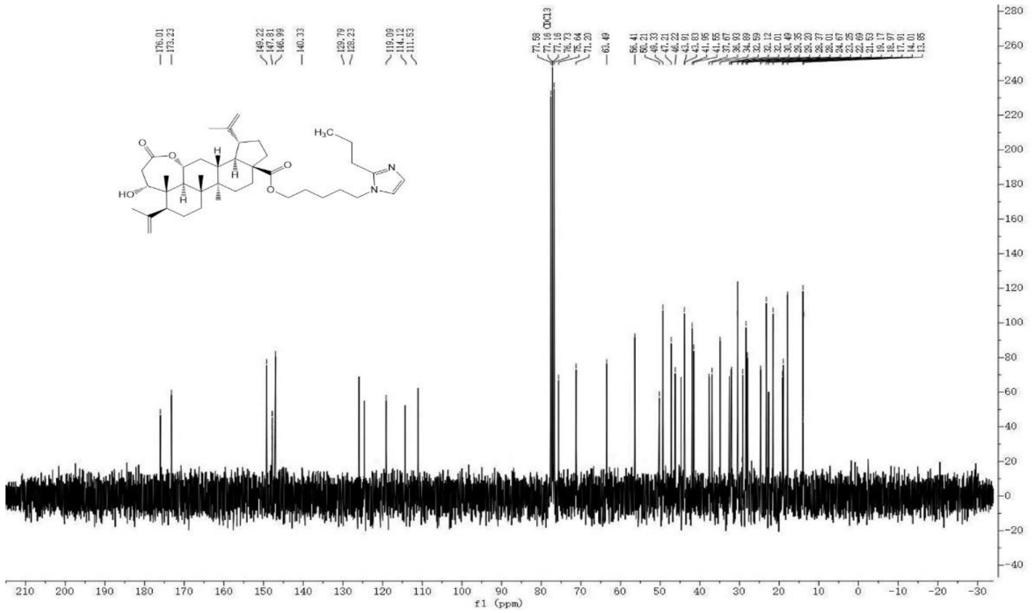

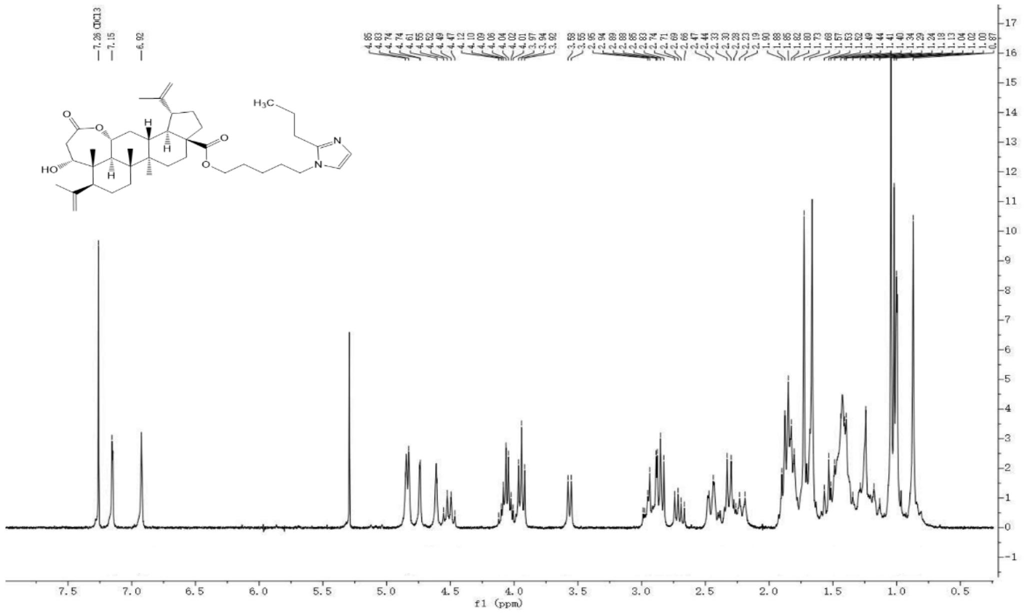

Figures S43. *^{13}C and ^{1}H NMR of compound **I-20**.*

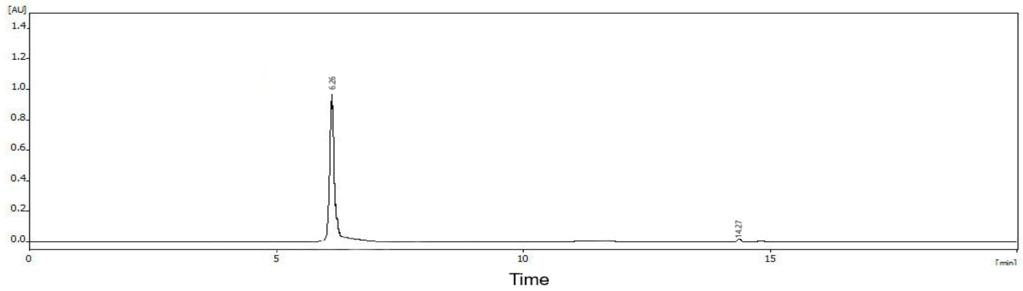

	RT	Area	Area %	Height
1	6.259	46311.292	98.7	2650.188
2	14.268	609.976	1.3	40.258

Time	A (water,0.5% Phosphoric acid)	B (Acetonitrile)
0	65	35
10	10	90
30	10	90
40	65	35

Figure S44. *HPLC tracing of compound **I-21.***

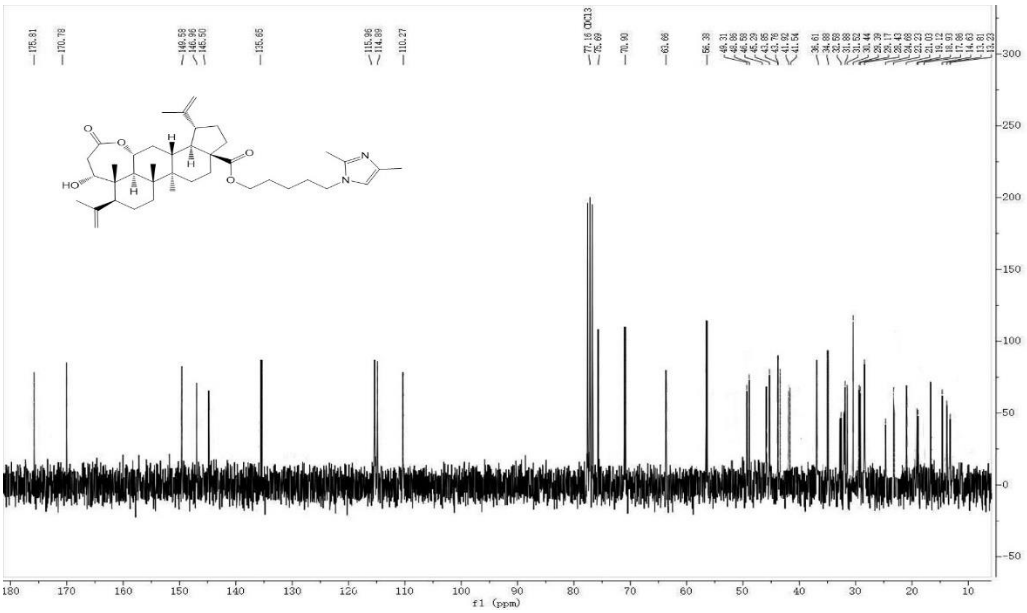

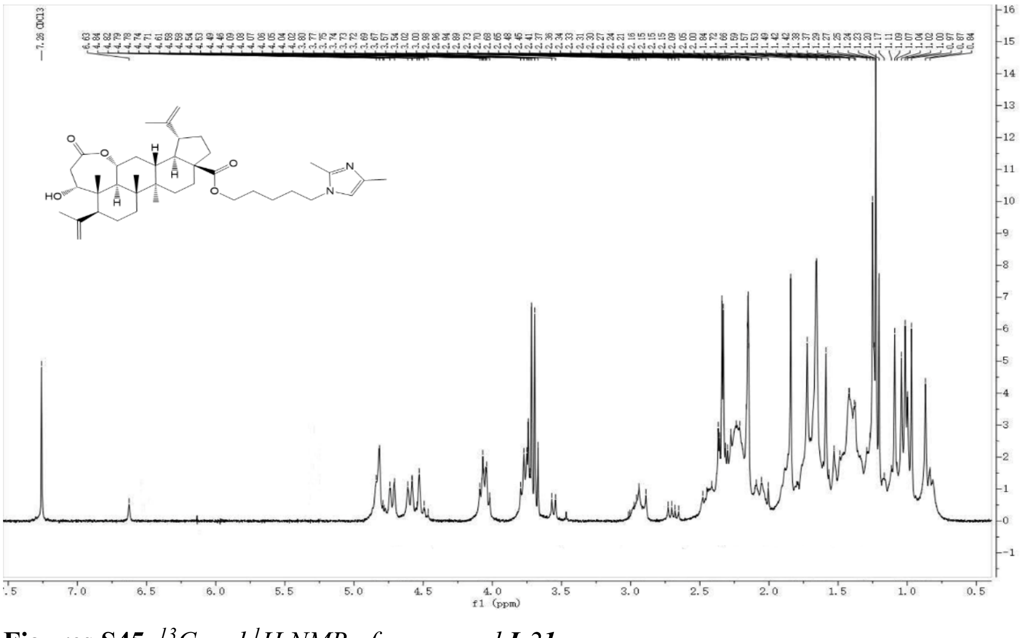

Figures S45. *^{13}C and ^{1}H NMR of compound **I-21**.*

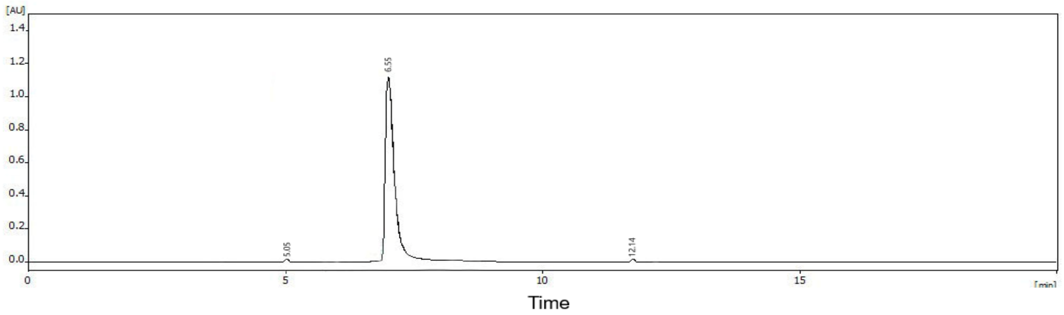

	RT	Area	Area %	Height
1	5.053	725.359	2.6	64.182
2	6.549	26587.185	95.3	2016.150
3	12.135	585.867	2.1	52.354

Time	A (water,0.5% Phosphoric acid)	B (Acetonitrile)
0	65	35
10	10	90
30	10	90
40	65	35

Figure S46. *HPLC tracing of compound* ***I-22***.

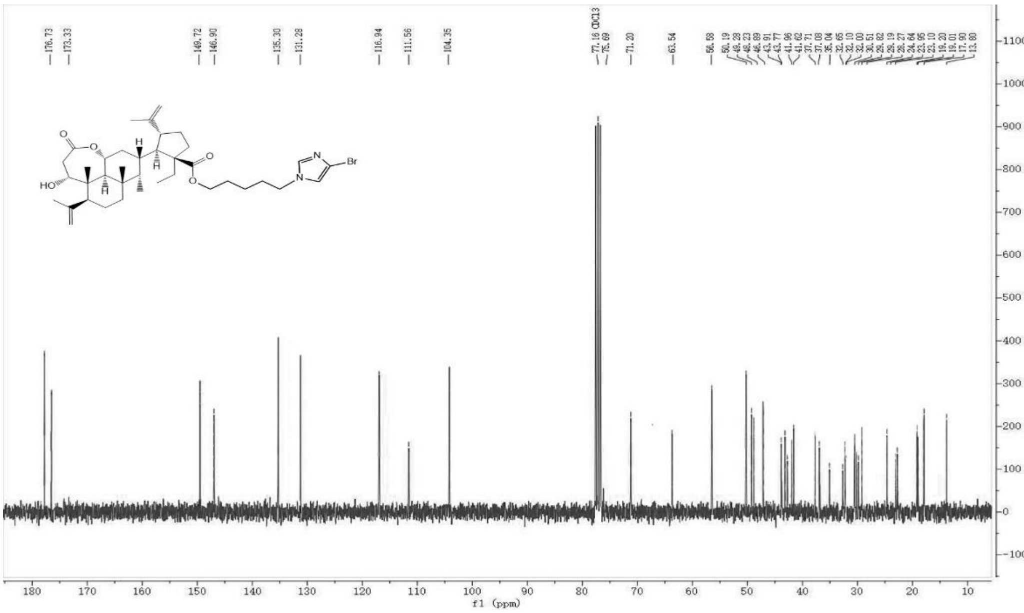

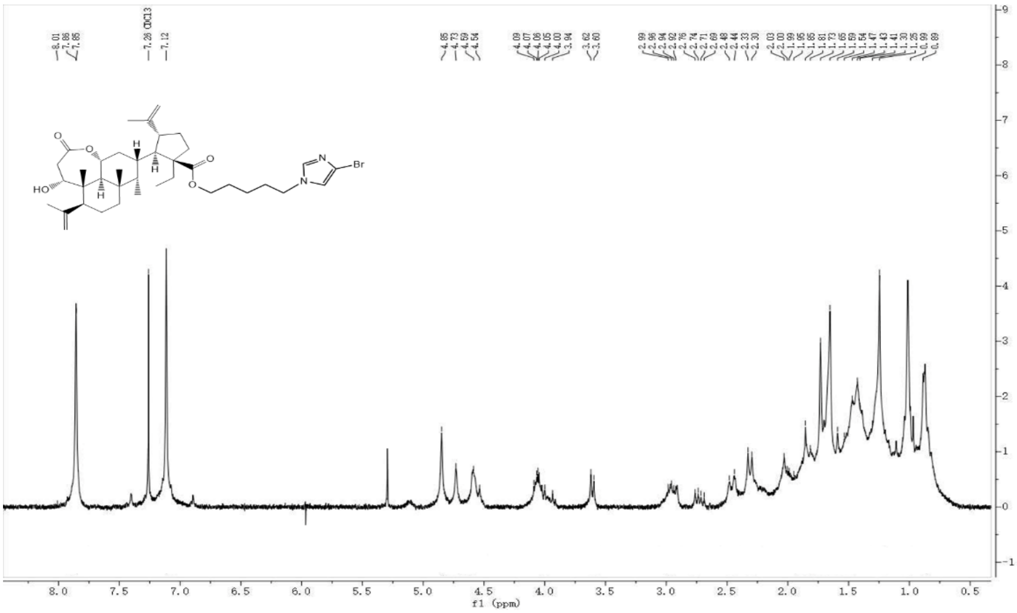

Figures S47. *^{13}C and ^{1}H NMR of compound* ***I-22***.

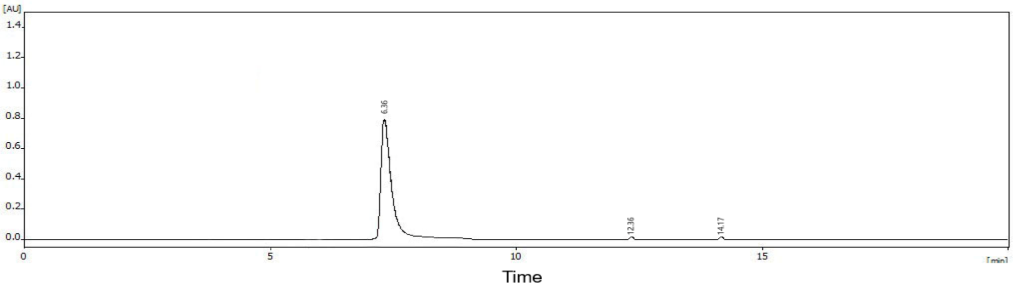

	RT	Area	Area %	Height
1	6.359	30284.820	97.5	2310.252
2	12.357	496.9882	1.6	37.925
3	14.167	279.552	0.9	25.325

Time	A (water,0.5% Phosphoric acid)	B (Acetonitrile)
0	65	35
10	10	90
30	10	90
40	65	35

Figure S48. *HPLC tracing of compound **I-23.***

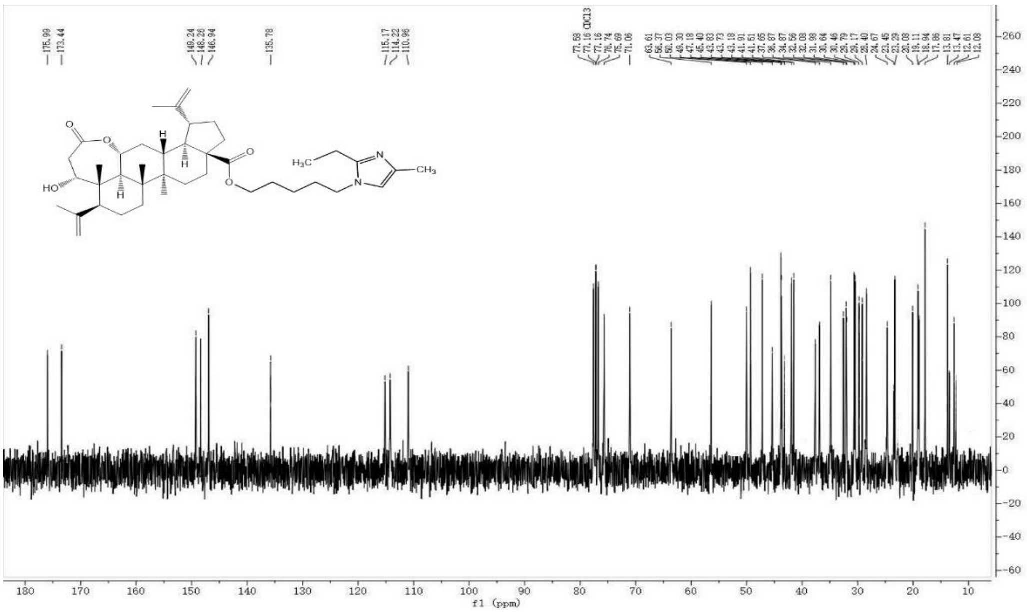

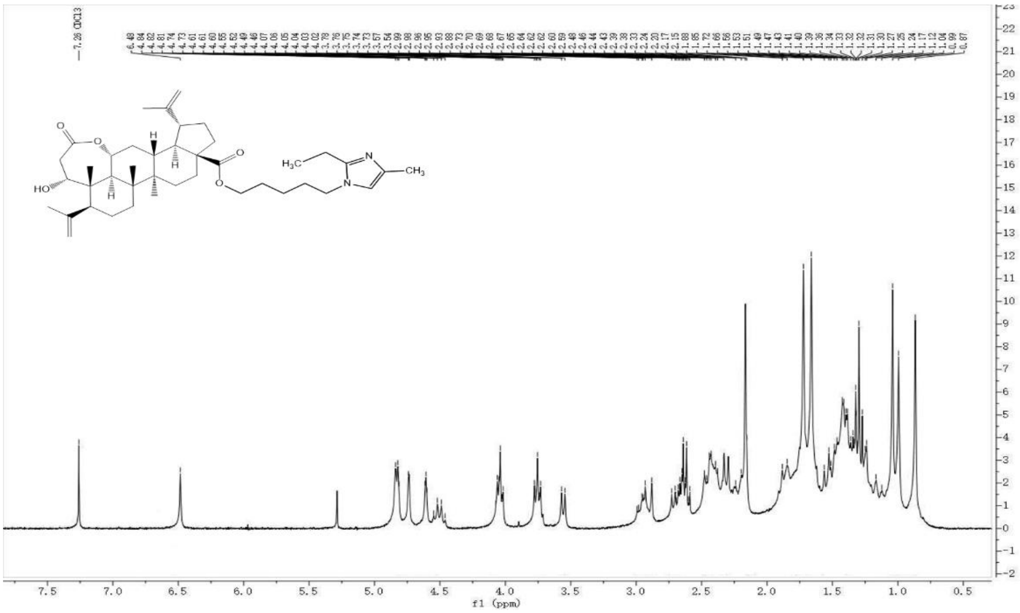

Figures S49. *^{13}C and ^{1}H NMR of compound **I-23**.*

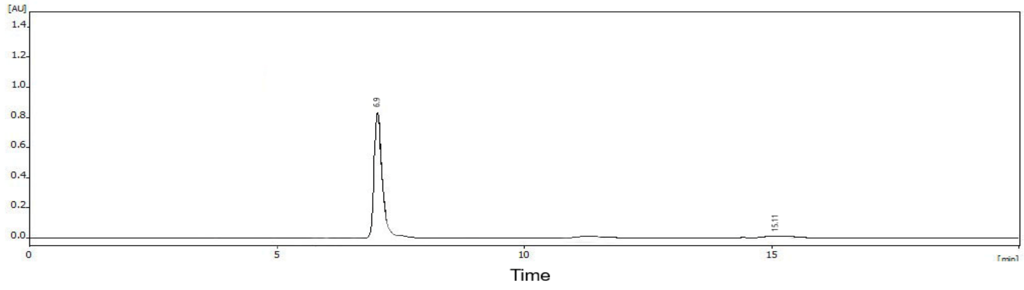

	RT	Area	Area %	Height
1	6.895	36849.185	96.4	1058.180
2	15.109	1376.110	3.6	40.675

Time	A (water,0.5% Phosphoric acid)	B (Acetonitrile)
0	65	35
10	10	90
30	10	90
40	65	35

Figure S50. *HPLC tracing of compound **I-24.***

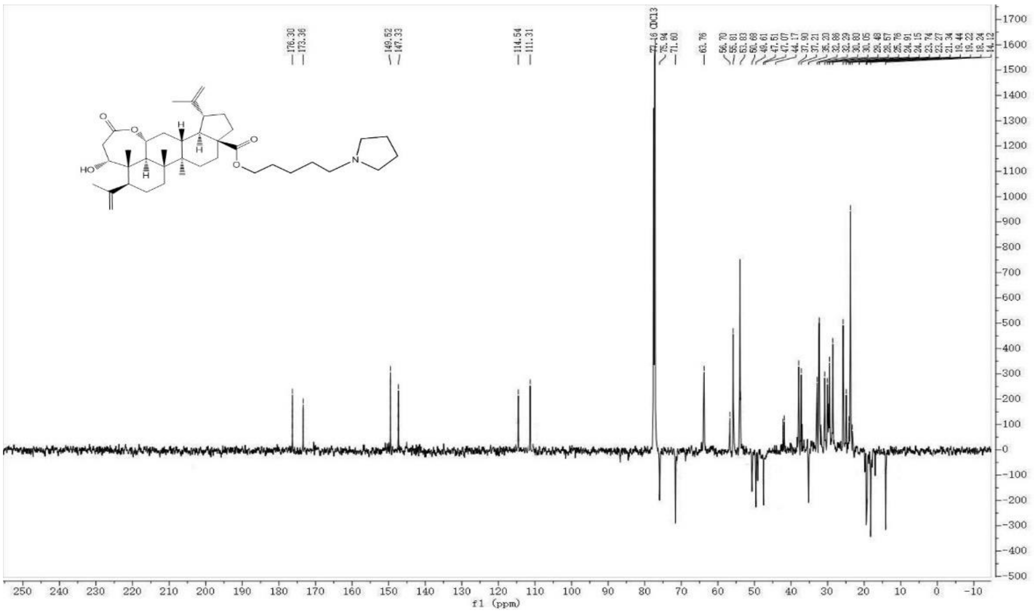

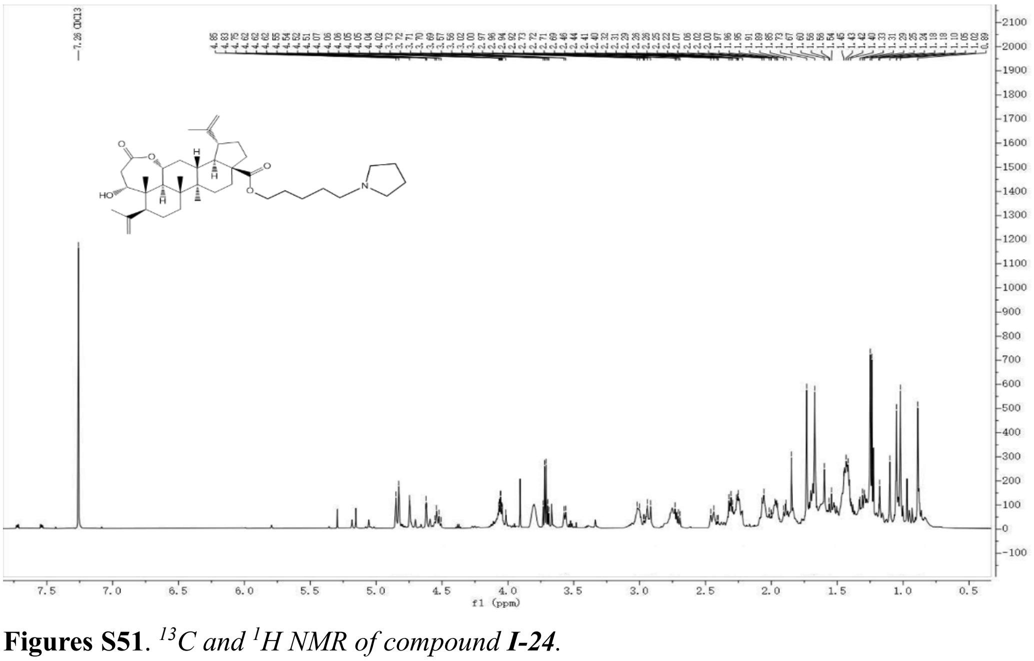

Figures S51. *^{13}C and ^{1}H NMR of compound **I-24**.*

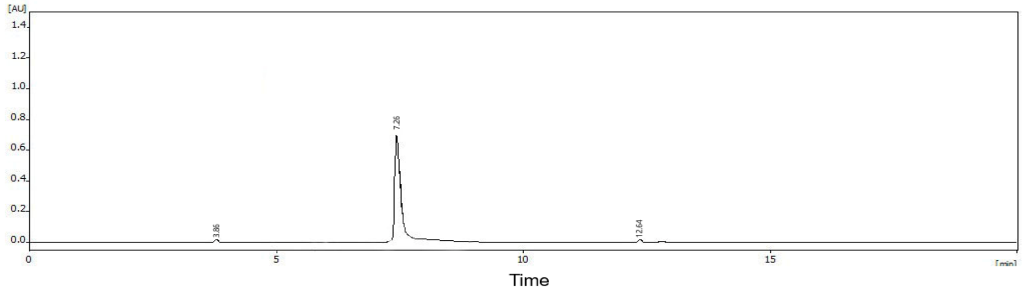

	RT	Area	Area %	Height
1	3.863	36412.532	97.5	1583.850
2	7.262	560.193	1.5	24.307
3	12.637	373.462	1.0	16.205

Time	A (water,0.5% Phosphoric acid)	B (Acetonitrile)
0	65	35
10	10	90
30	10	90
40	65	35

Figure S52. *HPLC tracing of compound **I-25.***

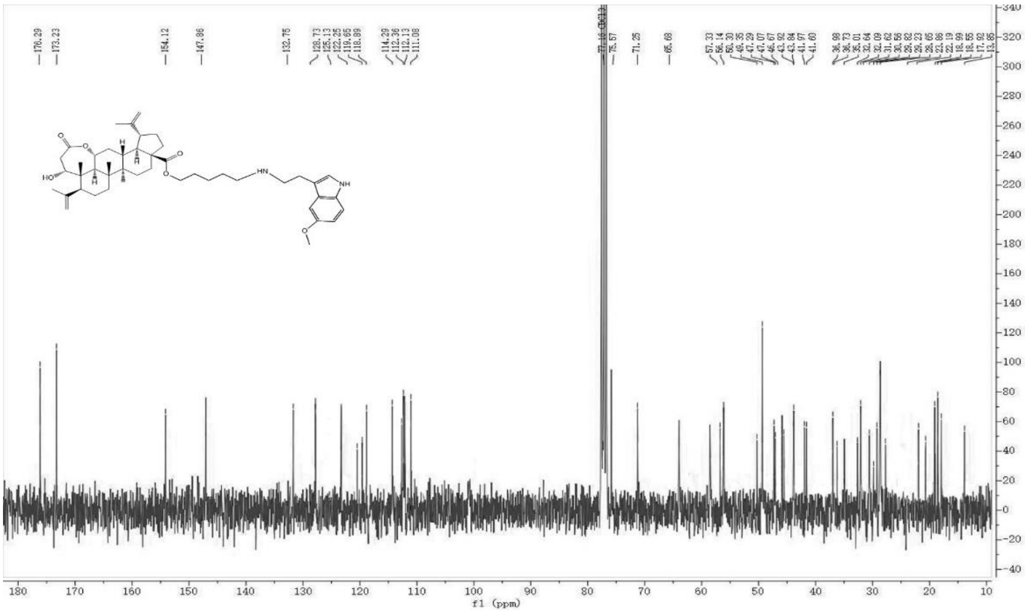

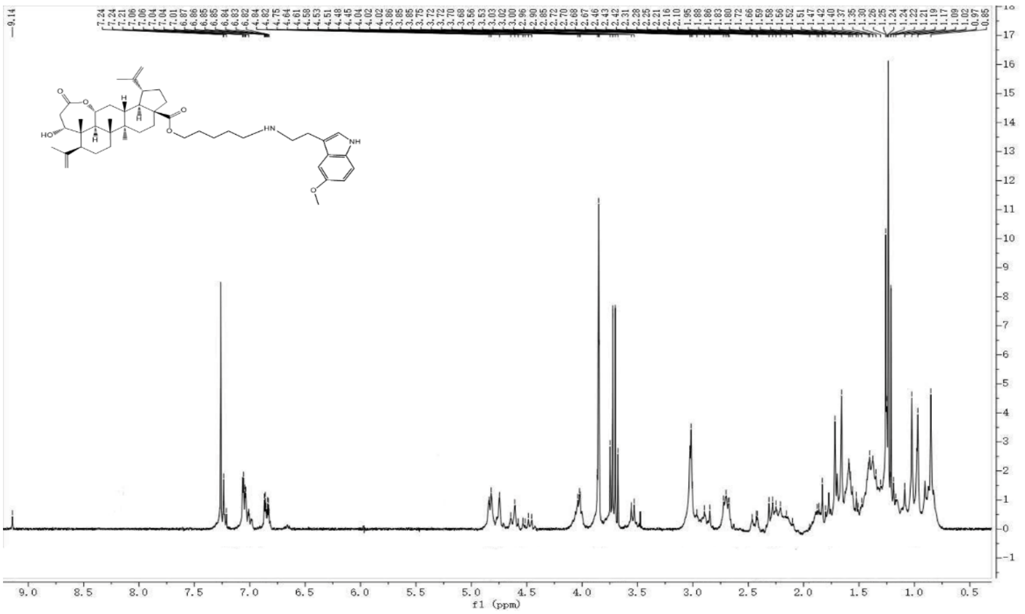

Figures S53. *^{13}C and ^{1}H NMR of compound **I-25**.*

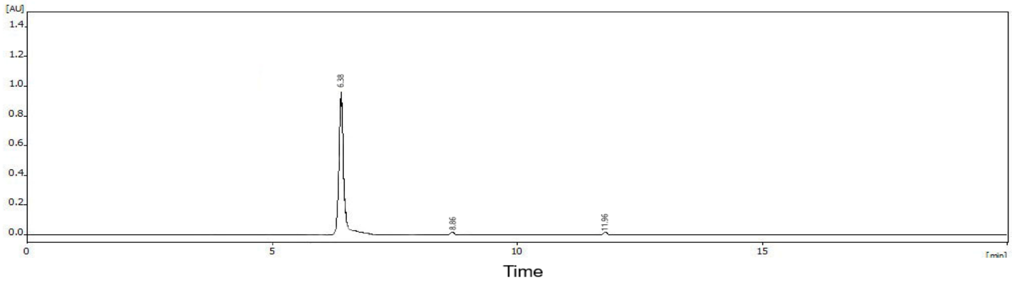

	RT	Area	Area %	Height
1	6.383	46520.155	98.1	3025.312
2	8.863	758.739	1.6	231.155
3	11.963	142.263	0.3	11.655

Time	A (water,0.5% Phosphoric acid)	B (Acetonitrile)
0	65	35
10	10	90
30	10	90
40	65	35

Figure S54. *HPLC tracing of compound **I-26.***

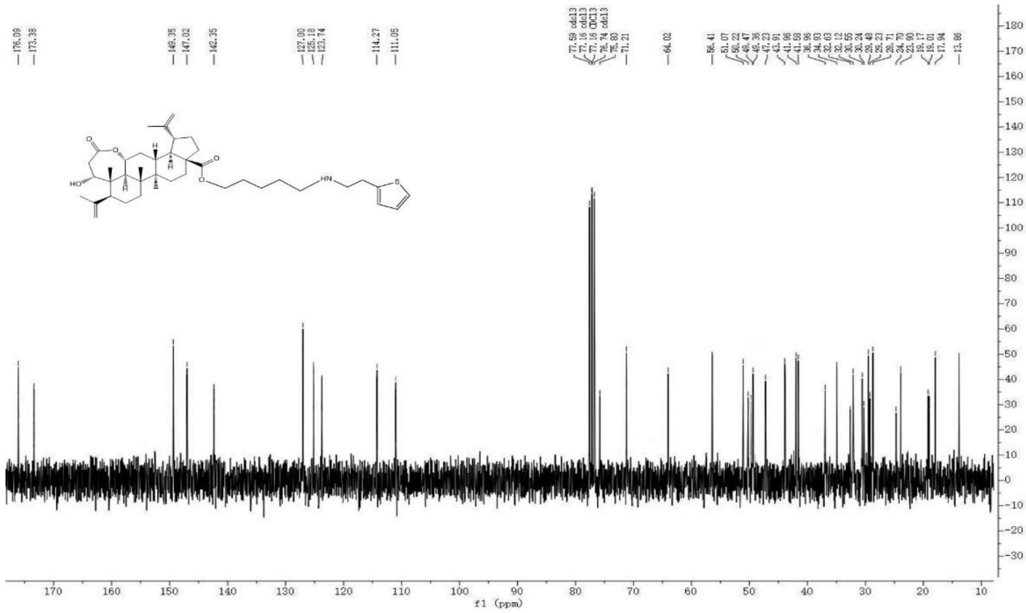

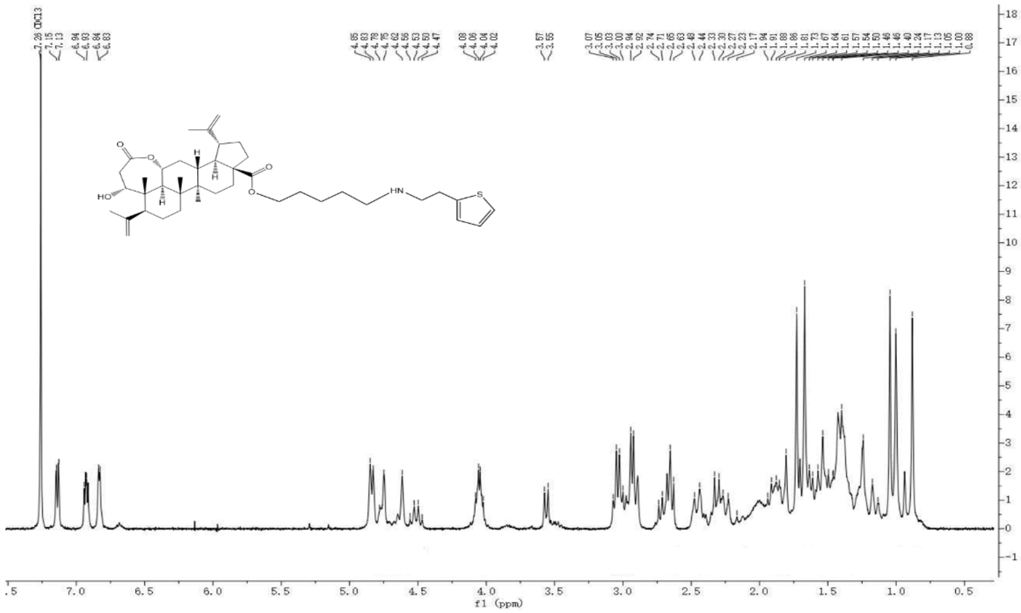

Figures S55. *^{13}C and ^{1}H NMR of compound **I-26**.*

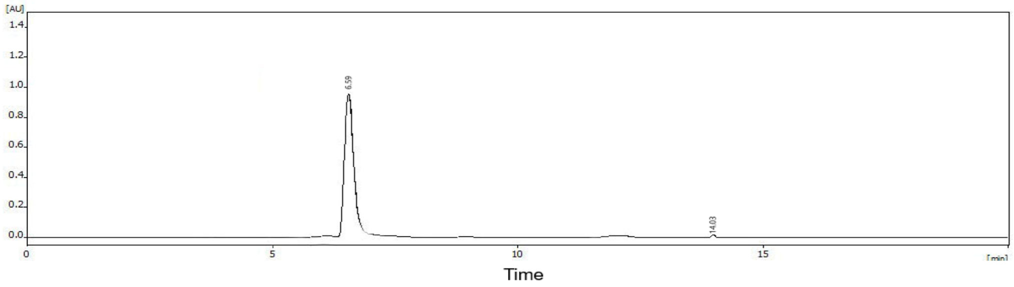

	RT	Area	Area %	Height
1	6.594	46201.352	96.3	1265.285
2	14.034	1775.130	3.7	354.058

Time	A (water,0.5% Phosphoric acid)	B (Acetonitrile)
0	65	35
10	10	90
30	10	90
40	65	35

Figure S56. *HPLC tracing of compound **I-27**.*

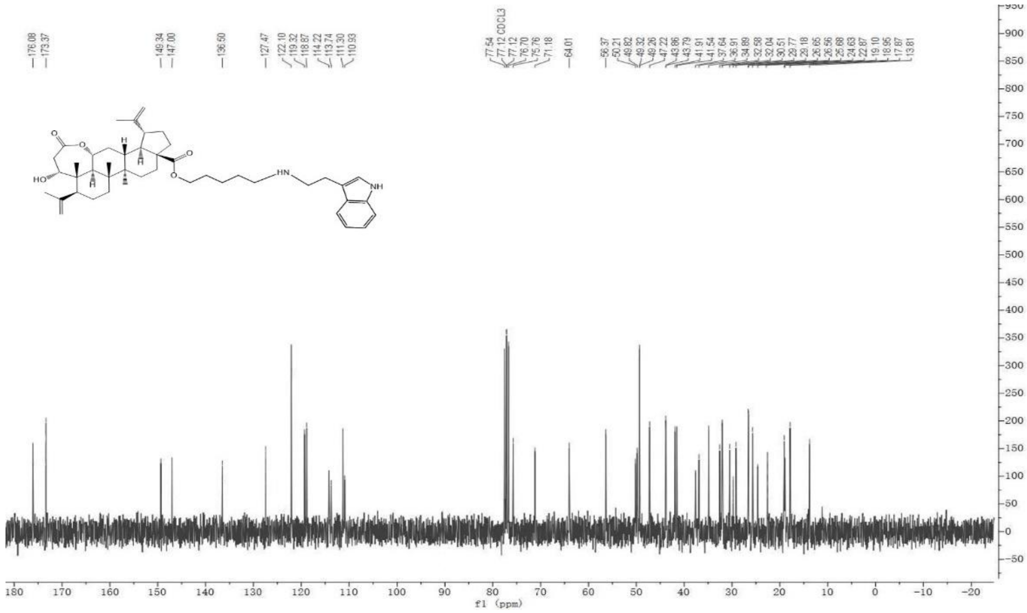

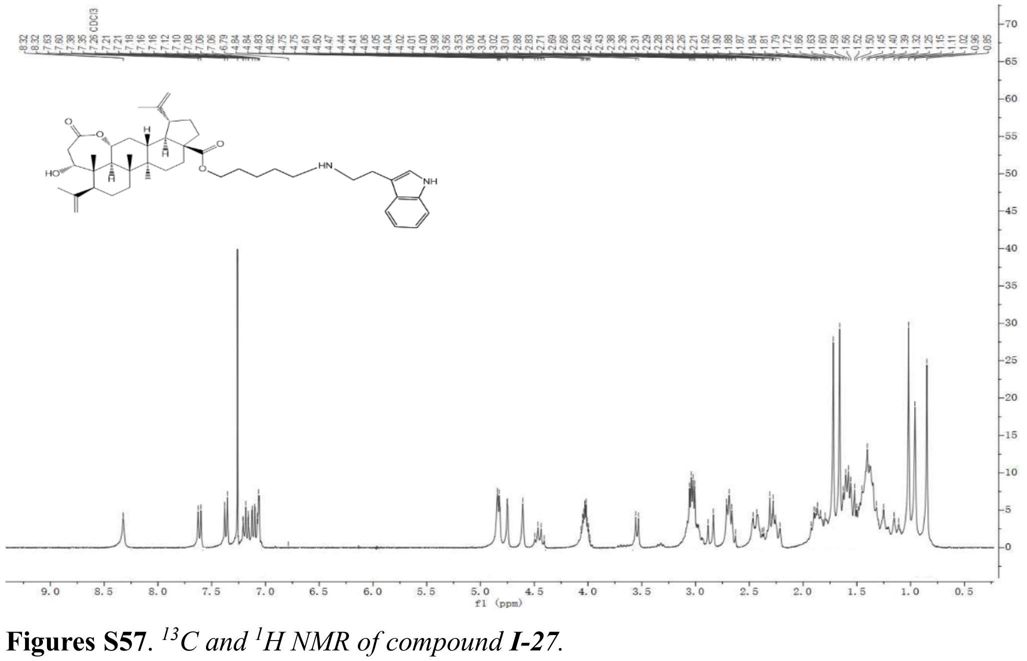

Figures S57. *^{13}C and ^{1}H NMR of compound **I-27**.*

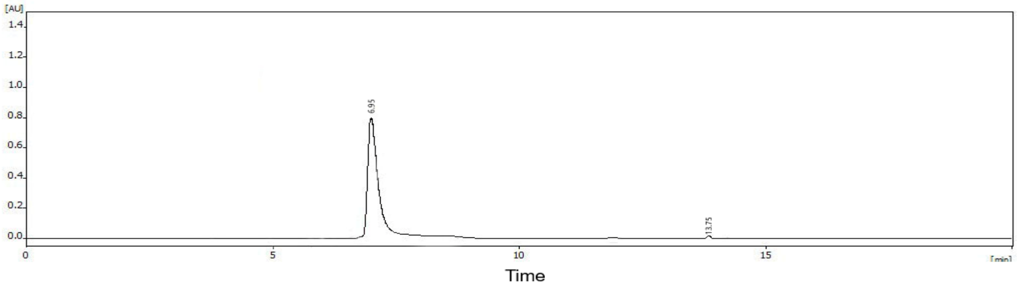

	RT	Area	Area %	Height
1	6.947	32511.651	98.2	2532.255
2	13.754	595.937	1.8	256.264

Time	A (water,0.5% Phosphoric acid)	B (Acetonitrile)
0	65	35
10	10	90
30	10	90
40	65	35

Figure S58. *HPLC tracing of compound **I-28**.*

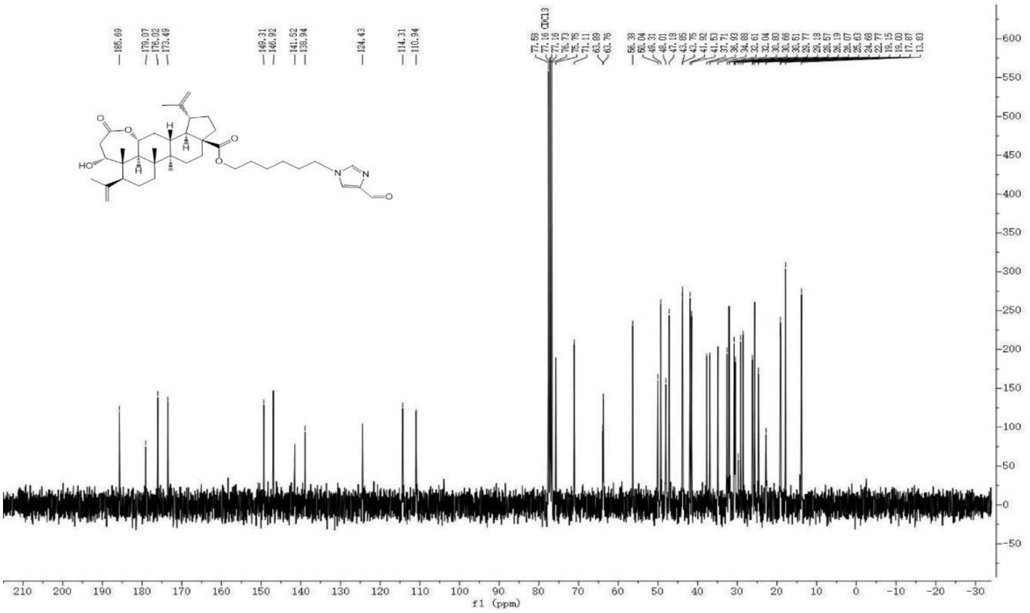

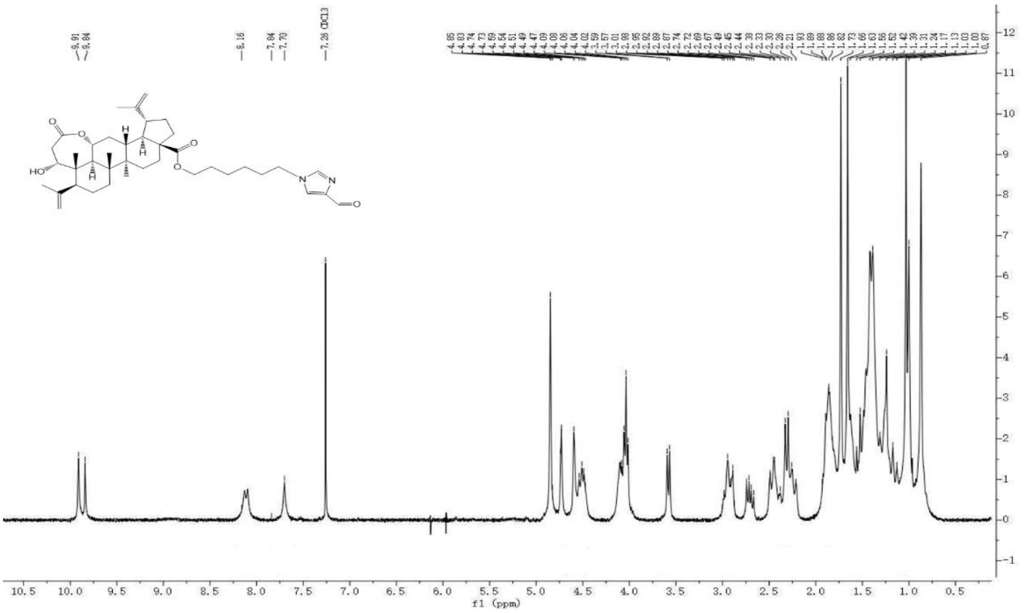

Figures S59. *^{13}C and ^{1}H NMR of compound **I-28**.*

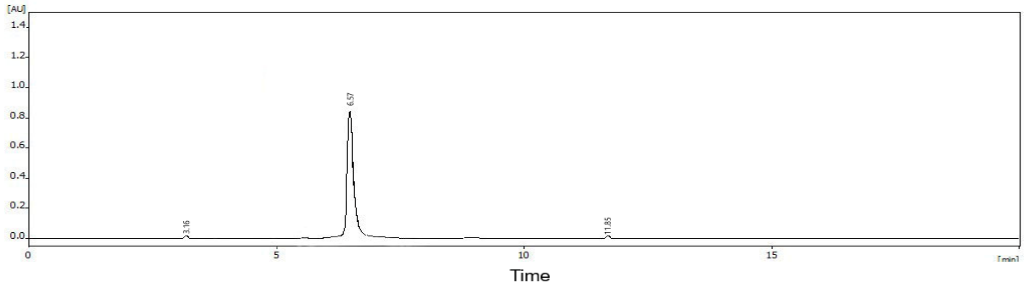

	RT	Area	Area %	Height
1	3.156	725.340	2.2	235.648
2	6.569	31585.255	95.8	1025.285
3	11.847	659.399	2.0	246.891

Time	A (water,0.5% Phosphoric acid)	B (Acetonitrile)
0	65	35
10	10	90
30	10	90
40	65	35

Figure S60. *HPLC tracing of compound **I-29**.*

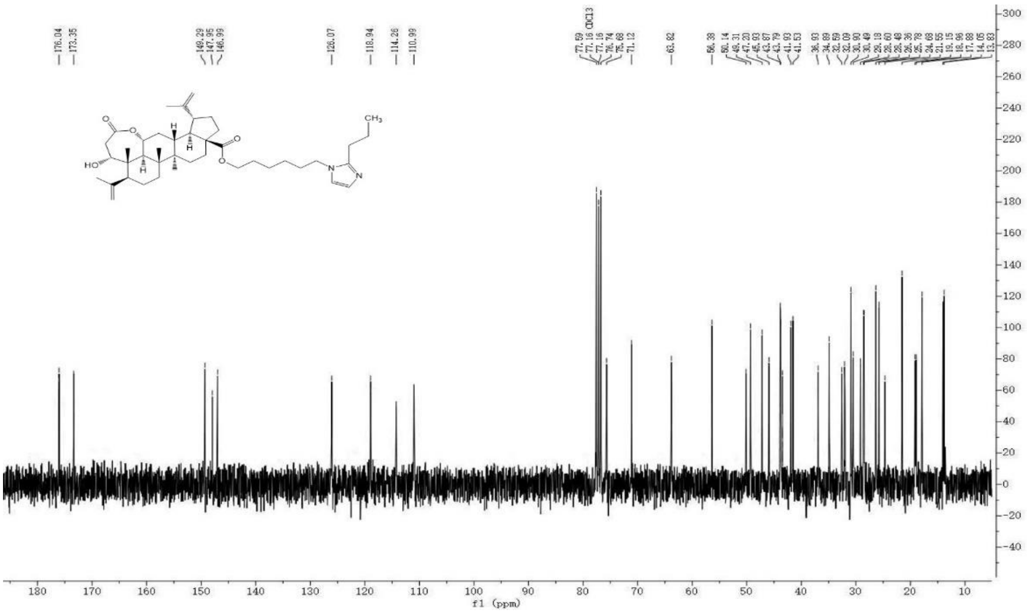

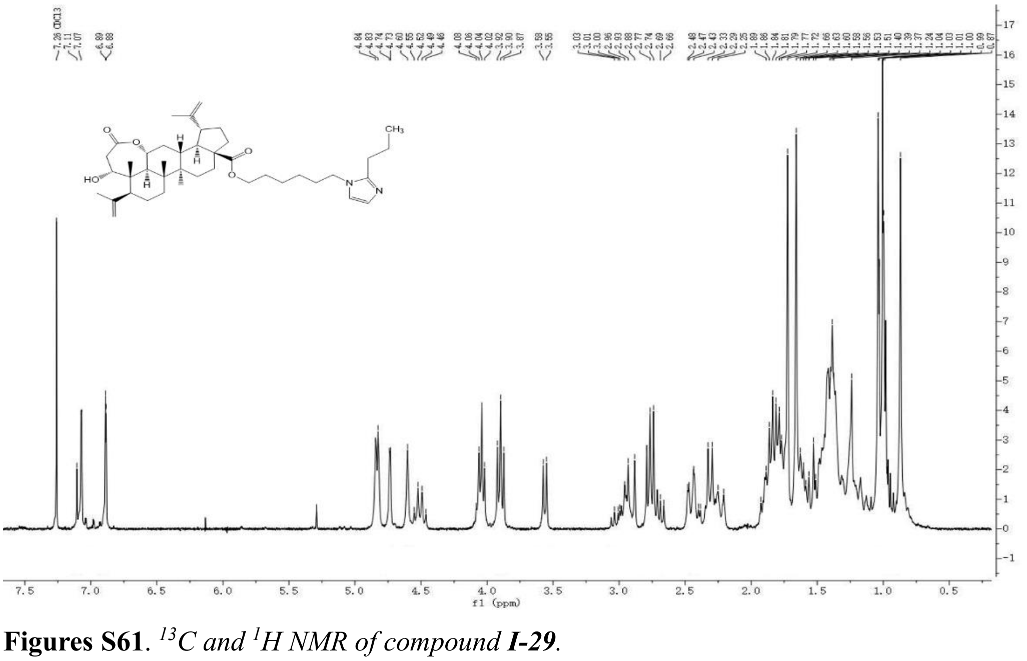

Figures S61. *^{13}C and ^{1}H NMR of compound **I-29**.*

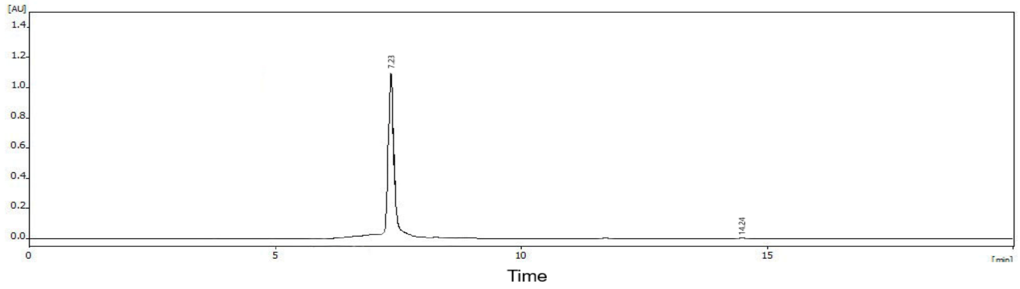

	RT	Area	Area %	Height
1	7.234	30258.864	98.1	1632.259
2	14.237	586.053	1.9	63.521

Time	A (water,0.5% Phosphoric acid)	B (Acetonitrile)
0	65	35
10	10	90
30	10	90
40	65	35

Figure S62. *HPLC tracing of compound **I-30**.*

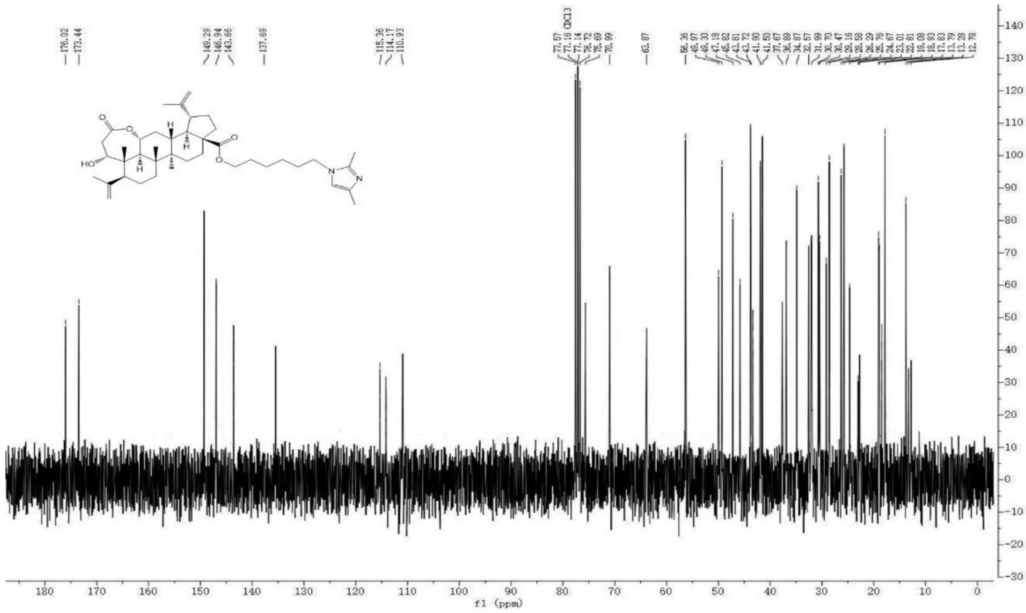

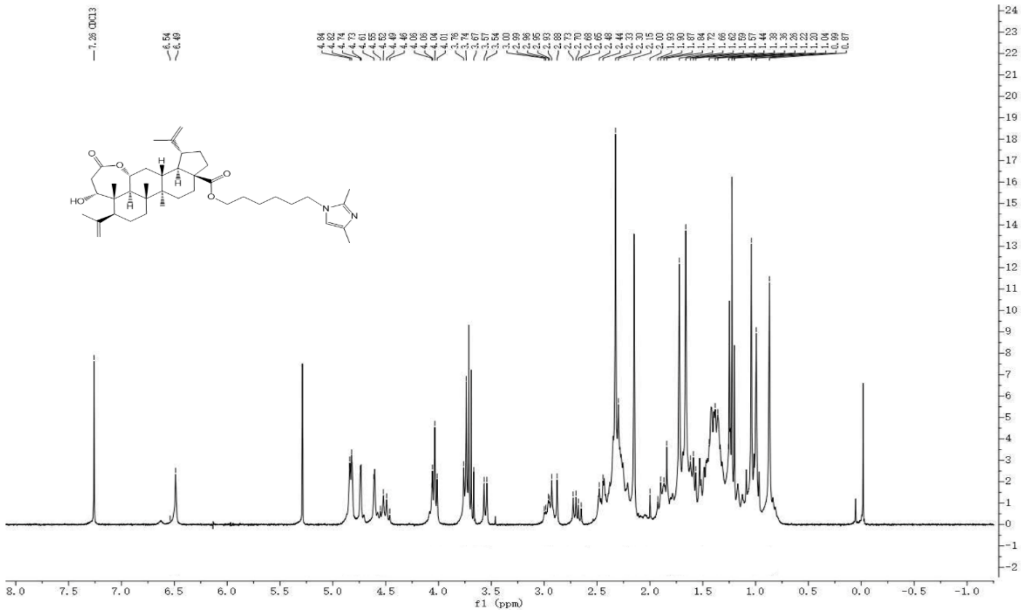

Figures S63. *^{13}C and ^{1}H NMR of compound **I-30**.*

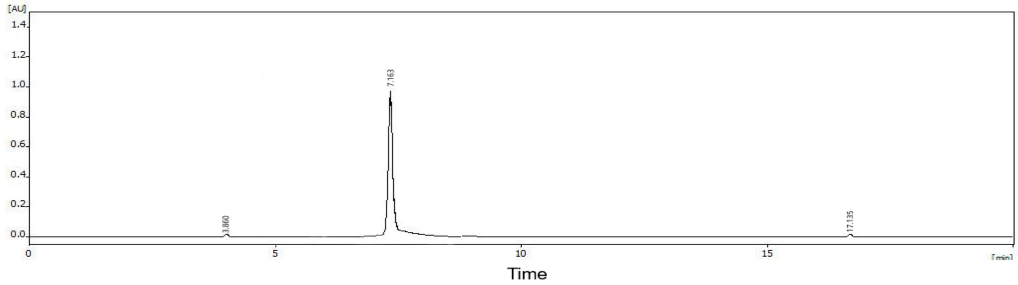

	RT	Area	Area %	Height
1	3.860	930.079	2.6	325.150
2	7.163	34520.250	96.5	2654.052
3	17.135	321.950	0.9	52.632

Time	A (water,0.5% Phosphoric acid)	B (Acetonitrile)
0	65	35
10	10	90
30	10	90
40	65	35

Figure S64. *HPLC tracing of compound **I-31**.*

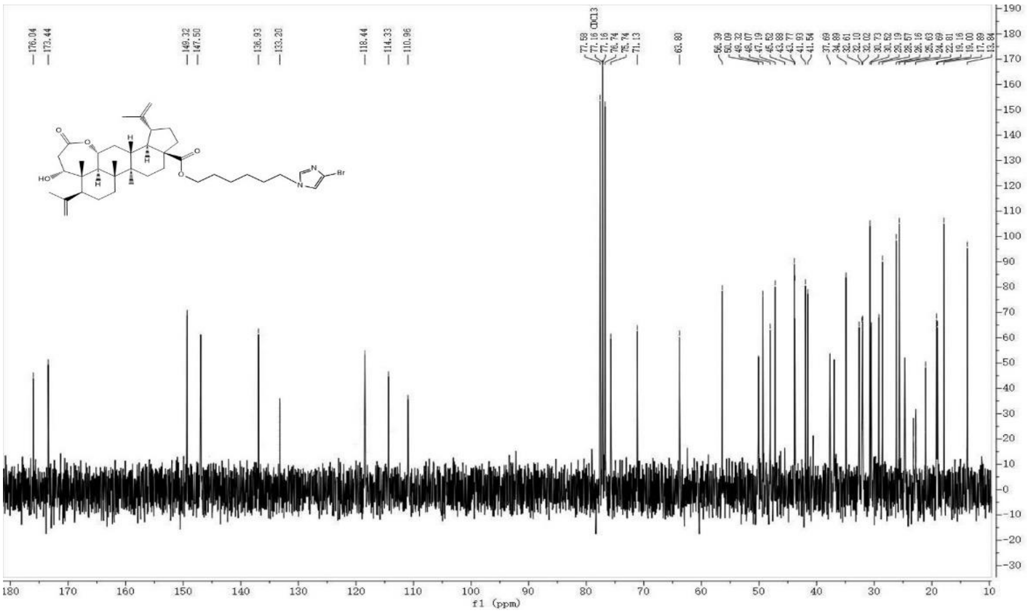

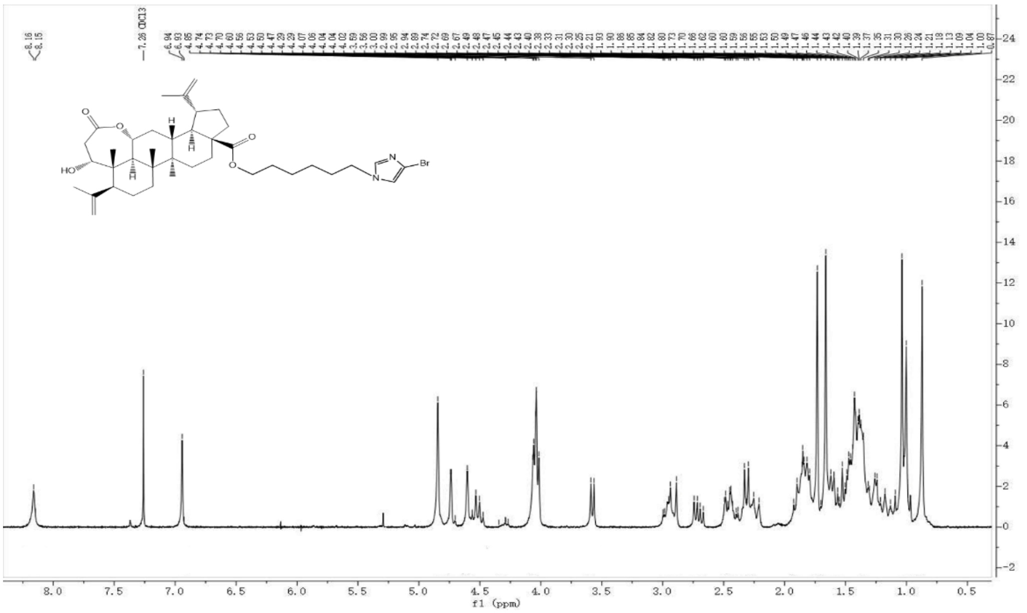

Figures S65. *^{13}C and ^{1}H NMR of compound **I-31**.*

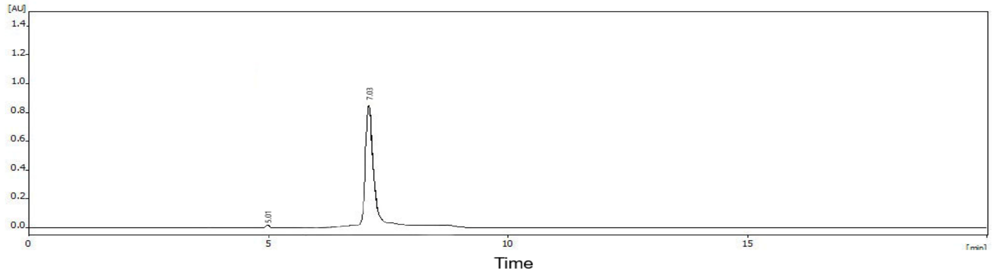

	RT	Area	Area %	Height
1	5.013	969.023	3.6	21.875
2	7.034	25948.285	96.4	2100.252

Time	A (water,0.5% Phosphoric acid)	B (Acetonitrile)
0	65	35
10	10	90
30	10	90
40	65	35

Figure S66. *HPLC tracing of compound **I-32**.*

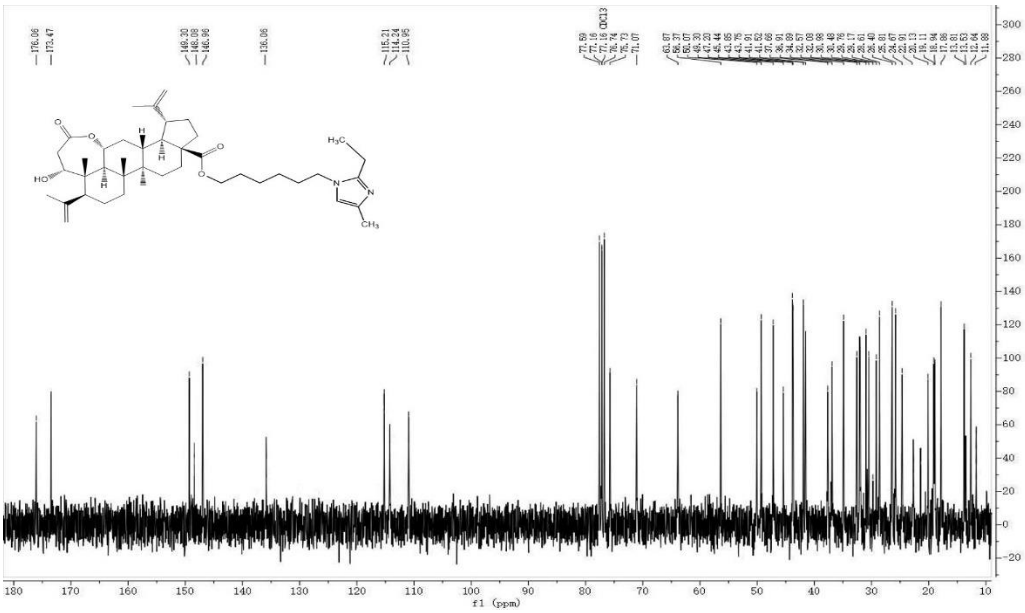

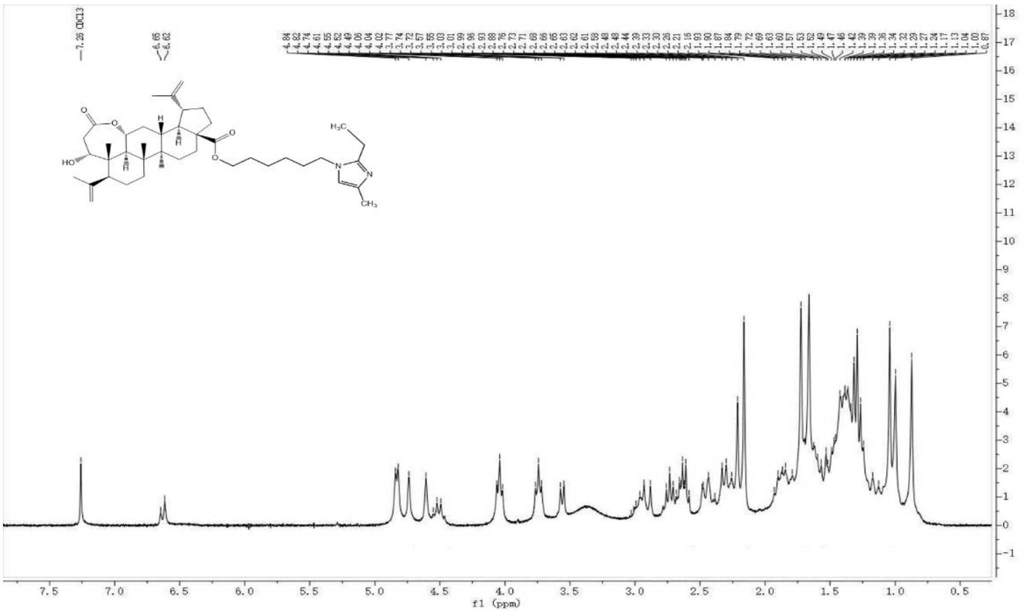

Figures S67. *^{13}C and ^{1}H NMR of compound **I-32**.*

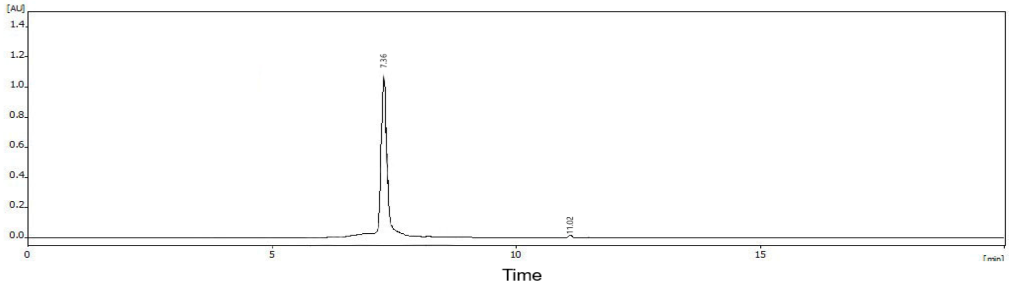

	RT	Area	Area %	Height
1	7.359	30156.558	97.4	2566.455
2	11.019	805.000	2.6	68.497

Time	A (water,0.5% Phosphoric acid)	B (Acetonitrile)
0	65	35
10	10	90
30	10	90
40	65	35

Figure S68. *HPLC tracing of compound **I-33**.*

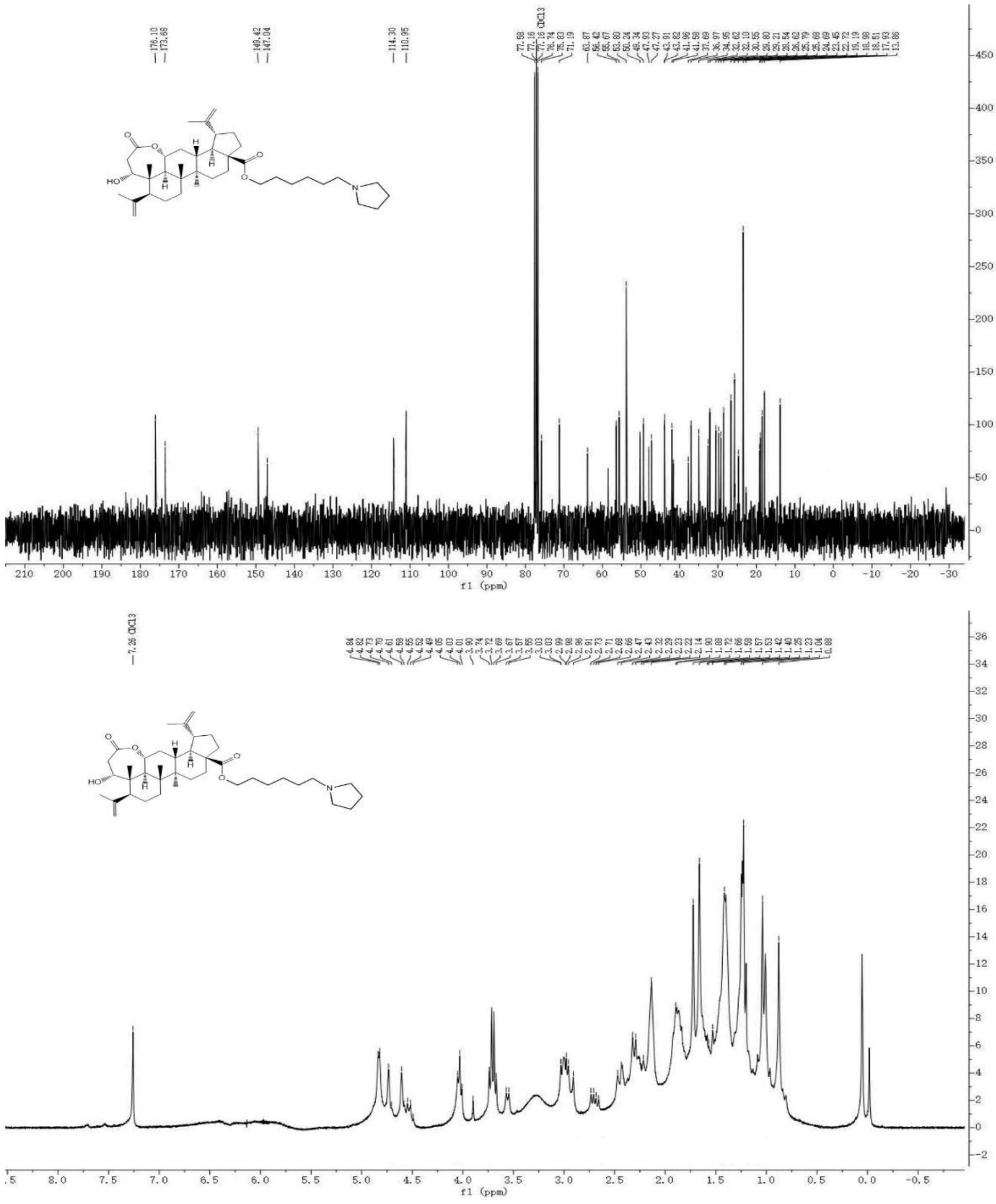

Figures S69. *^{13}C and ^{1}H NMR of compound **I-33**.*

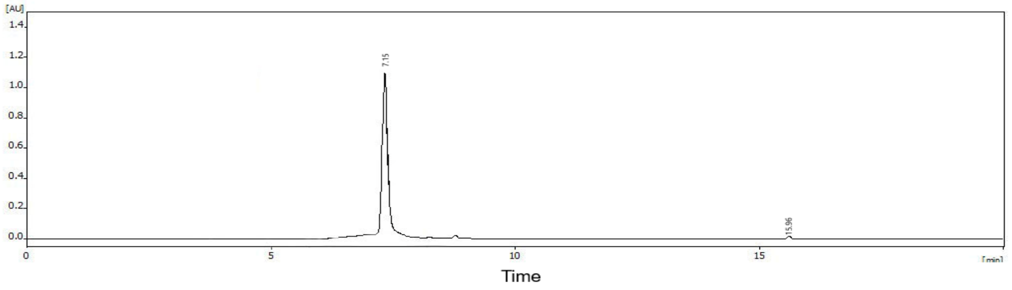

	RT	Area	Area %	Height
1	7.148	40254.525	98.2	1560.257
2	15.962	737.853	1.8	36.511

Time	A (water,0.5% Phosphoric acid)	B (Acetonitrile)
0	65	35
10	10	90
30	10	90
40	65	35

Figure S70. *HPLC tracing of compound **I-34**.*

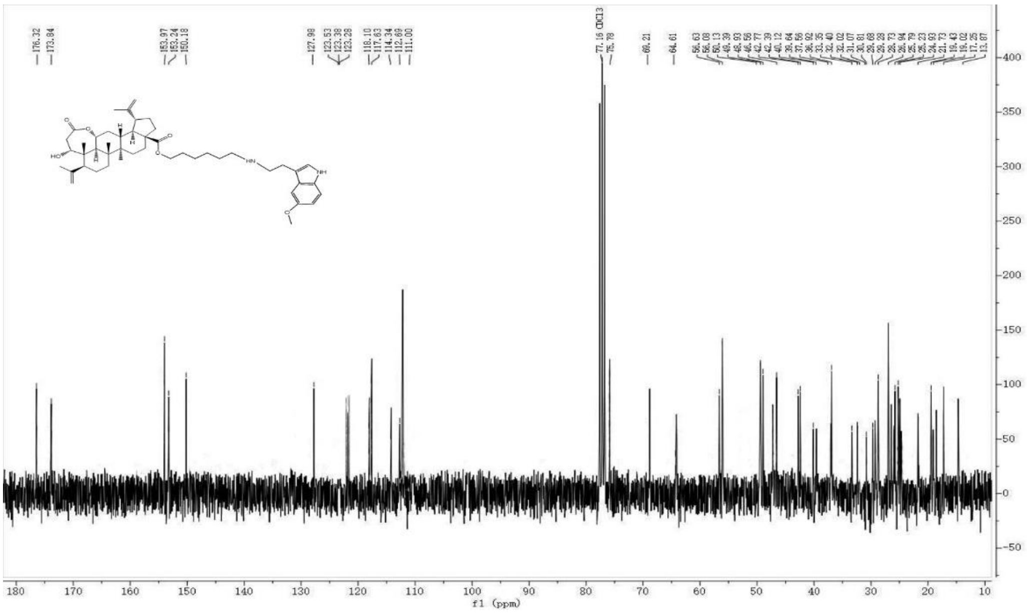

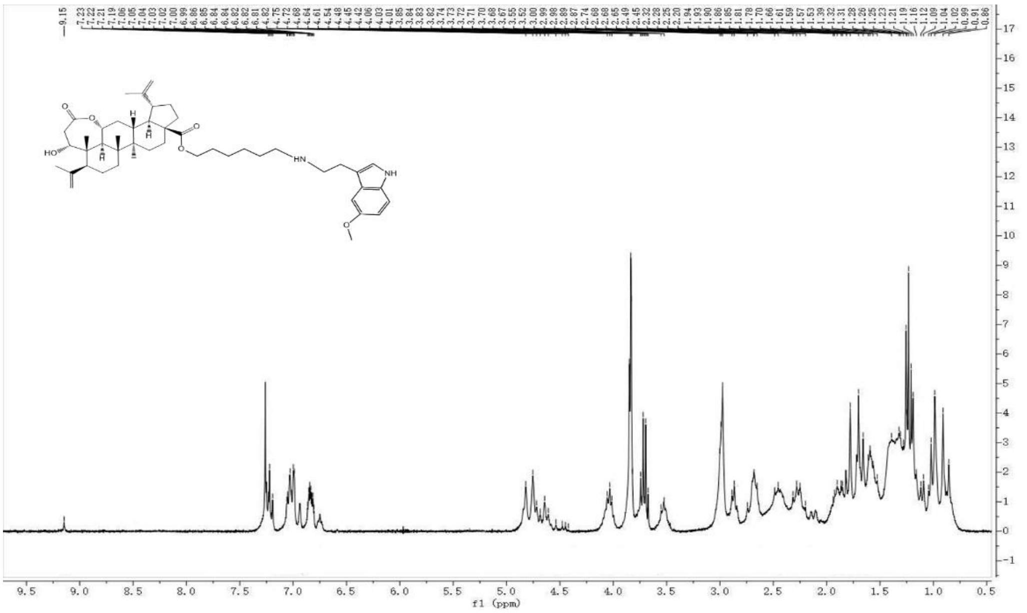

Figures S71. *^{13}C and ^{1}H NMR of compound **I-34**.*

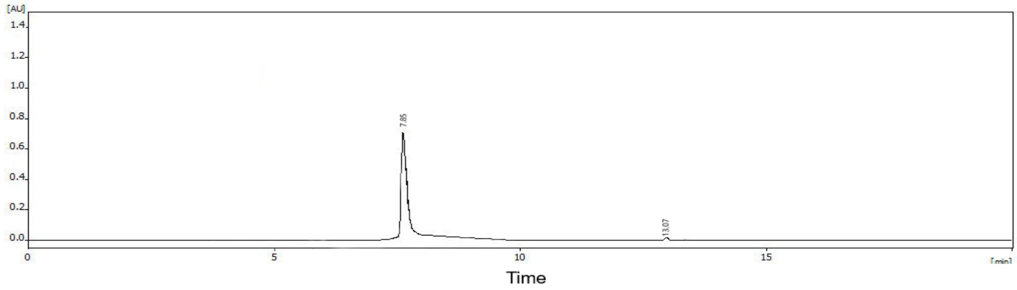

	RT	Area	Area %	Height
1	7.846	32215.502	97.2	1520.263
2	13.067	928.019	2.8	20.535

Time	A (water,0.5% Phosphoric acid)	B (Acetonitrile)
0	65	35
10	10	90
30	10	90
40	65	35

Figure S72. *HPLC tracing of compound **I-35**.*

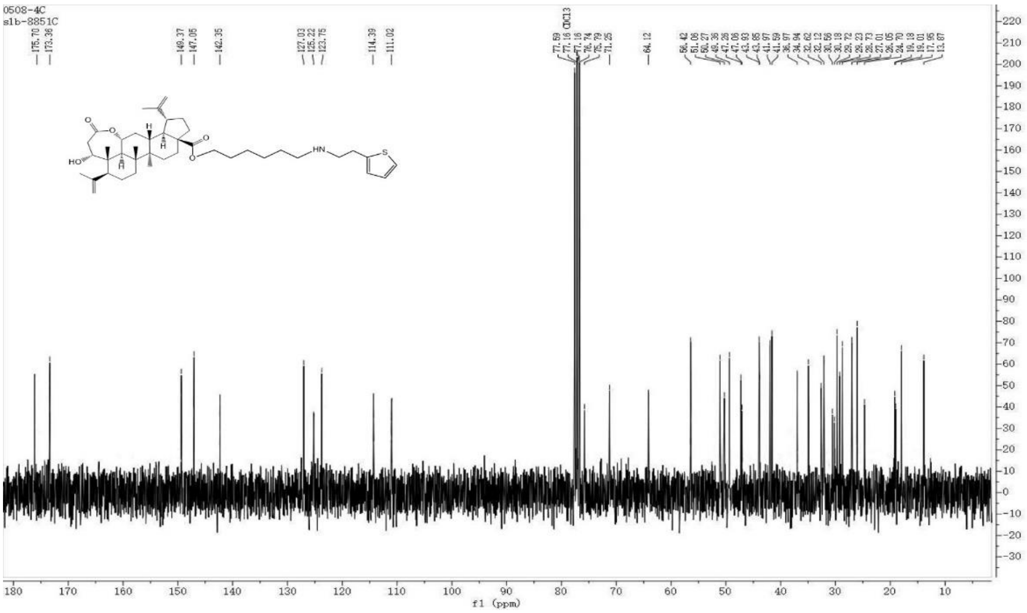

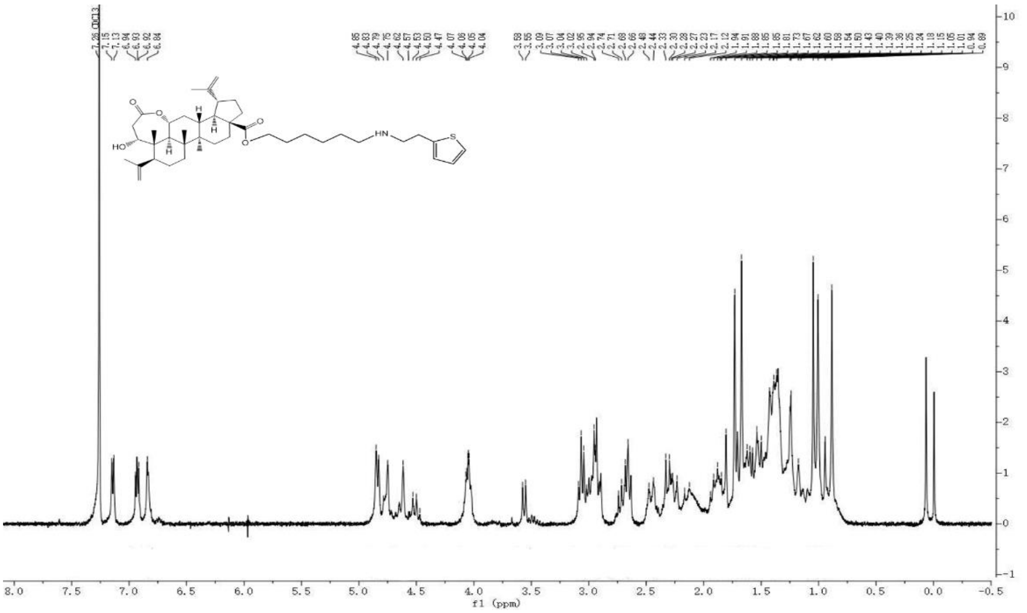

Figures S73. *^{13}C and ^{1}H NMR of compound **I-35**.*

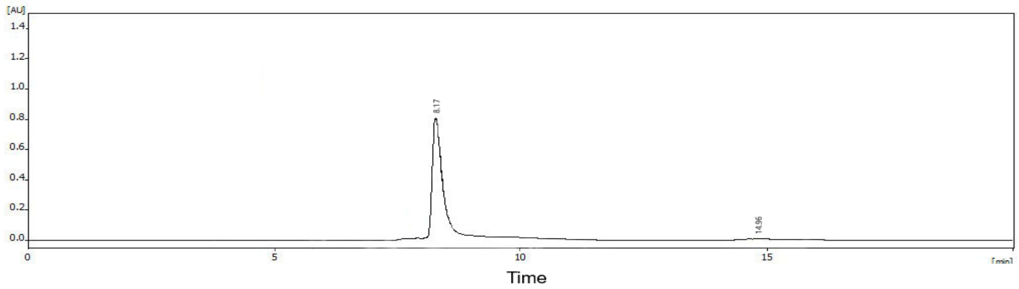

	RT	Area	Area %	Height
1	8.167	36554.583	96.5	3654.169
2	14.956	1325.813	3.5	133.218

Time	A (water,0.5% Phosphoric acid)	B (Acetonitrile)
0	65	35
10	10	90
30	10	90
40	65	35

Figure S74. *HPLC tracing of compound **I-36**.*

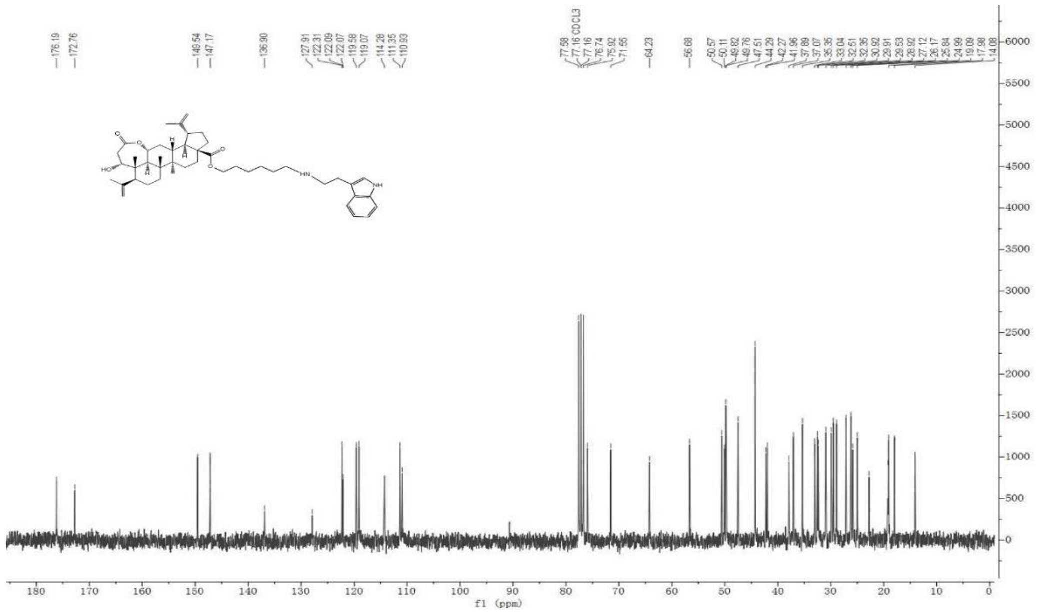

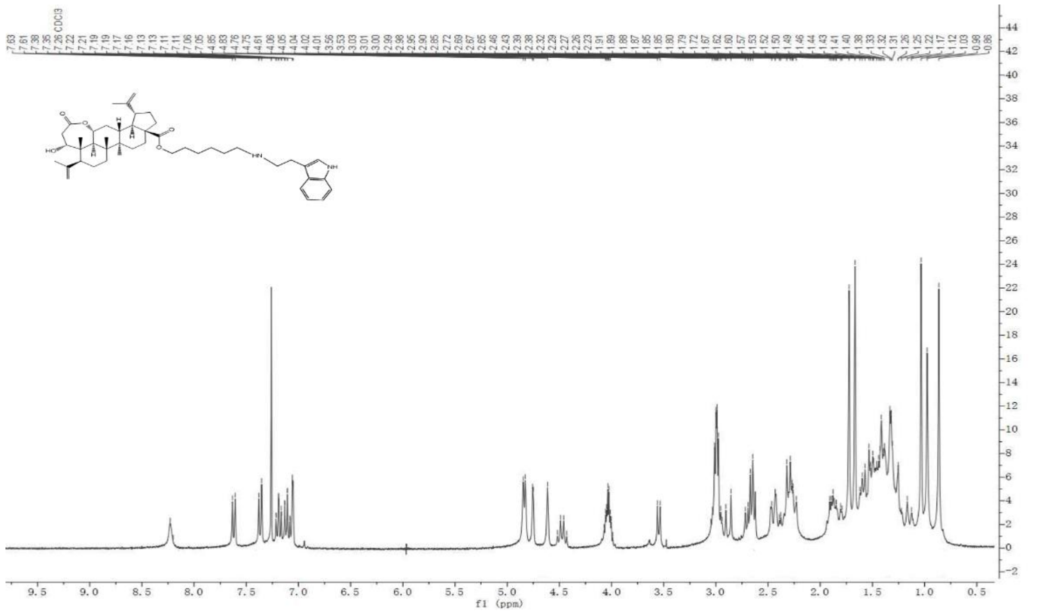

Figures S75. *^{13}C and ^{1}H NMR of compound **I-36**.*

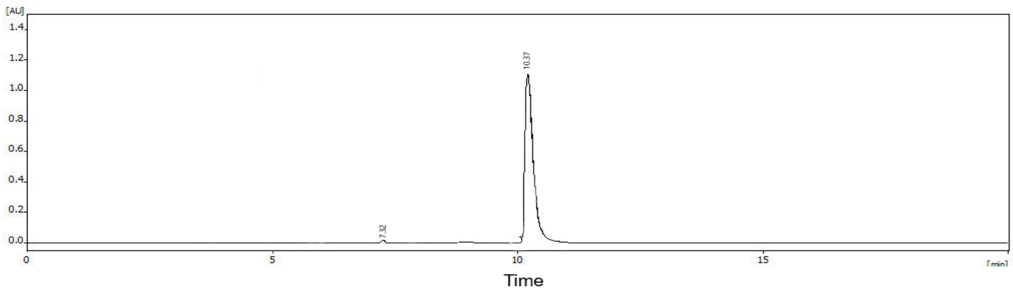

	RT	Area	Area %	Height
1	7.319	761.284	1.8	325.257
2	10.367	41532.321	98.2	2315.150

Time	A (water,0.5% Phosphoric acid)	B (Acetonitrile)
0	65	35
10	10	90
30	10	90
40	65	35

Figure S76. *HPLC tracing of compound **I-37**.*

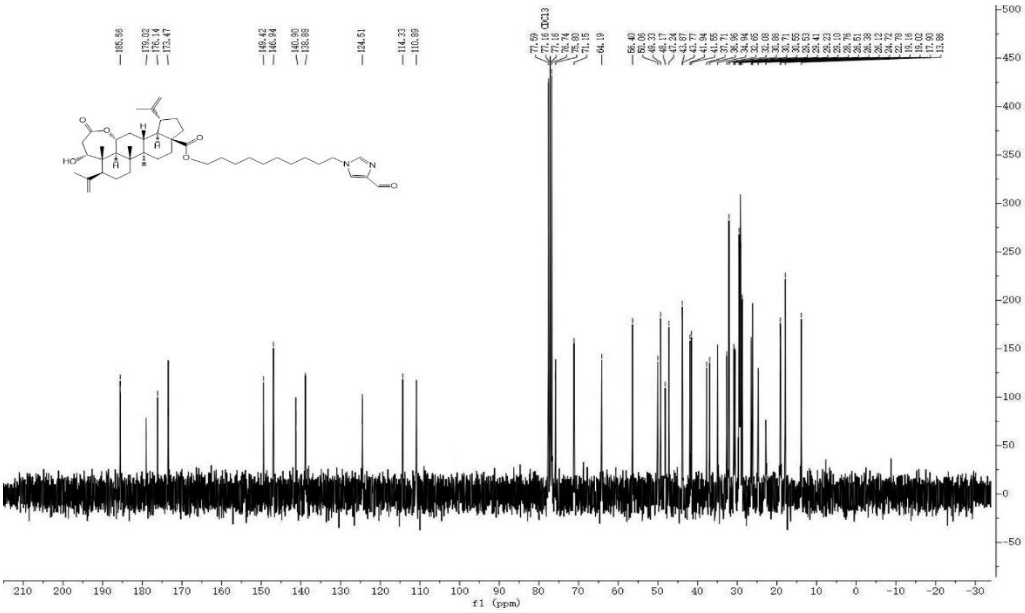

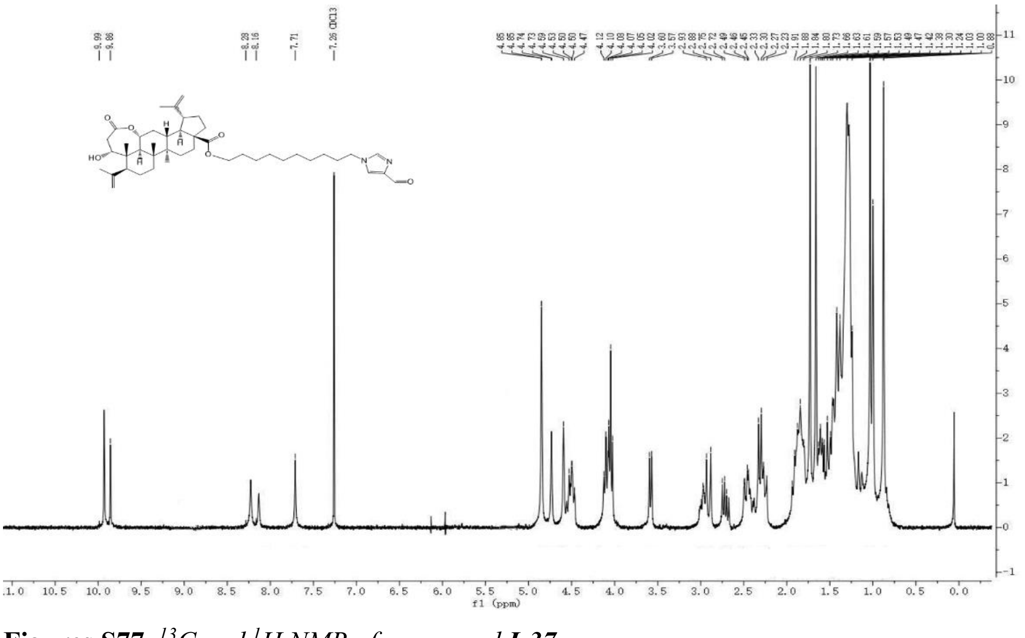

Figures S77. *^{13}C and ^{1}H NMR of compound **I-37**.*

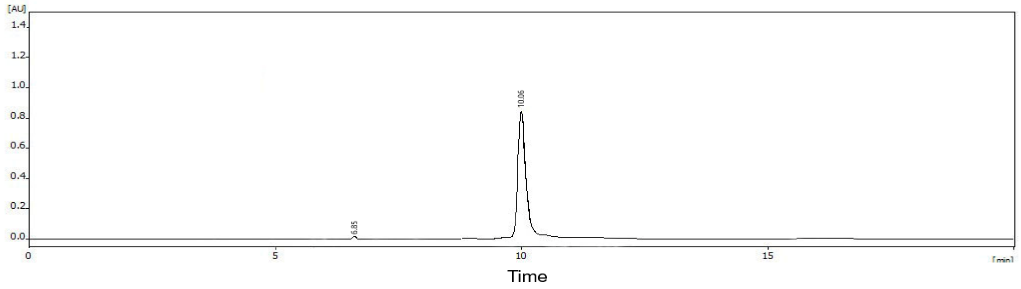

	RT	Area	Area %	Height
1	6.854	1369.946	3.8	65.162
2	10.059	34681.254	96.2	1458.579

Time	A (water,0.5% Phosphoric acid)	B (Acetonitrile)
0	65	35
10	10	90
30	10	90
40	65	35

Figure S78. *HPLC tracing of compound* ***I-38***.

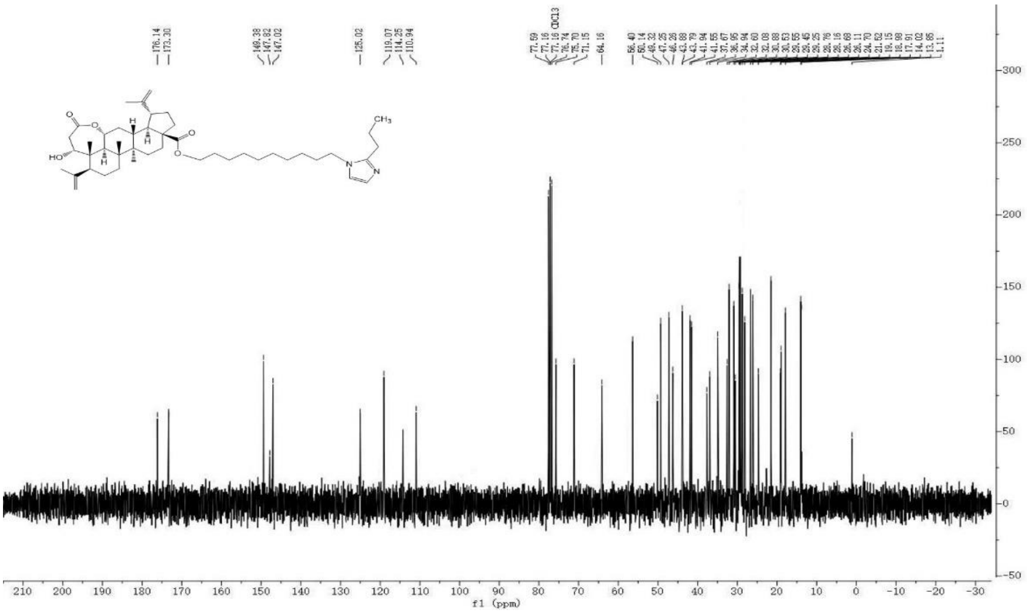

za

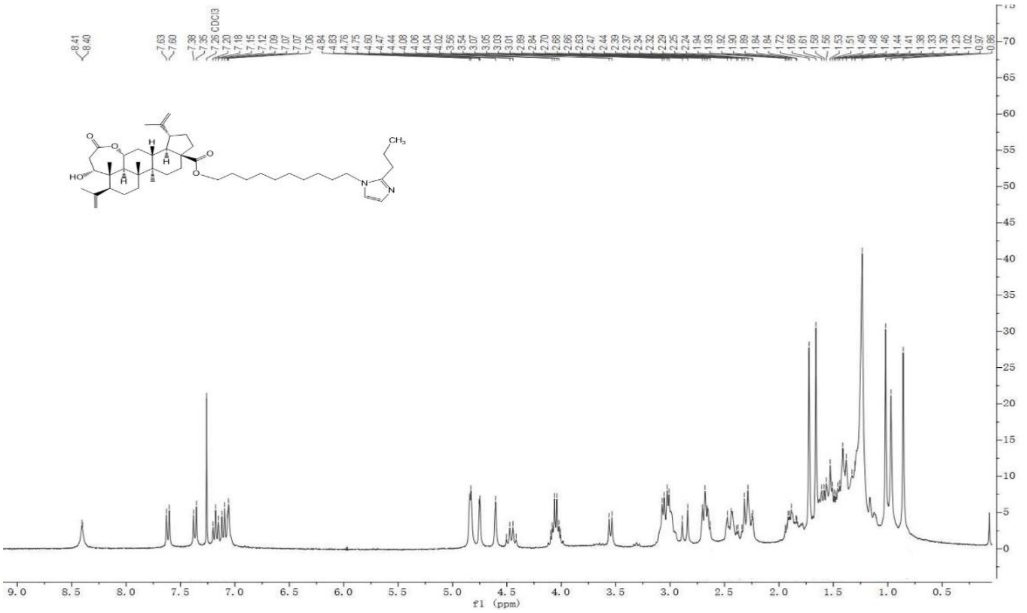

Figures S79. *^{13}C and ^{1}H NMR of compound* ***I-38***.

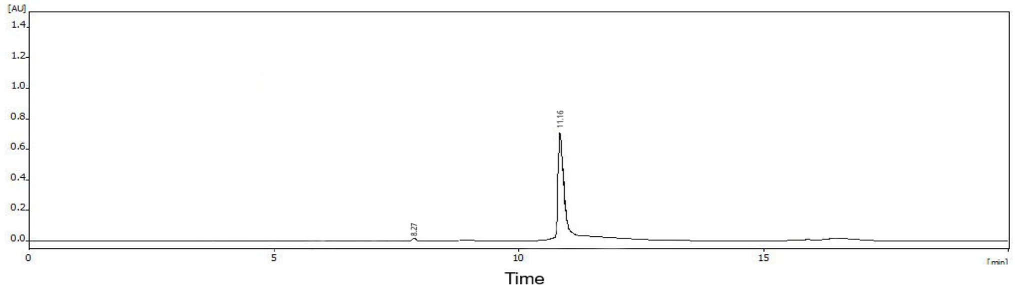

	RT	Area	Area %	Height
1	8.269	393.333	2.6	64.852
2	11.159	14734.862	97.4	2054.568

Time	A (water,0.5% Phosphoric acid)	B (Acetonitrile)
0	65	35
10	10	90
30	10	90
40	65	35

Figure S80. *HPLC tracing of compound **I-39**.*

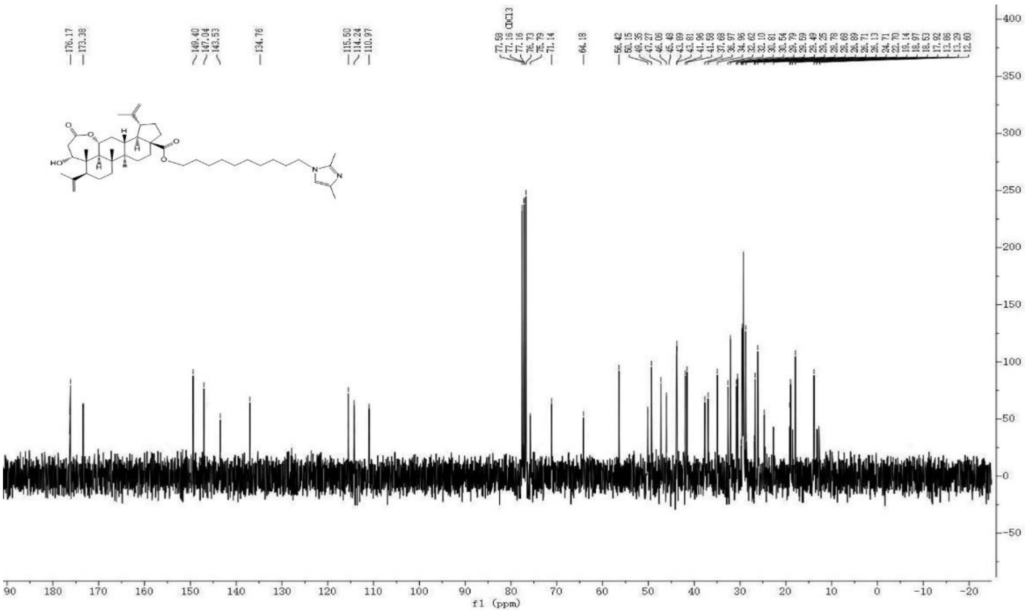

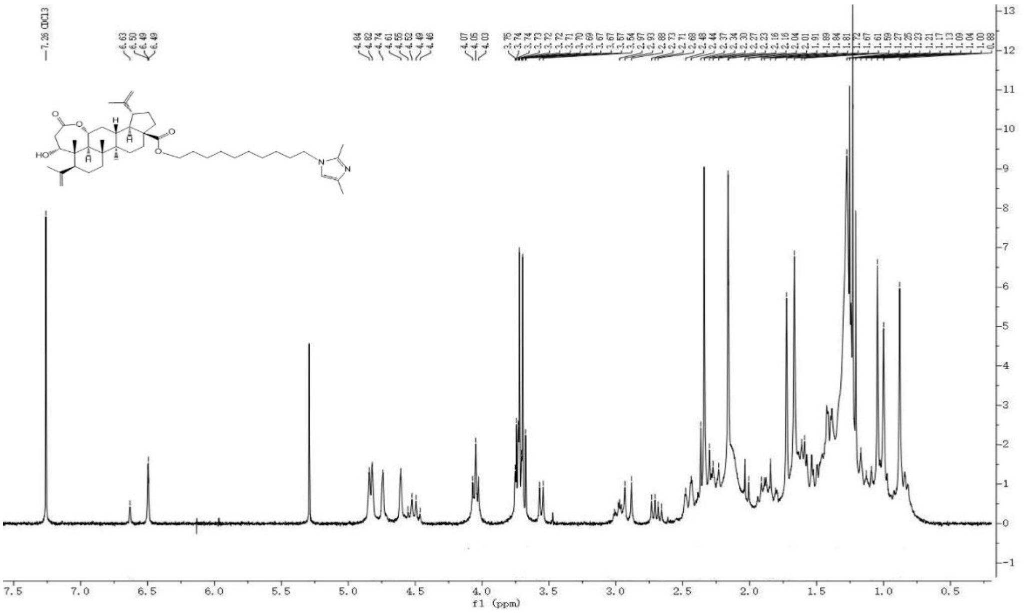

Figures S81. *^{13}C and ^{1}H NMR of compound **I-39**.*

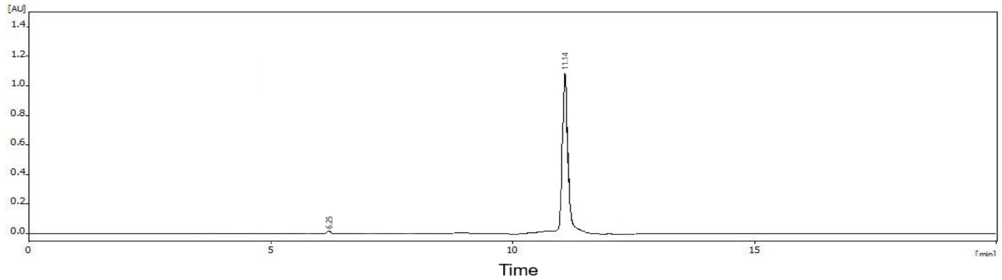

	RT	Area	Area %	Height
1	6.249	1683.805	4.4	62.482
2	11.137	36584.481	95.6	1325.560

Time	A (water,0.5% Phosphoric acid)	B (Acetonitrile)
0	65	35
10	10	90
30	10	90
40	65	35

Figure S82. *HPLC tracing of compound **I-40**.*

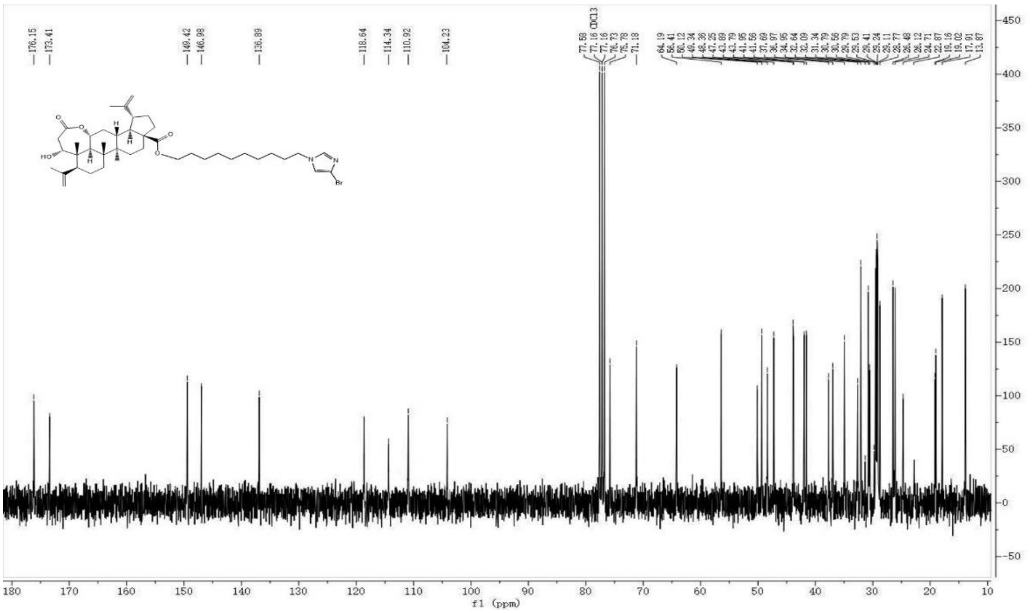

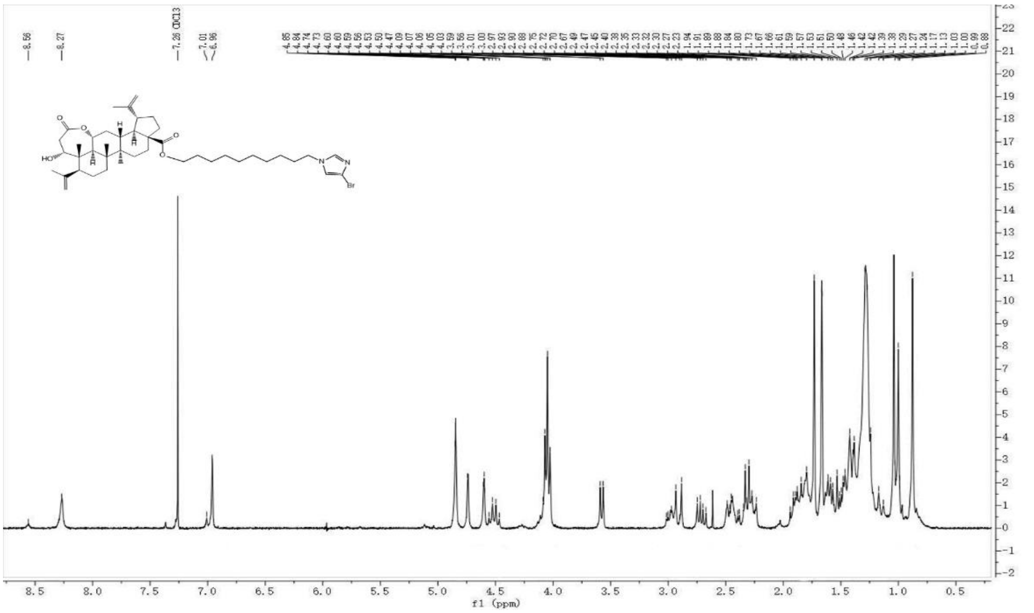

Figures S83. *^{13}C and ^{1}H NMR of compound **I-40**.*

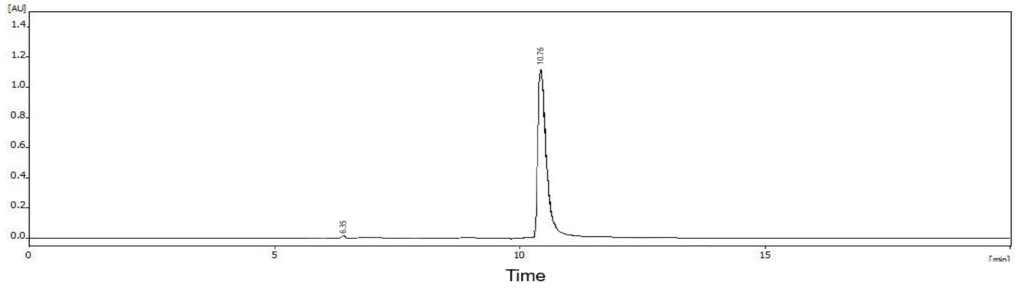

	RT	Area	Area %	Height
1	6.349	847.358	3.2	246.723
2	10.763	25632.583	96.8	1420.527

Time	A (water,0.5% Phosphoric acid)	B (Acetonitrile)
0	65	35
10	10	90
30	10	90
40	65	35

Figure S84. *HPLC tracing of compound* ***I-41***.

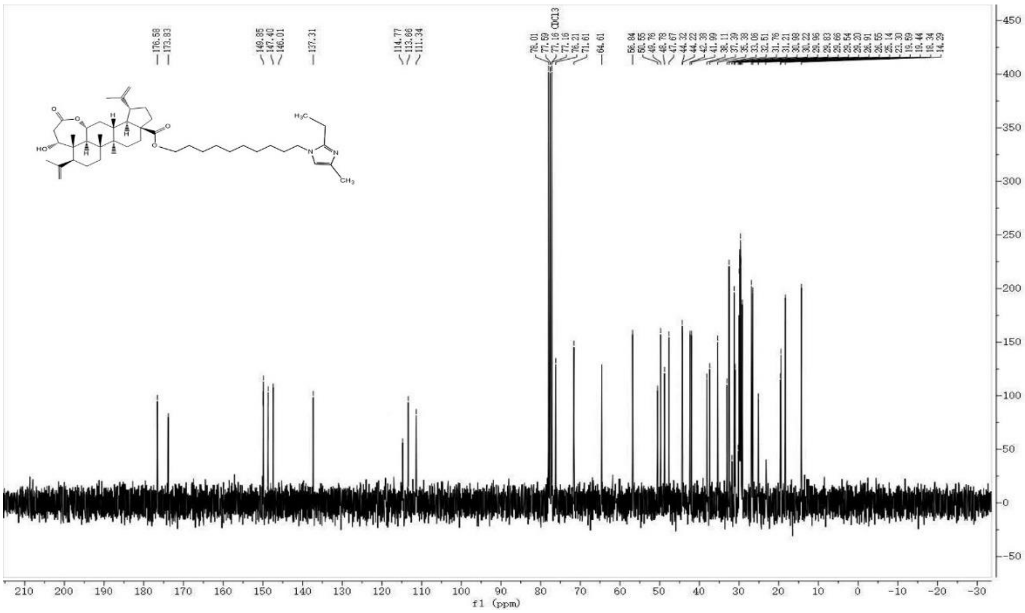

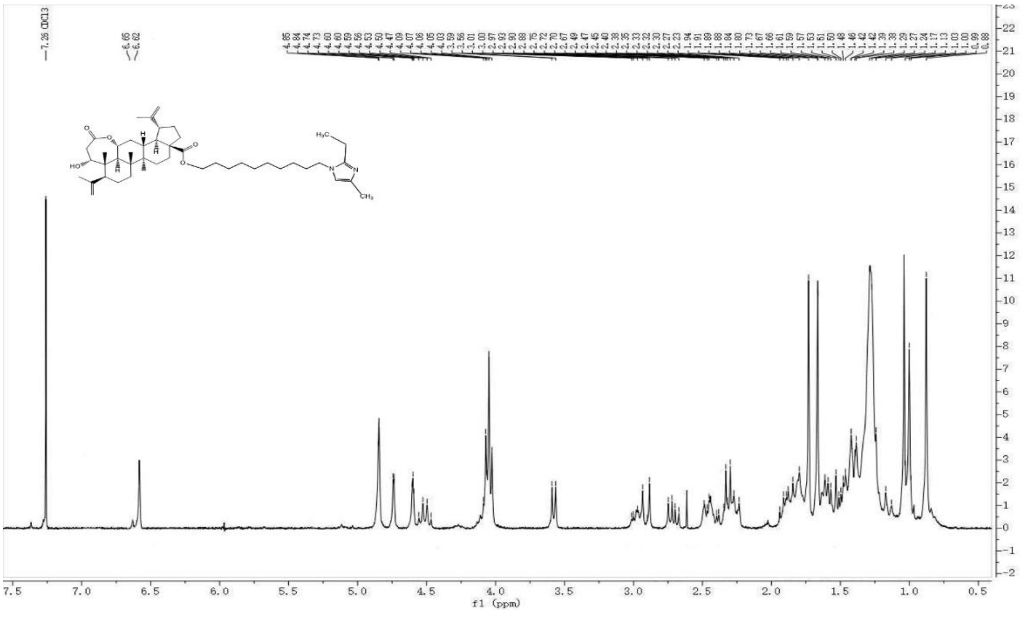

Figures S85. *^{13}C and ^{1}H NMR of compound* ***I-41***.

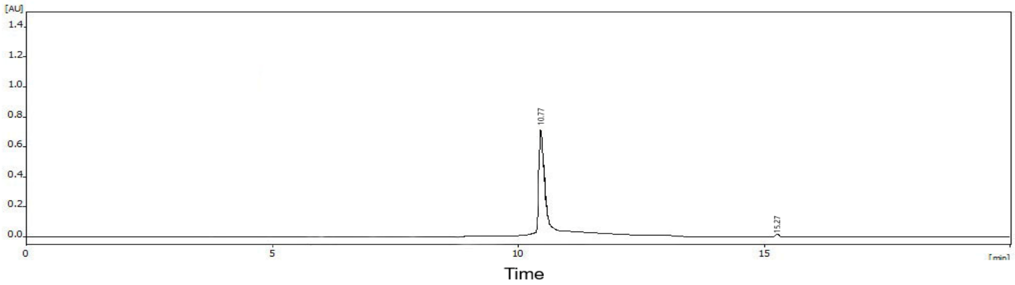

	RT	Area	Area %	Height
1	10.768	37821.592	97.9	1579.266
2	15.267	811.291	2.1	45.960

Time	A (water,0.5% Phosphoric acid)	B (Acetonitrile)
0	65	35
10	10	90
30	10	90
40	65	35

Figure S86. *HPLC tracing of compound* ***I-42***.

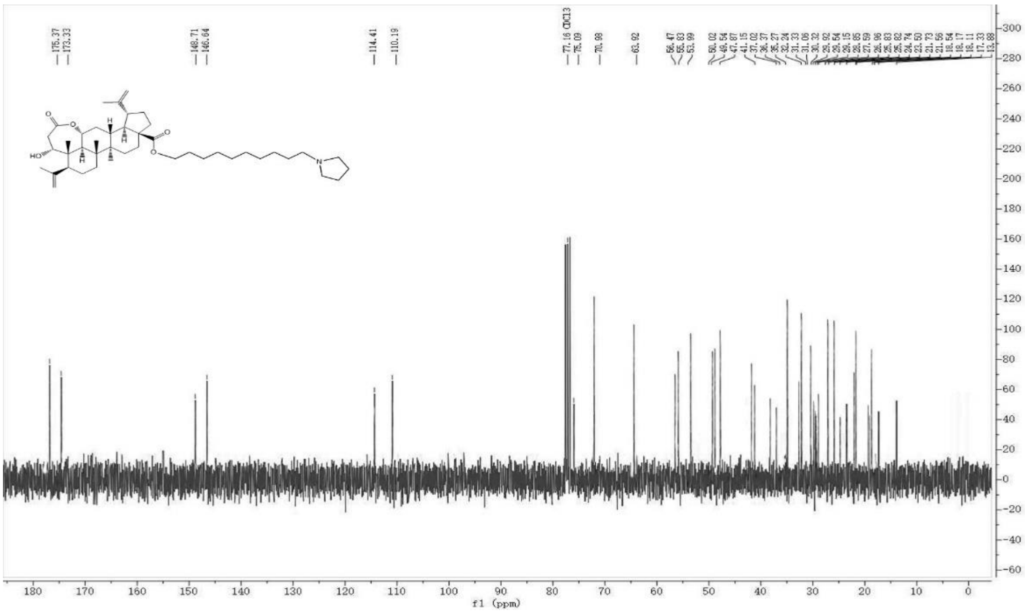

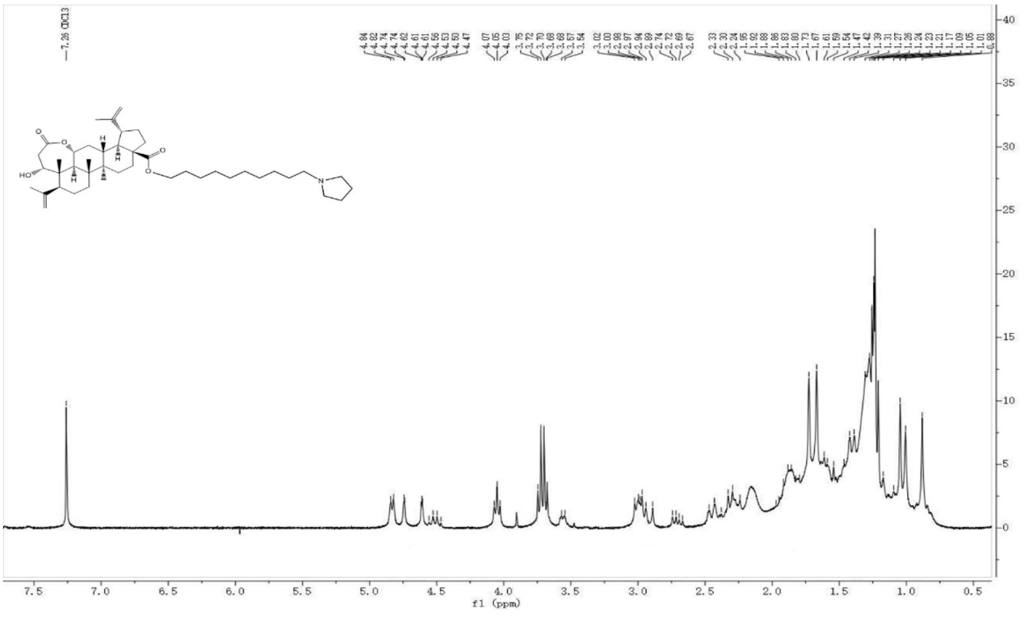

Figures S87. *^{13}C and ^{1}H NMR of compound* ***I-42***.

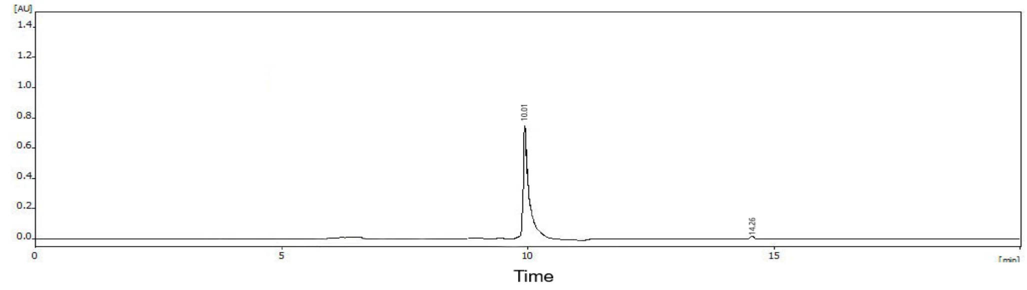

	RT	Area	Area %	Height
1	10.013	15968.265	98.2	2647.985
2	14.258	292.697	1.8	56.981

Time	A (water,0.5% Phosphoric acid)	B (Acetonitrile)
0	65	35
10	10	90
30	10	90
40	65	35

Figure S88. *HPLC tracing of compound **I-43**.*

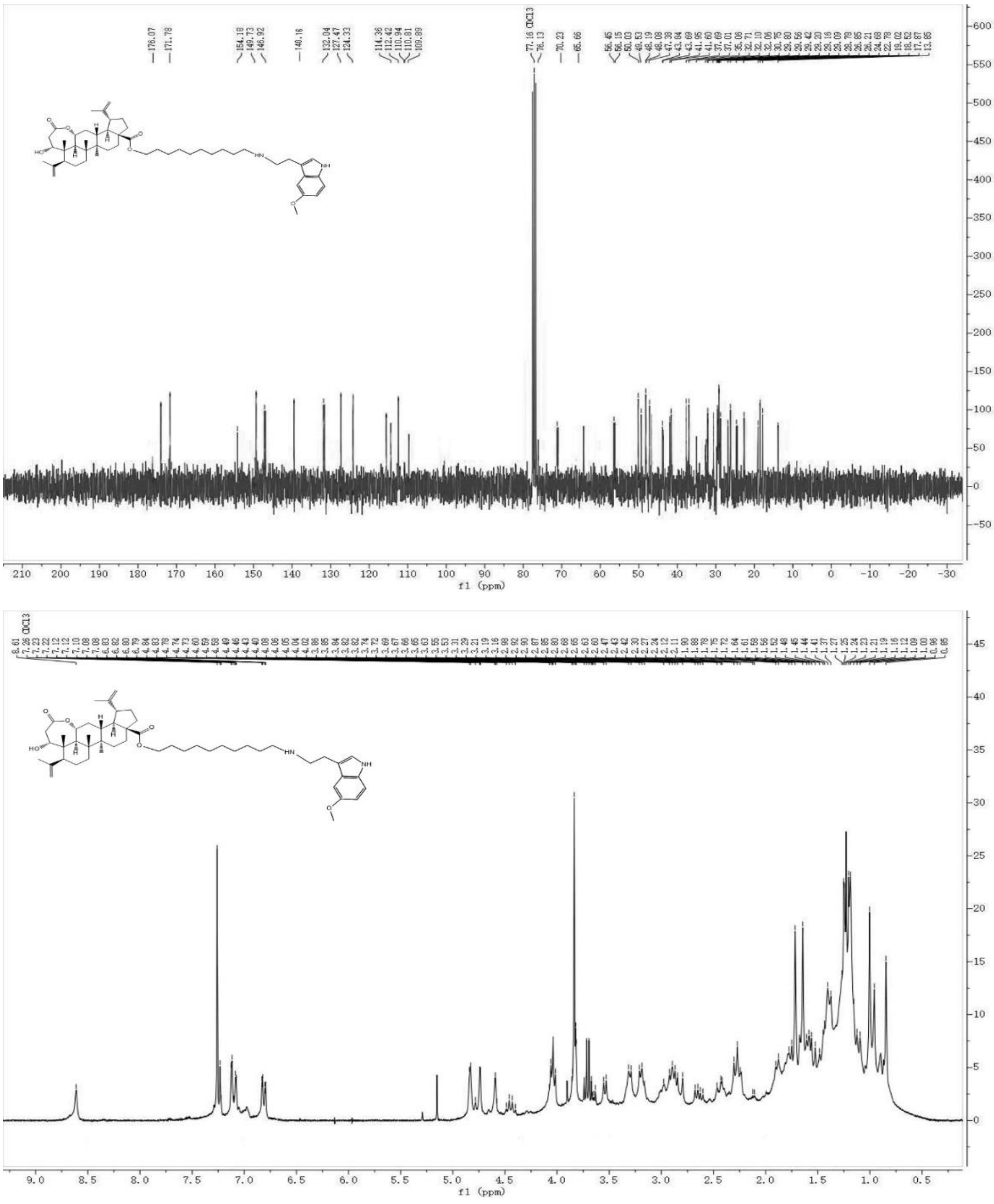

Figures S89. *^{13}C and ^{1}H NMR of compound **I-43**.*

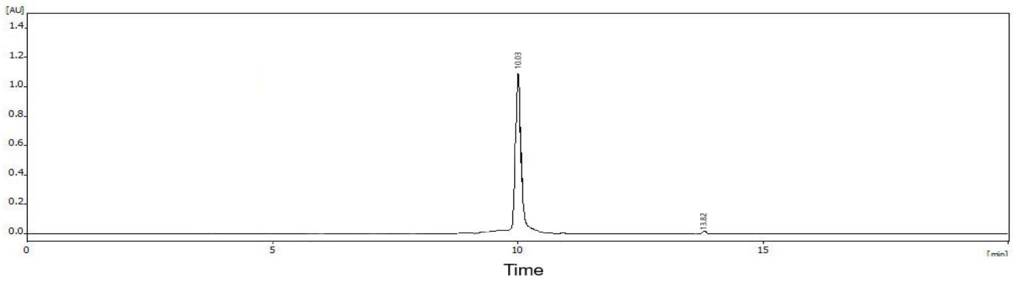

	RT	Area	Area %	Height
1	10.026	20697.286	97.3	1697.358
2	13.816	574.333	2.7	34.893

Time	A (water,0.5% Phosphoric acid)	B (Acetonitrile)
0	65	35
10	10	90
30	10	90
40	65	35

Figure S90. *HPLC tracing of compound **I-44**.*

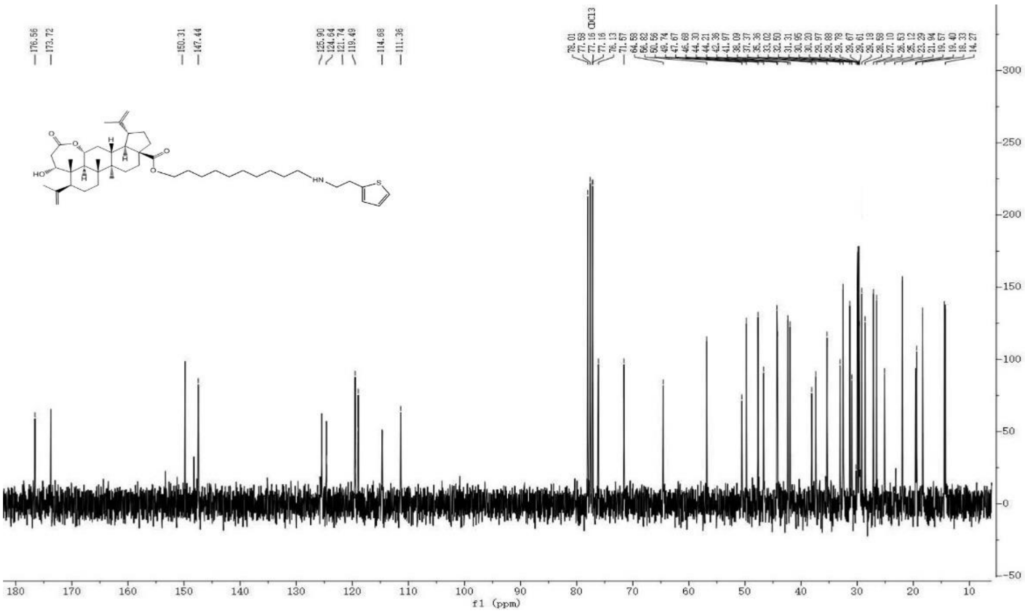

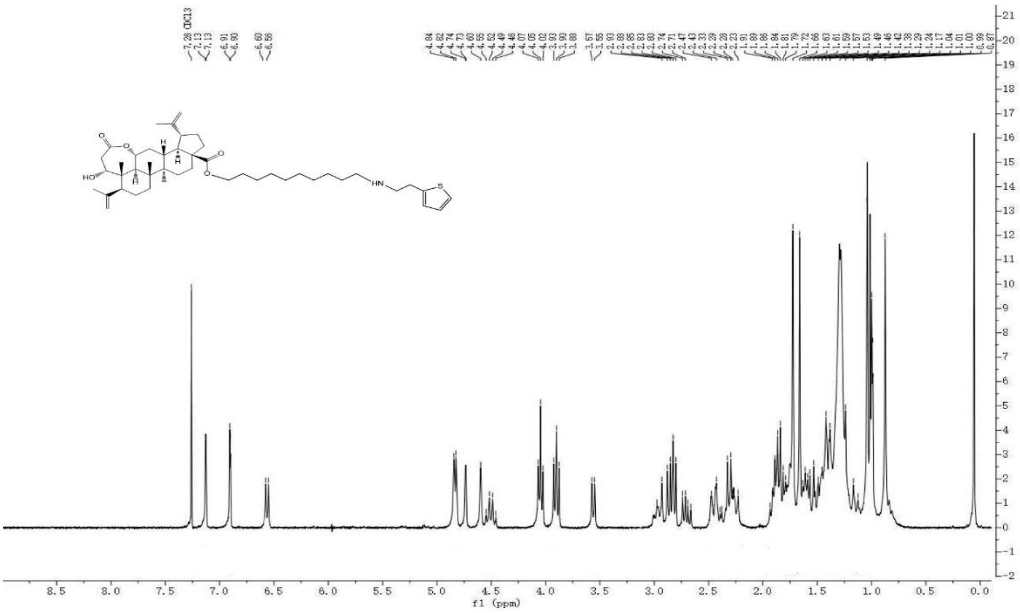

Figures S91. *^{13}C and ^{1}H NMR of compound **I-44**.*

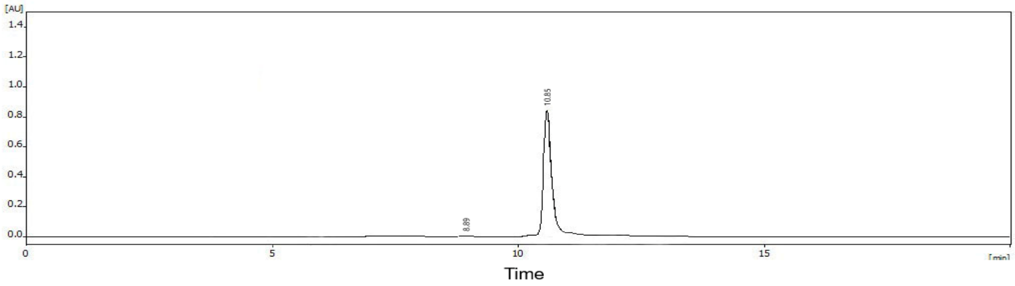

	RT	Area	Area %	Height
1	8.894	490.547	1.6	14.598
2	10.849	30168.655	98.4	2483.215

Time	A (water,0.5% Phosphoric acid)	B (Acetonitrile)
0	65	35
10	10	90
30	10	90
40	65	35

Figure S92. *HPLC tracing of compound* ***I-45***.

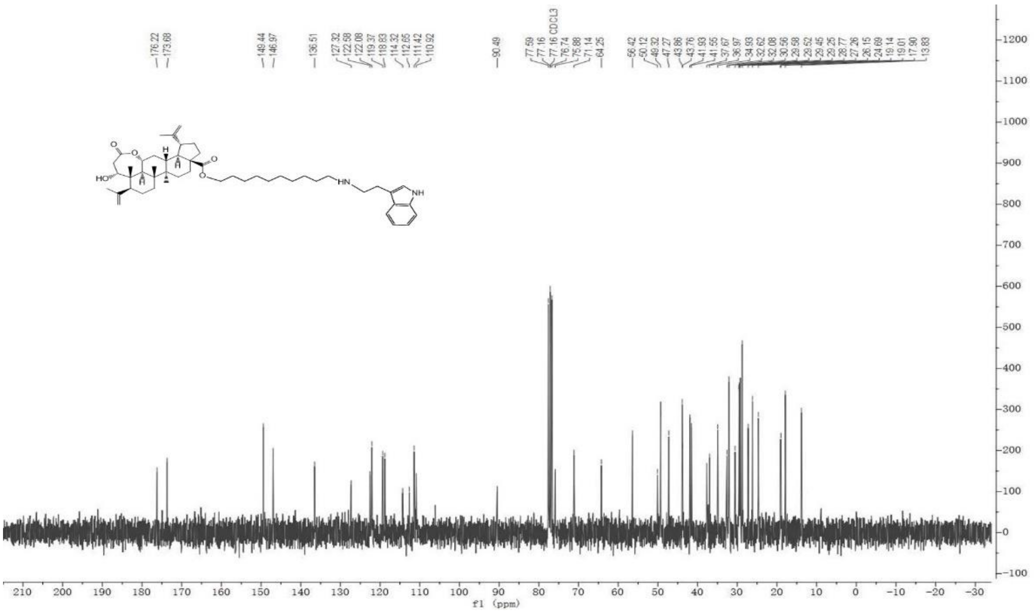

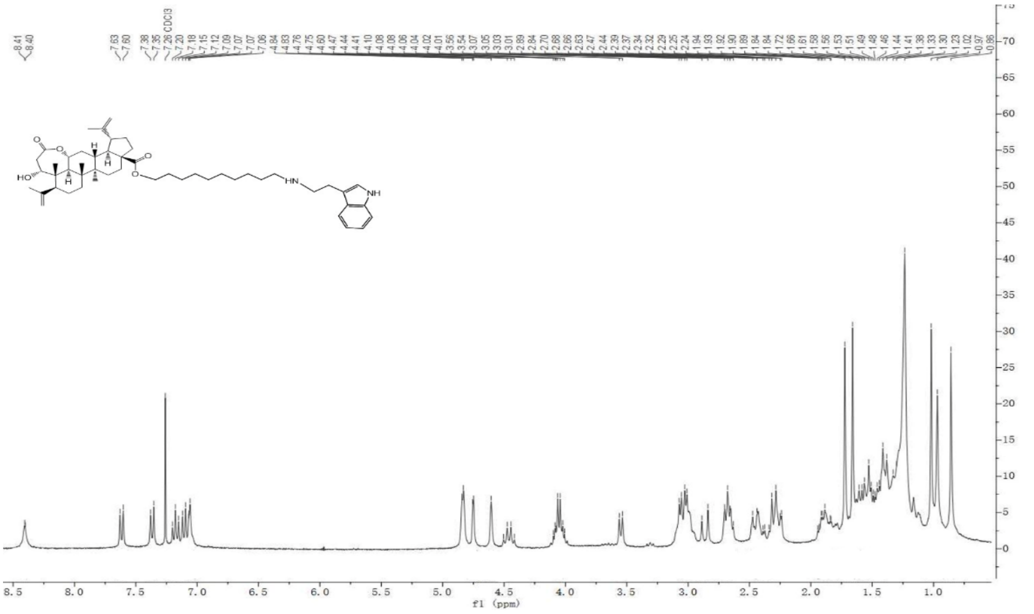

Figures S93. *^{13}C and ^{1}H NMR of compound* ***I-45***.

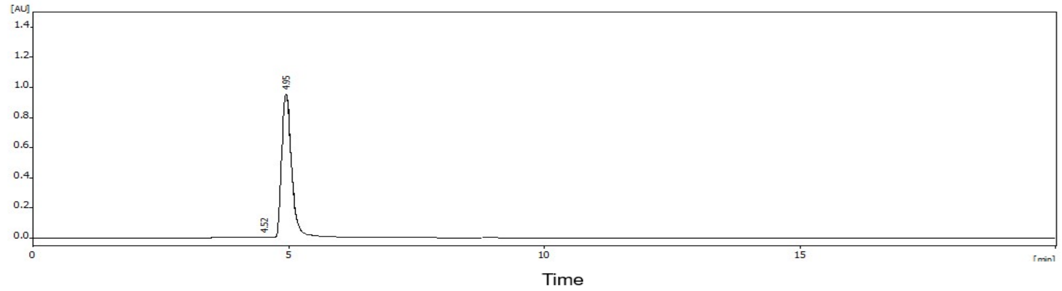

	RT	Area	Area %	Height
1	4.523	1279.238	3.6	15.284
2	4.950	34255.153	96.4	1064.598

Time	A (water,0.5% Phosphoric acid)	B (Acetonitrile)
0	65	35
10	10	90
30	10	90
40	65	35

Figure S94. *HPLC tracing of compound **II-46**.*

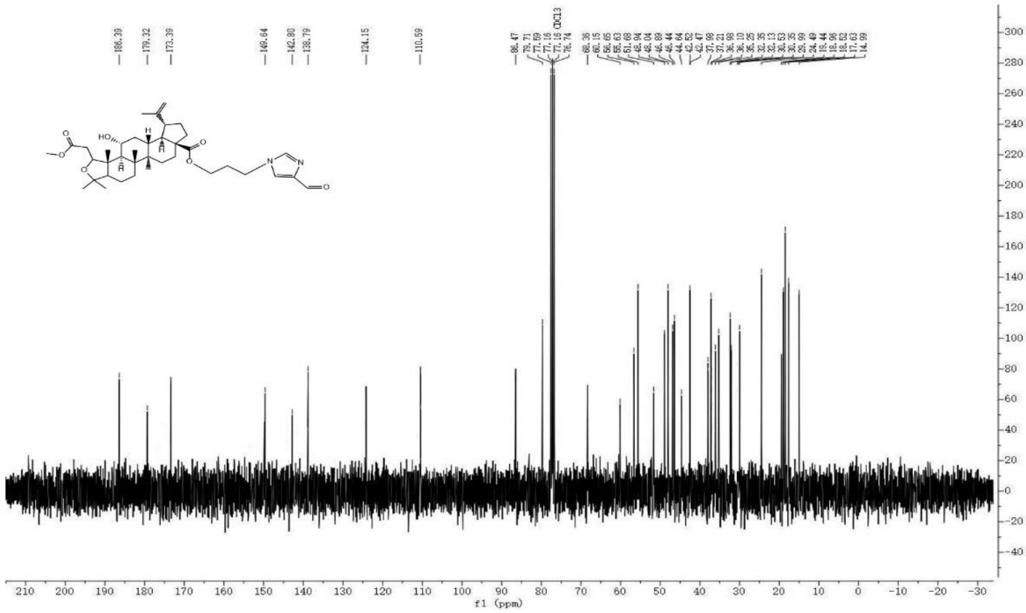

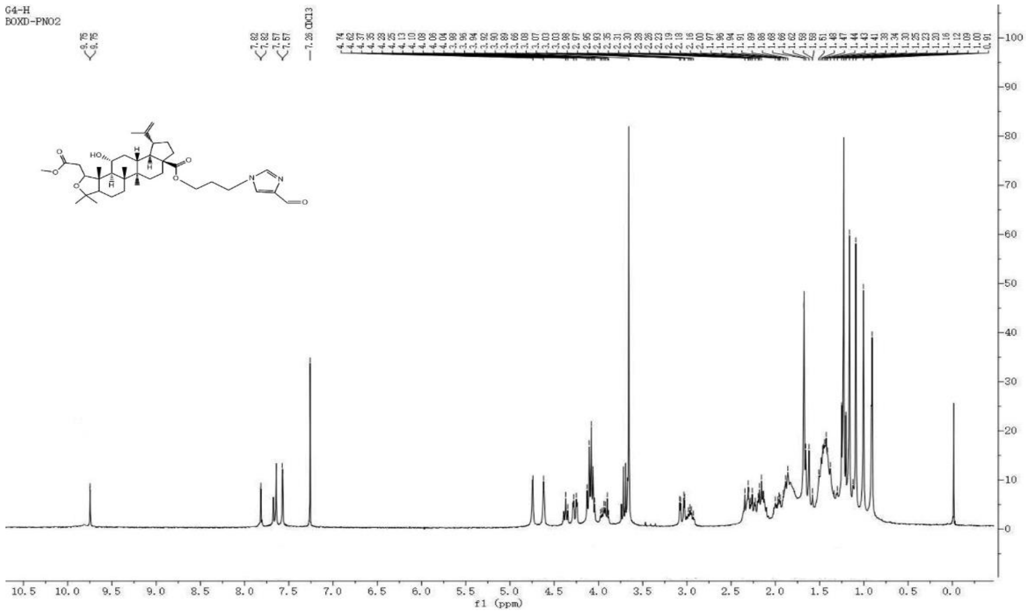

Figures S95. *^{13}C and ^{1}H NMR of compound **II-46**.*

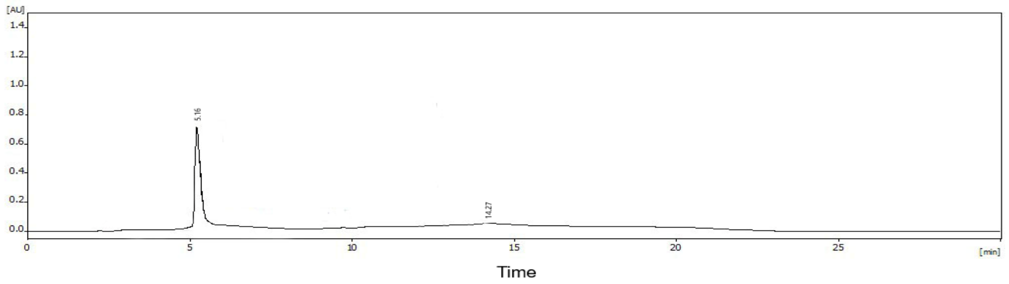

	RT	Area	Area %	Height
1	5.156	19435.226	98.0	708.666
2	14.265	396.638	2.0	5.199

Time	A (water,0.5% Phosphoric acid)	B (Acetonitrile)
0	65	35
10	10	90
30	10	90
40	65	35

Figure S96. *HPLC tracing of compound **II-47**.*

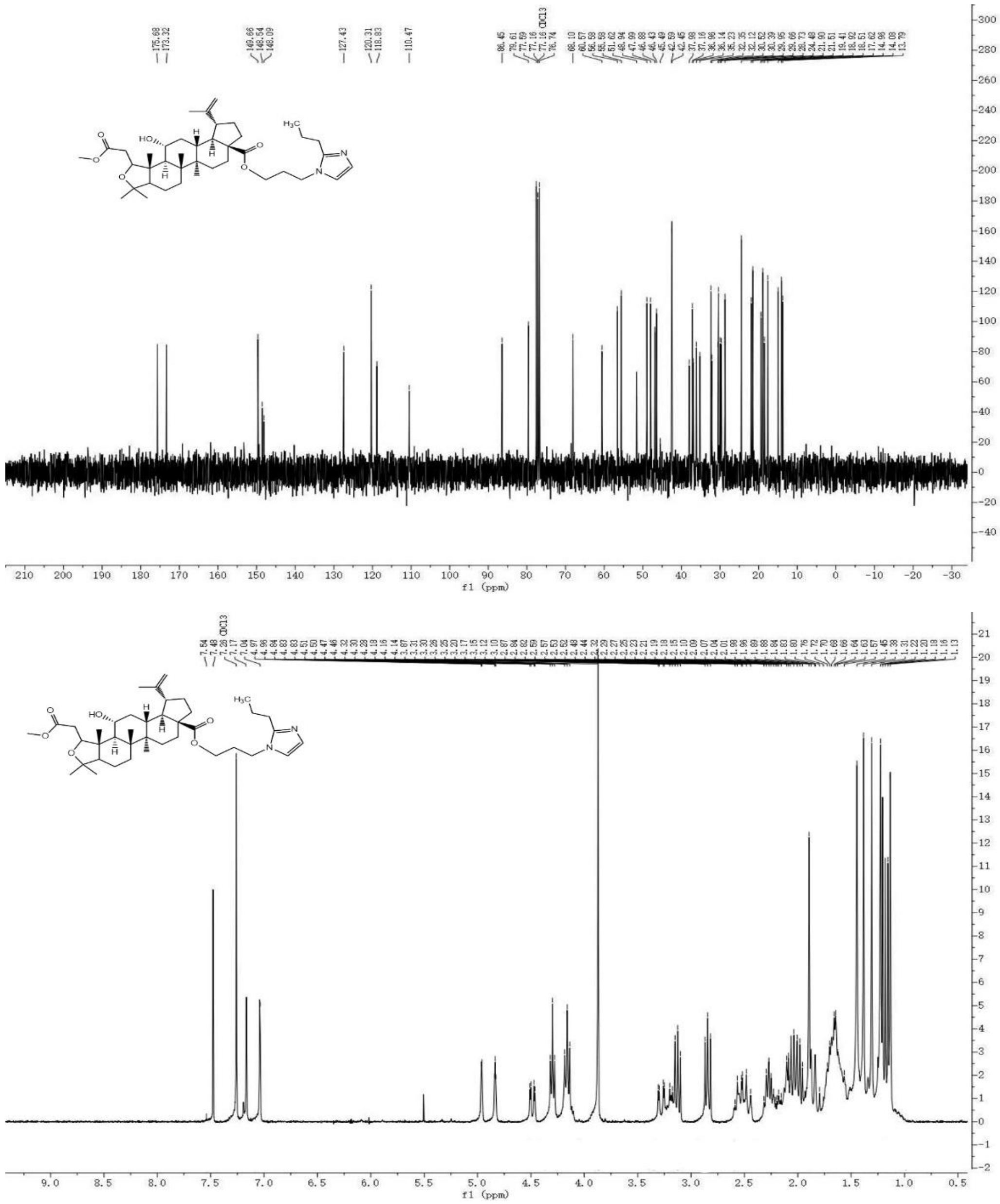

Figures S97. *^{13}C and ^{1}H NMR of compound **II-47**.*

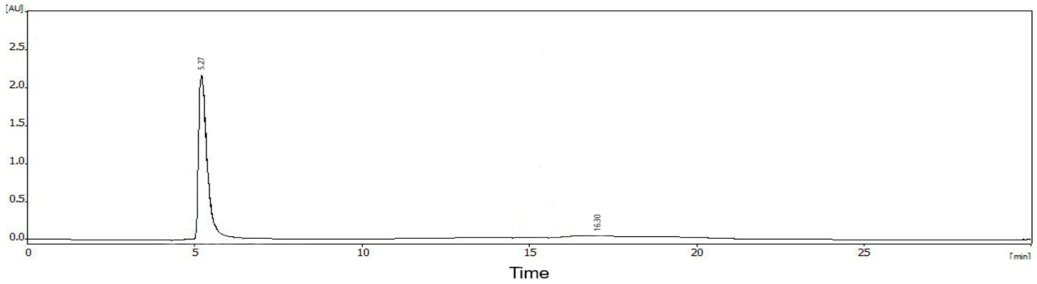

	RT	Area	Area %	Height
1	5.267	46700.403	99.1	2165.809
2	16.295	417.753	0.9	18.898

Time	A (water,0.5% Phosphoric acid)	B (Acetonitrile)
0	65	35
10	10	90
30	10	90
40	65	35

Figure S98. *HPLC tracing of compound **II-48**.*

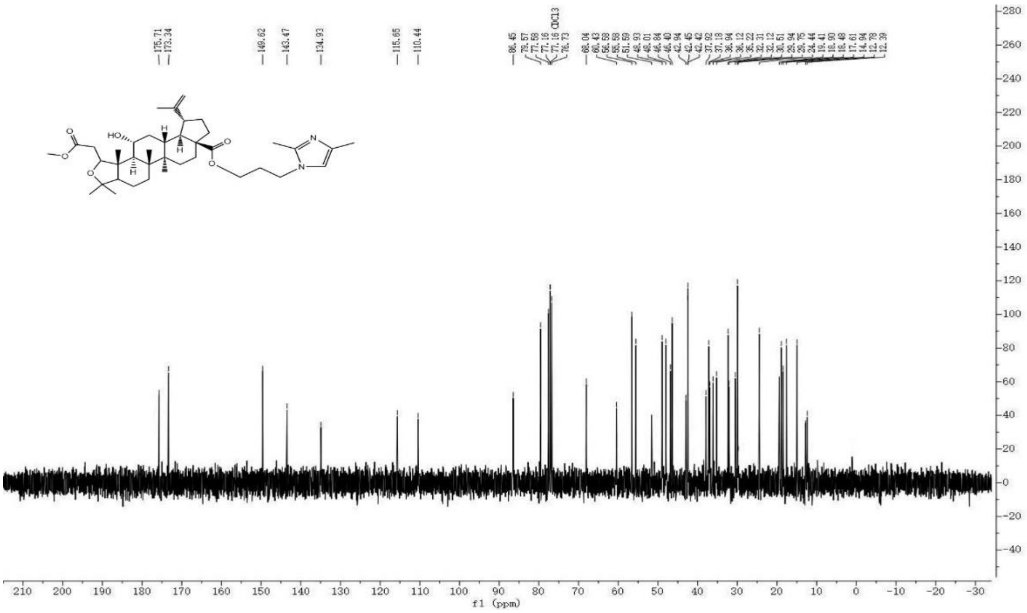

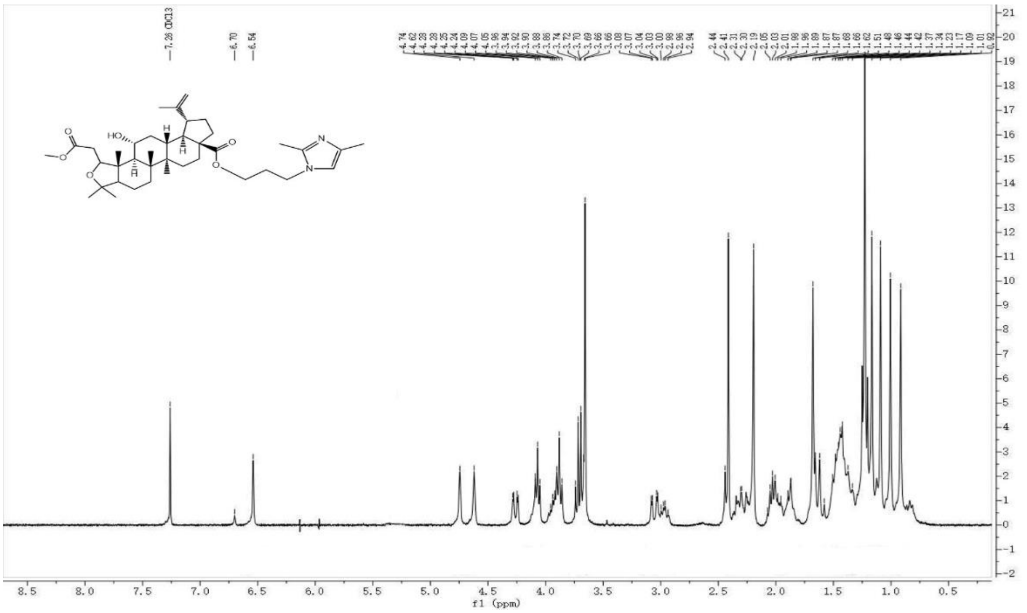

Figures S99. *^{13}C and ^{1}H NMR of compound **II-48**.*

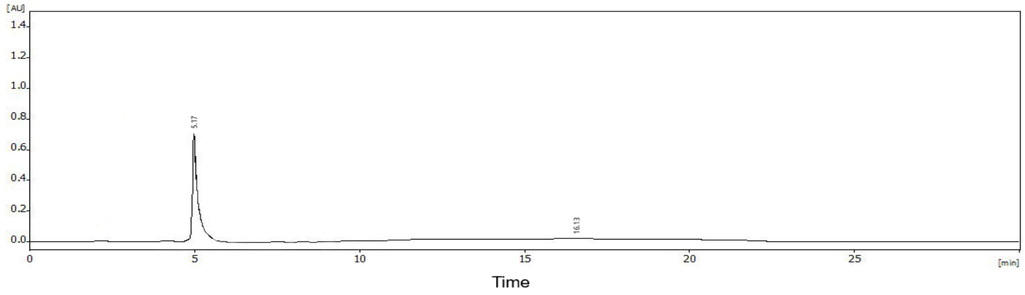

	RT	Area	Area %	Height
1	5.168	8846.283	95.3	707.003
2	16.134	435.778	4.7	8.123

Time	A (water,0.5% Phosphoric acid)	B (Acetonitrile)
0	65	35
10	10	90
30	10	90
40	65	35

Figure S100. *HPLC tracing of compound **II-49**.*

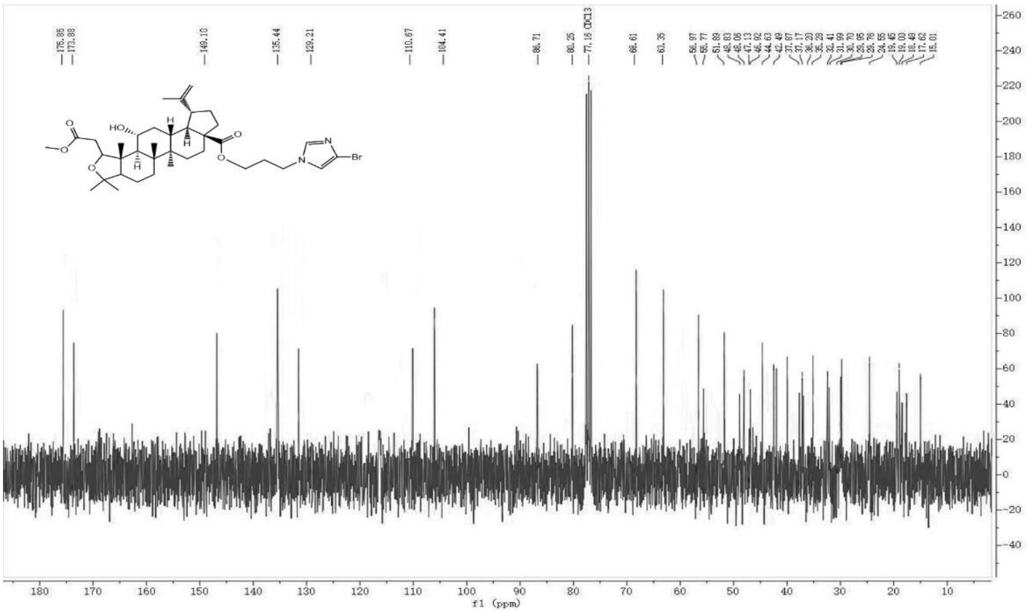

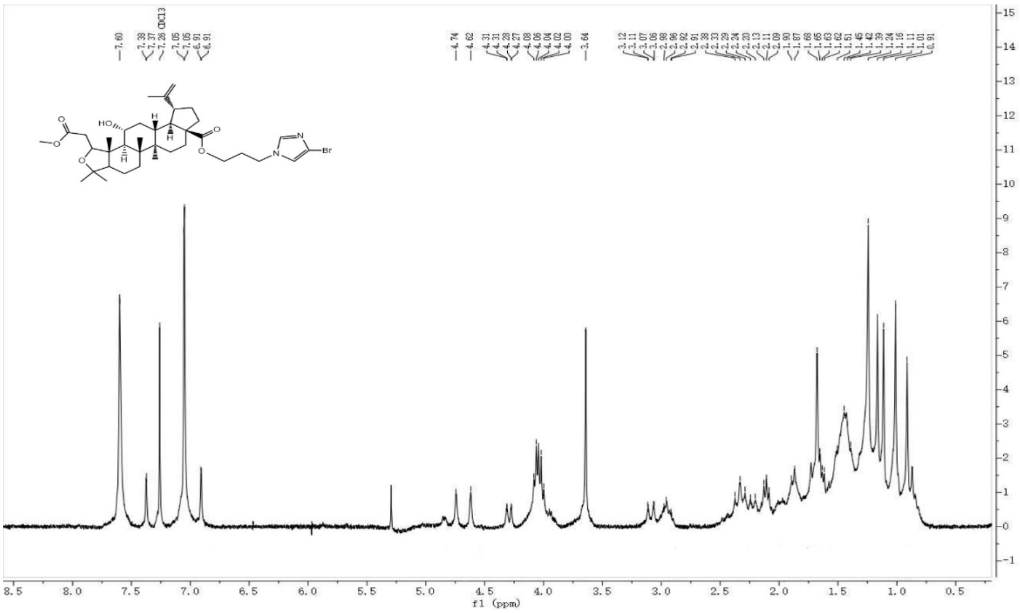

Figures S101. *^{13}C and ^{1}H NMR of compound **II-49**.*

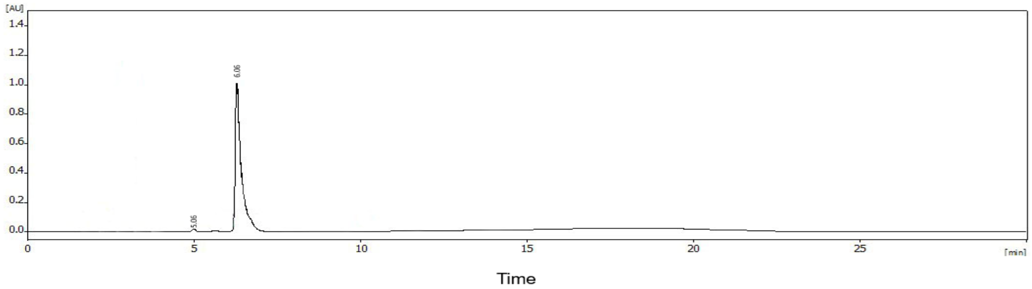

	RT	Area	Area %	Height
4	5.061	355.161	2.7	3.846
5	6.059	12798.935	97.3	1009.481

Time	A (water,0.5% Phosphoric acid)	B (Acetonitrile)
0	65	35
10	10	90
30	10	90
40	65	35

Figure S102. *HPLC tracing of compound* ***II-50***.

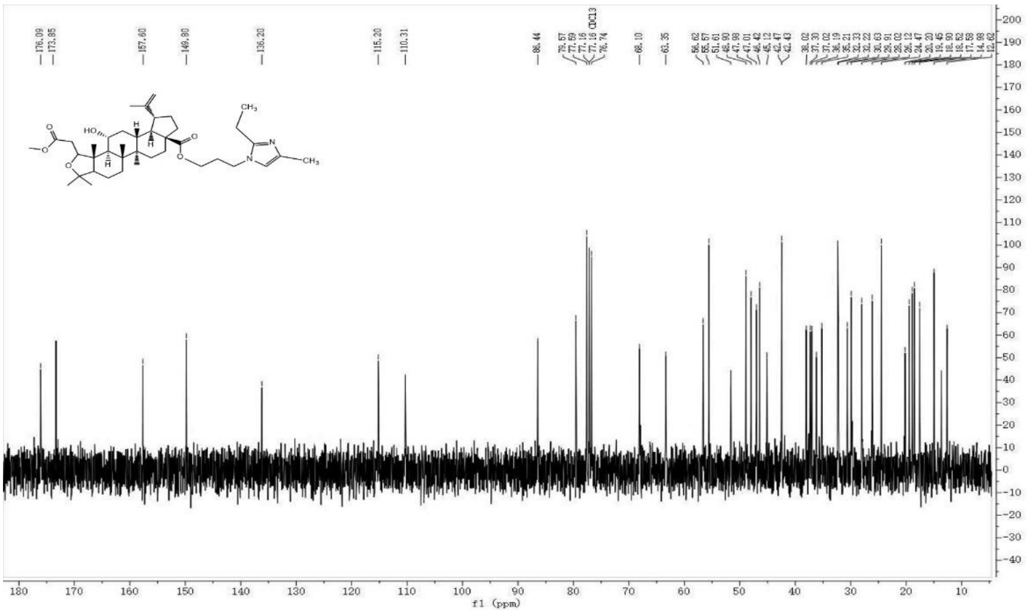

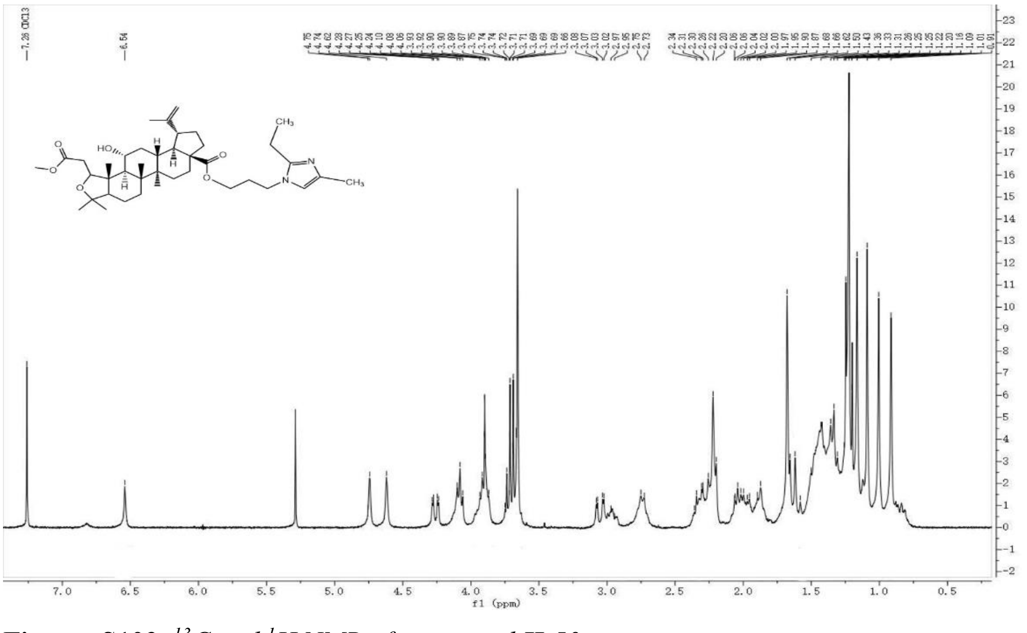

Figures S103. *^{13}C and ^{1}H NMR of compound* ***II-50***.

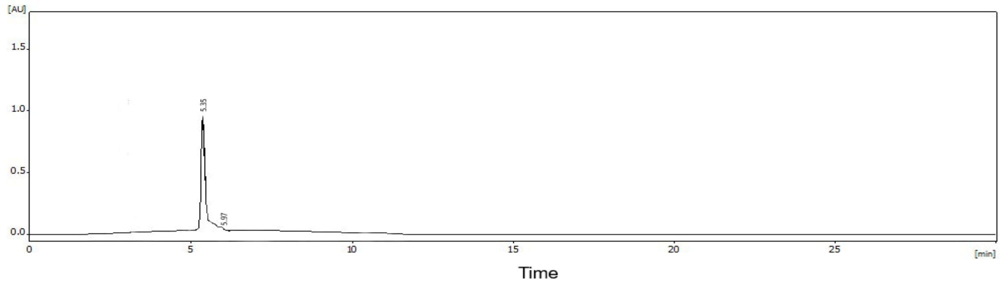

	RT	Area	Area %	Height
1	5.348	9290.031	96.8	934.310
2	5.967	311.532	3.2	33.207

Time	A (water,0.5% Phosphoric acid)	B (Acetonitrile)
0	65	35
10	10	90
30	10	90
40	65	35

Figure S104. *HPLC tracing of compound **II-51**.*

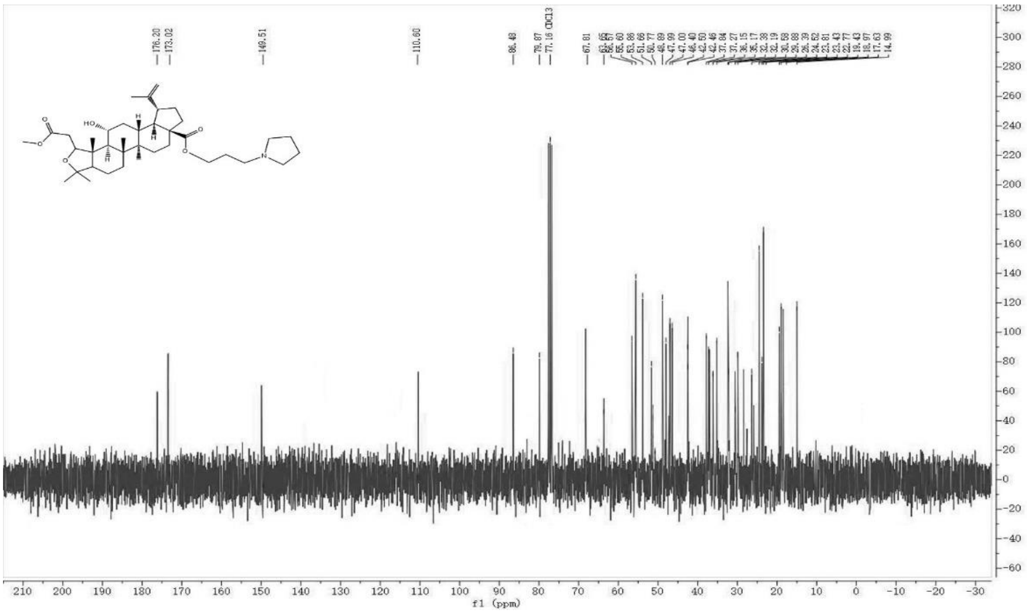

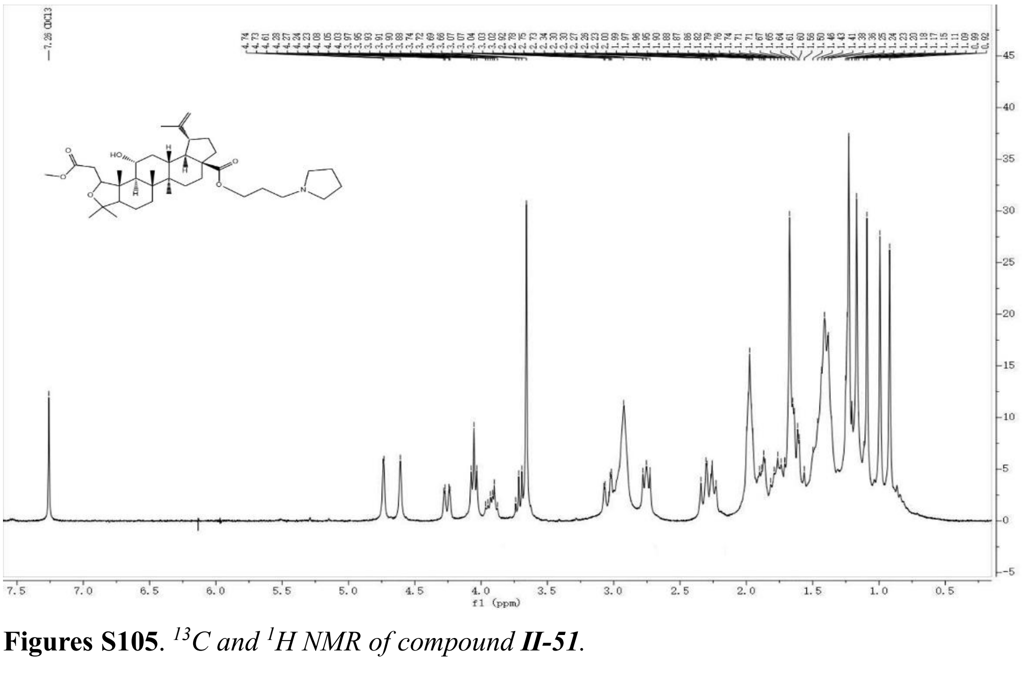

Figures S105. *^{13}C and ^{1}H NMR of compound **II-51**.*

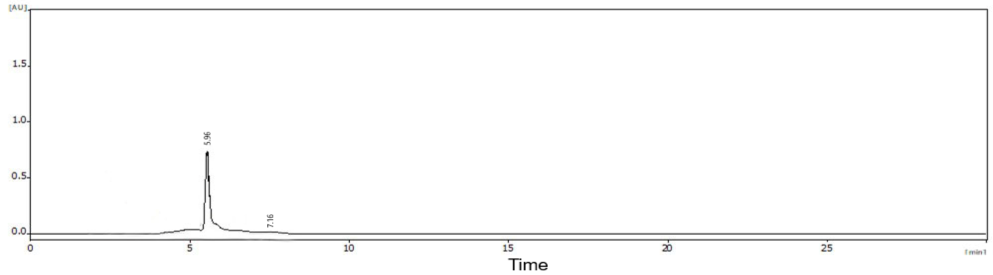

	RT	Area	Area %	Height
2	5.964	7325.767	98.1	709.166
3	7.164	141.885	1.9	9.004

Time	A (water,0.5% Phosphoric acid)	B (Acetonitrile)
0	65	35
10	10	90
30	10	90
40	65	35

Figure S106. *HPLC tracing of compound **II-52**.*

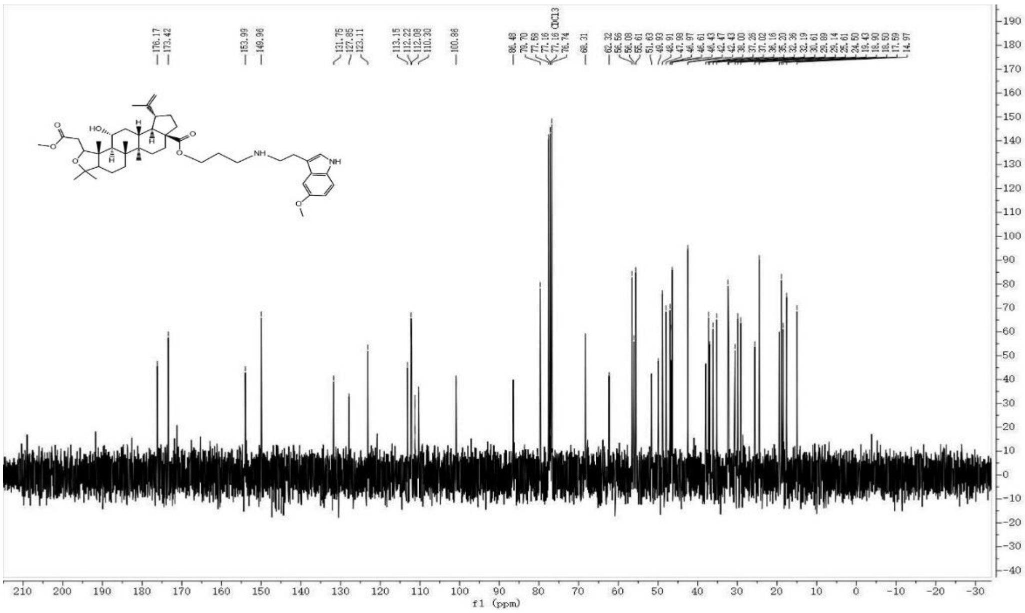

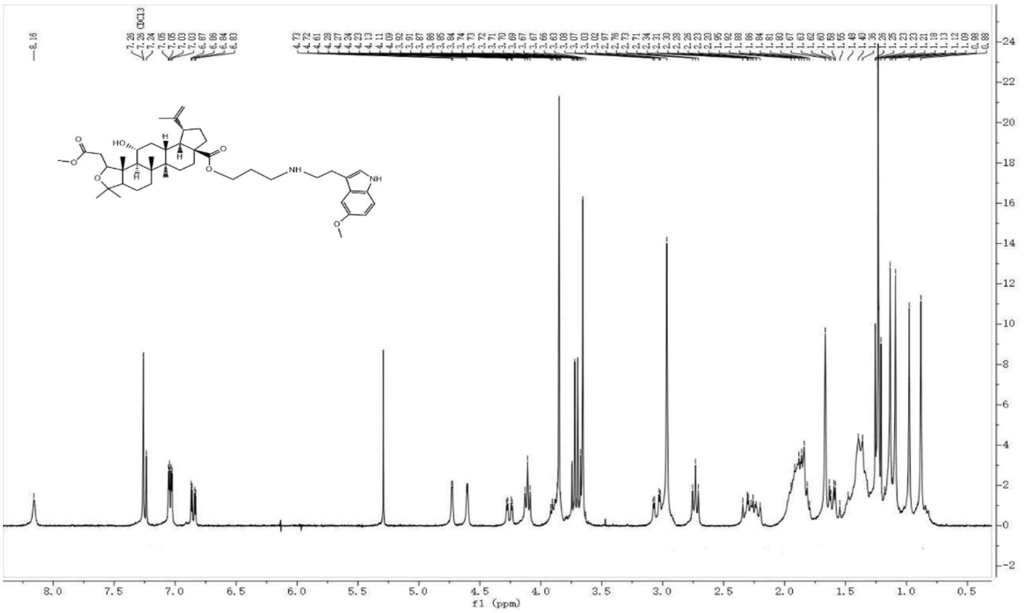

Figures S107. *^{13}C and ^{1}H NMR of compound **II-52**.*

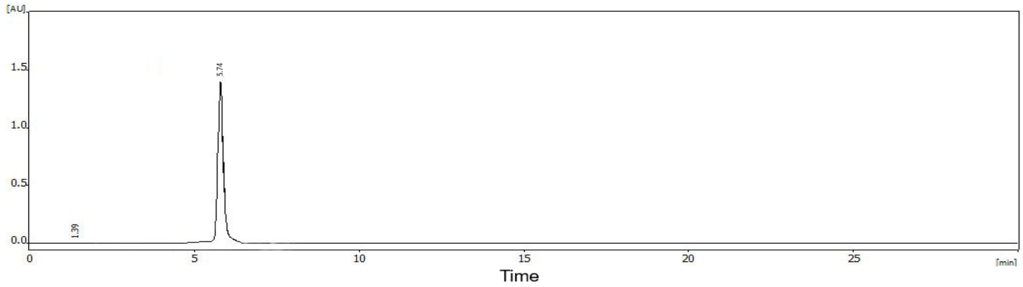

	RT	Area	Area %	Height
1	1.390	519.250	2.5	0.933
2	5.743	20250.761	97.5	1415.675

Time	A (water,0.5% Phosphoric acid)	B (Acetonitrile)
0	65	35
10	10	90
30	10	90
40	65	35

Figure S108. *HPLC tracing of compound* ***II-53***.

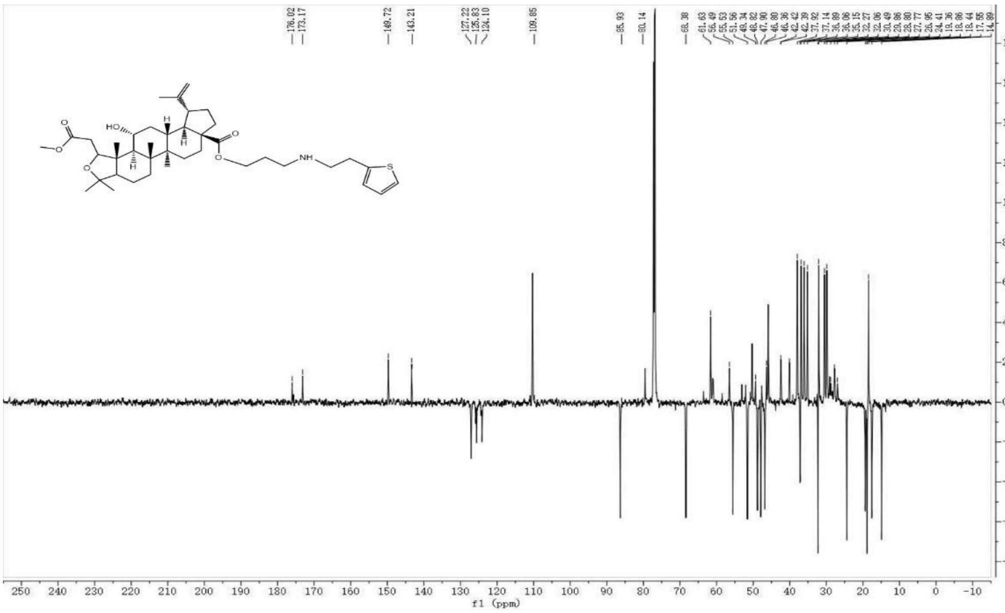

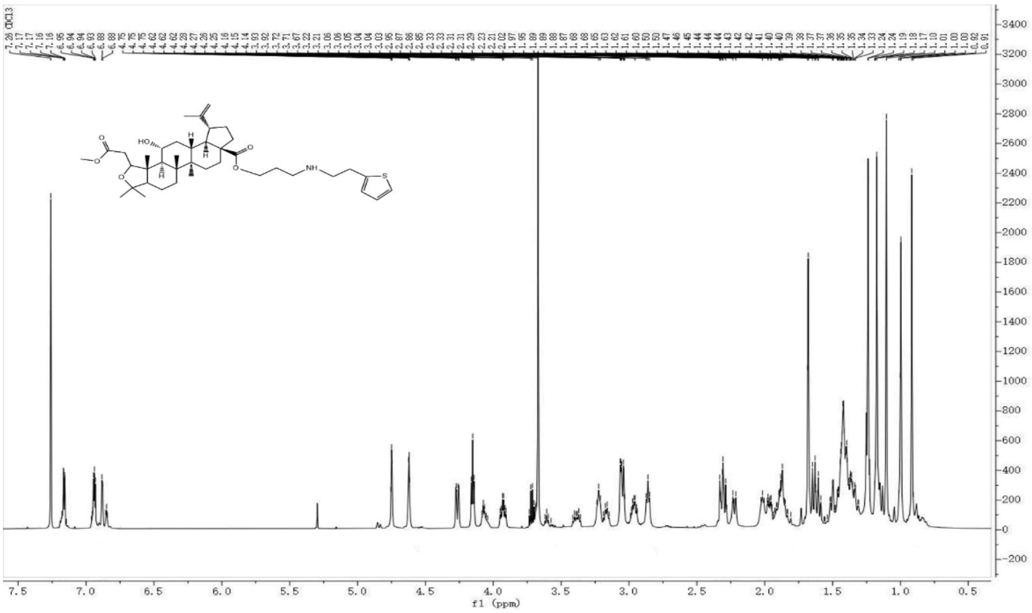

Figures S109. *^{13}C and ^{1}H NMR of compound* ***II-53***.

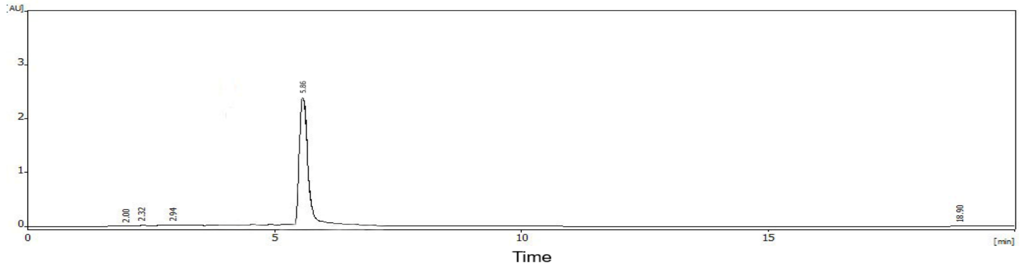

	RT	Area	Area %	Height
1	2.003	87.715	0.3	3.523
2	2.323	247.844	0.8	29.482
3	2.937	1187.534	3.7	22.003
4	5.864	30840.989	95.1	2376.103
5	18.900	67.310	0.2	1.359

Time	A (water,0.5% Phosphoric acid)	B (Acetonitrile)
0	65	35
10	10	90
30	10	90
40	65	35

Figure S110. *HPLC tracing of compound **II-54**.*

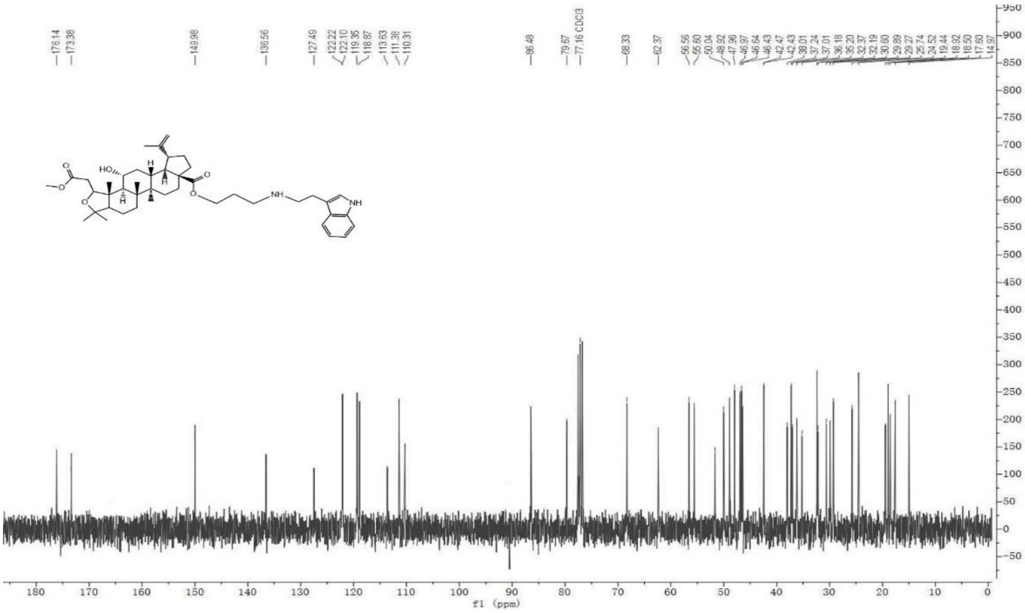

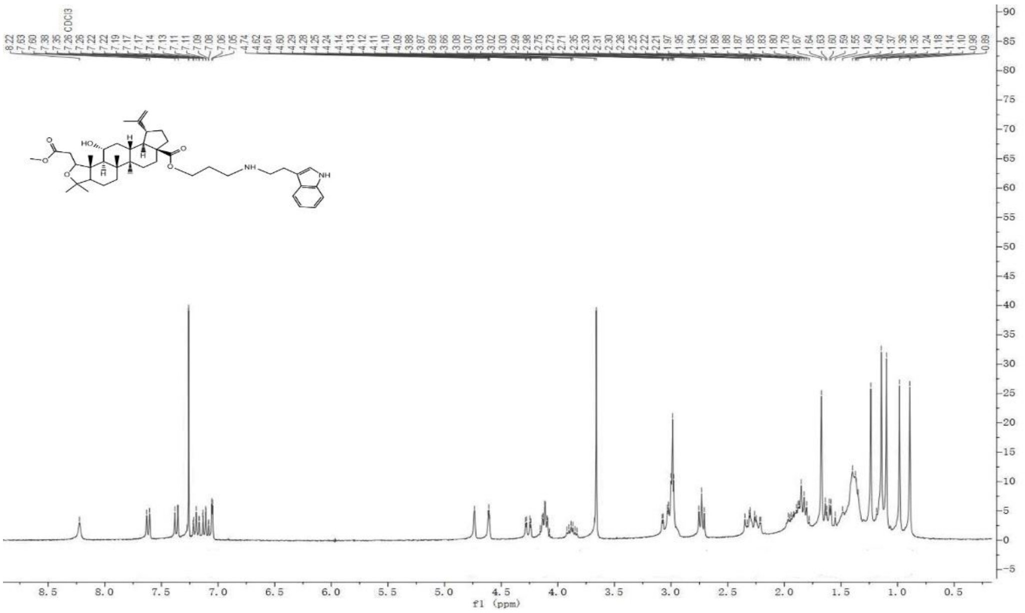

Figures S111. *^{13}C and ^{1}H NMR of compound **II-54.***

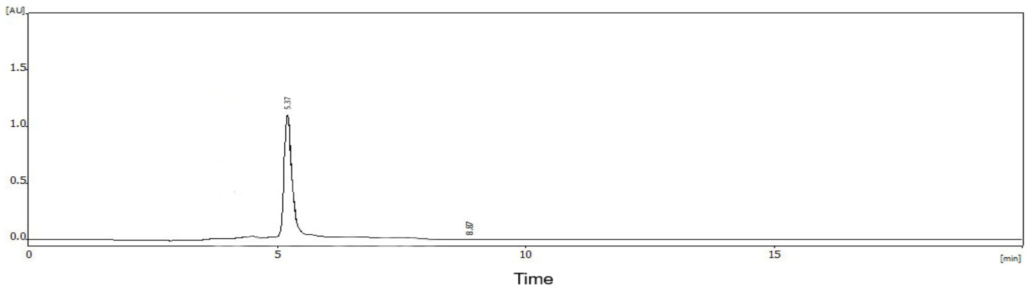

	RT	Area	Area %	Height
1	5.374	14797.941	99.8	1094.236
2	8.867	28.995	0.2	1.184

Time	A (water,0.5% Phosphoric acid)	B (Acetonitrile)
0	65	35
10	10	90
30	10	90
40	65	35

Figure S112. *HPLC tracing of compound **II-55**.*

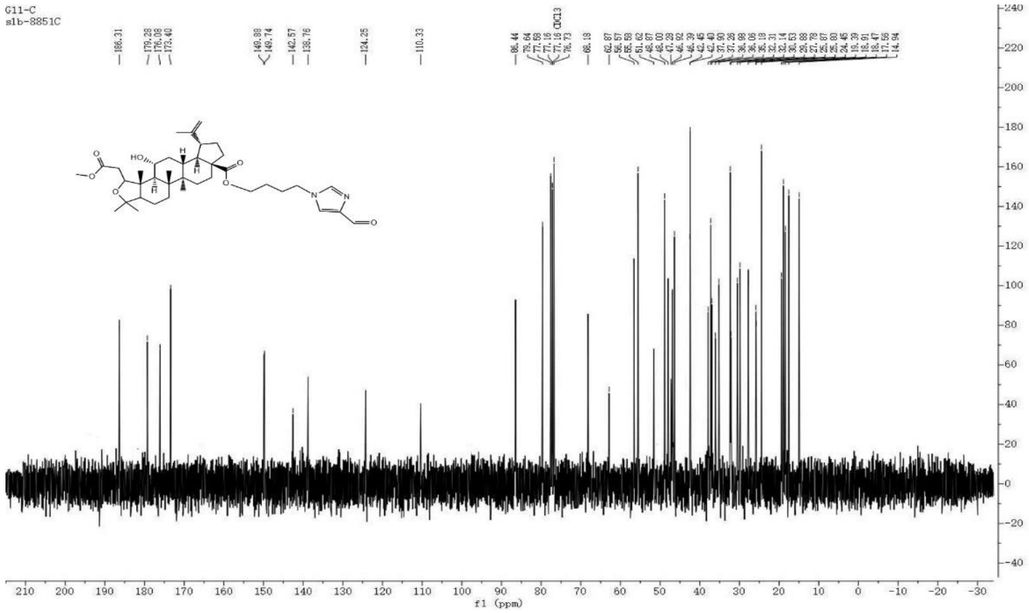

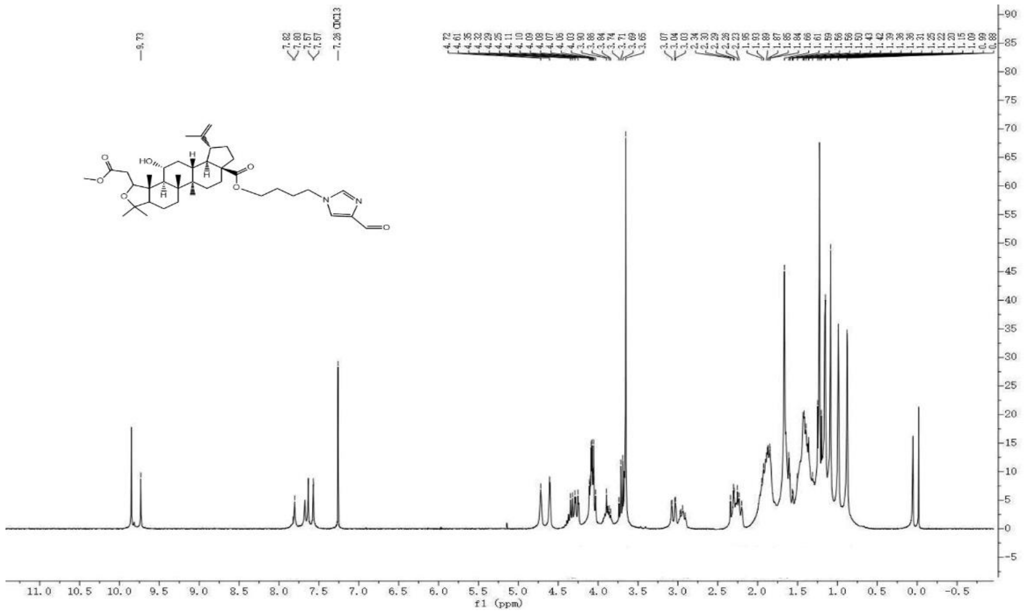

Figures S113. *^{13}C and ^{1}H NMR of compound **II-55**.*

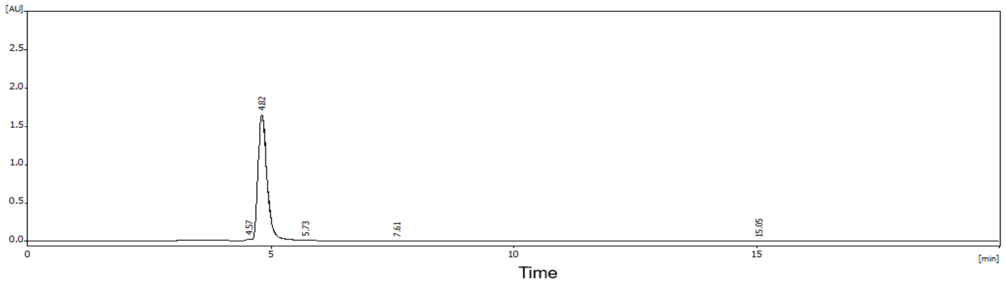

	RT	Area	Area %	Height
1	4.567	166.003	0.7	22.514
2	4.820	21619.247	97.0	1646.572
3	5.727	299.606	1.3	13.854
4	7.613	167.559	0.8	2.135
5	15.053	44.575	0.2	0.660

Time	A (water,0.5% Phosphoric acid)	B (Acetonitrile)
0	65	35
10	10	90
30	10	90
40	65	35

Figure S114. *HPLC tracing of compound* ***II-56****.*

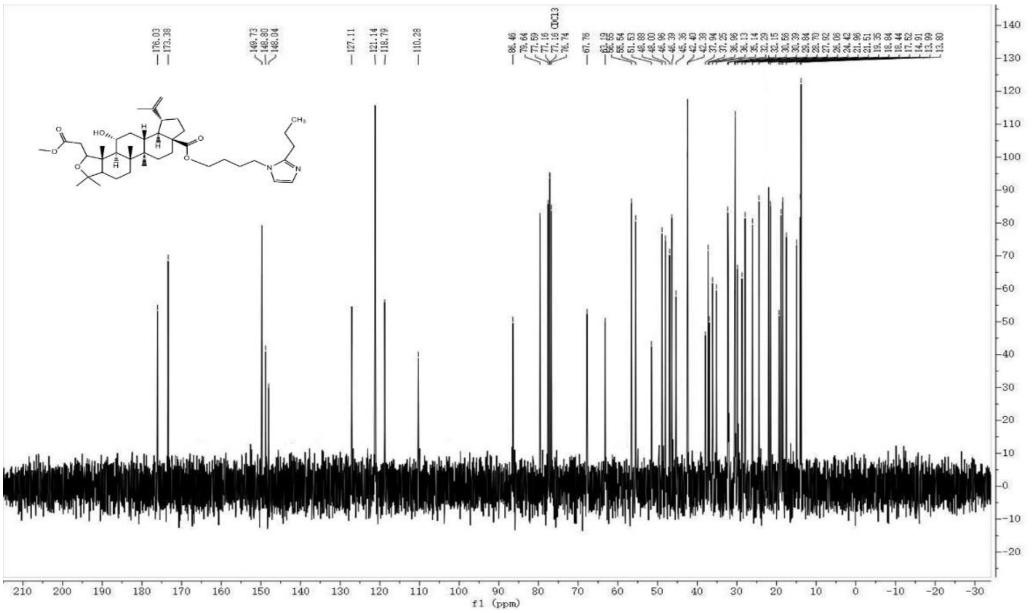

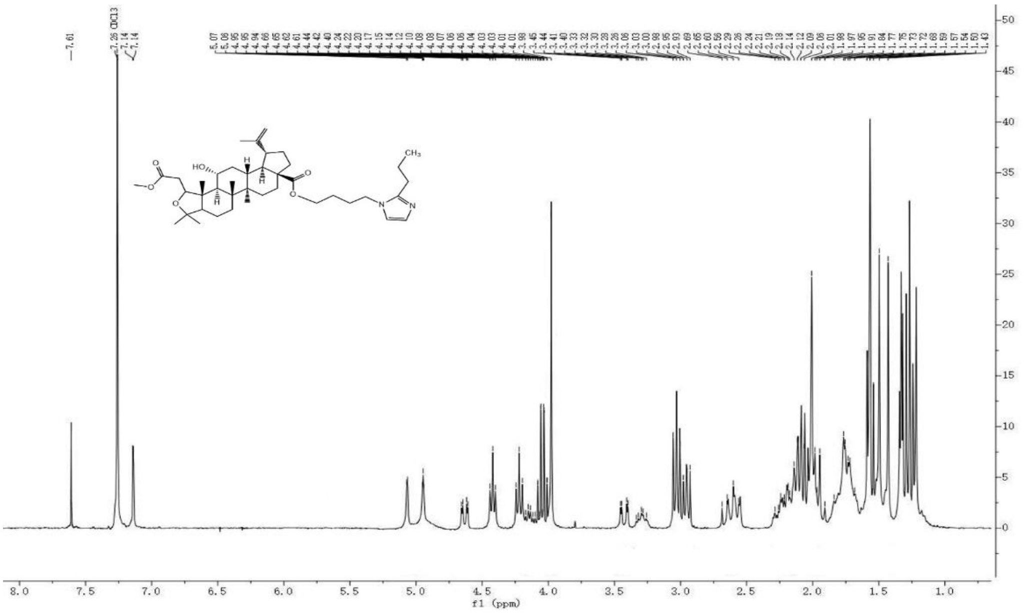

Figures S115. *^{13}C and ^{1}H NMR of compound* ***II-56****.*

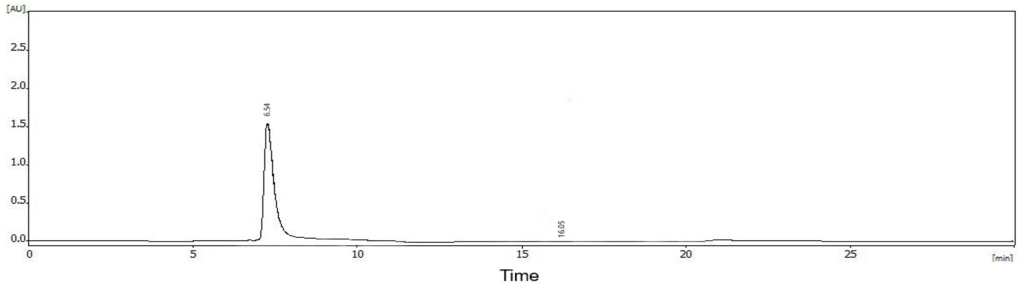

	RT	Area	Area %	Height
1	6.541	37801.810	99.7	1534.261
2	16.047	126.390	0.3	6.351

Time	A (water,0.5% Phosphoric acid)	B (Acetonitrile)
0	65	35
10	10	90
30	10	90
40	65	35

Figure S116. *HPLC tracing of compound **II-57**.*

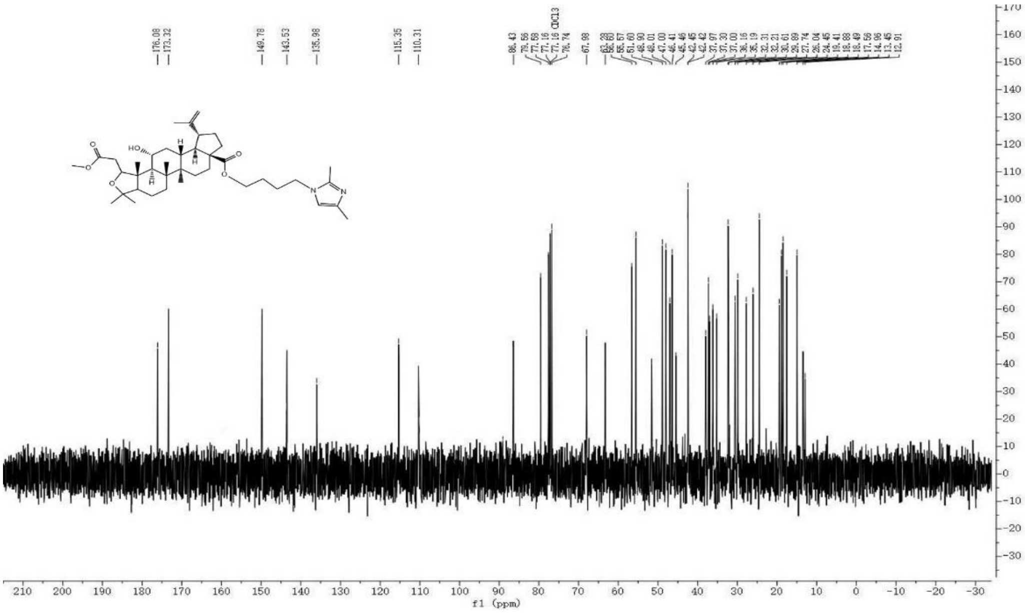

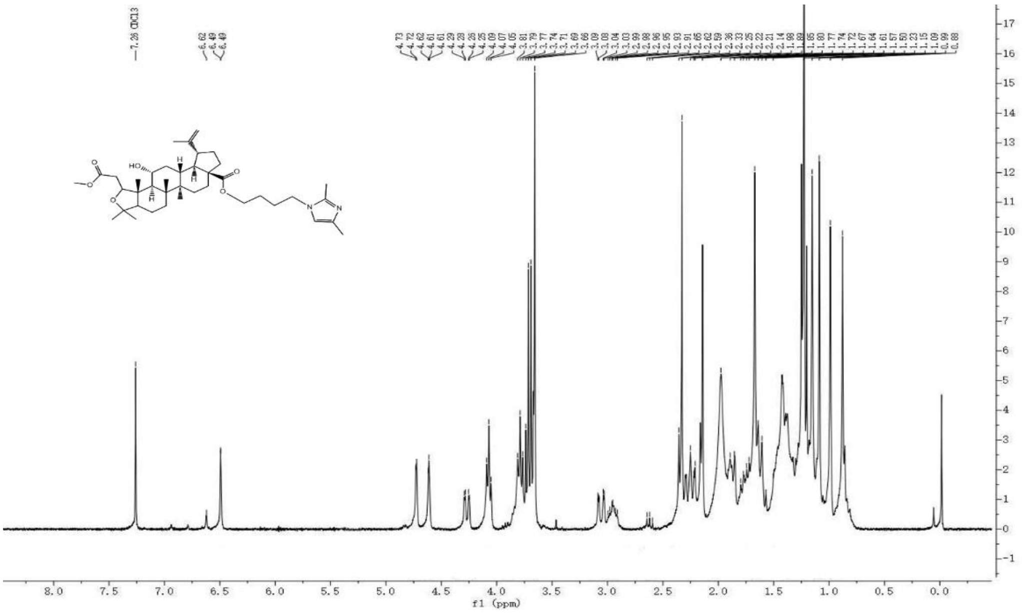

Figures S117. *^{13}C and ^{1}H NMR of compound **II-57**.*

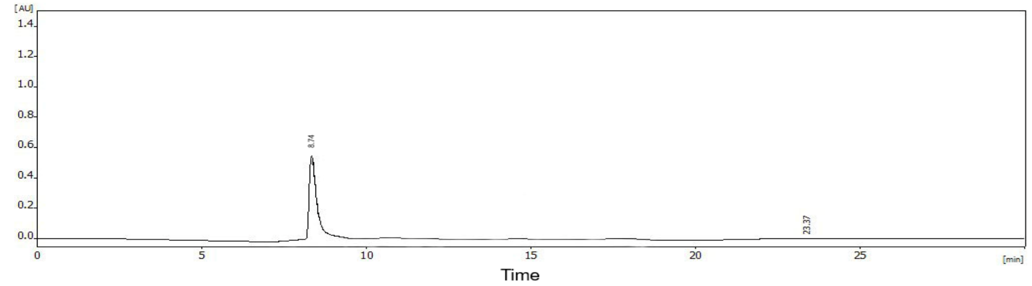

	RT	Area	Area %	Height
1	8.743	21275.004	95.2	603.337
2	23.373	1069.901	4.8	5.828

Time	A (water,0.5% Phosphoric acid)	B (Acetonitrile)
0	65	35
10	10	90
30	10	90
40	65	35

Figure S118. *HPLC tracing of compound **II-58**.*

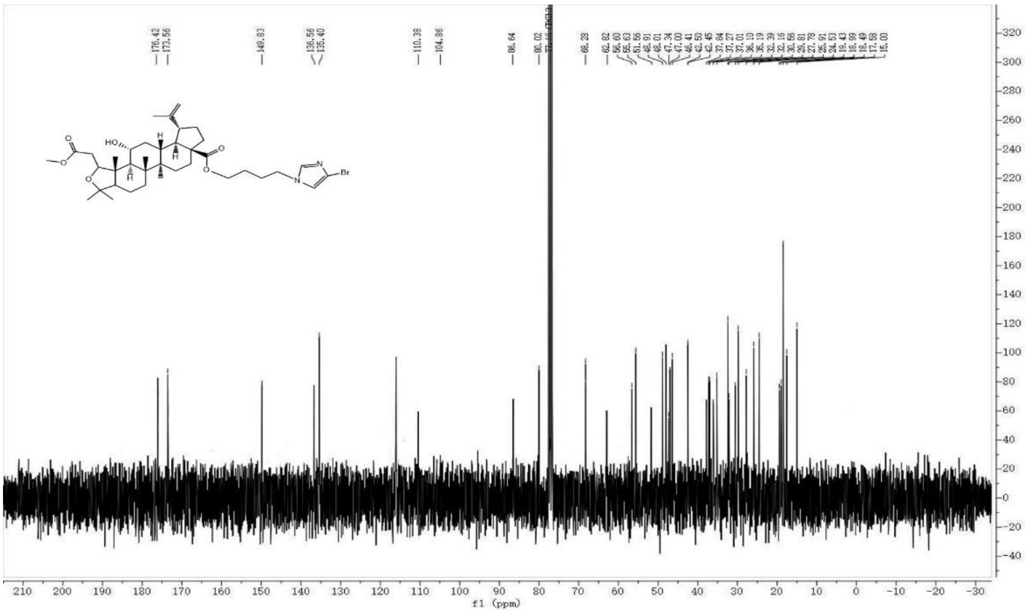

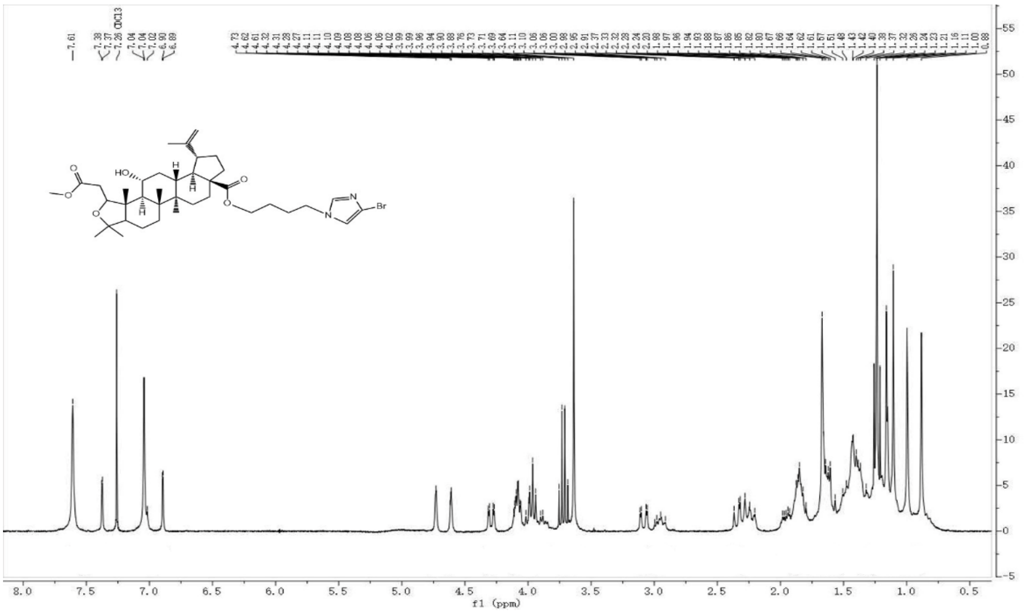

Figures S119. *^{13}C and ^{1}H NMR of compound **II-58**.*

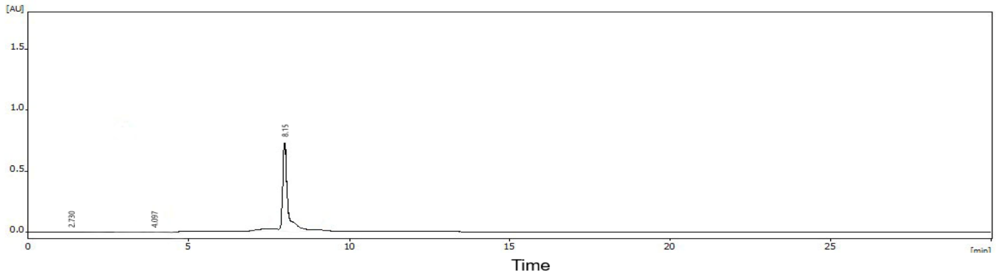

	RT	Area	Area %	Height
1	2.730	29.597	0.4	3.853
2	4.047	110.383	1.5	9.004
3	8.149	7325.767	98.1	709.166

Time	A (water,0.5% Phosphoric acid)	B (Acetonitrile)
0	65	35
10	10	90
30	10	90
40	65	35

Figure S120. *HPLC tracing of compound **II-59**.*

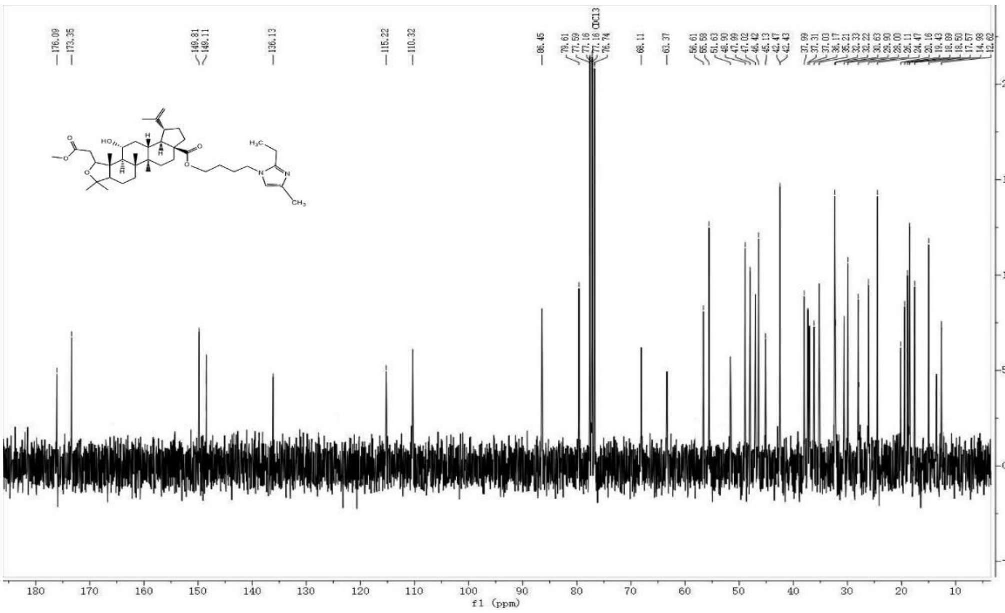

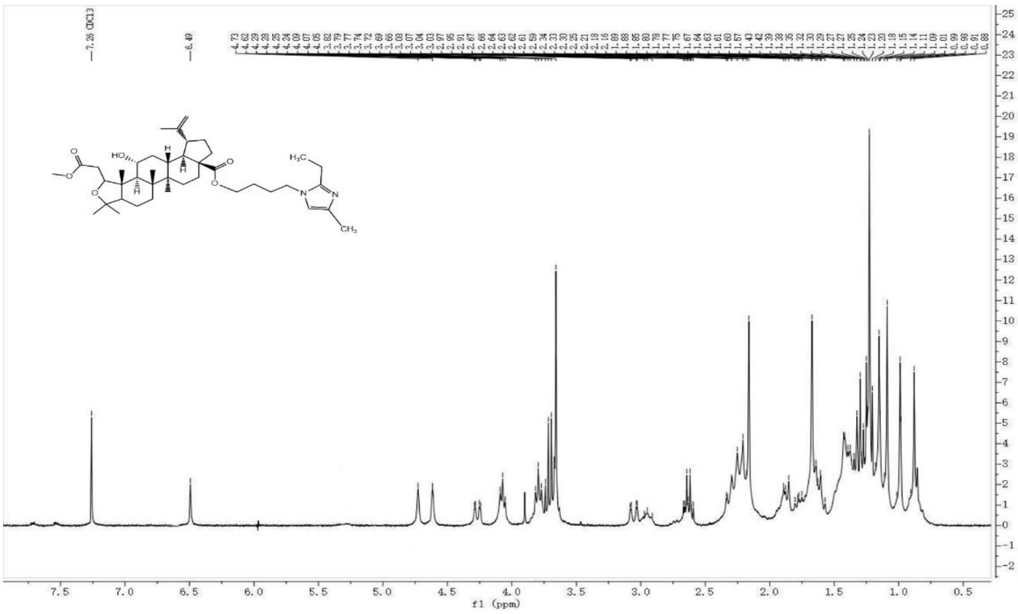

Figures S121. *^{13}C and ^{1}H NMR of compound **II-59**.*

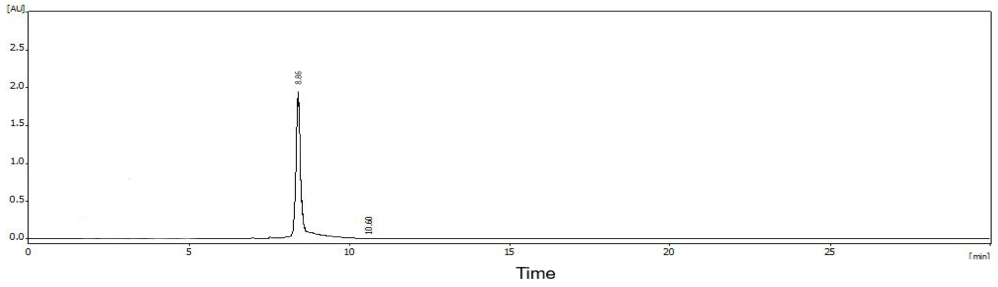

	RT	Area	Area %	Height
4	8.862	24520.988	95.5	1944.114
5	10.600	1155.439	4.5	0.969

Time	A (water,0.5% Phosphoric acid)	B (Acetonitrile)
0	65	35
10	10	90
30	10	90
40	65	35

Figure S122. *HPLC tracing of compound **II-60**.*

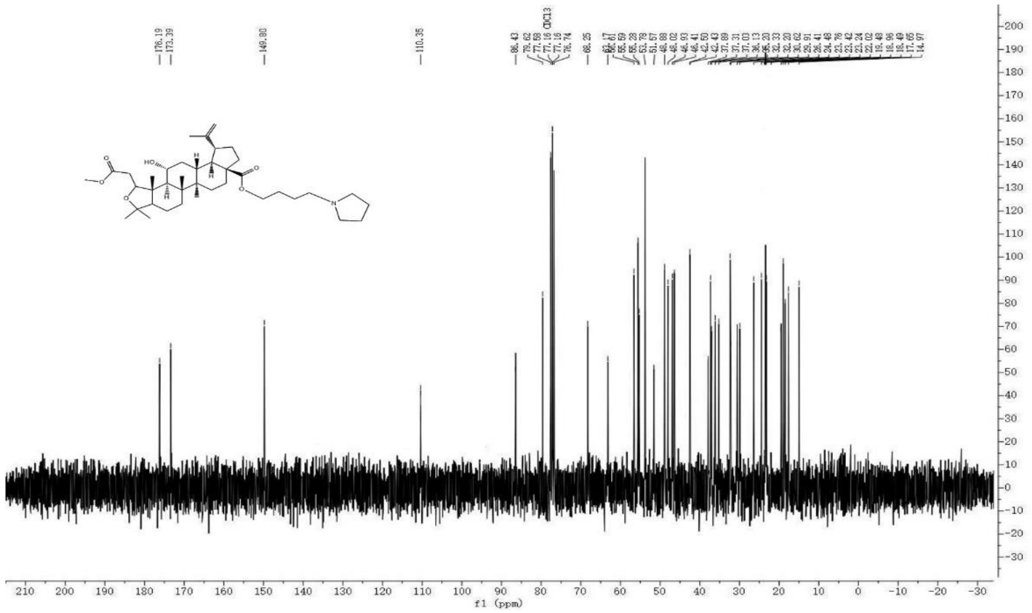

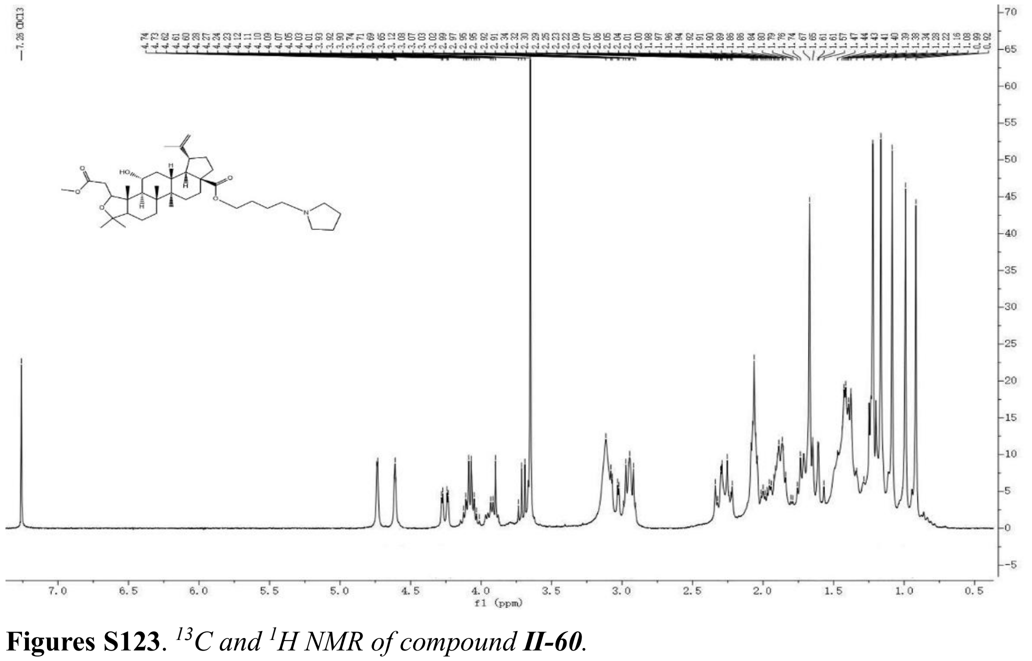

Figures S123. *^{13}C and ^{1}H NMR of compound **II-60**.*

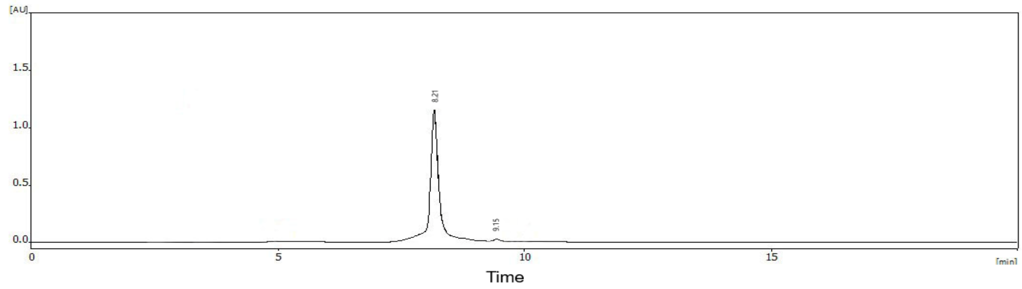

	RT	Area	Area %	Height
1	8.206	15671.441	95.3	1159.080
2	9.146	772.883	4.7	26.250

Time	A (water,0.5% Phosphoric acid)	B (Acetonitrile)
0	65	35
10	10	90
30	10	90
40	65	35

Figure S124. *HPLC tracing of compound **II-61**.*

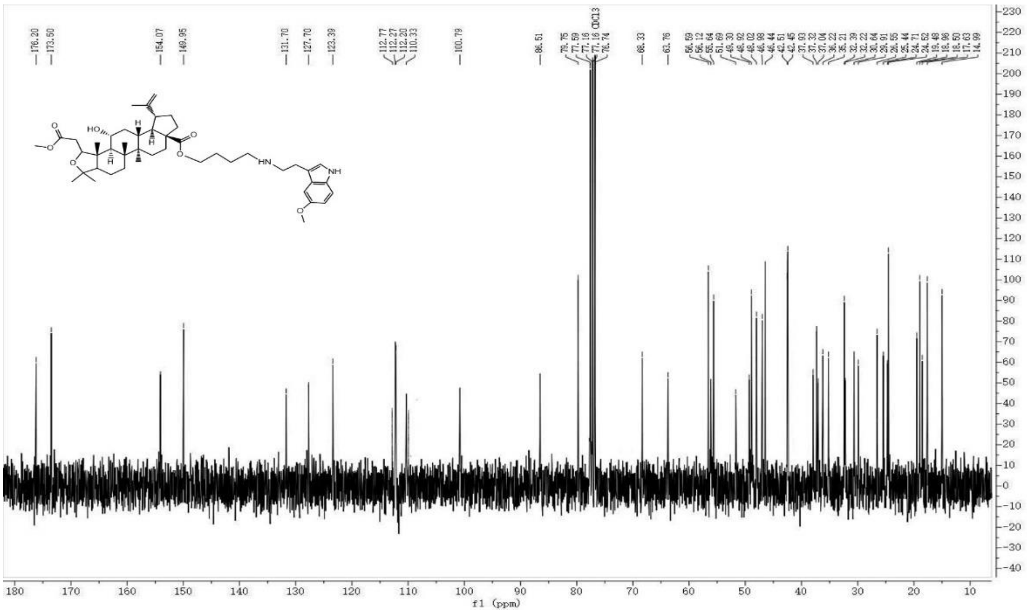

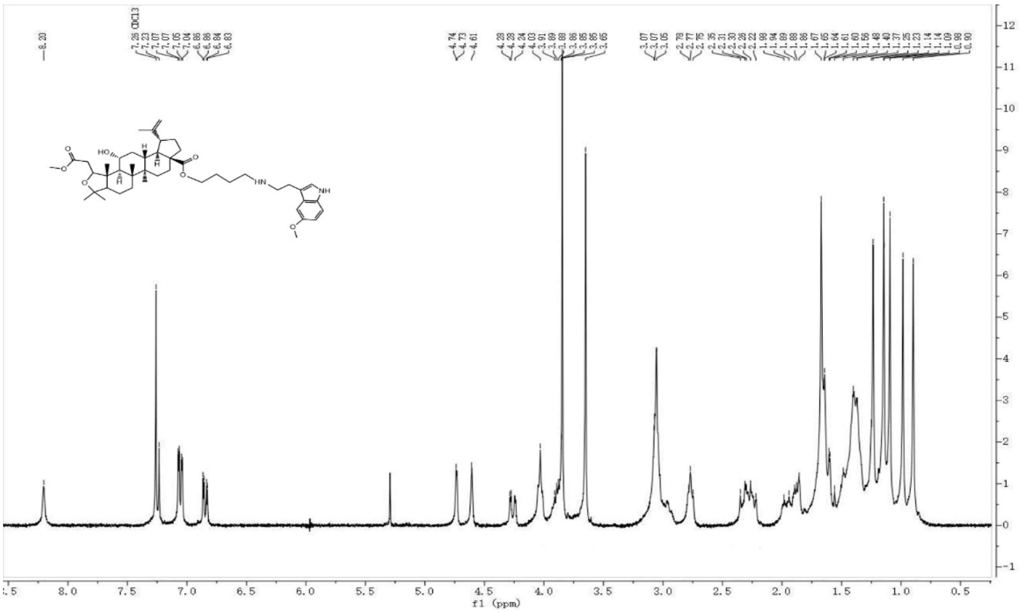

Figures S125. *^{13}C and ^{1}H NMR of compound **II-61**.*

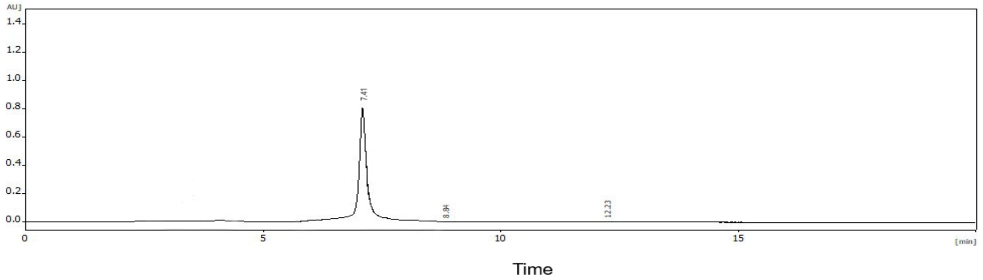

	RT	Area	Area %	Height
1	7.413	11038.300	99.8	799.926
2	8.843	8.078	0.1	0.460
3	12.230	15.819	0.1	0.399

Time	A (water,0.5% Phosphoric acid)	B (Acetonitrile)
0	65	35
10	10	90
30	10	90
40	65	35

Figure S126. *HPLC tracing of compound **II-62**.*

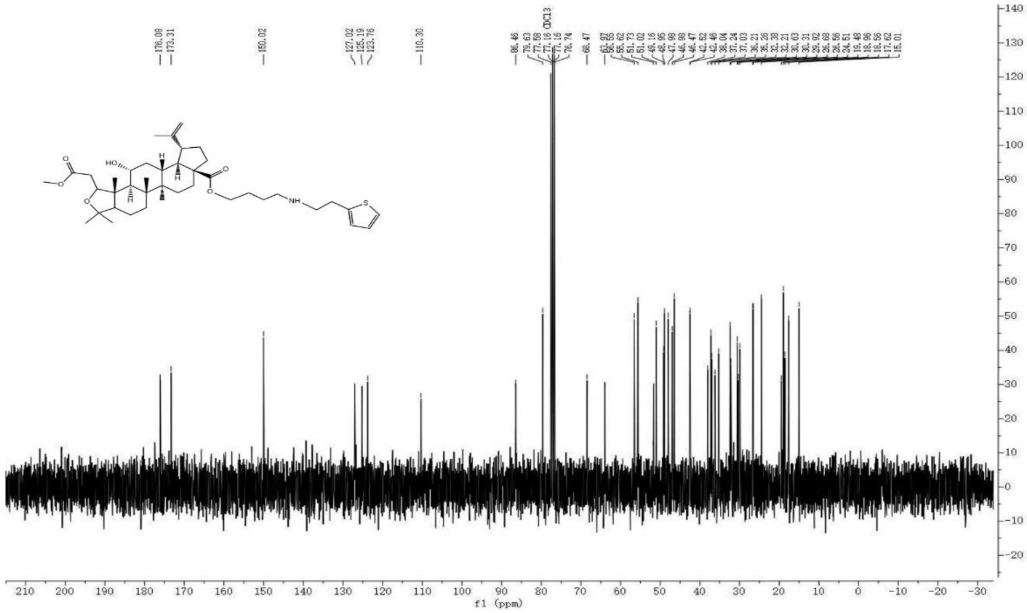

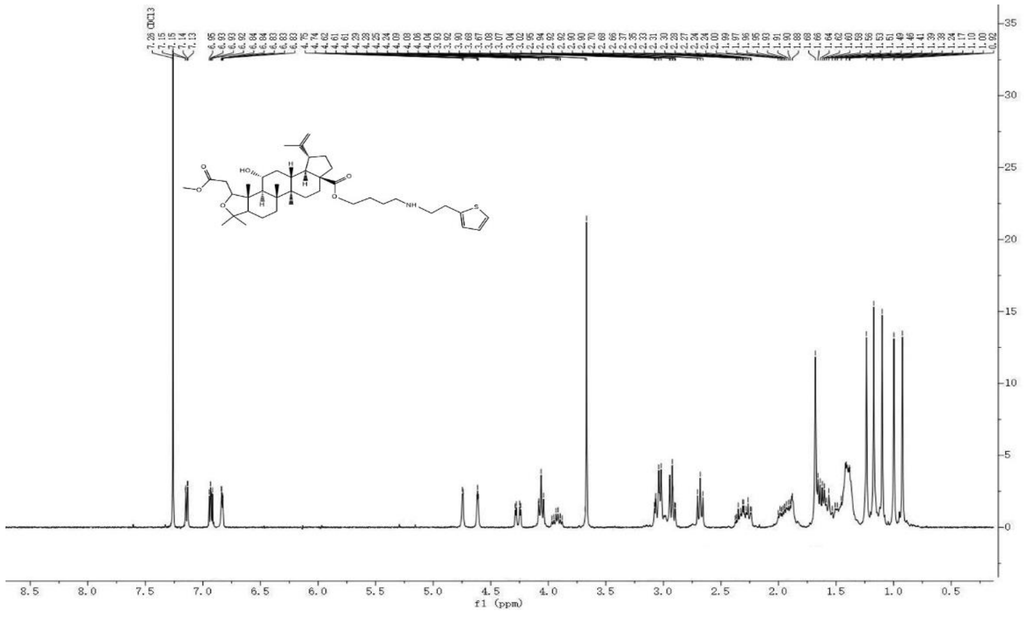

Figures S127. *^{13}C and ^{1}H NMR of compound **II-62**.*

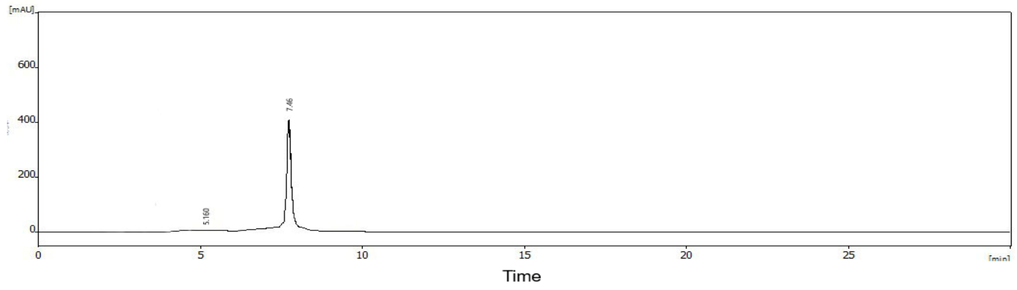

	RT	Area	Area %	Height
1	7.463	6321.676	95.0	411.071
2	5.160	333.736	5.0	4.192

Time	A (water,0.5% Phosphoric acid)	B (Acetonitrile)
0	65	35
10	10	90
30	10	90
40	65	35

Figure S128. *HPLC tracing of compound **II-63**.*

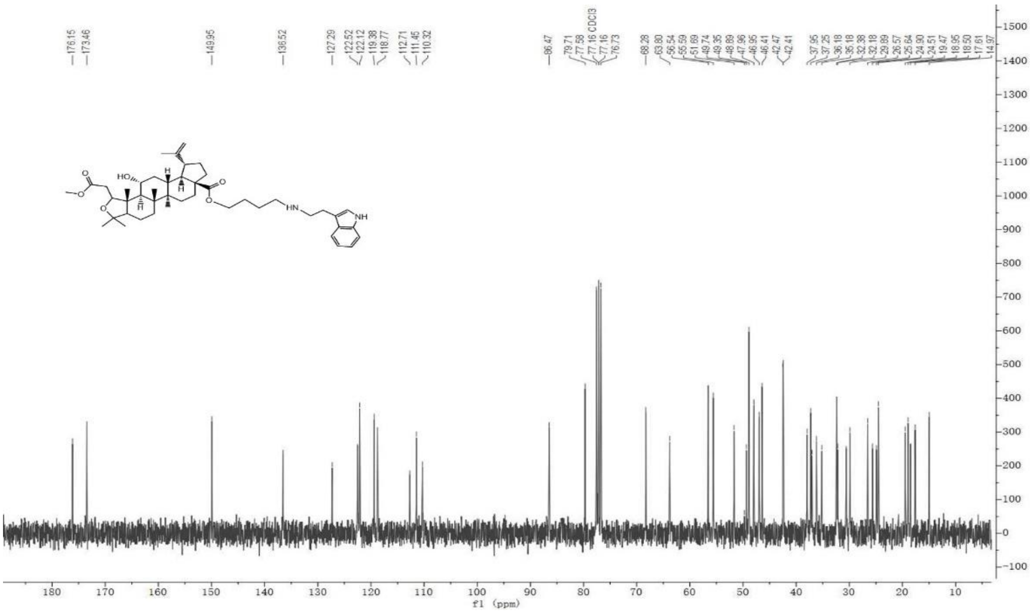

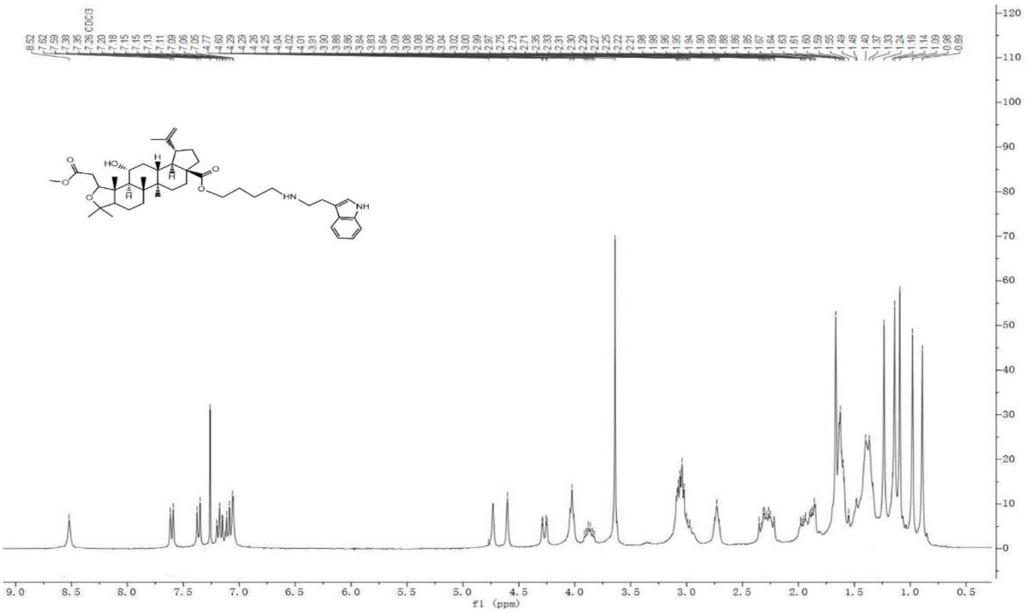

Figures S129. *^{13}C and ^{1}H NMR of compound **II-63**.*

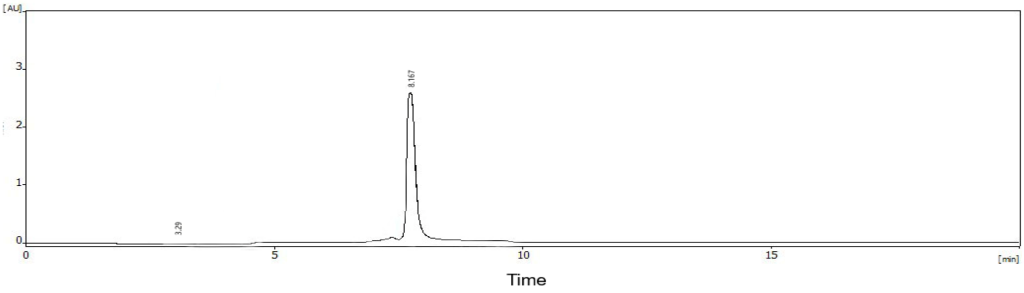

	RT	Area	Area %	Height
1	3.290	1687.313	4.5	85.625
2	8.167	35723.102	95.5	2577.913

Time	A (water,0.5% Phosphoric acid)	B (Acetonitrile)
0	65	35
10	10	90
30	10	90
40	65	35

Figure S130. *HPLC tracing of compound* ***II-64****.*

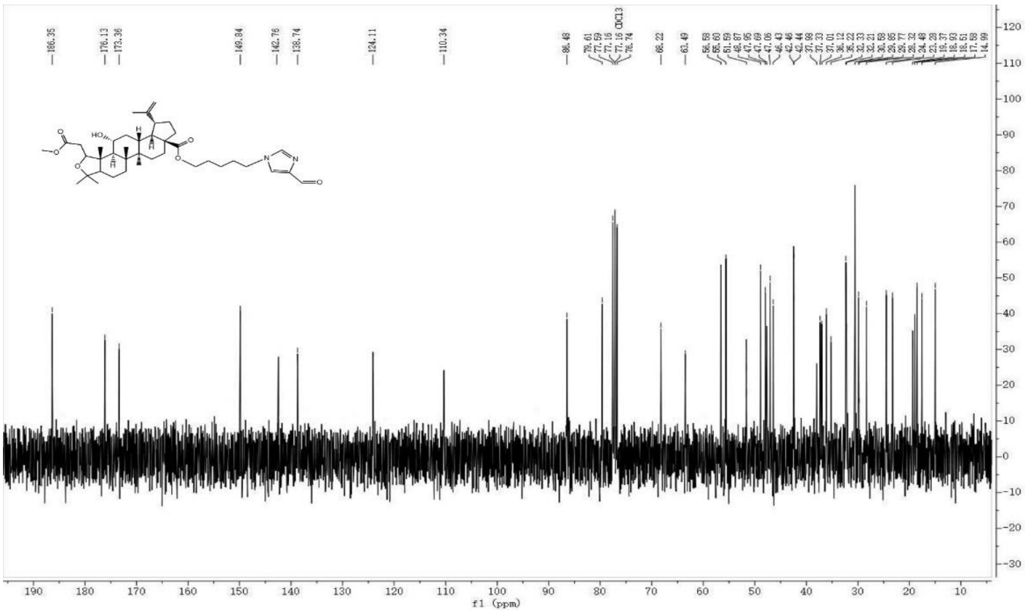

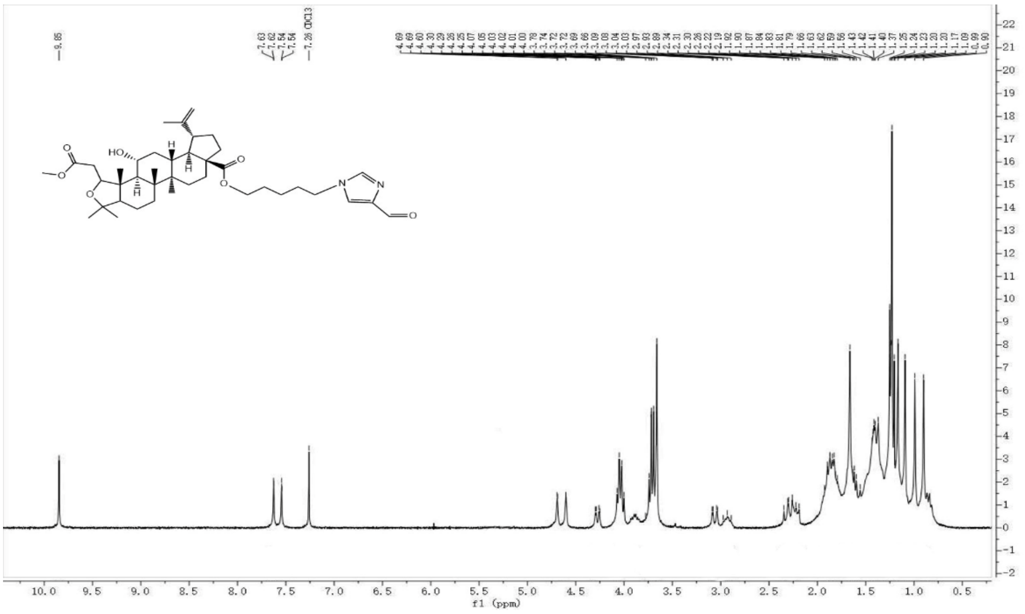

Figures S131. *^{13}C and ^{1}H NMR of compound* ***II-64****.*

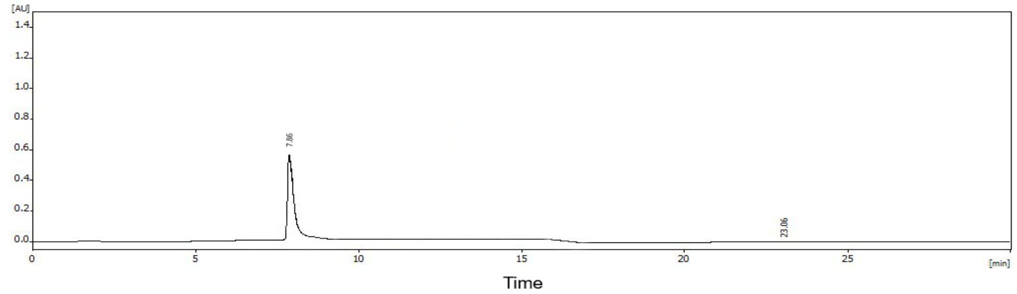

	RT	Area	Area %	Height
1	7.864	21814.415	95.4	589.881
2	23.063	1063.418	4.6	5.039

Time	A (water,0.5% Phosphoric acid)	B (Acetonitrile)
0	65	35
10	10	90
30	10	90
40	65	35

Figure S132. *HPLC tracing of compound **II-65**.*

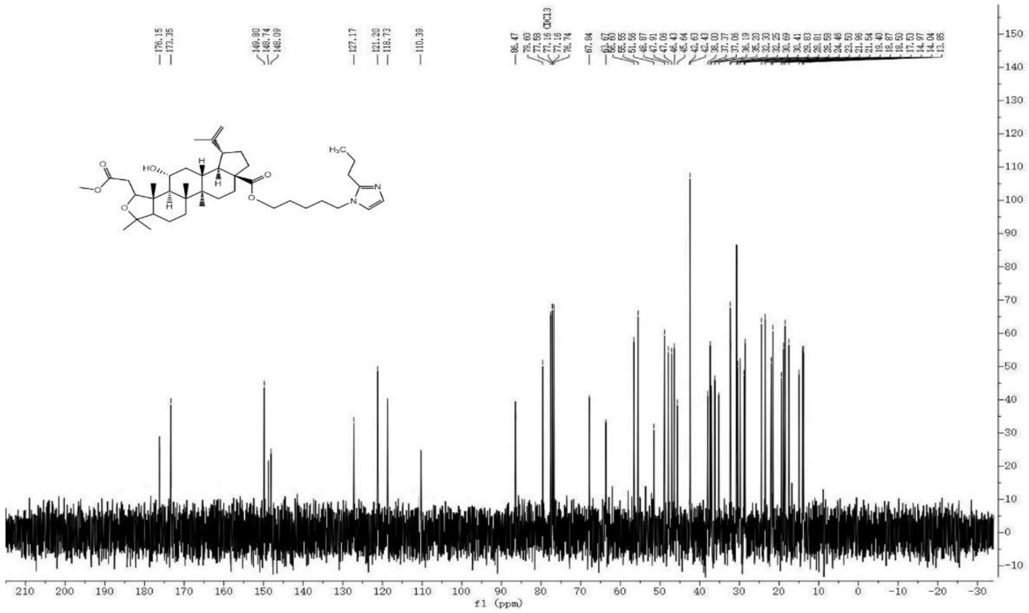

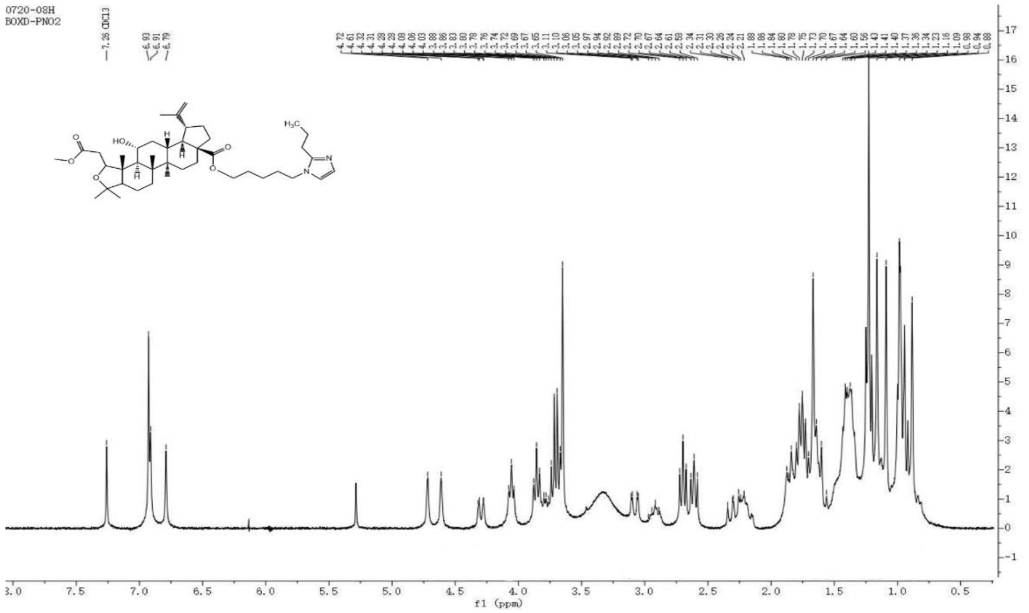

Figures S133. *^{13}C and ^{1}H NMR of compound **II-65**.*

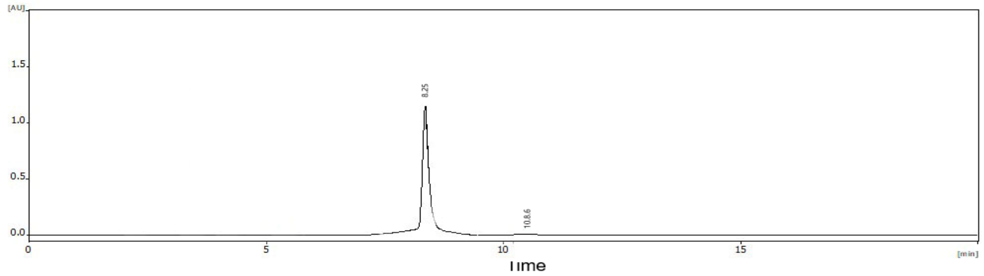

	RT	Area	Area %	Height
1	8.246	13727.831	96.4	1146.022
2	10.864	506.257	3.6	5.082

Time	A (water,0.5% Phosphoric acid)	B (Acetonitrile)
0	65	35
10	10	90
30	10	90
40	65	35

Figure S134. *HPLC tracing of compound **II-66**.*

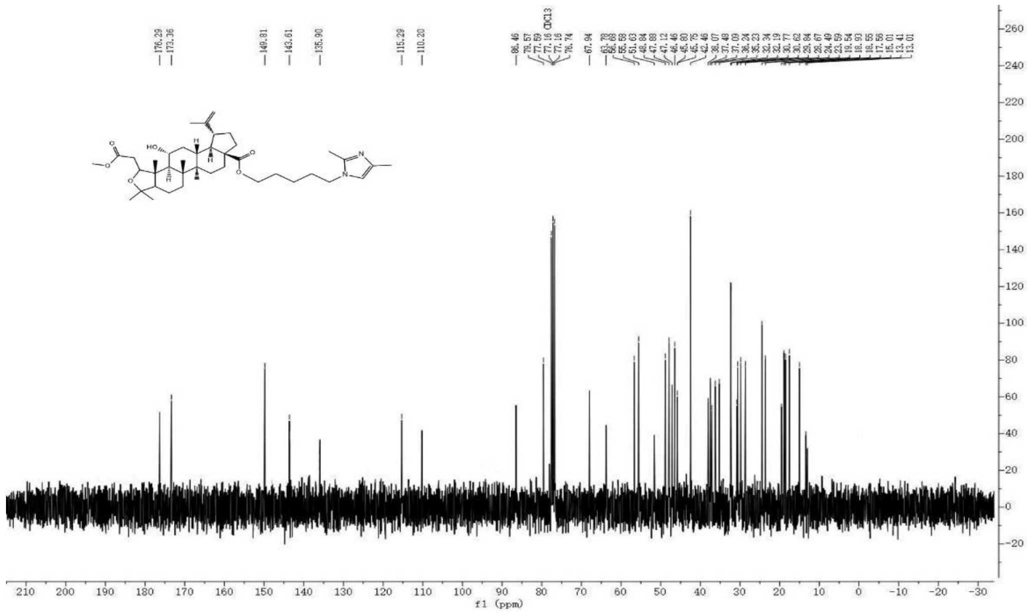

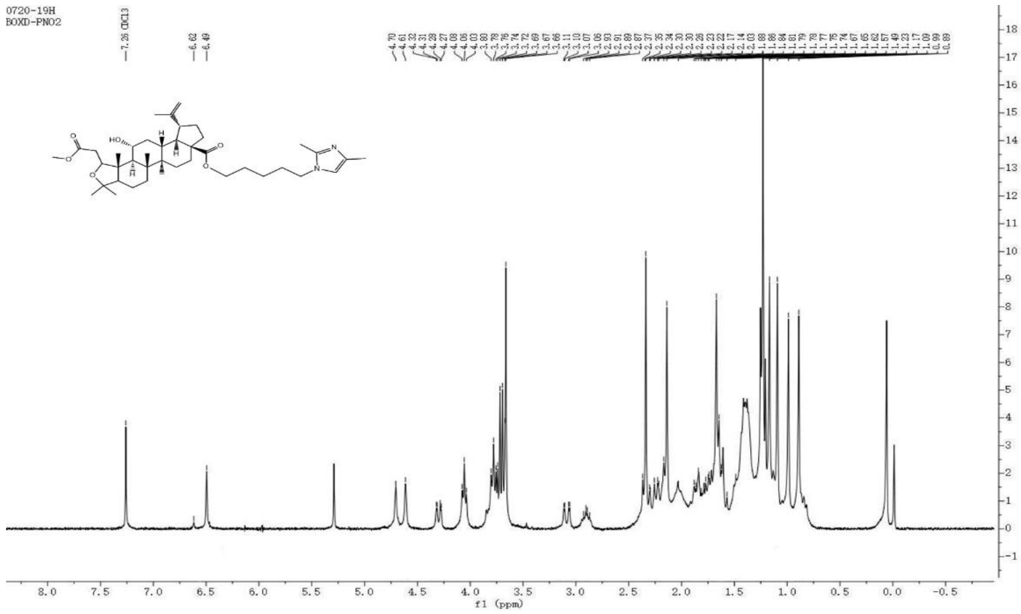

Figures S135. *^{13}C and ^{1}H NMR of compound **II-66**.*

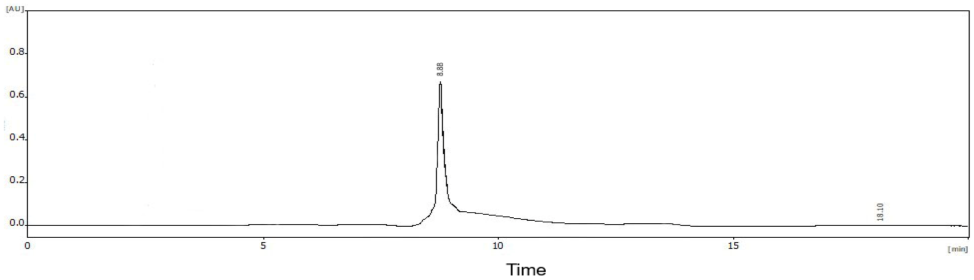

	RT	Area	Area %	Height
1	8.879	13482.359	98.9	674.692
2	18.103	144.336	1.1	0.891

Time	A (water,0.5% Phosphoric acid)	B (Acetonitrile)
0	65	35
10	10	90
30	10	90
40	65	35

Figure S136. *HPLC tracing of compound **II-67**.*

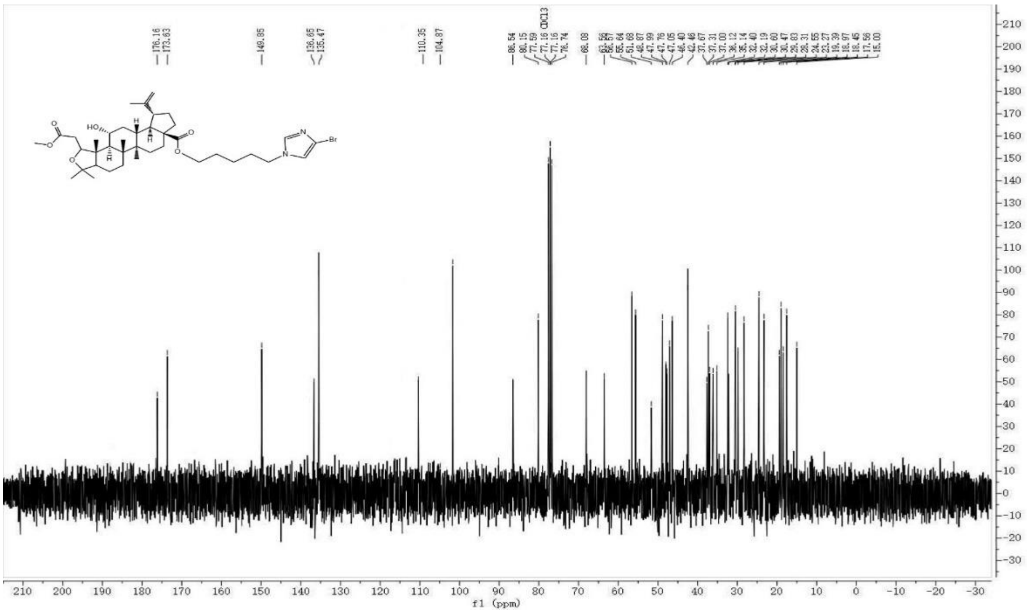

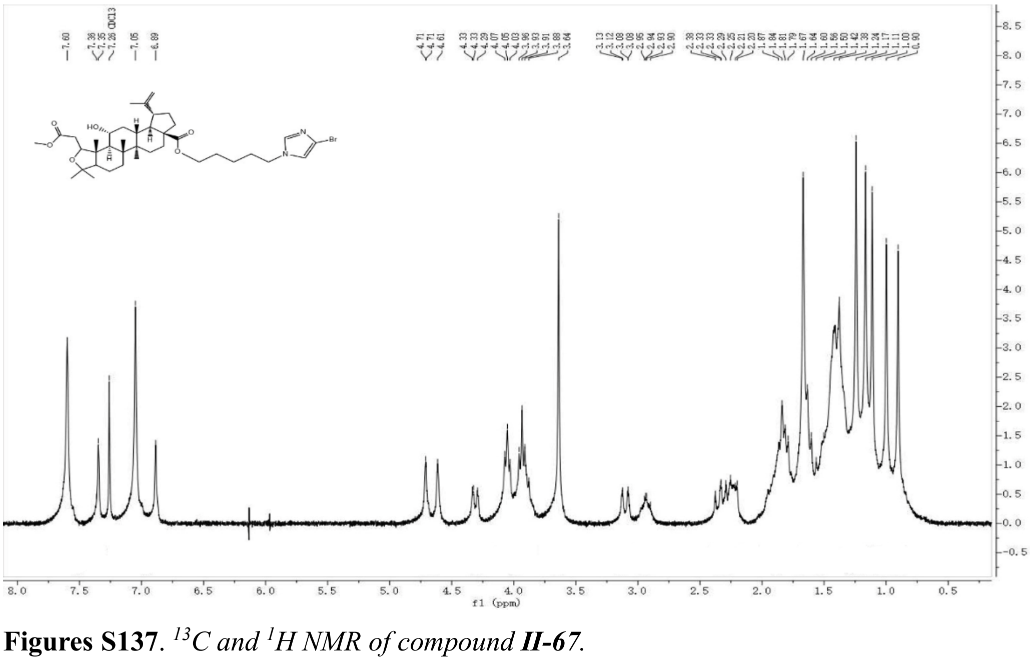

Figures S137. *^{13}C and ^{1}H NMR of compound **II-67**.*

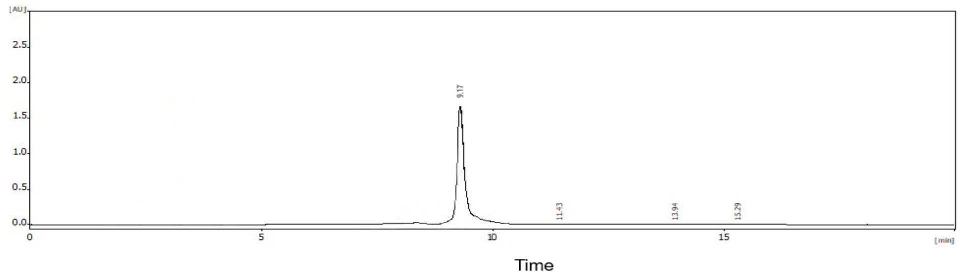

	RT	Area	Area %	Height
1	9.167	26988.746	98.9	1400.417
2	11.430	54.578	0.2	0.584
3	13.937	50.400	0.2	0.959
4	15.290	191.022	0.7	2.664

Time	A (water,0.5% Phosphoric acid)	B (Acetonitrile)
0	65	35
10	10	90
30	10	90
40	65	35

Figure S138. *HPLC tracing of compound **II-68**.*

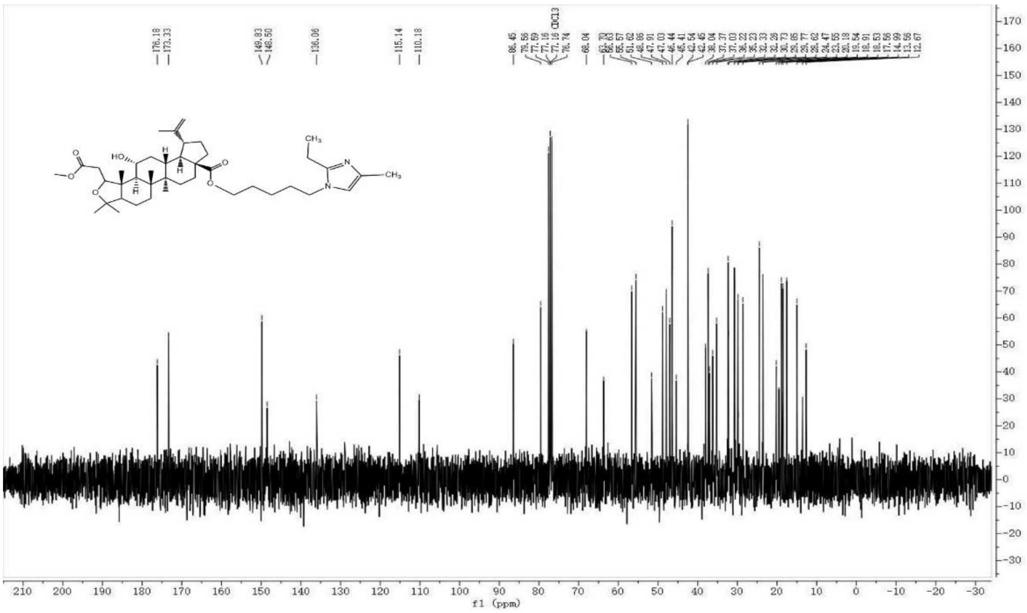

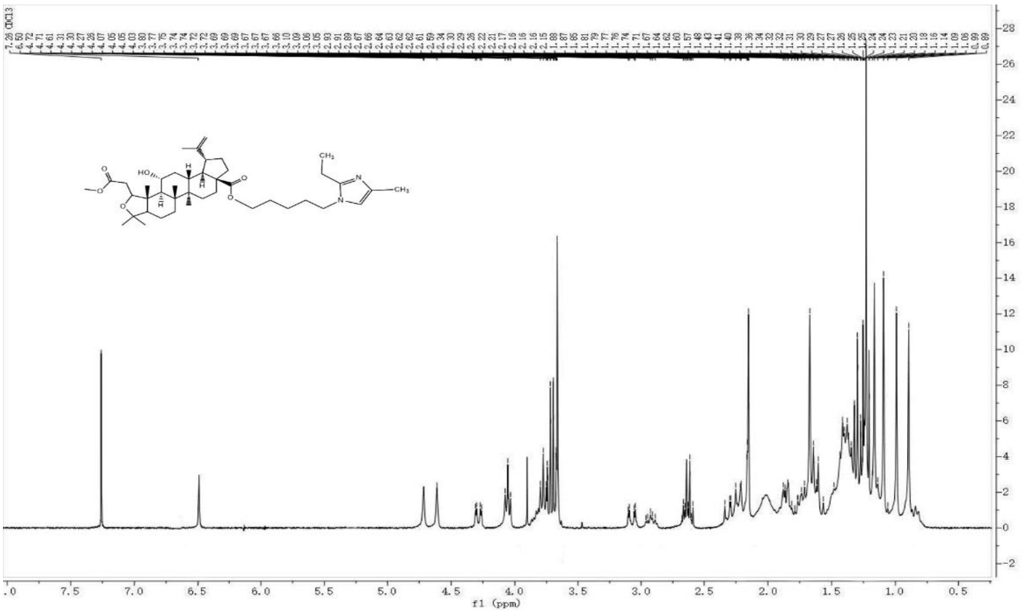

Figures S139. *^{13}C and ^{1}H NMR of compound **II-68**.*

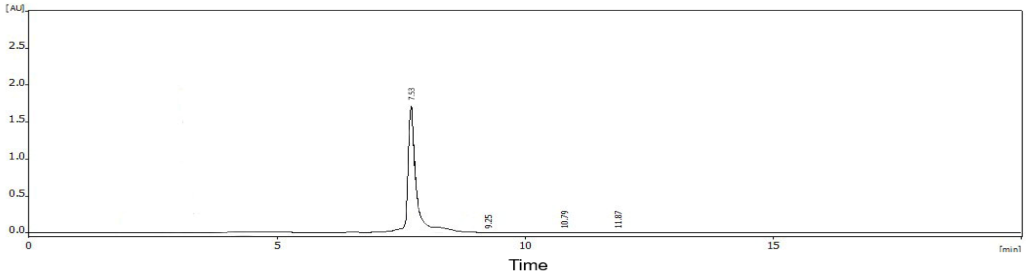

	RT	Area	Area %	Height
1	7.530	21803.323	97.3	1711.159
2	9.247	380.942	1.7	1.266
3	10.787	129.795	0.6	2.091
4	11.870	85.421	0.4	1.616

Time	A (water,0.5% Phosphoric acid)	B (Acetonitrile)
0	65	35
10	10	90
30	10	90
40	65	35

Figure S140. *HPLC tracing of compound **II-69**.*

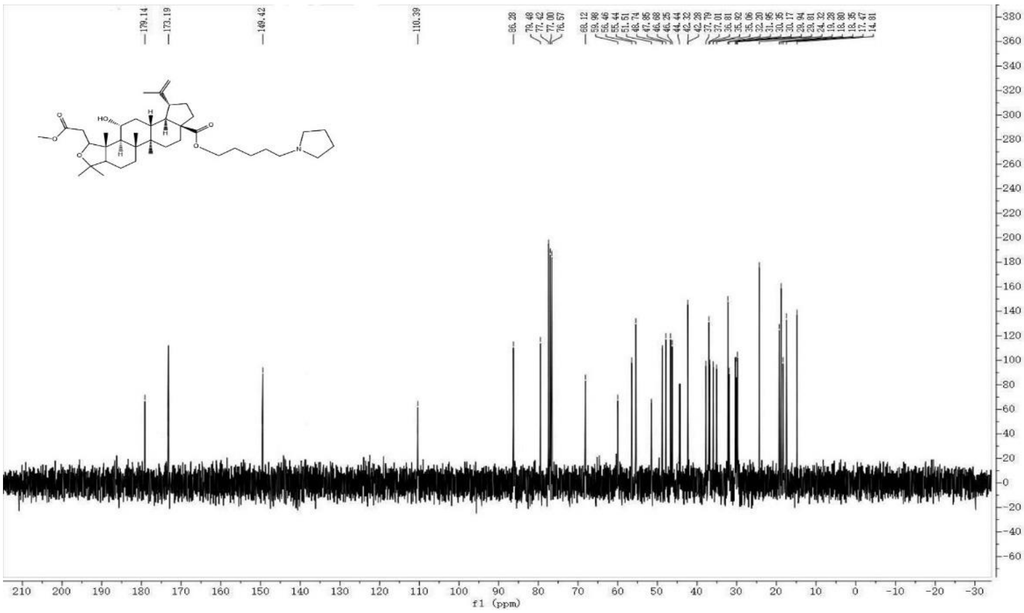

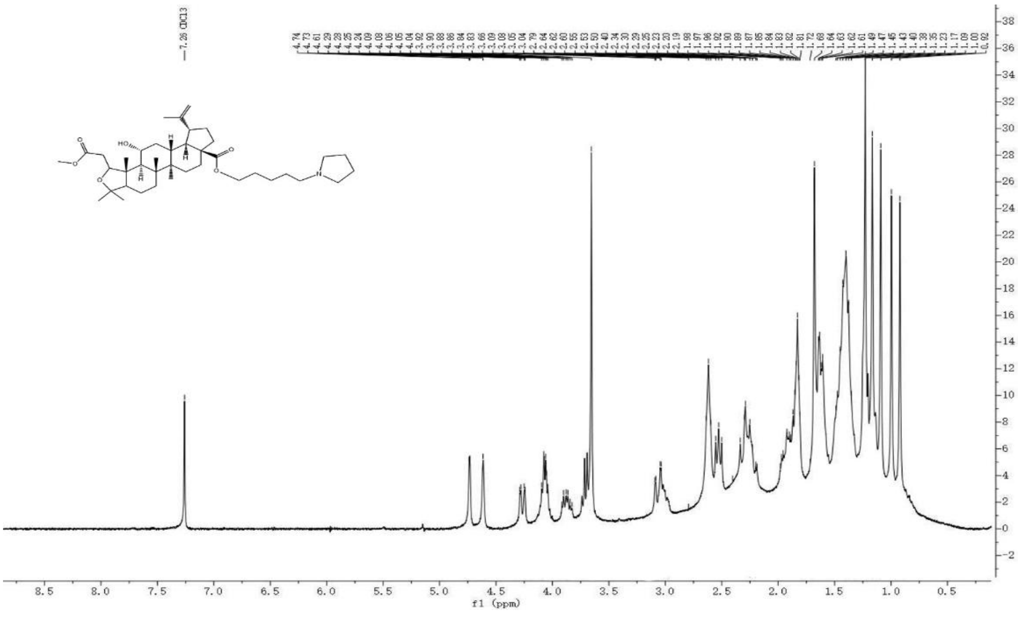

Figures S141. *^{13}C and ^{1}H NMR of compound **II-69**.*

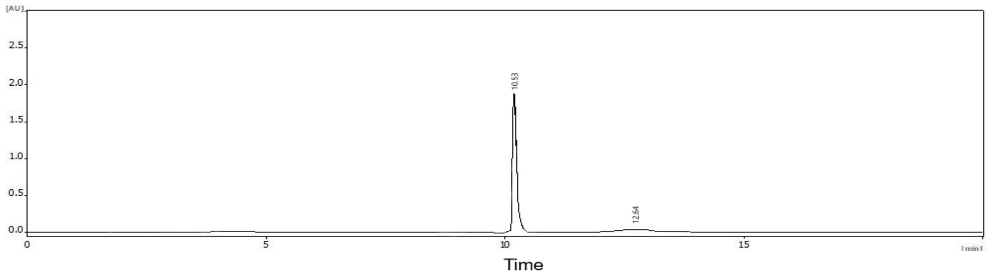

	RT	Area	Area %	Height
1	10.531	18952.403	99.8	1454.305
2	12.643	36.986	0.2	0.733

Time	A (water,0.5% Phosphoric acid)	B (Acetonitrile)
0	65	35
10	10	90
30	10	90
40	65	35

Figure S142. *HPLC tracing of compound* ***II-70****.*

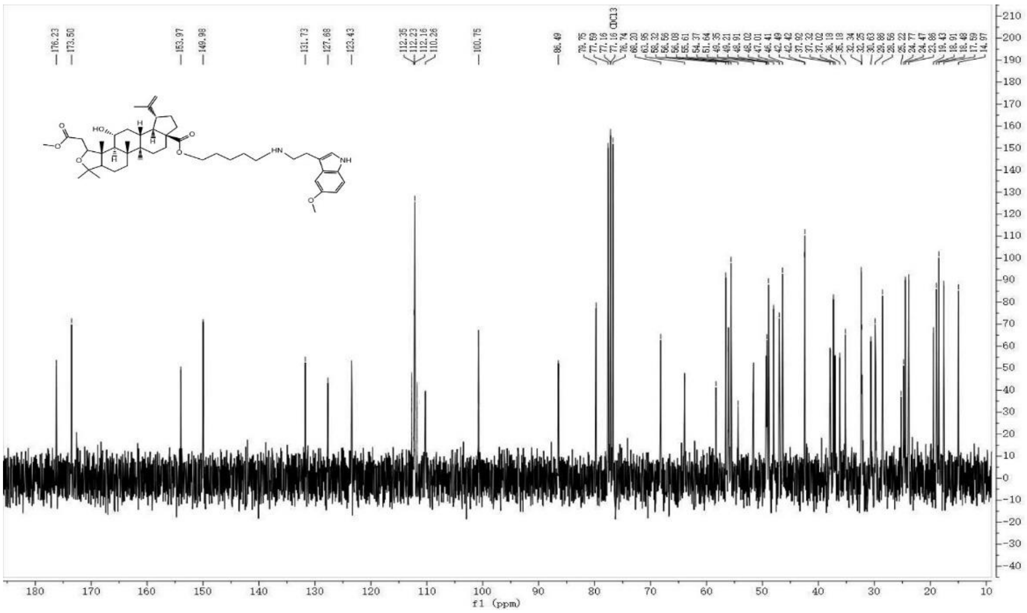

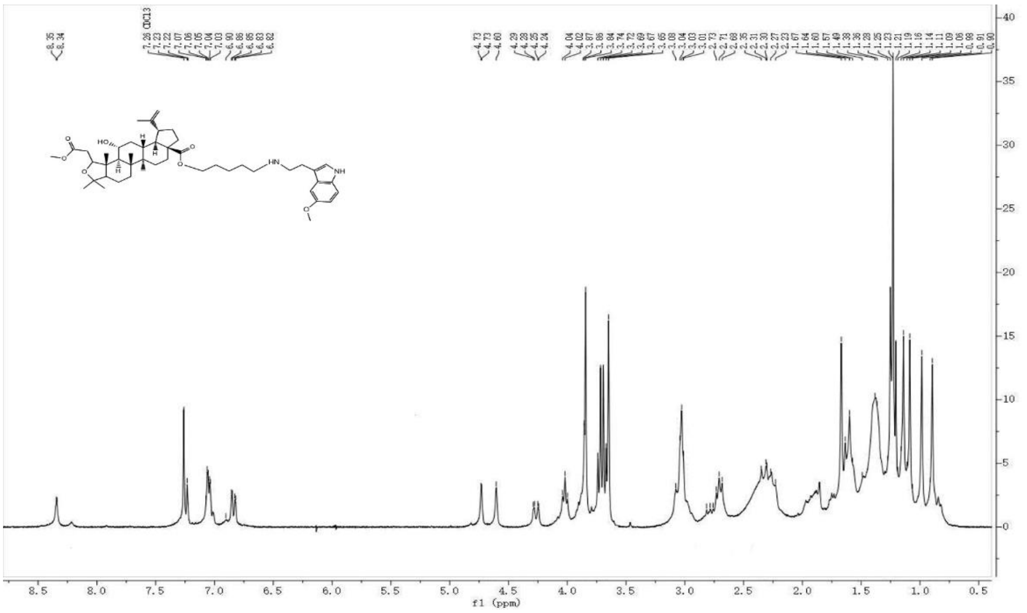

Figures S143. *^{13}C and ^{1}H NMR of compound* ***II-70****.*

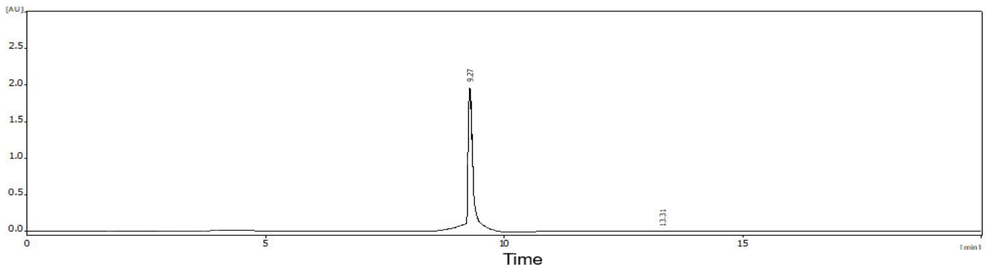

	RT	Area	Area %	Height
1	9.270	15749.693	98.5	1960.859
2	13.310	246.622	1.5	2.420

Time	A (water,0.5% Phosphoric acid)	B (Acetonitrile)
0	65	35
10	10	90
30	10	90
40	65	35

Figure S144. *HPLC tracing of compound **II-71**.*

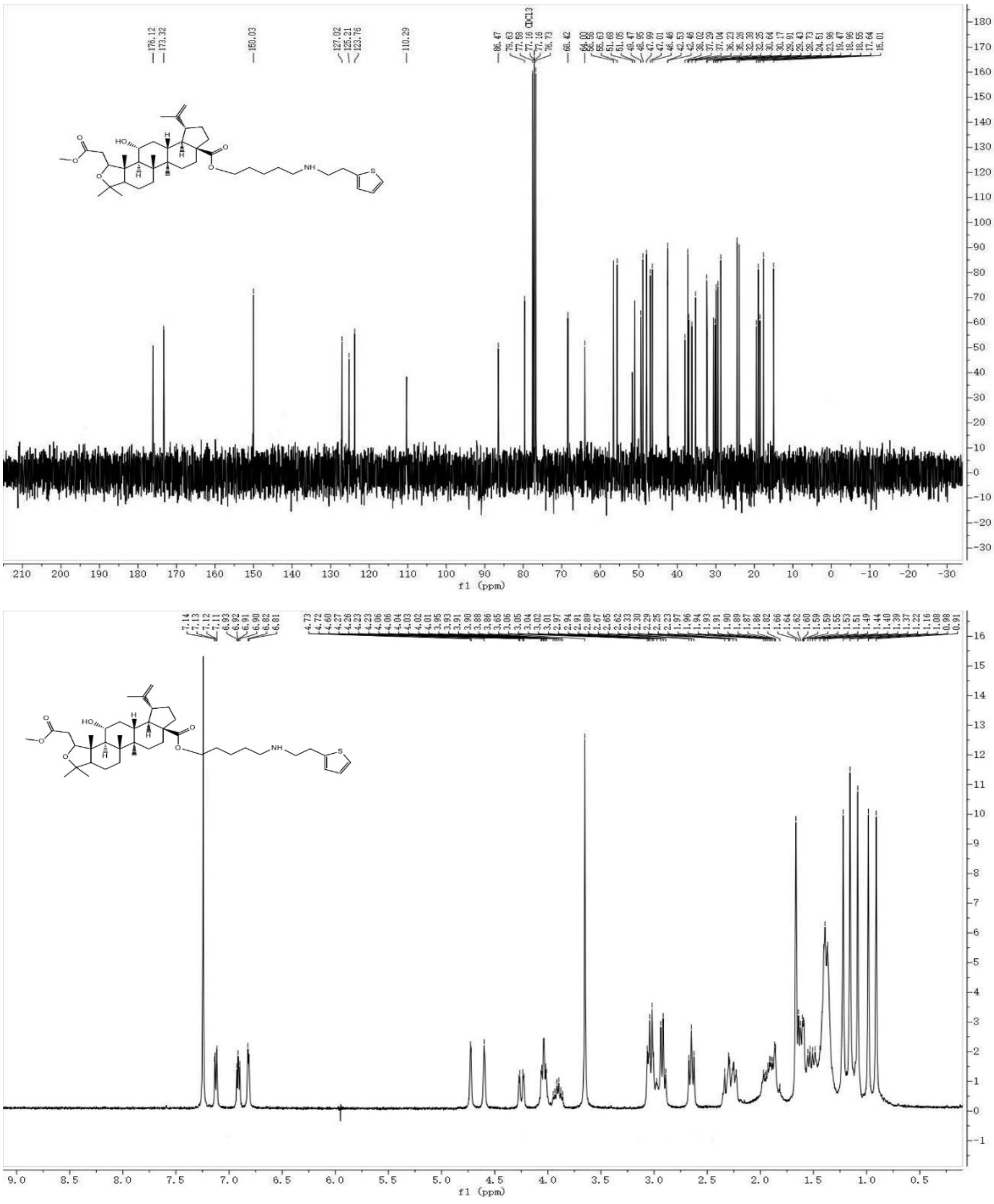

Figures S145. *^{13}C and ^{1}H NMR of compound **II-71**.*

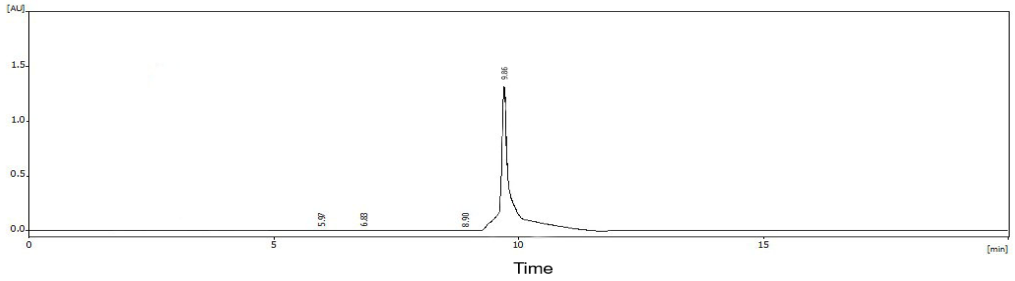

	RT	Area	Area %	Height
1	5.973	89.965	0.4	3.386
2	6.833	56.789	0.3	3.325
3	8.903	11.639	0.1	0.671
4	9.862	21295.712	99.3	1330.057

Time	A (water,0.5% Phosphoric acid)	B (Acetonitrile)
0	65	35
10	10	90
30	10	90
40	65	35

Figure S146. *HPLC tracing of compound* ***II-72***.

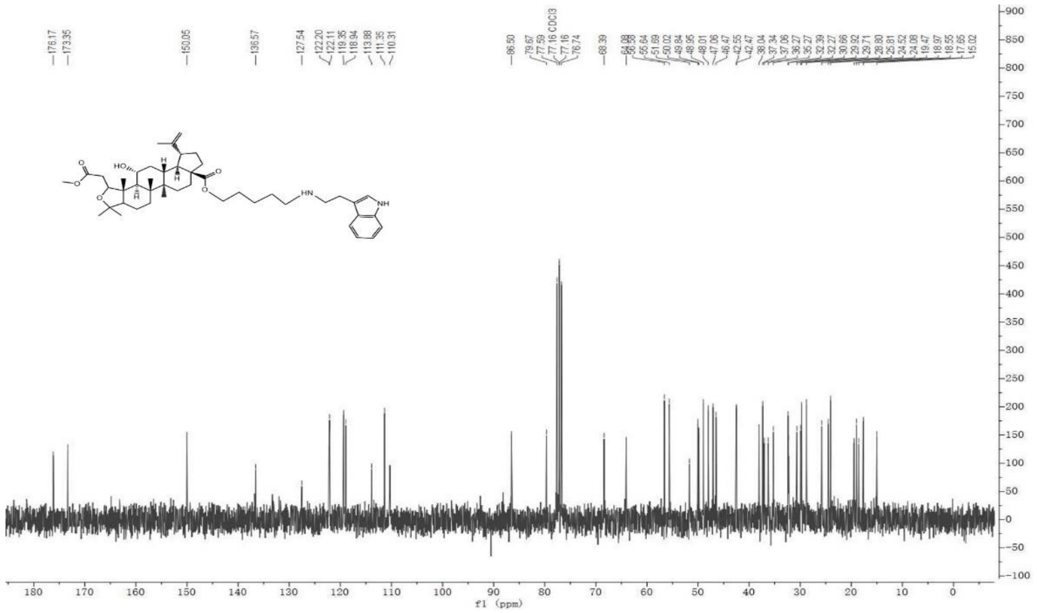

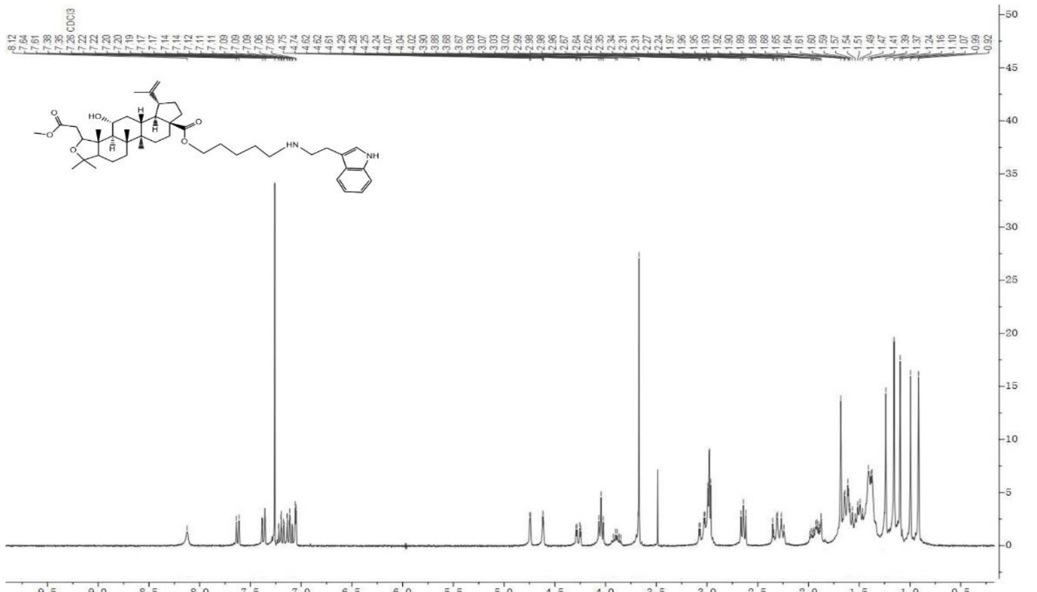

Figures S147. *^{13}C and ^{1}H NMR of compound* ***II-72***.

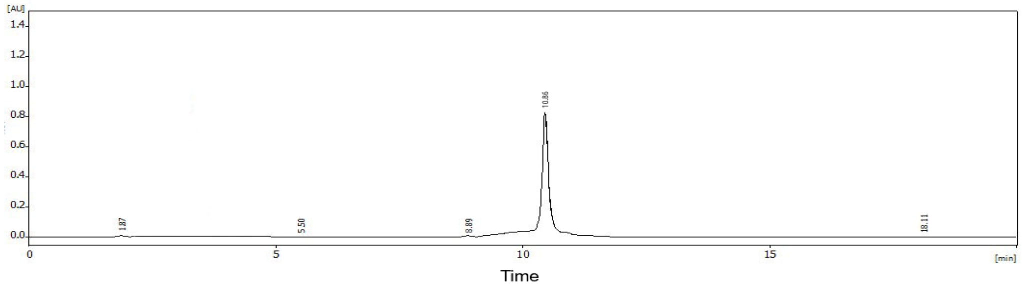

	RT	Area	Area %	Height
1	1.870	150.912	1.3	8.912
2	5.497	311.274	2.7	4.179
3	8.893	43.449	0.4	0.745
4	10.864	11108.178	95.6	829.946
5	18.113	8.357	0.1	0.257

Time	A (water,0.5% Phosphoric acid)	B (Acetonitrile)
0	65	35
10	10	90
30	10	90
40	65	35

Figure S148. *HPLC tracing of compound **II-73**.*

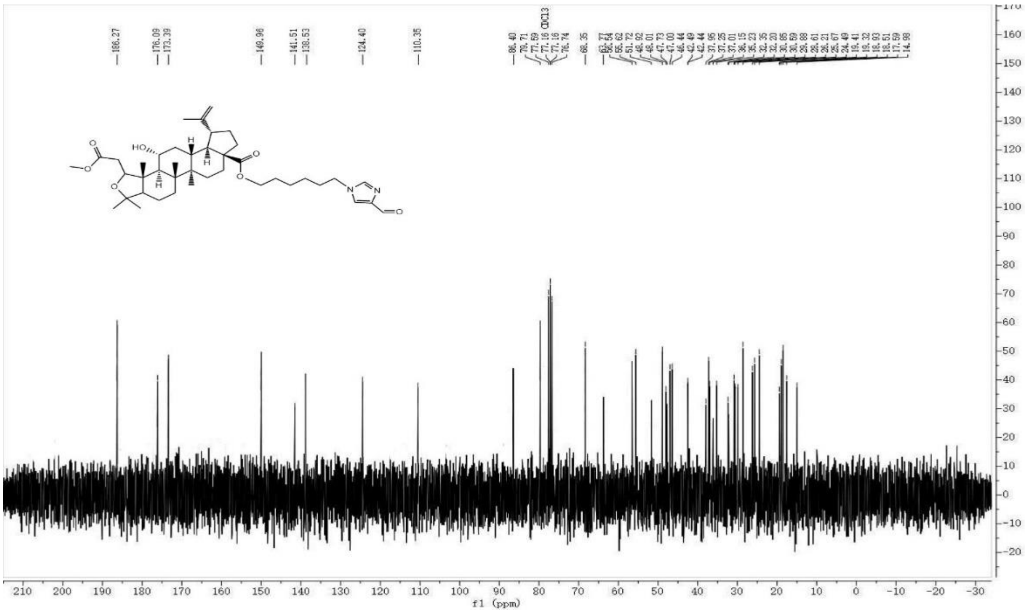

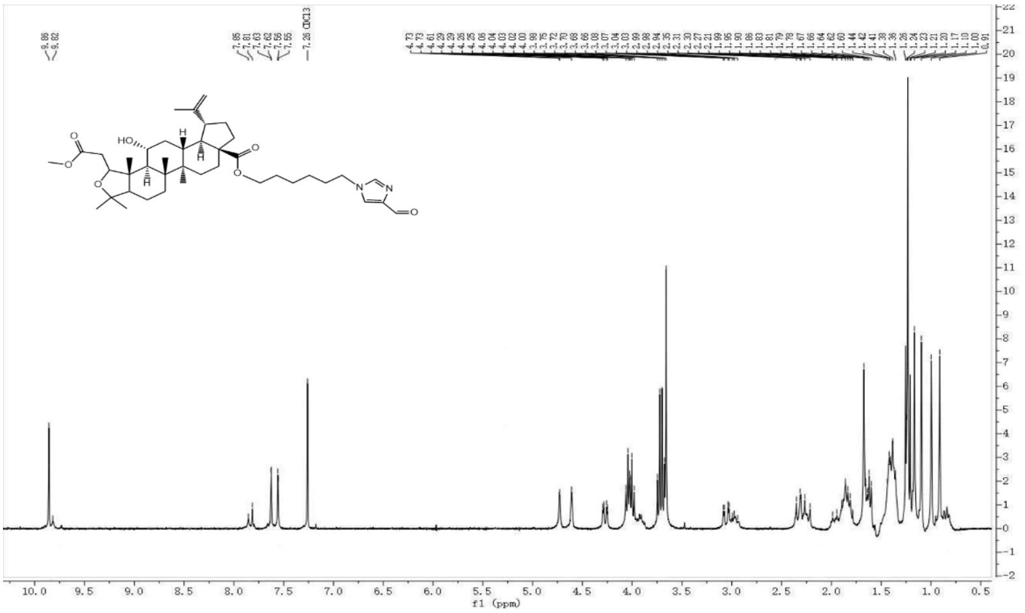

Figures S149. *^{13}C and ^{1}H NMR of compound **II-73**.*

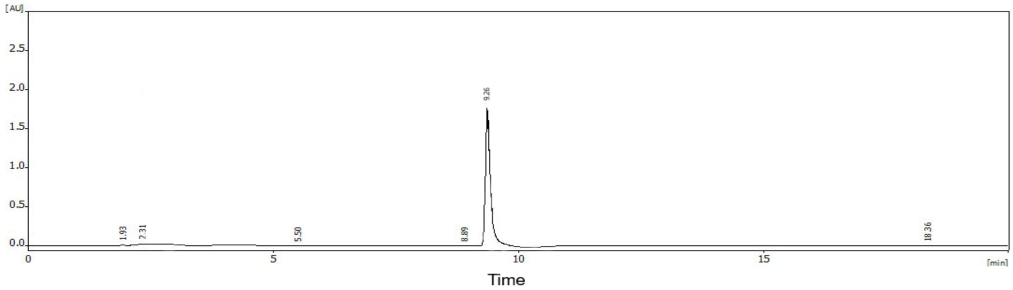

	RT	Area	Area %	Height
1	1.927	119.078	0.8	6.833
2	2.313	317.747	2.2	29.154
3	5.497	13.546	0.1	1.026
4	8.893	9.876	0.1	0.333
5	9.264	13785.856	96.2	1782.877
6	18.360	77.997	0.5	1.408

Time	A (water,0.5% Phosphoric acid)	B (Acetonitrile)
0	65	35
10	10	90
30	10	90
40	65	35

Figure S150. *HPLC tracing of compound **II-74**.*

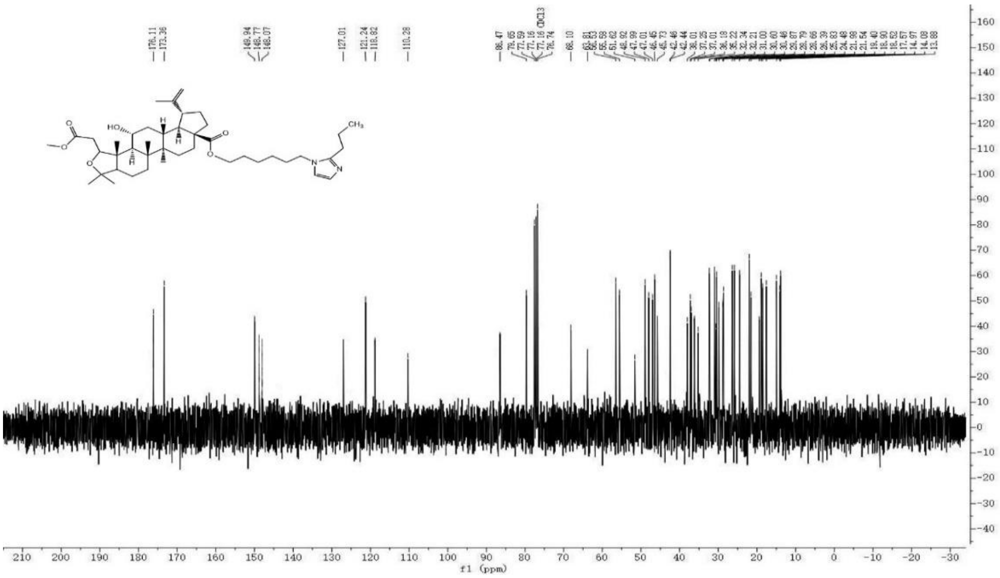

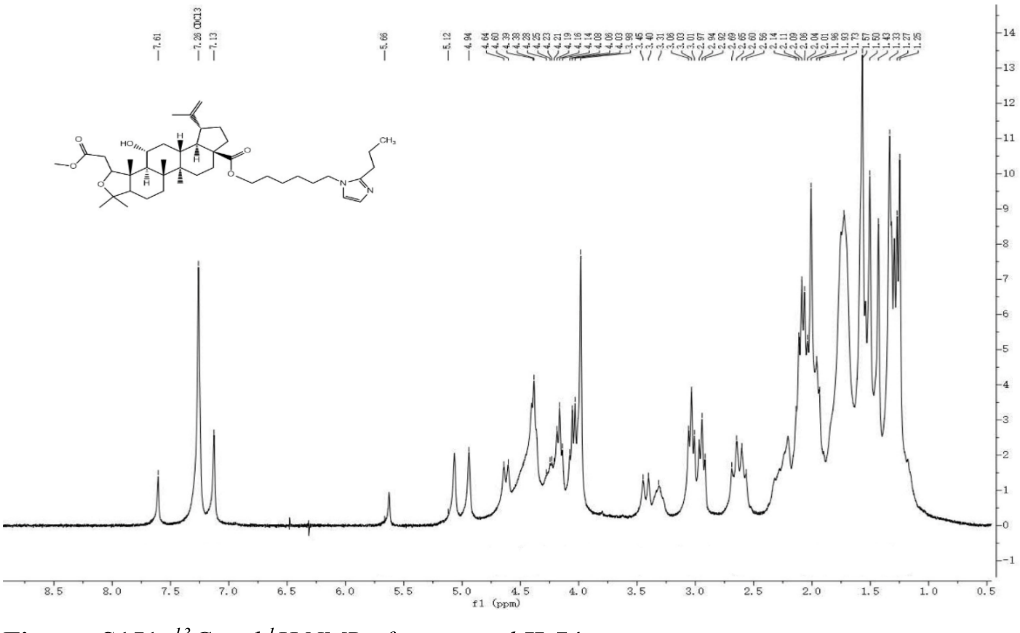

Figures S151. *^{13}C and ^{1}H NMR of compound **II-74**.*

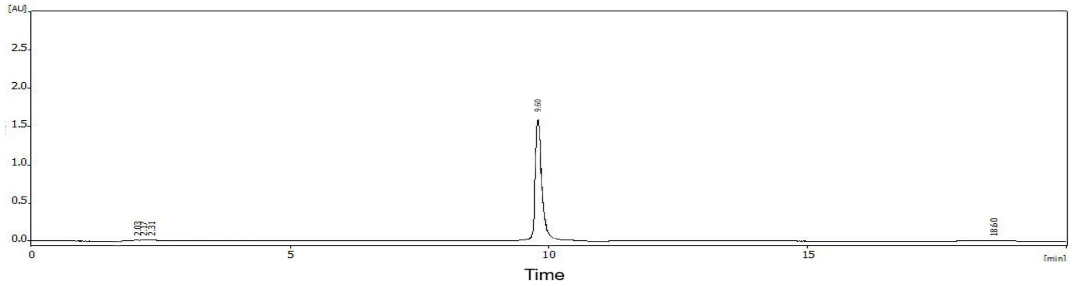

	RT	Area	Area %	Height
1	2.033	172.466	1.0	15.430
2	2.167	164.728	0.9	19.193
3	2.310	285.808	1.6	24.322
4	9.596	17064.762	96.2	1603.021
5	18.603	50.130	0.3	0.928

Time	A (water,0.5% Phosphoric acid)	B (Acetonitrile)
0	65	35
10	10	90
30	10	90
40	65	35

Figure S152. *HPLC tracing of compound **II-75**.*

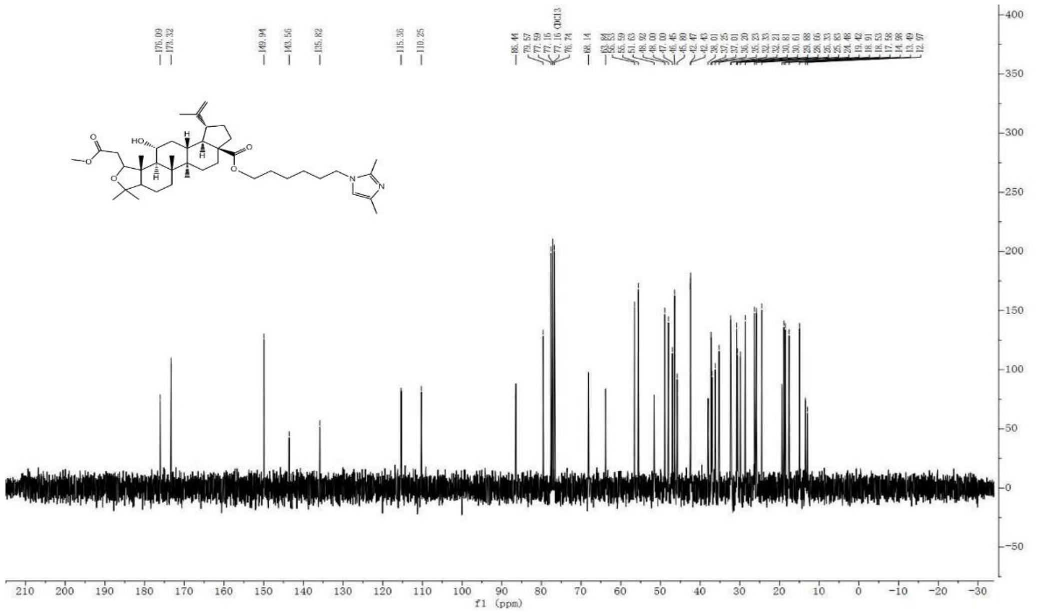

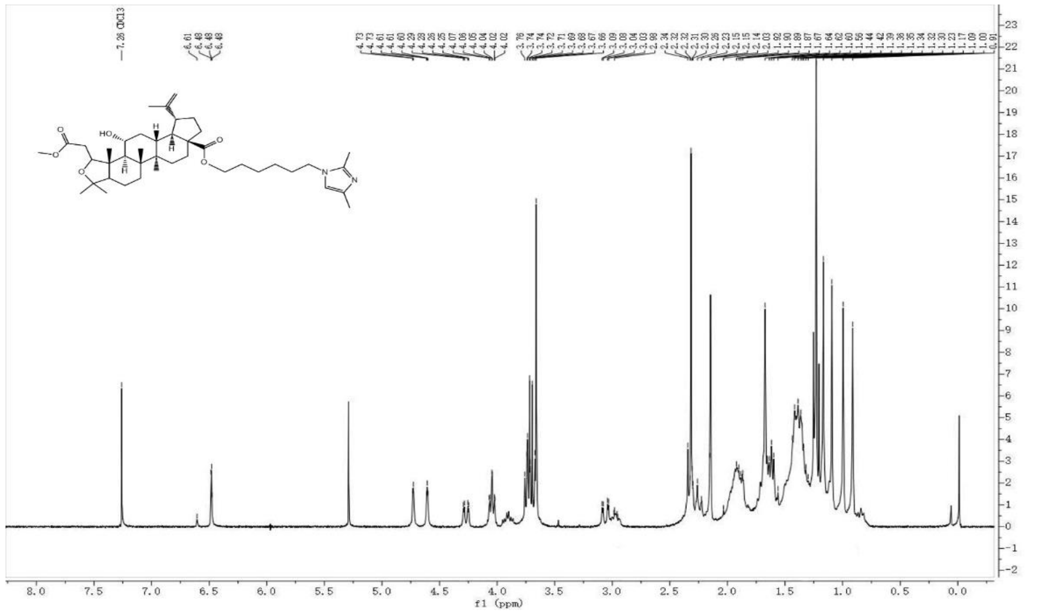

Figures S153. *^{13}C and ^{1}H NMR of compound **II-75**.*

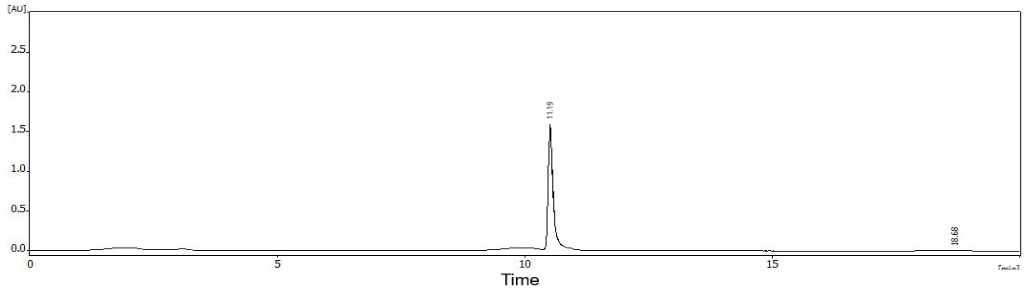

	RT	Area	Area %	Height
1	11.185	11532.391	99.7	1574.161
2	18.680	36.931	0.3	0.577

Time	A (water,0.5% Phosphoric acid)	B (Acetonitrile)
0	65	35
10	10	90
30	10	90
40	65	35

Figure S154. *HPLC tracing of compound **II-76**.*

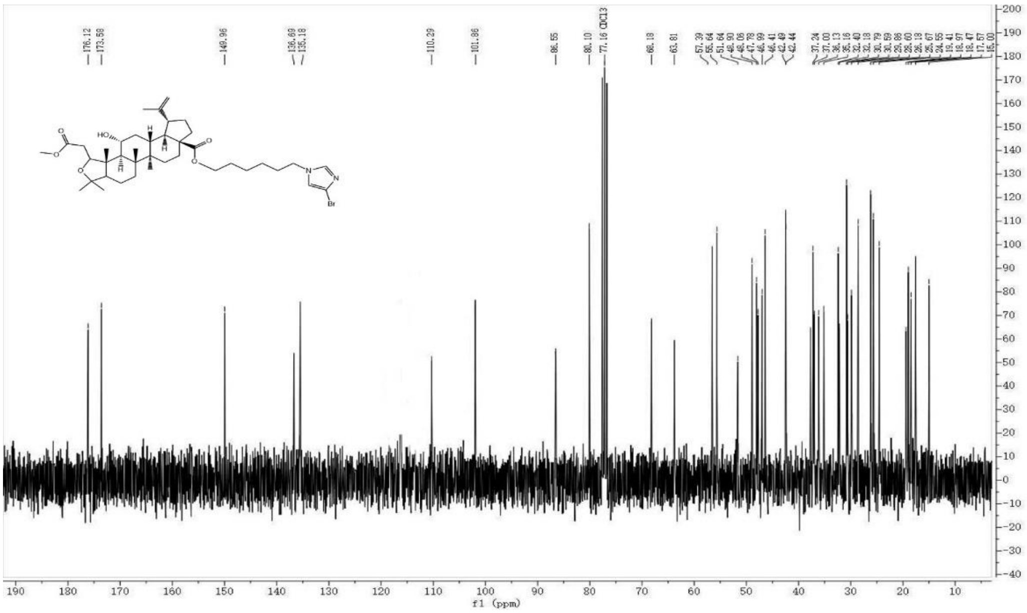

za

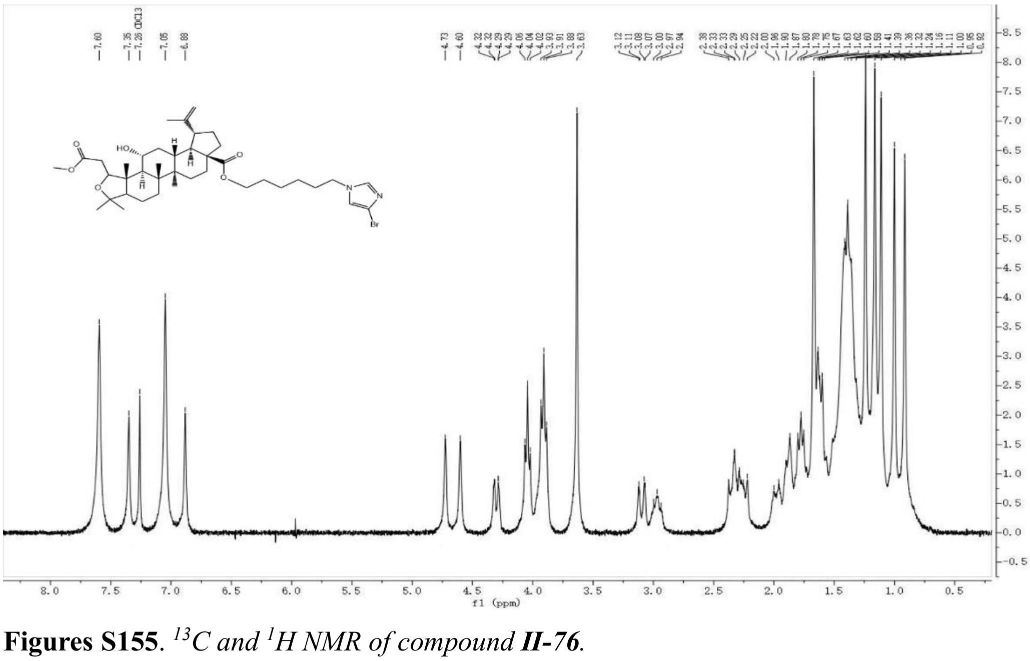

Figures S155. *^{13}C and ^{1}H NMR of compound **II-76**.*

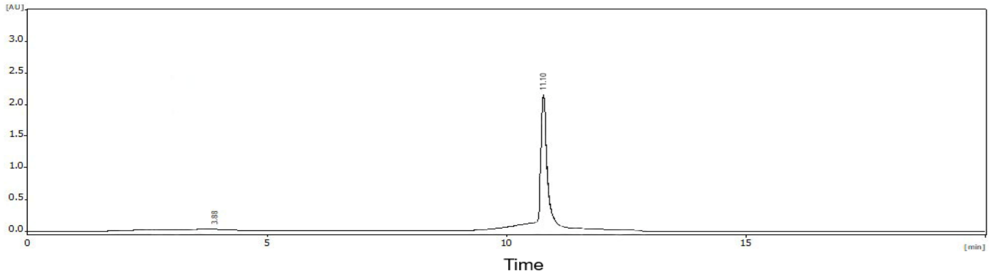

	RT	Area	Area %	Height
1	3.883	1258.154	4.7	33.281
2	11.098	25511.098	95.3	2139.482

Time	A (water,0.5% Phosphoric acid)	B (Acetonitrile)
0	65	35
10	10	90
30	10	90
40	65	35

Figure S156. *HPLC tracing of compound **II-77**.*

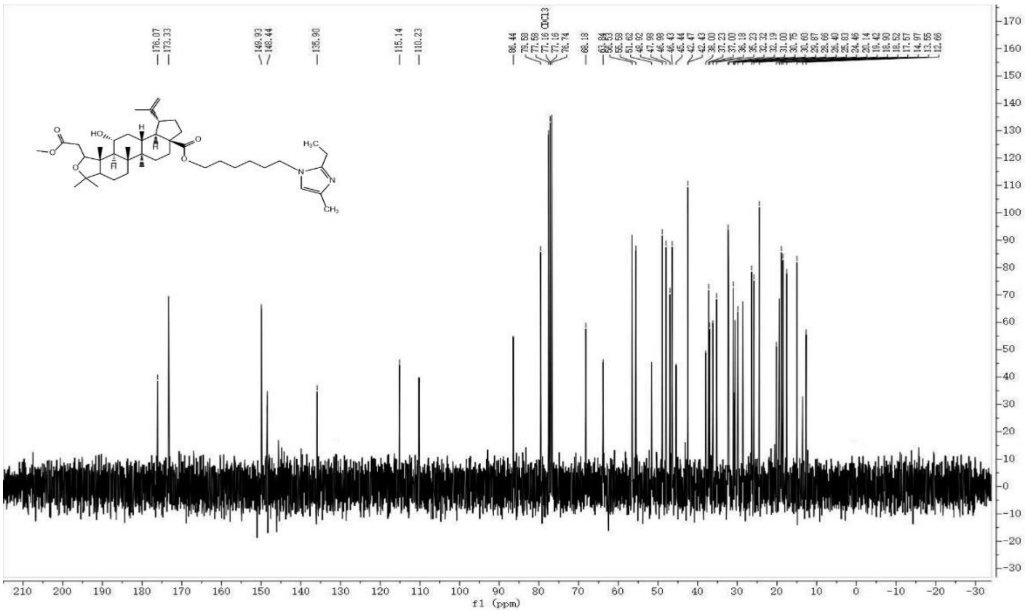

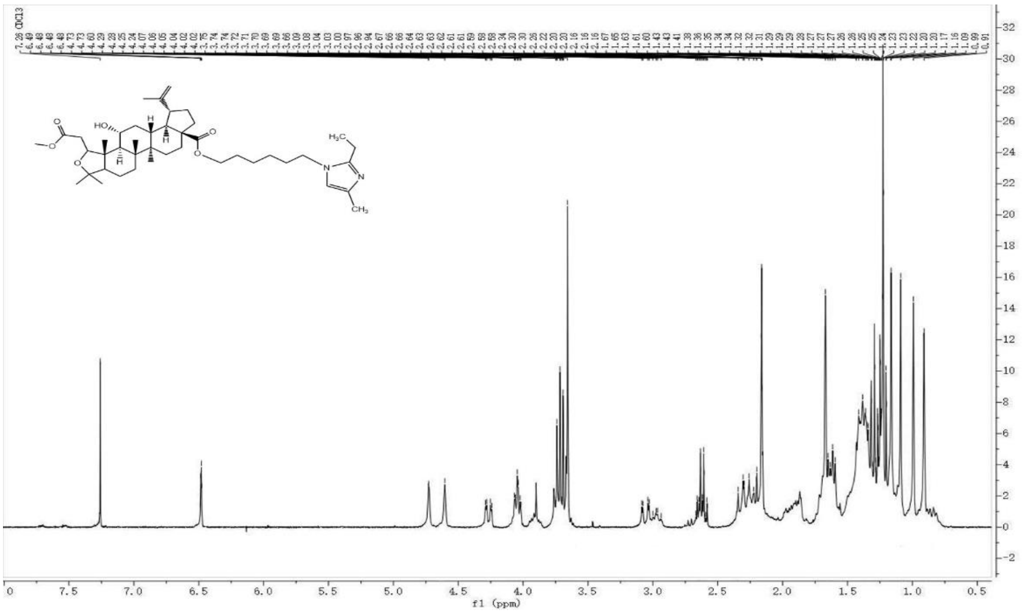

Figures S157. *^{13}C and ^{1}H NMR of compound **II-77**.*

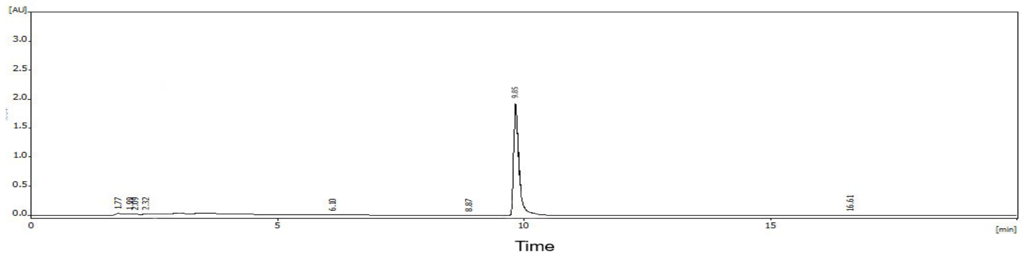

	RT	Area	Area %	Height
1	1.767	204.266	1.3	24.984
2	1.993	73.893	0.5	12.296
3	2.087	100.507	0.7	13.056
4	2.320	62.137	0.4	13.306
5	6.097	12.518	0.1	0.558
6	8.867	5.127	0.0	0.324
7	9.852	14929.115	96.7	1935.786
8	16.613	46.545	0.3	0.481

Time	A (water,0.5% Phosphoric acid)	B (Acetonitrile)
0	65	35
10	10	90
30	10	90
40	65	35

Figure S158. *HPLC tracing of compound* ***II-78***.

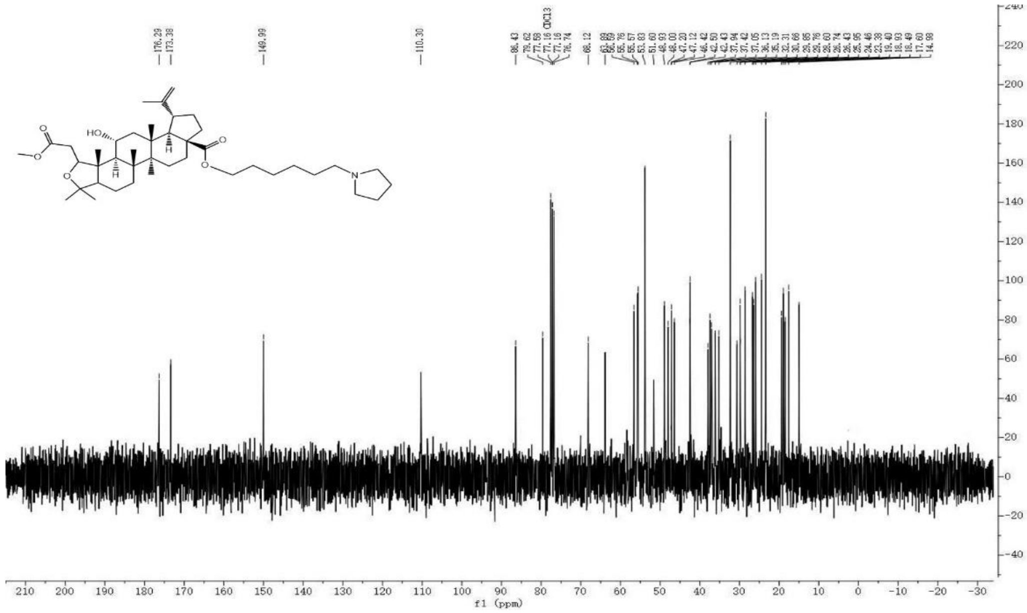

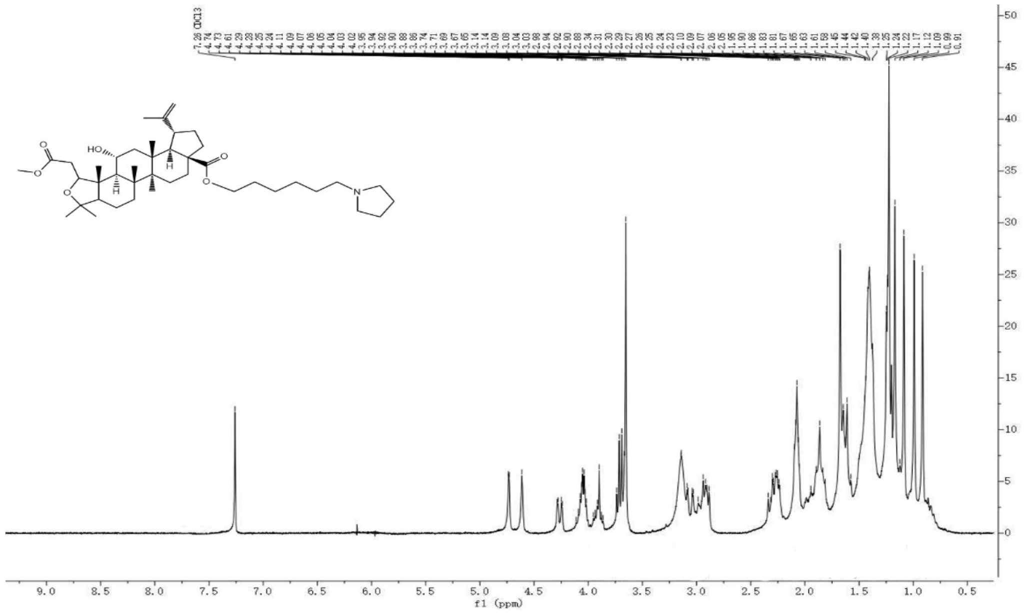

Figures S159. *^{13}C and ^{1}H NMR of compound* ***II-78***.

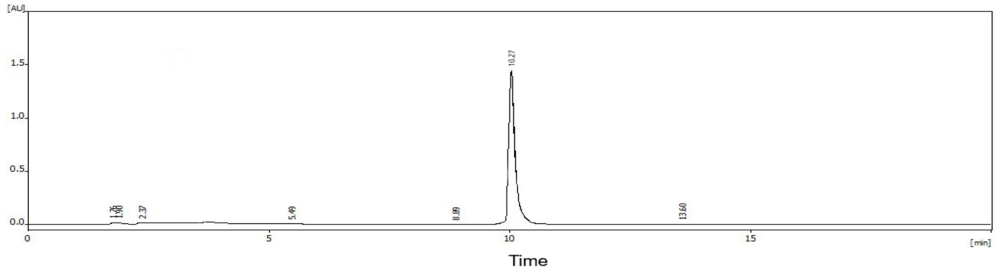

	RT	Area	Area %	Height
1	1.763	138.614	0.9	19.338
2	1.900	129.571	0.8	15.297
3	2.373	398.471	2.5	18.230
4	5.487	52.705	0.3	2.408
5	8.887	8.504	0.1	0.530
6	10.267	15417.572	95.4	1458.675
7	13.597	7.412	0.0	0.259

Time	A (water,0.5% Phosphoric acid)	B (Acetonitrile)
0	65	35
10	10	90
30	10	90
40	65	35

Figure S160. *HPLC tracing of compound **II-79**.*

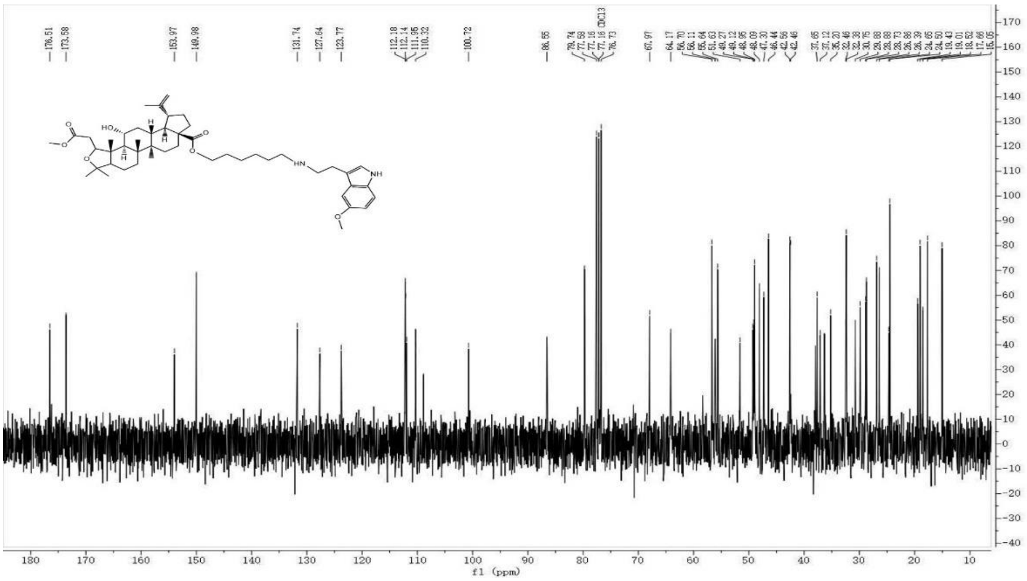

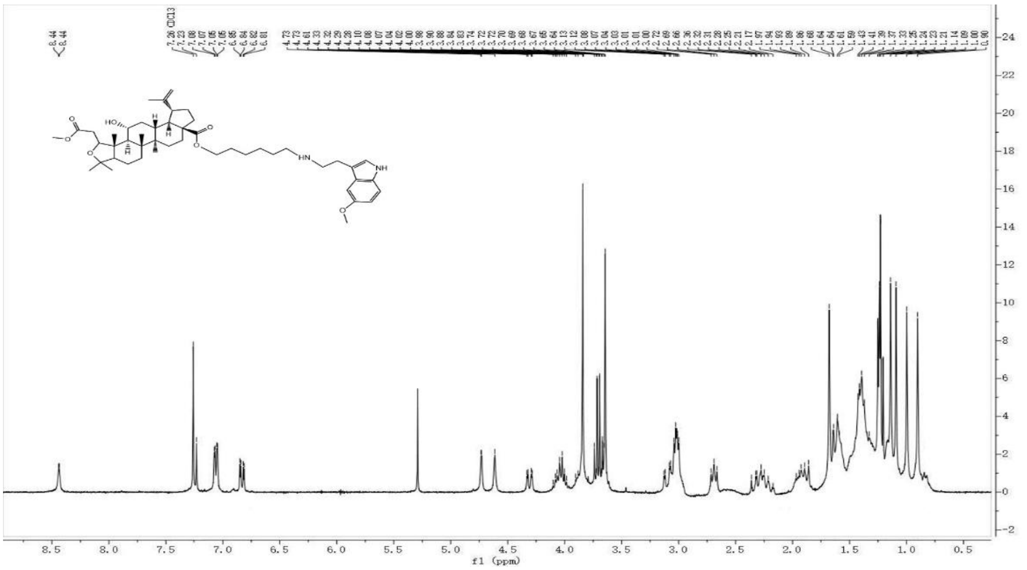

Figures S161. *^{13}C and ^{1}H NMR of compound **II-79**.*

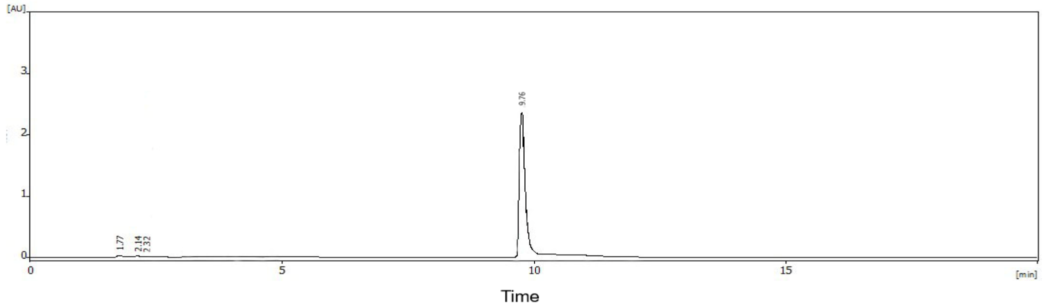

	RT	Area	Area %	Height
1	1.767	328.171	1.2	33.756
2	2.143	311.151	1.1	28.971
3	2.317	119.208	0.4	24.167
4	9.763	26609.231	97.3	2383.492

Time	A (water,0.5% Phosphoric acid)	B (Acetonitrile)
0	65	35
10	10	90
30	10	90
40	65	35

Figure S162. *HPLC tracing of compound **II-80**.*

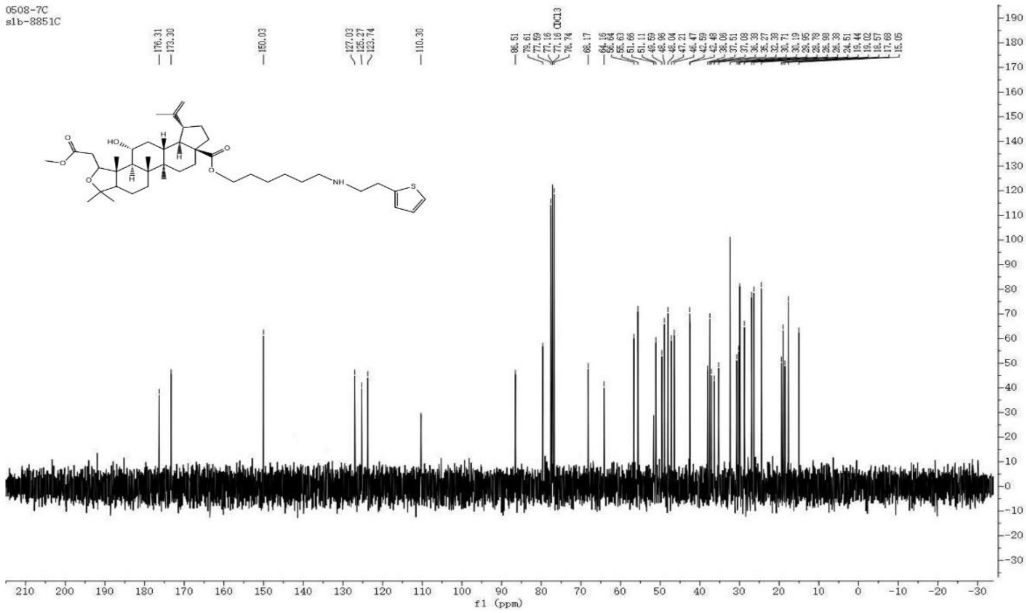

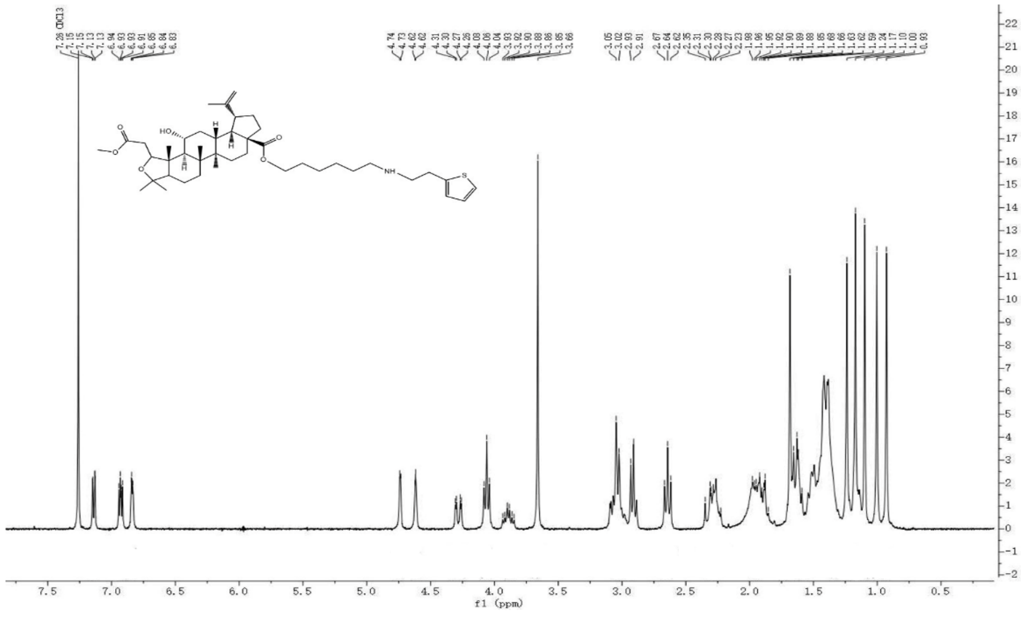

Figures S163. *^{13}C and ^{1}H NMR of compound **II-80**.*

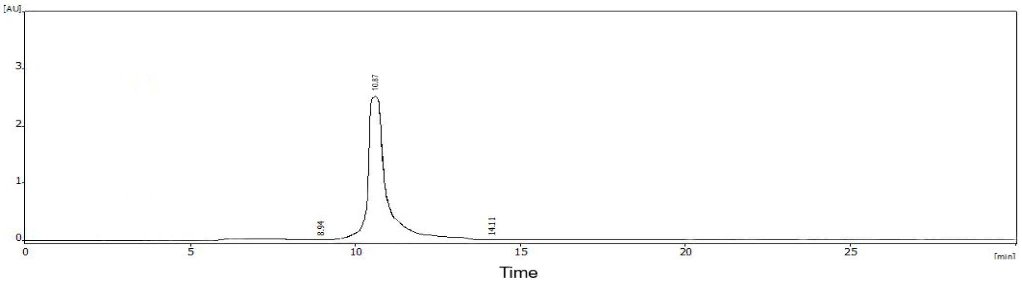

	RT	Area	Area %	Height
1	10.873	100633.074	98.2	2511.592
2	8.937	966.127	0.9	11.903
4	14.113	958.593	0.9	0.857

Time	A (water,0.5% Phosphoric acid)	B (Acetonitrile)
0	65	35
10	10	90
30	10	90
40	65	35

Figure S164. *HPLC tracing of compound **II-81**.*

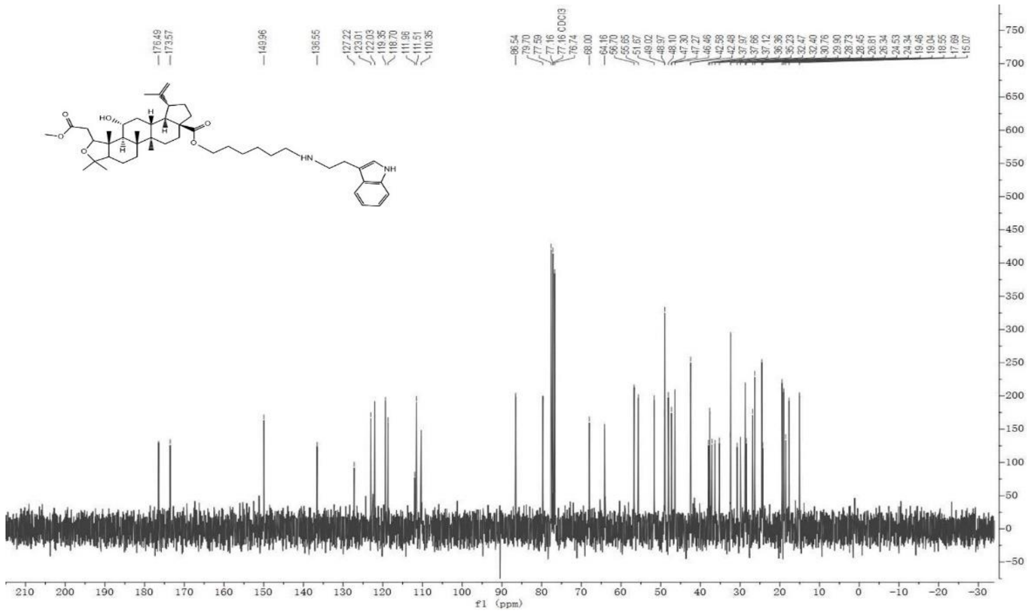

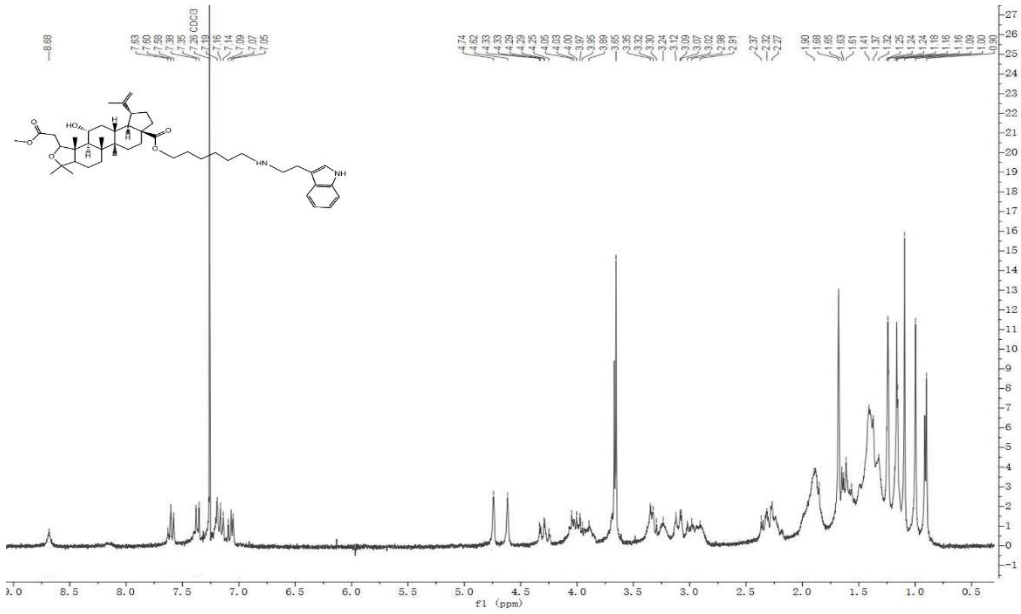

Figures S165. *^{13}C and ^{1}H NMR of compound **II-81**.*

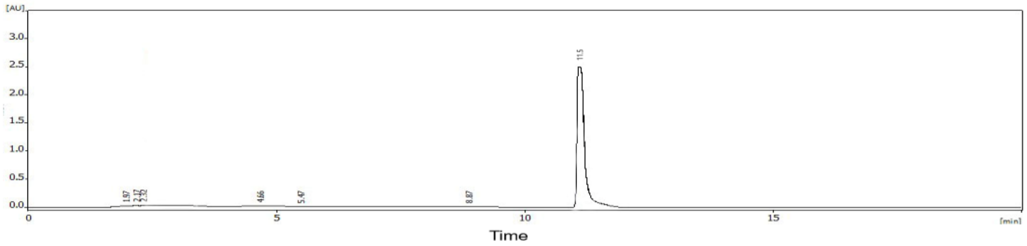

	RT	Area	Area %	Height
1	1.967	179.044	0.6	10.949
2	2.173	285.103	0.9	34.604
3	2.317	187.840	0.6	33.729
4	4.663	399.099	1.3	10.468
5	5.473	276.299	0.9	7.673
6	8.873	67.905	0.2	1.150
7	11.498	29318.419	95.5	2539.345

Time	A (water,0.5% Phosphoric acid)	B (Acetonitrile)
0	65	35
10	10	90
30	10	90
40	65	35

Figure S166. *HPLC tracing of compound **II-82**.*

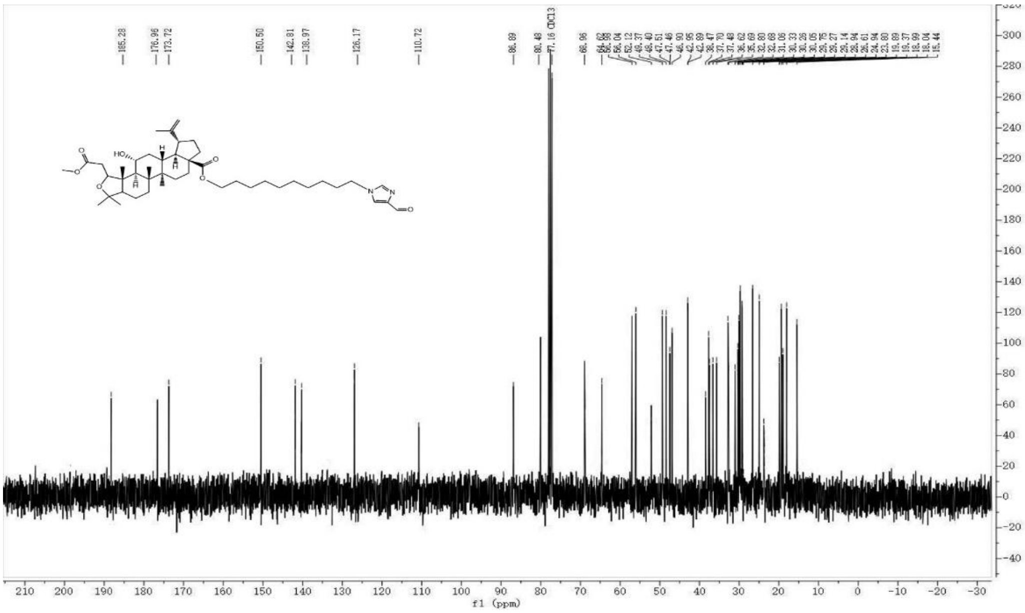

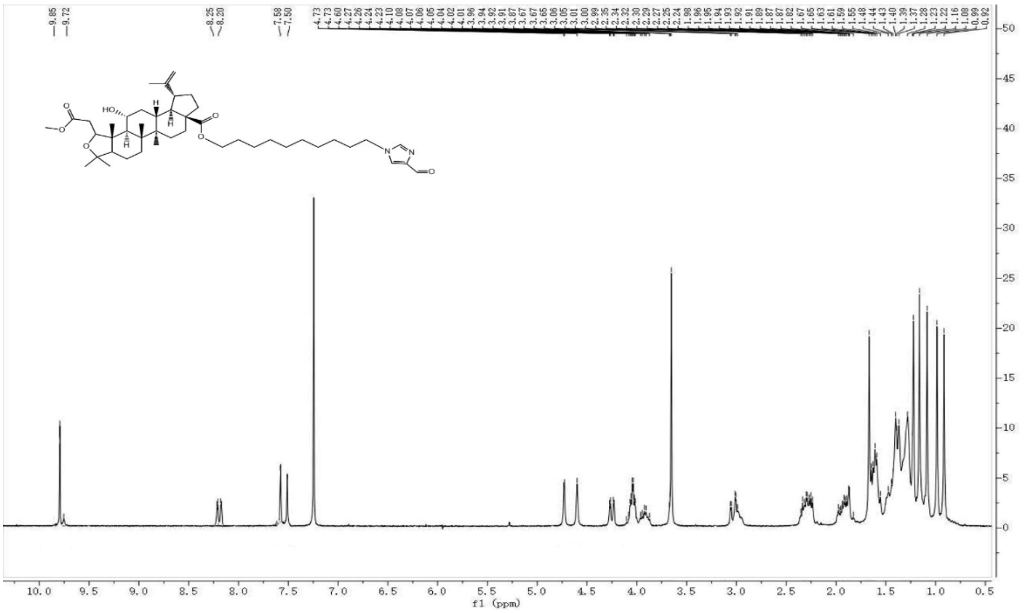

Figures S167. *^{13}C and ^{1}H NMR of compound **II-82**.*

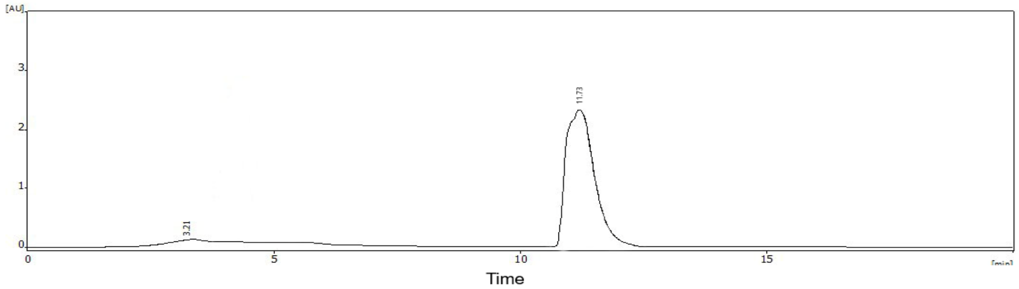

	RT	Area	Area %	Height
1	3.207	94.742	0.1	7.042
2	11.734	95246.637	99.9	2331.658

Time	A (water,0.5% Phosphoric acid)	B (Acetonitrile)
0	65	35
10	10	90
30	10	90
40	65	35

Figure S168. *HPLC tracing of compound **II-83**.*

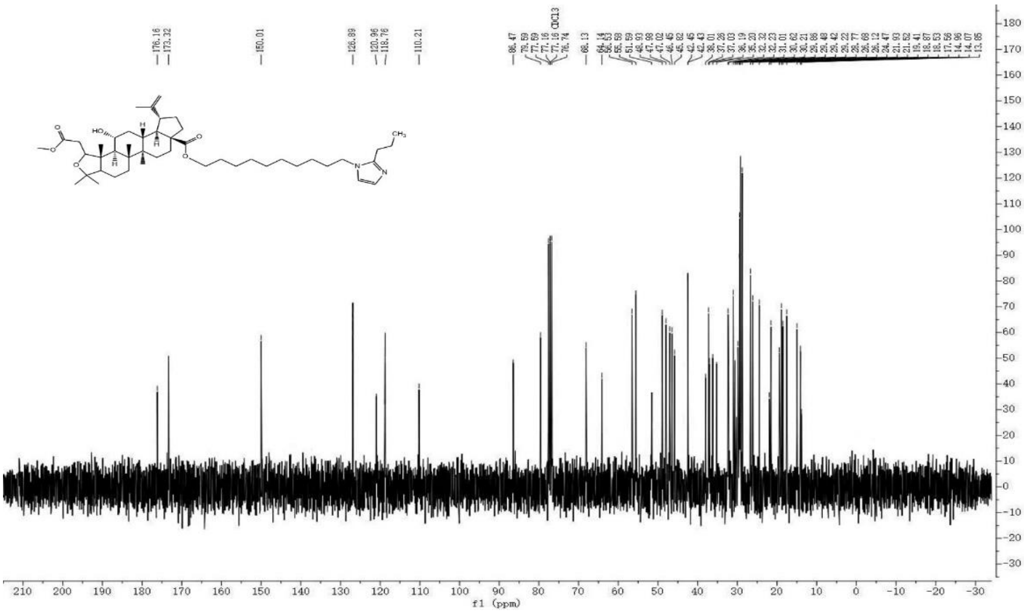

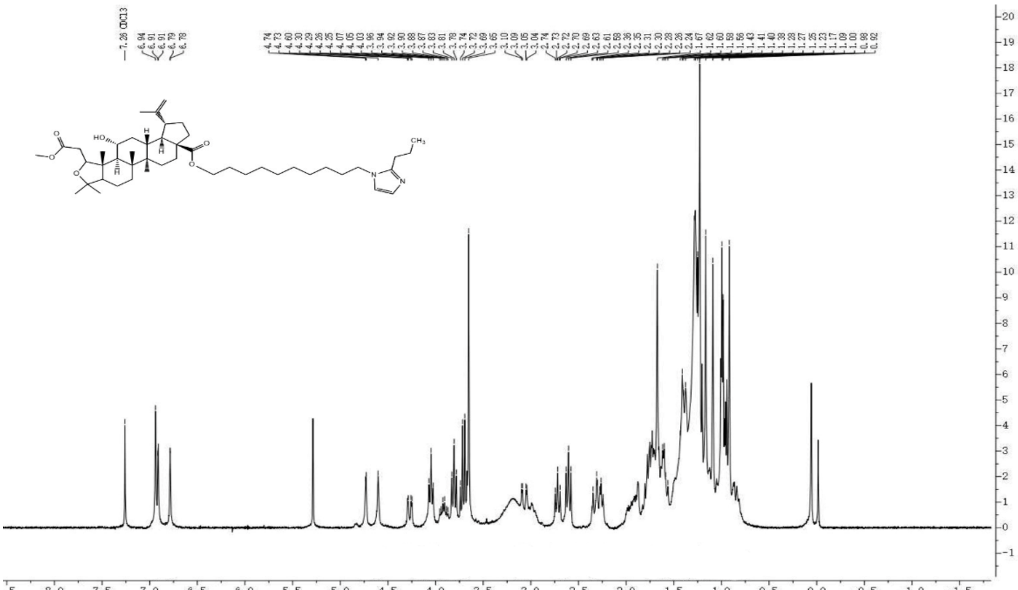

Figures S169. *^{13}C and ^{1}H NMR of compound **II-83**.*

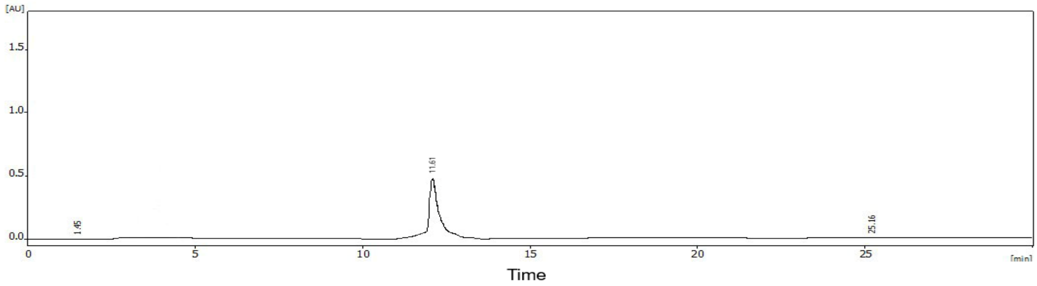

	RT	Area	Area %	Height
1	1.453	85.029	0.7	2.185
2	11.613	12716.116	97.9	476.740
3	25.163	184.030	1.4	0.782

Time	A (water,0.5% Phosphoric acid)	B (Acetonitrile)
0	65	35
10	10	90
30	10	90
40	65	35

Figure S170. *HPLC tracing of compound **II-84**.*

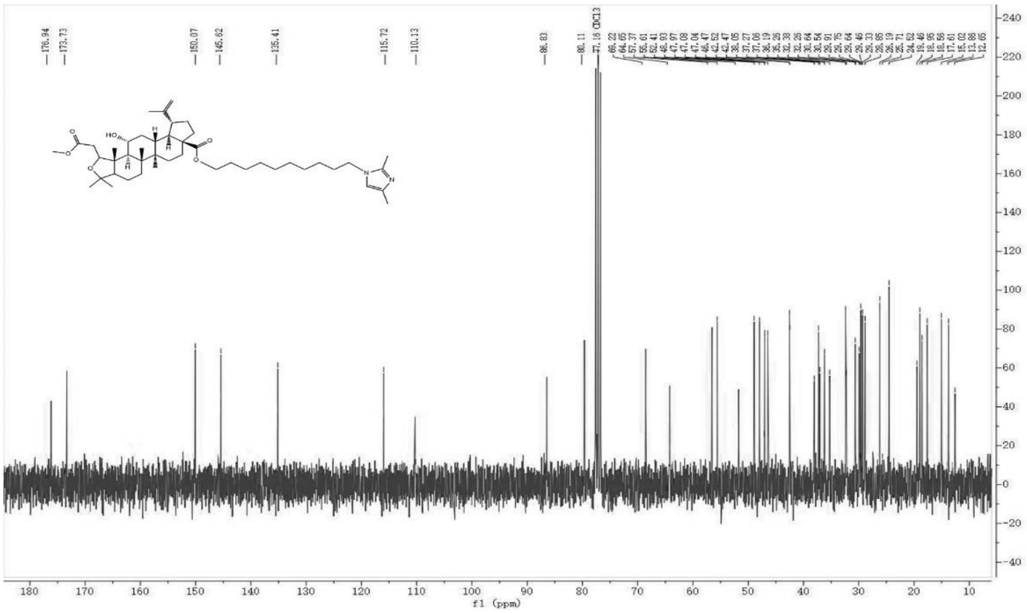

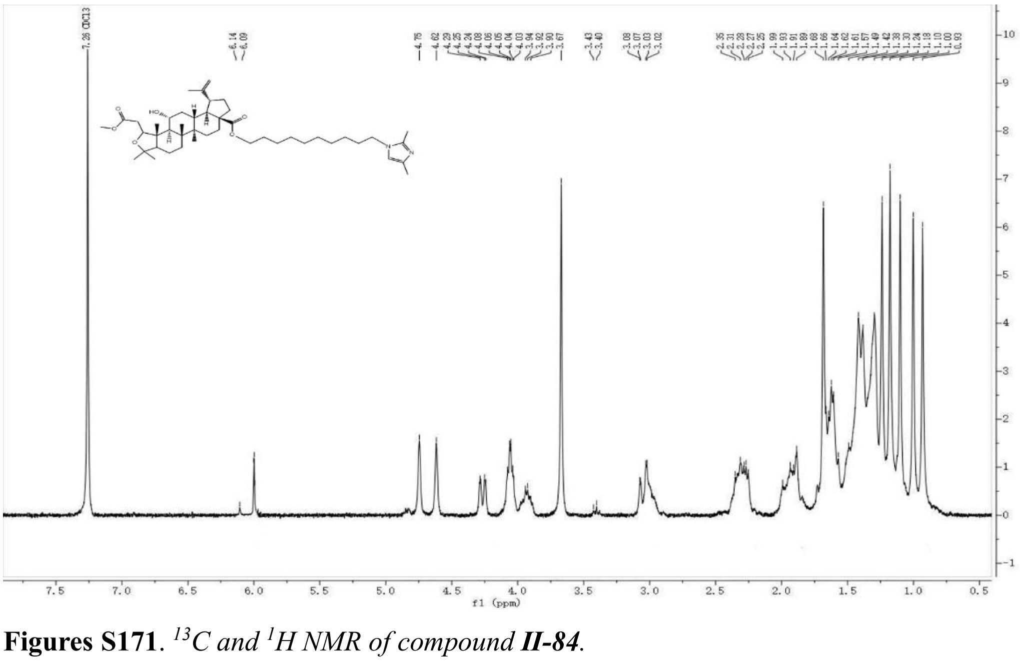

Figures S171. *^{13}C and ^{1}H NMR of compound **II-84**.*

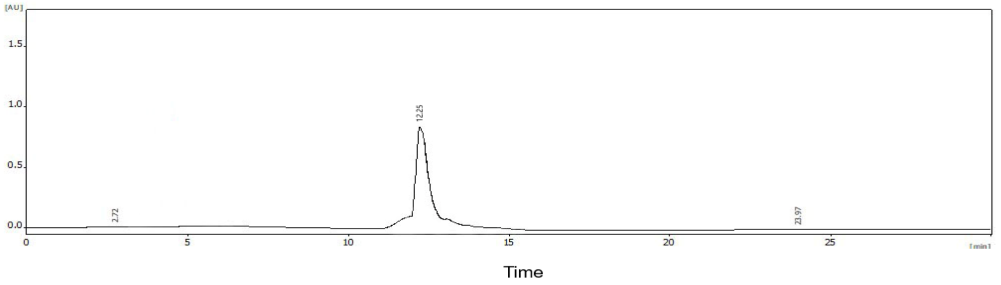

	RT	Area	Area %	Height
1	2.720	568.756	1.6	9.284
2	12.253	34196.448	96.2	850.165
3	23.973	785.248	2.2	3.858

Time	A (water,0.5% Phosphoric acid)	B (Acetonitrile)
0	65	35
10	10	90
30	10	90
40	65	35

Figure S172. *HPLC tracing of compound* ***II-85***.

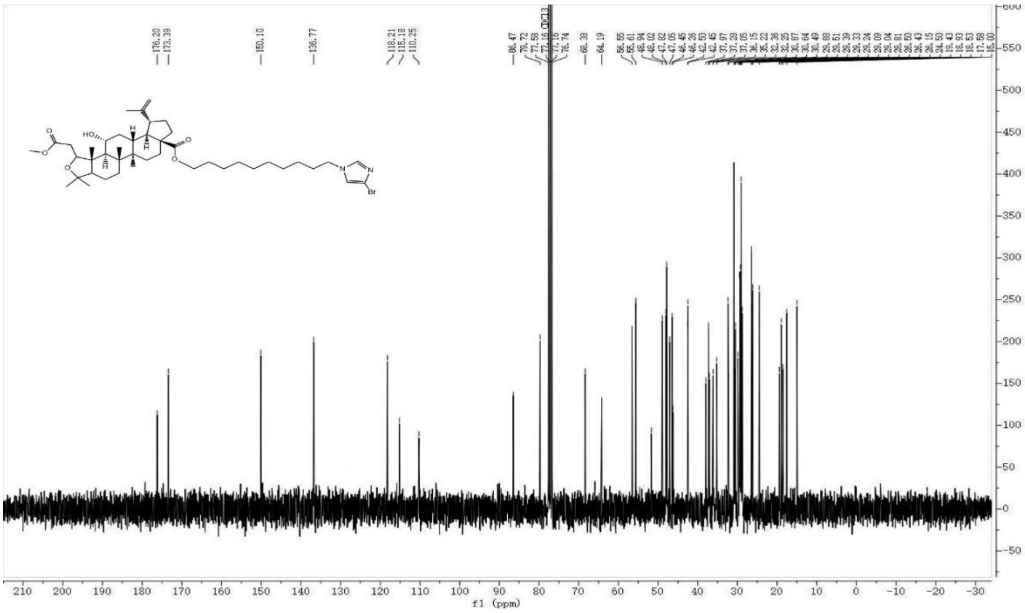

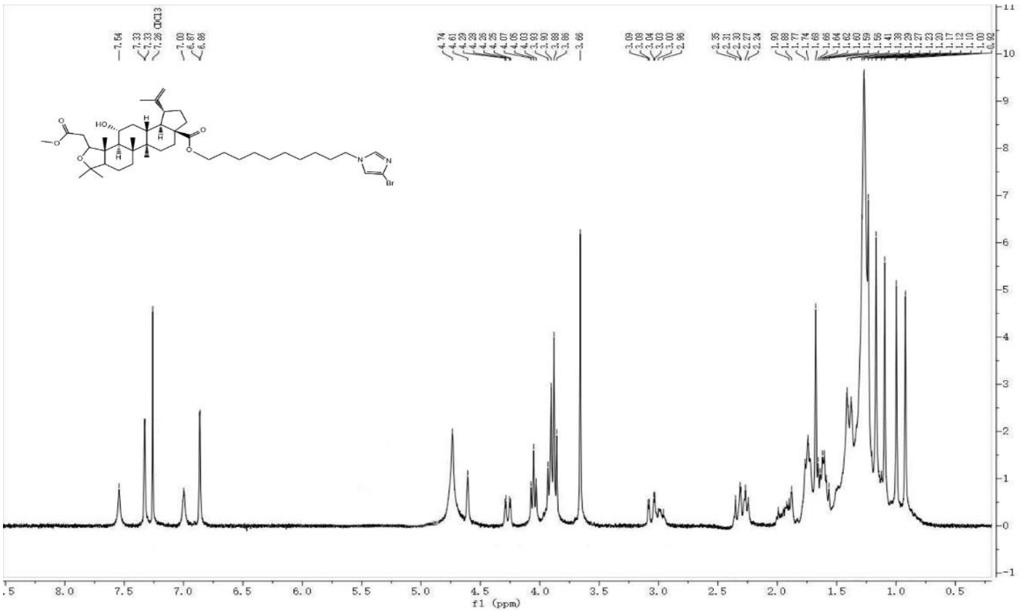

Figures S173. *^{13}C and ^{1}H NMR of compound* ***II-85***.

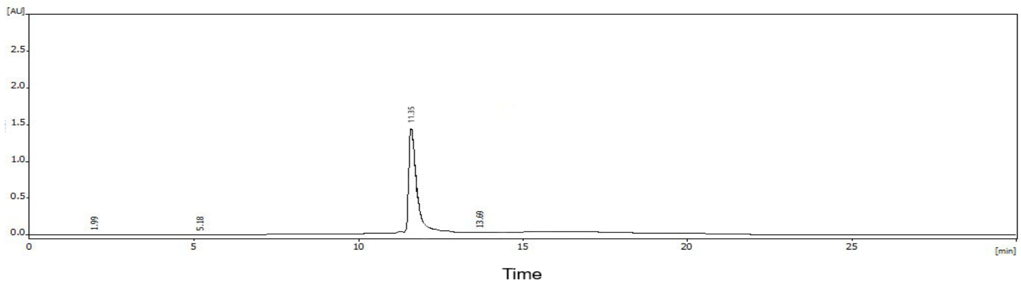

	RT	Area	Area %	Height
1	1.987	198.944	0.4	1.617
2	5.180	112.196	0.2	2.316
3	11.349	44272.793	95.9	1459.003
4	13.687	1615.795	3.5	35.661

Time	A (water,0.5% Phosphoric acid)	B (Acetonitrile)
0	65	35
10	10	90
30	10	90
40	65	35

Figure S174. *HPLC tracing of compound **II-86**.*

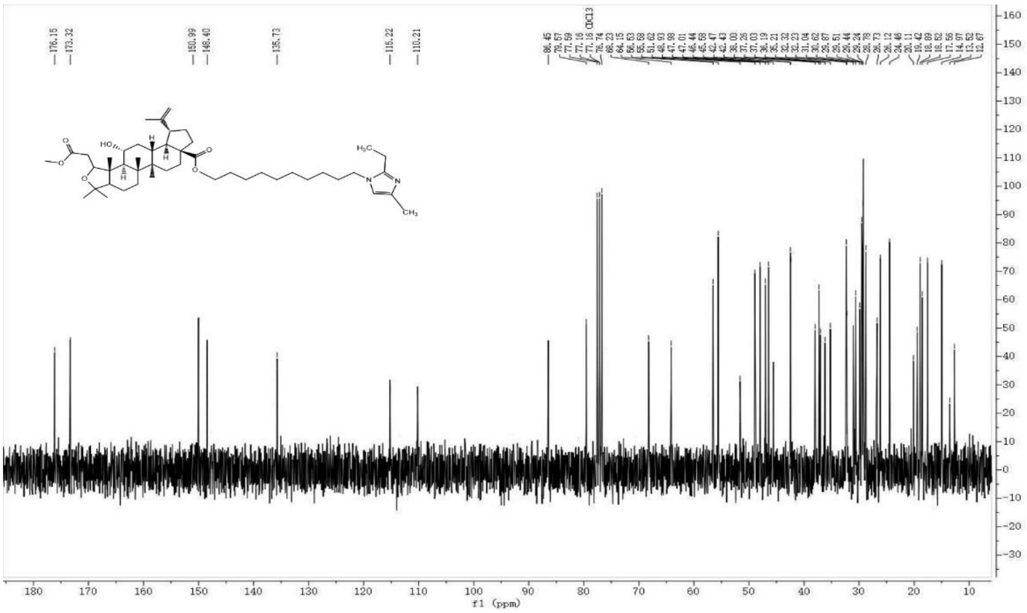

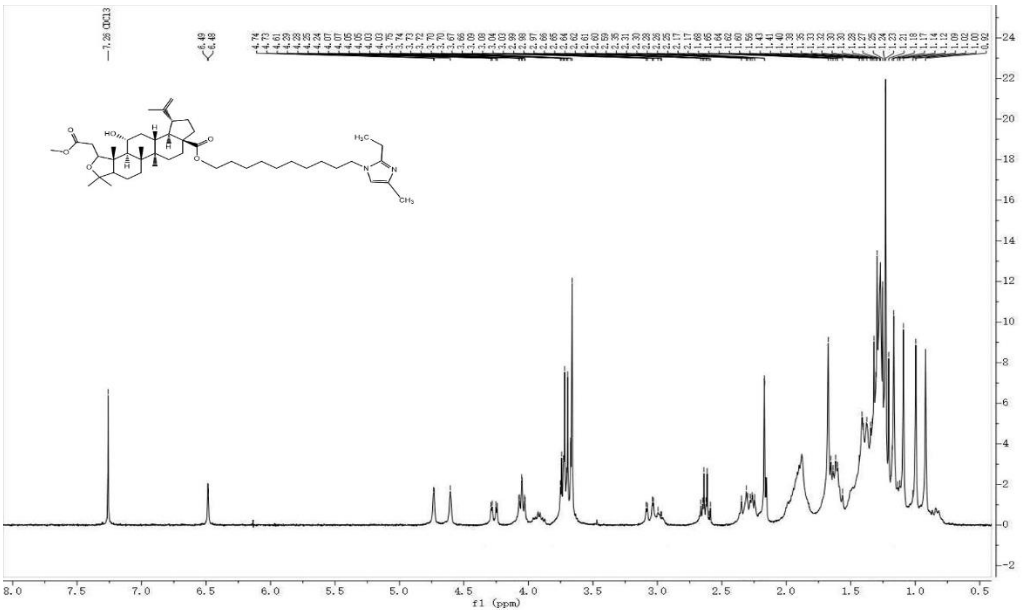

Figures S175. *^{13}C and ^{1}H NMR of compound **II-86**.*

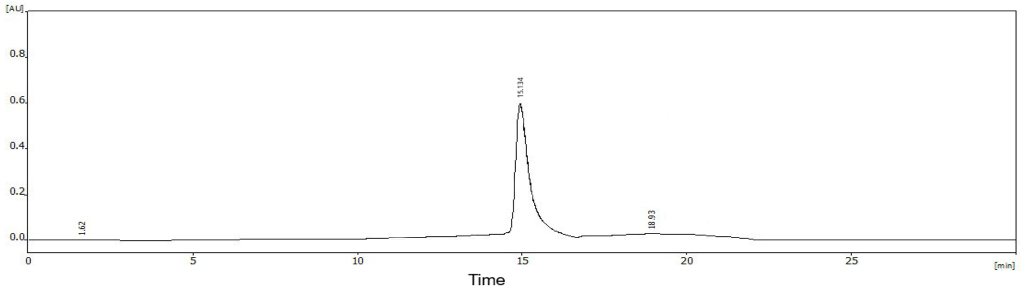

	RT	Area	Area %	Height
1	1.623	73.915	0.3	0.853
2	15.134	28045.495	95.7	600.556
3	18.930	1172.225	4.0	29.443

Time	A (water,0.5% Phosphoric acid)	B (Acetonitrile)
0	65	35
10	10	90
30	10	90
40	65	35

Figure S176. *HPLC tracing of compound **II-87**.*

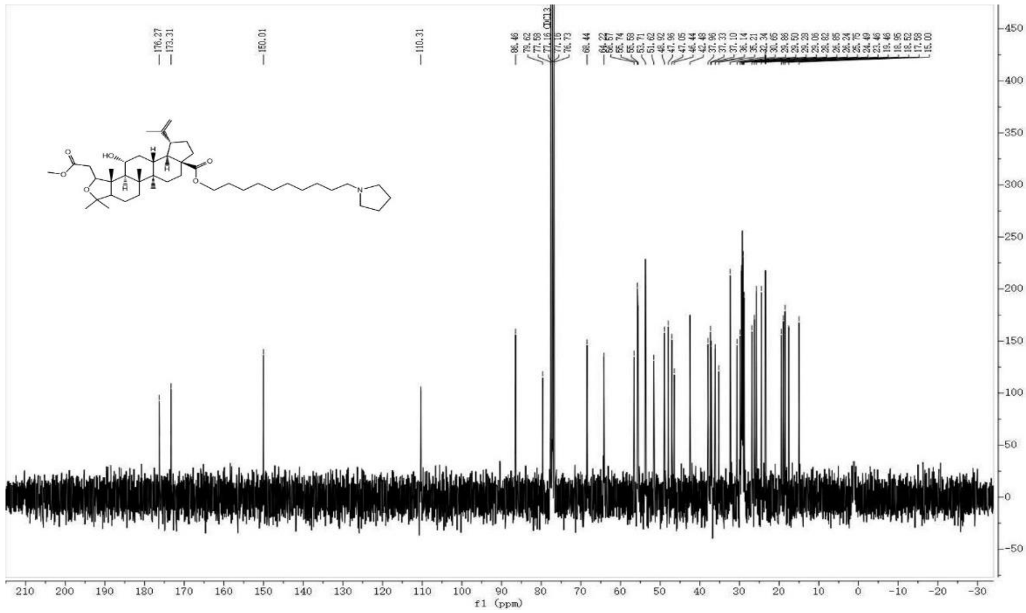

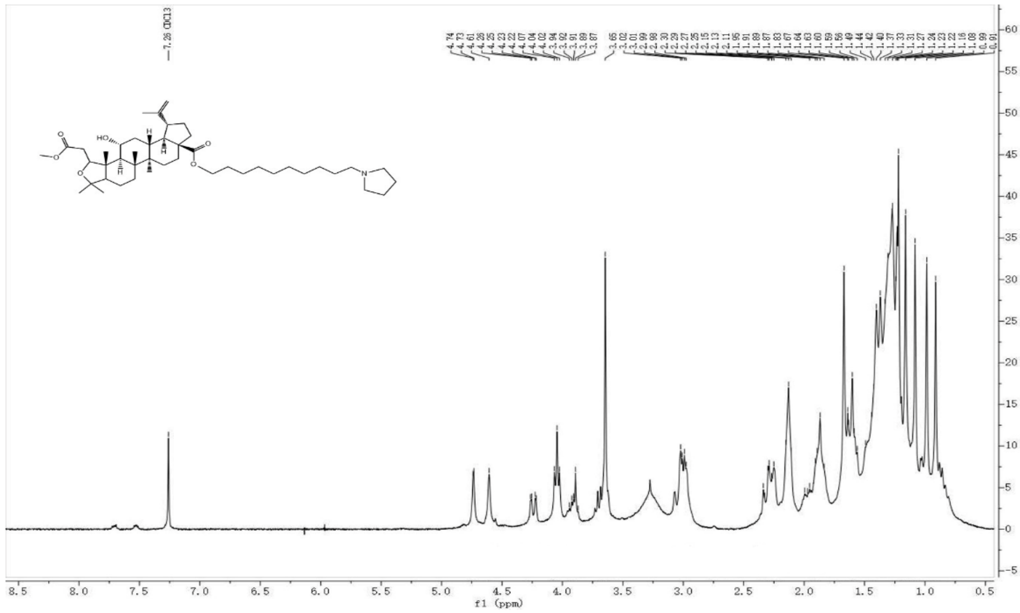

Figures S177. *^{13}C and ^{1}H NMR of compound **II-87**.*

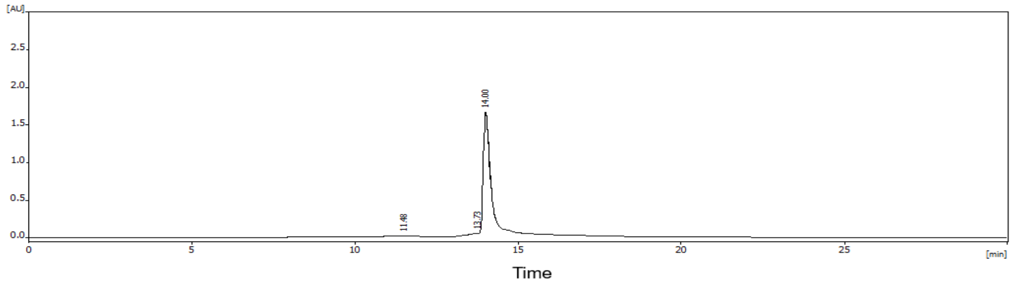

	RT	Area	Area %	Height
1	11.480	1377.409	3.1	21.583
2	13.730	88.865	0.2	57.843
3	14.003	42966.286	96.7	1671.033

Time	A (water,0.5% Phosphoric acid)	B (Acetonitrile)
0	65	35
10	10	90
30	10	90
40	65	35

Figure S178. *HPLC tracing of compound **II-88**.*

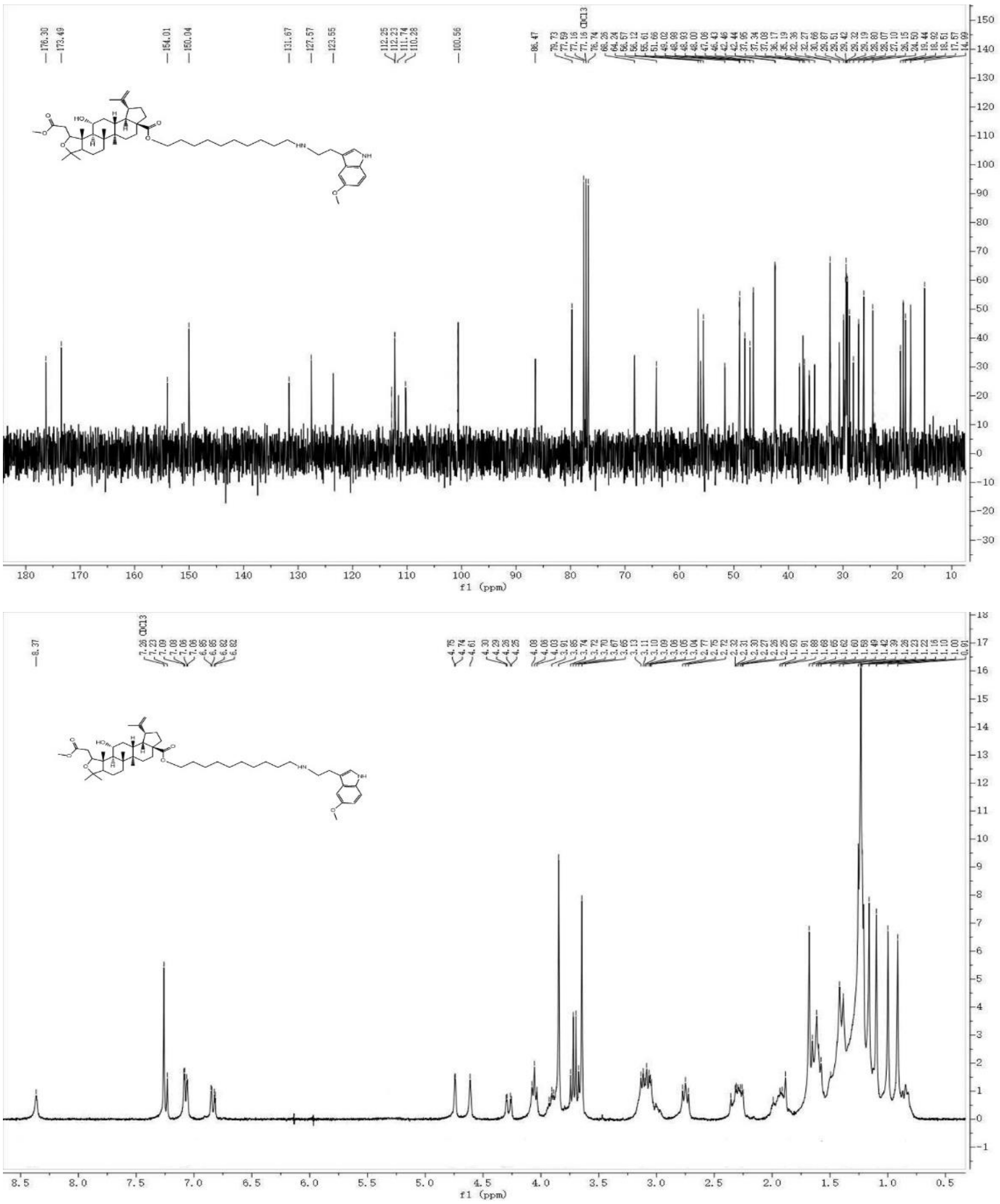

Figures S179. *^{13}C and ^{1}H NMR of compound **II-88**.*

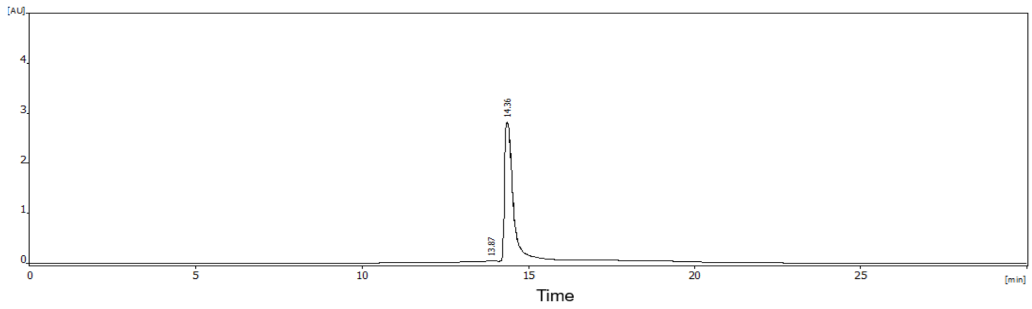

	RT	Area	Area %	Height
1	13.870	3780.658	5.0	41.077
2	14.363	71832.509	95.0	2824.672

Time	A (water,0.5% Phosphoric acid)	B (Acetonitrile)
0	65	35
10	10	90
30	10	90
40	65	35

Figure S180. *HPLC tracing of compound **II-89**.*

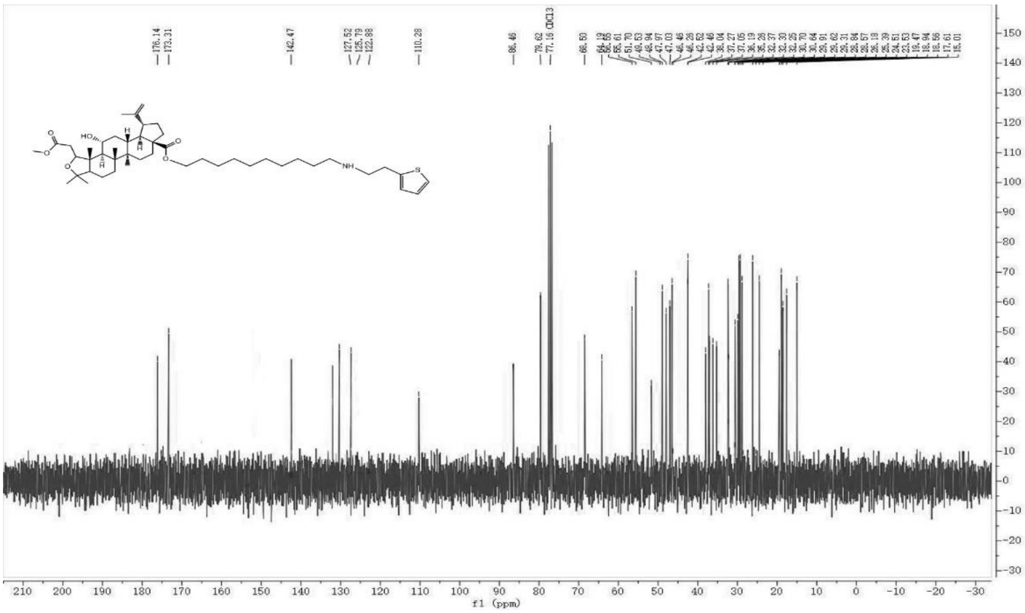

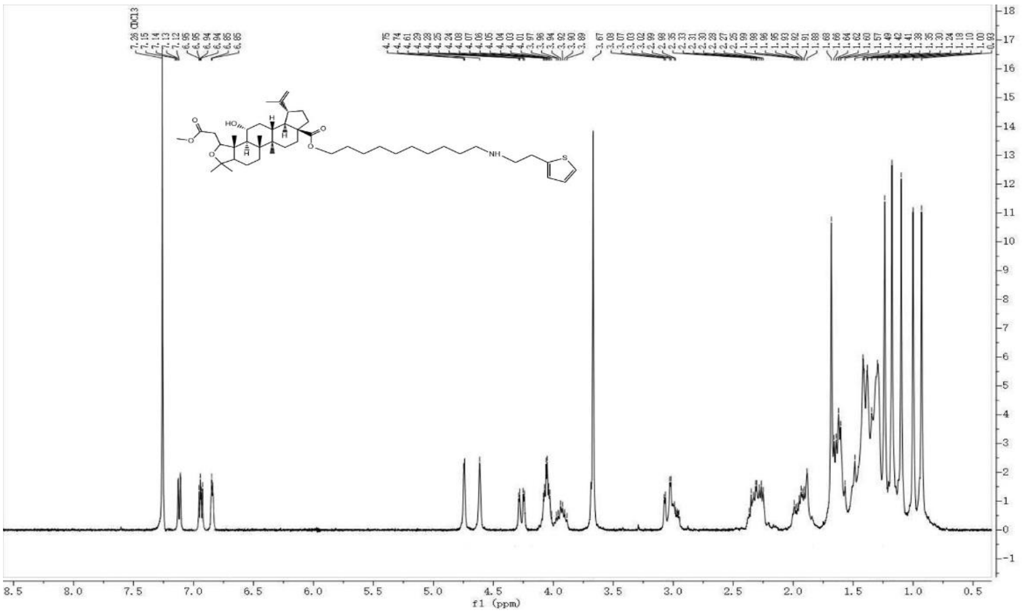

Figures S181. *^{13}C and ^{1}H NMR of compound **II-89**.*

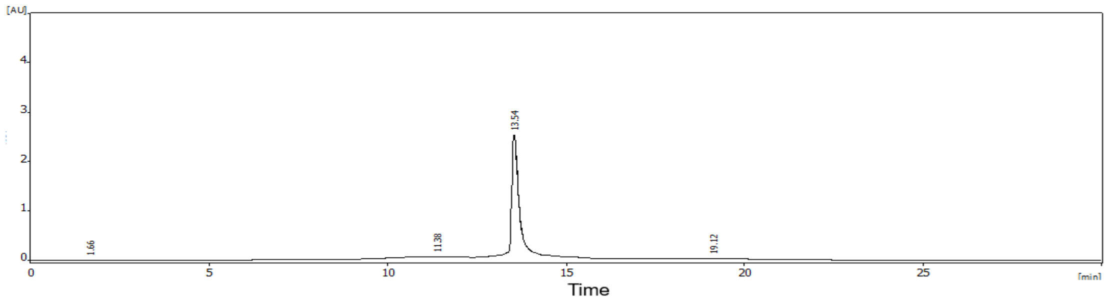

	RT	Area	Area %	Height
1	1.657	73.465	0.1	2.966
2	11.380	1689.688	2.3	61.153
3	13.543	70158.763	95.5	2535.254
4	19.123	1542.758	2.1	33.914

Time	A (water,0.5% Phosphoric acid)	B (Acetonitrile)
0	65	35
10	10	90
30	10	90
40	65	35

Figure S182. *HPLC tracing of compound **II-90**.*

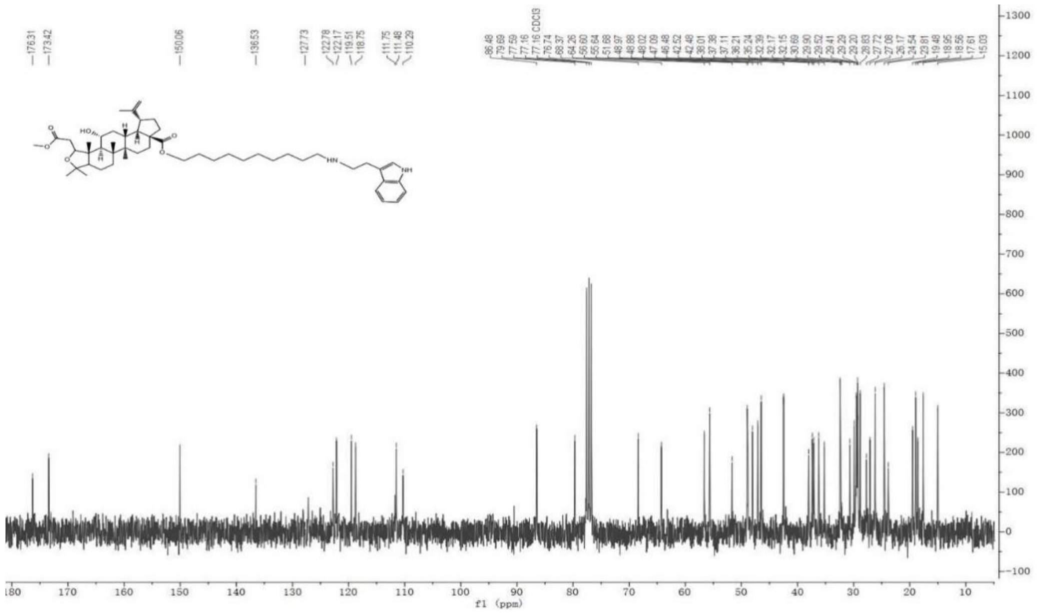

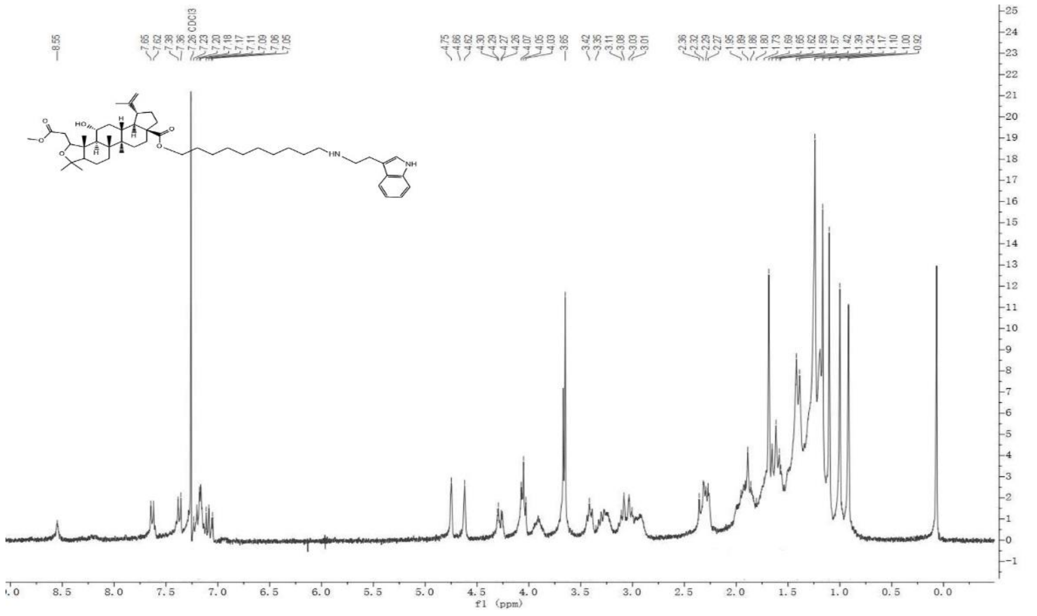

Figures S183. *^{13}C and ^{1}H NMR of compound **II-90**.*

Structural Identification

3-(4-formyl-1*H*-imidazol-1-yl)propyl(3*S*,3a*S*,3a1*S*,4*R*,7a*R*,8a*R*,8b*R*,9*R*,11a*S*,13a *R*, 13b*R*)-4-hydroxy-3a,13a,13b-trimethyl-6-oxo-3,9-di(prop-1-en-2-yl)octadecahydrocyclopenta[7,8]phenanthro[10,1-*bc*]oxepine-11a(1*H*)-carboxylate (Compound **I-1**). Chloroform / methanol = 60:1 / 50:1, white solid, purity of 97.1%, yield 52%. mp 106-107 °C. ^{1}H NMR (300 MHz, $CDCl_3$): δ ppm 9.88 (*d, J* = 3.3 Hz, 1H), 9.10 (*d, J* = 8.9 Hz, 1H), 8.30 (*d, J* = 6.3 Hz, 1H), 4.89 (*d, J* = 1.6 Hz, 1H), 4.81 (*d, J* = 2.3Hz, 1H), 4.75 (*d, J* = 2.3 Hz, 1H), 4.62 (*brs*, 1H), 4.44 (*q, J* = 9.0 Hz, 1H), 4.21−4.06 (*dt, J* = 17.3, 10.9, 5.6 Hz, 2H), 3.57 (*d, J* = 7.9 Hz, 1H), 3.49−3.45 *(t, J* = 6.4 Hz, 2H), 2.92 *(d, J* = 14.8 Hz, 1H), 2.95 (*d, J* =14.7 Hz, 1H), 2.73 (*d, J* = 7.9Hz, 1H), 2.67 (*d, J* = 7.9 Hz, 1H), 2.45 (*d, J* = 11.5 Hz, 1H), 2.36−2.27 (*m*, 1H), 2.27 (*d, J* = 2.6 Hz, 1H), 2.21−2.15 (*dt, J* = 6.1, 2.2 Hz, 2H), 1.94−1.81 (*m*, 6H), 1.73 (*s*, 3H), 1.67 (*s*,3H), 1.59 (*d, J* = 5.1 Hz, 1H), 1.51−1.10 (*m*, 8H), 1.05 (*s*, 3H), 1.01 (*s*,3H), 0.86 (*s*, 3H). ^{13}C NMR (75 MHz, $CDCl_3$): δ ppm185.67, 175.7, 173.43, 148.98, 146.89, 140.73, 138.66, 126, 114.37, 112.02, 75.63, 71.12, 60.52, 56.49, 50.07, 49.34, 47.1, 44.9, 43.88, 43.79, 41.95, 41.54, 37.71, 36.89, 34.88, 32.09, 31.97, 30.47, 30.05, 29.78, 29.28, 24.65, 22.73, 19.16, 19.01, 17.91, 13.84. HRMS Calcd for $C_{37}H_{52}N_2O_6$ $[M+H]^+$: 619.37526; found: 619.37358. See Figures S4 and S5.

3-(2-propyl-1*H*-imidazol-1-yl)propyl-chiisanogenin (Compound **I-2**). Chloroform / methanol = 65:1 / 60:1, white solid, purity of 97.0%, yield 65%. mp 156-157 °C. ^{1}H NMR (300 MHz, $CDCl_3$): δ ppm 6.51 (*d, J* = 3.6 Hz, 1H), 4.85 (*d, J* = 1.6 Hz, 1H), 4.83 (*d, J* = 2.3 Hz, 1H), 4.76 (*d, J* = 2.3 Hz, 1H), 4.65 (*brs*, 1H), 4.52 *(q, J* =8.9 Hz, 1H), 4.11−3.84 (*dt, J* = 17.3, 10.9, 5.6 Hz, 2H), 3.57 (*d, J* = 7.9 Hz, 1H), 3.49−3.45 (*t, J* = 6.4 Hz, 2H), 2.99 (*dt, J* = 13.4 Hz, 1H), 2.93 (*d, J* = 14.5 Hz, 1H), 2.74 (*d, J* = 7.9 Hz, 1H), 2.67 (*d, J* = 7.9 Hz, 1H), 2.47 (*d, J* = 11.4 Hz, 1H), 2.36−2.27 (*m*, 7H), 2.26(*d, J* = 2.6 Hz, 1H), 2.21− 2.15 (*dt, J* = 6.1, 2.2 Hz, 2H), 1.94−1.81 (*m*, 6H), 1.73 (*s*, 3H), 1.68 (*s*, 3H), 1.55 (*d, J* = 5.1 Hz, 1H), 1.64−1.13 (*m*, 8H), 1.06 (*s*, 3H), 1.01 (*s*, 3H), 0.88 (*s*, 3H).^{13}C NMR (75 MHz, $CDCl_3$): δ ppm 175.44, 173.23, 149.06, 147, 146.54, 136.15, 115.44, 114.33, 111.28, 75.63, 71.2, 60.76, 56.49, 49.38, 47.17, 47.09, 43.92, 43.87, 42.71, 42, 41.58, 37.71, 36.91, 34.9, 32.03, 30.47, 30.13, 29.88, 29.81, 29.3, 24.66, 22.64, 19.18, 18.99, 17.96, 13.86, 13.41, 12.86. HRMS Calcd for $C_{39}H_{58}N_2O_5$ $[M+H]^+$: 633.42730; found: 633.42565. See Figures S6 and S7.

3-(2,4-dimethyl-1*H*-imidazol-1-yl)propyl-chiisanogenin (Compound **I-3**). Chloroform / methanol = 55:1 / 45:1, separated to give a white solid, purity of 97.8%, yield 75%. mp 152-151 °C. ^{1}H NMR (300 MHz, $CDCl_3$): δ ppm 6.51 (*d, J* = 3.6 Hz, 1H), 4.85 (*d, J* = 1.6 Hz, 1H), 4.83 (*d, J* = 2.3 Hz, 1H), 4.76 (*d, J* = 2.3 Hz, 1H), 4.65 (*brs*, 1H), 4.52

(*q, J* =8.9 Hz, 1H), 4.11−3.84 (*dt, J* = 17.3, 10.9, 5.6 Hz, 2H), 3.57 (*d, J* = 7.9 Hz, 1H), 3.49−3.45 (*t, J* = 6.4 Hz, 2H), 2.99 (*dt, J* = 13.4 Hz, 1H), 2.93 (*d, J* = 14.5 Hz, 1H), 2.74 (*d, J* = 7.9 Hz, 1H), 2.67 (*d, J* = 7.9 Hz, 1H), 2.47 (*d, J* = 11.4 Hz, 1H), 2.36−2.27 (*m*, 7H), 2.26(*d, J* = 2.6 Hz, 1H), 2.21− 2.15 (*dt, J* = 6.1, 2.2 Hz, 2H), 1.94−1.81 (*m*, 6H), 1.73 (*s*, 3H), 1.68 (*s*, 3H), 1.55 (*d, J* = 5.1 Hz, 1H), 1.64−1.13 (*m*, 8H), 1.06 (*s*, 3H), 1.01 (*s*, 3H), 0.88 (*s*, 3H).^{13}C NMR (75 MHz, $CDCl_3$): δ ppm 175.44, 173.23, 149.06, 147, 146.54, 136.15, 115.44, 114.33, 111.28, 75.63, 71.2, 60.76, 56.49, 49.38, 47.17, 47.09, 43.92, 43.87, 42.71, 42, 41.58, 37.71, 36.91, 34.9, 32.03, 30.47, 30.13, 29.88, 29.81, 29.3, 24.66, 22.64, 19.18, 18.99, 17.96, 13.86, 13.41, 12.86. HRMS Calcd for $C_{38}H_{56}N_2O_5$ $[M+H]^+$: 619.41165; found: 619.41687. See Figures S8 and S9.

3-(4-bromo-1*H*-imidazol-1-yl)propyl-chiisanogenin (Compound **I-4**). Chloroform / methanol = 50:1 / 45:1, white solid, purity of 98.1%, yield 63%. mp 100-101 °C. ^{1}H NMR (300 MHz, $CDCl_3$): δ ppm 7.39 (*d, J* = 5.9 Hz, 1H), 6.99 (*d, J* = 9.5 Hz, 1H), 4.85 (*d, J* = 1.6 Hz, 1H), 4.83 (*d, J* = 2.3 Hz,1H), 4.76 (*d, J* = 2.3 Hz,1H), 4.63 (*brs*, 1H), 4.53 (*q, J* =8.9 Hz, 1H), 4.18−4.07 (*dt, J* = 17.3, 10.9, 5.6 Hz, 2H), 3.58 (*d, J* = 7.9 Hz, 1H), 3.03−2.94 (*t, J* = 6.4 Hz, 2H), 2.92 (*dt, J* = 13.4 Hz, 1H), 2.95 (*d, J* = 14.7 Hz, 1H), 2.75 (*d, J* = 7.9 Hz, 1H), 2.67 (*d, J* = 7.9 Hz, 1H), 2.47 (*d, J* = 11.5 Hz, 1H), 2.32−2.27 (*m*, 1H), 2.23 (*d, J* = 2.6 Hz, 1H), 2.28− 2.07 (*dt, J* = 6.1, 2.2 Hz, 2H), 1.94−1.83 (*m*, 6H), 1.73 (*s*, 3H), 1.67 (*s*, 3H), 1.57 (*d, J* = 5.1 Hz, 1H), 1.61−1.14 (*m*, 8H), 1.05 (*s*, 3H), 1.01 (*s*, 3H), 0.87 (*s*, 3H). ^{13}C NMR (75 MHz, $CDCl_3$): δ ppm 175.69, 173.38, 149.03, 146.92, 136.99, 118.36, 115.37, 114.4 ,111.18, 75.65, 71.15, 60.27, 56.49, 50.12, 49.37, 47.13, 44.67, 43.91, 43.82, 41.97, 41.56, 37.7, 36.89, 34.89, 32.61, 32.12, 31.99, 30.48, 30.23, 29.3, 24.68, 22.73, 19.17, 19.02, 17.93, 13.85. HRMS Calcd for $C_{36}H_{51}BrN_2O_5$ $[M+H]^+$: 669.29086; found: 669.29678. See Figures S10 and S11.

3-(2-ethyl-4-methyl-1*H*-imidazol-1-yl)propyl-chiisanogenin (Compound **I-5**). Chloroform / methanol = 45:1 / 40:1, white solid, purity of 98.4%, yield 65%. mp 116-117 °C. ^{1}H NMR (300 MHz, $CDCl_3$):δ ppm 6.49 (*brs*, 1H), 4.84 (*d, J* = 1.6 Hz, 1H), 4.82 (*d, J* = 2.3 Hz, 1H), 4.76 (*d, J* = 2.3 Hz, 1H), 4.62 (*brs*, 1H), 4.51 (*q, J* = 8.9 Hz, 1H), 4.07−3.71 (*dt, J* = 17.3, 10.9, 5.5 Hz, 2H), 3.56 (*d, J* = 7.9 Hz, 1H), 3.49−3.45 (*t, J* = 6.3 Hz, 2H), 2.97 (*dt, J* = 13.4 Hz, 1H), 2.97 (*d, J* = 14.7 Hz, 1H), 2.75 (*d, J* = 7.9 Hz, 1H), 2.66 (*d, J* = 7.9 Hz, 1H), 2.41 (*d, J* = 11.5 Hz, 1H), 2.36−2.28 (*m*, 3H), 2.23 (*d, J* = 2.6 Hz, 1H), 2.21− 2.16 (*dt, J* = 6.1, 2.2 Hz, 2H), 2.17 (*d, J* = 1.0 Hz, 3H)

2.10−1.83 (*m*, 6H), 2.17 (*d, J* = 1.0 Hz, 3H), 1.73 (*s*, 3H), 1.67 (*s*, 3H), 1.52 (*d, J* = 5.0 Hz, 1H), 1.61−1.09 (*m*, 8H), 1.06 (*s*, 3H), 1.00 (*s*, 3H), 0.87 (*s*, 3H). ^{13}C NMR (75 MHz, $CDCl_3$): δ ppm 175.72, 173.43, 149.05, 148.48, 146.92, 136.27, 115.18, 114.25, 111.12, 75.65, 71.05, 60.68, 56.45, 50.05, 49.33, 47.13, 46.86, 43.75, 42.85, 41.95, 41.52, 37.66, 36.88, 34.86, 32.55, 32.1, 31.98, 30.43, 30.31, 29.26, 24.64, 22.69, 20.07, 19.12, 18.95, 17.9, 13.81, 13.55, 12.58. HRMS Calcd for $C_{39}H_{58}N_2O_5$ $[M+H]^+$: 633.42730; found: 633.42564. See Figures S12 and S13.

3-(pyrrolidin-1-yl)propyl-chiisanogenin (Compound **I-6**). Chloroform / methanol = 45:1 / 35:1, s white solid, purity of 97.5%, yield 62%. mp 187-188 °C. ^{1}H NMR (300 MHz, CDCl3): δ ppm 4.89 (*d, J* = 1.6 Hz, 1H), 4.83 (*d, J* = 2.3Hz, 1H), 4.75 (*d, J* = 2.3 Hz, 1H), 4.62 (*brs*, 1H), 4.51 (*q, J* = 8.9Hz, 1H), 4.13−4.09 (*dt, J* = 17.3, 10.9, 5.8 Hz, 2H), 3.55 (*d, J* = 7.8 Hz, 1H), 3.73−3.66 (*t, J* = 6.4 Hz, 2H), 2.97 (*dt, J* = 13.4 Hz, 1H), 2.91 (*d, J* =14.7 Hz, 1H), 2.71 (*d, J* = 8 Hz, 1H), 2.66 (*d, J* = 7.9 Hz, 1H), 2.47 (*d, J* = 11.5 Hz, 1H), 2.39−2.23 (*m*, 5H), 2.23 (*d, J* = 2.6 Hz, 1H),1.95-1.81(*m*, 6H), 1.94−1.81 (*m*, 6H), 1.72 (*s*, 3H), 1.66 (*s*,3H), 1.52 (*d, J* = 5.1 Hz, 1H), 1.51−1.10 (*m*, 8H), 1.04 (*s*, 3H) , 0.99 (*s*, 3H), 0.87(*s*, 3H). ^{13}C NMR (75 MHz, $CDCl_3$): δ ppm 176.08, 173.55, 149.32, 147.03, 114.24, 111, 75.84, 71.14, 62.47, 58.46, 56.44, 54.29, 53.19, 50.17, 49.33, 47.22, 43.87, 43.79, 41.95, 41.57, 37.67, 36.94, 34.93, 32.61, 32.1, 32.06, 30.52, 29.21, 27.99, 24.68, 23.51, 22.68, 19.15, 18.99, 18.5, 17.94, 13.85. HRMS Calcd for $C_{37}H_{57}NO_5$ $[M+H]^+$: 594.41640; found: 594.41467. See Figures S14 and S15.

3-((2-(5-methoxy-1*H*-indol-3-yl)ethyl)amino)propyl-chiisanogenin (Compound **I-7**). Chloroform / methanol = 35:1 / 30:1, white solid, purity of 98.3%, yield 68%. mp 119-120 °C. ^{1}H NMR (300 MHz, $CDCl_3$): δ ppm 8.35 – 8.12 (*t, J* = 14.7 Hz, 1H), 7.01 (*dd, J* = 11.6, 6.8 Hz, 1H), 6.84 (*d, J* = 8.2 Hz, 1H), 5.29 (*d, J* = 7.2 Hz, 1H), 4.85 (*d, J* = 1.6 Hz, 1H), 4.83 (*d, J* = 2.3 Hz, 1H), 4.74 (*d, J* = 2.2 Hz, 1H), 4.62 (*brs*, 1H), 4.43 (*q, J* = 8.9 Hz, 1H), 4.16−4.07 (*dt, J* = 17.3, 10.9, 5.6 Hz, 2H), 3.85(*s*,3H), 3.52 (*d, J* = 7.9 Hz, 1H), 3.49−3.45 (*t, J* = 6.3 Hz, 2H), 2.97 (*dt, J* = 13.0 Hz, 1H), 2.92 (*d, J* = 14.5 Hz, 1H), 2.75 (*d, J* = 7.8 Hz, 1H), 2.71 (*d, J* = 7.7 Hz, 1H), 2.42 (*d, J* = 11.6 Hz, 1H), 2.36−2.27 (*m*, 7H), 2.23 (*d, J* = 2.6 Hz, 1H), 2.21− 2.15 (*dt, J* = 6.1, 2.2 Hz, 2H), 1.94−1.81 (*m*, 6H), 1.73 (*s*, 3H), 1.67 (*s*, 3H), 1.55 (*d, J* = 5.1 Hz, 1H), 1.51−1.10 (*m*, 8H), 0.96 (*s*, 3H), 0.88 (*s*, 3H), 0.84 (*s*, 3H).^{13}C NMR (75 MHz, $CDCl_3$): δ ppm 176.08, 173.89, 153.87, 149.34, 146.89, 131.74, 127.75, 123.16, 114.16, 112.56, 112.1, 110.88,

110.26, 100.72, 75.87, 70.95, 62.25, 56.36, 56, 49.79, 49.26, 48.88, 46.92, 46.46, 43.75, 43.64, 41.85, 41.47, 37.22, 36.88, 34.88, 32, 30.63, 30.48, 29.74, 29.14, 28.92, 25.41, 24.59, 22.73, 18.97, 18.45, 17.77, 13.71. HRMS Calcd for $C_{44}H_{62}N_2O_6$ $[M+H]^+$: 713.45351; found: 713.45265. See Figures S16 and S17.

3-((2-(thiophen-2-yl)ethyl)amino)propyl-chiisanogenin (Compound **I-8**). Chloroform / methanol = 45:1/40:1, white solid, purity of 98.6%, yield 57%. mp 124-125 °C. ^{1}H NMR (300 MHz, $CDCl_3$): δ ppm7.14 (*d, J* = 6.3 Hz, 1H) 6.84 (*d, J*= 6.8 Hz, 1H), 6.92 (*d, J* = 5.1 Hz,1H), 4.84 (*d, J* = 1.6 Hz, 1H), 4.83 (*d, J* = 2.3Hz, 1H), 4.76 (*d, J* = 2.5 Hz, 1H), 4.62 (*brs*, 1H), 4.51 (*q, J* = 9.0 Hz, 1H),4.13−4.09 (*dt, J* = 17.3, 10.9, 5.6 Hz, 2H), 3.55 (*d, J* = 7.9 Hz, 1H),3.77−3.66 (*t, J* = 6.4 Hz, 2H), 2.97 (*dt, J* = 13.4 Hz, 1H), 2.91 (*d, J* =14.7 Hz, 1H), 2.71 (*d, J* = 7.9 Hz, 1H), 2.66 *(d, J* = 8.0 Hz, 1H), 2.47 (*d, J* = 11.5 Hz, 1H), 2.39−2.23 (*m*, 5H), 2.23 (*d, J* = 2.6 Hz, 1H), 2.22−2.15 (*dt, J* = 6.1, 2.2 Hz, 2H), 1.95−1.81 (*m*, 6H), 1.73 (*s*, 3H), 1.67 (*s*,3H), 1.55 (*d, J* = 5.1 Hz, 1H), 1.51−1.10 (*m*, 9H), 1.04 (*s*, 3H), 1.00 (*s*, 3H), 0.88 (*s*, 3H).^{13}C NMR (75 MHz, $CDCl_3$): δ ppm176.07, 173.38, 149.29, 147.03, 142.33, 127.01, 125.17, 123.76, 114.28, 111.03, 75.76, 71.21, 62.28, 56.42, 51.16, 50.24, 49.33, 48.91, 47.21, 46.39, 43.89, 43.82, 41.95, 41.56, 37.67, 36.95, 34.91, 32.59, 32.05, 30.52, 30.35, 29.25, 24.66, 24.51, 22.61, 19.18, 19, 17.97, 13.85. HRMS Calcd for $C_{39}H_{57}NO_5S$ $[M+H]^+$: 650.38847; found: 650.38635. See Figures S18 and S19.

3-((2-(1*H*-indol-3-yl)ethyl)amino)propyl-chiisanogenin (Compound **I-9**). Chloroform / methanol = 30:1 / 25:1, white solid, purity of 97.2, yield 67%. mp 144-145 °C. ^{1}H NMR (300 MHz, $CDCl_3$): δ ppm 8.04 (*m*, 1H), 7.62 (*d, J* = 8.4 Hz, 1H), 7.38(*d, J* = 8.0 Hz, 1H), 7.21 (*t, J* = 8.2 Hz, 1H) 7.11 (*t, J* = 8.0 Hz, 1H), 4.87 (*d, J* = 1.6 Hz, 1H), 4.83 (*d, J* = 2.3Hz, 1H), 4.75 (*d, J* = 2.3 Hz, 1H), 4.62 (*brs*, 1H), 4.47 (*q, J* = 9.0 Hz, 1H), 4.28−4.15 (*dt, J* = 17.3, 10.9, 5.6 Hz, 4H), 3.54 (*d, J* = 7.9 Hz, 1H), 3.49−3.45 (*t, J* = 6.4 Hz, 2H), 2.99 (*m*, 2H), 2.92 (*dt, J* = 11.1, 4.4 Hz, 1H), 2.88 (*m*, 2H), 2.77 (*m*, 2H), 2.68 (*d, J* = 7.9 Hz, 1H), 2.64 (*d, J* = 7.9 Hz, 1H),2.43 (*d, J* = 11.5 Hz, 1H), 2.36−2.27 (*m*, 1H), 2.27 (*d, J* = 2.6 Hz, 1H), 2.21−2.15 (*dt, J* = 6.1, 2.2 Hz, 2H), 1.94−1.81 (*m*, 5H), 1.78 (*d, J* = 6.8 Hz, 1H), 1.72 (*s*, 3H), 1.67 (*s*,3H), 1.55 (*d, J* = 5.1 Hz, 1H), 1.51−1.10 (*m*, 8H), 1.04 (*s*, 3H), 0.96 (*s*, 3H), 0.86(*s*,3H). ^{13}C NMR (75 MHz, $CDCl_3$): δ ppm 176.09, 173.27, 149.3, 147.03, 136.51, 127.49, 122.15, 122.06, 119.38, 118.87, 114.23, 113.89, 111.31, 110.99, 77.12, 71.18, 62.39, 56.41, 50.28, 50.05, 49.33, 47.24,

46.6, 43.88, 43.82, 41.93, 41.55, 37.6, 36.93,34.95 ,32.58, 32.07, 30.53, 29.32, 29.19, 29.13, 25.79, 24.63, 22.65, 19.09, 18.97, 17.91, 13.83. HRMS Calcd for $C_{43}H_{60}N_2O_5$ $[M+H]^+$: 683.44295; found: 683.44425. See Figures S20 and S21.

4-(4-formyl-1*H*-imidazol-1-yl)butyl-chiisanogenin (Compound **I-10**). Chloroform / methanol = 60:1/50:1, white solid, purity of 98.6%, yield 71%. mp 124-125 °C. ^{1}H NMR (300 MHz, $CDCl_3$): δ ppm 9.95 (*d, J* = 2.9 Hz, 1H), 9.30 (*d, J* = 4.5 Hz, 1H), 8.05 (*d, J* = 6.5 Hz, 1H), 4.85 (*d, J* = 1.8 Hz, 1H), 4.83 (*d, J* = 1.8 Hz, 1H), 4.76 (*d, J* = 2.2 Hz, 1H), 4.62 (*brs*, 1H), 4.51 (*q, J* = 9.0 Hz, 1H), 4.13−4.08 (*dt, J* = 6.1, 1.9 Hz, 2H), 3.55 (*d, J* = 7.8 Hz, 1H), 3.46−3.42 (*t, J* = 6.5 Hz, 2H), 2.98 (*dt, J* = 11.5, 7.3, 5.2 Hz, 1H), 2.95 (*d, J* = 14.8 Hz, 1H), 2.75 (*d, J* = 8.1 Hz, 1H), 2.68 (*d, J* = 7.9 Hz, 1H), 2.48 (*d, J* = 12.4 Hz, 1H), 2.39 (*d, J* = 4.6 Hz, 1H), 2.32−2.25 (*m*, 1H), 2.24 (*d, J* = 3.8 Hz, 1H), 1.98−1.96 (*m*, 2H), 1.95−1.93 (*dt, J* = 8.2, 6.6, 2.5 Hz, 2H), 1.91−1.80 (*m*, 5H), 1.78 (*d, J* = 1.6 Hz, 1H), 1.73 (*s*, 3H), 1.68 (*s*, 3H), 1.55 (*d, J* = 4.5 Hz, 1H), 1.60−1.09 (*m*, 8H), 1.06 (*s*, 3H), 1.02 (*s*, 3H), 0.90 (*s*, 3H). ^{13}C NMR (75 MHz, $CDCl_3$): δ ppm 185.41, 176.76, 173.3, 149.17, 146.92, 139.28, 138.91, 127.38, 114.36, 111.06, 75.72, 71.17, 63.38, 56.45, 49.34, 47.67, 47.16, 43.9, 43.8, 41.95, 41.56, 39.5, 37.73, 36.93, 34.91, 32.64, 32.06, 30.51, 29.8, 29.23, 27.05, 26.47, 25.87, 24.69, 19.18, 19.03, 17.9, 13.85. HRMS Calcd for $C_{38}H_{54}N_2O_6$ $[M+H]^+$: 633.39091; found: 633.39254. See Figures S22 and S23.

4-(2-propyl-1*H*-imidazol-1-yl)butyl-chiisanogenin (Compound **I-11**). Chloroform / methanol = 60:1 / 55:1, white solid, purity of 96.2, yield 60%. mp 135-136 °C. ^{1}HNMR(300 MHz, $CDCl_3$): δ ppm 7.34 (*m*, 1H), 7.04 (*m*, 1H), 4.88 (*d, J* = 1.9 Hz, 1H), 4.83 (*d, J* = 1.8 Hz, 1H), 4.76 (*d, J* = 2.2 Hz,1H), 4.62 (*brs*, 1H), 4.52 (*q, J* = 8.9 Hz, 1H), 4.13−4.08 (*dt, J* = 6.1, 1.9Hz, 2H), 3.57 (*d, J* = 7.8 Hz, 1H), 3.46−3.42 (*t, J* = 6.5 Hz, 2H), 2.92(*dt, J* = 11.5, 7.3, 5.2 Hz, 1H), 2.95 (*d, J* = 14.8 Hz, 1H), 2.75 (*d, J* = 8.1Hz, 1H), 2.69 (*d, J* = 7.9 Hz, 1H), 2.48 (*d, J* = 12.4 Hz, 1H), 2.39 (*d, J* =4.6 Hz, 1H), 2.32−2.25 (*m*, 1H), 2.24 (*d, J* = 3.8 Hz, 1H), 1.98−1.96(*m*, 4H), 1.95−1.93 (*dt, J* = 8.2, 6.6, 2.5 Hz, 2H), 1.91−1.80 (*m*, 4H),1.78 (*d, J* = 1.6 Hz, 1H), 1.73 (*s*, 3H), 1.67 (*s*, 3H), 1.55 (*d, J* = 4.5 Hz,1H), 1.60−1.09 (*m*, 10H), 1.05 (*s*, 3H), 1.03 (*s*, 3H),0.97(*s*,3H) 0.87 (*s*, 3H). ^{13}C NMR (75 MHz, $CDCl_3$): δ ppm 175.99, 173.35, 149.16, 147.94, 146.97, 126.04, 118.97, 114.3, 111.09, 75.64, 71.14, 63.11, 56.44, 49.33, 47.21, 45.73, 43.89, 43.8, 41.96, 41.55, 37.69, 36.92, 34.91, 32.59, 32.59, 32.11, 30.49, 29.28, 29.23, 28.28,

27.68, 26.03, 24.66, 21.78, 21.52, 19.15, 18.97, 17.9, 14.04, 13.84. HRMS Calcd for $C_{40}H_{60}N_2O_5$ $[M+H]^+$: 647.44295; found: 647.44546. See Figures S24 and S25.

3-(2,4-dimethyl-1*H*-imidazol-1-yl)propyl-chiisanogenin (Compound **I-12**). Chloroform / methanol = 55:1 / 45:1, white solid, purity of 98.1% purity of 97.1%, yield 62%. mp 137-148 °C. ^{1}HNMR(300 MHz, $CDCl_3$): δ ppm 6.5 (*m*, 1H), 4.84 (*d, J* = 1.8 Hz, 1H), 4.82 (*d, J* = 2.3 Hz, 1H), 4.72 (*d, J* = 2.2 Hz, 1H), 4.62 (*brs*, 1H), 4.41 (*q, J* = 9.0 Hz, 1H), 4.13−4.08 (*dt, J* = 6.1, 1.9Hz, 2H), 3.56 (*d, J* = 7.8 Hz, 1H), 3.46−3.42 (*t, J* = 6.5 Hz, 2H), 2.94(*dt, J* = 11.5, 7.3, 5.2 Hz, 1H), 2.95 (*d, J* = 14.8 Hz, 1H), 2.72 (*d, J* = 8.1 Hz, 1H), 2.68 (*d, J* = 7.9 Hz, 1H), 2.43 (*d, J* = 12.4 Hz, 1H), 2.35(*s*,3H), 2.34 (*d, J* =4.6 Hz, 1H), 2.32−2.25 (*m*, 1H), 2.24 (*d, J* = 3.8 Hz, 1H), 1.98−1.96(*m*, 2H), 1.95−1.93 (*dt, J* = 8.2, 6.6, 2.5 Hz, 2H), 1.91−1.80 (*m*, 2H), 1.78(*d, J* = 1.6 Hz, 1H), 1.73 (*s*, 3H), 1.68 (*s*, 3H), 1.55 (*d, J* = 4.5 Hz, 1H), 1.60−1.09 (*m*, 10H),1.04(*s*, 3H), 1.00 (*s*, 3H), 0.86 (*s*, 3H). ^{13}C NMR (75 MHz, $CDCl_3$): δ ppm 176.01, 173.37, 149.2, 146.98, 143.76, 140.95, 115.37, 113.57, 112.42, 75.66, 71.13, 63.22, 56.44, 50.14, 49.34, 47.22, 45.55, 43.89, 43.8, 41.96, 41.56, 37.69, 36.92, 34.92, 32.6, 32, 30.49, 29.88, 29.23, 27.51, 26.02, 24.68, 22.91, 19.14, 18.97, 17.89, 13.84, 13.26, 13.2. HRMS Calcd for $C_{39}H_{58}N_2O_5$ $[M+H]^+$: 633.42730; found: 633.42564. See Figures S26 and S27.

4-(4-bromo-1*H*-imidazol-1-yl)butyl-chiisanogenin (Compound **I-13**). Chloroform / methanol = 50:1/40:1, white solid, purity of 98.4%, yield 59%. mp 136-137 °C. ^{1}H NMR (300 MHz, $CDCl_3$): δ ppm 7.36 (*d, J* = 6.3 Hz, 1H), 6.92 (*d, J* = 5.4 Hz, 1H), 4.85 (*d, J* = 1.9 Hz, 1H), 4.83 (*d, J* = 1.8 Hz, 1H), 4.74 (*d, J* = 2.3 Hz, 1H), 4.62 (*brs*, 1H), 4.52 (*q, J* = 9.0 Hz, 1H), 4.13−4.00 (*dt, J* = 6.1, 1.9 Hz, 2H), 3.58 (*d, J* = 7.8 Hz, 1H), 3.02−2.96 (*t, J* = 6.5 Hz, 2H), 2.92 (*dt, J* = 11.5, 7.3, 5.2 Hz, 1H), 2.95 (*d, J* = 14.8 Hz, 1H), 2.71 (d, J = 8.1 Hz, 1H), 2.68 (*d, J* = 7.9 Hz, 1H), 2.48 (*d, J* = 12.4 Hz, 1H), 2.39 (*d, J* = 4.6 Hz, 1H), 2.32−2.25 (*m*, 1H), 2.22 (*d, J* = 3.8 Hz, 1H), 2.08−1.93 (*m*, 2H), 1.95−1.93 (*dt, J* = 8.2, 6.6, 2.5 Hz, 2H), 1.91−1.80 (*m*, 2H), 1.78 (*d, J* = 1.6 Hz, 1H), 1.73 (*s*, 3H), 1.66 (*s*, 3H), 1.58 (*d, J* = 4.5 Hz, 1H), 1.63−1.06 (*m*, 10H), 1.04 (*s*, 3H), 1.01 (*s*, 3H), 0.85 (*s*, 3H). ^{13}C NMR (75 MHz, $CDCl_3$): δ ppm 175.97, 173.46, 149.19, 146.94, 136.89, 129.14, 118.11, 114.34, 111.07, 75.69, 71.13, 62.96, 56.43, 50.08, 49.33, 47.32, 47.16, 43.88, 43.77, 41.94, 41.53, 37.71, 36.93, 34.91, 32.59, 32.09,

31.99, 30.5, 29.22, 27.67, 25.88, 24.68, 22.74, 19.18, 19.01, 17.87, 13.83. HRMS Calcd for $C_{37}H_{53}BrN_2O_5$ $[M+H]^+$: 683.30651; found: 683.30443. See Figures S28 and S29.

4-(2-ethyl-4-methyl-1*H*-imidazol-1-yl)butyl-chiisanogenin (Compound **I-14**). Chloroform / methanol = 45:1 / 40:1, white solid, purity of 97.6%, yield 75%. mp 135-136 °C. ^{1}H NMR (300 MHz, $CDCl_3$): δ ppm 6.13(*brs*, 1H), 4.83 (*d, J* = 1.8 Hz, 1H), 4.73 (*d, J* = 2.2 Hz, 1H), 4.61 (*brs*, 1H), 4.53 (*q, J* = 9.0 Hz, 1H), 4.13−4.08 (*dt, J* = 6.1, 1.9 Hz, 2H), 3.56 (*d, J* = 7.8 Hz, 1H), 3.45−3.43 (*t, J* = 6.5 Hz, 2H), 2.98 (*dt, J* = 11.5, 7.3, 5.2 Hz, 1H), 2.95 (*d, J* = 14.8 Hz, 1H), 2.86 (*d, J* = 8.1 Hz, 1H), 2.70 (*d, J* = 7.9 Hz, 1H), 2.43 (*d, J* = 12.4 Hz, 1H), 2.39 (*d, J* = 4.6 Hz, 1H), 2.51−2.15 (*m*, 4H), 2.24 (*d, J* = 3.8 Hz, 1H), 1.98−1.96 (*m*, 6H), 1.95−1.93 (*dt, J* = 8.2, 6.6, 2.5 Hz, 2H), 1.91−1.80 (*m*, 3H), 1.85 (*d, J* = 1.6 Hz, 1H), 1.73 (*s*, 3H), 1.66 (*s*, 3H), 1.52 (*d, J* = 4.5 Hz, 1H), 1.60−1.09 (*m*, 11H), 1.50 (*s*, 3H), 1.01 (*s*, 3H), 0.88 (*s*, 3H). ^{13}C NMR (75 MHz, CDCl3): δ ppm 175.62, 173.07, 148.84, 146.97, 146.55, 136.37, 115.02, 113.87, 110.61, 75.35, 70.73, 62.86, 56.01, 55.03, 53.44, 49.74, 48.92, 46.79, 43.47, 43.39, 41.52, 41.14, 37.24, 36.52, 34.49, 32.18, 31.62, 30.10, 29.35, 28.81, 26.07, 24.24, 23.12, 23.02, 22.36, 18.73, 18.56, 17.51, 13.41, 12.56. HRMS Calcd for $C_{40}H_{60}N_2O_5$ $[M+H]^+$: 647.44295; found: 647.44047. See Figures S30 and S31.

4-(pyrrolidin-1-yl)butyl-chiisanogenin (Compound **I-15**). Chloroform / methanol = 40:1 / 35:1, yellow solid, purity of 97.0%, yield 65%. mp 130-131 °C. ^{1}HNMR(300 MHz, $CDCl_3$): δ ppm 4.85 (*d, J* = 1.9 Hz, 1H), 4.83 (*d, J* = 1.8 Hz, 1H), 4.74 (*d, J* = 2.2 Hz, 1H), 4.60 (*brs*, 1H), 4.53 (*q, J* = 9.0 Hz, 1H), 4.13−4.08 (*dt, J* = 6.1, 1.9Hz, 2H), 3.56 (*d, J* = 7.9 Hz, 1H), 3.46−3.42 (*t, J* = 6.5 Hz, 2H), 2.98(*dt, J* = 11.5, 7.3, 5.2 Hz, 1H), 2.95 (*d, J* = 14.8 Hz, 1H), 2.72 (*d, J* = 8.0Hz, 1H), 2.67 (*d, J* = 7.9 Hz, 1H), 2.46 (*d, J* = 12.4 Hz, 1H), 2.39 (*d, J* =4.6 Hz, 1H), 2.32−2.25 (*m*, 5H), 2.24 (*d, J* = 3.8 Hz, 1H), 1.98−1.96(*m*, 3H), 1.95−1.93 (*dt, J* = 8.2, 6.6, 2.5 Hz, 2H), 1.91−1.80 (*m*, 3H),1.78 (*d, J* = 1.6 Hz, 1H), 1.73 (*s*, 3H), 1.66 (*s*, 3H), 1.55 (*d, J* = 4.5 Hz, 1H), 1.60−1.09 (*m*, 12H), 1.04 (*s*, 3H), 1.00 (*s*, 3H), 0.86 (*s*, 3H). ^{13}C NMR (75 MHz, $CDCl_3$): δ ppm 176.04, 173.49, 149.27, 146.98, 114.31, 111.03, 75.77, 71.15, 63.28, 56.43, 55.45, 53.87, 50.16, 49.34, 47.21, 46.92, 43.89, 43.81, 41.94, 41.56, 37.66, 36.94, 34.92, 32.6, 32.04, 30.53, 29.77, 29.23, 26.5, 24.66, 23.54, 23.44, 22.78, 22.78, 19.16, 18.98, 17.94, 13.83. HRMS Calcd for $C_{38}H_{59}NO_5$ $[M+H]^+$: 608.43205; found: 608.43024. See Figures S32 and S33.

4-((2-(5-methoxy-1*H*-indol-3-yl)ethyl)amino)butyl-chiisanogenin (Compound **I-16**). Chloroform / methanol = 35:1/25:1, white solid, purity of 98.5%, yield 67%. mp 158-159 °C. ^{1}H NMR(300 MHz, $CDCl_3$): δ ppm 8.54 (*d, J* = 6.5 Hz, 1H), 7.17 (*d, J* = 3.2 Hz, 1H), 6.96-6.90(*m*, 1H), 6.75-7.69(*m*, 1H), 4.84 (*d, J* = 1.9 Hz, 1H), 4.83 (*d, J* = 1.8 Hz, 1H), 4.75 (*d, J* = 2.2 Hz, 1H), 4.52 (*brs*, 1H), 4.51 (*q, J* = 9.0 Hz, 1H), 4.23−4.03 (*dt, J* = 6.1, 1.9 Hz, 2H), 3.95(*s*, 3H), 3.57 (*d, J* = 7.8 Hz, 1H), 3.48−3.41 (*t, J* = 6.5 Hz, 2H), 3.06 (*dt, J* = 11.5, 7.3, 5.2 Hz, 1H), 2.92 (*d, J* = 14.8 Hz, 1H), 2.71 (*d, J* = 8.1 Hz, 1H), 2.68 (*d, J* = 7.9 Hz, 1H), 2.45 (*d, J* = 12.4 Hz, 1H), 2.39 (*d, J* = 4.6 Hz, 1H), 2.32−2.25 (*m*, 1H), 2.20 (*d, J* = 3.8 Hz, 1H), 1.98−1.94 (*m*, 3H), 1.95−1.93 (*dt, J* = 8.2, 6.6, 2.5 Hz, 2H), 1.91−1.80 (*m*, 7H), 1.88 (*d, J* = 1.6 Hz, 1H), 1.73 (*s*, 3H), 1.69 (*s*, 3H), 1.69 (*d, J* = 4.5 Hz, 1H), 1.71−1.09 (*m*, 10H), 1.05 (*s*, 3H), 1.01 (*s*, 3H), 0.87 (*s*, 3H). ^{13}C NMR (75 MHz, $CDCl_3$): δ ppm 176.42, 173.77, 156.06, 147.39, 132.75, 129.29, 126.46, 122.07, 120.81, 119.01, 114.22, 113.12, 111.71, 110.76, 76.06, 71.56, 63.53, 56.87, 50.58, 49.75, 47.63, 46.15, 44.31, 44.22, 42.38, 41.97, 38.11, 37.35, 35.33, 33.01, 32.53, 30.91, 29.65, 28.70, 28.10, 26.46, 25.08, 24.05, 23.79, 23.11, 21.95, 19.57, 19.39, 18.32, 14.26. HRMS Calcd for $C_{45}H_{64}N_2O_6$ $[M+H]^+$: 727.46916; found: 727.46741. See Figures S34 and S35.

4-((2-(thiophen-2-yl)ethyl)amino)butyl-chiisanogenin (Compound **I-17**). Chloroform / methanol = 50:1/40:1, yellow solid, purity of 96.0%, yield 65%. mp 140-141 °C. ^{1}HNMR(300 MHz, $CDCl_3$): δ ppm 7.13 (*d, J* = 5.1 Hz, 1H) 6.91 (*d, J* = 2.0 Hz, 1H), 6.83(*m*, 1H), 4.82 (*d, J* = 1.9 Hz, 1H), 4.81 (*d, J* = 1.8 Hz, 1H), 4.76 (*d, J* = 2.2 Hz, 1H), 4.62 (*brs*, 1H), 4.50 (*q, J* = 9.0 Hz, 1H), 4.13−4.08 (*dt, J* = 6.1, 1.9Hz, 2H), 3.55 (*d, J* = 7.8 Hz, 1H), 3.46−3.42 (*t, J* = 6.5 Hz, 2H), 2.98(*dt, J* = 11.5, 7.3, 5.2 Hz, 1H), 2.95 (*d, J* = 14.8 Hz, 1H), 2.75 (*d, J* = 8.1Hz, 1H), 2.68 (*d, J* = 7.9 Hz, 1H), 2.48 (*d, J* = 12.4 Hz, 1H), 2.39 (*d, J* =4.6 Hz, 1H), 2.32−2.25 (*m*, 6H), 2.24 (*d, J* = 3.8 Hz, 1H), 1.98−1.96(*m*, 2H), 1.95−1.93 (*dt, J* = 8.2, 6.6, 2.5 Hz, 2H), 1.91−1.80 (*m*, 3H), 1.78 (*d, J* = 1.6 Hz, 1H), 1.71 (*s*, 3H), 1.65 (*s*, 3H), 1.54 (*d, J* = 4.5 Hz,1H), 1.60−1.09 (*m*, 10H), 1.03 (*s*, 3H), 0.98 (*s*, 3H). 0.86 (*s*, 3H). ^{13}C NMR (75 MHz, $CDCl_3$): δ ppm 176.06, 173.42, 149.33, 147.01, 142.23, 127.02, 125.21, 123.78, 114.27, 110.99, 75.8, 71.19, 63.95, 56.42, 50.89, 50.18, 49.35, 49.02, 47.21, 43.9, 43.82, 42.74, 41.95, 41.57, 37.71, 36.94, 34.91, 32.61, 32.04, 30.54, 30.14, 29.23, 26.62, 26.37, 24.69, 22.73, 19.16, 19,

17.93, 13.86. HRMS Calcd for $C_{40}H_{59}NO_5S$ $[M+H]^+$: 664.40412; found: 664.40356. See Figures S36 and S37.

4-((2-(1*H*-indol-3-yl)ethyl)amino)butyl-chiisanogenin (Compound **I-18**). Chloroform / methanol = 30:1 / 25:1, yellow solid, purity of 96.4%, yield 63%. mp 155-156 °C. ^{1}HNMR(300 MHz, $CDCl_3$): δ ppm 8.16 (*d, J* = 12.2 Hz, 1H) 7.61 (*d, J* = 7.8 Hz, 1H), 7.37 (*d, J* = 8.0 Hz, 1H), 7.19 (*t, J* = 6.9 Hz, 1H), 7.14 (*t, J* = 8.0Hz, 1H), 7.06 (*d, J* = 2.3 Hz, 1H), 4.84 (*d, J* = 1.8 Hz, 1H), 4.82 (*d, J* =2.3 Hz, 1H), 4.74 (*d, J* = 2.3 Hz, 1H), 4.61 (*brs*, 1H), 4.45 (*q, J* = 8.8Hz, 1H), 4.07−3.97 (*m*, 4H), 3.53 (*d, J* = 7.8 Hz, 1H), 2.99−2.97 (*t, J* =2.5, 1.9 Hz, 2H), 2.94 (*dt, J* = 6.8 Hz, 1H), 2.88 (*d, J* = 14.8 Hz, 1H),2.72 (*d, J* = 6.9 Hz, 1H), 2.70−2.65 (*t, J* = 6.9 Hz, 2H), 2.65 (*d, J* = 6.9Hz, 1H), 2.47 (*d, J* = 12.4 Hz, 1H), 2.38 (*d, J* = 4.5 Hz, 1H), 2.35−2.26(*m*, 1H), 2.22 (*d, J* = 3.3 Hz, 1H), 1.93−1.84 (*m*, 4H), 1.79 (*d, J* = 4.4Hz, 1H), 1.72 (*s*, 3H), 1.66 (*s*, 3H), 1.52 (*d, J* = 3.2 Hz, 1H), 1.64−1.54 (*m*, 2H), 1.53 (*d, J* = 3.2 Hz, 1H), 1.52−1.06 (*m*, 10H), 1.02 (*s*, 3H), 0.96 (*s*,3H), 0.85 (*s*, 3H).^{13}C NMR (75 MHz, $CDCl_3$): δ ppm 176.11, 173.41, 149.38, 147.04, 136.55, 127.51, 122.24, 122.14, 119.36, 118.91, 114.26, 113.78, 111.34, 110.97, 75.8, 71.22, 64.05, 56.41, 50.25, 49.86, 49.36, 49.3, 47.26, 43.9, 43.83, 41.95, 41.58, 37.68, 36.95, 34.93, 32.62, 32.08, 30.55, 29.22, 26.69, 26.6, 26.44, 25.72, 24.67, 22.68, 19.14, 18.99, 17.91, 13.85. HRMS Calcd for $C_{44}H_{62}N_2O_5$ $[M+H]^+$: 697.45860; found: 697.45631. See Figures S38 and S39.

5-(4-formyl-1*H*-imidazol-1-yl)pentyl-chiisanogenin (Compound **I-19**). Chloroform / methanol = 70:1 / 60:1, white solid, purity of 97.8%, yield 65%. mp 133-134 °C. ^{1}HNMR(300 MHz, $CDCl_3$): δ ppm 9.14 (*d, J*=5.4Hz, 1H), 8.05 (*d, J*=7.9Hz, 1H), 7.73 (*d, J* =11.4Hz, 1H), 4.84 (*d, J* = 8.8 Hz, 1H), 4.81 (*d, J* = 2.1 Hz, 1H), 4.73 (*d, J* = 2.2 Hz, 1H), 4.62 (*brs*, 1H), 4.52 (*q, J* = 8.9 Hz, 1H), 4.09−4.07 (*dt, J* = 6.3, 3.9Hz, 2H), 3.58 (*d, J* = 7.8 Hz, 1H), 3.44−3.40 (*t, J* = 6.6 Hz, 2H), 2.99(*dt, J* = 12.0, 4.1 Hz, 1H), 2.93 (*d, J* = 11.6 Hz, 1H), 2.73 (*d, J* = 7.9 Hz, 1H), 2.68 (*d, J* = 7.7Hz, 1H), 2.47 (*d, J* = 11 Hz, 1H), 2.39 (*d, J* = 4.5Hz, 1H), 2.37−2.27 (*m*, 1H), 2.25 (*d, J* = 3.0 Hz, 1H), 1.96−1.85 (*m*, 6H), 1.83 (*d, J* = 4.8 Hz, 1H), 1.73 (*s*, 3H), 1.66 (*s*, 3H), 1.55 (*d, J* = 1.3 Hz, 1H), 1.54−1.07 (*m*, 12H), 1.09 (*s*, 3H), 1.01 (*s*, 3H), 0.97(*s*, 3H). ^{13}C NMR (75 MHz, $CDCl_3$): δ ppm 203.95, 175.87, 173.41, 149.3, 146.96, 144.81, 138.93, 125.78, 114.93, 110.42, 75.73, 71.16, 63.55, 56.39, 49.31, 48.81, 46.66, 43.88, 43.78, 41.92, 41.53, 40.59, 37.7, 36.71, 34.89, 32.61, 31.92, 30.59, 29.76, 29.3, 29.17, 28.29, 24.67,

23.3, 23.01, 19.55, 19.15, 17.88,13.83. HRMS Calcd for $C_{39}H_{56}N_2O_6$ $[M+H]^+$: 647.40656; found: 647.40420. See Figures S40 and S41.

5-(2-propyl-1*H*-imidazol-1-yl)pentyl-chiisanogenin (Compound **I-20**). Chloroform / methanol = 50:1/40:1, white solid, purity of 96.7%, yield 69%. mp 125-138 °C. ^{1}HNMR(300 MHz, $CDCl_3$): δ ppm 7.15(*d, J* =5.8 Hz, 1H), 6.92 (*d, J* =12.5 Hz, 1H), 4.84 (*d, J* = 1.5 Hz, 1H), 4.83 (*d, J* = 14.8 Hz, 1H), 4.75 (*d, J* = 2.3 Hz, 1H), 4.62 (*brs*, 1H), 4.51 (*q, J* = 8.9 Hz, 1H), 4.09−4.07 (*dt, J* = 6.3, 3.9Hz, 2H), 3.56 (*d, J* = 7.7 Hz, 2H), 3.44−3.40 (*t, J* = 6.6 Hz, 2H), 2.98 (*dt, J* = 12.0, 4.1 Hz, 1H), 2.95 (*d, J* = 14.8 Hz, 1H), 2.75 (*d, J* = 7.9 Hz, 1H), 2.68 (*d, J* = 7.9Hz, 1H), 2.45 (*d, J* = 9.8 Hz, 1H), 2.31 (*d, J* = 4.5Hz, 1H), 2.37−2.27 (*m*, 3H), 2.25 (*d, J* = 3.0 Hz, 1H), 1.96−1.85 (*m*, 7H), 1.83 (*d, J*= 4.8 Hz, 1H), 1.73 (*s*, 3H), 1.68 (*s*, 3H), 1.55 (*d, J* = 1.3,Hz, 1H), 1.54−1.07 (*m*, 12H), 1.04 (*s*, 3H), 1.02 (*s*, 3H),1.00 (*s*, 3H) 0.87 (*s*, 3H).^{13}C NMR (75 MHz, $CDCl_3$): δ ppm 176.01, 173.23, 149.22, 146.99, 146, 137, 119.09, 114.12, 111.53, 75.64, 71.2, 63.49, 56.41,50.21, 49.33, 47.21, 46.22, 43.91, 43.83, 41.95, 41.55, 37.67, 36.93, 34.89, 32.59, 32.12, 32.01, 30.49, 29.35, 29.2, 28.37, 28.01, 24.67, 23.25, 22.69, 21.53, 19.17, 18.97, 17.91,14.01,13.85. HRMS Calcd for $C_{41}H_{62}N_2O_5$ $[M+H]^+$: 661.45860; found: 661.45634. See Figures S42 and S43.

5-(2,4-dimethyl-1*H*-imidazol-1-yl)pentyl-chiisanogenin (Compound **I-21**). Chloroform / methanol = 55:1 / 45:1, white solid, purity of 98.7%, yield 63%. mp 110-111 °C. ^{1}H NMR (300 MHz, $CDCl_3$): δ ppm 6.63 (*brs*, 1H) 4.85 (*d, J* = 1.9 Hz, 1H), 4.83 (*d, J* = 1.8 Hz, 1H), 4.78 (*d, J* = 2.2 Hz, 1H), 4.62 (*brs*, 1H), 4.51 (*q, J* = 9.0 Hz, 1H), 4.13−4.08 (*dt, J* = 6.1, 1.9 Hz, 2H), 3.55 (*d, J* = 7.8 Hz, 1H), 3.46−3.42 (*t, J* = 6.5 Hz, 2H), 2.98 (*dt, J* = 11.5, 7.3, 5.2 Hz, 1H), 2.95 (*d, J* = 14.8 Hz, 1H), 2.75 (*d, J* = 8.1 Hz, 1H), 2.68 (*d, J* = 7.9 Hz, 1H), 2.48 (*d, J* = 12.4 Hz, 1H), 2.39 (*d, J* = 4.6 Hz, 1H), 2.32−2.25 (*m*, 6H), 2.24 (*d, J* = 3.8 Hz, 1H), 11.98−1.96 (*m*, 2H), 1.95−1.93 (*dt, J* = 8.2, 6.6, 2.5 Hz, 2H), 2.12−1.88 (*m*, 3H), 1.78 (*d, J* = 1.6 Hz, 1H), 1.72 (*s*, 3H), 1.66 (*s*, 3H), 1.55 (*d, J* = 4.5 Hz, 1H), 1.61−1.04 (*m*, 12H), 1.02 (*s*, 3H), 0.97 (*s*, 3H), 0.87 (*s*, 3H).^{13}C NMR (75 MHz, $CDCl_3$): δ ppm 175.81, 170.04, 149.58, 146.96, 144.82, 135.75, 115.38, 114.89, 110.35, 75.68, 71.03, 63.66, 56.38, 49.31, 48.86, 46.58, 45.3, 43.85, 43.76, 41.92, 41.52, 37.7, 36.61, 34.88, 32.59, 31.88, 31.52, 30.44, 29.39, 29.17, 28.43, 24.68, 23.23, 21.03, 18.93, 18.49, 17.86, 14.63, 13.81, 13.23. HRMS Calcd for $C_{40}H_{60}N_2O_5$ $[M+H]^+$: 647.44295; found: 647.44024. See Figures S44 and S45.

5-(4-bromo-1*H*-imidazol-1-yl)pentyl-chiisanogenin (Compound **I-22**). Chloroform / methanol = 45:1 / 40:1, white solid, purity of 95.3%, yield 53%. mp 126-127 °C. ^{1}H NMR (300 MHz, $CDCl_3$): δ ppm 7.95 (*d, J* = 6.9 Hz, 1H), 7.12 (*d, J* = 7.8 Hz, 1H) ,4.85 (*d, J* = 1.5 Hz, 1H), 4.83 (*d, J* = 2.3 Hz, 1H), 4.73 (*d, J* = 2.3 Hz, 1H), 4.56 (*brs*, 1H), 4.51 (*q, J* = 8.8 Hz, 1H), 4.09−4.00 (*dt, J* = 6.3, 3.9 Hz, 2H), 3.61 (*d, J* = 7.9 Hz, 1H), 3.05−2.88 (*t, J* = 6.6 Hz, 2H), 2.73 (*dt, J* = 12.0, 4.1 Hz, 1H), 2.95 (*d, J* = 14.8 Hz, 1H), 2.75 (*d, J* = 7.9 Hz, 1H), 2.67 (*d, J* = 8.0 Hz, 1H), 2.46 (*d, J* = 11.2 Hz, 1H), 2.31 (*d, J* = 4.5 Hz, 1H), 2.37−2.27 (*m*, 1H), 2.25 (*d, J* = 3.0 Hz, 1H), 2.08−1.88 (*m*, 8H), 1.83 (*d, J* = 4.8 Hz, 1H), 1.73 (*s*, 3H), 1.65 (*s*, 3H), 1.56 (*d, J* = 1.3, Hz, 1H), 1.54−1.07 (*m*, 12H), 1.25 (*s*, 3H), 0.99 (*s*, 3H), 0.89 (*s*, 3H).^{13}C NMR (75 MHz, $CDCl_3$): δ ppm 176.73, 173.33, 149.72, 146.9, 135.3, 131.28, 116.94, 111.56, 104.35, 75.69, 71.2, 63.54, 56.58, 50.19, 49.28, 48.23, 46.89, 43.91, 43.77, 41.96, 41.62, 37.71, 37.08, 35.04, 32.65, 32.1, 32,30.51, 29.82, 29.19, 28.27, 24.64, 23.95, 23.1, 19.2, 19.01, 17.9, 13.8. HRMS Calcd for $C_{38}H_{57}BrN_2O_5$ $[M+H]^+$: 697.32216; found: 697.32147. See Figures S46 and S47.

5-(2-ethyl-4-methyl-1*H*-imidazol-1-yl)pentyl-chiisanogenin (Compound **I-23**). Chloroform / methanol = 50:1 / 40:1, white solid, purity of 97.5%, yield 67%. mp 109-110 °C. ^{1}H NMR (300 MHz, $CDCl_3$): δ ppm 6.48 (*brs*, 1H) 4.85 (*d, J* = 1.5 Hz, 1H), 4.83 (*d, J* = 2.3 Hz, 1H), 4.74 (*d, J* = 2.3 Hz, 1H), 4.61 (*brs*, 1H), 4.50 (*q, J* = 8.9 Hz, 1H), 4.09−4.04 (*dt, J* = 6.3, 3.9 Hz, 2H), 3.56 (*d, J* = 7.9 Hz, 1H), 3.44−3.40 (*t, J* = 6.6 Hz, 2H), 2.98 (*dt, J* = 12.0, 4.1 Hz, 1H), 2.91 (*d, J* = 14.8 Hz, 1H), 2.72 (*d, J* = 7.9 Hz, 1H), 2.63 (*d, J* = 8.0 Hz, 1H), 2.44 (*d, J* = 11.2 Hz, 1H), 2.39 (*d, J* = 4.5 Hz, 1H), 2.37−2.27 (*m*, 7H), 2.16 (*d, J* = 3.0 Hz, 1H), 1.96−1.85 (*m*, 5H), 1.86 (*d, J* = 4.8 Hz, 1H), 1.72 (*s*, 3H), 1.66 (*s*, 3H), 1.55 (*d, J* = 1.3, Hz, 1H), 1.55−1.11 (*m*, 15H), 1.04 (*s*, 3H), 0.99 (*s*, 3H), 0.87 (*s*, 3H). ^{13}C NMR (75 MHz, $CDCl_3$): δ ppm 175.99, 173.44, 149.24, 148.26, 146.94, 135.78, 115.17, 114.22, 110.96, 75.69, 71.06, 63.61, 56.37, 49.3, 47.18, 45.4, 43.83, 43.73, 43.18, 41.91, 41.51, 37.65, 36.87, 34.87, 32.56, 32.08, 30.64, 30.46, 29.79, 29.17, 28.4, 24.67, 23.45, 23.29, 19.11, 18.94, 17.86, 13.81, 13.47, 12.61, 12.08. HRMS Calcd for $C_{41}H_{62}N_2O_5$ $[M+H]^+$: 661.45860; found: 661.45601. See Figures S48 and S49.

5-(pyrrolidin-1-yl)pentyl-chiisanogenin (Compound **I-24**). Chloroform / methanol = 45:1 / 40:1, white solid, purity of 96.4%, yield 67%. mp 104-105 °C. ^{1}H NMR (300

MHz, $CDCl_3$): δ ppm 4.85 (*d, J* = 1.5 Hz, 1H), 4.83 (*d, J* = 2.3 Hz, 1H), 4.75 (*d, J* = 2.3 Hz, 1H), 4.62 (*brs*, 1H), 4.51 (*q, J* = 8.9 Hz, 1H), 4.09−4.06 (*dt, J* = 6.3, 3.9 Hz, 2H), 3.57 (*d, J* = 7.9 Hz, 1H), 3.44−3.40 (*t, J* = 6.6 Hz, 2H), 2.96 (*dt, J* = 12.0, 4.1 Hz, 1H), 2.93 (*d, J* = 14.8 Hz, 1H), 2.74 (*d, J* = 7.9 Hz, 1H), 2.69 (*d, J* = 8.0 Hz, 1H), 2.41 (*d, J* = 11.2 Hz, 1H), 2.34 (*d, J* = 4.5 Hz, 1H), 2.37−2.27 (*m*, 7H), 2.01 (*d, J* = 3.0 Hz, 1H), 2.09−1.83 (*m*, 7H), 1.83 (*d, J* = 4.8 Hz, 1H), 1.73 (*s*, 3H), 1.67 (*s*, 3H), 1.55 (*d, J* = 1.3, Hz, 1H), 1.66−1.07 (*m*, 12H), 1.05 (*s*, 3H), 1.02 (*s*, 3H), 0.89 (*s*, 3H). ^{13}C NMR (75 MHz, $CDCl_3$): δ ppm 176.30, 173.36, 149.52, 147.33, 114.54, 111.31, 71.60, 63.76, 56.70, 55.81, 53.83, 50.68, 49.61, 47.51, 47.07, 44.17, 43.18, 42.22, 41.86, 37.90, 37.21, 35.20, 32.86, 32.29, 30.80, 30.05, 29.48, 28.57, 25.76, 24.91, 24.15, 23.74, 23.27, 22.73, 21.34, 19.44, 19.22, 18.24, 14.12. HRMS Calcd for $C_{39}H_{61}NO_5$ $[M+H]^+$: 622.44770; found: 622.44547. See Figures S50 and S51.

5-((2-(5-methoxy-1H-indol-3-yl)ethyl)amino)pentyl-chiisanogenin (Compound **I-25**) Chloroform / methanol = 35:1 / 25:1, white solid, purity of 97.5%, yield 65%. mp 114-124 °C. ^1H NMR (300 MHz, $CDCl_3$): δ ppm 9.14 (*brs*, 1H), 7.22 (*d, J* = 7.4 Hz, 1H), 7.05 (*dd, J* = 6.2, 2.4 Hz, 1H), 6.85 (*dt, J* = 8.7, 2.2 Hz, 1H), 4.84 (*d, J* = 1.5 Hz, 1H), 4.82 (*d, J* = 2.3 Hz, 1H), 4.75 (*d, J* = 2.3 Hz, 1H), 4.63 (*brs*, 1H), 4.50 (*q, J* = 8.9 Hz, 1H), 4.08−3.98 (*dt, J* = 6.3, 3.9 Hz, 2H), 3.85(*s*, 3H),3.54 (*d, J* = 7.9 Hz, 1H), 3.44−3.40 (*t, J* = 6.6 Hz, 2H), 3.02 (*dt, J* = 12.0, 4.1 Hz, 1H), 2.87 (*d, J* = 14.8 Hz, 1H), 2.75 (*d, J* = 7.9 Hz, 1H), 2.69 (*d, J* = 8.0 Hz, 1H), 2.49 (*d, J* = 11.2 Hz, 1H), 2.39 (*d, J* = 4.5 Hz, 1H), 2.28−2.07 (*m*, 6H), 2.25 (*d, J* = 3.0 Hz, 1H), 2.01−1.83 (*m*, 7H), 1.82 (*d, J* = 4.8 Hz, 1H), 1.73 (*s*, 3H), 1.68 (*s*, 3H), 1.55 (*d, J* = 1.3, Hz, 1H), 1.64−1.05 (*m*, 12H), 1.02 (*s*, 3H), 0.97 (*s*, 3H), 0.85 (*s*, 3H). ^{13}C NMR (75 MHz, $CDCl_3$): δ ppm 176.29, 173.23, 154.12, 147.86, 132.75, 128.73, 125.13, 122.25, 119.65, 118.89, 114.29, 112.36, 112.13, 111.08, 75.57, 71.25, 65.68, 57.33, 56.14, 50.30, 49.35, 47.29, 47.07, 46.67, 43.92, 43.84, 41.97, 41.60, 36.98, 36.73, 35.01, 32.64, 32.09, 31.62, 30.56, 29.82, 29.23, 28.65, 24.75, 23.86, 23.42, 22.19, 18.99, 18.55, 17.92, 13.85. HRMS Calcd for $C_{46}H_{66}N_2O_6$ $[M+H]^+$: 741.48481; found: 741.48367. See Figures S52 and S53.

5-((2-(thiophen-2-yl)ethyl)amino)penty-chiisanogenin (Compound **I-26**). Chloroform / methanol = 60:1 / 50:1, yellow solid, purity of 98.1%, yield 65%. mp 125-126 °C. ^1HNMR(300 MHz, $CDCl_3$): δ ppm7.14 (*d, J* = 5.1 Hz, 1H), 6.93 (*d, J* = 5.3 Hz, 1H), 6.83 (*d, J* = 3.0 Hz, 1H),4.84 (*d, J* = 6.5 Hz, 1H), 4.82 (*d, J* = 2.1 Hz, 1H), 4.77 (*d, J* =

9.8 Hz, 1H), 4.62 (*brs*, 1H), 4.51 (*q, J* = 8.8 Hz, 1H), 4.09−4.07 (*dt, J* = 6.3, 3.9Hz, 2H), 3.56 (*d, J* = 7.8 Hz, 1H), 3.44−3.40 (*t, J* = 6.6 Hz, 2H), 2.98(*dt, J* = 12.0, 4.1 Hz, 1H), 2.95 (*d, J* = 14.8 Hz, 1H), 2.75 (*d, J* = 7.9 Hz,1H), 2.67 (*d, J* = 8.0 Hz, 1H), 2.46 (*d, J* = 11.4 Hz, 1H), 2.39 (*d, J* = 4.5Hz, 1H), 2.37−2.27 (*m*, 5H), 2.25 (*d, J* = 3.0 Hz, 1H), 1.96−1.85 (*m*, 7H), 1.83 (*d, J* = 4.8 Hz, 1H), 1.73 (*s*, 3H), 1.68 (*s*, 3H), 1.55 (*d, J* = 1.3,Hz, 1H), 1.54−1.07 (*m*, 12H), 1.05 (*s*, 3H), 1.00 (*s*, 3H), 0.88 (*s*, 3H).^{13}C NMR (75 MHz, $CDCl_3$): δ ppm 176.09, 173.38, 149.35, 147.02, 142.35, 127, 125.18, 123.74, 114.31, 111.05, 75.8, 71.21, 64.02, 56.41, 50.23, 49.47, 49.36, 47.23, 47.14, 43.91, 43.83, 41.96, 41.58 ,37.72, 36.96, 34.93, 32.63, 32.12, 30.55, 30.24, 29.48, 29.23, 28.71, 28.50, 24.7, 23.9, 22.71, 19.17, 19.01, 17.94, 13.86. HRMS Calcd for $C_{41}H_{61}NO_5S$ $[M+H]^+$: 678.41977; found: 678.41720. See Figures S54 and S55.

5-((2-(1H-indol-3-yl)ethyl)amino)pentyl-chiisanogenin (Compound **I-27**). Chloroform / methanol = 25:1/20:1, yellow solid, purity of 96.3%, yield 48%. mp 112-113 °C. ^{1}HNMR(300 MHz, CDCl3): δ ppm8.16 (*d, J* = 12.2 Hz, 1H) ,7.62 (*d, J* = 7.8 Hz, 1H), 7.37 (*d, J* = 8.1 Hz, 1H), 7.18 (*t, J* = 6.9 Hz, 1H), 7.10 (*t, J* = 6.9Hz, 1H), 7.05 (*d, J* = 2.2 Hz, 1H), 4.85 (*d, J* = 1.9 Hz, 1H), 4.83 (*d, J* =1.9 Hz, 1H), 4.74 (*d, J* = 2.3 Hz, 1H), 4.61 (*brs*, 1H), 4.48 (*q, J* = 8.9Hz, 1H), 4.05−4.00 (*tt, J* = 6.6, 3.5 Hz, 4H), 3.56 (*d, J* = 7.8 Hz, 1H),3.06−3.01 (*dd, J* = 9.7, 4.9 Hz, 2H), 2.99 (*dt, J* = 13.1, 3.1 Hz, 1H), 2.98(*d, J* = 14.8 Hz, 1H), 2.71 (*d, J* = 7.3 Hz 1H), 2.70−2.66 (*m*, 2H), 2.63(*d, J* = 10.2 Hz, 1H), 2.47 (*d, J* = 10.6 Hz, 1H), 2.36 (*d, J* = 4.3 Hz, 1H),2.35−2.18 (*m*, 1H), 2.21 (*d, J* = 3.0 Hz, 1H), 1.93−1.78 (*m*, 7H), 1.76(*d, J* = 3.2 Hz, 1H), 1.72 (*s*, 3H), 1.66 (*s*, 3H), 1.52 (*d, J* = 5.7 Hz, 1H),1.62−1.07 (*m*, 12H), 1.03 (*s*, 3H), 0.97 (*s*, 3H), 0.86 (*s*, 3H).^{13}C NMR (75 MHz, $CDCl_3$): δ ppm 176.10, 173.75, 149.42, 146.98, 136.52, 127.20, 122.78, 122.23, 119.52, 118.75, 114.32, 114.15, 111.51, 111.01, 75.88, 71.16, 63.90, 56.40, 50.16, 49.33, 49.09, 48.81, 47.24, 43.88, 43.77, 41.93, 41.56, 37.66, 36.96, 34.92, 32.62, 32.05, 30.56, 29.20, 29.02, 28.47, 27.70, 24.66, 24.13, 23.71, 22.73, 19.13, 19.03, 17.89, 13.82. HRMS Calcd for $C_{45}H_{64}N_2O_5$ $[M+H]^+$: 711.47425; found: 711.47342. See Figures S56 and S57.

6-(4-formyl-1H-imidazol-1-yl)hexyl-chiisanogenin (Compound **I-28**). Chloroform / methanol = 60:1 / 50:1, white solid, purity of 98.2%, yield 65%. mp 126-127 °C. ^{1}HNMR(300 MHz, $CDCl_3$): δ ppm 9.88 (*d, J* = 21.1 Hz, 1H), 8.16 (*d, J* = 11 Hz, 1H), 7.70 (*d, J* = 6.8 Hz, 1H), 4.84 (*d, J* = 6.2 Hz, 1H), 4.83 (*d, J* = 2.1 Hz, 1H), 4.73 (*d, J* =

2.6 Hz, 1H), 4.62 (*brs*, 1H), 4.59 (*q, J* = 9.0 Hz, 1H), 4.07−4.05 (*dt, J* = 10.9,8.5 Hz, 2H), 3.54 (*d, J* = 5.4 Hz, 1H), 3.43−3.38 (*t, J* = 6.9 Hz, 1H),2.96 (*dt, J* = 10.9 Hz, 1H), 2.95 (*d, J* = 14.8 Hz, 1H), 2.76 (*d, J* = 7.9 Hz, 1H), 2.68 (*d, J* = 7.9 Hz, 1H), 2.47 (*d, J* = 11.1 Hz, 1H), 2.41 (*d, J* = 2.7Hz, 1H), 2.37−2.28 (*m*, 1H), 2.25 (*d, J* = 3.9 Hz, 1H), 1.96−1.85 (*m*, 7H), 1.82 (*d, J* = 2.0 Hz, 1H), 1.73 (*s*, 3H), 1.66 (*s*, 3H), 1.54 (*d, J* = 11.2Hz, 1H), 1.51−1.08 (*m*, 14H), 1.03 (*s*, 3H), 1.00 (*s*, 3H), 0.87 (*s*, 3H). ^{13}C NMR (75 MHz, $CDCl_3$): δ ppm 185.69, 176.02, 173.49, 149.31, 146.92, 141.52, 138.94, 124.43, 114.31, 110.94, 75.75, 71.11, 63.76, 56.38, 50.04, 49.31, 48.01, 47.18, 43.85, 43.75, 41.92, 41.53, 37.71, 36.93, 34.8, 32.61, 30.8, 30.66, 29.77, 29.18, 28.57, 26.19, 26.07, 25.63, 24.68, 22.77, 19.15, 19, 17.87,13.83. HRMS Calcd for $C_{40}H_{58}N_2O_6$ $[M+H]^+$: 661.42221; found: 661.42354. See Figures S58 and S59.

6-(2-propyl-1*H*-imidazol-1-yl)hexyl-chiisanogenin (Compound **I-29**). Chloroform / methanol = 50:1 / 40:1, white solid, purity of 95.8%, yield 58%. mp 116-117 °C. ^{1}HNMR(300 MHz, $CDCl_3$): δ ppm 7.09 (*d, J* = 1.7 Hz, 1H) 6.89 (*d, J* = 1.7 Hz, 1H), 4.84 (*d, J* = 1.6 Hz, 1H), 4.83 (*d, J* = 5.9 Hz, 1H), 4.74 (*d, J* = 2.3 Hz, 1H), 4.60 (*brs*, 1H), 4.51 (*q, J* = 8.9 Hz, 1H), 4.08−4.05 (*ddt, J* = 8.0,5.9, 3.6 Hz, 2H), 3.56 (*d, J* = 7.8 Hz, 1H), 3.43−3.38 (*t, J* = 6.8 Hz, 2H),2.95 (*dt, J* = 10.8 Hz, 1H), 2.91 (*d, J* = 14.8 Hz, 1H), 2.75 (*d, J* = 8.0 Hz, 1H), 2.67 (*d, J* = 7.9 Hz, 1H), 2.50 (*d, J* = 14.3 Hz, 1H), 2.39 (*d, J* = 4.5Hz, 1H), 2.34−2.18 (*m*, 5H), 2.24 (*d, J* = 3.3 Hz, 1H), 1.97−1.80 (*m*, 6H), 1.78 (*d, J* = 3.6 Hz, 1H), 1.72 (*s*, 3H), 1.66 (*s*, 3H), 1.59 (*d, J* = 3.4Hz, 1H), 1.63−1.08 (*m*, 14H), 1.04 (*s*, 3H), 1.01 (*s*, 3H), 0.99 (*s*, 3H),0.87 (*s*, 3H). ^{13}C NMR (75 MHz, $CDCl_3$): δ ppm 176.04, 173.35, 149.29, 147.95, 146.99, 126.07, 118.94, 114.26, 110.99, 75.68, 71.12, 63.82, 56.38, 50.14, 49.31, 47.2, 45.93, 43.87, 43.79, 41.93, 41.53, 37.68, 36.93, 34.89, 32.59, 32.09, 30.9, 30.49, 29.77, 29.18, 28.6, 28.48, 26.36, 25.78, 24.68, 22.71, 21.55, 19.15, 18.96, 17.88, 14.05, 13.83. HRMS Calcd for $C_{42}H_{64}N_2O_5$ $[M+H]^+$: 675.47425; found: 675.47204. See Figures S60 and S61.

6-(2,4-dimethyl-1*H*-imidazol-1-yl)hexyl-chiisanogenin (Compound **I-30**). Chloroform / methanol = 45:1 / 40:1, white solid, purity of 98.1%, yield 71%. mp 143-144 °C. ^{1}HNMR(300 MHz, $CDCl_3$): δ ppm 6.49 (*brs*, 1H),4.85 (*d, J* = 1.8 Hz, 1H), 4.83 (*d, J* = 5.1 Hz, 1H), 4.74 (*d, J* = 2.3 Hz, 1H), 4.62 (*brs*, 1H), 4.51 (*q, J* = 8.7 Hz, 1H), 4.07−4.05 (*dt, J* = 10.9,8.5 Hz, 2H), 3.56 (*d, J* = 7.8 Hz, 1H), 3.43−3.38 (*t, J* = 6.9 Hz, 1H),2.99 (*dt, J* = 10.9 Hz, 1H), 2.97 (*d, J* = 10.5 Hz, 1H), 2.71 (*d, J* = 7.9 Hz, 1H), 2.66(*d, J* =

7.9 Hz, 1H), 2.47 (*d, J* = 11.1 Hz, 1H), 2.41 (*d, J* = 2.7Hz, 1H), 2.37−2.28 (*m*, 7H), 2.25 (*d, J* = 3.9 Hz, 1H), 1.96−1.85 (*m*, 7H), 1.92 (*d, J* = 3.1 Hz, 1H), 1.72 (*s*, 3H), 1.66 (*s*, 3H), 1.58 (*d, J* = 6.7Hz, 1H), 1.51−1.08 (*m*, 14H), 1.04 (*s*, 3H), 0.99 (*s*, 3H), 0.87 (*s*, 3H). ^{13}C NMR (75 MHz, $CDCl_3$): δ ppm 176.02, 173.44, 149.28, 146.94, 143.66, 137.69, 115.36, 114.17, 110.92, 75.69, 70.99, 63.87, 56.36, 49.97, 49.3, 47.18, 45.82, 43.81, 43.72, 41.9, 41.5, 37.67, 36.89, 34.87, 32.57, 31.99, 30.7, 30.47, 29.16, 28.58, 26.29, 25.76, 24.67, 23.1, 22.74, 19.08, 18.93, 17.83, 13.79, 13.28, 12.78. HRMS Calcd for $C_{41}H_{62}N_2O_5$ $[M+H]^+$: 661.45860; found: 661.45602. See Figures S62 and S63.

6-(4-bromo-1*H*-imidazol-1-yl)hexyl-chiisanogenin (Compound **I-31**). Chloroform / methanol = 45:1 / 35:1, white solid, purity of 96.5%, yield 52%. mp 113-126 °C. ^{1}H NMR (300 MHz, $CDCl_3$): δ ppm 8.16 (*d, J* = 3.6 Hz, 1H), 6.94 (*d, J* = 4.9 Hz, 1H), 4.85 (*d, J* = 1.8 Hz, 1H), 4.83 (*d, J* = 1.8 Hz, 1H), 4.75 (*d, J* = 2.1 Hz, 1H), 4.62 (*brs*, 1H), 4.52 (*q, J* = 9.0 Hz, 1H), 4.07−4.04 (*dt, J* = 10.9, 8.5 Hz, 2H), 3.58 (*d, J* = 5.4 Hz, 1H), 3.43−3.38 (*t, J* = 6.9 Hz, 1H), 2.97 (*dt, J* = 10.9 Hz, 1H), 2.91 (*d, J* = 14.8 Hz, 1H), 2.73 (*d, J* = 7.9 Hz, 1H), 2.68 (*d, J* = 7.9 Hz, 1H), 2.46 (*d, J* = 11.1 Hz, 1H), 2.39 (*d, J* = 2.7 Hz, 1H), 2.37−2.28 (*m*, 1H), 2.27 (*d, J* = 3.9 Hz, 1H), 1.96−1.81 (*m*, 7H), 1.81 (*d, J* = 2.0 Hz, 1H), 1.73 (*s*, 3H), 1.66 (*s*, 3H), 1.55 (*d, J* = 4.5 Hz, 1H), 1.65−1.08 (*m*, 14H), 1.04 (*s*, 3H), 1.00 (*s*, 3H), 0.87 (*s*, 3H). ^{13}C NMR (75 MHz, $CDCl_3$): δ ppm176.04, 173.44, 149.32, 146.94, 136.93, 133.2, 118.44, 114.33, 110.96, 75.74, 71.13, 63.8, 56.39, 49.32, 48.07, 47.19, 45.52, 43.88, 43.77, 41.93, 41.54, 37.69, 36.94, 34.89, 32.1, 32.02, 30.73, 30.52, 29.19, 28.57, 26.16, 25.63, 24.69, 23.04, 22.78, 19.16, 19, 17.89, 13.84. HRMS Calcd for $C_{39}H_{57}BrN_2O_5$ $[M+H]^+$: 711.33781; found: 711.33501. See Figures S64 and S65.

6-(2-ethyl-4-methyl-1*H*-imidazol-1-yl)hexyl-chiisanogenin (Compound **I-32**). Chloroform / methanol = 55:1 / 45:1, white solid, purity of 96.4%, yield 64%. mp 136-148 °C. ^{1}H NMR (300 MHz, $CDCl_3$): δ ppm 6.63 (*d, J* = 9.5 Hz, 1H), 4.85 (*d, J* = 1.8 Hz, 1H), 4.83 (*d, J* = 1.8 Hz, 1H), 4.74 (*d, J* = 2.0 Hz, 1H), 4.61 (*brs*, 1H), 4.51 (*q, J* = 9.0 Hz, 1H), 4.07−4.04 (*dt, J* = 10.9, 8.5 Hz, 2H), 3.74 (*d, J* = 5.4 Hz, 1H), 3.43−3.38 (*t, J* = 6.9 Hz, 1H), 2.99 (*dt, J* = 10.9 Hz, 1H), 2.91 (*d, J* = 14.8 Hz, 1H), 2.73 (*d, J* = 7.9 Hz, 1H), 2.66 (*d, J* = 7.9 Hz, 1H), 2.46 (*d, J* = 11.1 Hz, 1H), 2.32 (*d, J* = 2.7 Hz, 1H), 2.37−2.28 (*m*, 6H), 2.21 (*d, J* = 3.9 Hz, 1H), 1.96−1.84 (*m*, 7H), 1.82 (*d, J* = 2.0 Hz, 1H), 1.72 (*s*, 3H), 1.69 (*s*, 3H), 1.55 (*d, J* = 4.5 Hz, 1H), 1.63−1.09 (*m*, 17H), 1.04

(*s*, 3H), 1.00 (*s*, 3H), 0.87 (*s*, 3H). ^{13}C NMR (75 MHz, CDCl3): δ ppm 176.06, 173.47, 149.3, 148.08, 146.96, 136.06, 115.21, 114.24, 110.95, 75.73, 71.07, 63.87, 56.37, 50.07, 49.3, 47.2, 45.44, 43.85, 43.75, 41.91, 41.52, 37.66, 36.91, 34.89, 32.57, 32.08, 30.98, 30.48, 29.76, 29.17, 28.61, 26.4, 25.81, 24.67, 22.91, 20.13, 19.11, 18.94, 17.86, 13.81, 13.53, 12.64. HRMS Calcd for $C_{42}H_{64}N_2O_5$ $[M+H]^+$: 675.47425; found: 675.47235. See Figures S66 and S67.

6-(pyrrolidin-1-yl)hexyl-chiisanogenin (Compound **I-33**). Chloroform / methanol = 50:1 / 40:1, white solid, purity of 97.4%, yield 64%. mp 140-141 °C. ^{1}HNMR(300 MHz, $CDCl_3$): δ ppm4.85 (*d, J* = 1.8 Hz, 1H), 4.83 (*d, J* = 1.8 Hz, 1H), 4.75 (*d, J* = 2.0 Hz,1H), 4.62 (*brs*, 1H), 4.53 (*q, J* = 9.9 Hz, 1H), 4.07−4.05 (*dt, J* = 10.9,8.5 Hz, 2H), 3.54 (*d, J* = 5.4 Hz, 1H), 3.43−3.38 (*t, J* = 6.9 Hz, 1H),2.98 (*dt, J* = 10.8 Hz, 1H), 2.95 (*d, J* = 14.8 Hz, 1H), 2.72 (*d, J* = 7.9 Hz, 1H), 2.67 (*d, J* = 7.4 Hz, 1H), 2.47 (*d, J* = 11.1 Hz, 1H), 2.41 (*d, J* = 2.7Hz, 1H), 2.37−2.28 (*m*, 5H), 2.25 (*d, J* = 3.9 Hz, 1H), 1.96−1.85 (*m*, 7H), 1.82 (*d, J* = 2.0 Hz, 1H), 1.72 (*s*, 3H), 1.68 (*s*, 3H), 1.55 (*d, J* = 4.5Hz, 1H), 1.51−1.08 (*m*, 18H), 1.04 (*s*, 3H), 1.01 (*s*, 3H), 0.88 (*s*, 3H).^{13}C NMR (75 MHz, $CDCl_3$): δ ppm 176.1, 173.68, 149.42, 147.04, 114.3, 110.95, 75.83, 71.19, 63.87, 56.42, 55.67, 53.8, 50.24, 49.34, 47.53, 47.27, 43.91, 43.82, 41.96, 41.58, 37.69, 36.97, 34.95, 32.62, 32.1, 31.63, 30.55, 29.21, 28.54, 26.62, 25.79, 25.68, 24.69, 23.45, 22.72, 19.19, 18.98, 18.51, 17.93, 13.86. HRMS Calcd for $C_{40}H_{63}NO_5$ $[M+H]^+$: 636.46335; found: 636.46420. See Figures S68 and S69.

6-((2-(5-methoxy-1*H*-indol-3-yl)ethyl)amino)hexyl-chiisanogenin (Compound **I-34**). Chloroform / methanol = 35:1 / 25:1, white solid, purity of 98.2%, yield 71%. mp 135-136 °C. ^{1}H NMR (300 MHz, $CDCl_3$): δ ppm 9.15 (*brs*, 1H), 7.24 – 7.17 (*m*, 1H), 6.83 (*dq, J* = 8.7, 2.9 Hz, 1H) 4.85 (*d, J* = 1.8 Hz, 1H), 4.82 (*d, J* = 1.8 Hz, 1H), 4.75 (*d, J* = 2.0 Hz, 1H), 4.62 (*brs*, 1H), 4.57 (*q, J* = 9.0 Hz, 1H), 3.88−3.79 (*dt, J* = 10.9, 8.5 Hz, 2H), 3.54 (*d, J* = 5.4 Hz, 1H), 3.43−3.38 (*t, J* = 6.9 Hz, 1H), 2.99 (*dt, J* = 10.9 Hz, 1H), 2.88 (*d, J* = 14.8 Hz, 1H), 2.77 (*d, J* = 7.9 Hz, 1H), 2.68 (*d, J* = 7.9 Hz, 1H), 2.47 (*d, J* = 11.1 Hz, 1H), 2.41 (*d, J* = 2.7 Hz, 1H), 2.37−2.28 (*m*, 8H), 2.26 (*d, J* = 3.9 Hz, 1H), 1.98−1.87 (*m*, 10H), 1.86 (*d, J* = 2.0 Hz, 1H), 1.78 (*s*, 3H), 1.63 (*s*, 3H), 1.60 (*d, J* = 4.5 Hz, 1H), 1.62−1.01 (*m*, 14H), 0.99 (*s*, 3H), 0.91 (*s*, 3H), 0.86 (*s*, 3H). ^{13}C NMR (75 MHz, $CDCl_3$): δ ppm 176.32, 173.84, 153.97, 153.24, 150.18, 127.98, 123.53, 123.38, 123.28, 118.10, 117.63, 114.34, 112.69, 111.00, 75.78, 69.21, 64.61, 56.63, 56.08,

50.13, 49.39, 48.93, 46.56, 42.77, 42.39, 40.12, 39.64, 37.56, 36.92, 33.35, 32.40, 32.02, 31.07, 30.81, 29.68, 29.28, 29.01, 28.73, 26.94, 25.79, 25.23, 24.93, 21.73, 19.43, 19.02, 17.25, 13.87. HRMS Calcd for $C_{47}H_{68}N_2O_6$ $[M+H]^+$: 755.50046; found: 755.50247. See Figures S70 and S71.

6-((2-(thiophen-2-yl)ethyl)amino)hexyl-chiisanogenin (Compound **I-35**). Chloroform / methanol = 50:1 / 40:1, white solid, purity of 97.2%, yield 55%. mp 110-111 °C. ^{1}HNMR(300 MHz, $CDCl_3$): δ ppm 7.14 (*d, J* = 5.7 Hz, 1H), 6.94 (*d, J* = 12.6Hz, 1H), 4.84 (*d, J* = 6.7 Hz, 1H), 4.83 (*d, J* = 2.1 Hz, 1H), 4.75 (*d, J* = 2.0 Hz, 1H), 4.62 (*brs*, 1H), 4.50 (*q, J* = 9.0 Hz, 1H), 4.07−4.05 (*dt, J* = 10.9,8.5 Hz, 2H), 3.56 (*d, J* = 7.8 Hz, 1H), 3.43−3.38 (*t, J* = 6.9 Hz, 1H),2.99 (*dt, J* = 10.9 Hz, 1H), 2.95 (*d, J* = 14.8 Hz, 1H), 2.73 (*d, J* = 7.9 Hz,1H), 2.67 (*d, J* = 6.8 Hz, 1H), 2.46 (*d, J* = 11.9 Hz, 1H), 2.41 (*d, J* = 2.7Hz, 1H), 2.37−2.28 (*m*, 7H), 2.31 (*d, J* = 3.9 Hz, 1H), 1.96−1.85 (*m*, 7H), 1.82 (*d, J* = 2.0 Hz, 1H), 1.73 (*s*, 3H), 1.68 (*s*, 3H), 1.57 (*d, J* = 3.7Hz, 1H), 1.51−1.08 (*m*, 14H), 1.05 (s, 3H), 1.01 (*s*, 3H), 0.89 (*s*, 3H). ^{13}C NMR (75 MHz, CDCl3): δ ppm 176.53, 173.36, 149.92, 147.05, 142.22, 127.03, 125.22, 123.75, 114.3, 110.9, 75.79, 71.25, 64.12, 56.42, 51.06, 50.27, 49.36, 47.26, 47.06, 43.93, 43.85, 41.97, 41.59, 37.74, 36.97, 34.94, 32.62, 32.12, 30.56, 30.18, 29.72, 29.23, 29.04, 28.73, 27.01, 26.05, 24.7, 22.69, 19.18, 19.01, 17.95, 13.87. HRMS Calcd for $C_{42}H_{63}NO_5S$ $[M+H]^+$: 692.43542; found: 692.43347. See Figures S72 and S73.

6-((2-(1*H*-indol-3-yl)ethyl)amino)hexyl-chiisanogenin (Compound **I-36**). Chloroform / methanol = 25:1 / 20:1, white solid, purity of 96.5%, yield 69%. mp 135-136 °C. ^{1}HNMR(300 MHz, $CDCl_3$): δ ppm 8.21 (*d, J* = 12.2 Hz, 1H), 7.62 (*d, J* = 7.8 Hz, 1H), 7.37 (*d, J* = 8.0 Hz, 1H), 7.19 (*t, J* = 6.9 Hz, 1H), 7.11 (*t, J* = 7.1Hz, 1H), 7.05 (*d, J* = 2.3 Hz, 1H), 4.85 (*d, J* = 1.8 Hz, 1H), 4.83 (*d, J* =2.3 Hz, 1H), 4.75 (*d, J* = 2.4 Hz, 1H), 4.61 (*brs*, 1H), 4.47 (*q, J* = 8.9Hz, 1H), 4.09−3.99(*dt, J* = 6.6, 3.2 Hz, 2H), 3.55 (*d, J* = 7.8 Hz, 1H),3.01−2.98 (*dt, J*= 10.6, 5.4 Hz, 2H), 2.93 (*d, J*= 14.5 Hz, 1H), 2.71 (*d, J*= 8.0 Hz, 1H), 2.67−2.62 (*t, J* = 7.2 Hz, 1H), 2.65 (*d, J* = 7.3 Hz, 1H),2.47 (*d, J* = 10.0 Hz, 1H), 2.39 (*d, J* = 4.2 Hz, 1H), 2.35−2.26 (*m*, 5H),2.23 (*d, J* = 3.1 Hz, 1H), 1.94−1.83 (*m*, 7H), 1.80 (*d, J* = 4.9 Hz, 1H),1.72 (*s*, 3H), 1.67 (*s*, 3H), 1.53 (*d, J* = 4.2 Hz, 1H), 1.52−1.07 (*m*,14H), 1.03 (*s*, 3H), 0.98 (*s*, 3H), 0.86 (*s*, 3H).^{13}C NMR (75 MHz, $CDCl_3$): δ ppm 176.19, 172.76, 149.54, 147.17, 136.9, 127.91, 122.31, 122.09, 122.07, 119.58, 119.07, 114.28, 111.35, 110.93, 75.92, 71.55, 64.23, 56.68, 50.57, 50.11, 49.82, 49.76,

47.51, 44.29, 43.96, 42.27, 41.96, 37.89, 37.07, 35.35, 33.04, 32.51, 32.35, 30.92, 29.91, 29.53, 28.92, 27.12, 26.17, 25.84, 24.99, 22.78, 19.21, 19.09, 17.98, 14.08. HRMS Calcd for $C_{46}H_{66}N_2O_5$ $[M+H]^+$: 725.48990; found: 725.48634. See Figures S74 and S75.

10-(4-formyl-1*H*-imidazol-1-yl)decyl-chiisanogenin (Compound **I-37**). Chloroform / methanol = 60:1 / 50:1, white solid, purity of 98.2%, yield 65%. mp 115-116 °C. ^{1}HNMR(300 MHz, $CDCl_3$): δ ppm9.86 (*d, J* = 5.8 Hz, 1H), 8.16 (*d, J* = 12.4 Hz, 1H), 7.71 (*d, J* = 11.3 Hz, 1H), 4.88 (*d, J* = 1.7 Hz, 1H), 4.83 (*d, J* = 2.1 Hz, 1H), 4.75 (*d, J* = 2.3 Hz, 1H), 4.62 (*brs*, 1H), 4.59(*q, J* = 8.9 Hz, 1H), 4.08−4.06 (*ddt, J* = 8.0,5.9, 3.6 Hz, 2H), 3.58 (*d, J* = 7.8 Hz, 1H), 3.43−3.38 (*t, J* = 6.8 Hz, 2H),2.98 (*dt, J* = 11.4 Hz, 1H), 2.95 (*d, J* = 14.8 Hz, 1H), 2.74 (*d, J* = 7.9 Hz, 1H), 2.69 (*d, J* = 7.9 Hz, 1H), 2.45 (*d, J* = 3.8 Hz, 1H), 2.31 (*d, J* = 9.7Hz, 1H), 2.36−2.27 (*m*, 1H), 2.24 (*d, J* = 3.3 Hz, 1H), 2.01−1.84 (*m*, 6H), 1.82 (*d, J* = 3.6 Hz, 1H), 1.73 (*s*, 3H), 1.66 (*s*, 3H), 1.60 (*d, J* = 6.7Hz, 1H), 1.53−1.09 (*m*, 22H), 1.03 (*s*, 3H), 1.01 (*s*, 3H), 0.88 (*s*, 3H). ^{13}C NMR (75 MHz, $CDCl_3$): δ ppm185.56, 176.14, 173.47, 149.42, 146.94, 140.9, 138.88, 124.51, 114.33, 110.89, 75.8, 71.15, 64.19, 56.4, 50.06, 49.33, 48.17, 47.24, 43.87, 43.77, 41.94, 41.55, 37.71, 36.96, 34.94, 32.65, 32.08, 30.86, 30.71, 30.55, 29.53, 29.41, 29.23, 29.1, 28.76, 26.51, 26.38, 26.12, 24.72, 22.78, 19.16, 19.02, 17.9, 13.86. HRMS Calcd for $C_{44}H_{66}N_2O_6$ $[M+H]^+$: 717.48481; found: 717.48241. See Figures S76 and S77.

10-(2-propyl-1*H*-imidazol-1-yl)decyl-chiisanogenin (Compound **I-38**). Chloroform / methanol = 50:1 / 40:1, yellow solid, purity of 96.2%, yield 65%. mp 120-121 °C. ^{1}HNMR(300 MHz, $CDCl_3$): δ ppm7.13 (*d, J* = 1.7 Hz, 1H), 6.90 (*d, J* = 1.7 Hz, 1H), 4.85 (*d, J* = 1.6 Hz, 1H), 4.83 (*d, J* = 5.9 Hz, 1H), 4.74 (*d, J* = 2.3 Hz, 1H), 4.62 (*brs*, 1H), 4.52 (*q, J* = 8.9 Hz, 1H), 4.08−4.06 (*ddt, J* = 8.0,5.9, 3.6 Hz, 2H), 3.56 (*d, J* = 7.8 Hz, 1H), 3.43−3.38 (*t, J* = 6.8 Hz, 2H),2.98 (*dt, J* = 10.8 Hz, 1H), 2.95 (*d, J* = 14.8 Hz, 1H), 2.75 (*d, J* = 8.0 Hz, 1H), 2.68 (*d, J* = 7.9 Hz, 1H), 2.45 (*d, J* = 14.3 Hz, 1H), 2.39 (*d, J* = 4.5Hz, 1H), 2.36−2.27 (*m*, 5H), 2.24 (*d, J* = 3.3 Hz, 1H), 2.01−1.84 (*m*, 7H), 1.82 (*d, J* = 3.6 Hz, 1H), 1.72 (*s*, 3H), 1.67 (*s*, 3H), 1.54 (*d, J* = 3.4Hz, 1H), 1.53−1.09 (*m*, 24H), 1.04 (*s*, 3H), 1.0 (*s*, 3H), 0.99 (*s*, 3H),0.87 (*s*, 3H).^{13}C NMR (75 MHz, CDCl3): δ ppm 176.04, 173.35, 149.29, 147.95, 146.99, 131.89, 126.07, 118.94, 114.26, 110.99, 77.59, 77.16, 76.74, 75.68, 71.12, 63.82, 56.38, 50.14, 49.31, 47.20, 45.93, 43.87, 43.79, 41.93, 41.53, 37.68, 36.93, 34.89, 32.59, 32.09, 30.90, 30.49, 29.77, 29.18, 28.60, 28.48, 26.36, 25.78, 24.68, 22.71, 21.55, 19.15, 18.96, 17.88, 14.05, 13.83.

HRMS Calcd for $C_{46}H_{72}N_2O_5$ $[M+H]^+$: 731.53685; found: 731.53432. See Figures S78 and S79.

10-(2,4-dimethyl-1*H*-imidazol-1-yl)decyl-chiisanogenin (Compound **I-39**). Chloroform / methanol = 50:1 / 40:1, white solid, purity of 97.4%, yield 73%. mp 98-99 °C. ^{1}HNMR(300 MHz, $CDCl_3$): δ ppm 6.6 (*d, J* = 7.5 Hz, 1H), 4.85 (*d, J* = 1.6 Hz, 1H), 4.83 (*d, J* = 6.6 Hz, 1H), 4.74 (*d, J* = 2.0 Hz, 1H), 4.62 (*brs*, 1H), 4.51 (*q, J* = 8.8 Hz, 1H), 4.08−4.06 (*ddt, J* = 8.0,5.9, 3.6 Hz, 2H), 3.56 (*d, J* = 7.8 Hz, 1H), 3.43−3.38 (t, J = 6.8 Hz, 2H),2.98 (*dt, J* = 11.4 Hz, 1H), 2.91 (*d, J* = 14.8 Hz, 1H), 2.75 (*d, J* = 8.0 Hz, 1H), 2.67 (*d, J* = 8.0 Hz, 1H), 2.48 (*d, J* = 11.6 Hz, 1H), 2.39 (*d, J* = 4.5Hz, 1H), 2.36−2.27 (*m*, 6H), 2.24 (*d, J* = 3.3 Hz, 1H), 2.01−1.84 (*m*, 7H), 1.82 (*d, J* = 3.6 Hz, 1H), 1.72 (s, 3H), 1.67 (*s*, 3H), 1.54 (*d, J* = 3.4Hz, 1H), 1.53−1.09 (*m*, 22H), 1.04 (*s*, 3H), 1.00 (*s*, 3H), 0.88 (*s*, 3H). ^{13}C NMR (75 MHz, $CDCl_3$): δ ppmB176.17, 173.38, 149.4, 147.04, 143.53, 134.76, 115.5, 114.24, 110.97, 75.79, 71.14, 64.18, 56.42, 49.35, 47.27, 46.06, 45.48, 43.89, 43.81, 41.96, 41.58, 37.68, 36.97, 34.96, 32.1, 30.81, 30.54, 29.79, 29.59, 29.48, 29.25, 28.78, 28.68, 26.89, 26.71, 26.13, 25.31, 24.71, 22.7, 19.14, 18.53, 17.92, 13.86, 13.29, 12.6. HRMS Calcd for $C_{45}H_{70}N_2O_5$ $[M+H]^+$: 717.52120; found: 717.52345. See Figures S80 and S81.

10-(4-bromo-1*H*-imidazol-1-yl)decyl-chiisanogenin (Compound **I-40**). Chloroform / methanol = 55:1 / 45:1, white solid, purity of 95.6%, yield 63%. mp 123-135 °C. ^{1}H NMR (300 MHz, $CDCl_3$): δ ppm 8.27 (*d, J* = 5.9 Hz, 1H), 7.07 (*d, J* = 3.6 Hz, 1H), 4.85 (*d, J* = 1.6 Hz, 1H), 4.83 (*d, J* = 2.3 Hz, 1H), 4.74 (*d, J* = 2.3 Hz, 1H), 4.60 (*brs*, 1H), 4.51 (q, J = 8.8 Hz, 1H), 4.08−4.06 (ddt, J = 8.0, 5.9, 3.6 Hz, 2H), 3.58 (*d, J* = 7.7 Hz, 1H), 3.43−3.38 (*t, J* = 6.8 Hz, 2H), 2.99 (*dt, J* = 11.4 Hz, 1H), 2.91 (*d, J* = 14.8 Hz, 1H), 2.73 (*d, J* = 8.0 Hz, 1H), 2.69 (*d, J* = 7.9 Hz, 1H), 2.47 (*d, J* = 11.6 Hz, 1H), 2.39 (*d, J* = 4.5 Hz, 1H), 2.36−2.27 (*m*, 1H), 2.28 (*d, J* = 3.3 Hz, 1H), 1.97−1.85 (*m*, 6H), 1.82 (*d, J* = 3.6 Hz, 1H), 1.73 (*s*, 3H), 1.60 (*s*, 3H), 1.54 (*d, J* = 3.4 Hz, 1H), 1.64−1.10 (*m*, 22H), 1.03 (*s*, 3H), 1.00 (*s*, 3H), 0.88 (*s*, 3H).^{13}C NMR (75 MHz, $CDCl_3$): δ ppm 176.15, 173.41, 149.42, 146.98, 136.89, 118.64, 114.34, 110.92, 104.23, 75.78, 71.18, 64.19, 56.41, 50.12, 49.34, 48.36, 47.25, 43.89, 43.79, 41.95, 41.56, 37.69, 36.97, 34.95, 32.64, 32.09, 31.34, 30.79, 30.56, 29.79, 29.53, 29.41, 29.24, 29.11, 28.77, 26.48, 26.12, 24.71, 22.87, 19.16, 19.02, 17.91, 13.87. HRMS Calcd for $C_{43}H_{65}BrN_2O_5$ $[M+H]^+$: 767.40041; found: 767.40247. See Figures S82 and S83.

10-(2-ethyl-4-methyl-1*H*-imidazol-1-yl)decyl-chiisanogenin (Compound **I-41**). Chloroform / methanol = 45:1 / 40:1, white solid, purity of 96.8%, yield 65%. mp 154-155 °C. ^{1}H NMR (300 MHz, $CDCl_3$): δ ppm 6.65 (*d, J* = 3.5 Hz, 1H), 4.85 (*d, J* = 1.6 Hz, 1H), 4.83 (*d, J* = 2.3 Hz, 1H), 4.74 (*d, J* = 2.3 Hz, 1H), 4.60 (*brs*, 1H), 4.51 (*q, J* = 8.8 Hz, 1H), 4.05−4.06 (*ddt, J* = 8.0, 5.9, 3.6 Hz, 2H), 3.58 (*d, J* = 7.7 Hz, 1H), 3.43−3.38 (*t, J* = 6.8 Hz, 2H), 2.99 (*dt, J* = 11.4 Hz, 1H), 2.91 (*d, J* = 14.8 Hz, 1H), 2.73 (*d, J* = 8.0 Hz, 1H), 2.69 (*d, J* = 7.9 Hz, 1H), 2.47 (*d, J* = 11.6 Hz, 1H), 2.39 (*d, J* = 4.5 Hz, 1H), 2.36−2.27 (*m*, 2H), 2.28 (*d, J* = 3.3 Hz, 1H), 1.97−1.85 (*m*, 10H), 1.82 (*d, J* = 3.6 Hz, 1H), 1.73 (*s*, 3H), 1.60 (*s*, 3H), 1.54 (*d, J* = 3.4 Hz, 1H), 1.53−1.09 (*m*, 25H), 1.03 (*s*, 3H), 1.00 (*s*, 3H), 0.88 (*s*, 3H). ^{13}C NMR (75 MHz, $CDCl_3$): δ ppm 176.58, 173.83, 149.85, 147.40, 146.01, 137.31, 114.77, 113.66, 111.34, 78.01, 71.61, 64.61, 56.84, 50.55, 49.76, 48.78, 47.67, 44.32, 44.22, 42.38, 41.99, 38.11, 37.39, 35.38, 33.06, 32.51, 31.76, 31.21, 30.98, 30.22, 29.96, 29.83, 29.66, 29.54, 29.31, 29.20, 26.91, 26.55, 26.03, 25.14, 23.30, 19.59, 19.44, 18.34, 14.29, 13.26. HRMS Calcd for $C_{46}H_{72}N_2O_5$ $[M+H]^+$: 731.53685; found: 731.53501. See Figures S84 and S85.

10-(pyrrolidin-1-yl)decyl-chiisanogenin (Compound **I-42**). Chloroform / methanol = 45:1/40:1, white solid, purity of 97.9%, yield 43%. mp 124-125 °C. ^{1}H NMR (300 MHz, $CDCl_3$): δ ppm 4.85 (*d, J* = 1.6 Hz, 1H), 4.83 (*d, J* = 2.3 Hz, 1H), 4.74 (*d, J* = 2.3 Hz, 1H), 4.62 (*brs*, 1H), 4.51 (*q, J* = 8.8 Hz, 1H), 4.08−4.05 (*ddt, J* = 8.0, 5.9, 3.6 Hz, 2H), 3.56 (*d, J* = 7.7 Hz, 1H), 3.43−3.38 (*t, J* = 6.8 Hz, 2H), 2.98 (*dt, J* = 11.4 Hz, 1H), 2.92 (*d, J* = 14.8 Hz, 1H), 2.70 (*d, J* = 8.0 Hz, 1H), 2.67 (*d, J* = 7.9 Hz, 1H), 2.48 (*d, J* = 11.6 Hz, 1H), 2.39 (*d, J* = 4.5 Hz, 1H), 2.50−2.21 (*m*, 9H), 2.24 (*d, J* = 3.3 Hz, 1H), 2.00−1.77 (*m*, 7H), 1.82 (*d, J* = 3.6 Hz, 1H), 1.73 (*s*, 3H), 1.67 (*s*, 3H), 1.60 (*d, J* = 3.4 Hz, 1H), 1.64−1.08 (*m*, 22H), 1.05 (*s*, 3H), 1.01 (*s*, 3H), 0.88 (*s*, 3H). ^{13}C NMR (75 MHz, $CDCl_3$): δ ppm 175.37, 173.33, 148.71, 146.64, 114.41, 110.19, 75.09, 70.98, 63.92, 56.47, 55.83, 53.99, 50.02, 49.54, 47.87, 46.24, 42.73, 42.44, 41.15, 40.93, 37.02, 36.37, 35.27, 32.24, 31.33, 31.06, 30.32, 29.92, 29.54, 29.15, 28.85, 27.59, 26.96, 25.83, 25.82, 24.74, 23.50, 21.73, 21.56, 18.54, 18.17, 18.11, 17.33, 13.88. HRMS Calcd for $C_{44}H_{71}NO_5$ $[M+H]^+$: 692.52595; found: 692.52428. See Figures S86 and S87.

10-((2-(5-methoxy-1*H*-indol-3-yl)ethyl)amino)decyl-chiisanogenin (Compound **I-43**). Chloroform / methanol = 35:1 / 25:1, white solid, purity of 98.2%, yield 75%. mp 113-

114 °C. ^{1}H NMR (300 MHz, $CDCl_3$): δ ppm 8.61 (*brs*, 1H), 7.23 (*d, J* = 3.1 Hz, 1H), 7.15 – 7.05 (*m*, 1H),6.81 (*dd, J* = 8.8, 2.4 Hz, 1H),4.85 (*d, J* = 1.6 Hz, 1H), 4.83 (*d, J* = 2.3 Hz, 1H), 4.74 (*d, J* = 2.3 Hz, 1H), 4.63 (*brs*, 1H), 4.44 (*q, J* = 8.8 Hz, 1H), 4.08−4.04 (*ddt,* J = 8.0, 5.9, 3.6 Hz, 2H), 3.54 (*d, J* = 7.7 Hz, 1H), 3.34−3.13 (*t, J* = 6.8 Hz, 2H), 3.01 (*dt, J* = 11.4 Hz, 1H), 2.95 (*d, J* = 14.8 Hz, 1H), 2.82 (*d, J* = 8.0 Hz, 1H), 2.64 (*d, J* = 7.9 Hz, 1H), 2.48 (*d, J* = 11.6 Hz, 1H), 2.39 (*d, J* = 4.5 Hz, 1H), 2.36−2.27 (*m,* 9H), 2.29 (*d, J* = 3.3 Hz, 1H), 2.01−1.84 (*m*, 7H), 1.89 (*d, J* = 3.6 Hz, 1H), 1.75 (*s*, 3H), 1.64 (*s*, 3H), 1.57 (*d, J* = 3.4 Hz, 1H), 1.62−1.03 (*m*, 22H), 1.00 (*s*, 3H), 0.96 (*s*, 3H), 0.85 (*s*, 3H).^{13}C NMR (75 MHz, $CDCl_3$): δ ppm 176.07, 171.78, 154.18, 149.73, 146.92, 140.16, 132.04, 127.47, 124.33, 114.36, 112.42, 110.94, 110.81, 109.89, 76.13, 70.23, 65.66, 56.45, 56.15, 50.03, 49.53, 48.19, 48.08, 47.38, 43.84, 43.69, 41.95, 41.60, 37.69, 37.01, 35.06, 32.71, 32.10, 32.06, 30.75, 29.80, 29.56, 29.42, 29.20, 29.16, 29.09, 28.78, 26.85, 26.51, 26.21, 24.68, 22.78, 19.02, 18.52, 17.87, 13.85. HRMS Calcd for $C_{51}H_{76}N_2O_6$ $[M+H]^+$: 811.56306; found: 811.56127. See Figures S88 and S89.

10-((2-(thiophen-2-yl)ethyl)amino)decyl-chiisanogenin (Compound **I-44**). Chloroform / methanol = 50:1 / 40:1, white solid, purity of 97.3%, yield 56%. mp 121-122 °C. ^{1}H NMR (300 MHz, $CDCl_3$): δ ppm 7.13 (*d, J* = 2.4 Hz, 1H), 6.90 (*d, J* = 2.2 Hz, 1H), 6.60(*d, J* = 7.4 Hz, 1H), 4.85 (*d, J* = 1.6 Hz, 1H), 4.83 (*d, J* = 2.3 Hz, 1H), 4.74 (*d, J* = 2.3 Hz, 1H), 4.62 (*brs*, 1H), 4.50 (*q, J* = 8.8 Hz, 1H), 4.08−4.05 (*ddt, J* = 8.0, 5.9, 3.6 Hz, 2H), 3.56 (*d, J* = 7.7 Hz, 1H), 3.43−3.38 (*t, J* = 6.8 Hz, 2H), 2.98 (*dt, J* = 11.4 Hz, 1H), 2.81 (*d, J* = 14.8 Hz, 1H), 2.73 (*d, J* = 8.0 Hz, 1H), 2.68 (*d, J* = 7.9 Hz, 1H), 2.45 (*d, J* = 11.6 Hz, 1H), 2.39 (*d, J* = 4.5 Hz, 1H), 2.34−2.21 (*m*, 2H), 2.24 (*d, J* = 3.3 Hz, 1H), 1.94−1.81 (*m*, 10H), 1.80 (*d, J* = 3.6 Hz, 1H), 1.72 (*s*, 3H), 1.66 (*s*, 3H), 1.54 (*d, J* = 3.4 Hz, 1H), 1.64−1.09 (*m*, 22H), 1.04 (*s*, 3H), 1.01 (*s*, 3H), 0.87 (*s*, 3H). ^{13}C NMR (75 MHz, $CDCl_3$): δ ppm 176.56, 173.72, 150.31, 147.44, 125.90, 124.64, 121.74, 119.49, 114.68, 111.36, 78.01, 71.57, 64.58, 56.82, 50.56, 49.74, 47.67, 46.68, 44.30, 44.21, 42.36, 41.97, 38.09, 37.37, 35.36, 33.02, 32.50, 31.31, 30.95, 30.20, 29.97, 29.88, 29.78, 29.67, 29.61, 29.18, 28.58, 27.10, 26.53, 25.12, 23.29, 21.94, 19.57, 19.40, 18.33, 14.27. HRMS Calcd for $C_{46}H_{71}NO_5S$ $[M+H]^+$: 748.49802; found: 748.49634. See Figures S90 and S91.

10-((2-(1*H*-indol-3-yl)ethyl)amino)decyl-chiisanogenin (Compound **I-45**). Chloroform / methanol = 50:1/40:1, white solid, purity of 98.4%, yield 77%. mp 125-126 °C.

^{1}HNMR(300 MHz, $CDCl_3$): δ ppm 8.41 (*d, J* = 12.2 Hz, 1H),7.62 (*d, J* = 7.8 Hz, 1H),7.37 (*d, J* = 8.0 Hz, 1H), 7.18 (*t, J* = 7.5 Hz, 1H), 7.09 (*t, J* = 8.4 Hz, 1H), 7.05 (*d, J* = 2.3 Hz, 1H), 4.84 (*d, J* = 3.8 Hz, 2H), 4.75 (*d, J* = 2.3Hz, 1H), 4.62 (*brs*, 1H), 4.44 (*q, J* = 8.9 Hz, 1H), 4.09−3.99 (*dt, J* =13.6, 6.4 Hz, 4H), 3.54 (*d, J* = 7.7 Hz, 1H), 3.04−3.01 (*dd, J* = 13.5, 5.5Hz, 2H), 2.95-2.90 (*m*, 1H), 2.86 (*d, J* = 14.8 Hz, 1H), 2.84−2.65(*t, J* = 7.9 Hz, 2H), 2.86 (*d, J* = 8.0 Hz, 1H), 2.69 (*d, J* = 7.9 Hz, 1H),2.47 (*d, J* = 10.3 Hz, 1H), 2.38 (*d, J* = 7.0 Hz, 1H), 2.35−2.27 (*m*, 3H), 2.24 (*d, J* = 3.2 Hz, 1H), 1.94−1.83 (*m*, 5H), 1.80 (*d, J* = 4.9 Hz, 1H),1.72 (*s*, 3H), 1.66 (*s*, 3H), 1.53 (*d, J* = 5.9 Hz, 1H), 1.51−1.04 (*m*, 22H),1.02 (*s*, 3H), 0.97 (*s*, 3H), 0.86 (*s*, 3H).^{13}C NMR (75 MHz, $CDCl_3$): δ ppm 176.05, 173.52, 149.27, 146.80, 136.34, 127.15, 122.41, 121.91, 119.20, 118.66, 114.20, 112.49, 111.26, 110.75, 75.71, 70.97, 64.09, 56.25, 49.96, 49.22, 49.14, 47.64, 47.10, 43.69, 43.59, 41.76, 41.38, 37.51, 36.80, 34.77, 32.44, 31.91, 30.38, 29.41, 29.35, 29.28, 29.18, 29.08, 29.04, 28.60, 28.49, 27.09, 25.98, 24.53, 23.65, 22.57, 18.97, 18.83, 17.72, 13.66. HRMS Calcd for $C_{50}H_{74}N_2O_5$ $[M+H]^+$: 781.55250; found: 781.55015. See Figures S92 and S93.

3-(4-formyl-1*H*-imidazol-1-yl)propyl(5a*R*,5b*R*,7a*S*,10*R*,10a*R*,10b*R*,12*R*,12a*S*,12b*R*)-12-hydroxy-1-(2-methoxy-2-oxoethyl)-3,3,5a,5b,12b-pentamethyl-10-(prop-1-en-2-yl)octadecahydro-7a*H*-cyclopenta[7,8]phenanthro[1,2-c]furan-7a-carboxylate (Compound **II-46**). Chloroform / methanol = 60:1 / 50:1, white solid, purity of 96.4%, yield 65%. mp 108-109 °C. ^{1}H NMR (300 MHz,$CDCl_3$): δ ppm 9.75 (*d, J* = 1.0 Hz, 1H) 7.82 (*d, J* = 0.9 Hz, 1H), 7.57 (*d, J* = 1.3 Hz, 1H),4.74 (*brs*, 1H), 4.62 (*brs*, 1H), 4.26 (*dd, J* = 8.6, 2.7Hz, 1H), 4.24−4.18 (*m*, 2H), 3.93 (*dt, J* = 10.9, 5.0 Hz, 1H), 3.66 (*s*, 3H), 3.49−3.45 (*t, J* = 6.5 Hz, 2H), 3.03 (*d, J* = 2.8 Hz, 1H), 2.96-2.91 (*m*, 1H), 2.31 (*d, J* = 2.6 Hz, 1H), 2.30−2.24 (*m*, 1H), 2.22 (*dt, J*= 6.3 Hz, 1H), 2.20−2.15 (*dt, J* = 6.3 Hz, 2H), 1.97−1.88 (*m*, 7H), 1.69(*s*, 3H), 1.68 (*d, J* = 2.8 Hz, 1H), 1.63 (*d, J* = 3.2 Hz, 1H), 1.58−1.10(*m*, 11H), 1.24 (*s*, 3H), 1.16 (*s*, 3H), 1.09 (*s*, 3H), 1.00 (*s*, 3H), 0.91 (*s*,3H).^{13}C NMR (75 MHz, CDCl3): δ ppm 186.39, 179.32, 173.39, 149.64, 142.8, 138.79, 124.15, 110.59, 86.47, 79.71, 68.36, 60.15, 56.65, 55.63, 51.68, 48.94, 48.04, 46.89, 46.44, 44.64, 42.52, 42.47, 37.98, 37.21, 36.98, 36.1, 35.25, 32.35, 32.13, 30.53, 30.35, 29.99, 24.49, 19.44, 18.96, 18.52, 17.63, 14.99. HRMS Calcd for $C_{38}H_{56}N_2O_7$ $[M+H]^+$: 651.40148; found: 651.40352. See Figures S94 and S95.

3-(2-propyl-1*H*-imidazol-1-yl)propyl-acanthosessiligenins (Compound **II-47**). Chloroform / methanol = 50:1 / 40:1, white solid, purity of 98.0%, yield 73%. mp 120-121 °C. ^{1}H NMR (300 MHz, $CDCl_3$): δ ppm 7.04 (*d, J* = 0.6 Hz, 1H), 6.95 (*d, J* = 1.3 Hz, 1H), 6.82 (*d, J* = 1.4 Hz, 1H). 4.75 (*brs*, 1H), 4.62 (*brs*, 1H), 4.24 (*dd, J* = 8.5, 2.6Hz, 1H), 4.26−4.18 (*m*, 2H), 3.94 (*dt, J* = 10.9, 5.0 Hz, 1H), 3.65 (*s*,3H), 3.49−3.45 (*t, J* = 6.5 Hz, 2H), 3.07 (*d, J* = 2.8 Hz, 1H), 2.96-2.92 (*m*, 1H), 2.31 (*d, J* = 2.6 Hz, 1H), 2.30−2.24 (*m*, 3H), 2.22 (*dt, J*= 6.3 Hz, 1H), 2.21−2.16 (*dt, J* = 6.3 Hz, 2H), 1.97−1.88 (*m*, 3H), 1.69(*s*, 3H), 1.66 (*d, J* = 2.8 Hz, 1H), 1.61 (*d, J* = 3.2 Hz, 1H), 1.58−1.10(*m*, 13H), 1.22 (*s*, 3H), 1.20 (*s*, 3H), 1.18 (*s*,3H),1.16 (*s*, 3H), 1.13 (*s*, 3H),0.97(*s*, 3H).^{13}C NMR (75 MHz, CDCl3): δ ppm 175.68, 173.32, 149.66, 148.09, 127.43, 118.83, 110.47, 86.45, 79.61, 68.1, 60.57, 56.58, 55.58, 51.62, 48.94, 47.99, 46.88, 46.43, 45.49, 42.59, 42.45, 37.98, 37.16, 36.96, 36.14 ,35.23, 32.35, 32.12, 30.39, 29.95, 29.66, 28.73, 24.48, 21.51, 19.41, 18.92, 18.51, 17.62 ,14.96, 13.79. HRMS Calcd for $C_{40}H_{62}N_2O_6$ $[M+H]^+$: 665.45351; found: 665.45578. See Figures S96 and S97.

3-(2,4-dimethyl-1*H*-imidazol-1-yl)propyl-acanthosessiligenins (Compound **II-48**). Chloroform/methanol = 60:1/50:1, white solid, purity of 99.1%, yield 65%. mp 142-143 °C. ^{1}H NMR (300 MHz, $CDCl_3$): δ ppm 6.62 (*d, J* = 48.1 Hz, 1H),4.74 (*brs*, 1H), 4.62 (*brs*, 1H), 4.28 (*dd, J* = 8.7, 2.7 Hz, 1H), 4.24−4.19 (*m*, 2H), 4.07 (*dt, J* = 10.9, 5.0 Hz, 1H), 3.66 (*s*, 3H), 3.49−3.45 (*t, J* = 6.5 Hz, 2H), 3.08 (*d, J* = 2.8 Hz, 1H), 3.03-2.96 (*m*, 1H), 2.31 (*d, J* = 2.6 Hz, 1H), 2.30−2.24 (*m*, 4H), 2.22 (*dt, J* = 6.3 Hz, 1H), 2.20−2.15 (*dt, J* = 6.3 Hz, 2H), 2.09−1.80 (*m*, 7H), 1.68 (*s*, 3H), 1.64 (*d, J* = 2.8 Hz, 1H), 1.54 (*d, J* = 3.2 Hz, 1H), 1.66−1.12(*m*, 11H), 1.23 (*s*, 3H), 1.17 (*s*, 3H), 1.09 (*s*, 3H), 1.01 (*s*, 3H), 0.92 (*s*, 3H). ^{13}C NMR (75 MHz, $CDCl_3$): δ ppm 175.71, 173.34, 149.62, 143.47, 134.93, 115.65, 110.44, 86.45, 79.57, 68.04, 60.43, 56.58, 55.58, 51.59, 48.93, 48.01, 46.84, 46.4, 42.94, 42.45, 42.42, 37.92, 37.18, 36.94, 36.12, 35.22, 32.31, 32.12, 30.51, 29.94, 29.75, 24.44, 19.41, 18.9, 18.48, 17.61, 14.94, 12.78, 12.39. HRMS Calcd for $C_{39}H_{60}N_2O_6$ $[M+H]^+$: 651.43786; found: 651.43975. See Figures S98 and S99.

(5-bromo-1*H*-imidazol-1-yl)propyl-acanthosessiligenins (Compound **II-49**). Chloroform/methanol = 55:1/45:1, white solid, purity of 95.3%, yield 67%. mp 108-109 °C. ^{1}H NMR (300 MHz, $CDCl_3$): δ ppm 7.49 (*d, J* = 68.2 Hz, 1H), 7.06 – 6.88 (*m*, 1H), 4.74 (*brs*, 1H), 4.62 (*brs*, 1H), 4.24 (*dd, J* = 8.7, 2.7 Hz, 1H), 4.24−4.04 (*m*, 2H), 3.93 (*dt, J* = 10.9, 5.0 Hz, 1H), 3.64 (s, 3H), 3.15−3.08 ((*t, J* = 6.5 Hz, 2H), 3.07 (*d, J*

= 2.8 Hz, 1H), 2.96-2.90 (*m*, 1H), 2.31 (*d, J* = 2.6 Hz, 1H), 2.30−2.24 (*m*, 1H), 2.22 (*dt, J* = 6.3 Hz, 1H), 2.20−2.14 (*dt, J* = 6.3 Hz, 2H), 1.97−1.87 (*m*, 4H), 1.68 (s, 3H), 1.64 (*d, J* = 2.8 Hz, 1H), 1.62 (*d, J* = 3.2 Hz, 1H), 1.56−1.08(*m*, 11H), 1.24 (*s*, 3H), 1.16 (*s*, 3H), 1.11 (*s*, 3H), 1.01 (*s*, 3H), 0.91 (*s*, 3H). ^{13}C NMR (75 MHz, CDCl3): δ ppm 176.2, 173.19, 149.65, 137.46, 129.94, 110.31, 104.32, 86.19, 79.7, 68.24, 64.15, 56.25, 55.44, 51.56, 48.58, 47.87, 46.69, 46.28, 44.37, 42.34, 42.31, 37.85, 37.28, 36.89, 35.9, 35.04, 32.19, 32.09, 30.37, 29.83, 28.36, 24.24, 19.31, 18.78, 18.34, 17.45, 14.84. HRMS Calcd for $C_{37}H_{55}BrN_2O_6$ $[M+H]^+$: 701.31707; found: 701.31578. See Figures S100 and S101.

3-(2-ethyl-4-methyl-1*H*-imidazol-1-yl)propyl-acanthosessiligenins (Compound **II-50**). Chloroform / methanol = 55:1 / 45:1, white solid, purity of 97.3%, yield 60%. mp 114-115 °C. ^{1}H NMR (300 MHz, $CDCl_3$): δ ppm 6.54 (*brs*, 1H), 4.75 (*brs*, 1H), 4.62 (*brs*, 1H), 4.26 (*dd, J* = 8.7, 2.7 Hz, 1H), 4.24−4.08 (*m*, 2H), 3.95 (*dt, J* = 10.9, 5.0 Hz, 1H), 3.66 (*s*, 3H), 3.49−3.45 (*t, J* = 6.5 Hz, 2H), 3.05 (*d, J* = 2.8 Hz, 1H), 2.69 (*dt, J* = 10.8 Hz, 1H), 2.30 (*d, J* = 2.6 Hz, 1H), 2.39−2.17 (*m*, 6H), 2.23 (*dt, J* = 6.3 Hz, 1H), 2.09−2.13 (*m*, 2H), 1.97−1.83 (*m*, 3H), 1.68 (*s*, 3H), 1.64 (*d*, J = 2.8 Hz, 1H), 1.58(*d, J* = 3.2 Hz, 1H), 1.49−1.07(*m*, 14H), 1.22 (*s*, 3H), 1.16 (*s*, 3H), 1.09 (*s*, 3H), 1.01 (*s*, 3H), 0.91 (*s*, 3H). ^{13}C NMR (75 MHz, $CDCl_3$): δ ppm 176.09, 173.31, 157.6, 149.8, 136.2, 115.2, 110.31, 86.44, 79.57,68.1, 63.35, 56.62, 55.57, 51.61, 48.9, 47.98, 46.42, 45.12, 42.47, 42.43, 38.02, 37.3, 37.02, 36.19, 35.21, 32.33, 32.22, 30.63, 29.91, 28.02, 26.12, 24.47, 20.2, 19.45, 18.9, 18.52, 17.58,8 14.98, 13.65, 12.62. HRMS Calcd for $C_{40}H_{62}N_2O_6$ $[M+H]^+$: 665.45351; found: 665.45374. See Figures S102 and S103.

3-(pyrrolidin-1-yl)propyl-acanthosessiligenins (Compound **II-51**). Chloroform / methanol = 60:1/50:1, white solid, purity of 96.8%, yield 64%. mp 134-135 °C. ^{1}H NMR (300 MHz, $CDCl_3$): δ ppm 4.74 (brs, 1H), 4.61 (brs, 1H), 4.26 (*dd, J* = 8.7, 2.7 Hz, 1H), 4.24−4.05 (*m*, 2H), 3.93 (*dt, J* = 10.9, 5.0 Hz, 1H), 3.63 (*s*, 3H), 3.49−3.45 (*t, J* = 6.5 Hz, 2H), 3.05 (*d, J* = 2.8 Hz, 1H), 2.99-2.96 (*m*, 1H), 2.30 (*d, J* = 2.6 Hz, 1H), 2.37−2.20 (*m*, 5H), 2.22 (*dt, J* = 6.3 Hz, 1H), 2.20−2.15 (*dt, J* = 6.3 Hz, 2H), 2.03−1.68 (*m*, 3H), 1.67 (*s*, 3H), 1.64 (*d, J* = 2.8 Hz, 1H), 1.61 (*d, J* = 3.2 Hz, 1H), 1.56−1.03(*m*, 16H), 1.23 (*s*, 3H), 1.17 (*s*, 3H), 1.09 (*s*, 3H), 0.99 (*s*, 3H), 0.92 (*s*, 3H). ^{13}C NMR (75 MHz, $CDCl_3$): δ ppm 176.20, 173.02, 149.51, 110.60, 86.48, 79.87, 67.81, 63.65, 56.57, 55.60, 53.86, 51.66, 50.77, 48.89, 47.99, 47.00, 46.40, 42.50, 42.46, 37.84, 37.27, 36.15,

35.17, 32.38, 32.19, 30.58, 29.88, 29.77, 28.43, 26.39, 24.52, 23.81, 23.43, 22.77, 19.43, 18.97, 17.63, 14.99. HRMS Calcd for $C_{38}H_{61}NO_6$ $[M+H]^+$: 626.44261; found: 626.44047. See Figures S104 and S105.

3-((2-(5-methoxy-1*H*-indol-3-yl)ethyl)amino)propyl-acanthosessiligenins (Compound **II-52**). Chloroform / methanol = 30:1 / 20:1, white solid, purity of 98.1%, yield 53%. mp 134-135 °C. ^{1}H NMR (300 MHz, $CDCl_3$): δ ppm 8.16 (*brs*, 1H), 7.24 (*d, J* = 4.7 Hz, 1H), 7.04 (*dd, J* = 6.4, 2.4 Hz, 1H), 6.85 (*dd, J* = 8.8, 2.4 Hz, 1H), 4.73 (*brs*, 1H), 4.61 (*brs*, 1H), 4.26 (*dd, J* = 8.7, 2.7 Hz, 1H), 4.24−4.11 (*m*, 2H), 3.91 (*dt, J* = 10.9, 5.0 Hz, 1H), 3.85 (*s*, 3H) ,3.66 (*s*,3H), 3.77−3.63 (*t, J* = 6.5 Hz, 2H), 3.05 (*d, J* = 2.8 Hz, 1H), 2.97-2.91 (*m*, 1H), 2.30 (*d, J* = 2.6 Hz, 1H), 2.37−2.24 (*m*, 3H), 2.22 (*dt, J* = 6.3 Hz, 1H), 2.20−2.17 (*dt, J* = 6.3 Hz, 2H), 2.00−1.77 (*m*, 3H), 1.67 (*s*, 3H), 1.63 (*d, J* = 2.8 Hz, 1H), 1.59 (*d, J* = 3.2 Hz, 1H), 1.55−1.04 (*m*, 16H), 1.23 (*s*, 3H), 1.13 (*s*, 3H), 1.09 (*s*, 3H), 0.98 (*s*, 3H), 0.88 (*s*, 3H). ^{13}C NMR (75 MHz, $CDCl_3$): δ ppm 176.17, 173.42, 153.99, 149.96, 131.75, 127.85, 123.11, 113.15, 112.22, 112.08, 110.3, 100.86, 86.48, 79.7, 68.31, 62.32, 56.08, 55.61, 51.63, 49.93, 48.91, 47.98, 46.97, 46.61, 46.43, 42.47, 42.43, 38, 37.26, 37.02, 36.16, 35.2, 32.36, 32.19, 30.61, 29.89, 29.14, 28.68, 25.61, 24.5, 19.43, 18.9, 18.5, 17.59, 14.97. HRMS Calcd for $C_{45}H_{66}N_2O_7$ $[M+H]^+$: 745.47973; found: 745.47347. See Figures S106 and S107.

3-((2-(thiophen-2-yl)ethyl)amino)propyl-acanthosessiligenins (Compound **II-53**). Chloroform / methanol = 50:1 / 40:1, white solid, purity of 97.5%, yield 67%. mp 134-135 °C. ^{1}H NMR (300 MHz, $CDCl_3$): δ ppm 7.18 – 7.14 (*m*, 1H), 6.97 – 6.93 (*m*, 1H), 6.89 – 6.84 (*m*, 1H), 4.75 (*brs*, 1H), 4.63 (*brs*, 1H), 4.27 (*dd, J* = 8.7, 2.7 Hz, 1H), 4.44−4.09 (*m*, 2H), 4.15 (*dt, J* = 10.9, 5.0 Hz, 1H), 3.67 (*s*, 3H), 3.49−3.45 (*t, J* = 6.5 Hz, 2H), 3.17 (*d, J* = 2.8 Hz, 1H), 2.96-2.91 (*m*, 1H), 2.31 (*d, J* = 2.6 Hz, 1H), 2.34−2.27 (*m*, 1H), 2.02 (*dt, J* = 6.3 Hz, 1H), 2.24−2.20 (*dt, J* = 6.3 Hz, 2H), 1.99−1.81 (*m*, 8H), 1.68 (*s*, 3H), 1.64 (*d, J* = 2.8 Hz, 1H), 1.62 (*d, J* = 3.2 Hz, 1H), 1.62−1.10 (*m*, 12H), 1.24 (*s*, 3H), 1.17 (*s*, 3H), 1.10 (*s*, 3H), 1.00 (*s*, 3H), 0.92 (*s*, 3H).^{13}C NMR (75 MHz, $CDCl_3$): δ ppm 176.16, 173.31, 149.86, 127.36, 125.97, 124.24, 109.99, 86.07, 80.28, 68.53, 61.77, 56.64, 55.67, 51.71, 50.92, 49.49, 48.97, 48.04, 46.95, 46.50, 42.57, 42.54, 39.32, 38.07, 37.29, 37.04, 36.21, 35.30, 32.41, 32.21, 30.63, 28.95, 27.91, 27.10, 24.55, 19.50, 19.01, 18.58, 17.70, 15.03. HRMS Calcd for $C_{40}H_{61}NO_6S$ $[M+H]^+$: 682.41468; found: 682.41667. See Figures S108 and S109.

3-((2-(1*H*-indol-3-yl)ethyl)amino)propyl-acanthosessiligenins (Compound **II-54**). Chloroform / methanol = 30:1 / 20:1, white solid, purity of 95.1%, yield 74%. mp 111-112 °C. ^{1}H NMR (300 MHz, $CDCl_3$): δ ppm 8.22 (*d, J* = 8.5Hz, 1H),7.62 (*d, J* = 7.7Hz, 1H), 7.36 (*d, J*= 7.9 Hz, 1H), 7.19 (*td, J*= 6.9, 6.1, 1.2 Hz, 1H), 7.11 (*td, J* = 7.5, 7.0, 1.1 Hz, 1H), 7.05 (*d, J* = 2.3 Hz, 1H), 4.73 (*brs*, 1H),4.61 (*brs*, 1H), 4.24 (*dd, J* = 11.4, 2.8 Hz, 1H), 4.14−4.09 (*t, J* = 6.54Hz, 4H), 3.88 (*dt, J* = 10.7, 5.0 Hz, 1H), 3.66 (*s*, 3H), 3.07 (*d, J* = 2.8Hz, 1H), 3.00−2.98 (*t, J* = 6.9 Hz, 1H), 3.00−2.94 (*m*, 1H), 2.75−2.71(*t, J* = 7.2 Hz, 2H), 2.30 (*d, J* = 2.8 Hz, 1H), 2.35−2.20 (*m*, 2H), 1.98−1.79 (*m*, 5H), 1.67 (*s*, 3H), 1.63 (*d, J* = 3.7 Hz, 1H), 1.60 (*d, J* = 4.2 Hz, 1H), 1.56−1.06 (*m*, 14H), 1.24 (*s*, 3H), 1.14 (*s*, 3H), 1.10 (*s*, 3H), 0.98(*s*, 3H), 0.89 (*s*, 3H). ^{13}C NMR (75 MHz, $CDCl_3$): δ ppm 176.14, 173.38, 149.98, 136.56, 127.49, 122.22, 122.1, 119.35, 118.87, 113.63, 111.38, 110.31, 86.48, 79.67, 68.33, 62.37, 56.56, 55.6, 51.68, 50.04, 48.92, 47.96, 46.97, 46.64, 46.43, 42.47, 42.43, 38.01, 37.24, 37.01, 36.18, 35.2, 32.37, 32.19, 30.6, 29.89, 29.27, 25.74, 24.52, 19.44, 18.92, 18.5, 17.6, 14.97. HRMS Calcd for $C_{44}H_{64}N_2O_6$ $[M+H]^+$: 715.46916; found: 715.46725. See Figures S110 and S111.

4-(4-formyl-1*H*-imidazol-1-yl)butyl-acanthosessiligenins (Compound **II-55**). Chloroform / methanol = 60:1 / 50:1, white solid, purity of 99.8%, yield 67%. mp 109-110 °C. ^{1}H NMR (300 MHz,$CDCl_3$): δ ppm 9.73 (*d, J* =5.9 Hz, 1H), 7.81 (*d, J* = 6.1 Hz, 1H), 7.57 (*d, J* = 1.3 Hz, 1H), 4.72 (*brs*, 1H), 4.62 (*brs*, 1H), 4.32 (*dd, J* = 11.4, 2.9Hz, 1H), 4.15−4.06 (*m*, 2H), 3.95 (*dt, J* = 10.9, 5.1 Hz, 1H), 3.67 (*s*,3H), 3.46−3.42 (*t, J* = 6.5 Hz, 2H), 3.08 (*d, J* = 2.8 Hz, 1H), 3.03-2.96 (*m*, 1H), 2.31 (*d, J* = 4.1 Hz, 1H), 2.42−2.21 (*m*, 2H), 2.01−1.77 (*m*, 5H), 1.68 (*s*, 3H), 1.66 (*d, J* = 4.1 Hz, 1H), 1.60 (*d*, J = 4.8 Hz,1H), 1.72−1.15 (*m*, 15H), 1.22 (*s*, 3H), 1.15 (*s*, 3H), 1.09 (*s*, 3H), 0.99(*s*, 3H), 0. 88 (*s*, 3H).^{13}C NMR (75 MHz, $CDCl_3$): δ ppm 186.31, 179.28, 173.4, 149.74, 142.57, 138.76, 124.25, 110.33, 86.44, 79.64, 68.18, 62.87, 56.57, 55.5, 51.62, 48.87, 48, 47.28, 46.92, 46.39, 42.45, 42.4, 37.98, 37.26, 36.98, 36.06, 35.18, 32.31, 32.14, 30.53, 29.88, 27.78, 25.87, 24.45, 19.39, 18.91, 18.47, 17.56, 14.94. HRMS Calcd for $C_{39}H_{58}N_2O_7$ $[M+H]^+$: 665.41713; found: 665.41402. See Figures S112 and S113.

4-(2-propyl-1*H*-imidazol-1-yl)butyl-acanthosessiligenins (Compound **II-56**). Chloroform / methanol = 50:1 / 40:1, white solid, purity of 97.0%, yield 74%. mp 141-142 °C. ^{1}H NMR (300 MHz,$CDCl_3$): δ ppm 7.61 (*d, J* = 1.7 Hz, 1H), 7.14 (*d, J* = 1.4

Hz, 1H), 4.75 (*brs*, 1H), 4.61 (*brs*, 1H), 4.25 (*dd, J* = 10.3, 3.2Hz, 1H), 4.15−4.06 (*m*, 2H), 3.98 (*dt, J* = 10.3, 5.0 Hz, 1H), 3.67 (*s*, 3H), 3.46−3.42 (*t, J* = 6.5 Hz, 2H), 3.07 (*d, J* = 3.1 Hz, 1H), 2.97-2.94 (*m*, 1H), 2.31 (*d, J* = 4.1 Hz, 1H), 2.42−2.21 (*m*, 6H), 2.01−1.77 (*m*, 7H), 1.68 (*s*, 3H), 1.66 (*d, J* = 4.1 Hz, 1H), 1.61 (*d, J* = 4.3 Hz, 1H), 1.72−1.43 (*m*, 15H), 1.42 (*s*, 3H), 1.30 (*s*, 3H), 1.26 (*s*, 3H), 1.24(*s*, 3H),122 (*s*, 3H). ^{13}C NMR (75 MHz, $CDCl_3$): δ ppm 176.03, 173.38, 149.73, 148.8, 148.04, 118.79, 110.28, 86.46, 79.64, 67.76, 63.19, 56.55, 55.54, 51.53, 48.88, 48, 46.96, 46.39, 45.36, 42.4, 42.38, 37.94, 37.25, 36.96, 36.13, 35.14, 32.29, 32.15, 30.56, 30.39, 29.84, 28.7, 27.92, 24.42, 21.51, 19.35, 18.84, 18.44, 17.52, 14.91, 13.8. HRMS Calcd for $C_{41}H_{64}N_2O_6$ $[M+H]^+$: 679.46916; found: 679.46750. See Figures S114 and S115.

4-(2,4-dimethyl-1*H*-imidazol-1-yl)butyl-acanthosessiligenins (Compound **II-57**). Chloroform / methanol = 50:1 / 40:1, white solid, purity of 99.7%, yield 69%. mp 143-144 °C. ^{1}H NMR (300 MHz,$CDCl_3$): δ ppm 6.65 (*d, J* = 5.6Hz, 1H), 4.73 (*brs*, 1H), 4.62 (*brs*, 1H), 4.29 (*dd, J* = 10.3, 3.2Hz, 1H), 4.15−4.06 (*m*, 2H), 3.94 (*dt, J* = 10.3, 5.0 Hz, 1H), 3.66 (*s*, 3H), 3.46−3.42 (*t, J* = 6.5 Hz, 2H), 3.07 (*d, J* = 2.9 Hz, 1H), 3.03-2.96(*m*, 1H), 2.31 (*d, J* = 4.1 Hz, 1H), 2.42−2.21 (*m*, 8H), 2.01−1.77 (*m*, 5H), 1.68 (*s*, 3H), 1.67 (*d, J* = 4.1 Hz, 1H), 1.63 (*d, J* = 4.3 Hz, 1H), 1.72−1.15 (*m*, 14H), 1.23 (*s*, 3H), 1.15 (*s*, 3H), 1.09 (*s*, 3H), 0.99(*s*, 3H), 0.88 (*s*, 3H).^{13}C NMR (75 MHz, $CDCl_3$): δ ppm 176.08, 173.32, 149.78, 143.53, 135.98, 115.35, 110.31, 86.43, 79.56, 67.98, 63.28, 56.6, 55.57, 51.6, 48.9, 48.01, 47, 46.41, 45.46, 42.45, 42.42, 37.97, 37.3, 37, 36.16, 35.19, 32.31, 32.21, 30.61, 29.89, 27.74, 26.04, 24.45, 19.41, 18.88, 18.49, 17.56, 14.96, 13.45, 12.91. HRMS Calcd for $C_{40}H_{62}N_2O_6$ $[M+H]^+$: 665.45351; found: 665.45637. See Figures S116 and S117.

4-(4-bromo-1*H*-imidazol-1-yl)butyl-acanthosessiligenins (Compound **II-58**). Chloroform / methanol = 55:1/45:1, white solid, purity of 95.2%, yield 62%. mp 135-136 °C. ^{1}H NMR (300 MHz, $CDCl_3$): δ ppm 7.72 – 7.34 (*m*, 1H), 6.97 (*dd, J* = 44.5, 1.4 Hz, 1H), 4.73 (*brs*, 1H), 4.61 (*brs*, 1H), 4.29 (*dd, J* = 8.7, 2.7 Hz, 1H), 4.24−4.08 (*m*, 2H), 4.09 (*dt, J* = 10.9, 5.0 Hz, 1H), 3.64 (*s*, 3H), 3.49−3.45 (*t, J* = 6.5 Hz, 2H), 3.08 (*d, J* = 2.8 Hz, 1H), 2.95-2.90 (*m*, 1H), 2.32 (*d, J* = 2.6 Hz, 1H), 2.40−2.24 (*m*, 1H), 2.22 (*dt, J* = 6.3 Hz, 1H), 2.20−2.17 (*dt, J* = 6.3 Hz, 2H), 2.03−1.77 (*m*, 3H), 1.67 (*s*, 3H), 1.65 (*d, J* = 2.8 Hz, 1H), 1.61 (*d, J* = 3.2 Hz, 1H), 1.59−1.06(*m*, 14H), 1.24 (*s*, 3H), 1.16 (*s*, 3H), 1.11 (*s*, 3H), 1.00 (*s*, 3H), 0.88 (*s*, 3H).^{13}C NMR (75 MHz, $CDCl_3$):

δ ppm 176.42, 173.19, 149.65, 137.46, 129.94, 110.31, 104.32, 86.19, 79.7, 68.24, 63.76, 56.25, 55.44, 51.56, 49.3, 48.58, 47.87, 46.69, 46.28, 42.34, 42.31, 37.85, 37.28, 36.89, 35.9, 35.04, 32.19, 32.09, 30.37, 29.83, 26.55, 25.44, 24.24, 19.31, 18.78, 18.34, 17.45, 14.84. HRMS Calcd for $C_{38}H_{57}BrN_2O_6$ $[M+H]^+$: 715.33272; found: 715.33427. See Figures S118 and S119.

4-(2-ethyl-4-methyl-1*H*-imidazol-1-yl)butyl-acanthosessiligenins (Compound **II-59**). Chloroform / methanol = 50:1 / 40:1, white solid, purity of 98.1%, yield 63%. mp 108-109 °C. ^1H NMR (300 MHz, $CDCl_3$): δ ppm 6.49 (*brs*, 1H), 4.73 (*brs*, 1H), 4.62 (*brs*, 1H), 4.27 (*dd, J* = 10.3, 3.2 Hz, 1H), 4.15−4.07 (*m*, 2H), 3.76 (*dt, J* = 10.3, 5.0 Hz, 1H), 3.66 (*s*, 3H), 3.46−3.42 (*t, J* = 6.5 Hz, 2H), 3.05 (*d, J* = 3.1 Hz, 1H), 2.95-2.90 (*m*, 1H), 2.27 (*d, J* = 4.1 Hz, 1H), 2.42−2.20 (*m*, 6H), 1.92− 1.72 (*m*, 10H), 1.67 (*s*, 3H), 1.64 (*d, J* = 4.1 Hz, 1H), 1.60 (*d, J* = 4.3 Hz, 1H), 1.59−1.06 (*m*, 13H), 1.23 (*s*, 3H), 1.15 (*s*, 3H), 1.09 (*s*, 3H), 0.99 (*s*, 3H), 0.88 (*s*, 3H). ^{13}C NMR (75 MHz, $CDCl_3$): δ ppm 176.09, 173.35, 149.81, 149.11, 136.13, 115.22, 110.32, 86.45, 79.61, 68.11, 63.37, 56.61, 55.58, 51.63, 48.9, 47.99, 47.02, 46.42, 45.13, 42.47, 42.43, 37.99, 37.31, 37.03, 36.17, 35.21, 32.33, 32.22, 30.63, 29.9, 27.9, 26.11, 24.47, 20.16, 19.43, 18.89, 18.5, 17.57, 14.98, 13.55, 12.62. HRMS Calcd for $C_{41}H_{64}N_2O_6$ $[M+H]^+$: 679.46916; found: 679.46724. See Figures S120 and S121.

4-((2-(5-methoxy-1*H*-indol-3-yl)ethyl)amino)butyl-acanthosessiligenins (Compound **II-60**). Chloroform / methanol = 50:1 / 40:1, white solid, purity of 95.5%, yield 59%. mp 137-138 °C. ^1H NMR (300 MHz,$CDCl_3$): δ ppm 4.75 (*brs*, 1H), 4.62 (*brs*, 1H), 4.24 (*dd, J* = 10.3, 3.2Hz, 1H), 4.15−4.06 (*m*, 2H), 3.94 (*dt, J* = 10.3, 5.0 Hz, 1H), 3.67 (*s*,3H), 3.46−3.42 (*t*, J = 6.5 Hz, 2H), 3.03 (*d, J* = 3.1 Hz, 1H), 2.97-2.91 (*m*, 1H), 2.31 (*d, J* = 4.1 Hz, 1H), 2.42−2.21 (*m*, 4H), 2.01−1.77 (*m*, 10H), 1.67 (*s*, 3H), 1.63 (*d, J* = 4.1 Hz, 1H), 1.59 (*d, J* = 11.3 Hz, 1H), 1.72−1.15 (*m*, 15H), 1.22 (*s*, 3H), 1.16 (*s*, 3H), 1.08 (*s*, 3H), 0.99(*s*, 3H), 0.92 (*s*, 3H). ^{13}C NMR (75 MHz, $CDCl_3$): δ ppm 176.19, 173.39, 149.8, 110.35, 86.43, 79.62, 68.25, 63.17, 56.61, 55.59, 55.28, 53.78, 51.57, 48.88, 48.02, 46.93, 46.41, 42.5, 42.43, 37.89, 37.31, 37.03, 36.13, 35.2, 32.33, 32.2, 30.62, 29.91, 26.41, 24.48, 23.76, 23.42, 23.24, 22.02, 19.48, 18.96, 18.49, 17.65, 14.97. HRMS Calcd for $C_{39}H_{63}NO_6$ $[M+H]^+$: 640.45826; found: 640.45274. See Figures S122 and S123.

4-((2-(5-methoxy-1*H*-indol-3-yl)ethyl)amino)butyl-acanthosessiligenins (Compound **II-61**). Chloroform / methanol = 30:1 / 20:1, white solid, purity of 95.3%, yield 69%. mp 147-148 °C. ^{1}H NMR (300 MHz, $CDCl_3$): δ ppm 8.20 (*brs*, 1H), 7.23 (*d, J* = 6.9 Hz, 1H), 7.06 (*dd, J* = 8.3, 2.4 Hz, 2H), 6.85 (*dd, J* = 8.8, 2.4 Hz, 1H), 4.73 (*brs*, 1H), 4.61 (*brs*, 1H), 4.23 (*dd, J* = 10.3, 3.2 Hz, 1H), 4.15−4.21 (*m*, 2H), 3.91 (*dt, J* = 10.3, 5.0 Hz, 1H), 3.83 (*s*,3H), 3.63 (*s*, 3H), 3.46−3.42 (*t, J* = 6.5 Hz, 2H), 3.07 (*d, J* = 3.1 Hz, 1H), 2.97-2.90 (*m*, 1H), 2.30 (*d, J* = 4.1 Hz, 1H), 2.38−2.19 (*m*, 5H), 2.03− 1.83 (*m*, 7H), 1.67 (*s*, 3H), 1.65 (*d, J* = 4.1 Hz, 1H), 1.60 (*d, J* = 4.3 Hz, 1H), 1.58−1.07 (*m*, 14H), 1.23 (*s*, 3H), 1.14 (*s*, 3H), 1.09 (*s*, 3H), 0.98 (*s*, 3H), 0.90 (*s*, 3H). ^{13}C NMR (75 MHz, $CDCl_3$): δ ppm 176.2, 173.5, 154.07, 149.95, 131.7, 127.7, 123.39, 112.77, 112.27, 112.27, 110.33, 100.79, 86.51, 79.75, 68.33, 63.76, 56.59, 56.12, 55.64, 51.69, 49.3, 48.92, 48.02, 46.98, 46.44, 42.51, 42.45, 37.93, 37.32, 37.04, 36.22, 35.21, 32.39, 32.22, 30.64, 29.91, 29.03, 26.55, 25.44, 24.71, 24.52, 19.48, 18.96, 18.5, 17.63, 14.99. HRMS Calcd for $C_{46}H_{68}N_2O_7$ $[M+H]^+$: 759.49538; found: 759.49367. See Figures S124 and S125.

4-((2-(thiophen-2-yl)ethyl)amino)butyl-acanthosessiligenins (Compound **II-62**). Chloroform / methanol = 50:1/40:1, white solid, purity of 99.8%, yield 74%. mp 104-105 °C. ^{1}HNMR(300 MHz, $CDCl_3$): δ ppm 7.14 (*dd, J* = 5.1, 1.2 Hz, 1H), 6.93 (*dd, J* = 5.1, 3.4 Hz, 1H), 6.87 – 6.80 (*m*, 1H), 4.75 (*brs*, 1H), 4.62 (*brs*, 1H), 4.28 (*dd, J* = 10.3, 3.2Hz, 1H), 4.15−4.06 (*m*, 2H), 3.94 (*dt, J* = 10.3, 5.0 Hz, 1H), 3.67 (*s*, 3H), 3.46−3.42 (*t, J* = 6.5 Hz, 2H), 3.07 (*d, J* = 3.3 Hz, 1H), 3.03-2.93 (*m*, 1H), 2.31 (*d, J* = 4.1 Hz, 1H), 2.42−2.21 (*m*, 6H), 2.01−1.77 (*m*, 8H), 1.68 (*s*, 3H), 1.65 (*d, J* = 4.1 Hz, 1H), 1.61 (*d, J* = 5.3 Hz, 1H), 1.72−1.15 (*m*, 17H), 1.24 (*s*, 3H), 1.17 (*s*, 3H), 1.10 (*s*, 3H), 1.00(*s*, 3H),1.00 (*s*, 3H). ^{13}C NMR (75 MHz, $CDCl_3$): δ ppm 176.08, 173.31, 150.02, 127.02, 125.19, 123.76, 110.30, 86.46, 79.63, 77.58, 77.16, 76.74, 68.47, 63.97, 56.55, 55.62, 51.73, 51.02, 49.16, 48.95, 47.98, 46.98, 46.47, 42.52, 42.46, 38.04, 37.24, 37.03, 36.21, 35.26, 32.38, 30.63, 29.92, 26.68, 26.56, 24.51, 19.48, 18.96, 18.56, 17.62, 15.01. HRMS Calcd for $C_{41}H_{63}NO_6S$ $[M+H]^+$: 696.43033; found: 696.43250. See Figures S126 and S127.

4-((2-(1*H*-indol-3-yl)ethyl)amino)butyl-acanthosessiligenins (Compound **II-63**). Chloroform / methanol = 30:1 / 20:1, white solid, purity of 95.0%, yield 55%. mp 144-145 °C. ^{1}H NMR (300 MHz,$CDCl_3$): δ ppm 8.52(d, J = 8.9 Hz, 1H), 7.59 (*d, J* = 7.8 Hz,

1H), 7.35 (*d, J* = 8.0 Hz, 1H), 7.16 (*t, J* = 7.2 Hz, 1H), 7.09 (*d, J* = 15.6 Hz, 1H), 7.05 (*d, J* = 2.2 Hz, 1H), 4.77 (*brs*, 1H), 4.60(*brs*, 1H), 4.25 (*dd, J* = 11.5, 2.7 Hz, 1H), 4.03−3.99 (*d, J* = 7.1 Hz, 4H), 3.87 (*dt, J* = 10.7, 4.8 Hz, 1H), 3.64 (*s*, 3H), 3.08 (*d, J* = 2.5 Hz, 1H), 3.07−3.02 (*m*, 2H), 2.97-2.92 (*m*, 1H), 2.74−2.72 (*t, J* =6.7 Hz, 2H), 2.32 (*d, J* = 2.6 Hz, 1H), 2.36−2.21 (*m*, 2H), 1.98−1.81(*m*, 6H), 1.67 (*s*, 3H), 1.64 (*d, J* = 3.2 Hz, 1H), 1.56 (*d, J* = 3.5 Hz, 1H),1.61−1.15 (*m*, 14H), 1.24 (*s*, 3H), 1.14 (*s*, 3H), 1.09 (*s*, 3H), 0.98 (*s*,3H), 0.89 (*s*, 3H). ^{13}C NMR (75 MHz, $CDCl_3$): δ ppm 176.15, 173.46, 149.95, 136.52, 127.29, 122.52, 122.12, 119.38, 118.77, 112.71, 111.45, 110.32, 86.47, 79.71, 68.28, 63.8, 56.54, 55.59, 51.69, 49.74, 49.35, 48.89, 47.96, 46.95, 46.41, 42.47, 42.41, 37.95, 37.25, 37.03, 36.18, 35.18, 32.38, 32.18, 30.67, 29.89, 26.57, 25.64, 24.9, 24.51, 19.47, 18.95, 18.5, 17.61, 14.9. HRMS Calcd for $C_{45}H_{66}N_2O_6$ $[M+H]^+$: 729.48481; found: 729.48257. See Figures S128 and S1129.

5-(4-formyl-1*H*-imidazol-1-yl)pentyl-acanthosessiligenins (Compound **II-64**). Chloroform / methanol = 60:1 / 50:1, white solid, purity of 95.5%, yield 60%. mp 127-128 °C. ^{1}H NMR (300 MHz, $CDCl_3$): δ ppm 9.85 (*brs*, 1H), 7.63 (*d, J* = 1.3 Hz, 1H), 7.54 (*d, J* = 1.3 Hz, 1H), 4.72 (*brs*, 1H), 4.60 (*brs*, 1H), 4.27 (*dd, J* = 11.3, 2.9 Hz, 1H), 4.10−3.97 (*dt, J* = 11.0, 5.3 Hz, 2H), 3.94 (*dt, J* = 17.9, 6.4 Hz, 1H), 3.66 (*s*, 3H), 3.44−3.40 (*t, J* = 6.6 Hz, 2H), 3.06 (*d, J* = 2.9 Hz, 1H), 2.96-2.86 (*m*, 1H), 2.30 (*d, J* = 2.6 Hz, 1H), 2.37−2.16 (*m*, 2H), 1.95−1.76 (*m*, 3H), 1.66 (*s*, 3H), 1.67−1.62 (*m*, 3H), 1.62 (*d, J* = 2.2 Hz, 1H), 1.58 (*d, J* = 4.3 Hz, 1H), 1.51−1.05 (*m*, 15H), 1.23 (*s*, 3H), 1.17 (*s*, 3H), 1.09 (*s*, 3H), 0.99 (*s*, 3H), 0.90 (*s*, 3H). ^{13}C NMR (75 MHz, $CDCl_3$): δ ppm 186.35, 176.13, 173.36, 149.84, 142.76, 138.74, 124.11, 110.34, 86.48, 79.61, 68.22, 63.49, 56.58, 55.6, 51.59, 48.87, 47.95, 47.69, 47.06, 46.43, 42.46, 42.44, 37.98, 37.33, 37.01, 36.12, 35.22, 32.33, 32.21, 30.58, 29.85, 29.77, 28.32, 24.48, 23.28, 19.37, 18.93, 18.51, 17.58, 14.99. HRMS Calcd for $C_{40}H_{60}N_2O_7$ $[M+H]^+$: 679.43278; found: 679.43456. See Figures S130 and S131.

5-(2-propyl-1*H*-imidazol-1-yl)pentyl-acanthosessiligenins (Compound **II-65**). Chloroform / methanol = 50:1 / 40:1, white solid, purity of 95.4%, yield 55%. mp 144-145 °C. ^{1}H NMR (300 MHz,$CDCl_3$): δ ppm 6.92 (*d, J* = 4.8 Hz, 1H), 6.79 (*d, J* = 5.6 Hz, 1H), 4.76 (*brs*, 1H), 4.62 (*brs*, 1H), 4.25 (*dd, J* = 11.3, 2.9Hz, 1H), 4.11−4.05 (*dt, J* = 11.0, 5.3 Hz, 2H), 3.95 (*dt, J* = 17.9, 6.4 Hz, 1H), 3.67 (*s*, 3H), 3.44−3.40 (*t, J* = 6.6 Hz, 2H), 3.07 (*d, J* = 2.9 Hz, 1H), 2.97-2.93 (*m*, 1H), 2.31 (*d, J* = 2.6 Hz, 1H), 2.30−2.23

(*m*, 4H), 2.05−1.84 (*m*, 3H), 1.69 (*s*, 3H), 1.67−1.62 (*m*, 4H), 1.65 (*d, J* =2.2 Hz, 1H), 1.62 (*d, J* = 4.3 Hz, 1H), 1.55−1.13 (*m*, 16H), 1.23 (*s*, 3H), 1.16 (*s*, 3H), 1.09 (*s*, 3H), 0.98 (*s*, 3H),0.94 (*s*, 3H),0.88 (*s*, 3H).^{13}C NMR (75 MHz, $CDCl_3$): δ ppm 176.15, 173.35, 149.8, 148.74, 148.09, 118.73, 110.39, 86.47, 79.6, 67.84, 63.67, 56.6, 55.55, 51.56, 48.87, 47.91, 47.06, 46.43, 45.64, 42.63, 42.43, 38, 37.37, 37.06, 36.19, 35.2, 32.3, 32.25, 30.69, 30.41, 29.83, 28.81, 28.58, 24.46, 23.5, 21.96, 19.4, 18.87, 18.5, 17.53, 14.97, 13.85. HRMS Calcd for $C_{42}H_{66}N_2O_6$ $[M+H]^+$: 693.48481; found: 693.48357. See Figures S132 and S133.

5-(2,4-dimethyl-1*H*-imidazol-1-yl)pentyl-acanthosessiligenins (Compound **II-66**). Chloroform / methanol = 50:1 / 40:1, white solid, purity of 96.4%, yield 81%. mp 133-134 °C. ^{1}H NMR (300 MHz,$CDCl_3$): δ ppm 6.66 (*d, J* = 5.6 Hz, 1H), 4.70 (*brs*, 1H), 4.62 (*brs*, 1H), 4.28 (*dd, J* = 11.3, 2.8Hz, 1H), 4.11−4.05 (*dt, J* = 11.0, 5.3 Hz, 2H), 3.95 (*dt, J* = 17.9, 6.4 Hz, 1H), 3.67 (*s*, 3H), 3.44−3.40 (*t, J* = 6.6 Hz, 2H), 3.07 (*d, J* = 2.9 Hz, 1H), 2.97-2.93 (*m*, 1H), 2.31 (*d, J* = 2.6 Hz, 1H), 2.30−2.23 (*m*, 9H), 2.05−1.84 (*m*, 3H), 1.69 (*s*, 3H), 1.67−1.62 (*m*, 3H), 1.65 (*d, J* =2.2 Hz, 1H), 1.63 (*d*, J = 4.3 Hz, 1H), 1.55−1.13 (*m*, 14H), 1.23 (*s*, 3H), 1.17 (*s*, 3H), 1.09 (*s*, 3H), 0.99 (*s*, 3H), 0.89 (*s*, 3H).^{13}C NMR (75 MHz, $CDCl_3$): δ ppm 176.29, 173.36, 149.81, 143.61, 135.9, 115.29, 110.2, 86.46, 79.57, 67.94, 63.78, 56.68, 55.58, 51.63, 48.84, 47.88, 47.12, 46.46, 45.8, 45.75, 42.46, 38.07, 37.48, 37.09, 36.24, 35.23, 32.34, 32.19, 30.77, 30.62, 29.84, 28.67, 24.49, 23.59, 19.54, 18.93, 18.55, 17.56, 15.01, 13.41, 13.01. HRMS Calcd for $C_{41}H_{64}N_2O_6$ $[M+H]^+$: 679.46916; found: 679.46756. See Figures S134 and S135.

5-(5-bromo-1*H*-imidazol-1-yl)pentyl-acanthosessiligenins (Compound **II-67**). Chloroform / methanol = 55:1 / 45:1, white solid, purity of 98.9%, yield 74%. mp 137-138 °C. ^{1}H NMR (300 MHz, $CDCl_3$): δ ppm 7.47 (*d, J* = 75.4 Hz, 1H), 6.97 (*d, J* = 49.0 Hz, 1H), 4.71 (*brs*, 1H), 4.61 (*brs*, 1H), 4.26 (*dd, J* = 11.3, 2.9 Hz, 1H), 4.11−4.05 (*dt, J* = 11.0, 5.3 Hz, 2H), 3.99 (*dt, J* = 17.9, 6.4 Hz, 1H), 3.64 (*s*, 3H), 3.18−3.05 (*t, J* = 6.6 Hz, 2H), 3.01 (*d, J* = 2.9 Hz, 1H), 2.97-2.92 (*m*, 1H), 2.36 (*d, J* = 2.6 Hz, 1H), 2.36−2.30 (*m*, 2H), 2.05−1.83 (*m*, 3H), 1.67 (*s*, 3H), 1.67−1.62 (*m*, 3H), 1.65 (*d, J* = 2.2 Hz, 1H), 1.62 (*d, J* = 4.3 Hz, 1H), 1.50−1.13 (*m*, 15H), 1.24 (*s*, 3H), 1.17 (*s*, 3H), 1.11 (*s*, 3H), 1.00 (*s*, 3H), 0.90 (*s*, 3H). ^{13}C NMR (75 MHz, $CDCl_3$): δ ppm 176.16, 173.63, 149.85, 136.65, 135.47, 110.35, 104.87, 86.54, 80.15, 76.74, 68.08, 63.56, 56.57, 55.64, 51.68, 48.87, 47.99, 47.76, 47.05, 46.40, 42.46, 37.67, 37.31, 37.00, 36.12, 35.14, 32.40, 32.19,

30.60, 30.47, 29.83, 28.31, 24.55, 23.27, 19.39, 18.97, 18.45, 17.56, 15.00. HRMS Calcd for $C_{39}H_{59}BrN_2O_6$ $[M+H]^+$: 729.34837; found: 729.34247. See Figures S136 and S137.

5-(2-ethyl-4-methyl-1*H*-imidazol-1-yl)pentyl-acanthosessiligenins (Compound **II-68**). Chloroform / methanol = 40:1 / 30:1, white solid, purity of 98.9%, yield 76%. mp 138-139 °C. ^{1}H NMR (300 MHz, $CDCl_3$): δ ppm 6.50 (*brs*, 1H), 4.72 (*brs*, 1H), 4.61 (*brs*, 1H), 4.28 (*dd, J* = 11.3, 2.9 Hz, 1H), 4.11−4.05 (*dt, J* = 11.0, 5.3 Hz, 2H), 3.83 (*dt, J* = 17.9, 6.4 Hz, 1H), 3.66 (*s*, 3H), 3.44−3.40 (*t, J* = 6.6 Hz, 2H), 3.08 (*d, J* = 2.9 Hz, 1H), 2.99-2.93 (*m*, 1H), 2.30 (*d, J* = 2.6 Hz, 1H), 2.37−2.12 (*m*, 2H), 1.;91−1.68 (*m*, 8H), 1.67 (*s*, 3H), 1.67−1.62 (*m*, 6H), 1.63 (*d, J* = 2.2 Hz, 1H), 1.59 (*d, J* = 4.3 Hz, 1H), 1.54−1.05 (*m*, 15H), 1.23 (*s*, 3H), 1.16 (*s*, 3H), 1.09 (*s*, 3H), 0.99 (*s*, 3H), 0.89 (*s*, 3H). ^{13}C NMR (75 MHz, $CDCl_3$): δ ppm 176.18, 173.33, 149.83, 148.5, 136.06, 115.14, 110.18, 86.45, 79.56, 68.04, 63.7, 56.63, 55.57, 51.62, 48.86, 47.91, 47.03, 46.44, 45.41, 42.54, 42.45, 38.04, 37.37, 37.03, 36.22, 35.23, 32.33, 32.26, 30.73, 29.85, 29.77, 28.62, 24.47, 23.55, 20.18, 19.54, 18.91, 18.53, 17.56, 14.99, 13.56, 12.67. HRMS Calcd for $C_{42}H_{66}N_2O_6$ $[M+H]^+$: 693.48481; found: 693.48375. See Figures S138 and S139.

5-(pyrrolidin-1-yl)pentyl-acanthosessiligenins (Compound **II-69**). Chloroform / methanol = 50:1 / 40:1, white solid, purity of 987.3%, yield 61%. mp 122-123 °C. ^{1}H NMR (300 MHz,$CDCl_3$): δ ppm 4.73 (*brs*, 1H), 4.62 (*brs*, 1H), 4.28 (*dd, J* = 11.3, 2.9Hz, 1H), 4.11−4.05 (*dt, J* = 11.0, 5.3 Hz, 2H), 3.95 (*dt, J* = 17.9, 6.4 Hz, 1H), 3.66 (*s*, 3H), 3.44−3.40 (*t, J* = 6.6 Hz, 2H), 3.09 (*d, J* = 2.8 Hz, 1H), 2.97-2.90 (*m*, 1H), 2.31 (*d, J* = 2.6 Hz, 1H), 2.30−2.23 (*m*, 2H), 2.05−1.84 (*m*, 8H), 1.68 (*s*, 3H), 1.67−1.62 (*m*, 7H), 1.64 (*d, J* =2.2 Hz, 1H), 1.61 (*d, J* = 4.3 Hz, 1H), 1.55−1.13 (*m*, 14H), 1.24 (*s*, 3H), 1.17 (*s*, 3H), 1.09 (*s*, 3H), 1.00 (*s*, 3H), 0.92 (*s*, 3H).^{13}C NMR (75 MHz, CDCl3): δ ppm 179.14, 173.19, 149.42, 110.39, 86.28, 79.48, 68.12, 59.98, 56.46, 55.44, 51.51, 51.41, 48.74, 47.85, 46.68, 46.25, 44.44, 42.32, 42.28, 37.79, 37.01, 36.81, 35.92, 35.06, 32.2, 31.95, 30.35, 30.35, 30.17, 29.94, 29.81, 29.51, 24.32, 19.37, 19.28, 19.19, 18.8, 18.35, 17.47,14.81. HRMS Calcd for $C_{40}H_{65}NO_6$ $[M+H]^+$: 654.47391; found: 654.47537. See Figures S140 and S141.

5-((2-(5-methoxy-1*H*-indol-3-yl)ethyl)amino)pentyl-acanthosessiligenins (Compound **II-70**). Chloroform / methanol = 30:1 / 20:1, white solid, purity of 99.8%, yield 72%.

mp 114-115 °C. ^{1}H NMR (300 MHz, $CDCl_3$): δ ppm 8.48 – 8.22 (*m*, 1H), 7.23 (*d, J* = 2.5 Hz, 1H), 7.05 (*dt, J* = 5.8, 2.5 Hz, 1H), 6.84 (*dd, J* = 8.8, 2.4 Hz, 1H) ,4.76 (*brs*, 1H), 4.60 (*brs,* 1H), 4.27 (*dd, J* = 11.3, 2.9 Hz, 1H), 4.11−4.02 (*dt, J* = 11.0, 5.3 Hz, 2H), 3.80 (*dt, J* = 17.9, 6.4 Hz, 1H), 3.84(*s*, 3H), 3.65 (*s*, 3H), 3.44−3.40 (*t, J* = 6.6 Hz, 2H), 3.04-2.93 (*m*, 1H), 2.75 (*dt, J* = 11.3 Hz, 1H), 2.31 (*d, J* = 2.6 Hz, 1H), 2.40−2.20 (*m*, 7H), 2.05−1.84 (*m*, 3H), 1.64 (*s*, 3H), 1.67−1.62 (*m*, 3H), 1.65 (*d, J* = 2.2 Hz, 1H), 1.62 (*d, J* = 4.3 Hz, 1H), 1.55−1.33 (*m*, 16H), 1.23 (*s*, 3H), 1.14 (*s*, 3H), 1.09 (*s*, 3H), 0.98 (*s*, 3H), 0.90 (*s*, 3H). ^{13}C NMR (75 MHz, $CDCl_3$): δ ppm 176.23, 173.5, 153.97, 149.98, 131.73, 127.68, 123.43, 112.35, 112.23, 112.16, 110.26, 100.75, 86.49, 79.75, 68.2, 63.95, 56.08, 55.61, 51.64, 49.35, 49.21, 48.91, 48.02, 47.01, 46.41, 42.49, 42.42, 37.92, 37.32, 37.02, 36.18, 35.18, 32.34, 32.25, 30.63, 29.86, 29.30, 28.56, 25.22, 24.77, 24.47, 23.86, 19.43, 18.91, 18.48, 17.59, 14.97. HRMS Calcd for $C_{47}H_{70}N_2O_7$ $[M+H]^+$: 787.53401; found: 787.53247. See Figures S142 and S143.

5-((2-(thiophen-2-yl)ethyl)amino)pentyl-acanthosessiligenins (Compound **II-71**). Chloroform / methanol = 50:1 / 40:1, white solid, purity of 98.5%, yield 66%. mp 144-145 °C. ^{1}H NMR (300 MHz,$CDCl_3$): δ ppm 7.13 (dd, J = 5.1, 1.2 Hz, 1H), 6.91 (*dd, J* = 5.1, 3.4 Hz, 1H), 6.82 (*d, J* = 3.4 Hz, 1H), 4.73 (*brs*, 1H), 4.60 (*brs*, 1H), 4.25 (*dd, J* = 11.3, 2.9Hz, 1H), 4.11−4.05 (*dt, J* = 11.0, 5.3 Hz, 2H), 3.99 (*dt, J* = 17.9, 6.4 Hz,1H), 3.65 (*s*, 3H), 3.44−3.40 (*t, J* = 6.6 Hz, 2H), 3.07-2.93 (*m*, 1H), 2.65 (*dt, J* = 11.3 Hz, 1H), 2.31 (*d, J* = 2.6 Hz, 1H), 2.31−2.19 (*m*, 2H), 2.05−1.84 (*m*, 7H), 1.66 (*s*, 3H), 1.67−1.62 (*m*, 3H), 1.63 (*d, J* =2.2 Hz, 1H), 1.62 (*d, J* = 4.3 Hz, 1H), 1.63−1.06 (*m*, 16H), 1.22 (*s*, 3H), 1.16 (*s*, 3H), 1.08 (*s*, 3H), 0.98(*s*, 3H), 0.91 (*s*, 3H).^{13}C NMR (75 MHz, $CDCl_3$): δ ppm 176.12, 173.32, 150.03, 142.54, 127.02, 125.21, 123.76, 110.29, 86.47, 79.63, 68.42, 64, 56.56, 55.63, 51.68, 51.05, 49.47, 48.95, 47.99, 47.01, 46.46, 42.53, 42.46, 38.02, 37.29, 37.04, 36.23, 35.26, 32.38, 32.25, 30.64, 30.17, 29.91, 29.43, 28.73, 24.51, 23.96, 19.47,18.96, 18.55, 17.64, 15.01. HRMS Calcd for $C_{42}H_{65}NO_6S$ $[M+H]^+$: 710.44598; found: 710.44425. See Figures S144 and S145.

5-((2-(1*H*-indol-3-yl)ethyl)amino)pentyl-acanthosessiligenins (Compound **II-72**). Chloroform / methanol = 30:1 / 20:1, white solid, purity of 99.3%, yield 59%. mp 122-123 °C. ^{1}HNMR(300 MHz, $CDCl_3$): δ ppm 8.12 (*d, J* = 8.1, 1H), 7.62 (*d, J* = 7.9 Hz, 1H), 7.37 (*d, J* = 8.2 Hz, 1H), 7.20 (*t, J* = 7.0 Hz, 1H), 7.11 (*t, J* = 6.9Hz, 1H), 7.05 (*d, J* = 2.4 Hz, 1H), 4.74 (*brs*, 1H), 4.62 (*brs*, 1H), 4.25(*dd, J* = 11.4, 2.8 Hz, 1H), 4.04−4.02

(*t, J* = 6.5 Hz, 4H), 3.88 (*dt, J* =10.8, 4.9 Hz, 1H), 3.67 (*s*, 3H), 3.03 (*d, J* = 2.9 Hz, 1H), 2.99−2.95 (*dt, J* = 8.6 Hz, 2H), 2.98-2.90 (*m*, 1H), 2.67−2.62 (*t, J* = 14.4 Hz, 2H), 2.31 (*d, J* = 2.6 Hz, 1H), 2.36−2.23 (*m*, 2H), 2.00−1.83 (*m*, 6H),1.68 (*s*, 3H), 1.65 (*d, J* = 2.1 Hz, 1H), 1.61 (*d, J* = 2.6 Hz, 1H), 1.60−1.12 (*m*, 16H), 1.24 (*s*, 3H), 1.16 (*s*, 3H), 1.10 (*s*, 3H), 0.99 (*s*, 3H),0.92 (*s*, 3H). ^{13}C NMR (75 MHz, $CDCl_3$): δ ppm 176.17, 173.35, 150.05, 136.57, 127.54, 122.2, 122.11, 119.35, 118.94, 113.88, 111.35, 110.31, 86.5, 79.67, 68.39, 64.08, 56.58, 55.64, 51.69, 50.02, 49.84, 48.95, 48.01, 47.06, 46.47, 42.55, 42.47, 38.04, 37.34, 37.06, 36.27, 35.27, 32.39, 32.27, 30.66, 29.92, 29.71, 28.8, 25.81, 24.52, 24.08, 19.47, 18.97, 18.55, 17.65, 15.02. HRMS Calcd for $C_{46}H_{68}N_2O_6$ $[M+H]^+$: 743.50046; found: 743.50257. See Figures S146 and S147.

6-(4-formyl-1*H*-imidazol-1-yl)hexyl-acanthosessiligenins (Compound **II-73**). Chloroform / methanol = 60:1 / 50:1, white solid, purity of 95.6%, yield 74%. mp 123-124°C. ^{1}H NMR (300 MHz, $CDCl_3$): δ ppm 9.86 (*brs*, 1H), 7.83 (*d, J* = 12.0 Hz, 1H), 7.59 (*dd, J* = 20.5, 1.3 Hz, 2H),4.73 (*brs*, 1H), 4.61 (*brs*, 1H), 4.27 (*dd, J* = 11.3, 2.9 Hz, 1H), 4.10−4.02 (*dt, J* = 10.7, 5.7 Hz, 2H), 3.71 (*dt, J* = 9.9 Hz, 1H), 3.66 (*s*, 3H), 3.38−3.18 (*t, J* = 6.8 Hz, 2H), 3.06 (*d, J* = 2.8 Hz, 1H), 2.91 (*dt, J* = 14.2, 3.5 Hz, 1H), 2.31 (*d, J* = 2.6 Hz, 1H), 2.39−2.24 (*m*, 2H), 2.02−1.75 (*m*, 5H), 1.67 (*s*, 3H), 1.65 (*d, J* = 4.9 Hz, 1H), 1.61 (*d, J* = 5.6 Hz, 1H), 1.65−1.13 (*m*, 17H), 1.24 (*s*, 3H), 1.17 (*s*, 3H), 1.10 (*s*, 3H), 1.00 (*s*, 3H), 0.91 (*s*, 3H). ^{13}C NMR (75 MHz, $CDCl_3$): δ ppm 186.27, 176.09, 173.39, 149.96, 141.51, 138.84, 124.4, 110.35, 86.4, 79.71, 68.35, 63.77, 56.54, 55.62, 51.72, 48.92, 48.01, 47.73, 47, 46.44, 42.49, 42.44, 37.95, 37.25, 37.01, 36.15, 35.23, 32.35, 32.2, 30.85, 30.59, 29.88, 28.61, 26.21, 25.67, 24.49, 19.32, 18.93, 18.51, 17.59, 14.98. HRMS Calcd for $C_{41}H_{62}N_2O_7$ $[M+H]^+$: 693.44843; found: 693.44357. See Figures S148 and S149.

6-(2-propyl-1*H*-imidazol-1-yl)hexyl-acanthosessiligenins (Compound **II-74**). Chloroform / methanol = 50:1 / 40:1, white solid, purity of 96.2%, yield 58%. mp 133-134 °C. ^{1}H NMR (300 MHz,$CDCl_3$): δ ppm 7.61 (*d, J* = 6.6 Hz, 1H), 7.13 (*d, J* = 7.5 Hz, 1H), 5.66(*d, J* = 8.2 Hz, 1H), 4.75 (*brs*, 1H), 4.62 (*brs*, 1H), 4.25 (*dd, J* = 11.3, 2.9Hz, 1H), 4.10−4.04 (*dt, J* = 10.7, 5.7 Hz, 2H), 3.95 (*dt, J* = 9.9 Hz, 1H), 3.69 (*s*, 3H), 3.43−3.39 (*t, J* = 6.8 Hz, 2H), 3.07 (*d, J* = 2.8 Hz, 1H), 2.62 (*dt, J* = 14.2, 3.5 Hz, 1H), 2.31 (*d, J* = 2.6 Hz, 1H), 2.39−2.24 (*m*, 4H), 2.03−1.82 (*m*, 6H), 1.69 (*s*, 3H), 1.67 (*d, J* = 4.9 Hz, 1H), 1.61 (*d, J* = 5.6 Hz, 1H), 1.65−1.25 (*m*, 18H), 1.57 (*s*, 3H)1.50 (*s*, 3H),

1.43 (*s*, 3H), 1.33(*s*, 3H), 1.27 (*s*, 3H), 1.25 (*s*, 3H).^{13}C NMR (75 MHz, $CDCl_3$): δ ppm 176.11, 173.36, 149.94, 127.01, 121.24, 118.82, 110.28, 86.47, 79.65, 68.1, 63.81, 56.53, 55.58, 51.62, 48.92, 47.99, 47.01, 46.45, 45.73, 42.46, 42.43, 38.01, 37.25, 37.01, 36.18, 35.22, 32.34, 32.21, 30.6, 30.46, 29.87, 28.79, 28.66, 26.39, 25.83, 24.48, 21.54, 19.4, 18.9, 18.52, 17.57, 14.97, 13.88. HRMS Calcd for $C_{43}H_{68}N_2O_6$ $[M+H]^+$: 707.50046; found: 707.50358. See Figures S150 and S151.

6-(2,4-dimethyl-1*H*-imidazol-1-yl)hexyl-acanthosessiligenins (Compound **II-75**). Chloroform / methanol = 50:1 / 40:1, white solid, purity of 96.2%, yield 66%. mp 111-112 °C. ^{1}H NMR (300 MHz,$CDCl_3$): δ ppm6.68 (*d, J* = 5.7 Hz, 1H) 4.73 (brs, 1H), 4.62 (*brs*, 1H), 4.29 (*dd, J* = 11.3, 2.9Hz, 1H), 4.10−4.04 (*dt, J* = 10.7, 5.7 Hz, 2H), 3.95 (*dt, J* = 9.9 Hz, 1H),3.69 (*s*, 3H), 3.43−3.39 (*t, J* = 6.8 Hz, 2H), 3.08 (*d, J* = 2.8 Hz, 1H), 2.98 (*dt, J* = 14.2, 3.5 Hz, 1H), 2.31 (*d, J* = 2.6 Hz, 1H), 2.39−2.24 (*m*, 5H), 2.03−1.82 (*m*, 7H), 1.67 (*s*, 3H), 1.67 (*d, J* = 4.9 Hz, 1H), 1.61 (*d, J* = 5.6 Hz, 1H), 1.65−1.13 (*m*, 19H), 1.24 (*s*, 3H), 1.17 (*s*, 3H), 1.09(*s*, 3H), 0.91 (*s*, 3H), 0.91 (*s*, 3H).^{13}C NMR (75 MHz, $CDCl_3$): δ ppm 176.09, 173.32, 149.94, 143.56, 135.82, 115.36, 110.25, 86.44, 79.57, 68.14, 63.84, 56.53, 55.59, 51.63, 48.92, 48, 47, 46.45, 45.8, 42.47, 42.43, 38.01, 37.25, 37.01, 36.2, 35.23, 32.33, 32.21, 30.81, 30.61, 29.88, 28.66, 26.33, 25.83, 24.48, 19.42, 18.91, 18.53, 17.58, 14.98, 13.49, 12.97. HRMS Calcd for $C_{42}H_{66}N_2O_6$ $[M+H]^+$: 693.48481; found: 693.48032. See Figures S152 and S153.

6-(5-bromo-1*H*-imidazol-1-yl)hexyl-acanthosessiligenins (Compound **II-76**). Chloroform / methanol = 50:1 / 40:1, white solid, purity of 99.7%, yield 52%. mp 106-107 °C. ^{1}H NMR (300 MHz, $CDCl_3$): δ ppm 7.47 (*d, J* = 73.7 Hz, 1H), 6.97 (*d, J* = 49.7 Hz, 1H), 4.73 (*brs*, 1H), 4.60 (*brs*, 1H), 4.25 (*dd, J* = 11.3, 2.9 Hz, 1H), 4.10−4.04 (*dt, J* = 10.7, 5.7 Hz, 2H), 3.98 (*dt, J* = 9.9 Hz, 1H), 3.63 (*s*, 3H), 3.43−3.39 (*t, J* = 6.8 Hz, 2H), 3.04 (*d, J* = 2.8 Hz, 1H), 2.91 (*dt, J* = 14.2, 3.5 Hz, 1H), 2.30 (*d, J* = 2.6 Hz, 1H), 2.36−2.30 (*m*, 2H), 2.03−1.72 (*m*, 5H), 1.67 (*s*, 3H), 1.61 (*d, J* = 4.9 Hz, 1H), 1.60 (*d, J* = 5.6 Hz, 1H), 1.54−1.05 (*m*, 18H), 1.24 (*s*, 3H), 1.16 (*s*, 3H), 1.11 (*s*, 3H), 1.00 (*s*, 3H), 0.92 (*s*, 3H). ^{13}C NMR (75 MHz, $CDCl_3$): δ ppm 176.12, 173.58, 149.96, 136.69, 135.18, 110.29, 101.86, 86.55, 80.10, 68.18, 63.81, 57.39, 55.64, 51.64, 48.90, 48.06, 47.78, 46.99, 46.41, 42.49, 42.44, 37.70, 37.24, 37.00, 36.13, 35.16, 32.40, 32.18, 30.79, 30.59, 29.86, 28.60, 26.18, 25.67, 24.55, 19.41, 18.97, 18.47, 17.57, 15.00. HRMS

Calcd for $C_{40}H_{61}BrN_2O_6$ $[M+H]^+$: 743.36402; found: 743.36634. See Figures S154 and S155.

6-(2-ethyl-4-methyl-1*H*-imidazol-1-yl)hexyl-acanthosessiligenins (Compound **II-77**). Chloroform / methanol = 55:1 / 45:1, white solid, purity of 95.3%, yield 73%. mp 143-144 °C. ^1H NMR (300 MHz, $CDCl_3$): δ ppm 6.48 (*q, J* = 1.0 Hz, 1H), 4.75 (*brs*, 1H), 4.60 (*brs*, 1H), 4.27 (*dd, J* = 11.3, 2.9 Hz, 1H), 4.10−4.04 (*dt, J* = 10.7, 5.7 Hz, 2H), 3.71 (*dt, J* = 9.9 Hz, 1H), 3.66 (*s*, 3H), 3.43−3.39 (*t, J* = 6.8 Hz, 2H), 3.06 (*d, J* = 2.8 Hz, 1H), 2.97 (*dt, J* = 14.2, 3.5 Hz, 1H), 2.30 (*d, J* = 2.6 Hz, 1H), 2.37−2.13 (*m*, 8H), 2.03−1.82 (*m*, 5H), 1.67 (*s*, 3H), 1.64 (*d, J* = 4.9 Hz, 1H), 1.60 (*d, J* = 5.6 Hz, 1H), 1.55−1.04 (*m*, 20H), 1.23 (*s*, 3H), 1.13 (*s*, 3H), 1.09 (*s*, 3H), 0.99 (*s*, 3H), 0.91 (*s*, 3H). ^{13}C NMR (75 MHz, $CDCl_3$): δ ppm 176.07, 173.33, 149.93, 148.44, 135.9, 115.14, 110.23, 86.44, 79.58, 68.18, 63.84, 56.53, 55.58, 51.62, 48.92, 47.98, 46.98, 46.43, 45.44, 42.47, 42.43, 38, 37.23, 37, 36.18, 35.23, 32.32, 32.19, 30.75, 30.6, 29.87, 28.66, 26.4, 25.83, 24.46, 20.14, 19.42, 18.9, 18.52, 17.57, 14.97, 13.55, 12.66. HRMS Calcd for $C_{43}H_{68}N_2O_6$ $[M+H]^+$: 707.50046; found: 707.50025. See Figures S156 and S157.

6-(pyrrolidin-1-yl)hexyl-acanthosessiligenins (Compound **II-78**). Chloroform / methanol = 50:1 / 40:1, white solid, purity of 96.7%, yield 72%. mp 120-121 °C. ^1H NMR (300 MHz,$CDCl_3$): δ ppm 4.73 (*brs*, 1H), 4.61 (*brs*, 1H), 4.28 (*dd, J* = 11.3, 2.9Hz, 1H), 4.10−4.04 (*dt, J* = 10.7, 5.7 Hz, 2H), 4.05 (*dt, J* = 9.9 Hz, 1H), 3.64 (*s*, 3H), 3.43−3.39 (*t, J* = 6.8 Hz, 2H), 3.07 (*d, J* = 2.8 Hz, 1H),2.98 (*dt, J* = 14.2, 3.5 Hz, 1H), 2.31 (*d, J* = 2.6 Hz, 1H), 2.39−2.24 (*m*, 6H), 2.03−1.82 (*m*, 9H), 1.69 (s, 3H), 1.64 (*d, J* = 4.9 Hz, 1H), 1.59 (*d, J* = 5.6 Hz, 1H), 1.65−1.13 (*m*, 20H), 1.23 (s, 3H), 1.17 (*s*, 3H), 1.09(*s*, 3H), 0.99 (*s*, 3H), 0.91 (*s*, 3H). ^{13}C NMR (75 MHz, $CDCl_3$): δ ppm 176.29, 173.38, 149.99, 110.3, 86.43, 79.62, 68.12, 63.89, 56.59, 55.76, 55.57, 53.83, 51.6, 48.93, 48, 47.2, 47.12, 46.42, 42.5, 42.43, 37.94, 37.42, 37.05, 36.13, 35.19, 32.31, 30.66, 29.85, 29.76, 29.10, 28.6, 26.74, 26.43, 25.95, 25.10, 24.46, 23.38, 19.4, 18.93, 18.49, 17.6, 14.98. HRMS Calcd for $C_{42}H_{69}NO_6$ $[M+H]^+$: 682.51253; found: 682.51357. See Figures S158 and S159.

6-((2-(5-methoxy-1*H*-indol-3-yl)ethyl)amino)hexyl-acanthosessiligenins (Compound **II-79**). Chloroform / methanol = 30:1 / 20:1, white solid, purity of 95.4%, yield 75%. mp 136-137 °C. ^1H NMR (300 MHz, $CDCl_3$): δ ppm 8.55 – 8.35 (*m*, 1H), 7.23 (*brs*,

1H), 7.06 (*dd, J* = 6.7, 2.4 Hz, 1H), 6.83 (*dd, J* = 8.8, 2.4 Hz, 1H), 4.75 (*brs*, 1H), 4.61 (*brs*, 1H), 4.31 (*dd, J* = 11.3, 2.9 Hz, 1H), 4.10−4.04 (*dt, J* = 10.7, 5.7 Hz, 2H), 3.89 (*dt, J* = 9.9 Hz, 1H), 3.84 (s, 3H), 3.77−3.61 (t, J = 6.8 Hz, 2H), 3.10 (d, J = 2.8 Hz, 1H), 3.02 (*dt, J* = 14.2, 3.5 Hz, 1H), 2.31 (*d, J* = 2.6 Hz, 1H), 2.39−2.14 (*m*, 5H), 2.03−1.91 (*m*, 9H), 1.68 (*s*, 3H), 1.64 (*d, J* = 4.9 Hz, 1H), 1.60 (*d, J* = 5.6 Hz, 1H), 1.64−1.05 (*m*, 20H), 1.23 (*s*, 3H), 1.14 (*s*, 3H), 1.09 (*s*, 3H), 1.00 (*s*, 3H), 0.90 (*s*, 3H). ^{13}C NMR (75 MHz, $CDCl_3$): δ ppm 176.51, 173.58, 153.97, 149.98, 131.74, 127.64, 123.77, 112.18, 112.14, 111.95, 110.32, 100.72, 86.55, 79.74, 67.97, 64.17, 56.11, 55.64, 51.63, 49.27, 49.12, 48.95, 48.09, 47.3, 46.44, 42.56, 42.46, 37.94, 37.65, 37.12, 36.34, 35.2, 32.46, 32.38, 30.75, 29.88, 28.88, 28.73, 26.86, 26.39, 24.65, 24.5, 22.37, 19.43, 19.01, 18.52, 17.66, 15.05. HRMS Calcd for $C_{48}H_{72}N_2O_7$ $[M+H]^+$: 787.52668; found: 787.52735. See Figures S160 and S161.

5-((2-(thiophen-2-yl)ethyl)amino)pentyl-acanthosessiligenins (Compound **II-80**). Chloroform / methanol = 50:1 / 40:1, white solid, purity of 97.3%, yield 70%. mp 135-136 °C. ^{1}H NMR (300 MHz,$CDCl_3$): δ ppm 7.14 (d, J = 5.1, 1H), 6.93 (*dd, J* = 5.1, 3.4 Hz, 1H),6.88 (*d, J* = 6.2, 1H), 4.74 (*brs*, 1H), 4.62 (*brs*, 1H), 4.26 (*dd, J* = 11.3, 2.8Hz, 1H), 4.10−4.04 (*dt, J* = 10.7, 5.7 Hz, 2H), 3.89 (*dt, J* = 9.9 Hz, 1H),3.66 (*s*, 3H), 3.43−3.39 (*t, J* = 6.8 Hz, 2H), 3.07 (*d, J* = 2.8 Hz, 1H),2.98 (*dt, J* = 14.2, 3.5 Hz, 1H), 2.31 (*d, J* = 2.6 Hz, 1H), 2.39−2.24 (*m*, 3H), 2.03−1.82 (*m*, 7H), 1.69 (*s*, 3H), 1.68 (*d, J* = 4.9 Hz, 1H), 1.64 (*d, J* = 5.6 Hz, 1H), 1.65−1.13 (*m*, 20H), 1.24 (*s*, 3H), 1.17 (*s*, 3H), 1.10(*s*, 3H), 1.00 (*s*, 3H), 0.93 (*s*, 3H). ^{13}C NMR (75 MHz, $CDCl_3$): δ ppm 176.31, 173.3, 150.03, 142.33, 127.03, 125.27, 123.74, 110.3, 86.51, 79.61, 68.17, 64.16, 56.64, 55.63, 51.66, 51.11, 49.59, 48.96, 48.04, 47.21, 46.47, 42.59, 42.48, 38.06, 37.51, 37.08, 36.38, 35.27, 32.38, 32.3, 30.71, 30.19, 29.95, 29.10, 28.78, 26.98, 26.38, 24.51, 19.44, 19.02, 18.57, 17.68, 15.05. HRMS Calcd for $C_{43}H_{67}NO_6S$ $[M+H]^+$: 710.45369; found: 710.45658. See Figures S162 and S163.

6-((2-(1*H*-indol-3-yl)ethyl)amino)hexyl-acanthosessiligenins (Compound **II-81**). Chloroform / methanol = 30:1 / 20:1, white solid, purity of 98.2%, yield 73%. mp 133-134 °C. ^{1}HNMR(300 MHz, $CDCl_3$): δ ppm 8.68 (*d, J* = 8.1, 1H), 7.60 (*d, J* = 7.8 Hz, 1H), 7.37 (*d, J* = 7.9 Hz, 1H), 7.16 (*t, J* = 7.7 Hz, 1H), 7.07 (*t, J* = 5.4Hz, 1H), 7.05 (*d, J* = 2.5 Hz, 1H), 4.74 (*brs*, 1H), 4.62 (*brs*, 1H), 4.29 (*dd, J*= 11.8, 3.6 Hz, 1H), 4.09−3.95 (*m*, 4H), 3.89 (*dt, J*= 14.7, 9.1 Hz, 1H), 3.65 (*s*, 3H), 3.37−3.20 (*m*, 4H), 3.12 (*d, J* =

3.4 Hz, 1H), 2.96(*dt, J* = 22.6, 10.0 Hz, 1H), 2.31 (*d, J*= 3.8 Hz, 1H), 2.38−2.16 (*m*, 2H),2.04−1.83 (*m*, 5H), 1.68 (*s*, 3H), 1.64 (*d, J* = 4.3 Hz, 1H), 1.61 (d, *J* =2.7 Hz, 1H), 1.62−1.16 (*m*, 19H), 1.24 (*s*, 3H), 1.16 (*s*, 3H), 1.09 (*s*, 3H), 1.00 (*s*, 3H), 0.90 (*s*, 3H). ^{13}C NMR (75 MHz, $CDCl_3$): δ ppm 176.49, 173.57, 149.96, 136.55, 127.22, 123.01, 122.03, 119.35, 118.7, 111.96, 111.51, 110.35, 86.54, 79.7, 68, 64.16, 56.7, 55.65, 51.67, 49.02, 48.97, 48.1, 47.5, 47.3, 46.46, 42.58, 42.48, 37.97, 37.66, 37.12, 36.36, 35.23, 32.47, 32.4, 30.76, 29.9, 28.73, 28.45, 26.81, 26.34, 24.53, 24.34, 19.46, 19.04, 18.55, 17.69, 15.07. HRMS Calcd for $C_{47}H_{70}N_2O_6$ $[M+H]^+$: 757.51611; found: 757.51537. See Figures S164 and S165.

10-(4-formyl-1*H*-imidazol-1-yl)decyl-acanthosessiligenins (Compound **II-82**). Chloroform / methanol = 60:1 / 50:1, white solid, purity of 95.5%, yield 58%. mp 138-139°C. ^{1}H NMR (300 MHz, $CDCl_3$): δ ppm 9.79 (*d, J* = 8.5 Hz, 1H), 8.25 (*d, J* = 8.9 Hz, 1H),7.58 (*d, J* = 5.2 Hz, 1H), 4.73 (*brs*, 1H), 4.60 (*brs*, 1H), 4.25 (*dd, J* = 11.4, 2.8 Hz, 1H), 4.06−4.05 (*dt, J* = 10.7, 6.7 Hz, 2H), 3.99 (*dt, J* = 10.8, 5.0 Hz, 1H), 3.66 (*s*, 3H), 3.41−3.37 (*t, J* = 6.8 Hz, 2H), 3.06 (*d, J* = 2.9 Hz, 1H), 2.98 (*dt, J* = 10.9, Hz3.5 Hz, 1H), 2.30 (*d, J* = 2.7 Hz, 1H), 2.28−2.23 (*m*, 2H), 2.02−1.83 (*m*, 6H), 1.67 (*s*, 3H), 1.60 (*d, J* = 5.0 Hz, 1H), 1.60 (*d, J* = 5.4 Hz, 1H), 1.65−1.25 (*m*, 25H), 1.23 (*s*, 3H), 1.16 (*s*, 3H), 1.09 (*s*, 3H), 0.99 (*s*, 3H), 0.92 (*s*, 3H). ^{13}C NMR (75 MHz, $CDCl_3$): δ ppm 185.28, 176.96, 173.72, 150.50, 142.81, 138.97, 126.17, 110.72, 86.89, 80.48, 68.96, 64.62, 56.98, 56.04, 52.12, 49.37, 48.40, 47.51, 47.46, 46.90, 42.95, 42.89, 38.47, 37.70, 37.48, 36.62, 35.69, 32.80, 32.68, 31.06, 30.33, 30.26, 30.05, 29.75, 29.27, 29.14, 28.94, 26.61, 24.94, 23.80, 19.89, 19.37, 18.99, 18.04, 15.44. HRMS Calcd for $C_{45}H_{70}N_2O_7$ $[M+H]^+$: 749.51103; found: 749.51378. See Figures S166 and S167.

10-(2-propyl-1*H*-imidazol-1-yl)decyl-acanthosessiligenins (Compound **II-83**). Chloroform / methanol = 50:1 / 40:1, white solid, purity of 99.9%, yield 65%. mp 144-145 °C. ^{1}H NMR (300 MHz,$CDCl_3$): δ ppm 6.94 (*d, J* = 2.5 Hz, 1H), 6.91 (*d, J* = 1.3 Hz, 1H), 4.73 (*brs*, 1H), 4.60 (*brs*, 1H), 4.23 (*dd, J* = 11.4, 2.8Hz, 1H), 4.06−4.04 (*dt, J* = 10.7, 6.7 Hz, 2H), 3.91 (*dt, J* = 10.4, 4.5 Hz, 1H), 3.66 (*s*, 3H), 3.41−3.37 (*t, J* = 6.8 Hz, 2H), 3.09 (*d, J* = 2.8 Hz, 1H), 2.98 (*dt, J* = 10.9 Hz, 3.5 Hz, 1H), 2.30 (*d, J* = 2.7 Hz, 1H), 2.29−2.24 (*m*, 4H), 2.03−1.78 (*m*, 6H), 1.67 (*s*, 3H), 1.65 (*d, J* = 5.0 Hz, 1H), 1.61 (*d, J* = 5.4 Hz, 1H), 1.64−1.11 (*m*, 27H), 1.24 (*s*, 3H), 1.17 (*s*, 3H), 1.09(*s*, 3H), 1.00 (*s*, 3H) 0.99 (*s*, 3H), 0.92 (*s*, 3H). ^{13}C NMR (75 MHz, $CDCl_3$): δ ppm 176.16,

173.32, 150.01, 126.89, 120.96, 118.76, 110.21, 86.47, 79.59, 68.13, 64.14, 56.53, 55.58, 51.59, 48.93, 47.98, 47.02, 46.45, 45.82, 42.45, 42.43, 38.01, 37.26, 37.03, 36.19, 35.2, 32.32, 32.23, 31.01, 30.62, 30.21, 29.86, 29.48, 29.42, 29.22, 28.77, 26.68, 26.12, 24.47, 21.93, 19.41, 18.87, 18.53, 17.56, 14.96, 14.07, 13.85. HRMS Calcd for $C_{47}H_{76}N_2O_6$ $[M+H]^+$: 763.56306; found: 763.56347. See Figures S168 and S169.

10-(2,4-dimethyl-1*H*-imidazol-1-yl)decyl-acanthosessiligenins (Compound **II-84**). Chloroform / methanol = 55:1 / 45:1, white solid, purity of 97.9%, yield 58%. mp 120-121 °C. ^1H NMR (300 MHz, $CDCl_3$): δ ppm 6.14 (*d, J* = 3.1Hz, 1H), 4.75 (*brs*, 1H), 4.62 (*brs*, 1H), 4.21 (*dd, J* = 11.4, 2.8 Hz, 1H), 4.05 (*dt, J* = 10.7, 6.7 Hz, 2H), 3.98 (*dt, J* = 10.8, 5.0 Hz, 1H), 3.67 (*s*, 3H), 3.41−3.37 (*t, J* = 6.8 Hz, 2H), 3.11 (*d, J* = 2.9 Hz, 1H), 2.99 (*dt, J* = 10.9 Hz, 3.5 Hz, 1H), 2.30 (*d, J* = 2.7 Hz, 1H), 2.29−2.24 (*m*, 2H), 2.04−1.85 (*m*, 12H), 1.68 (*s*, 3H), 1.61 (*d, J* = 5.0 Hz, 1H), 1.60 (*d, J* = 5.4 Hz, 1H), 1.65−1.13 (*m*, 25H), 1.24 (*s*, 3H), 1.18 (*s*, 3H), 1.10 (*s*, 3H), 1.00 (*s*, 3H), 0.93 (*s*, 3H). ^{13}C NMR (75 MHz, $CDCl_3$): δ ppm 176.94, 173.73, 150.07, 145.62, 135.41, 115.72, 110.13, 86.83, 80.11, 69.22, 64.65, 57.37, 55.61, 52.41, 48.93, 47.97, 47.08, 47.04, 46.47, 42.52, 42.47, 38.05, 37.27, 37.06, 36.19, 35.26, 32.38, 32.26, 30.64, 30.54, 29.91, 29.75, 29.64, 29.46, 29.33, 28.85, 26.19, 25.71, 24.52, 19.46, 18.95, 18.56, 17.61, 15.02, 13.86, 12.65. HRMS Calcd for $C_{46}H_{74}N_2O_6$ $[M+H]^+$: 749.54741; found: 749.54241. See Figures S170 and S171.

10-(5-bromo-1*H*-imidazol-1-yl)decyl-acanthosessiligenins (Compound **II-85**). Chloroform / methanol = 55:1 / 40:1, white solid, purity of 96.2%, yield 67%. mp 135-136 °C. ^1H NMR (300 MHz, $CDCl_3$): δ ppm 7.64 – 7.30 (*m*, 1H), 7.06 – 6.82 (*m*, 1H), 4.74 (*brs*, 1H), 4.61 (*brs*, 1H), 4.27 (*dd*, J = 11.4, 2.8 Hz, 1H), 4.10−3.82 (*dt, J* = 10.7, 6.7 Hz, 2H), 3.92 (*dt, J* = 10.8, 5.0 Hz, 1H), 3.66 (*s*, 3H), 3.41−3.37 (*t, J* = 6.8 Hz, 2H), 3.06 (*d, J* = 2.9 Hz, 1H), 2.98 (*dt, J* = 10.9 Hz, 3.5 Hz, 1H), 2.31 (*d, J* = 2.7 Hz, 1H), 2.39−2.21 (*m*, 2H), 2.05−1.71 (*m*, 6H), 1.68 (*s*, 3H), 1.64 (*d, J* = 5.0 Hz, 1H), 1.61 (*d, J* = 5.4 Hz, 1H), 1.59−1.05 (*m*, 25H), 1.23 (*s*, 3H), 1.17 (*s*, 3H), 1.10 (*s*, 3H), 1.00 (*s*, 3H), 0.92 (*s*, 3H). ^{13}C NMR (75 MHz, $CDCl_3$): δ ppm 176.2, 173.39, 150.1, 136.77, 118.21, 115.18, 110.25, 86.47, 79.72, 68.38, 64.19, 56.55, 55.61, 51.69, 48.94, 47.82, 47.05, 46.45, 46.26, 42.5, 42.45, 37.97, 37.28, 37.05, 36.15, 35.22, 32.36, 32.25, 30.64, 30.49, 29.88, 29.33, 29.24, 29.09, 29.04, 28.81, 26.43, 26.15, 24.5, 19.43, 18.93, 18.53,

17.58, 15. HRMS Calcd for $C_{44}H_{69}BrN_2O_6$ $[M+H]^+$: 799.42662; found: 799.42537. See Figures S172 and S173.

10-(2-ethyl-4-methyl-1*H*-imidazol-1-yl)decyl-acanthosessiligenins (Compound **II-86**). Chloroform / methanol = 45:1 / 30:1, white solid, purity of 95.9%, yield 69%. mp 196-197 °C. ^{1}H NMR (300 MHz, $CDCl_3$): δ ppm 6.49 (*d, J* = 1.1 Hz, 1H), 4.73 (*brs*, 1H), 4.61 (*brs*, 1H), 4.27 (*dd, J* = 11.4, 2.8 Hz, 1H), 4.06−4.05 (*dt, J* = 10.7, 6.7 Hz, 2H), 3.78 (*dt, J* = 10.8, 5.0 Hz, 1H), 3.66 (*s*, 3H), 3.41−3.37 (*t, J* = 6.8 Hz, 2H), 3.06 (*d, J* = 2.9 Hz, 1H), 2.98 (*dt, J* = 10.9 Hz, 3.5 Hz, 1H), 2.31 (*d, J* = 2.7 Hz, 1H), 2.38−2.21 (*m*, 4H), 2.17−1.78 (*m*, 10H), 1.68 (*s*, 3H), 1.65 (*d, J* = 5.0 Hz, 1H), 1.61 (*d, J* = 5.4 Hz, 1H), 1.57−1.05 (*m*, 27H), 1.23 (*s*, 3H), 1.17 (*s*, 3H), 1.09 (*s*, 3H), 1.00 (*s*, 3H), 0.92 (*s*, 3H). ^{13}C NMR (75 MHz, $CDCl_3$): δ ppm 176.15, 173.32, 150.02, 148.4, 135.73, 115.22, 110.21, 86.45, 79.57, 68.23, 64.15, 56.53, 55.58, 51.62, 48.93, 47.98, 47.01, 46.44, 45.58, 42.47, 42.43, 38, 37.26, 37.03, 36.19, 35.21, 32.23, 31.04, 30.62, 29.87, 29.51, 29.44, 29.24, 28.78, 28.60, 26.73, 26.12, 24.46, 23.32, 20.11, 19.42, 18.89, 18.52, 17.56, 14.97, 13.52, 12.67. HRMS Calcd for $C_{47}H_{76}N_2O_6$ $[M+H]^+$: 763.56306; found: 763.56389. See Figures S174 and S175.

10-(pyrrolidin-1-yl)decyl-acanthosessiligenins (Compound **II-87**). Chloroform / methanol = 50:1 / 40:1, s white solid, purity of 95.7%, yield 67%. mp 121-122 °C. ^{1}H NMR (300 MHz, $CDCl_3$): δ ppm 4.74 (*brs*, 1H), 4.61 (*brs*, 1H), 4.24 (*dd, J* = 11.4, 2.8 Hz, 1H), 4.06−4.04 (*dt, J* = 10.7, 6.7 Hz, 2H), 3.97 (*dt, J* = 10.8, 5.0 Hz, 1H), 3.65 (*s*, 3H), 3.41−3.37 (*t, J* = 6.8 Hz, 2H), 3.00 (*d, J* = 2.9 Hz, 1H), 2.98 (*dt, J* = 10.9 Hz, 3.5 Hz, 1H), 2.33 (*d, J* = 2.7 Hz, 1H), 2.23−2.22 (*m*, 7H), 2.02−1.81 (*m*, 5H), 1.67 (*s*, 3H), 1.62 (*d, J* = 5.0 Hz, 1H), 1.33 (*d, J* = 5.4 Hz, 1H), 1.64−1.11 (*m*, 29H), 1.22 (*s*, 3H), 1.16 (*s*, 3H), 1.08 (*s*, 3H), 0.99 (*s*, 3H), 0.91 (*s*, 3H).^{13}C NMR (75 MHz, $CDCl_3$): δ ppm 176.27, 173.31, 150.01, 110.31, 86.46, 79.62, 68.44, 64.22, 56.57, 55.74, 55.58, 53.71, 51.62, 48.92, 47.96, 47.05, 46.94, 46.44, 42.48, 42.45, 37.96, 37.33, 37.1, 36.14, 35.21, 32.34, 32.29, 30.65, 29.86, 29.78, 29.5, 29.28, 29.26, 29.06, 28.82, 26.85, 26.24, 25.75, 24.49, 23.46, 19.46, 18.95, 18.52, 17.58, 15. HRMS Calcd for $C_{45}H_{75}NO_6$ $[M+H]^+$: 724.55216; found: 724.55587. See Figures S176 and S177.

10-((2-(5-methoxy-1*H*-indol-3-yl)ethyl)amino)decy-acanthosessiligenins (Compound **II-88**). Chloroform / methanol = 30:1 / 20:1, white solid, purity of 96.7%, yield 63%.

mp 127-128 °C. ^{1}H NMR (300 MHz, $CDCl_3$): δ ppm 8.37 (*brs*, 1H), 7.23 (*d, J* = 6.7 Hz, 1H), 7.07 (*dd, J* = 7.5, 2.3 Hz, 1H), 6.83 (*dd, J* = 8.8, 2.5 Hz, 1H), 4.71 (*brs*, 1H), 4.61 (*brs*, 1H), 4.28 (*dd, J* = 11.4, 2.8 Hz, 1H), 4.06−4.04 (*dt, J* = 10.7, 6.7 Hz, 2H), 3.86 (*dt, J* = 10.8, 5.0 Hz, 1H), 3.85 (*s*, 3H), 3.66 (*s*, 3H), 3.41−3.37 (*t, J* = 6.8 Hz, 2H), 3.08 (*d, J* = 2.9 Hz, 1H), 2.98 (*dt, J* = 10.9 Hz, 3.5 Hz, 1H), 2.32 (*d, J* = 2.7 Hz, 1H), 2.36−2.24 (*m*, 7H), 2.02−1.87 (*m*, 7H), 1.68 (*s*, 3H), 1.65 (*d, J* = 5.0 Hz, 1H), 1.58 (*d, J* = 5.4 Hz, 1H), 1.55−1.05 (*m*, 25H), 1.23 (*s*, 3H), 1.16 (*s*, 3H), 1.10 (*s*, 3H), 1.00 (*s*, 3H), 0.91 (*s*, 3H). ^{13}C NMR (75 MHz, $CDCl_3$): δ ppm 176.3, 173.49, 154.01, 150.04, 131.67, 127.57, 123.55, 112.25, 112.23, 111.74, 110.28, 100.56, 86.47, 79.73, 68.26, 64.24, 56.57, 55.61, 55.43, 51.66, 49.02, 48.99, 48.98, 48, 47.06, 46.43, 42.46, 42.44, 37.95, 37.34, 37.08, 36.17, 35.19, 32.36, 32.27, 30.66, 29.87, 29.51, 29.42, 29.32, 29.19, 28.8, 28.07, 27.1, 26.15, 24.5, 24.12, 19.44, 18.92, 18.51, 17.57, 14.99. HRMS Calcd for $C_{52}H_{80}N_2O_7$ $[M+H]^+$: 843.58928; found: 843.58967. See Figures S178 and S179.

10-((2-(thiophen-2-yl)ethyl)amino)decyl-acanthosessiligenins (Compound **II-89**). Chloroform / methanol = 35:1 / 25:1, white solid, purity of 95.0%, yield 71%. mp 100-101°C. ^{1}H NMR (300 MHz,$CDCl_3$): δ ppm7.14 (*dd, J* = 5.1, 1.2 Hz, 1H), 6.94 (*dd, J* = 5.1, 3.4 Hz, 1H), 6.85 (*d, J* = 3.4 Hz, 1H), 4.74 (*brs*, 1H), 4.61 (brs, 1H), 4.26 (*dd, J* = 11.4, 2.8 Hz, 1H), 4.06−4.04 (*dt, J* = 10.7, 6.7 Hz, 2H), 3.92 (*dt, J* = 10.8, 5.0 Hz, 1H), 3.67 (*s*, 3H), 3.41−3.25 (*t, J* = 6.8 Hz, 2H), 3.05 (*d, J* = 2.9 Hz, 1H), 2.98 (*dt, J* = 10.9 Hz, 3.5 Hz, 1H), 2.31 (*d, J* = 2.7 Hz, 1H), 2.40−2.22 (*m*, 7H), 2.02−1.85 (*m*, 6H), 1.68 (*s*, 3H), 1.65 (*d, J* = 5.0 Hz, 1H), 1.61 (*d, J* = 5.4 Hz, 1H), 1.55−1.05 (*m*, 25H), 1.24 (*s*, 3H), 1.18 (*s*, 3H), 1.10 (*s*, 3H), 1.00 (*s*, 3H), 0.93 (*s*, 3H).^{13}C NMR (75 MHz, $CDCl_3$): δ ppm 176.14, 173.31, 142.47, 127.52, 125.79, 122.88, 110.28, 86.46, 79.62, 68.50, 64.19, 56.55, 55.61, 51.70, 49.53, 48.94, 47.97, 47.03, 46.46, 46.26, 42.52, 42.46, 38.04, 37.27, 37.05, 36.19, 35.26, 32.37, 32.30, 32.25, 30.70, 30.64, 29.91, 29.62, 29.31, 29.13, 28.84, 28.57, 26.18, 25.39, 24.51, 23.53, 19.47, 18.94, 18.56, 17.61, 15.01. HRMS Calcd for $C_{47}H_{75}NO_6S$ $[M+H]^+$: 780.52423; found: 780.52657. See Figures S180 and S181.

10-((2-(1*H*-indol-3-yl)ethyl)amino)decyl-acanthosessiligenins (Compound **II-90**). Chloroform / methanol = 30:1 / 20:1, white solid, purity of 95.5%, yield 64%. mp 104-105 °C. ^{1}HNMR(300 MHz, $CDCl_3$): δ ppm 8.55 (*d, J* = 7.7 Hz, 1H), 7.62 (*d, J* = 7.7 Hz, 1H), 7.36 (*d, J* = 7.9 Hz, 1H), 7.16 (*t, J* = 4.5 Hz, 1H), 7.09 (*t, J* = 11.9,4.3 Hz, 1H),

7.05 (*d, J* = 3.4 Hz, 1H), 4.75 (*brs*, 1H), 4.62 (*brs*, 1H), 4.26 (*dd, J* = 11.3, 3.4 Hz, 1H), 4.07−4.03 (*t, J* = 6.6 Hz, 2H), 3.95−3.87 (*m*, 2H), 3.88 (*dt, J* = 11.0, 5.8, 4.7 Hz, 1H), 3.65 (*s*, 3H), 3.47−3.27 (*m*, 4H), 3.08 (*d, J* = 4.1 Hz, 1H), 2.98 (*dd,* J = 14.3, 6.1 Hz, 1H),2.36−2.27 (*m*, 2H), 2.32 (*d, J* = 2.3 Hz, 1H), 2.04−1.80 (*m*, 7H), 1.69(*s*, 3H), 1.64 (*d, J* = 11.5 Hz, 1H), 1.58 (*d, J* = 2.4 Hz, 1H), 1.65−1.15(*m*, 25H), 1.24 (*s*, 3H), 1.17 (*s*, 3H), 1.10 (*s*, 3H), 1.00 (*s*, 3H), 0.92 (*s*,3H). ^{13}C NMR (75 MHz, $CDCl_3$): δ ppm 176.31, 173.42, 150.06, 136.53, 127.73, 122.78, 122.17, 119.51, 118.75, 111.75, 111.48, 110.29, 86.48, 79.69, 68.37, 64.26, 56.6, 55.64, 51.68, 48.97, 48.88, 48.02, 47.09, 46.48, 42.52, 42.48, 38.01, 37.38, 37.11, 36.21, 35.24, 32.39, 32.3, 32.15, 30.69, 29.9, 29.52, 29.41, 29.29, 29.2, 28.83, 27.72, 27.08, 26.17, 24.54, 23.81, 19.48, 18.95, 18.56, 17.61, 15.03. HRMS Calcd for $C_{51}H_{78}N_2O_6$ $[M+H]^+$: 813.57871; found: 813.57634. See Figures S182 and S183.
