# Supplementary material for: Design and synthesis of 3,4-seco-Lupane triterpene derivatives: targeting tumor angiogenesis and inducing apoptosis in triple-negative breast cancer
Source: Front Chem. 2025 Jul 31;13:1630939. doi: 10.3389/fchem.2025.1630939 (PMC12351286; doi:10.3389/fchem.2025.1630939)

**TSP-1·(Cell Repeat·1)**


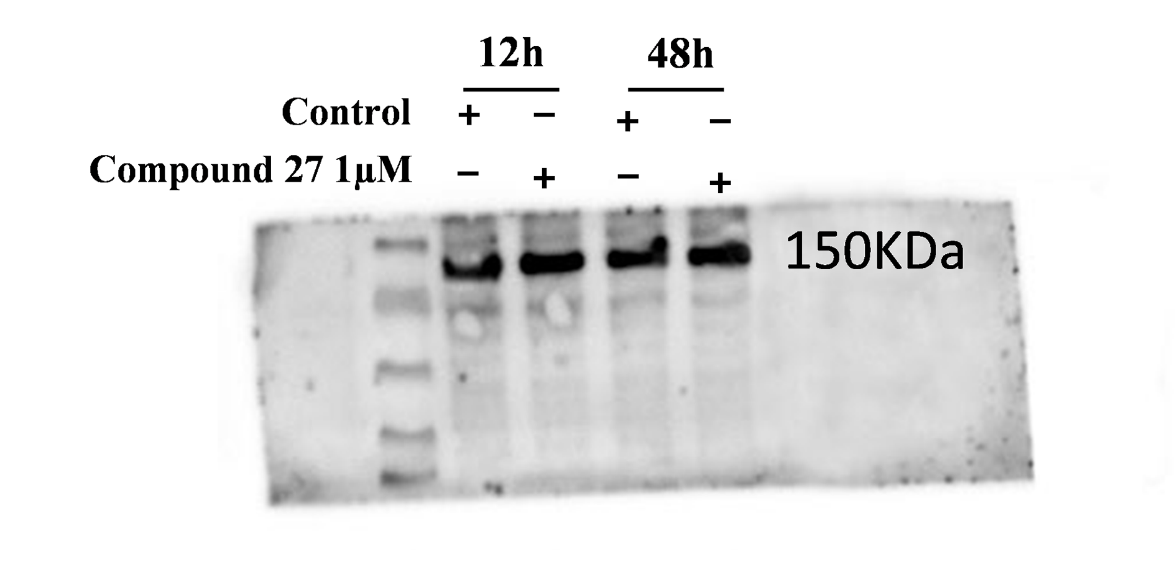


**TSP-1·(Cell Repeat·2)**


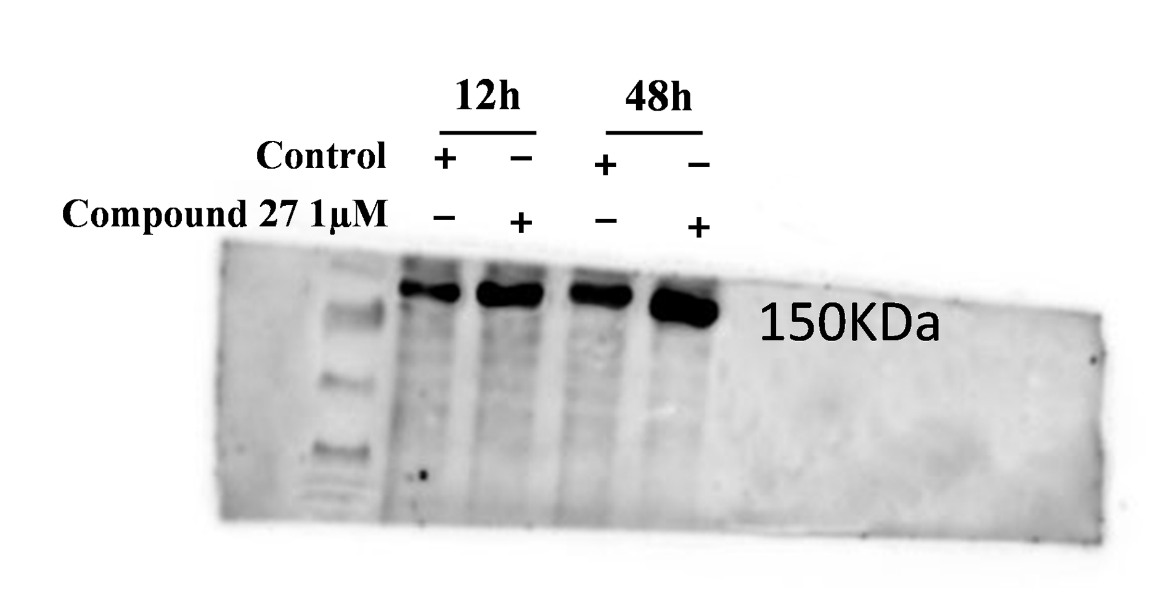


**TSP-1·(Cell Repeat·3)**


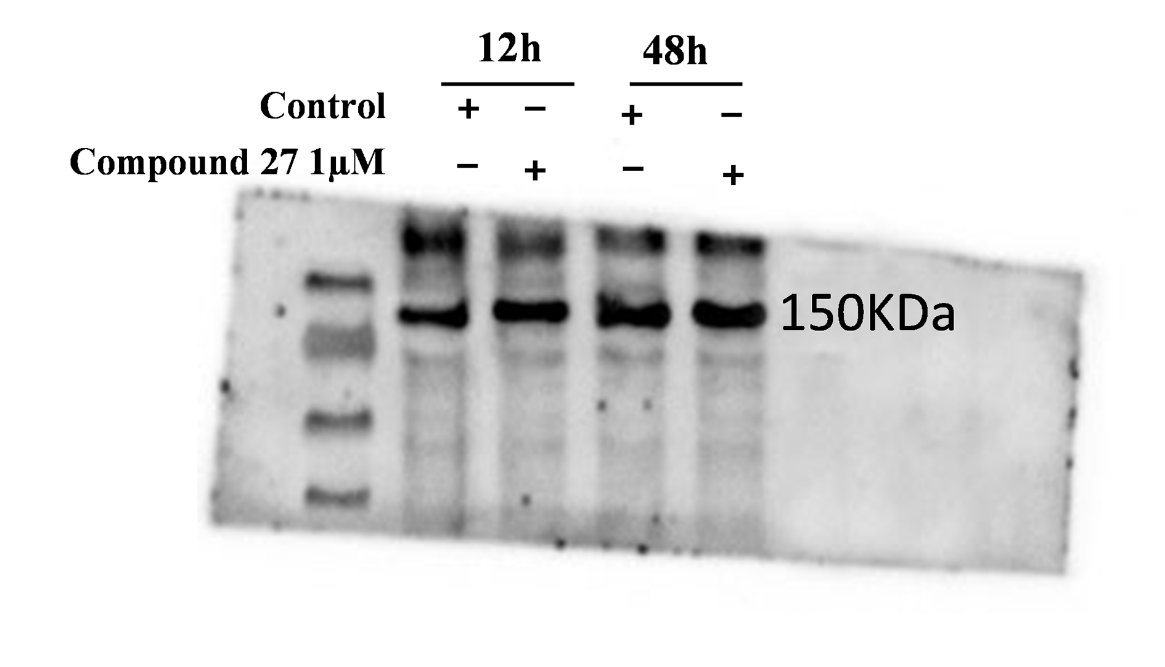


**ID1·(Cell Repeat·1)**


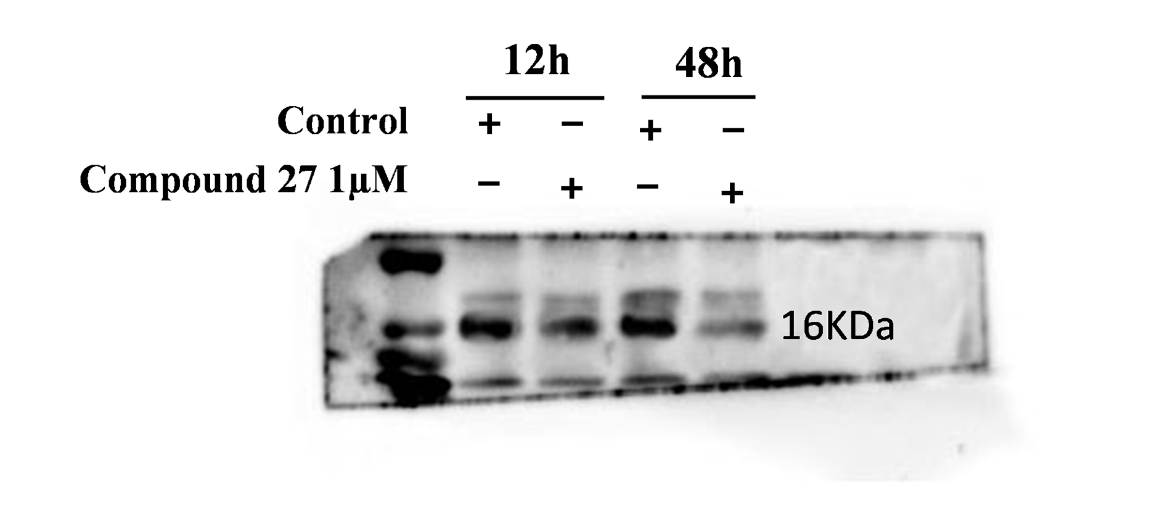


**ID1·(Cell Repeat·2)**


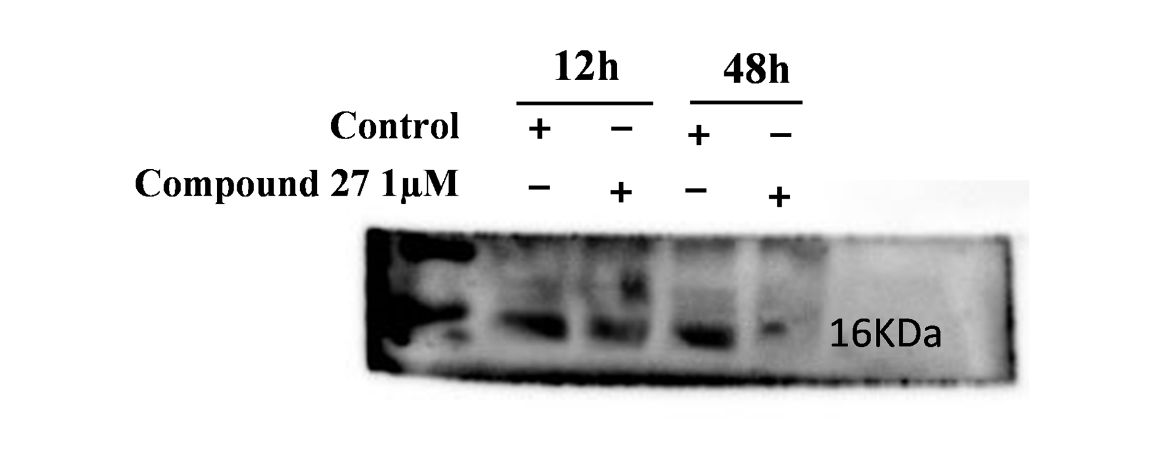


**ID1·(Cell Repeat·3)**


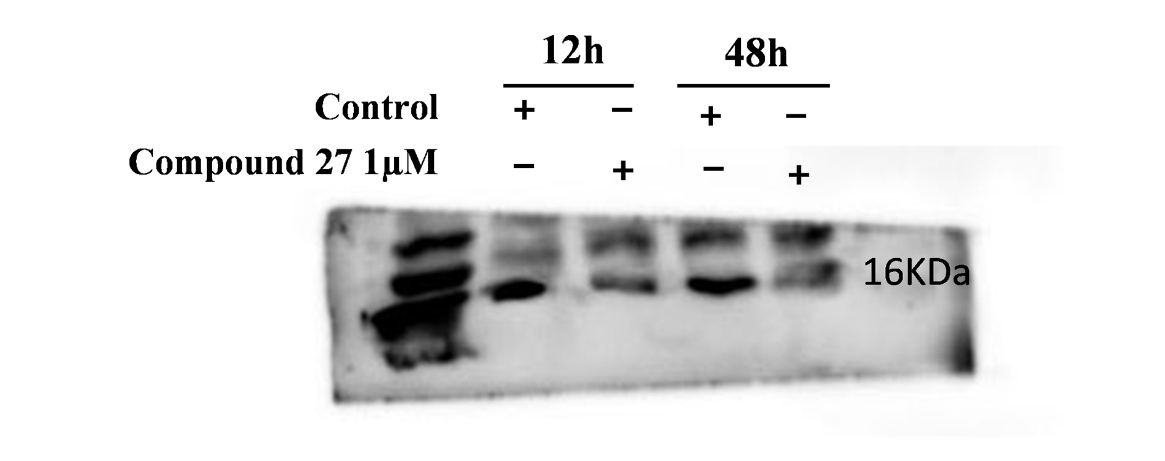


**β-actin (Cell Repeat·1)**


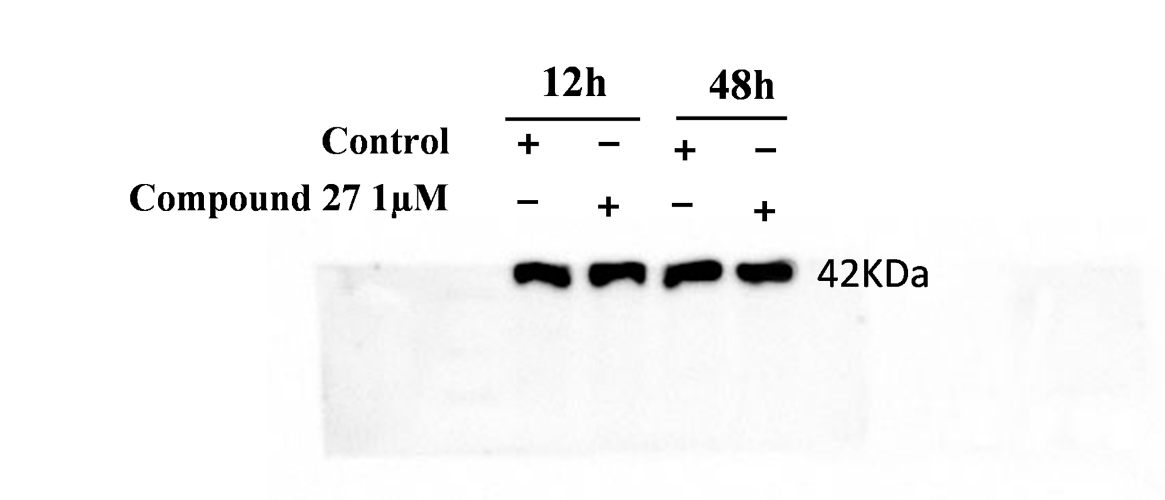


**β-actin (Cell Repeat·2)**


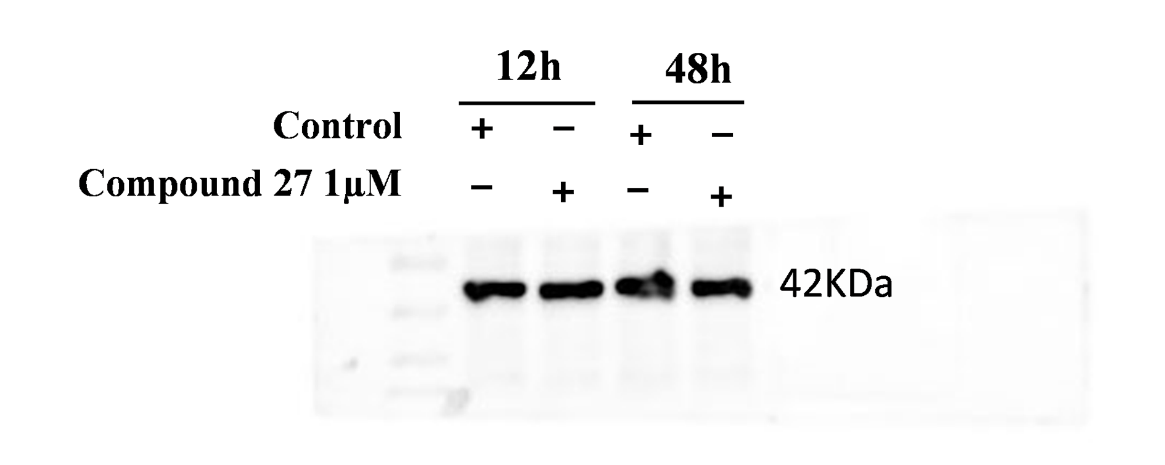


**β-actin (Cell Repeat·3)**


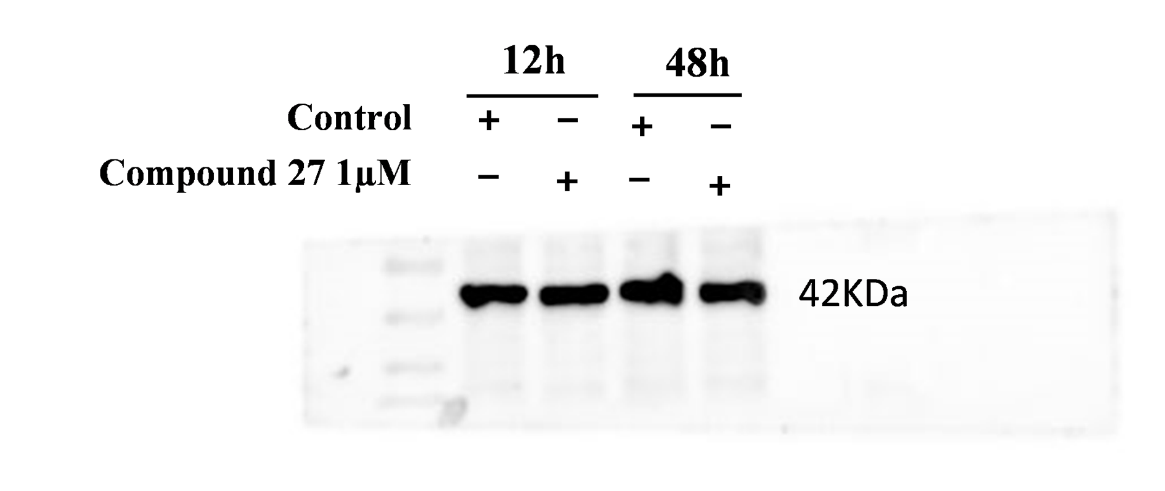


**PI3K·(Cell Repeat·1)**


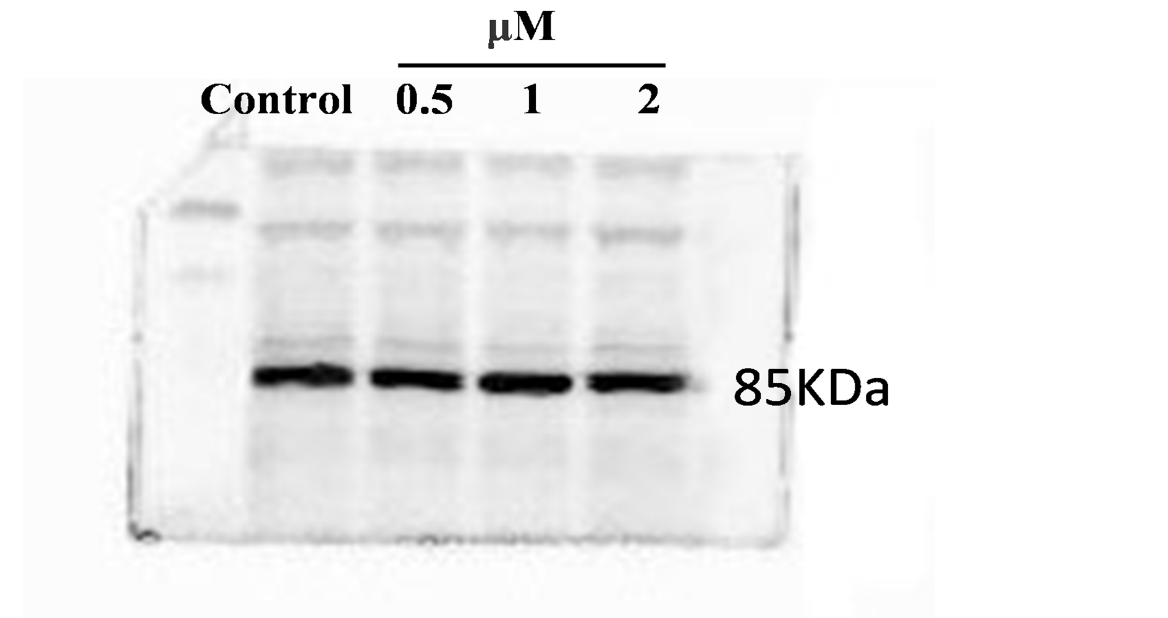


**PI3K·(Cell Repeat·2)**


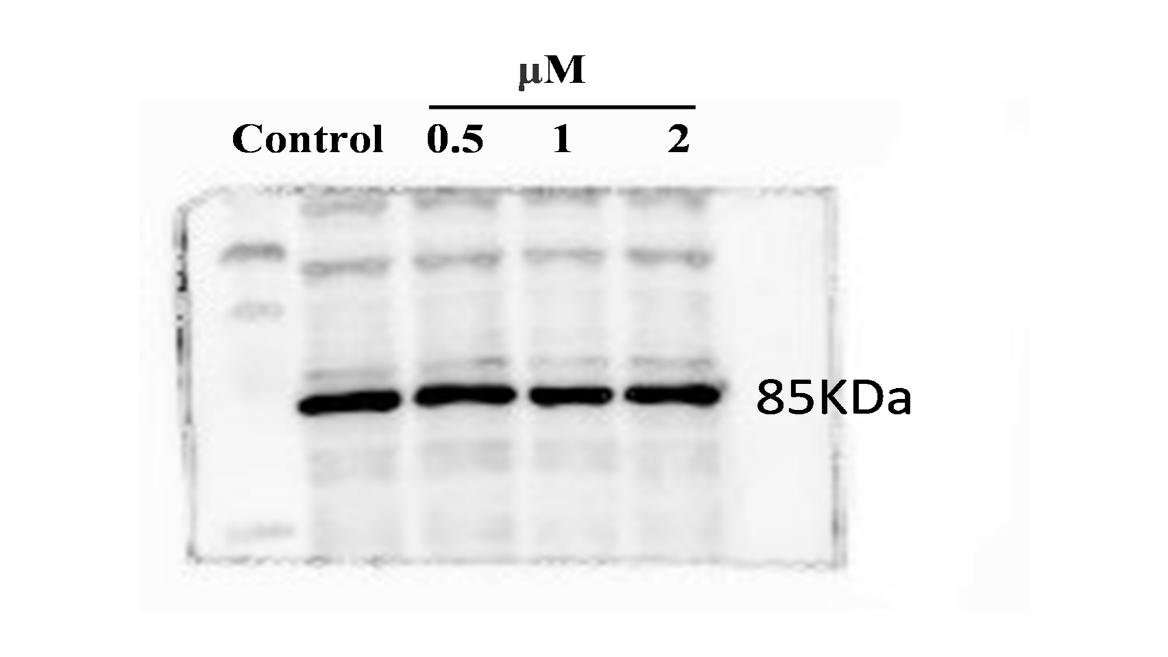
**PI3K·(Cell Repeat·2)**


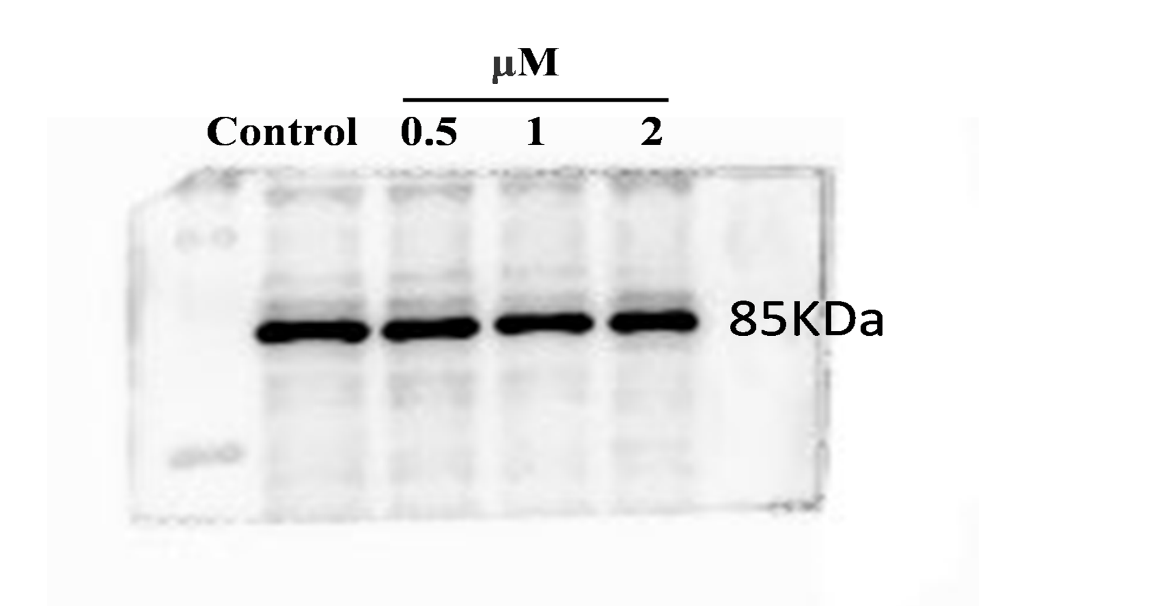


**p- PI3K·(Cell Repeat·1)**


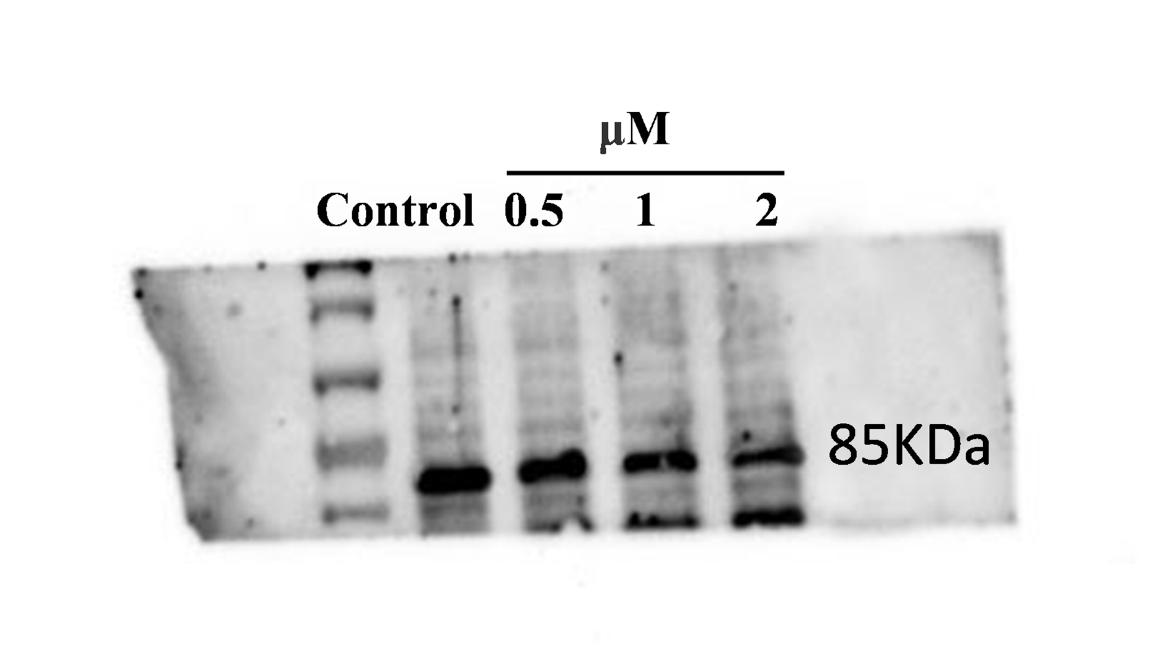


**p- PI3K·(Cell Repeat·2)**


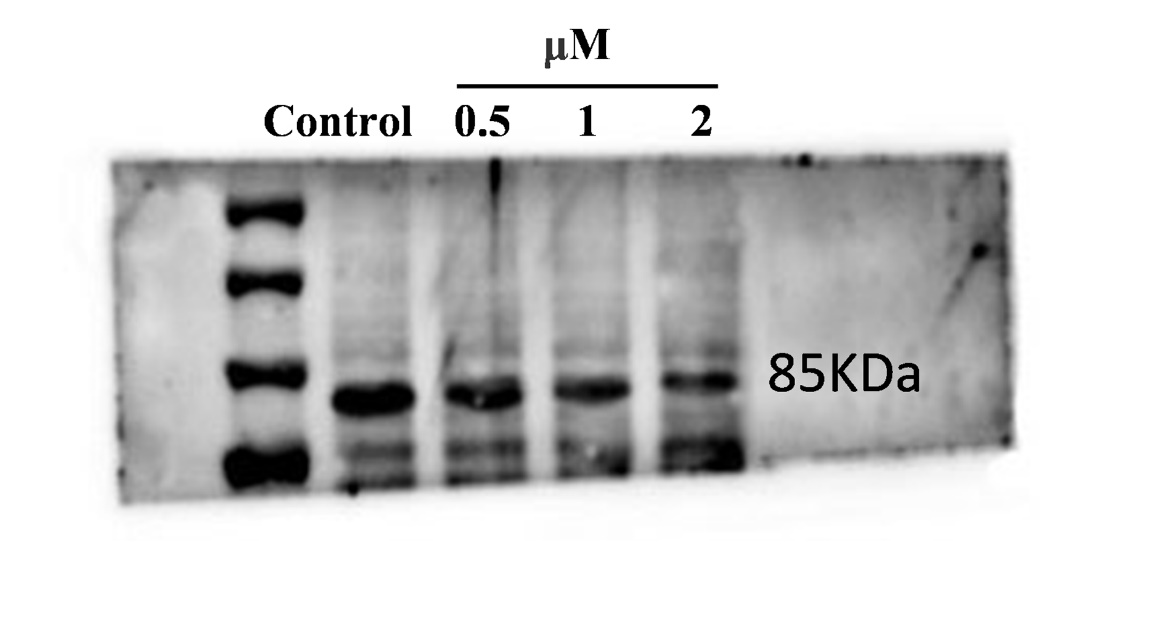


**p- PI3K·(Cell Repeat·2)**


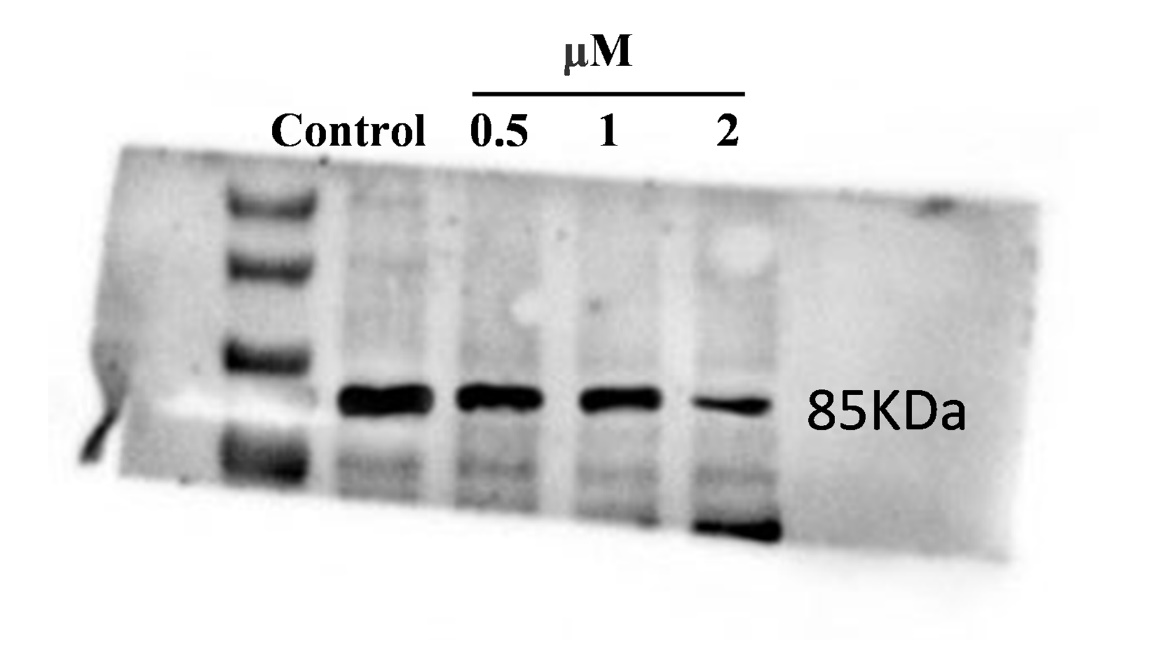


**AKT·(Cell Repeat·1)**


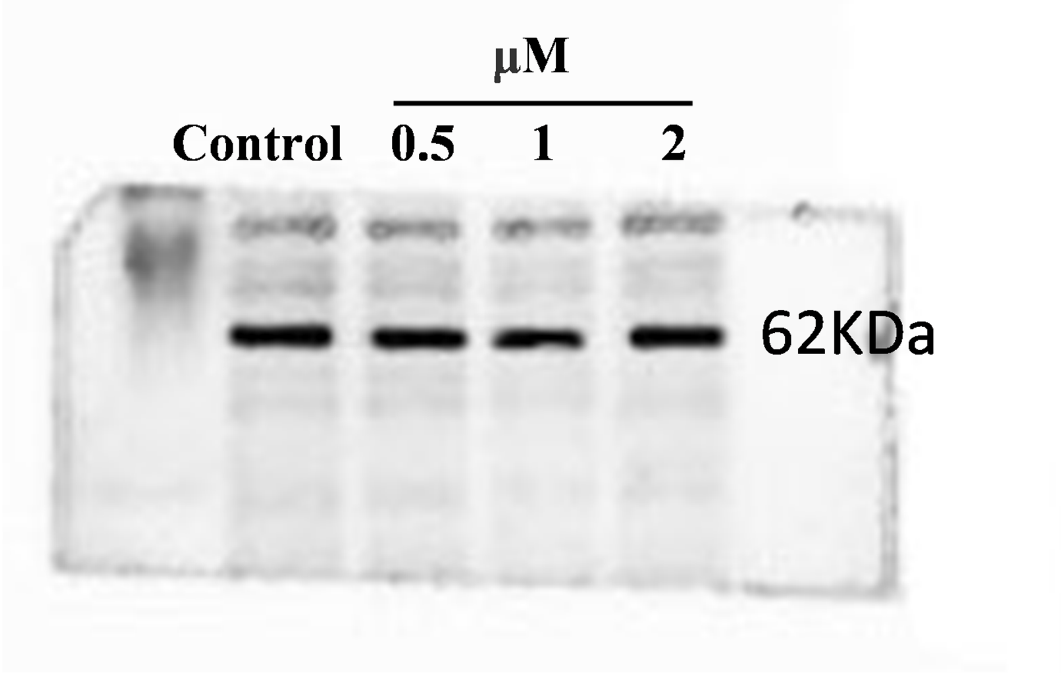


**AKT·(Cell Repeat·2)**


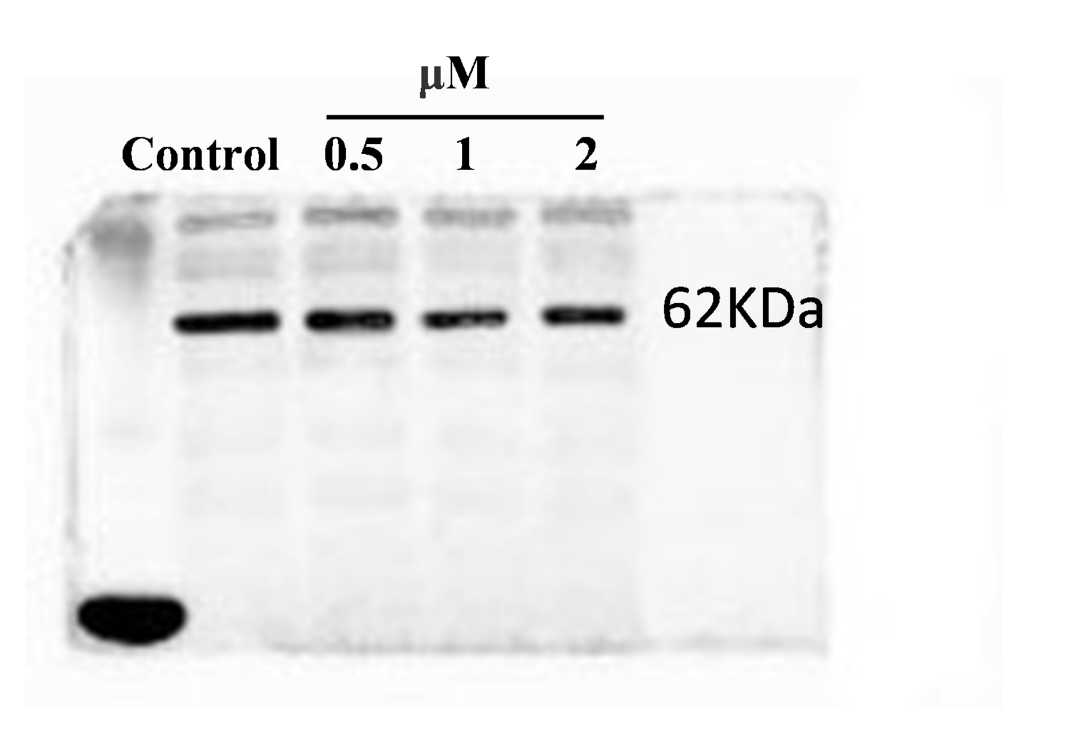


**AKT·(Cell Repeat·3)**


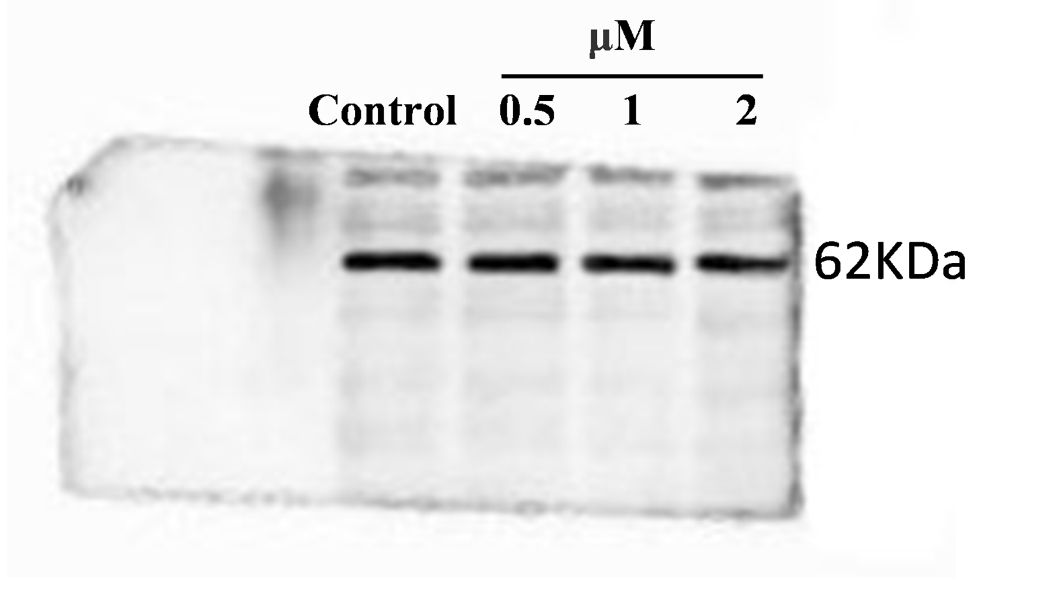


**p-AKT·(Cell Repeat·1)**


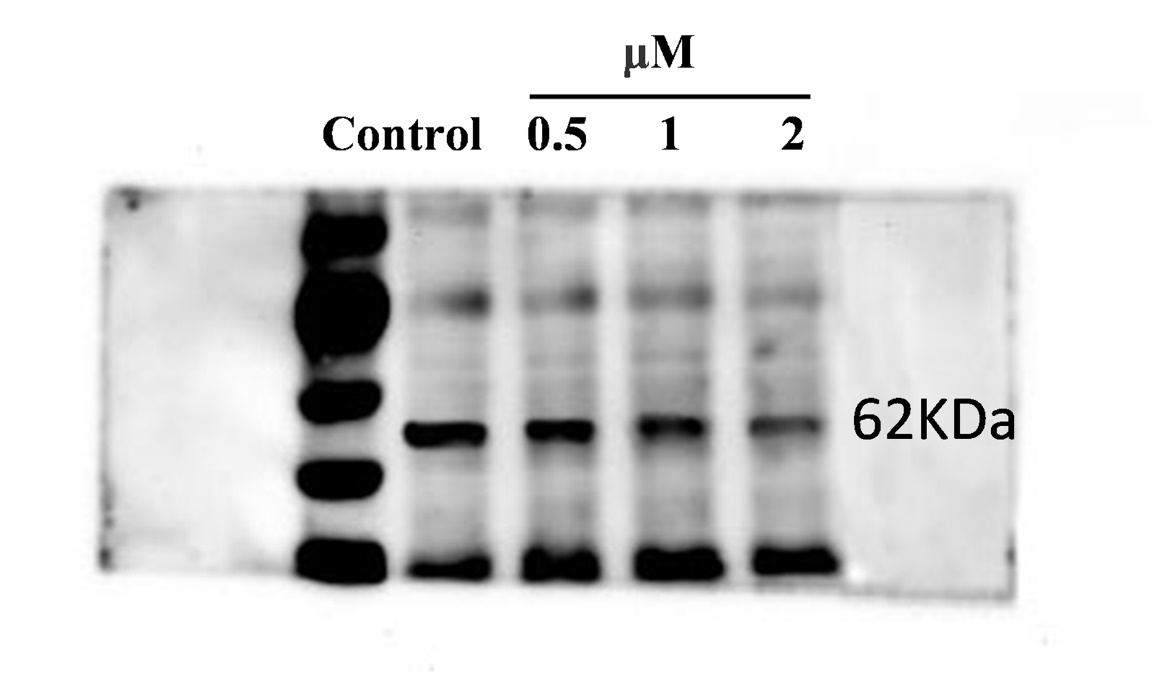


**p-AKT·(Cell Repeat·2)**


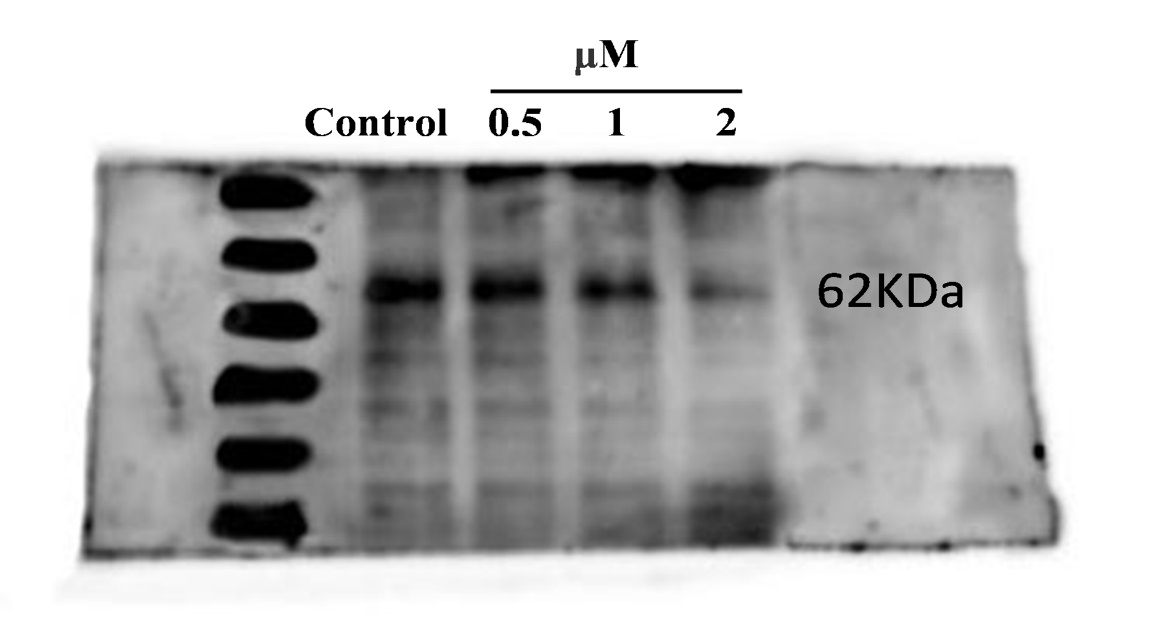


**p-AKT·(Cell Repeat·3)**


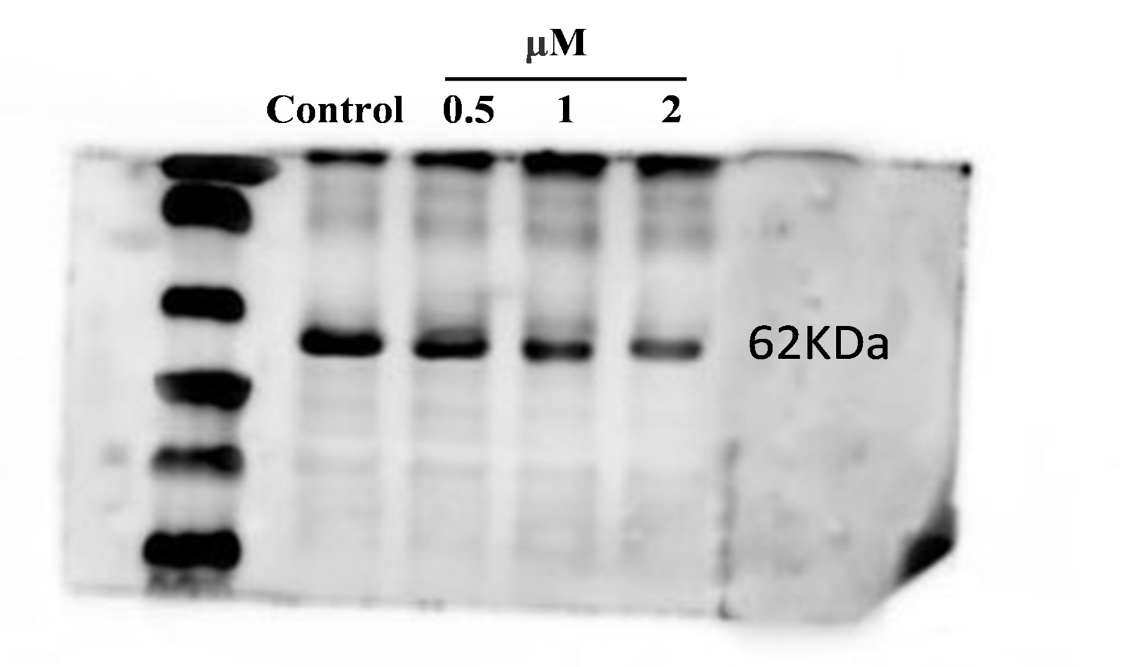


**FoxO1 (Cell Repeat·1)**


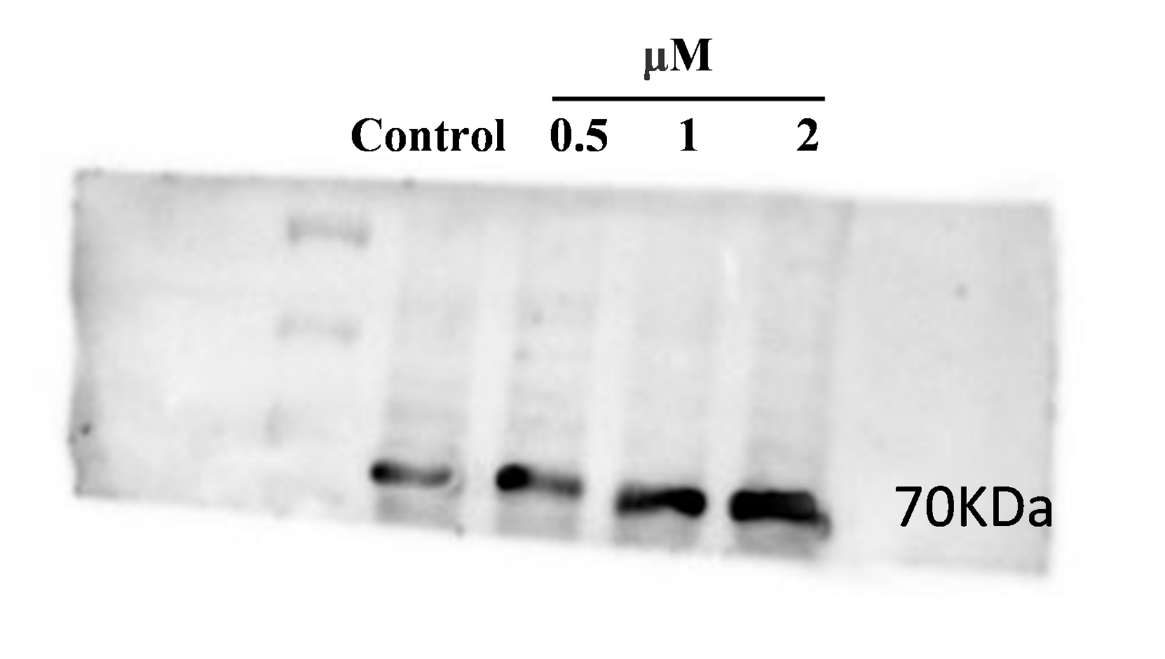


**FoxO1 (Cell Repeat·2)**


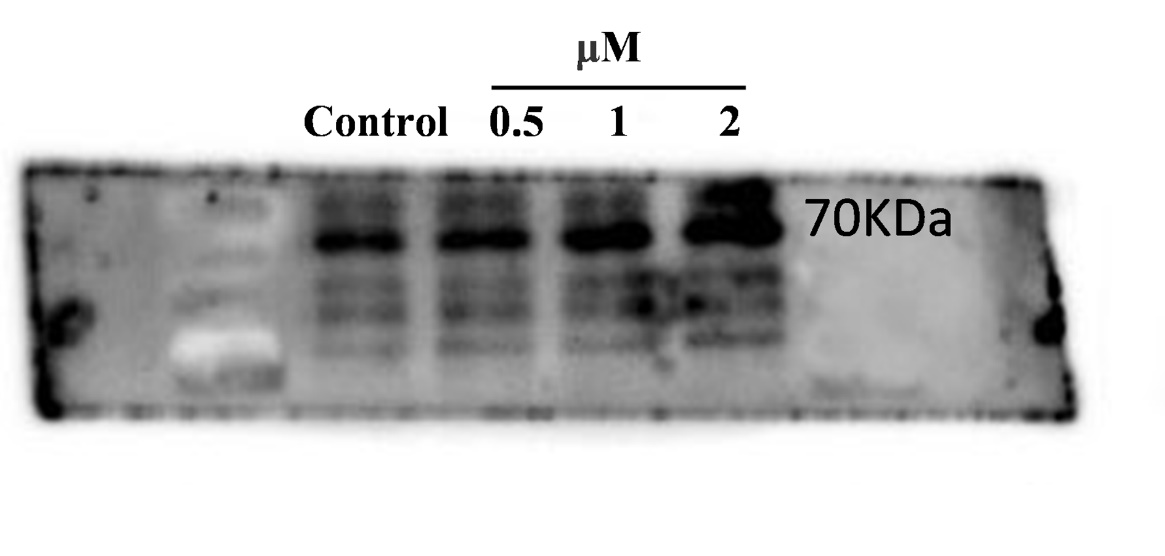


**FoxO1 (Cell Repeat·3)**


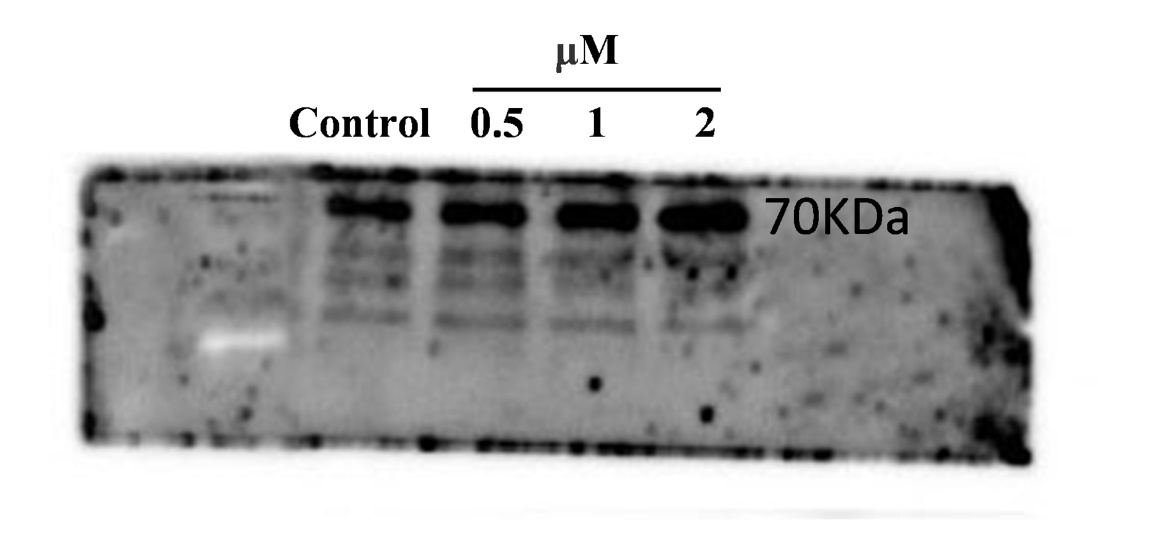


**β-actin (Cell Repeat·1)**


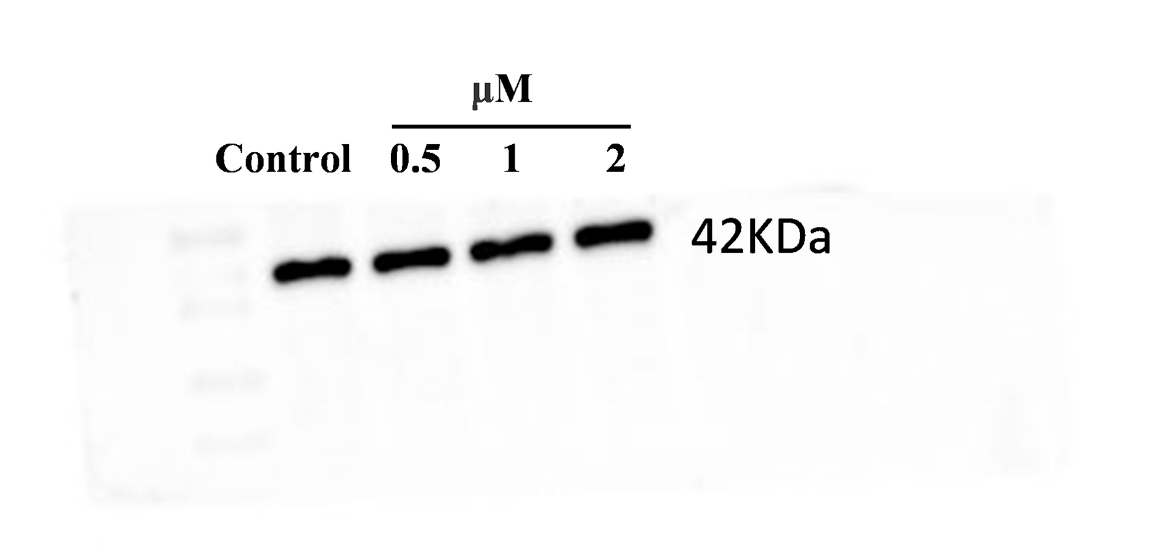


**β-actin (Cell Repeat·2)**


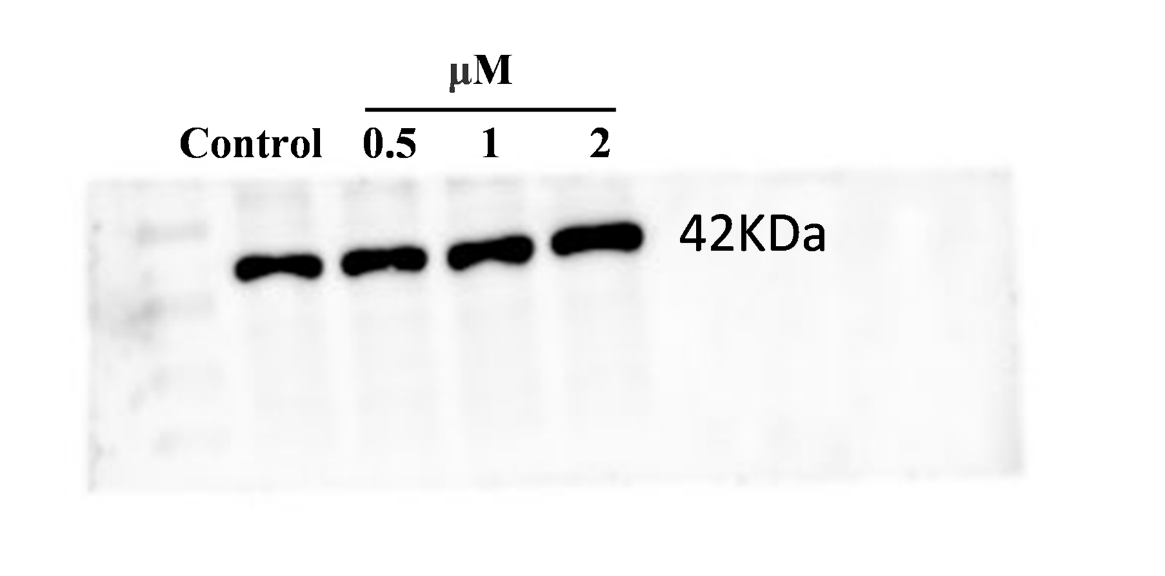


**β-actin (Cell Repeat·3)**


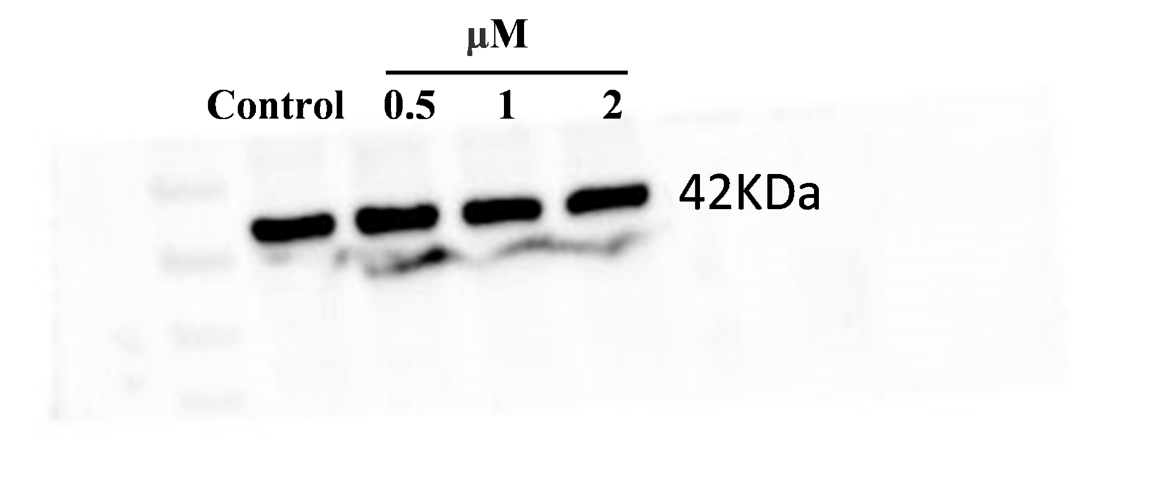


**KD-ID1 (Cell Repeat·1)**


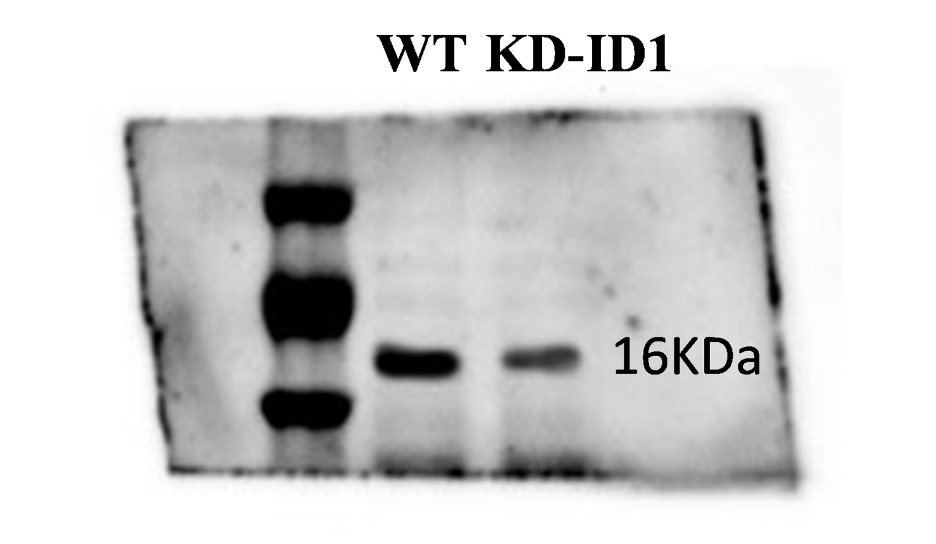


**KD-ID1 (Cell Repeat·2)**


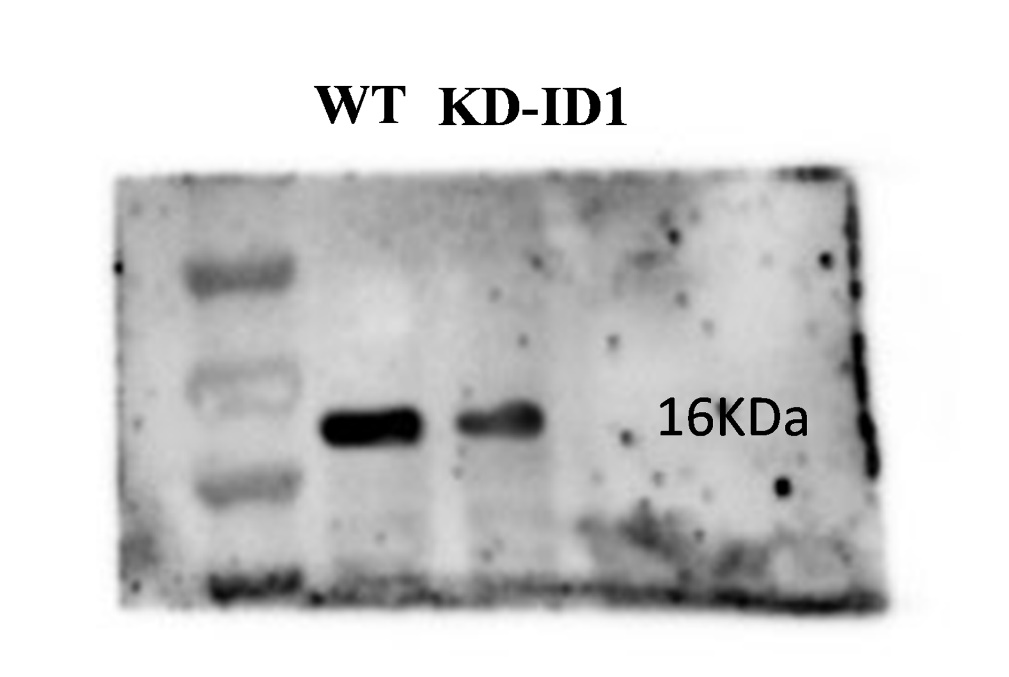


**KD-ID1 (Cell Repeat·3)**


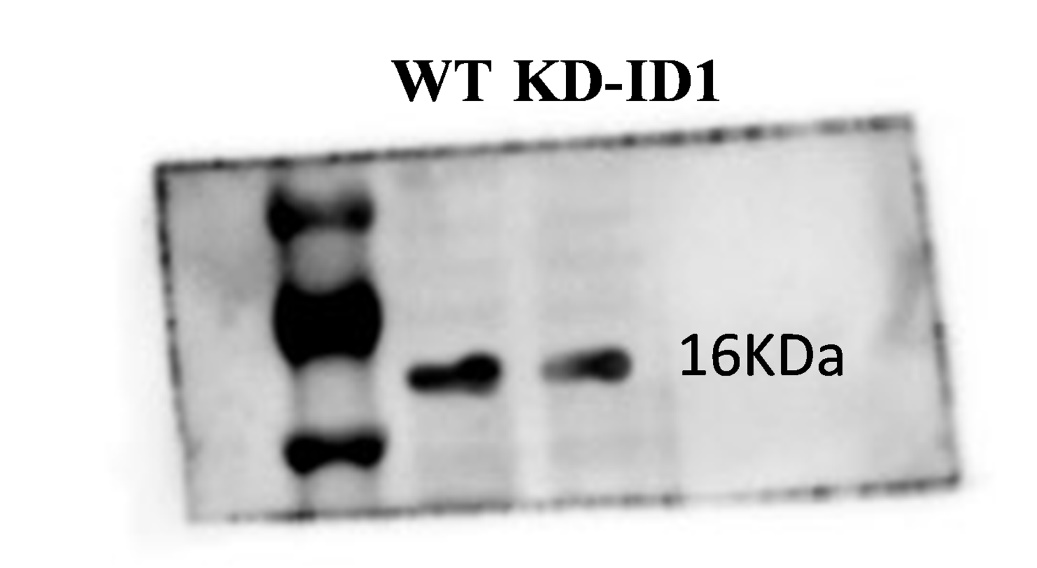


**β-actin (Cell Repeat·1)**


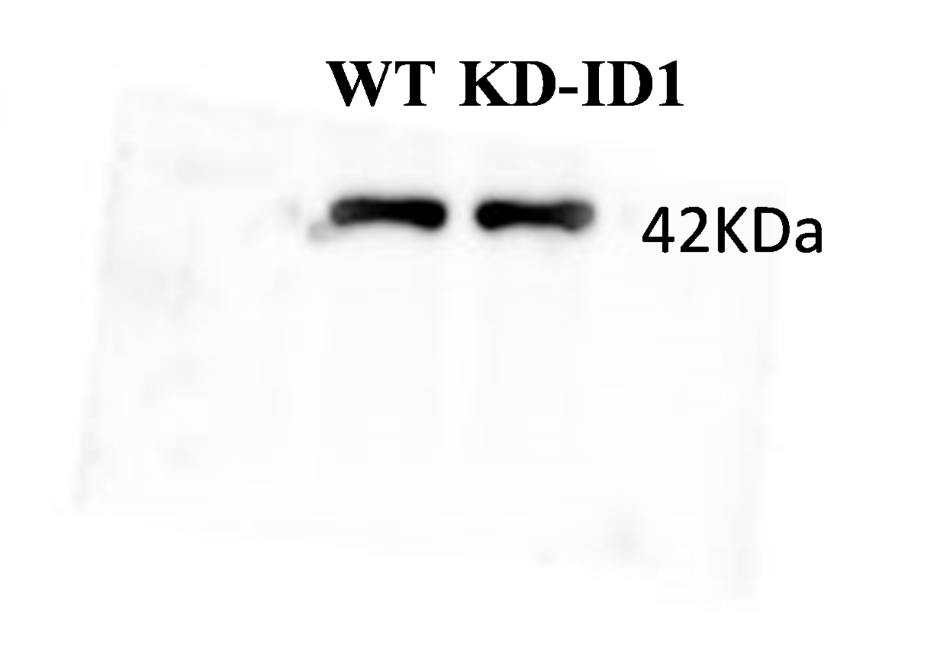


**β-actin (Cell Repeat·2)**


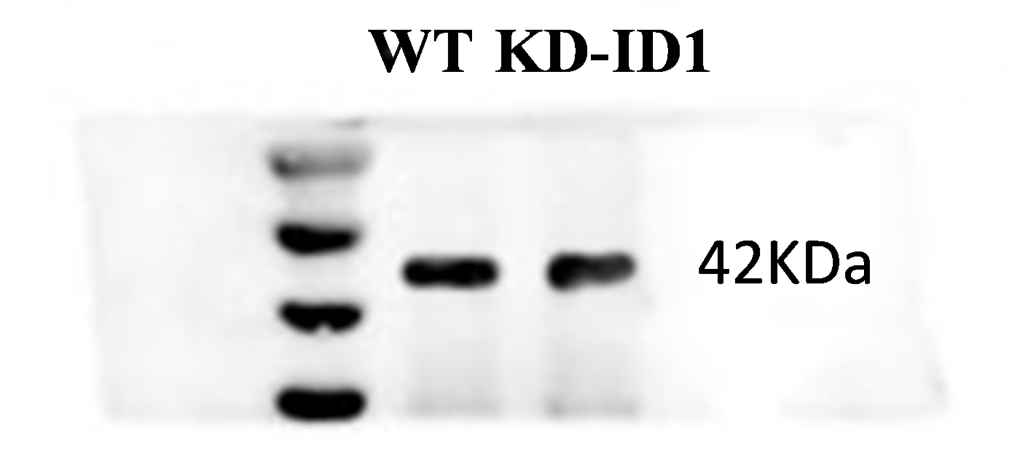


**β-actin (Cell Repeat·3)**


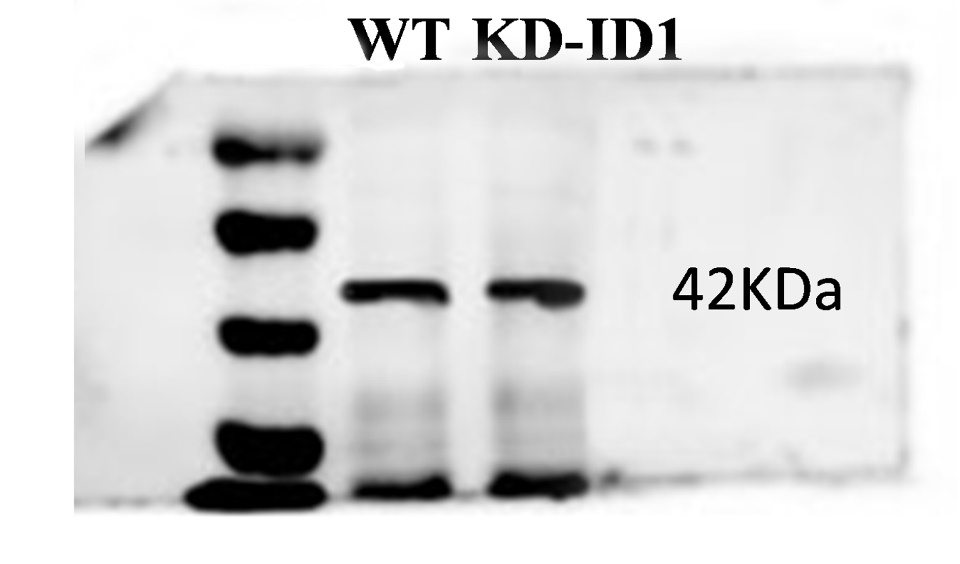


**KD-ID1 TSP1 (Cell Repeat·1)**


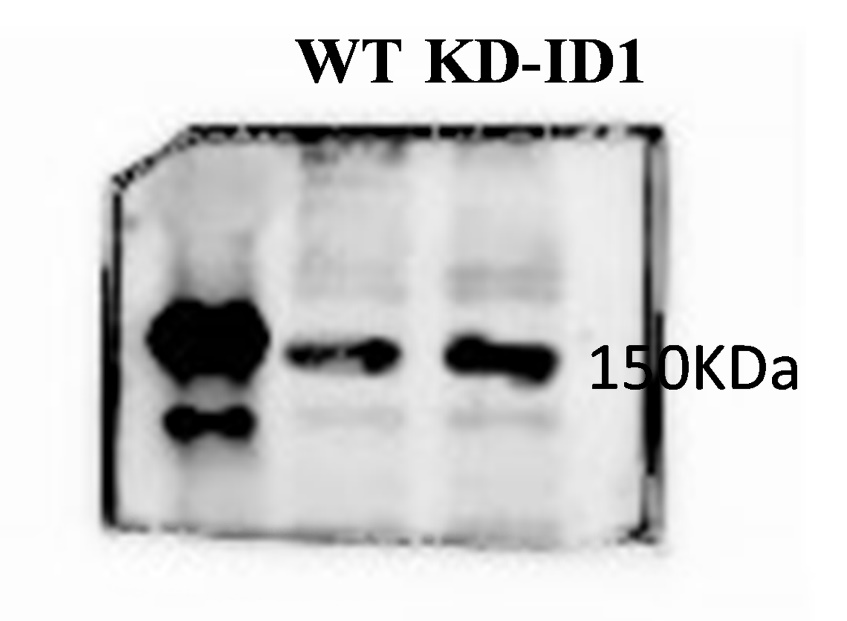


**KD-ID1 TSP1(Cell Repeat·2)**


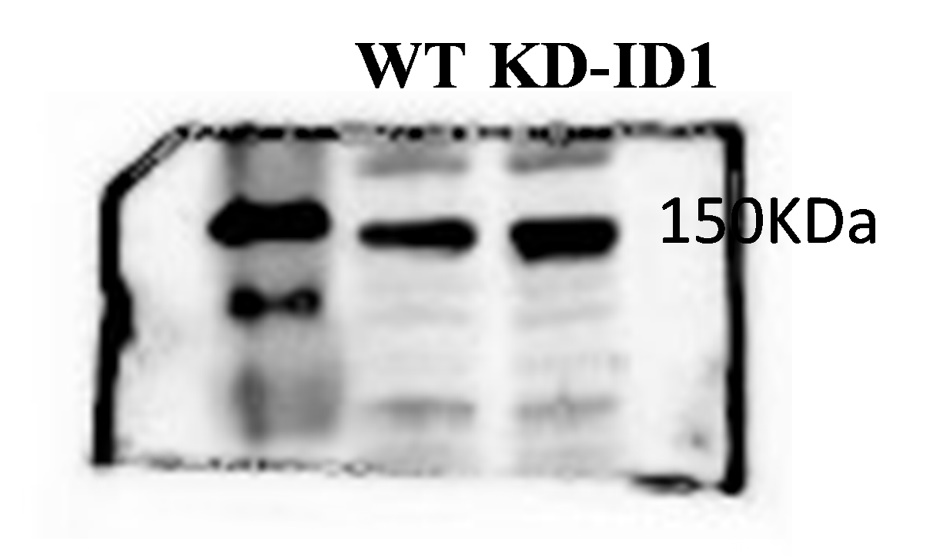


**KD-ID1 TSP1(Cell Repeat·3)**


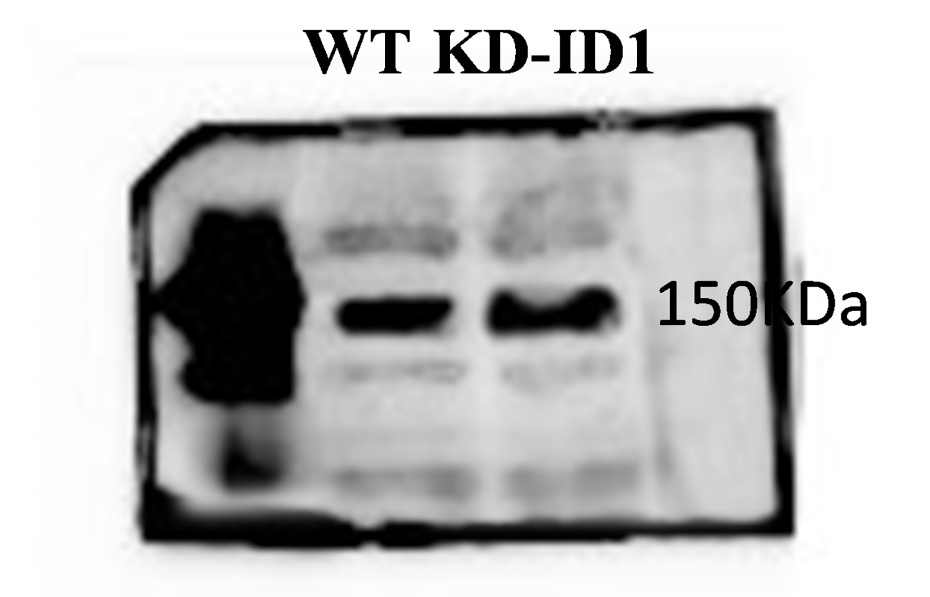


**β-actin (Cell Repeat·1)**


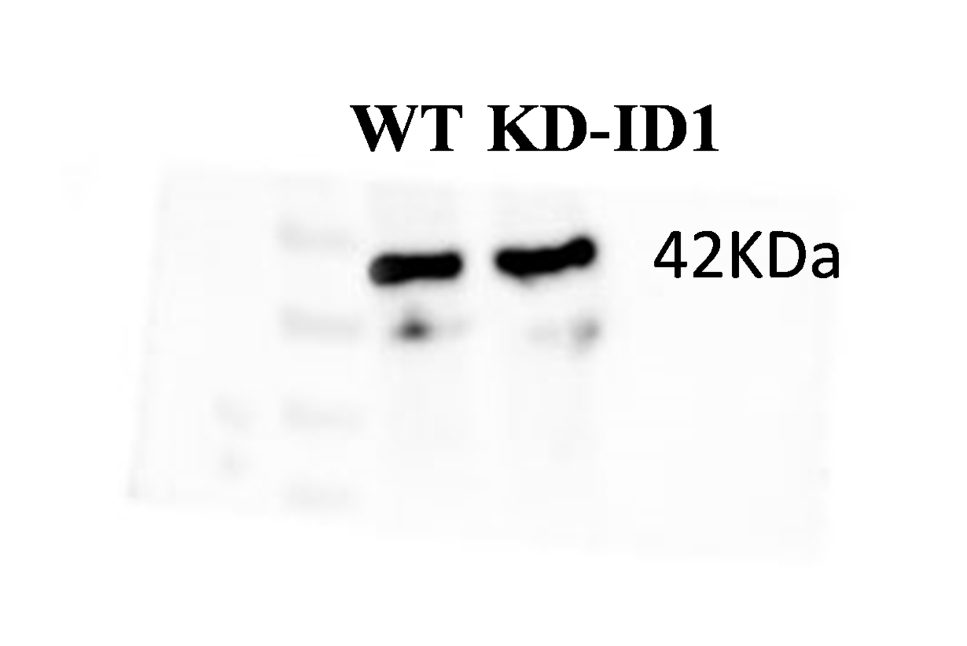


**β-actin (Cell Repeat·2)**


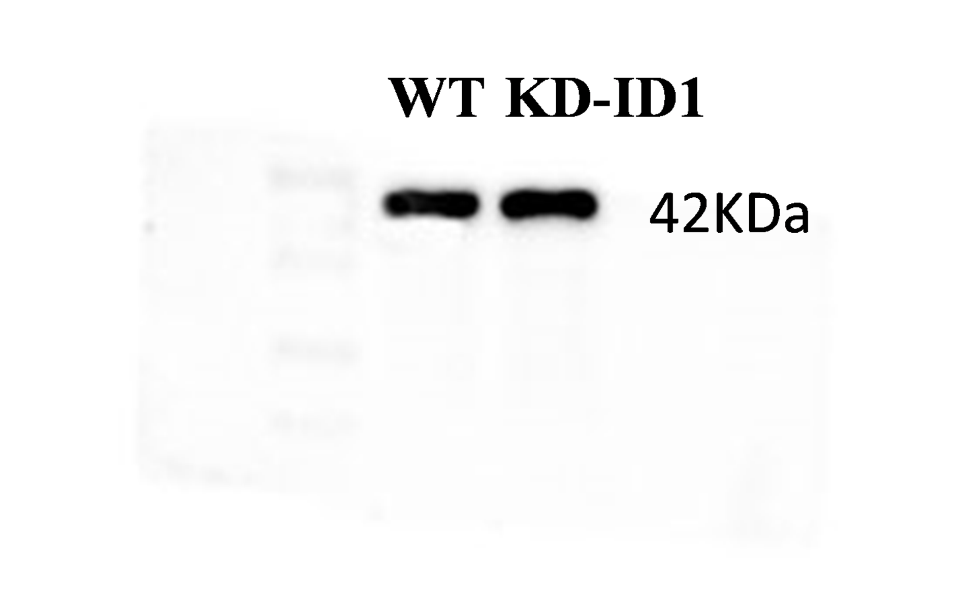


**β-actin (Cell Repeat·3)**


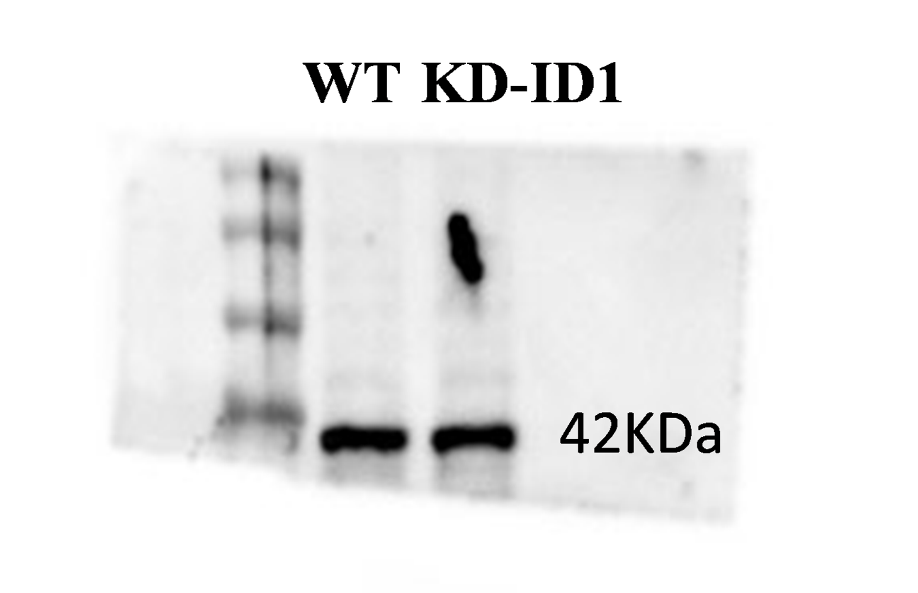

Supplement: Supplementary file 2 [file DataSheet1.docx]
